# Supplementary material for: Membrane environment sets the functional pKa of ionizable lipids
Source: Biophys J. 2026 Jun 26;125(15):3918–37. doi: 10.1016/j.bpj.2026.06.030 (PMC13507446; doi:10.1016/j.bpj.2026.06.030)
Supplement: Supplementary file 1 [file mmc1.pdf]

Biophysical Journal, Volume *125*

Supplemental information

Membrane environment sets the functional pK_a of ionizable lipids

Marius F.W. Trollmann, Paolo Rossetti, and Rainer A. Böckmann

Supplementary Material: Membrane Environment Sets the Functional pK_a of Ionizable Lipids

Marius F.W. Trollmann,[*,†,§] Paolo Rossetti,[†] and Rainer A. Böckmann[*,†,‡,¶,§]

†*Computational Biology, Department of Biology, Friedrich-Alexander-Universität Erlangen-Nürnberg, Erlangen, Germany*

‡*FAU Profile Center Immunomedicine (FAU I-MED), Erlangen, Germany*

¶*FAU Research Center New Bioactive Compounds – FAU NeW, Erlangen, Germany*

§*Erlangen National High-Performance Computing Center (NHR@FAU), Erlangen, Germany*

E-mail: marius.trollmann@fau.de; rainer.boeckmann@fau.de

Phone: +49 (0)9131 8525409

The PDF file includes

- Tables S1-S4
- Figures S1 – S105,
- References

Supplementary Tables

Table S1: All-atom molecular dynamics simulations using the constant-pH method to parametrize and validate $V^{MM}(\lambda)$.

System	Aminolipid:H_2O:Na^+:Cl^-:Buf	Sim. Time (μs)	pH	Objective
$DODAP_A$	1:6945:19:20:1	0.6	-	Thermodynamic Integration
$DLin\text{-}MC3\text{-}DMA_A$ [a]	1:6944:19:20:1	0.6	-	Thermodynamic Integration
$DLin\text{-}KC2\text{-}DMA_A$	1:6948:19:20:1	0.6	-	Thermodynamic Integration
$SM\text{-}102_A$	1:6941:19:20:1	1.7	-	Thermodynamic Integration
$ALC\text{-}0315_A$ [b]	1:6938:19:20:1	1.7	-	Thermodynamic Integration
$DODAP_B$	1:6944:19:20:3	0.6	-	Goodness of polynomial coefficients
$DLin\text{-}MC3\text{-}DMA_B$ [a]	1:6943:19:20:3	0.3	-	Goodness of polynomial coefficients
$DLin\text{-}KC2\text{-}DMA_B$	1:6947:19:20:3	0.6	-	Goodness of polynomial coefficients
$SM\text{-}102_B$	1:6940:19:20:3	1.0	-	Goodness of polynomial coefficients
$ALC\text{-}0315_B$ [b]	1:6937:19:20:3	1.7	-	Goodness of polynomial coefficients
$DODAP_C$	1:6944:19:20:3	10×0.6	5–14	Intrinsic pK_a
$DLin\text{-}MC3\text{-}DMA_C$ [a]	1:6943:19:20:3	10×0.3	5–14	Intrinsic pK_a
$DLin\text{-}KC2\text{-}DMA_C$	1:6947:19:20:3	10×0.6	5–14	Intrinsic pK_a
$SM\text{-}102_C$	1:6940:19:20:3	10×1.0	5–14	Intrinsic pK_a
$ALC\text{-}0315_C$ [b]	1:6937:19:20:3	10×1.0	4.17–14.17	Intrinsic pK_a

[a] Equivalent to.[1]
[b] Equivalent to[2]

Table S2: All-atom molecular dynamics simulations of ternary membranes including the helperlipid DOPC using the constant-pH method.

System	Aminolipid:DOPC:Chol	H_2O:Na^+:Cl^-:Buf	Sim. Time (μs)	pH	Objective
$DODAP_A$	48:144:48	11903:0:48:97	0.5	3–11	Apparent pK_a
$DLin\text{-}MC3\text{-}DMA_A$[a]	48:144:48	11903:0:48:97	0.5	3–11	Apparent pK_a
$DLin\text{-}KC2\text{-}DMA_A$	48:144:48	11903:0:48:97	0.5	3–11	Apparent pK_a
$SM\text{-}102_A$	48:144:48	11317:0:48:97	0.5	3–11	Apparent pK_a
$ALC\text{-}0315_A$	48:144:48	11833:0:48:97	0.5	3.17–14.17	Apparent pK_a
$DODAP_A$	48:96:96	11903:0:48:97	0.5	4–8	Apparent pK_a
$DLin\text{-}MC3\text{-}DMA_A$	48:96:96	11903:0:48:97	0.5	6–10	Apparent pK_a
$DLin\text{-}KC2\text{-}DMA_A$	48:96:96	11903:0:48:97	0.5	4–8	Apparent pK_a
$SM\text{-}102_A$	48:96:96	11855:0:48:97	0.5	5–9	Apparent pK_a
$ALC\text{-}0315_A$	48:96:96	11855:0:48:97	0.5	5–9	Apparent pK_a
$DODAP_B$	48:144:48	11903:0:48/48/44/27/11/3/0/0/0:24	0.5	3–11	Apparent pK_a
$DLin\text{-}MC3\text{-}DMA_B$	48:144:48	11903:0:48/48/48/45/31/15/2/0/0:22	0.5	3–11	Apparent pK_a
$DLin\text{-}KC2\text{-}DMA_B$	48:144:48	11903:0:48/48/46/34/9/3/0/0/0:12	0.5	3–11	Apparent pK_a
$SM\text{-}102_B$	48:144:48	11317:0:48/48/48/45/30/9/4/1/0:26	0.5	3–11	Apparent pK_a
$ALC\text{-}0315_B$	48:144:48	11833:0:34/13/4/2/0/0/0/0/0:18	0.5	3.17–14.17	Apparent pK_a
$DODAP_B$	48:96:96	11903:0:48/46/29/9/0:26	0.5	4–8	Apparent pK_a
$DLin\text{-}MC3\text{-}DMA_B$	48:96:96	11903:0:47/36/11/1/0:20	0.5	6–10	Apparent pK_a
$DLin\text{-}KC2\text{-}DMA_B$	48:96:96	11903:0:48/43/33/12/1:14	0.5	4–8	Apparent pK_a
$SM\text{-}102_B$	48:96:96	11855:0:47/42/24/8/4:22	0.5	5–9	Apparent pK_a
$ALC\text{-}0315_B$	48:96:96	11855:0:46/32/17/2/0:22	0.5	5–9	Apparent pK_a
$DLin\text{-}MC3\text{-}DMA_C$	48:144:48	11903:0:48/48/48/45/31/15/2/0/0:22	1.0	3–11	Apparent pK_a
$DLin\text{-}KC2\text{-}DMA_C$	48:144:48	11903:0:48/48/46/34/9/3/0/0/0:12	1.0	3–11	Apparent pK_a
$SM\text{-}102_C$	48:144:48	11317:0:48/48/48/45/30/9/4/1/0:26	1.0	3–11	Apparent pK_a
$ALC\text{-}0315_C$	48:144:48	11833:0:34/13/4/2/0/0/0/0/0:18	1.0	3.17–14.17	Apparent pK_a
$DLin\text{-}MC3\text{-}DMA_C$	48:96:96	11903:0:47/36/11/1/0:20	1.0	6–10	Apparent pK_a
$DLin\text{-}KC2\text{-}DMA_C$	48:96:96	11903:0:48/43/33/12/1:14	1.0	4–8	Apparent pK_a
$SM\text{-}102_C$	48:96:96	11855:0:47/42/24/8/4:22	1.0	5–9	Apparent pK_a
$ALC\text{-}0315_C$	48:96:96	11855:0:46/32/17/2/0:22	1.0	5–9	Apparent pK_a

[a] Equivalent to[2]

Table S3: All-atom molecular dynamics simulations of ternary membranes including the helperlipid DSPC using the constant-pH method.

System	Aminolipid:DSPC:Chol	H_2O:Na^+:Cl^-:Buf	Sim. Time (μs)	pH	Objective
$DODAP_A$	48:144:48	11903:0:48:97	0.5	3–11	Apparent pK_a
DLin-MC3-DMA_A	48:144:48	11903:0:48:97	0.5	3–11	Apparent pK_a
DLin-KC2-DMA_A	48:144:48	11903:0:48:97	0.5	3–11	Apparent pK_a
SM-102_A	48:144:48	11317:0:48:97	0.5	3–11	Apparent pK_a
ALC-0315_A	48:144:48	11833:0:48:97	0.5	3–11	Apparent pK_a
$DODAP_A$	48:96:96	11903:0:48:97	0.5	4–8	Apparent pK_a
DLin-MC3-DMA_A	48:96:96	11903:0:48:97	0.5	6–10	Apparent pK_a
DLin-KC2-DMA_A	48:96:96	11903:0:48:97	0.5	4–8	Apparent pK_a
SM-102_A	48:96:96	11855:0:48:97	0.5	5–9	Apparent pK_a
ALC-0315_A	48:96:96	11855:0:48:97	0.5	5–9	Apparent pK_a
$DODAP_B$	48:144:48	11903:0:48/48/44/27/11/3/0/0/0:24	0.5	3–11	Apparent pK_a
DLin-MC3-DMA_B	48:144:48	11903:0:48/48/48/45/31/15/2/0/0:22	0.5	3–11	Apparent pK_a
DLin-KC2-DMA_B	48:144:48	11903:0:48/48/46/34/9/3/0/0/0:12	0.5	3–11	Apparent pK_a
SM-102_B	48:144:48	11317:0:48/48/48/45/30/9/4/1/0:26	0.5	3–11	Apparent pK_a
ALC-0315_B	48:144:48	11833:0:34/13/4/2/0/0/0/0/0:18	0.5	3–11	Apparent pK_a
$DODAP_B$	48:96:96	11903:0:48/46/29/9/0:26	0.5	4–8	Apparent pK_a
DLin-MC3-DMA_B	48:96:96	11903:0:47/36/11/1/0:20	0.5	6–10	Apparent pK_a
DLin-KC2-DMA_B	48:96:96	11903:0:48/43/33/12/1:14	0.5	4–8	Apparent pK_a
SM-102_B	48:96:96	11855:0:47/42/24/8/4:22	0.5	5–9	Apparent pK_a
ALC-0315_B	48:96:96	11855:0:46/32/17/2/0:22	0.5	5–9	Apparent pK_a
DLin-MC3-DMA_C	48:144:48	11903:0:48/48/48/45/31/15/2/0/0:22	1.0	3–11	Apparent pK_a
DLin-KC2-DMA_C	48:144:48	11903:0:48/48/46/34/9/3/0/0/0:12	1.0	3–11	Apparent pK_a
SM-102_C	48:144:48	11317:0:48/48/48/45/30/9/4/1/0:26	1.0	3–11	Apparent pK_a
ALC-0315_C	48:144:48	11833:0:34/13/4/2/0/0/0/0/0:18	1.0	3–11	Apparent pK_a
DLin-MC3-DMA_C	48:96:96	11903:0:47/36/11/1/0:20	1.0	6–10	Apparent pK_a
DLin-KC2-DMA_C	48:96:96	11903:0:48/43/33/12/1:14	1.0	4–8	Apparent pK_a
SM-102_C	48:96:96	11855:0:47/42/24/8/4:22	1.0	5–9	Apparent pK_a
ALC-0315_C	48:96:96	11855:0:46/32/17/2/0:22	1.0	5–9	Apparent pK_a

Table S4: All-atom molecular dynamics simulations of ternary membranes including the helperlipid DOPC using the constant-pH method. The simulations were started with a different initialization by inserting all aminolipids in a deprotonated state into the membrane core.

System	Aminolipid:DOPC:Chol	H_2O:Na^+:Cl^-:Buf	Sim. Time (μs)	pH	Objective
SM-102	48:144:48	11903:0:0:97	2.0	3	Core initialization
SM-102	48:144:48	11903:0:0:97	2.0	5	Core initialization
SM-102	48:144:48	11910:0:0:90	2.0	7	Core initialization
SM-102	48:144:48	11972:0:0:28	2.0	9	Core initialization
ALC-0315	48:144:48	11903:0:0:97	2.0	3	Core initialization
ALC-0315	48:144:48	11903:0:0:97	2.0	5	Core initialization
ALC-0315	48:144:48	11943:0:0:57	2.0	7	Core initialization
ALC-0315	48:144:48	11980:0:0:20	2.0	9	Core initialization
SM-102	48:96:96	11903:0:0:97	2.0	3	Core initialization
SM-102	48:96:96	11903:0:0:97	2.0	5	Core initialization
SM-102	48:96:96	11920:0:0:80	2.0	7	Core initialization
SM-102	48:96:96	11974:0:0:26	2.0	9	Core initialization
ALC-0315	48:96:96	11903:0:0:97	2.0	3	Core initialization
ALC-0315	48:96:96	11903:0:0:97	2.0	5	Core initialization
ALC-0315	48:96:96	11949:0:0:51	2.0	7	Core initialization
ALC-0315	48:96:96	11979:0:0:21	2.0	9	Core initialization

Supplementary Figures

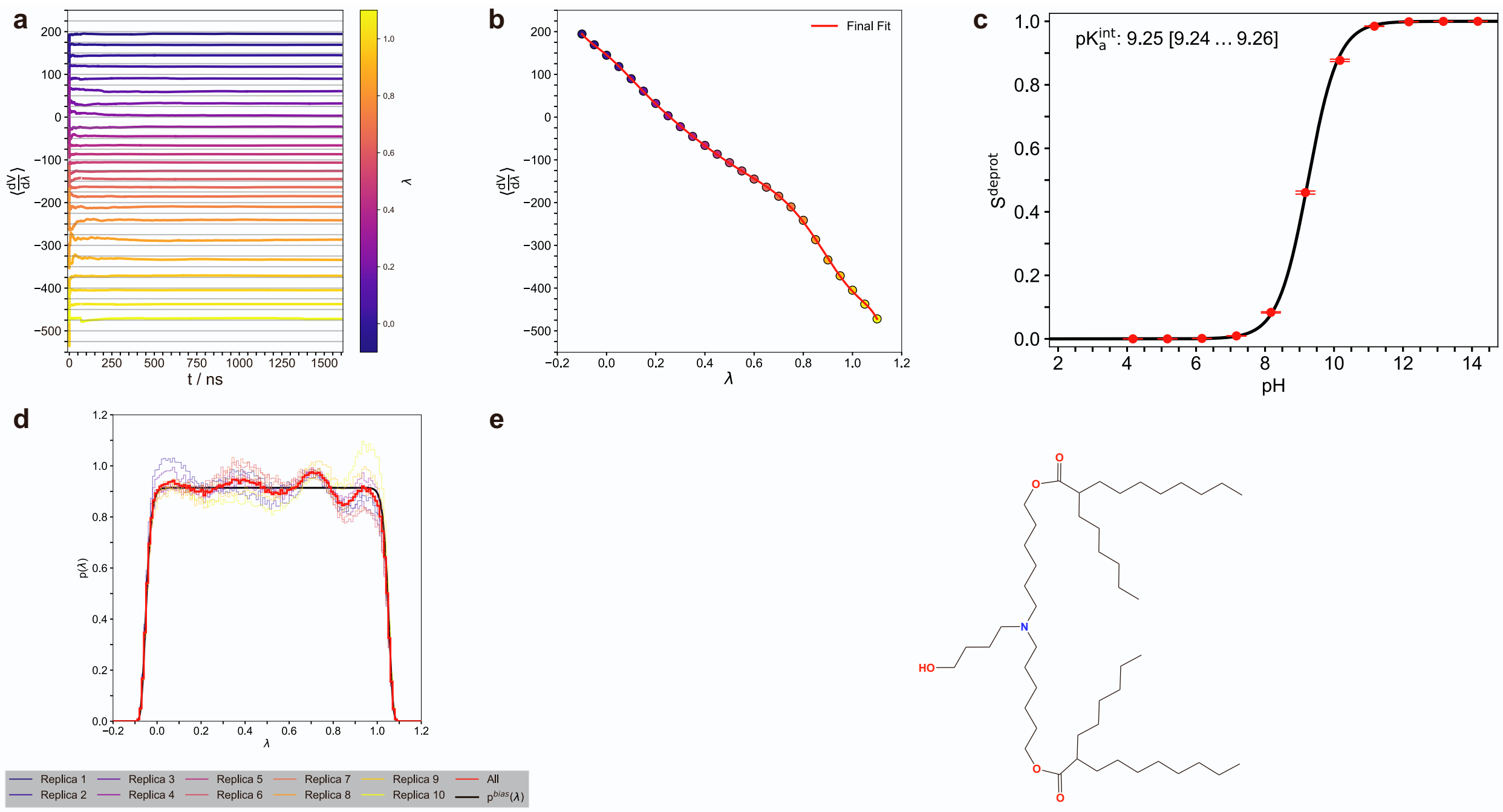

Figure S1: **a, b** Parameterization and **c, d** validation of polynomial coefficients used to model the correction potential for the protonation free energy of the **e** aminolipid ALC-0315. **a** Convergence of the estimated averages for $\frac{\partial V^{MM}}{\partial \lambda}$ obtained from thermodynamic integration simulations. **b** Fit of a ninth-order polynomial to the estimates of $\langle \frac{\partial V^{MM}}{\partial \lambda} \rangle$. **c** Titration curve of the aminolipid at infinite dilution, fitted with the Henderson-Hasselbalch equation. Error bars represent the standard error of the mean over ten replicas per pH level. The λ-dependent potentials $V^{bias}(\lambda)$ (barrier height 7.5 kJ·mol^{-1}), $V^{pH}(\lambda)$ (corresponding to the respective pH value), and the parameterized $V^{MM}(\lambda)$ were applied. **d** Distributions of the λ-coordinate obtained from ten replica simulations of the aminolipid at infinite dilution. In this case, only $V^{bias}(\lambda)$ (barrier height 0.0 kJ·mol^{-1}) and the parameterized $V^{MM}(\lambda)$ were applied. **e** Molecular structure of the aminolipid.

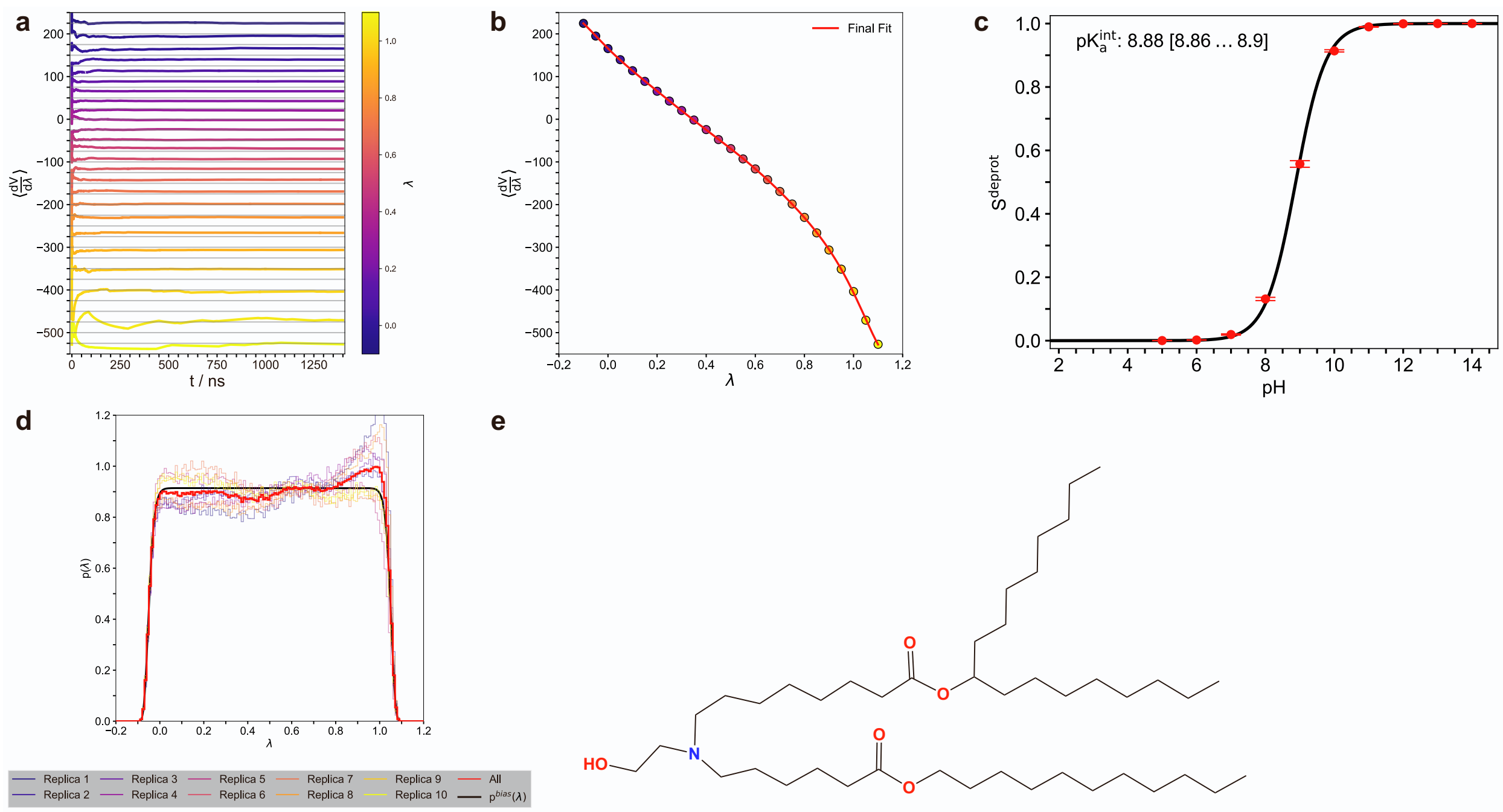

Figure S2: **a, b** Parameterization and **c, d** validation of polynomial coefficients used to model the correction potential for the protonation free energy of the **e** aminolipid SM-102. **a** Convergence of the estimated averages for $\frac{\partial V^{MM}}{\partial \lambda}$ obtained from thermodynamic integration simulations. **b** Fit of a ninth-order polynomial to the estimates of $\langle \frac{\partial V^{MM}}{\partial \lambda} \rangle$. **c** Titration curve of the aminolipid at infinite dilution, fitted with the Henderson-Hasselbalch equation. Error bars represent the standard error of the mean over ten replicas per pH level. The λ-dependent potentials $V^{bias}(\lambda)$ (barrier height 7.5 kJ·mol^{-1}), $V^{pH}(\lambda)$ (corresponding to the respective pH value), and the parameterized $V^{MM}(\lambda)$ were applied. **d** Distributions of the λ-coordinate obtained from ten replica simulations of the aminolipid at infinite dilution. In this case, only $V^{bias}(\lambda)$ (barrier height 0.0 kJ·mol^{-1}) and the parameterized $V^{MM}(\lambda)$ were applied. **e** Molecular structure of the aminolipid.

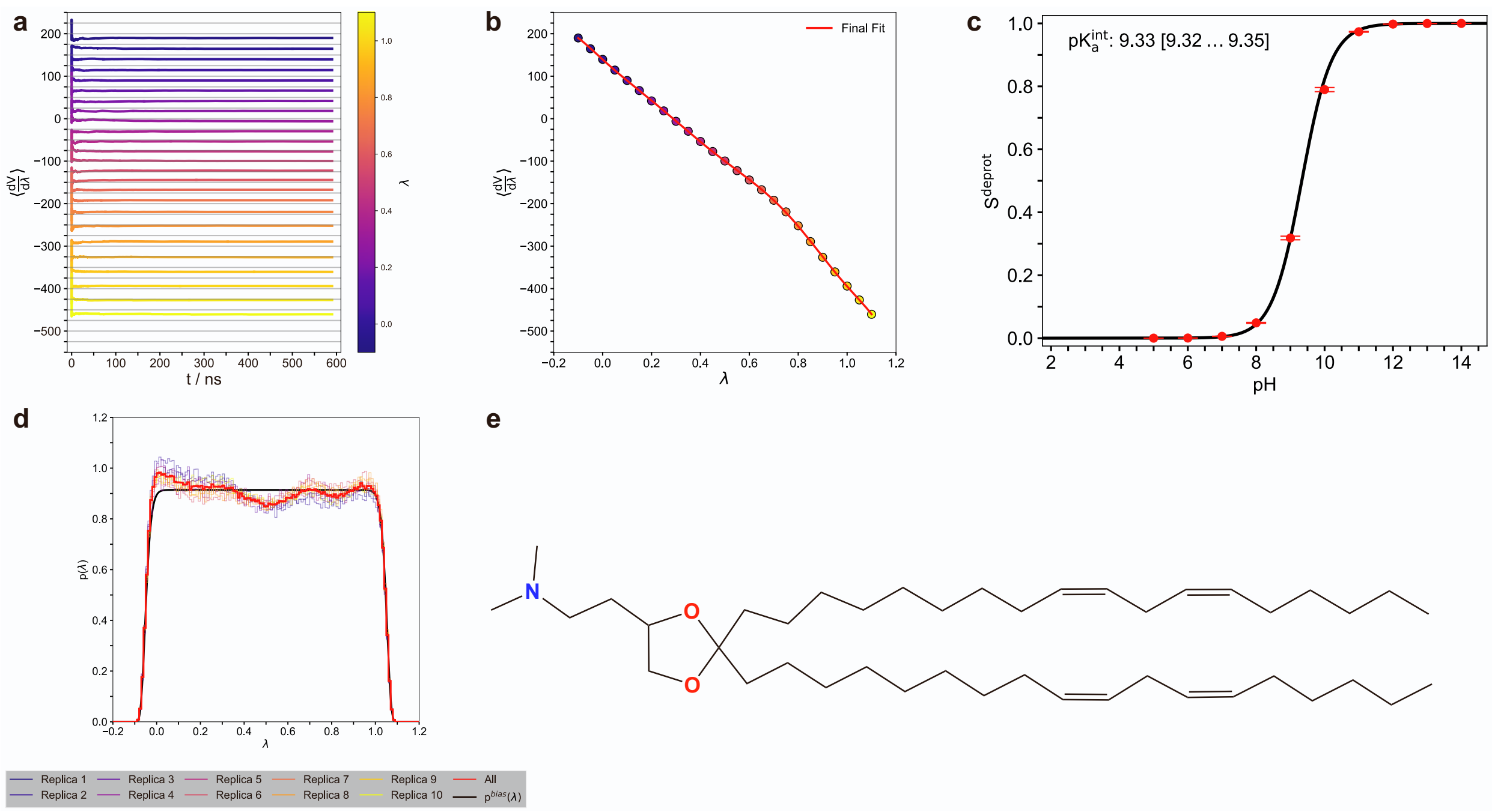

Figure S3: **a, b** Parameterization and **c, d** validation of polynomial coefficients used to model the correction potential for the protonation free energy of the **e** aminolipid DLin-KC2-DMA. **a** Convergence of the estimated averages for $\frac{\partial V^{MM}}{\partial \lambda}$ obtained from thermodynamic integration simulations. **b** Fit of an eighth-order polynomial to the estimates of $\langle \frac{\partial V^{MM}}{\partial \lambda} \rangle$. **c** Titration curve of the aminolipid at infinite dilution, fitted with the Henderson-Hasselbalch equation. Error bars represent the standard error of the mean over ten replicas per pH level. The λ-dependent potentials $V^{bias}(\lambda)$ (barrier height $7.5\,\text{kJ}\cdot\text{mol}^{-1}$), $V^{pH}(\lambda)$ (corresponding to the respective pH value), and the parameterized $V^{MM}(\lambda)$ were applied. **d** Distributions of the λ-coordinate obtained from ten replica simulations of the aminolipid at infinite dilution. In this case, only $V^{bias}(\lambda)$ (barrier height $0.0\,\text{kJ}\cdot\text{mol}^{-1}$) and the parameterized $V^{MM}(\lambda)$ were applied. **e** Molecular structure of the aminolipid.

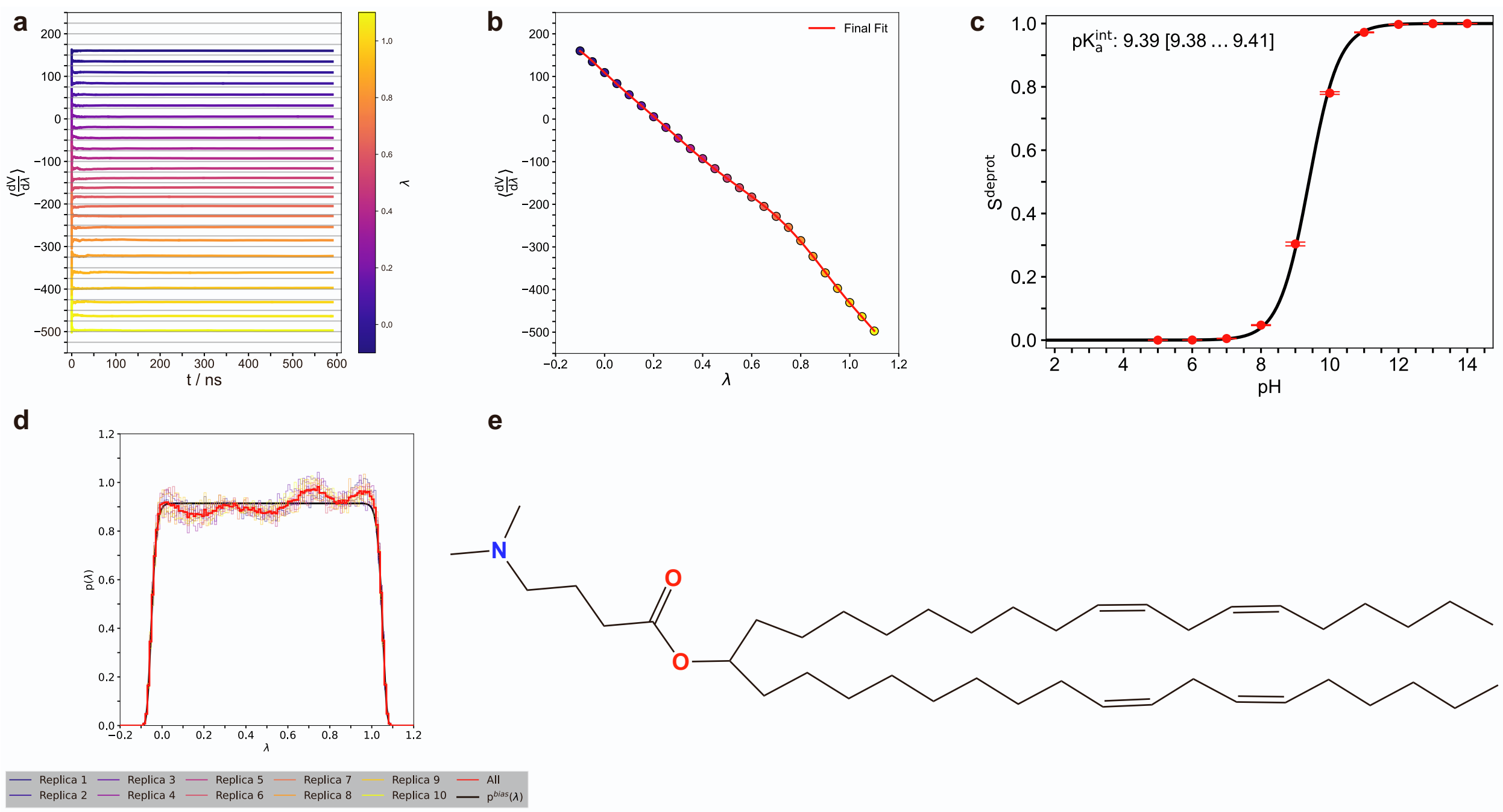

Figure S4: **a, b** Parameterization and **c, d** validation of polynomial coefficients used to model the correction potential for the protonation free energy of the **e** aminolipid DLin-MC3-DMA. **a** Convergence of the estimated averages for $\frac{\partial V^{MM}}{\partial \lambda}$ obtained from thermodynamic integration simulations. **b** Fit of an eighth-order polynomial to the estimates of $\langle\frac{\partial V^{MM}}{\partial \lambda}\rangle$. **c** Titration curve of the aminolipid at infinite dilution, fitted with the Henderson-Hasselbalch equation. Error bars represent the standard error of the mean over ten replicas per pH level. The λ-dependent potentials $V^{bias}(\lambda)$ (barrier height 7.5 kJ·mol^{-1}), $V^{pH}(\lambda)$ (corresponding to the respective pH value), and the parameterized $V^{MM}(\lambda)$ were applied. **d** Distributions of the λ-coordinate obtained from ten replica simulations of the aminolipid at infinite dilution. In this case, only $V^{bias}(\lambda)$ (barrier height 0.0 kJ·mol^{-1}) and the parameterized $V^{MM}(\lambda)$ were applied. **e** Molecular structure of the aminolipid.

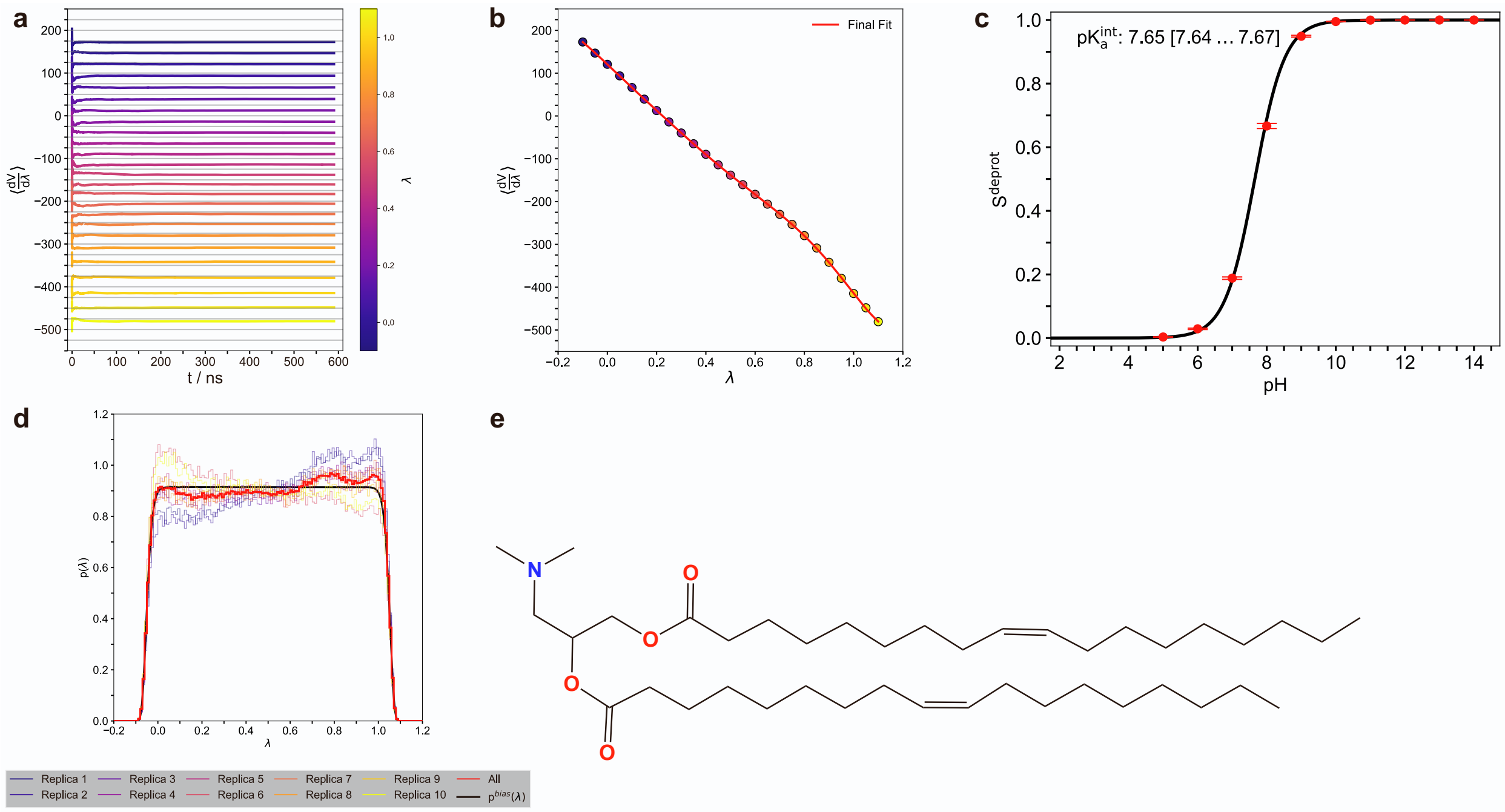

Figure S5: **a, b** Parameterization and **c, d** validation of polynomial coefficients used to model the correction potential for the protonation free energy of the **e** aminolipid DODAP. **a** Convergence of the estimated averages for $\frac{\partial V^{MM}}{\partial \lambda}$ obtained from thermodynamic integration simulations. **b** Fit of an eighth-order polynomial to the estimates of $\langle \frac{\partial V^{MM}}{\partial \lambda} \rangle$. **c** Titration curve of the aminolipid at infinite dilution, fitted with the Henderson-Hasselbalch equation. Error bars represent the standard error of the mean over ten replicas per pH level. The λ-dependent potentials $V^{bias}(\lambda)$ (barrier height 7.5 kJ·mol^{-1}), $V^{pH}(\lambda)$ (corresponding to the respective pH value), and the parameterized $V^{MM}(\lambda)$ were applied. **d** Distributions of the λ-coordinate obtained from ten replica simulations of the aminolipid at infinite dilution. In this case, only $V^{bias}(\lambda)$ (barrier height 0.0 kJ·mol^{-1}) and the parameterized $V^{MM}(\lambda)$ were applied. **e** Molecular structure of the aminolipid.

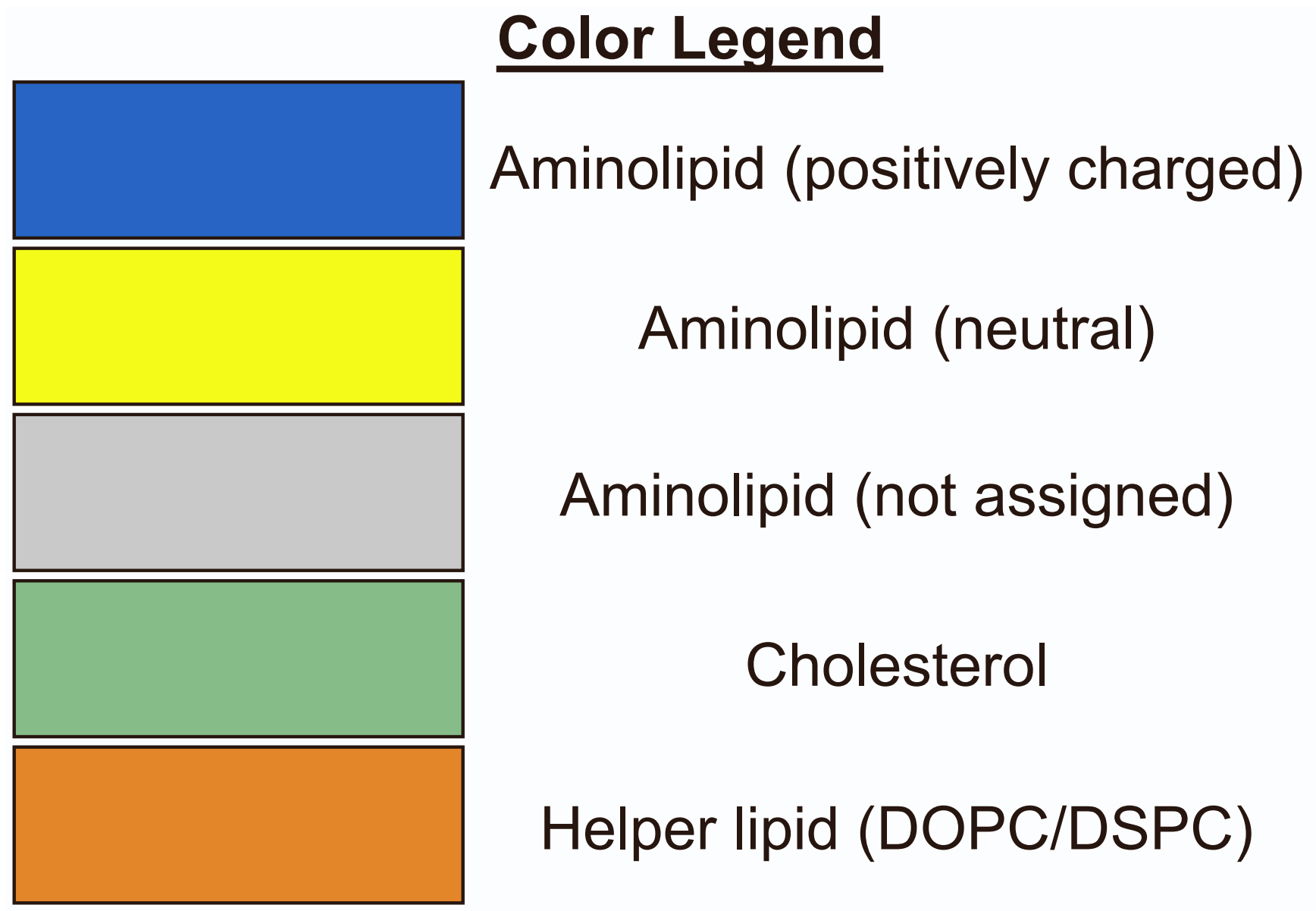

Figure S6: Color legend for all structural snapshots in the main text and Supporting Information. An aminolipid is classified as positively charged (blue) when its λ coordinate is below 0.2 (protonated) and as neutral (yellow) when λ exceeds 0.8 (deprotonated). For $0.2 \leq \lambda \leq 0.8$, the aminolipid is considered to be in a transition state (grey). Cholesterol is shown in green, and the helper lipids (DOPC/DSPC) in orange.

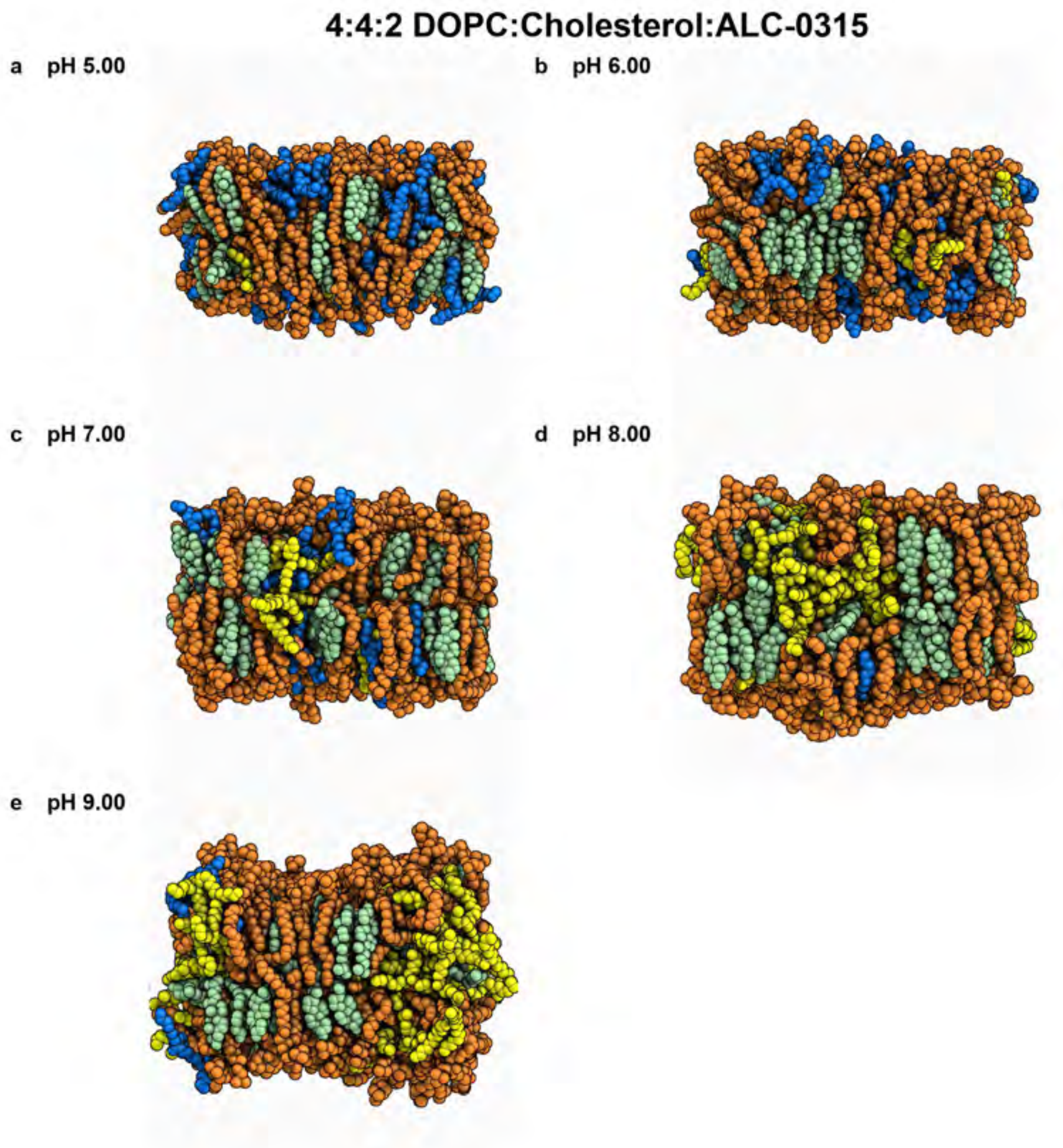

Figure S7: Side-view snapshots of the membrane in the final frame of the simulation at the corresponding pH values. The helper lipid is shown in orange, cholesterol in green, the protonated aminolipid (net charge +1) in blue, the deprotonated aminolipid (net charge 0) in yellow, and the aminolipids without a specific state ($0.2 \leq \lambda \leq 0.8$) in grey.

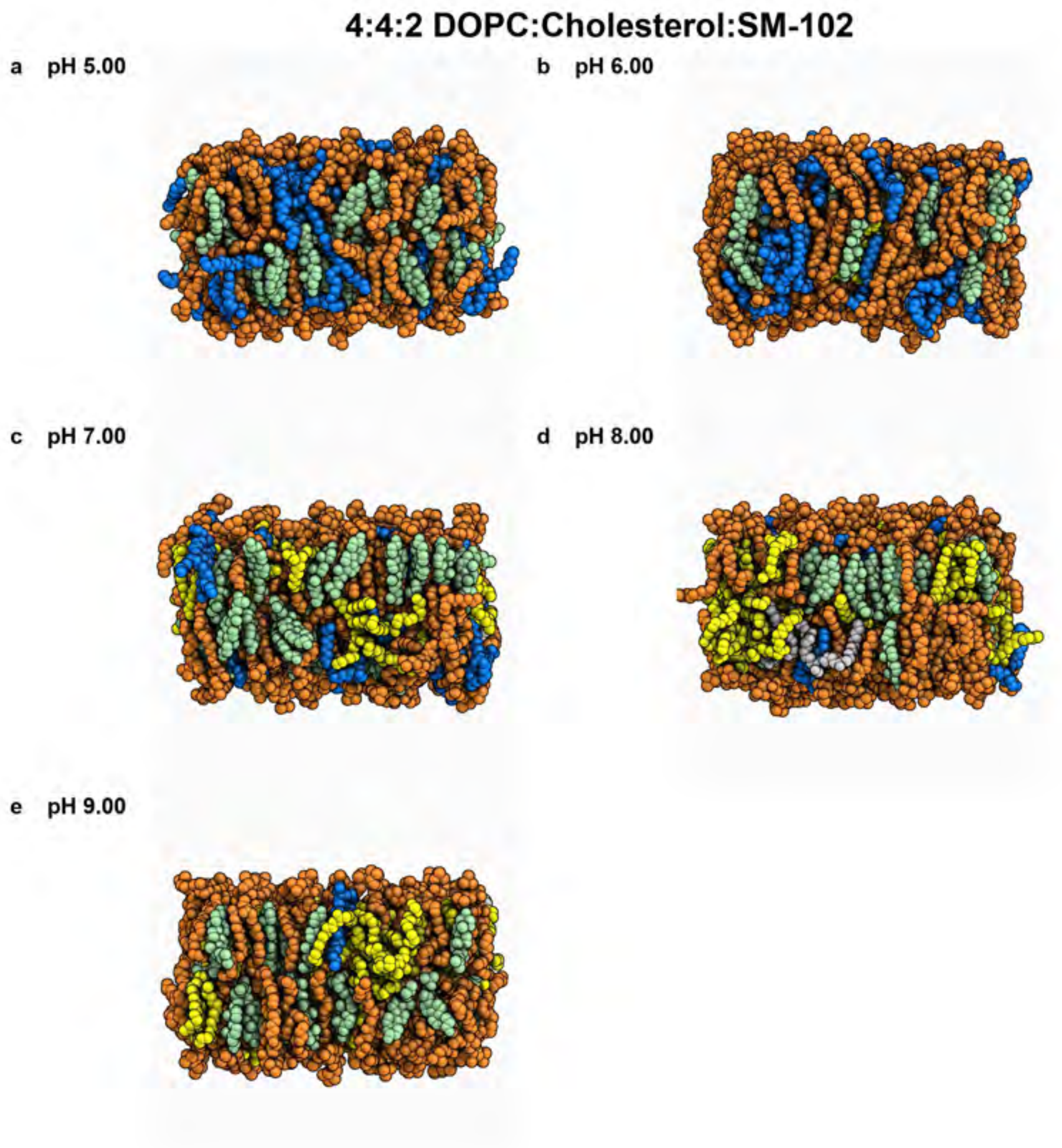

Figure S8: Side-view snapshots of the membrane in the final frame of the simulation at the corresponding pH values. The helper lipid is shown in orange, cholesterol in green, the protonated aminolipid (net charge +1) in blue, the deprotonated aminolipid (net charge 0) in yellow, and the aminolipids without a specific state ($0.2 \leq \lambda \leq 0.8$) in grey.

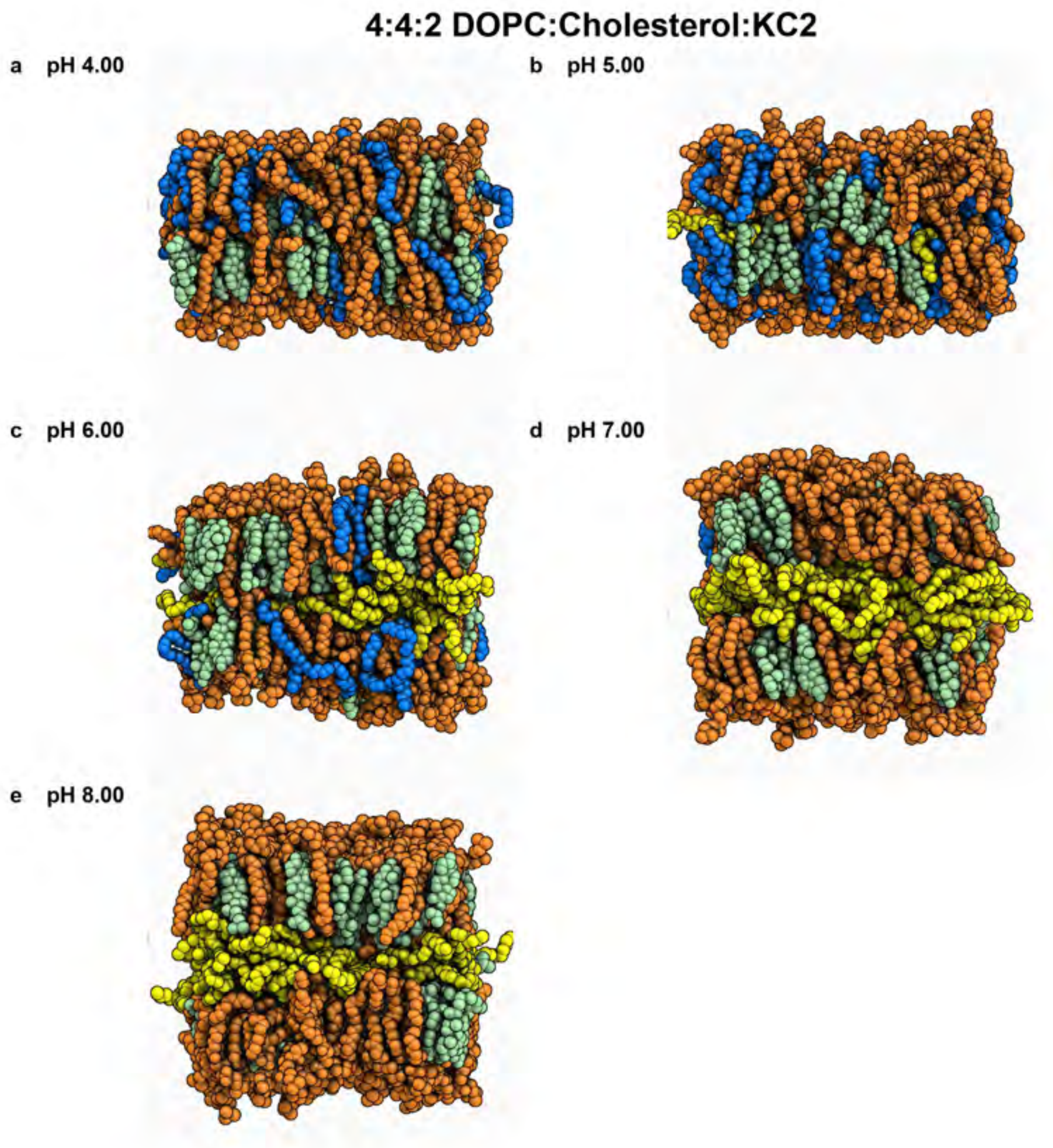

Figure S9: Side-view snapshots of the membrane in the final frame of the simulation at the corresponding pH values. The helper lipid is shown in orange, cholesterol in green, the protonated aminolipid (net charge +1) in blue, the deprotonated aminolipid (net charge 0) in yellow, and the aminolipids without a specific state ($0.2 \leq \lambda \leq 0.8$) in grey.

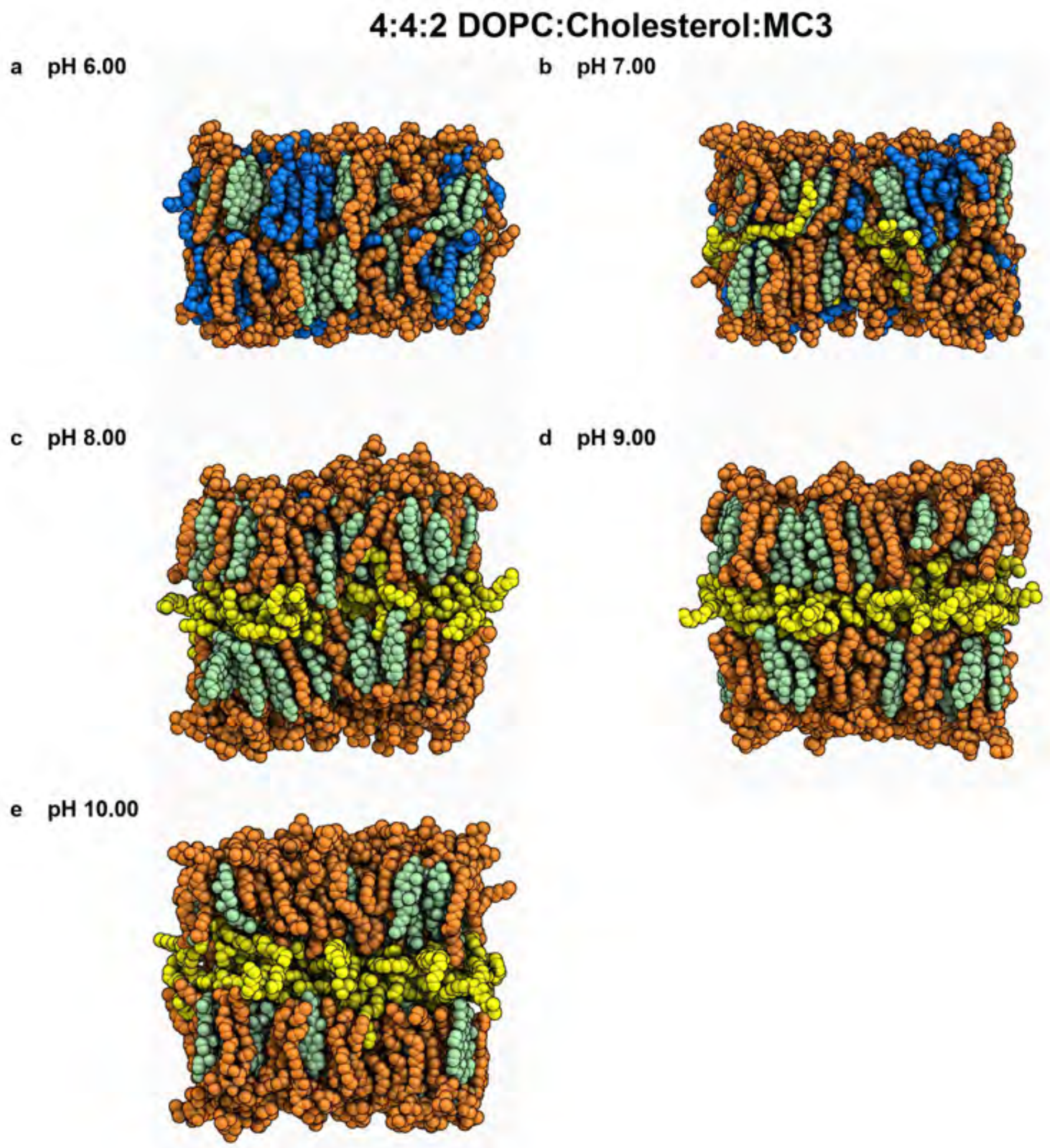

Figure S10: Side-view snapshots of the membrane in the final frame of the simulation at the corresponding pH values. The helper lipid is shown in orange, cholesterol in green, the protonated aminolipid (net charge +1) in blue, the deprotonated aminolipid (net charge 0) in yellow, and the aminolipids without a specific state ($0.2 \leq \lambda \leq 0.8$) in grey.

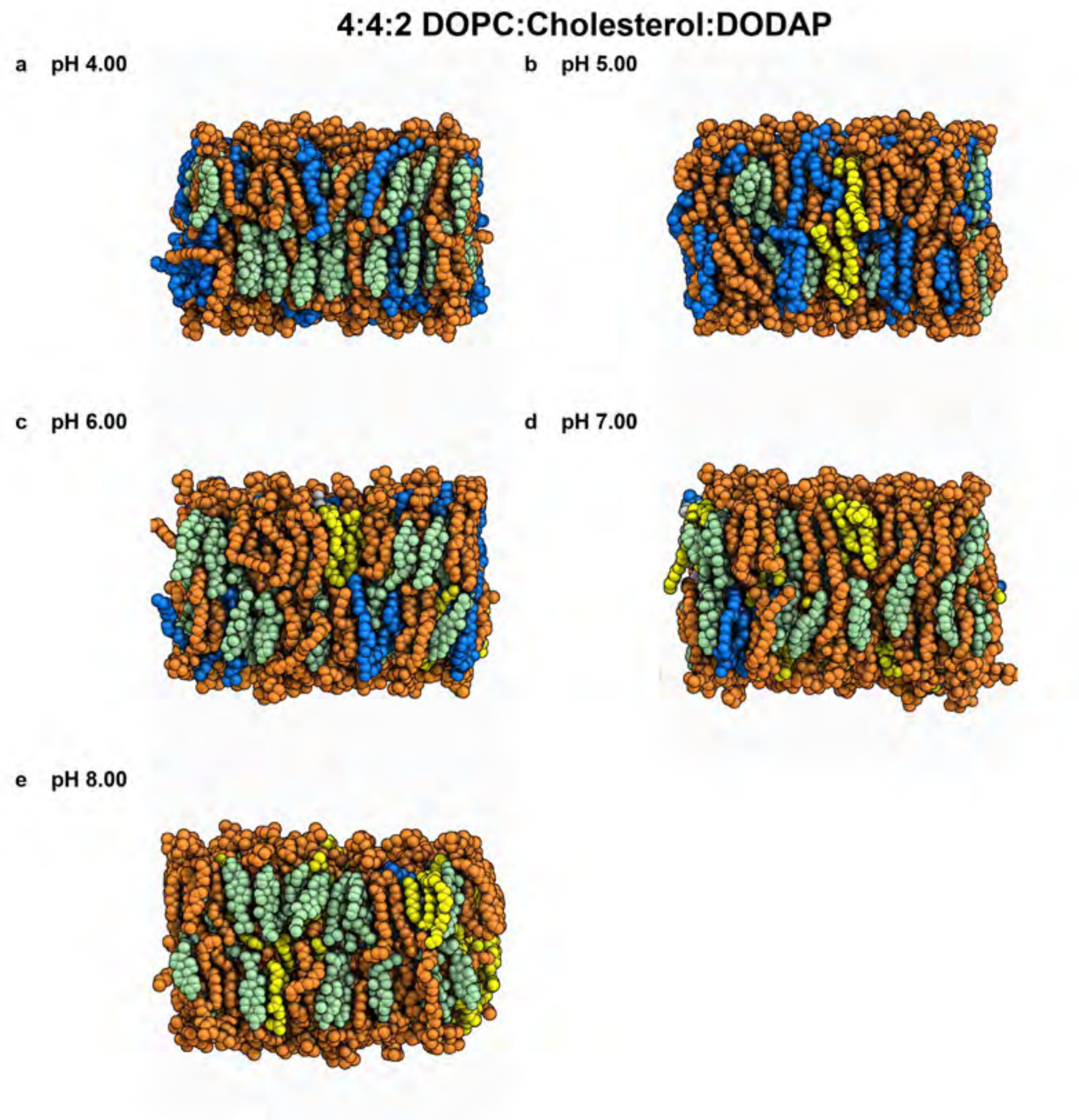

Figure S11: Side-view snapshots of the membrane in the final frame of the simulation at the corresponding pH values. The helper lipid is shown in orange, cholesterol in green, the protonated aminolipid (net charge +1) in blue, the deprotonated aminolipid (net charge 0) in yellow, and the aminolipids without a specific state ($0.2 \leq \lambda \leq 0.8$) in grey.

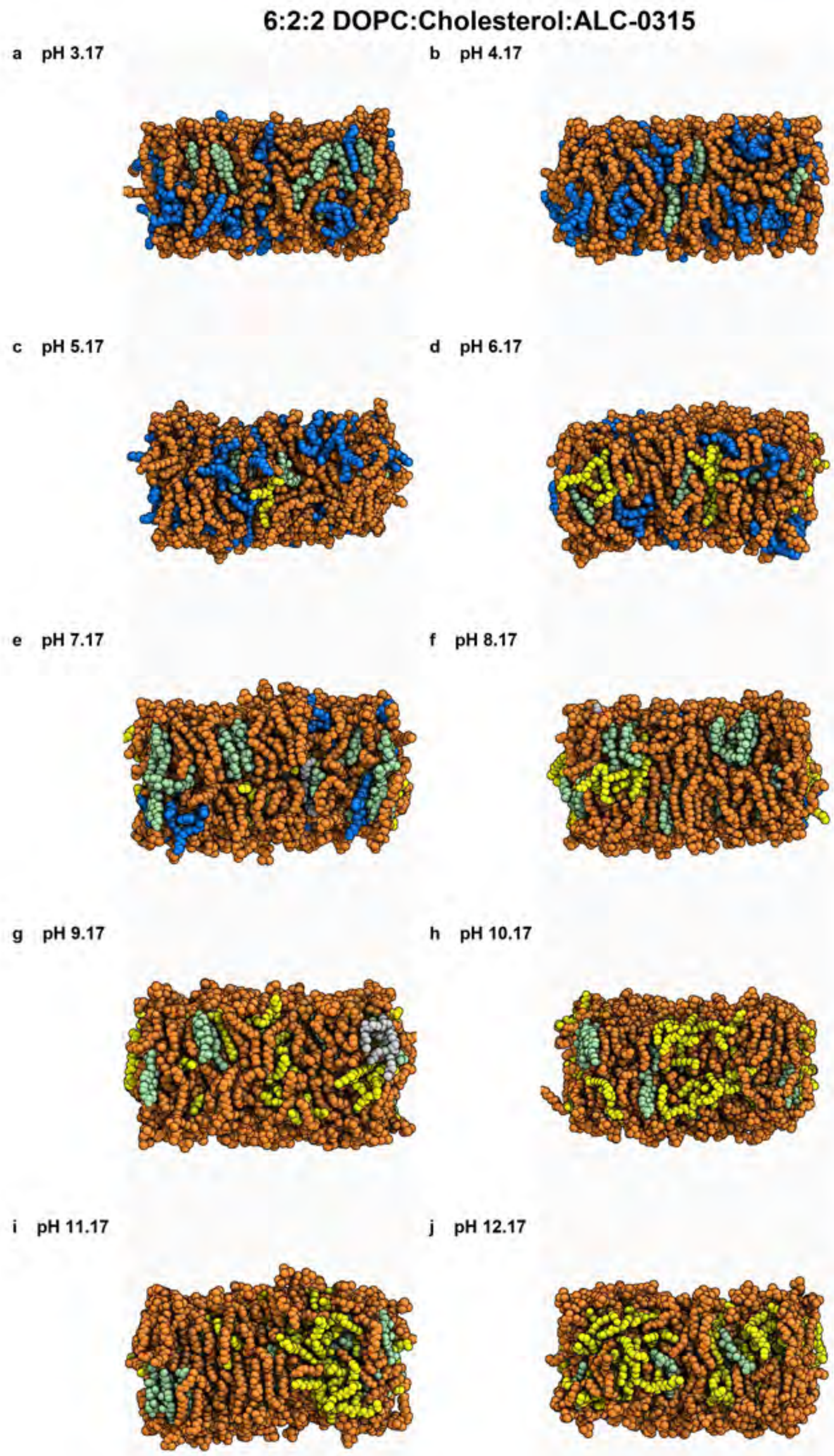

Figure S12: Side-view snapshots of the membrane in the final frame of the simulation at the corresponding pH values. The helper lipid is shown in orange, cholesterol in green, the protonated aminolipid (net charge +1) in blue, the deprotonated aminolipid (net charge 0) in yellow, and the aminolipids without a specific state ($0.2 \leq \lambda \leq 0.8$) in grey.

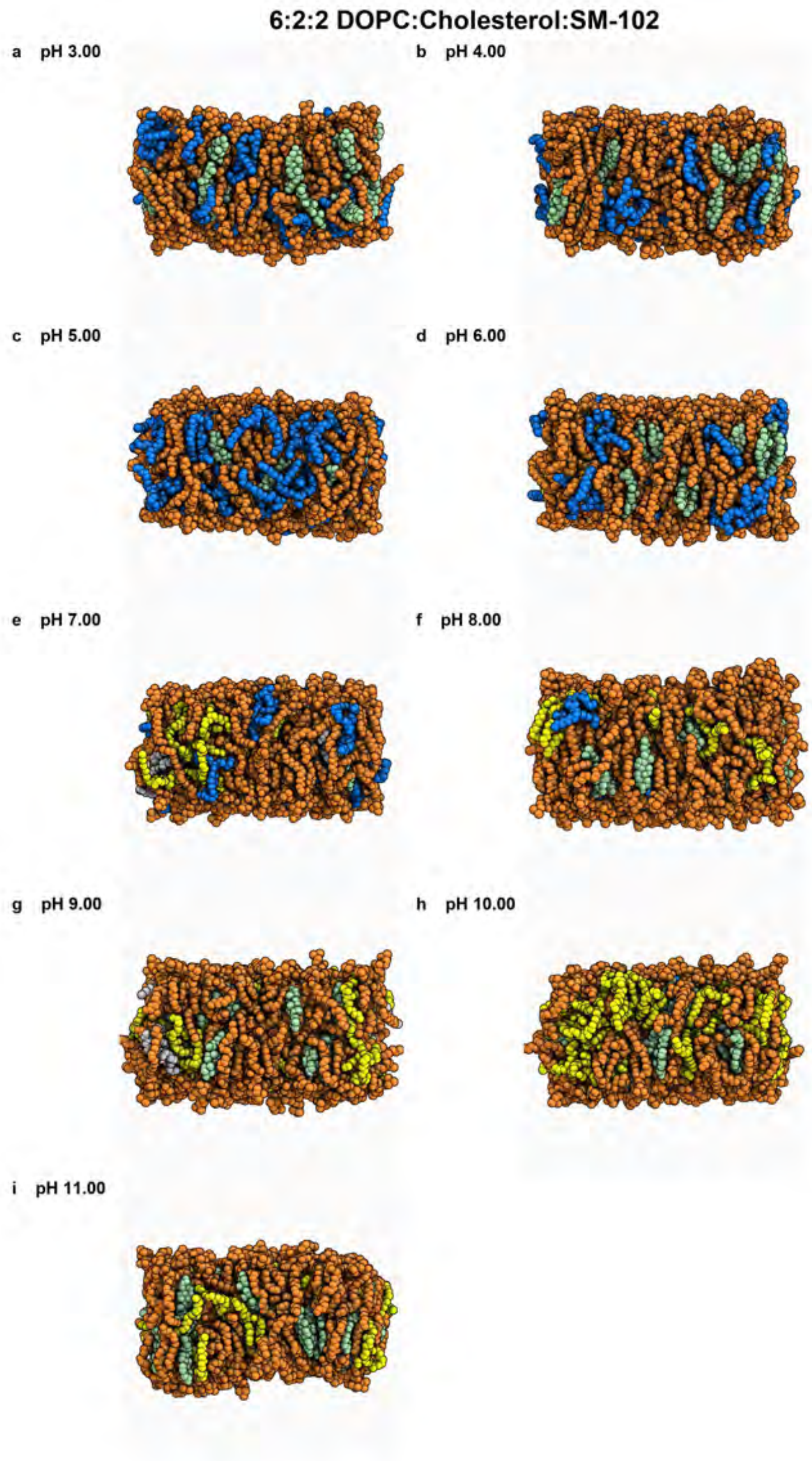

Figure S13: Side-view snapshots of the membrane in the final frame of the simulation at the corresponding pH values. The helper lipid is shown in orange, cholesterol in green, the protonated aminolipid (net charge +1) in blue, the deprotonated aminolipid (net charge 0) in yellow, and the aminolipids without a specific state ($0.2 \leq \lambda \leq 0.8$) in grey.

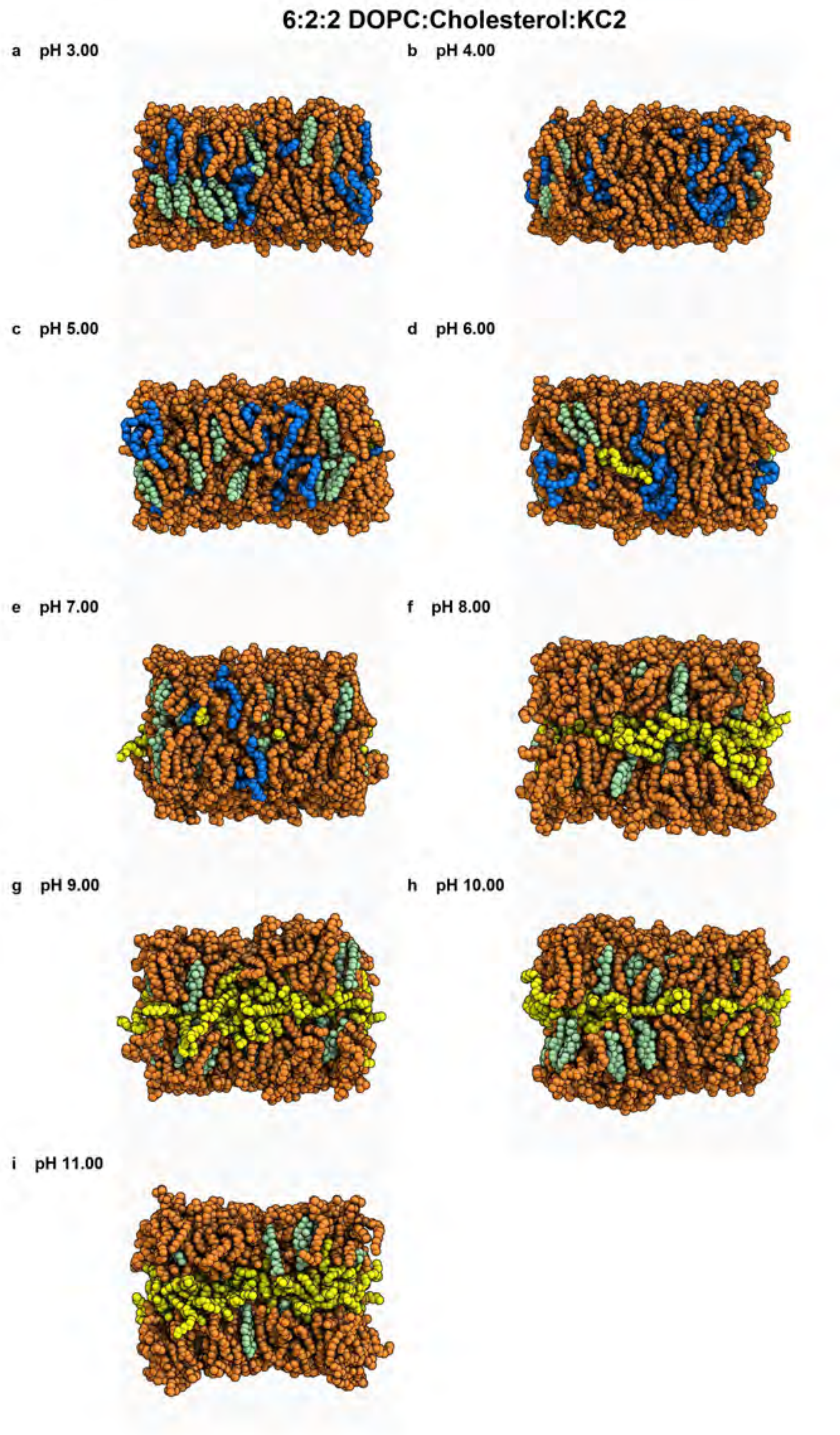

Figure S14: Side-view snapshots of the membrane in the final frame of the simulation at the corresponding pH values. The helper lipid is shown in orange, cholesterol in green, the protonated aminolipid (net charge +1) in blue, the deprotonated aminolipid (net charge 0) in yellow, and the aminolipids without a specific state ($0.2 \leq \lambda \leq 0.8$) in grey.

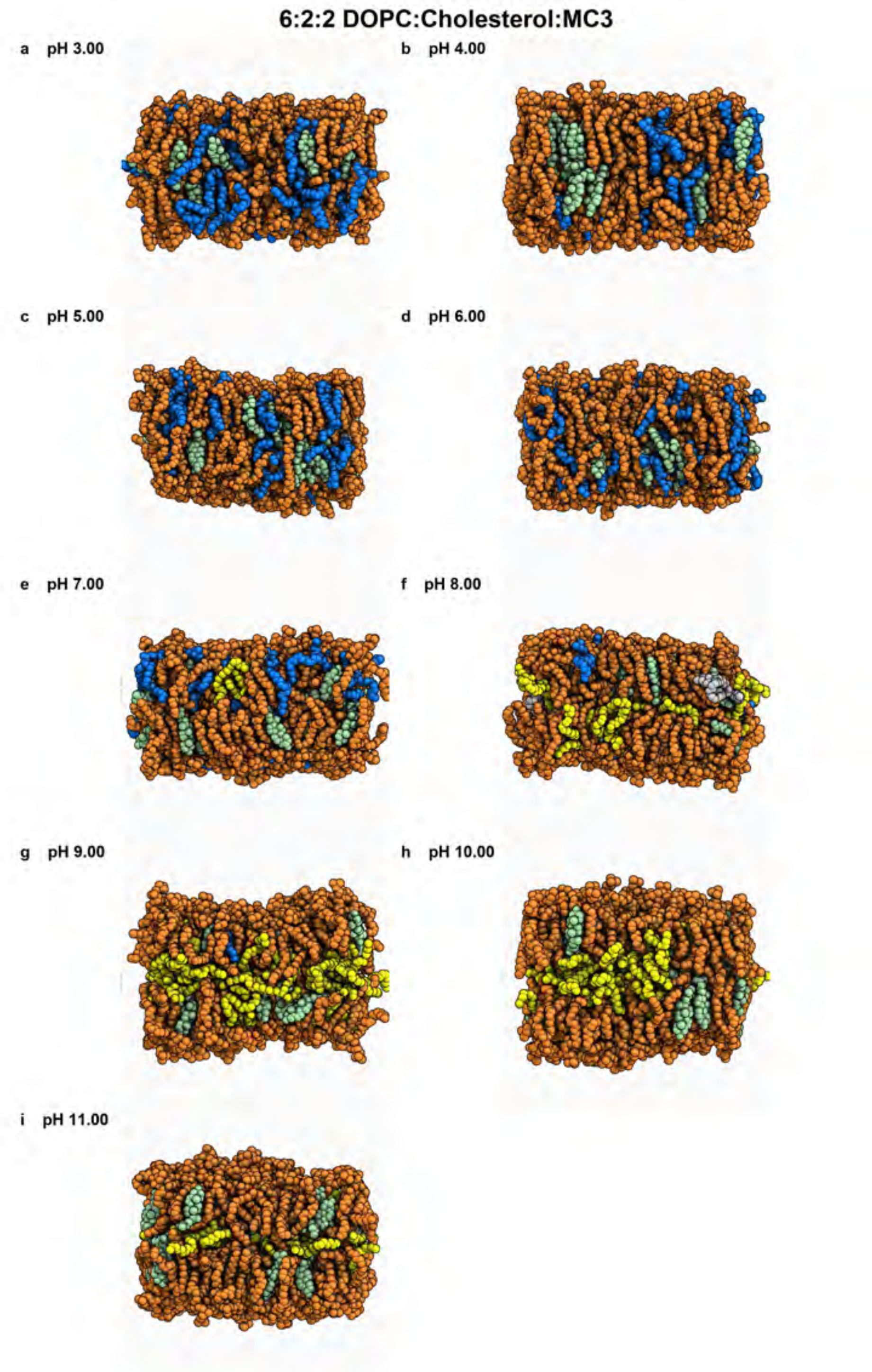

Figure S15: Side-view snapshots of the membrane in the final frame of the simulation at the corresponding pH values. The helper lipid is shown in orange, cholesterol in green, the protonated aminolipid (net charge +1) in blue, the deprotonated aminolipid (net charge 0) in yellow, and the aminolipids without a specific state ($0.2 \leq \lambda \leq 0.8$) in grey.

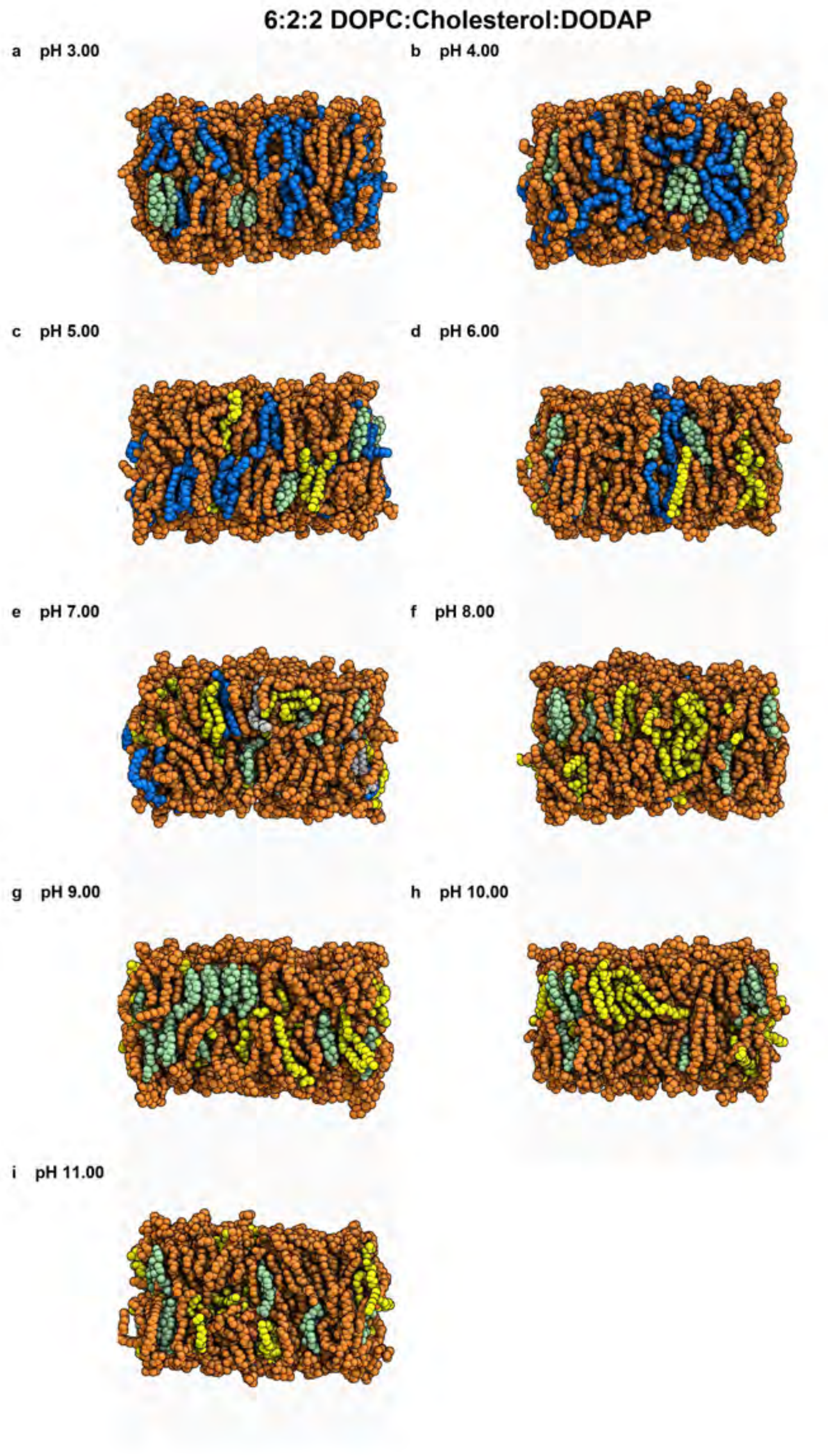

Figure S16: Side-view snapshots of the membrane in the final frame of the simulation at the corresponding pH values. The helper lipid is shown in orange, cholesterol in green, the protonated aminolipid (net charge +1) in blue, the deprotonated aminolipid (net charge 0) in yellow, and the aminolipids without a specific state ($0.2 \leq \lambda \leq 0.8$) in grey.

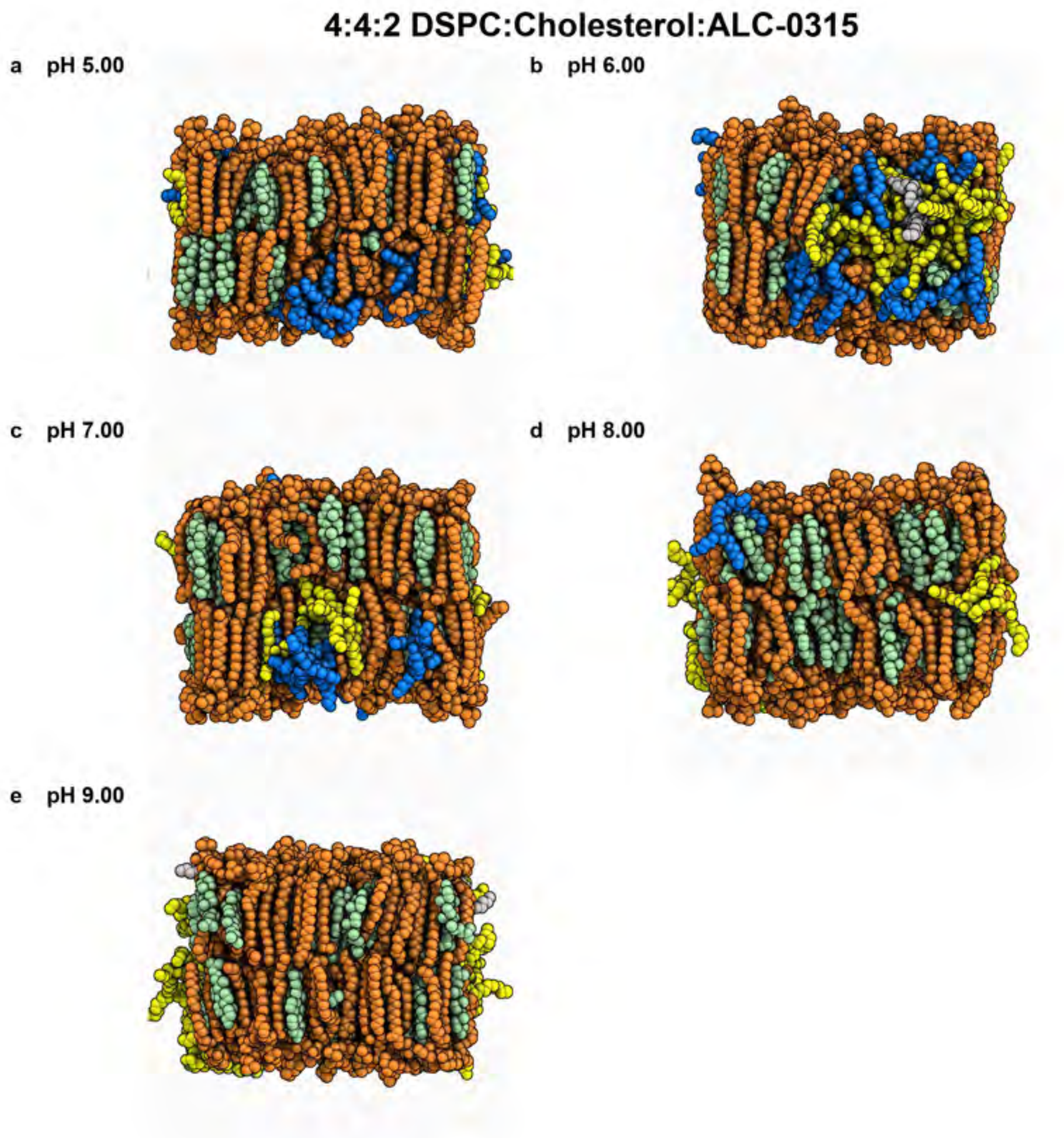

Figure S17: Side-view snapshots of the membrane in the final frame of the simulation at the corresponding pH values. The helper lipid is shown in orange, cholesterol in green, the protonated aminolipid (net charge +1) in blue, the deprotonated aminolipid (net charge 0) in yellow, and the aminolipids without a specific state ($0.2 \leq \lambda \leq 0.8$) in grey.

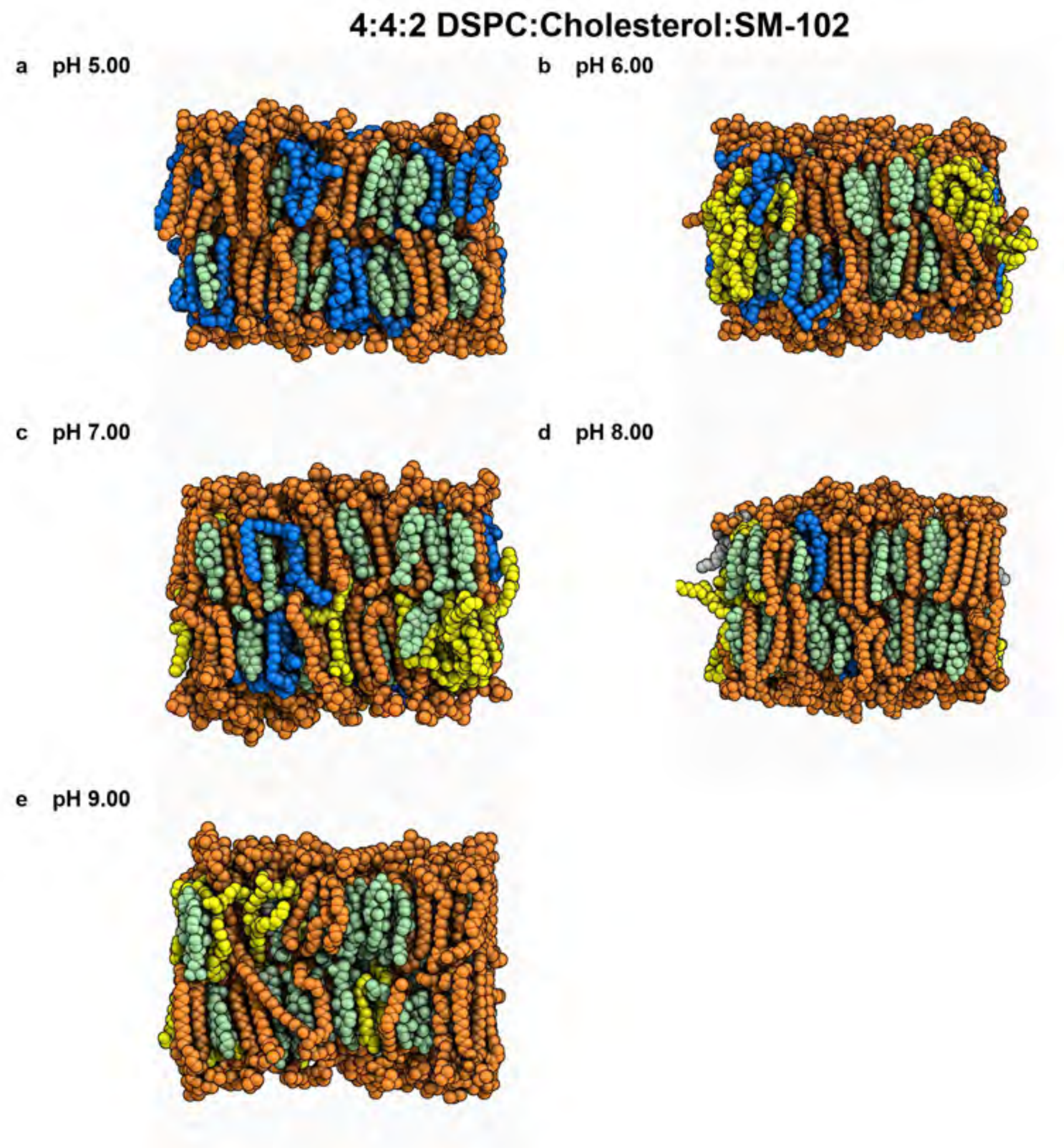

Figure S18: Side-view snapshots of the membrane in the final frame of the simulation at the corresponding pH values. The helper lipid is shown in orange, cholesterol in green, the protonated aminolipid (net charge +1) in blue, the deprotonated aminolipid (net charge 0) in yellow, and the aminolipids without a specific state ($0.2 \leq \lambda \leq 0.8$) in grey.

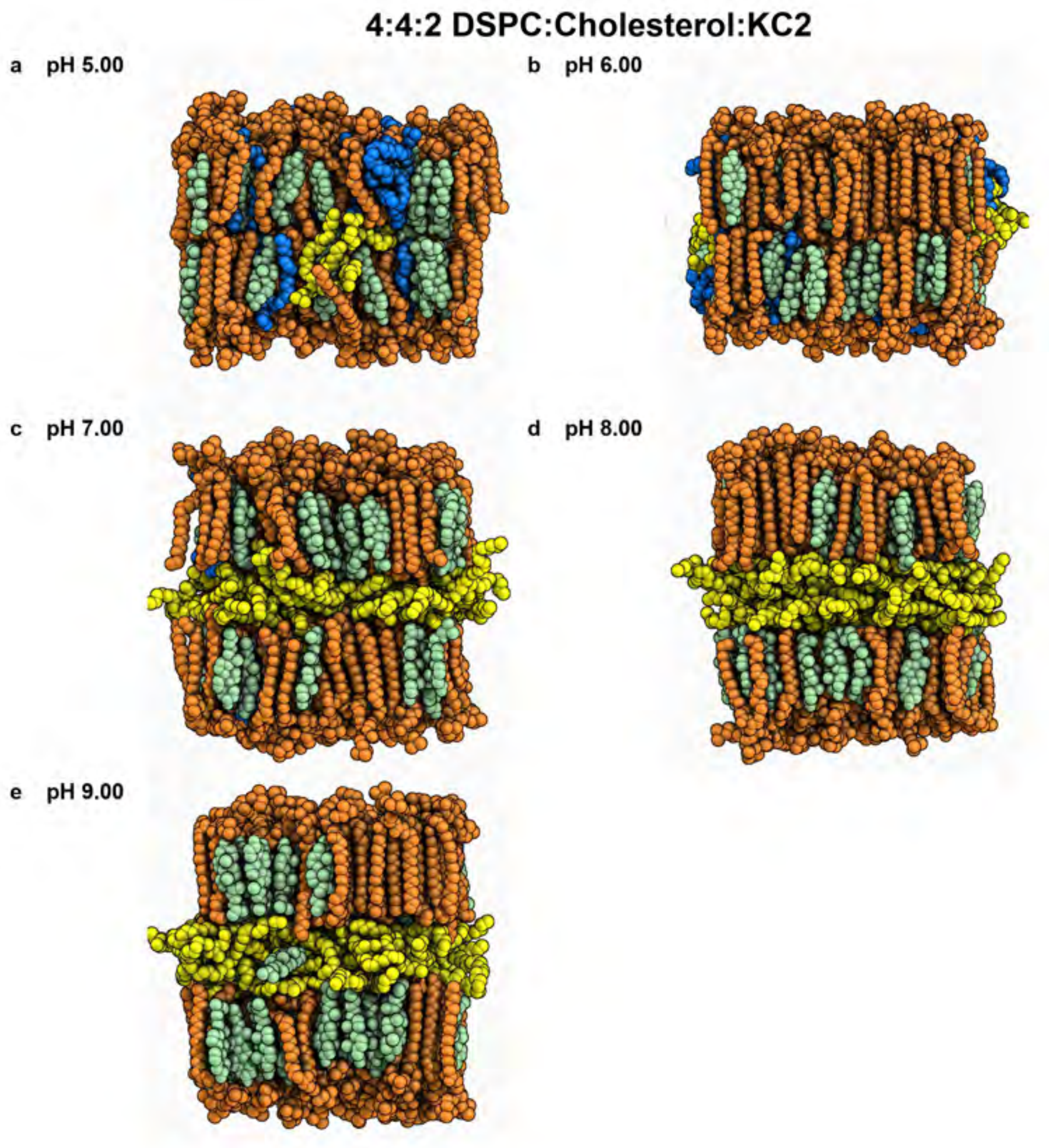

Figure S19: Side-view snapshots of the membrane in the final frame of the simulation at the corresponding pH values. The helper lipid is shown in orange, cholesterol in green, the protonated aminolipid (net charge +1) in blue, the deprotonated aminolipid (net charge 0) in yellow, and the aminolipids without a specific state ($0.2 \leq \lambda \leq 0.8$) in grey.

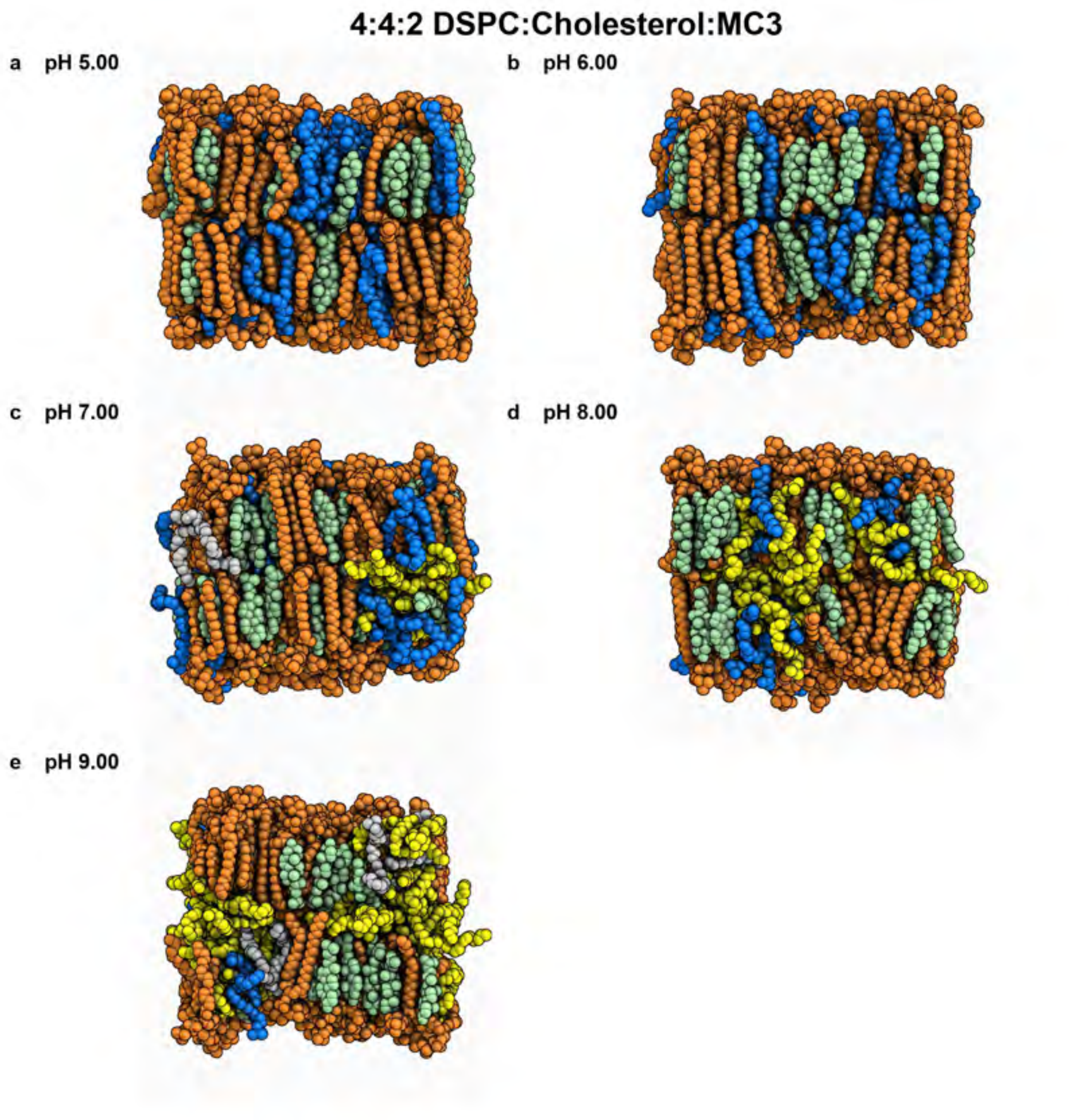

Figure S20: Side-view snapshots of the membrane in the final frame of the simulation at the corresponding pH values. The helper lipid is shown in orange, cholesterol in green, the protonated aminolipid (net charge +1) in blue, the deprotonated aminolipid (net charge 0) in yellow, and the aminolipids without a specific state ($0.2 \leq \lambda \leq 0.8$) in grey.

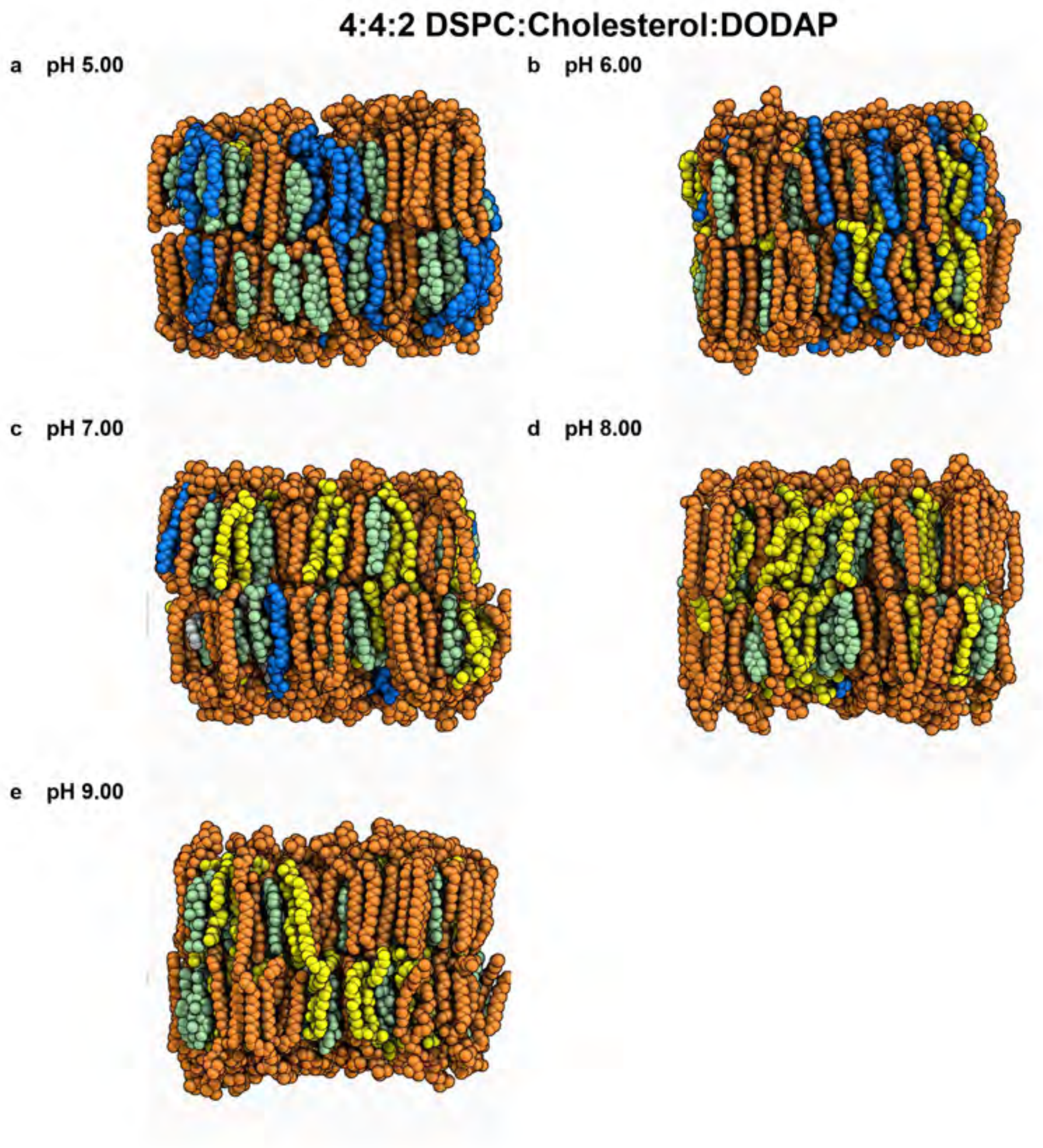

Figure S21: Side-view snapshots of the membrane in the final frame of the simulation at the corresponding pH values. The helper lipid is shown in orange, cholesterol in green, the protonated aminolipid (net charge +1) in blue, the deprotonated aminolipid (net charge 0) in yellow, and the aminolipids without a specific state ($0.2 \leq \lambda \leq 0.8$) in grey.

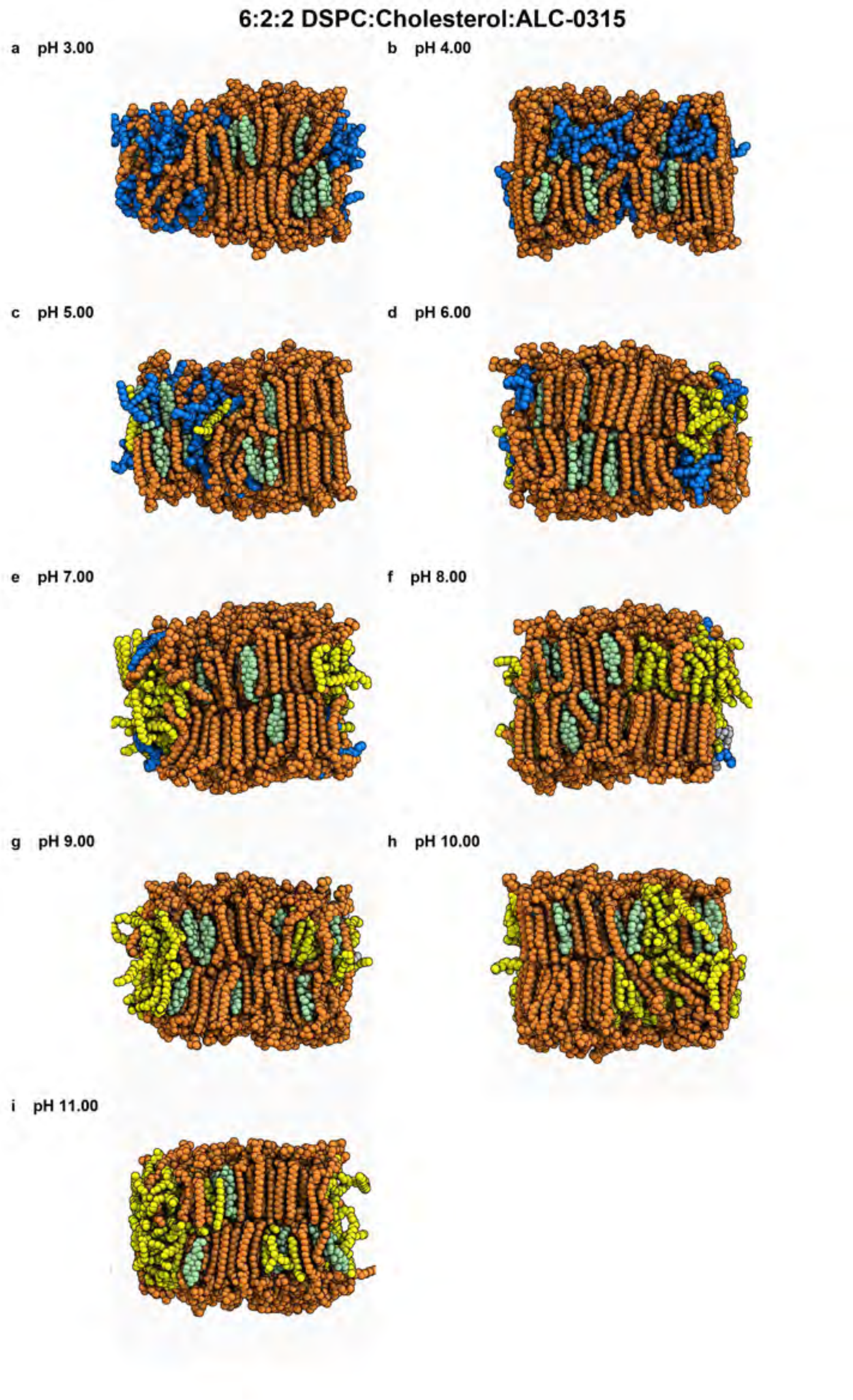

Figure S22: Side-view snapshots of the membrane in the final frame of the simulation at the corresponding pH values. The helper lipid is shown in orange, cholesterol in green, the protonated aminolipid (net charge +1) in blue, the deprotonated aminolipid (net charge 0) in yellow, and the aminolipids without a specific state ($0.2 \leq \lambda \leq 0.8$) in grey.

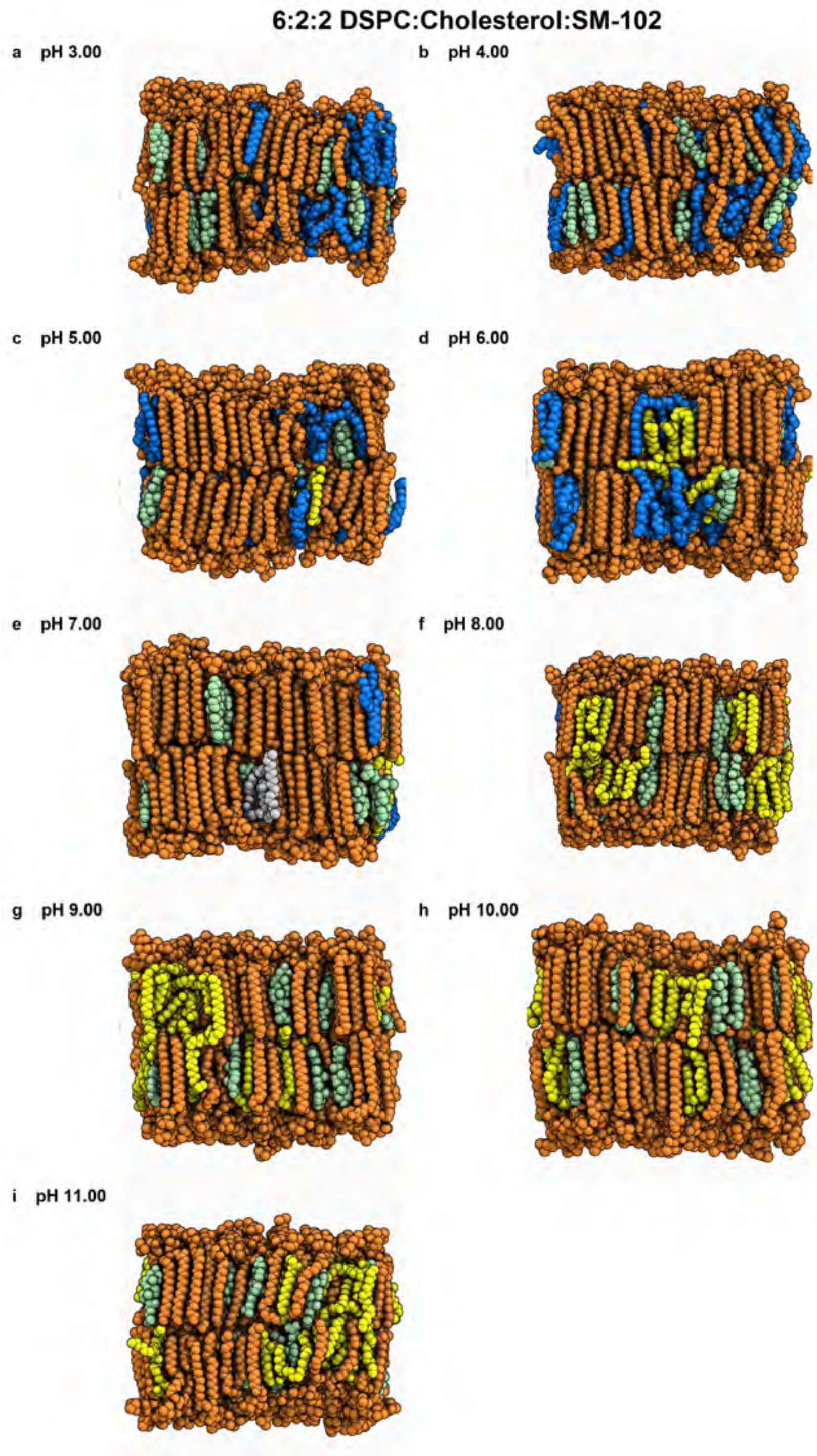

Figure S23: Side-view snapshots of the membrane in the final frame of the simulation at the corresponding pH values. The helper lipid is shown in orange, cholesterol in green, the protonated aminolipid (net charge +1) in blue, the deprotonated aminolipid (net charge 0) in yellow, and the aminolipids without a specific state ($0.2 \leq \lambda \leq 0.8$) in grey.

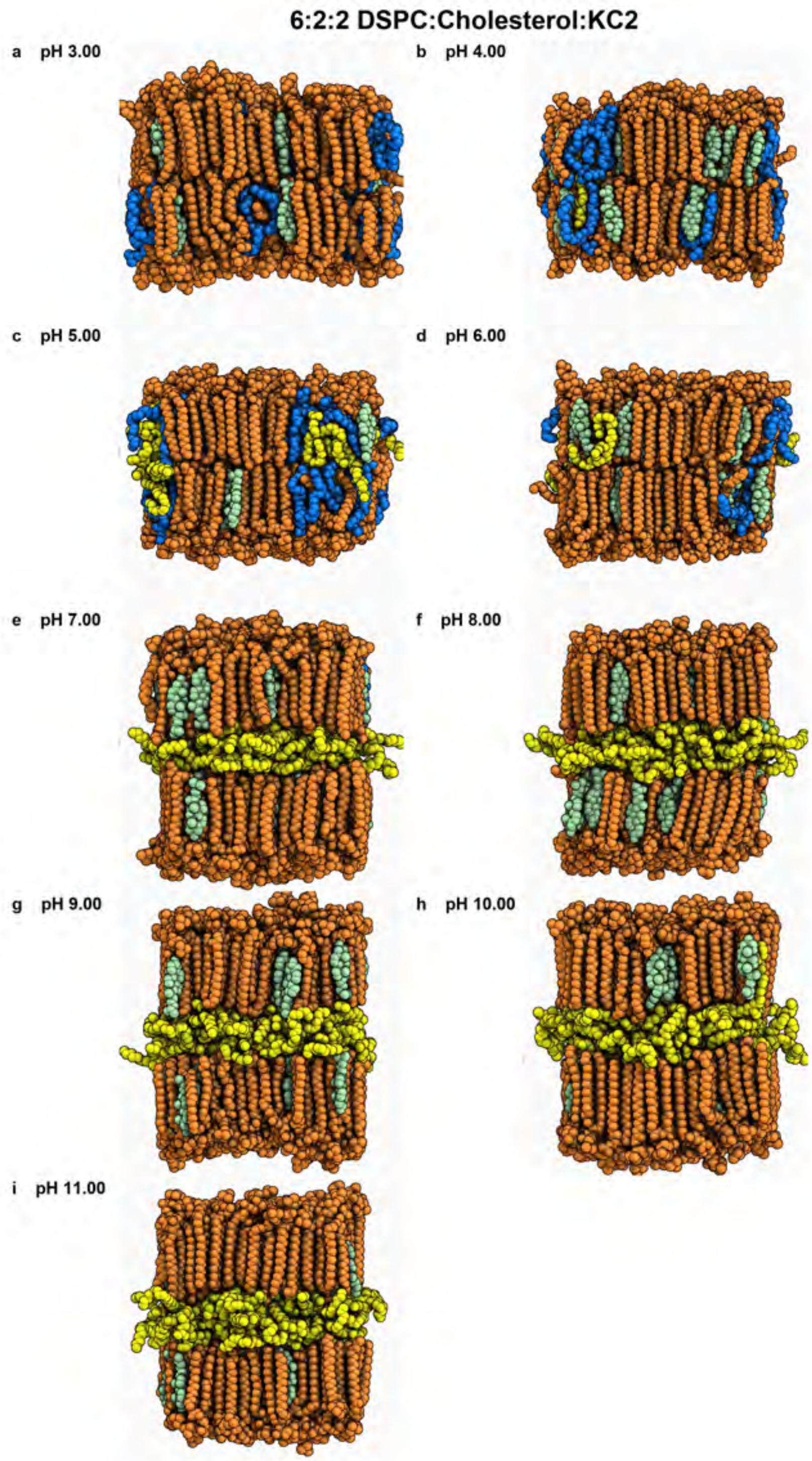

Figure S24: Side-view snapshots of the membrane in the final frame of the simulation at the corresponding pH values. The helper lipid is shown in orange, cholesterol in green, the protonated aminolipid (net charge +1) in blue, the deprotonated aminolipid (net charge 0) in yellow, and the aminolipids without a specific state ($0.2 \leq \lambda \leq 0.8$) in grey.

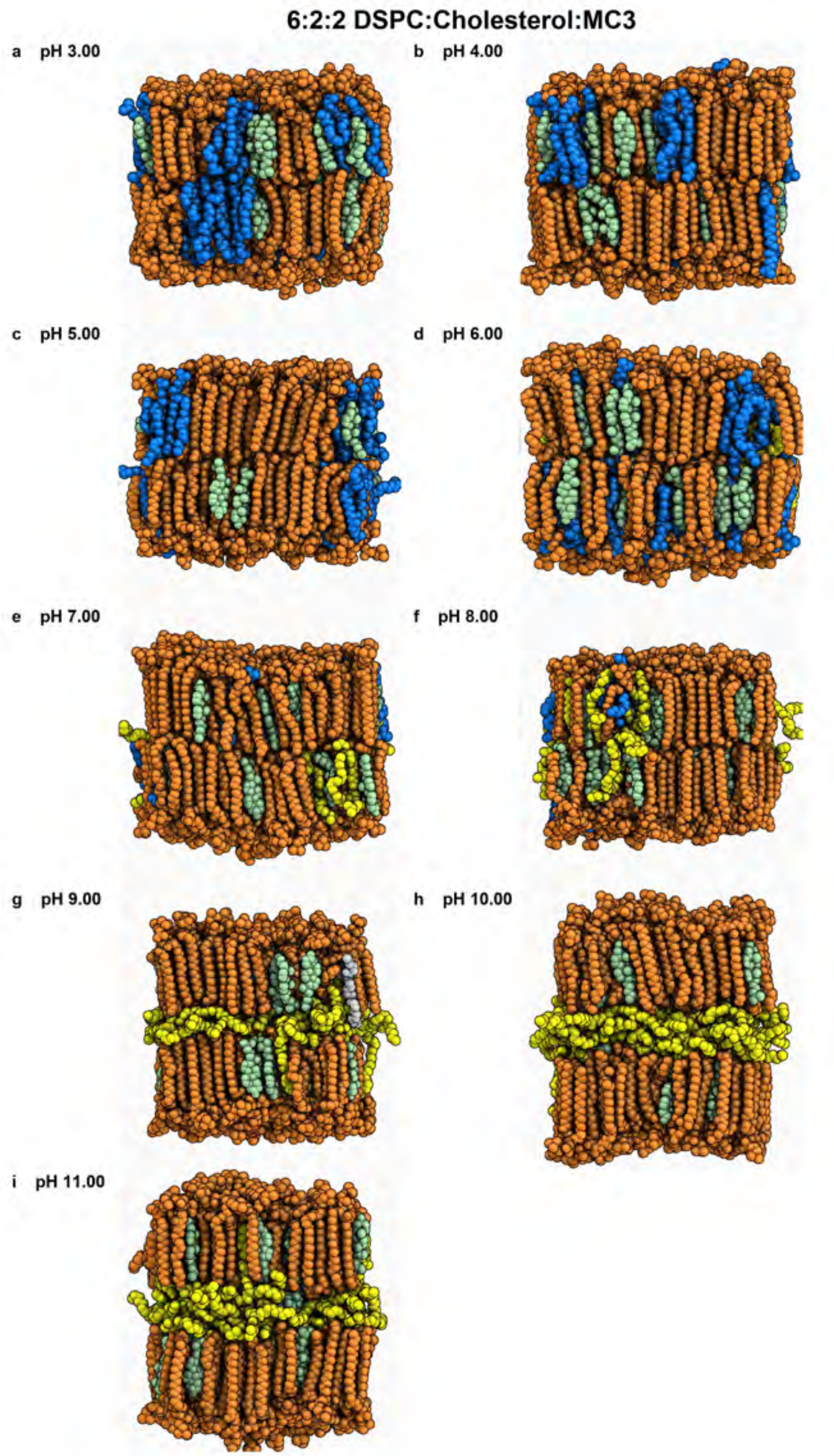

Figure S25: Side-view snapshots of the membrane in the final frame of the simulation at the corresponding pH values. The helper lipid is shown in orange, cholesterol in green, the protonated aminolipid (net charge +1) in blue, the deprotonated aminolipid (net charge 0) in yellow, and the aminolipids without a specific state ($0.2 \leq \lambda \leq 0.8$) in grey.

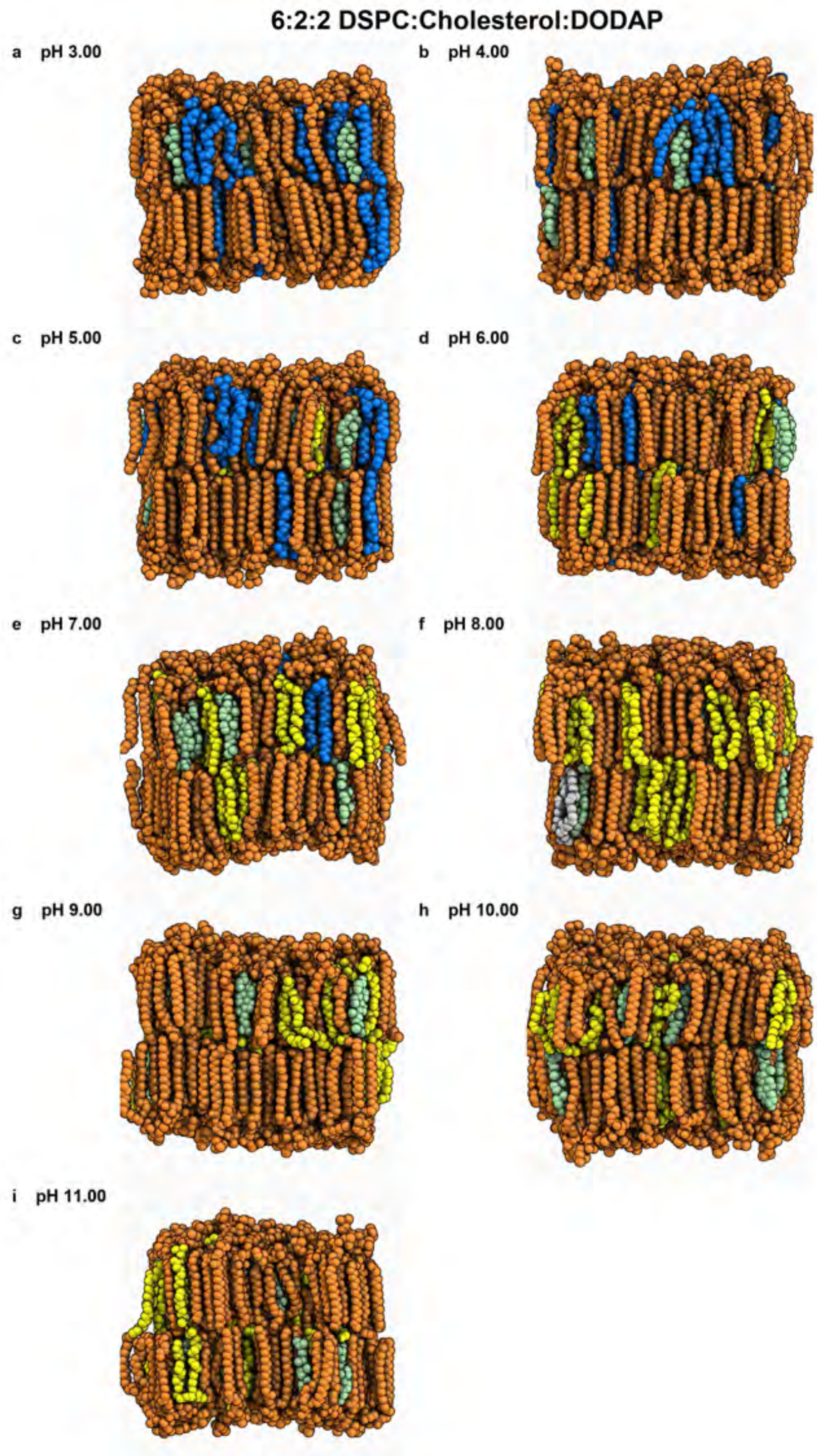

Figure S26: Side-view snapshots of the membrane in the final frame of the simulation at the corresponding pH values. The helper lipid is shown in orange, cholesterol in green, the protonated aminolipid (net charge +1) in blue, the deprotonated aminolipid (net charge 0) in yellow, and the aminolipids without a specific state ($0.2 \leq \lambda \leq 0.8$) in grey.

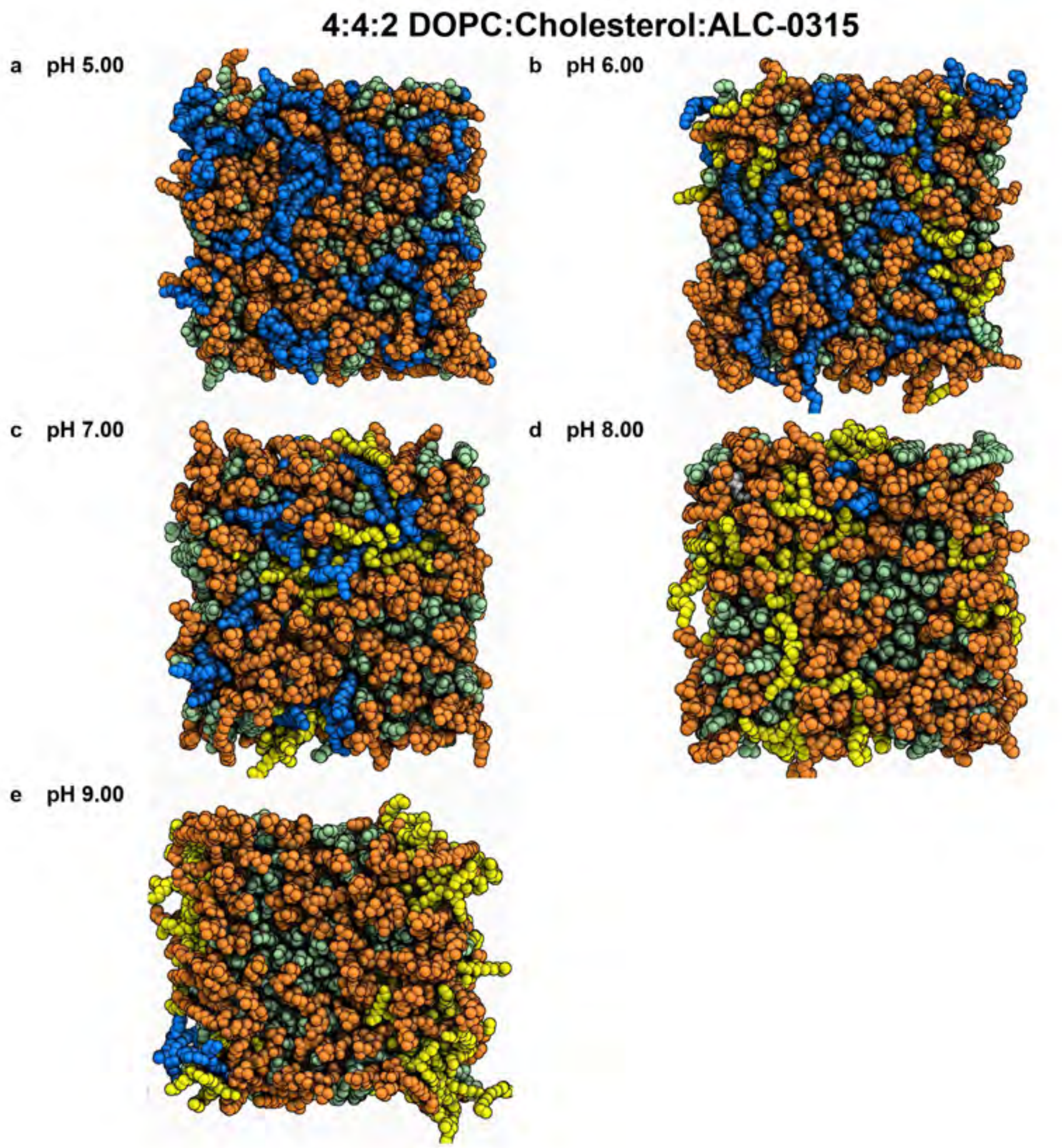

Figure S27: Top-view snapshots of the membrane in the final frame of the simulation at the corresponding pH values. The helper lipid is shown in orange, cholesterol in green, the protonated aminolipid (net charge +1) in blue, the deprotonated aminolipid (net charge 0) in yellow, and the aminolipids without a specific state ($0.2 \leq \lambda \leq 0.8$) in grey.

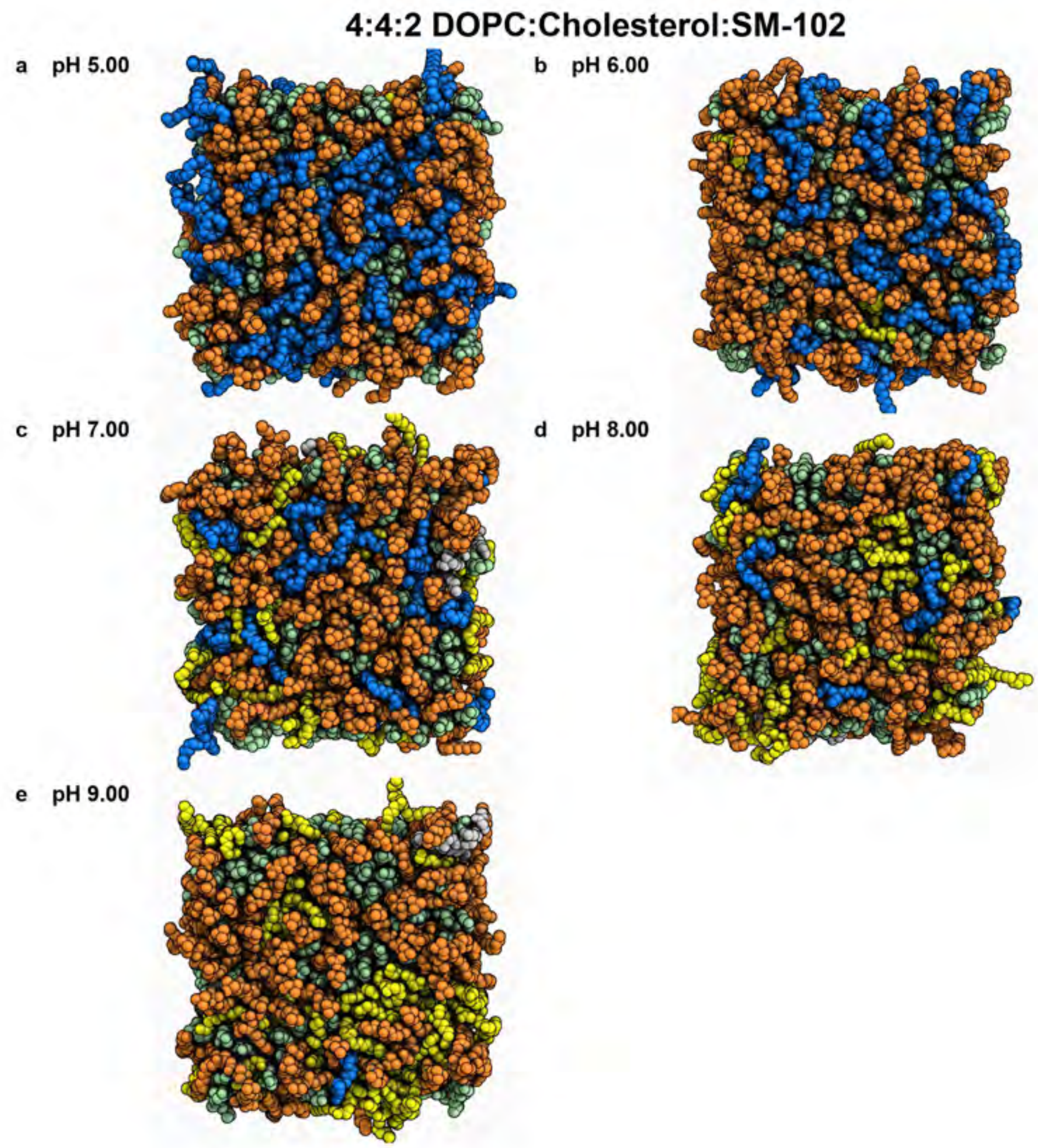

Figure S28: Top-view snapshots of the membrane in the final frame of the simulation at the corresponding pH values. The helper lipid is shown in orange, cholesterol in green, the protonated aminolipid (net charge +1) in blue, the deprotonated aminolipid (net charge 0) in yellow, and the aminolipids without a specific state ($0.2 \leq \lambda \leq 0.8$) in grey.

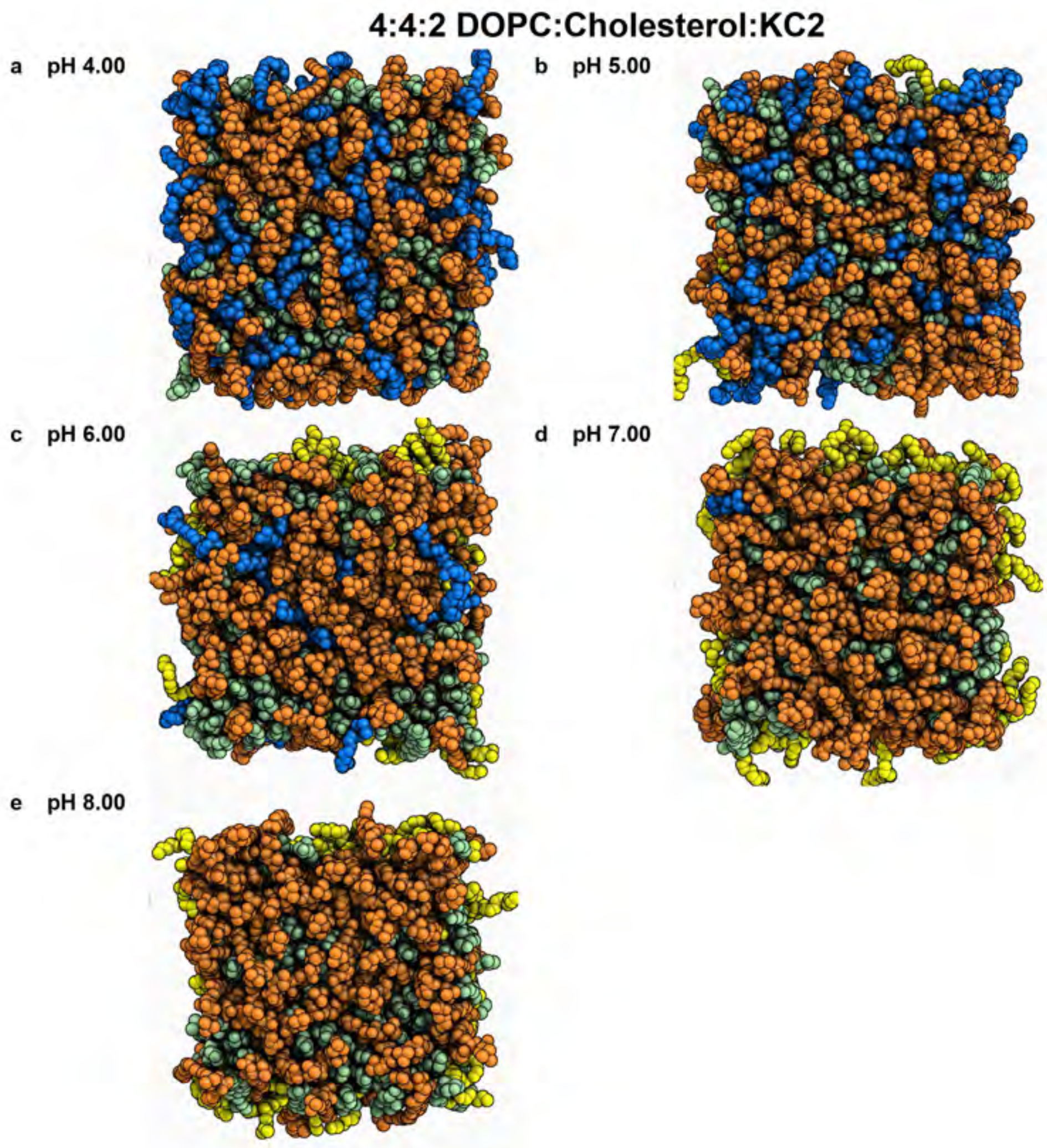

Figure S29: Top-view snapshots of the membrane in the final frame of the simulation at the corresponding pH values. The helper lipid is shown in orange, cholesterol in green, the protonated aminolipid (net charge +1) in blue, the deprotonated aminolipid (net charge 0) in yellow, and the aminolipids without a specific state ($0.2 \leq \lambda \leq 0.8$) in grey.

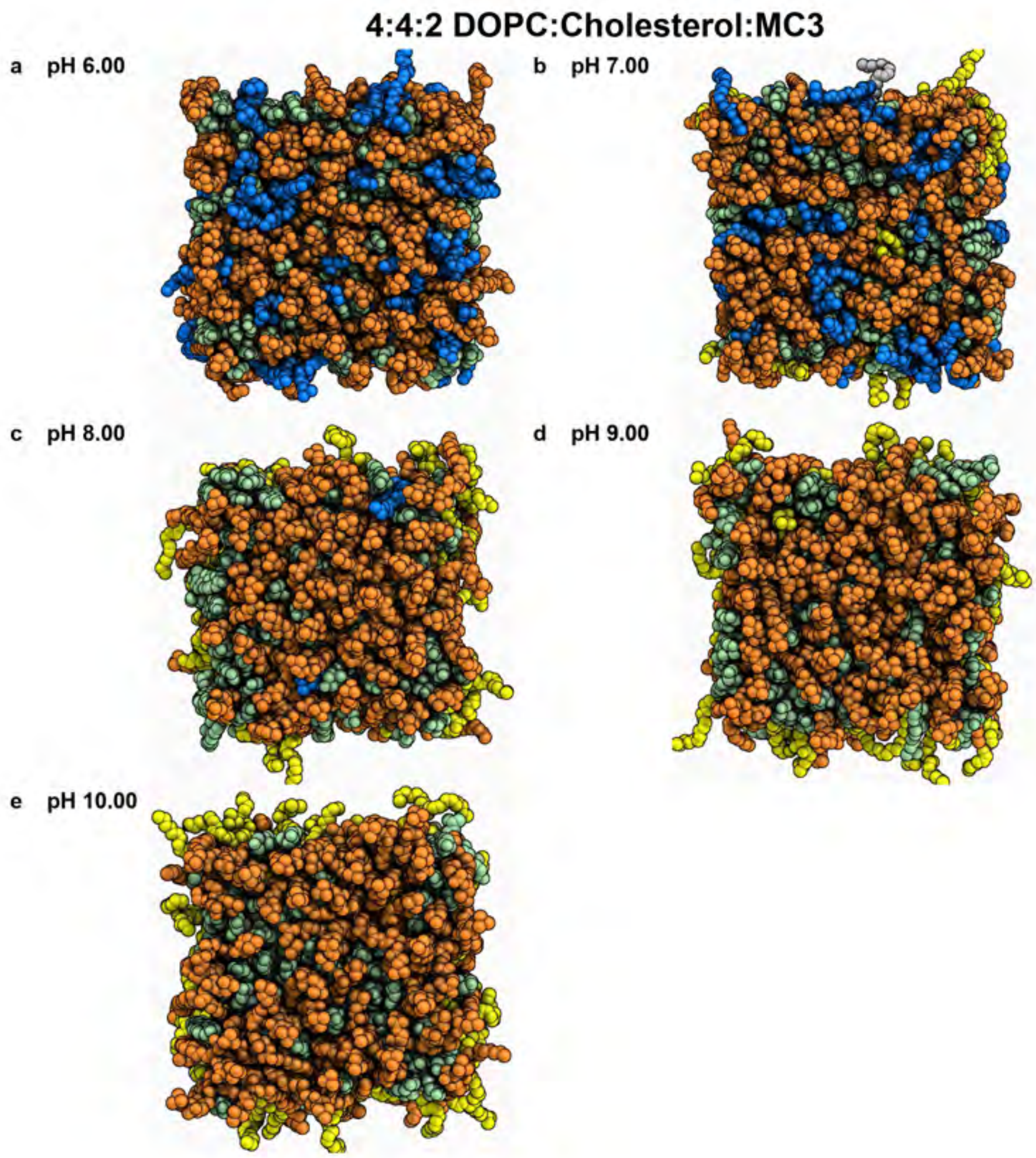

Figure S30: Top-view snapshots of the membrane in the final frame of the simulation at the corresponding pH values. The helper lipid is shown in orange, cholesterol in green, the protonated aminolipid (net charge +1) in blue, the deprotonated aminolipid (net charge 0) in yellow, and the aminolipids without a specific state ($0.2 \leq \lambda \leq 0.8$) in grey.

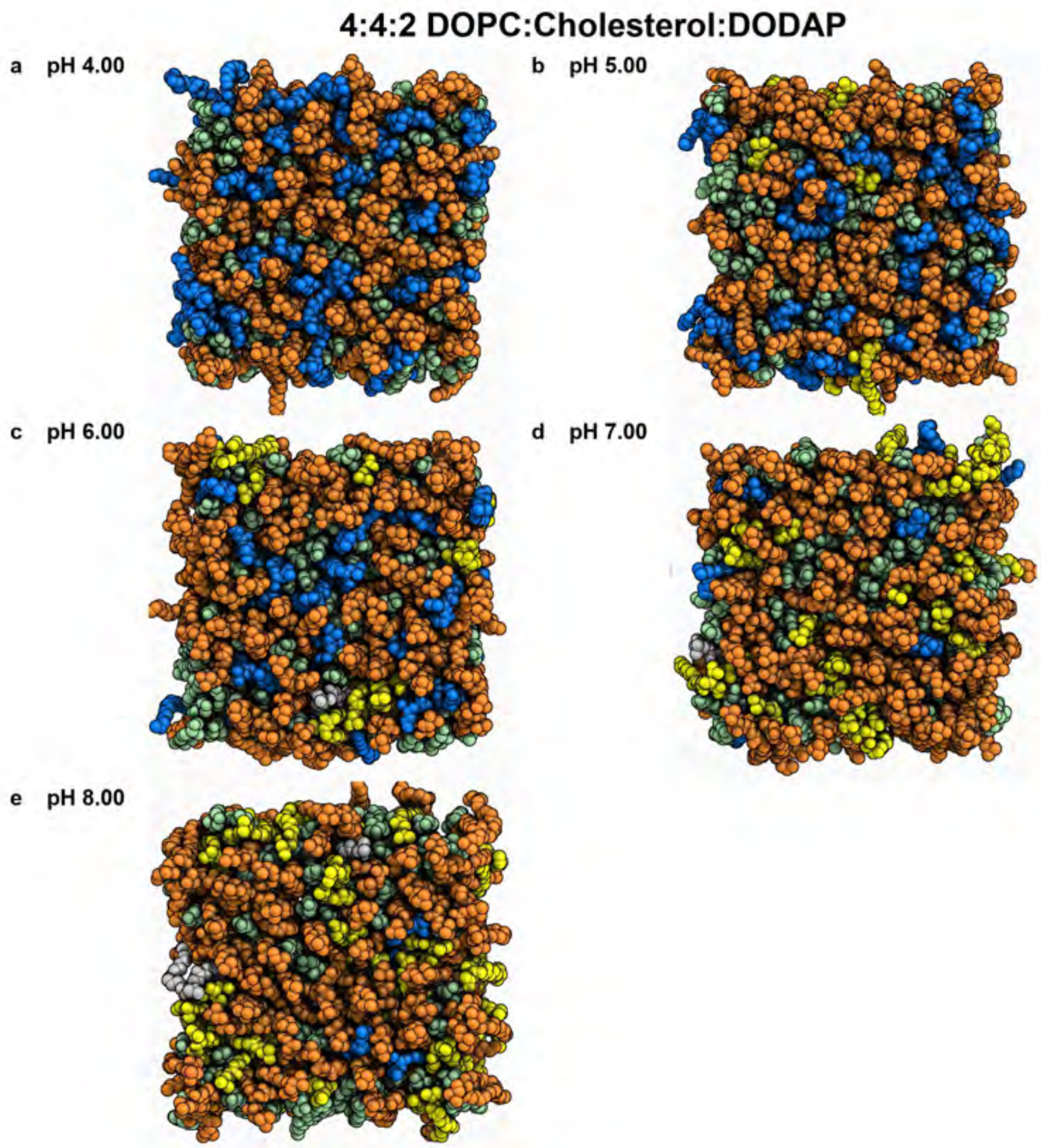

Figure S31: Top-view snapshots of the membrane in the final frame of the simulation at the corresponding pH values. The helper lipid is shown in orange, cholesterol in green, the protonated aminolipid (net charge +1) in blue, the deprotonated aminolipid (net charge 0) in yellow, and the aminolipids without a specific state ($0.2 \leq \lambda \leq 0.8$) in grey.

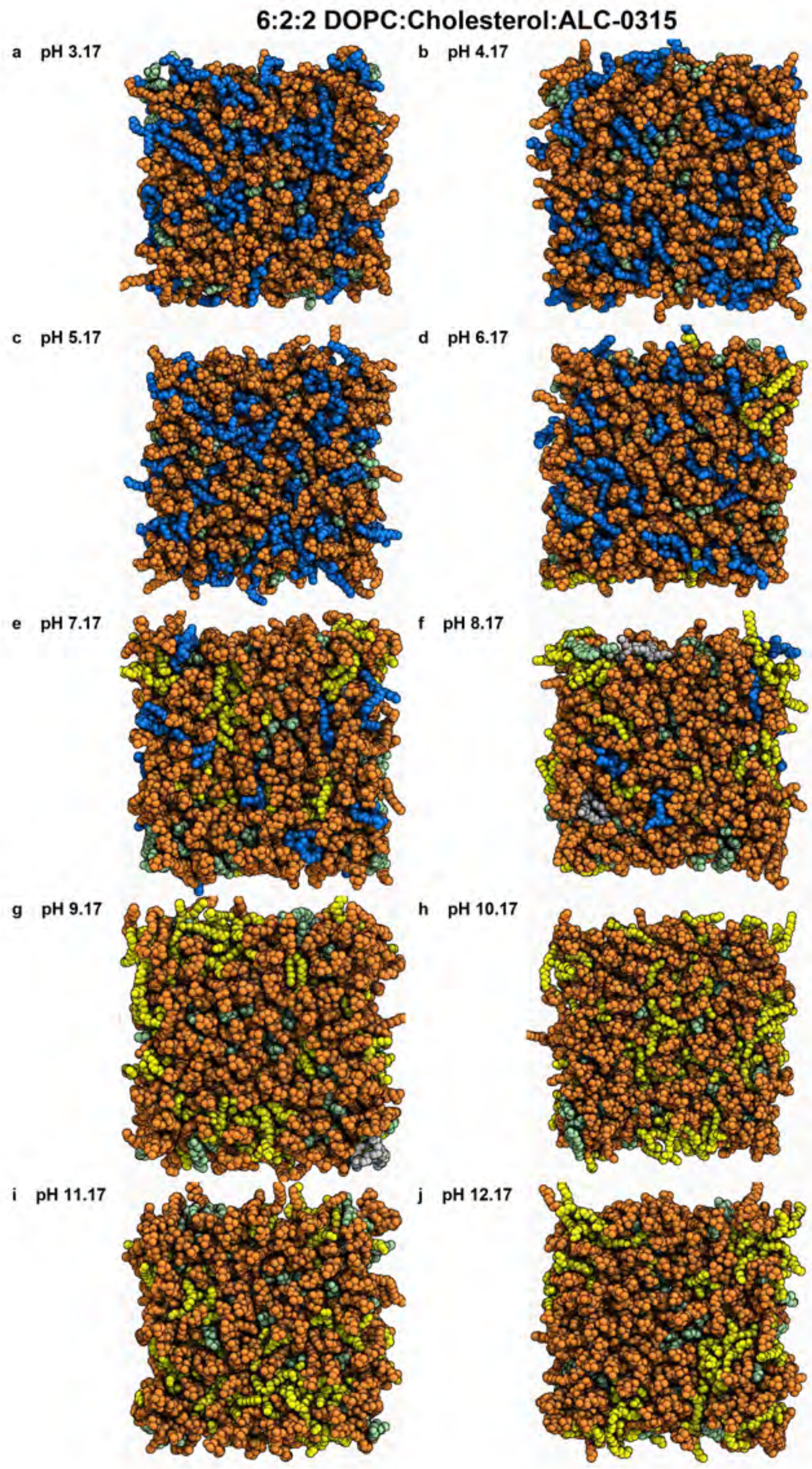

Figure S32: Top-view snapshots of the membrane in the final frame of the simulation at the corresponding pH values. The helper lipid is shown in orange, cholesterol in green, the protonated aminolipid (net charge +1) in blue, the deprotonated aminolipid (net charge 0) in yellow, and the aminolipids without a specific state ($0.2 \leq \lambda \leq 0.8$) in grey.

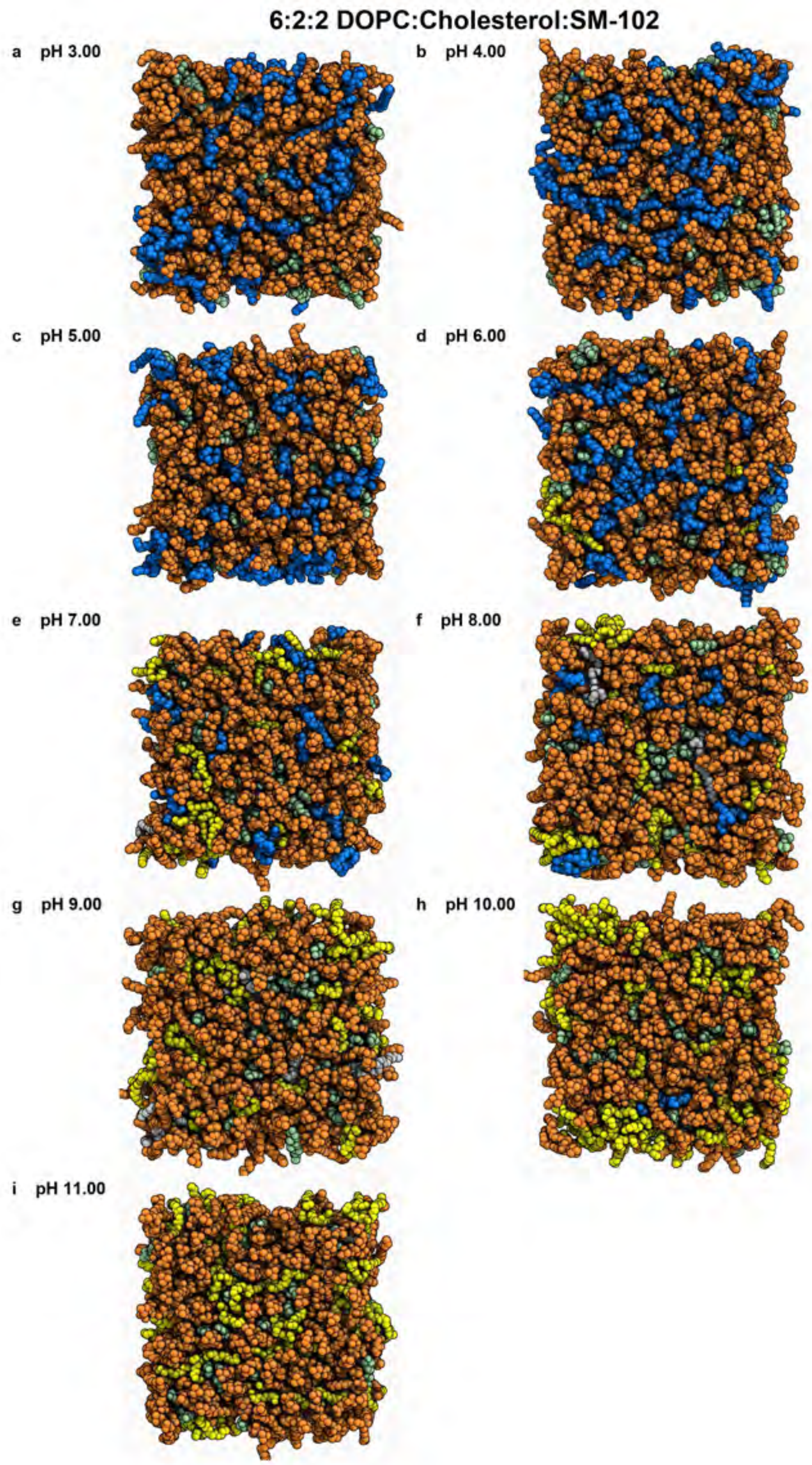

Figure S33: Top-view snapshots of the membrane in the final frame of the simulation at the corresponding pH values. The helper lipid is shown in orange, cholesterol in green, the protonated aminolipid (net charge +1) in blue, the deprotonated aminolipid (net charge 0) in yellow, and the aminolipids without a specific state ($0.2 \leq \lambda \leq 0.8$) in grey.

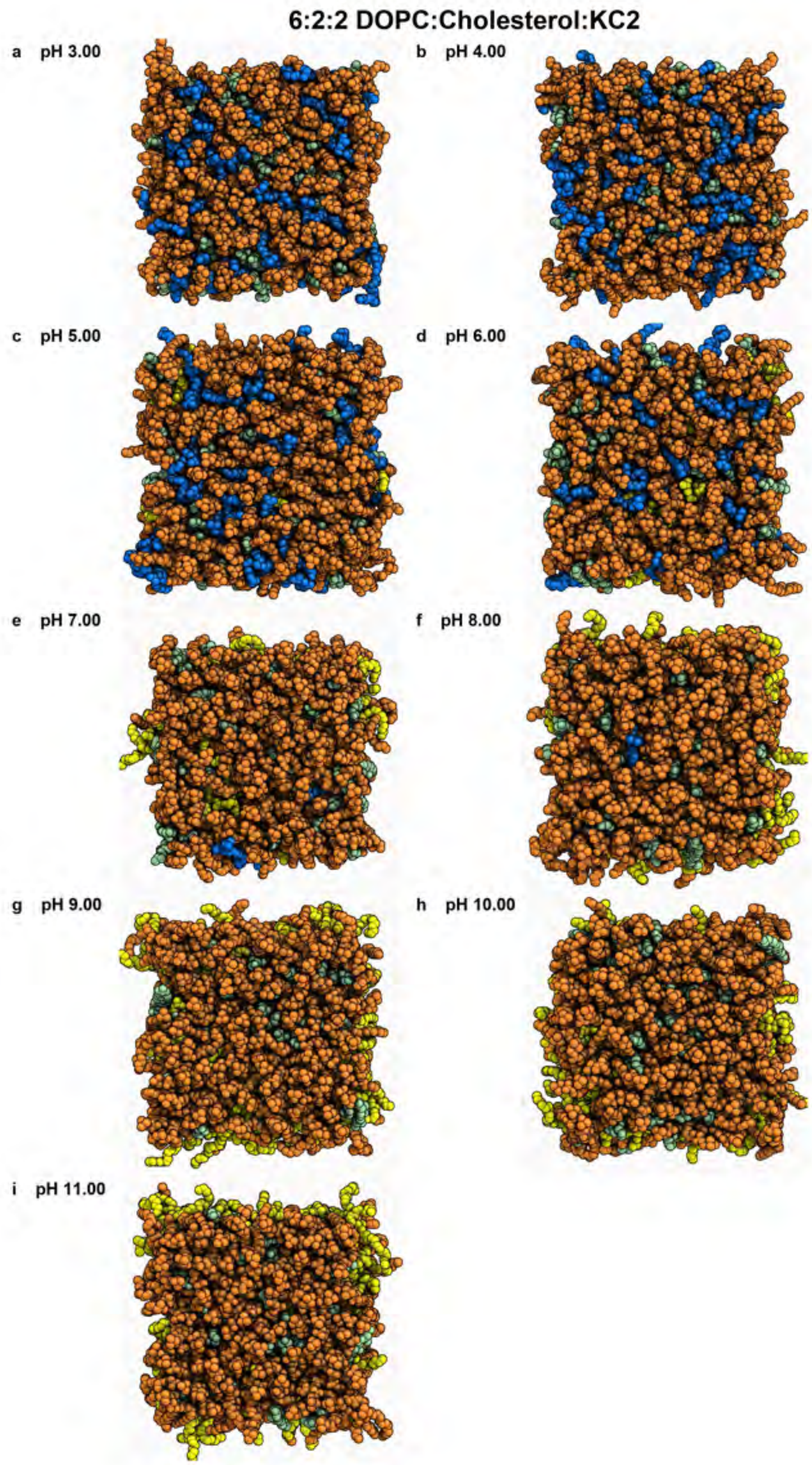

Figure S34: Top-view snapshots of the membrane in the final frame of the simulation at the corresponding pH values. The helper lipid is shown in orange, cholesterol in green, the protonated aminolipid (net charge +1) in blue, the deprotonated aminolipid (net charge 0) in yellow, and the aminolipids without a specific state ($0.2 \leq \lambda \leq 0.8$) in grey.

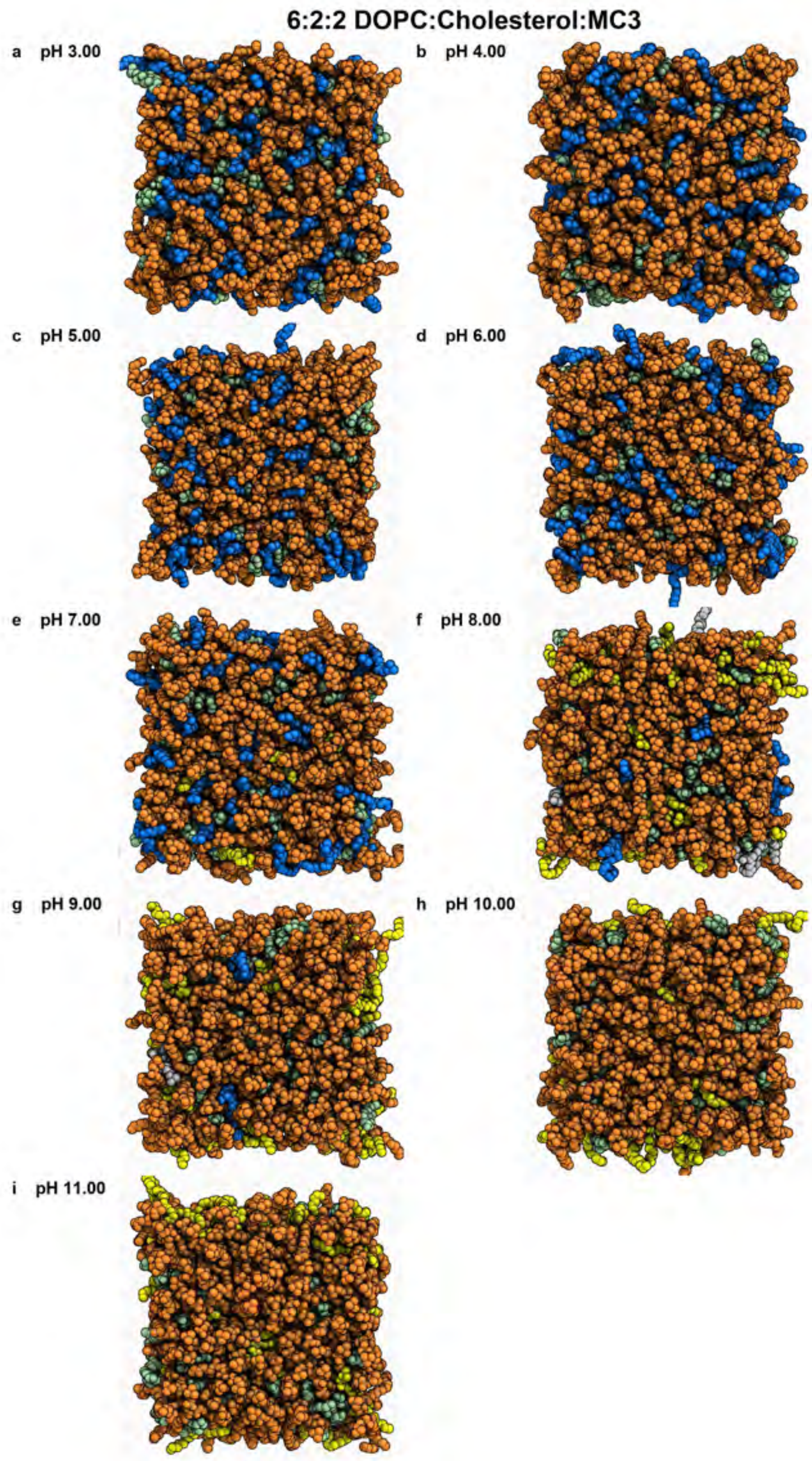

Figure S35: Top-view snapshots of the membrane in the final frame of the simulation at the corresponding pH values. The helper lipid is shown in orange, cholesterol in green, the protonated aminolipid (net charge +1) in blue, the deprotonated aminolipid (net charge 0) in yellow, and the aminolipids without a specific state ($0.2 \leq \lambda \leq 0.8$) in grey.

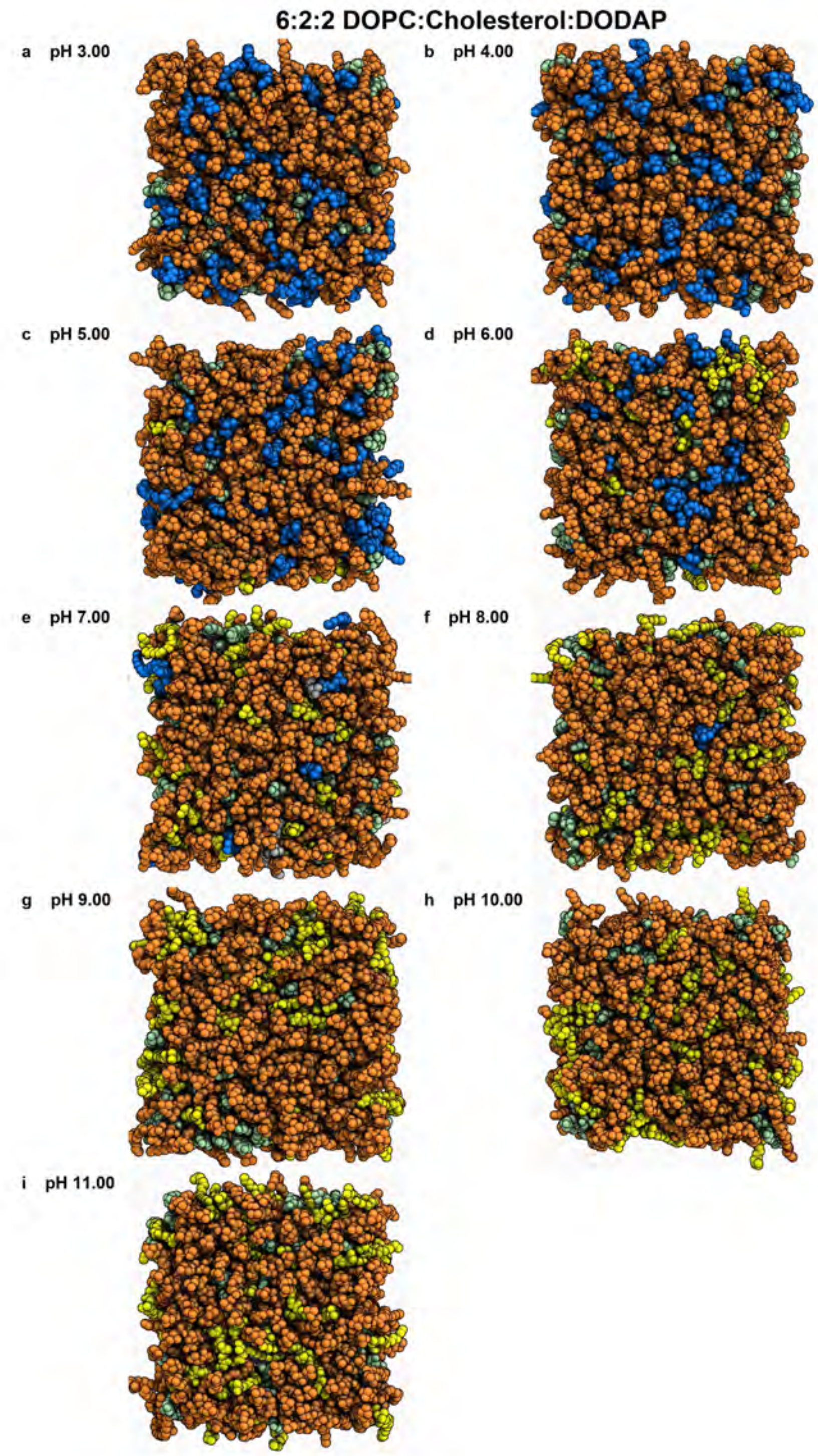

Figure S36: Top-view snapshots of the membrane in the final frame of the simulation at the corresponding pH values. The helper lipid is shown in orange, cholesterol in green, the protonated aminolipid (net charge +1) in blue, the deprotonated aminolipid (net charge 0) in yellow, and the aminolipids without a specific state ($0.2 \leq \lambda \leq 0.8$) in grey.

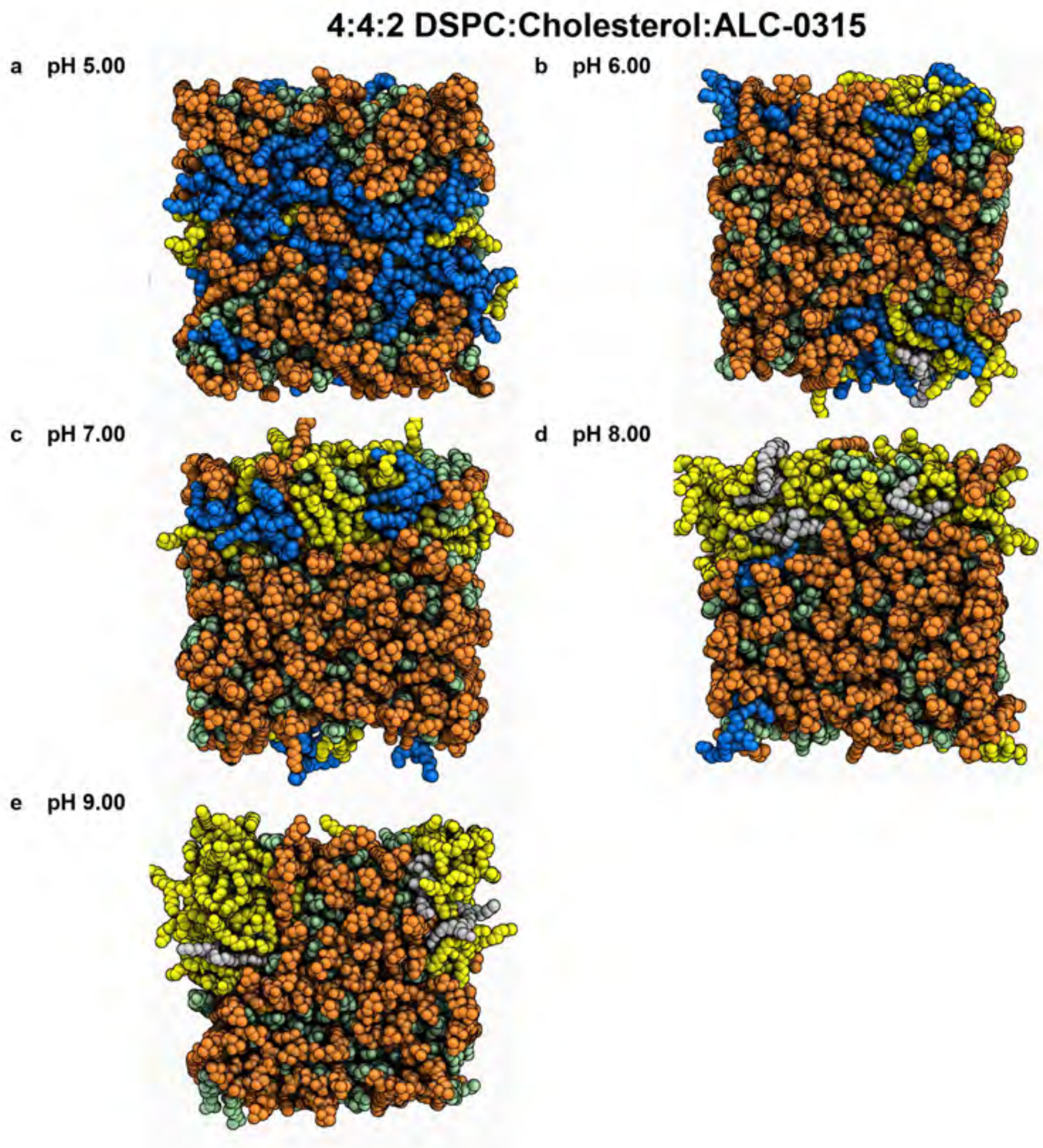

Figure S37: Top-view snapshots of the membrane in the final frame of the simulation at the corresponding pH values. The helper lipid is shown in orange, cholesterol in green, the protonated aminolipid (net charge +1) in blue, the deprotonated aminolipid (net charge 0) in yellow, and the aminolipids without a specific state ($0.2 \leq \lambda \leq 0.8$) in grey.

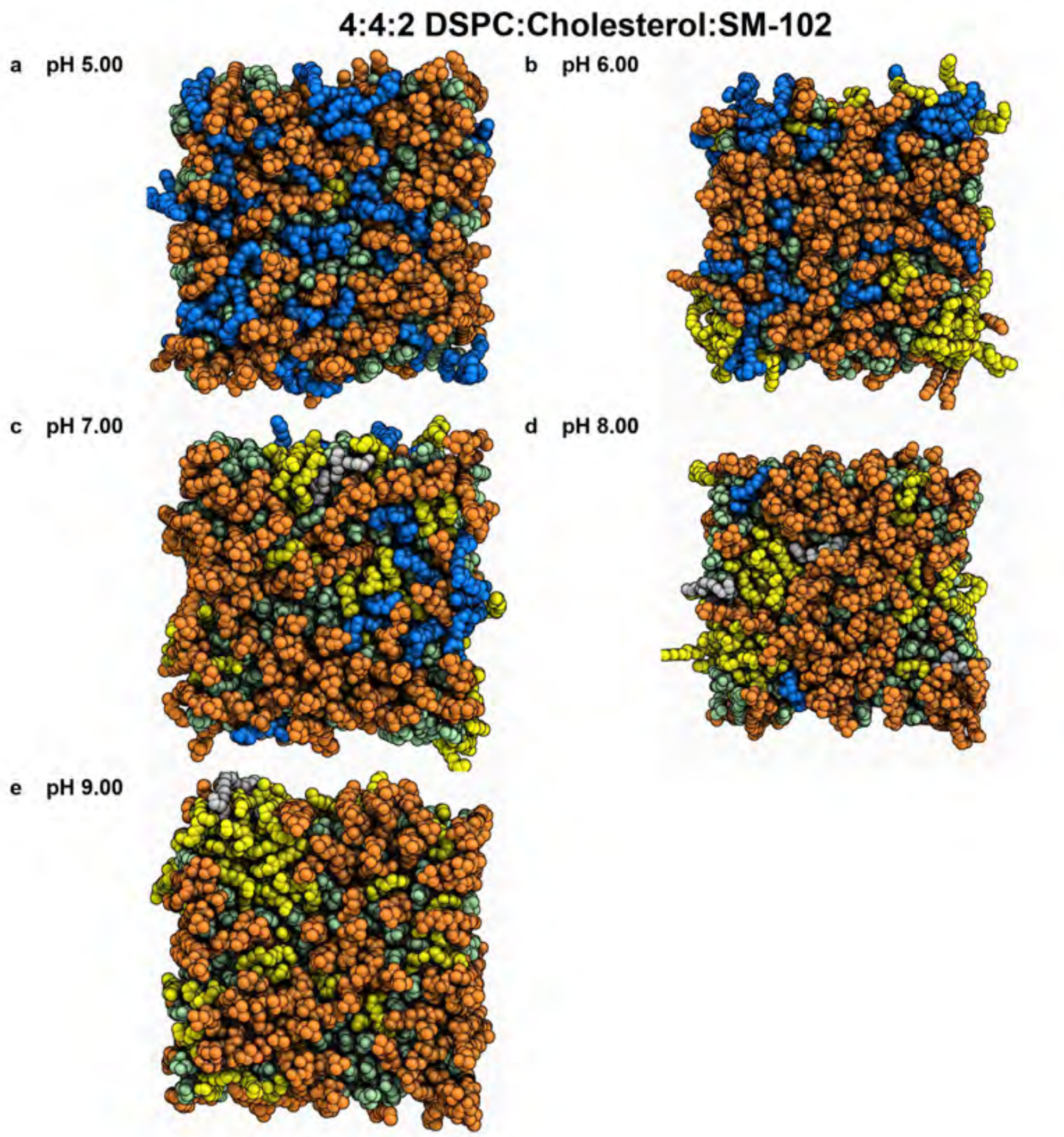

Figure S38: Top-view snapshots of the membrane in the final frame of the simulation at the corresponding pH values. The helper lipid is shown in orange, cholesterol in green, the protonated aminolipid (net charge +1) in blue, the deprotonated aminolipid (net charge 0) in yellow, and the aminolipids without a specific state ($0.2 \leq \lambda \leq 0.8$) in grey.

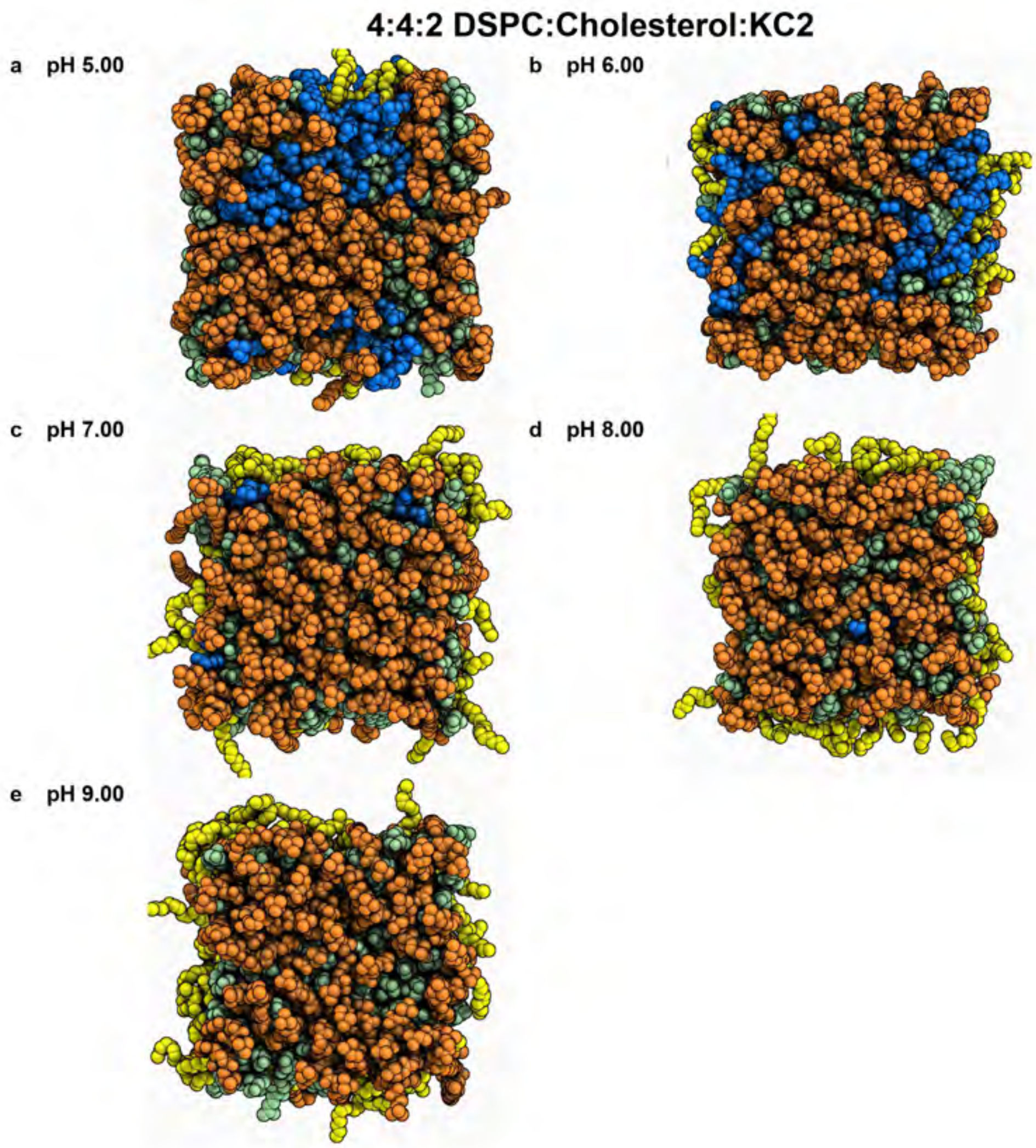

Figure S39: Top-view snapshots of the membrane in the final frame of the simulation at the corresponding pH values. The helper lipid is shown in orange, cholesterol in green, the protonated aminolipid (net charge +1) in blue, the deprotonated aminolipid (net charge 0) in yellow, and the aminolipids without a specific state ($0.2 \leq \lambda \leq 0.8$) in grey.

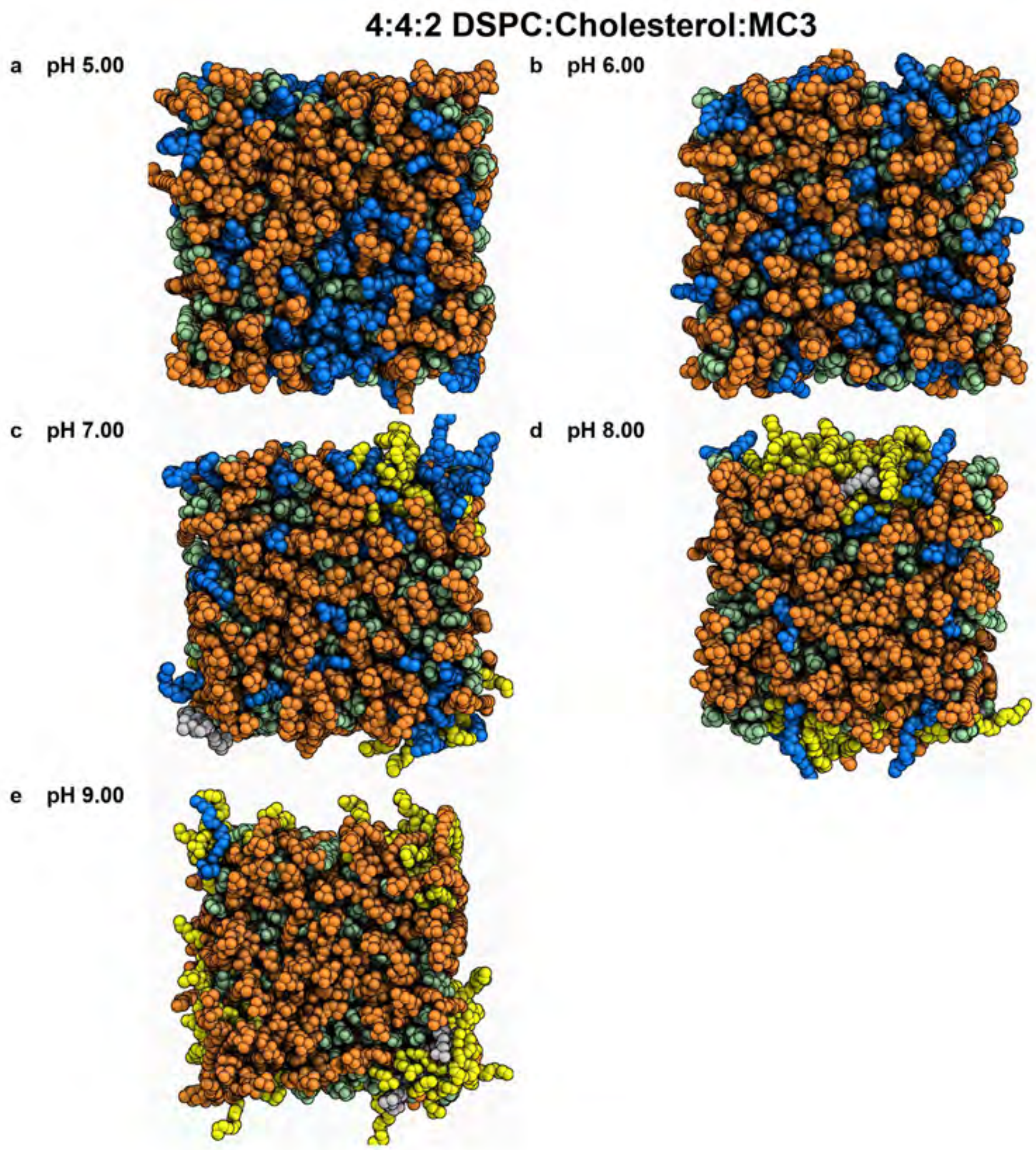

Figure S40: Top-view snapshots of the membrane in the final frame of the simulation at the corresponding pH values. The helper lipid is shown in orange, cholesterol in green, the protonated aminolipid (net charge +1) in blue, the deprotonated aminolipid (net charge 0) in yellow, and the aminolipids without a specific state ($0.2 \leq \lambda \leq 0.8$) in grey.

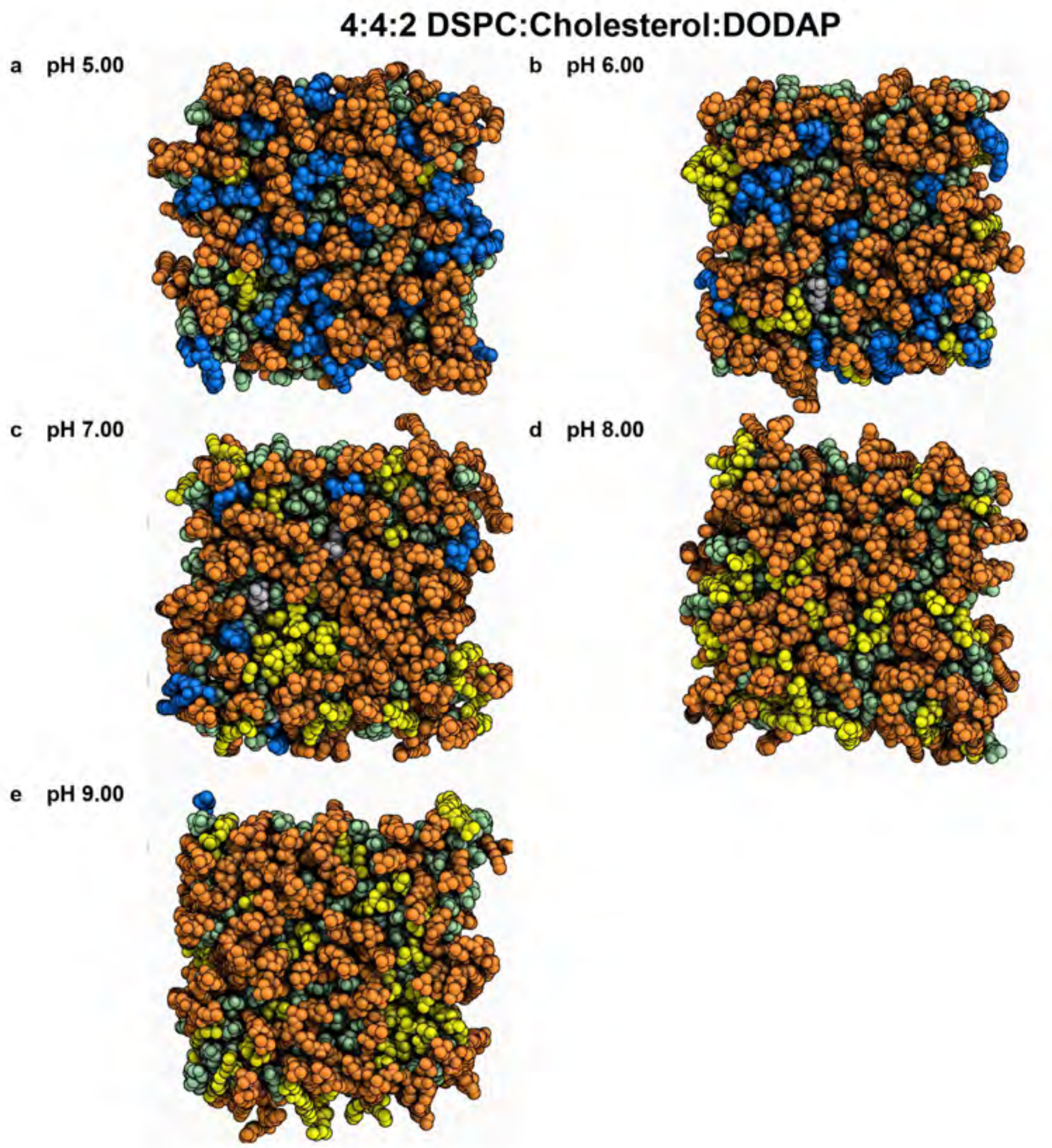

Figure S41: Top-view snapshots of the membrane in the final frame of the simulation at the corresponding pH values. The helper lipid is shown in orange, cholesterol in green, the protonated aminolipid (net charge +1) in blue, the deprotonated aminolipid (net charge 0) in yellow, and the aminolipids without a specific state ($0.2 \leq \lambda \leq 0.8$) in grey.

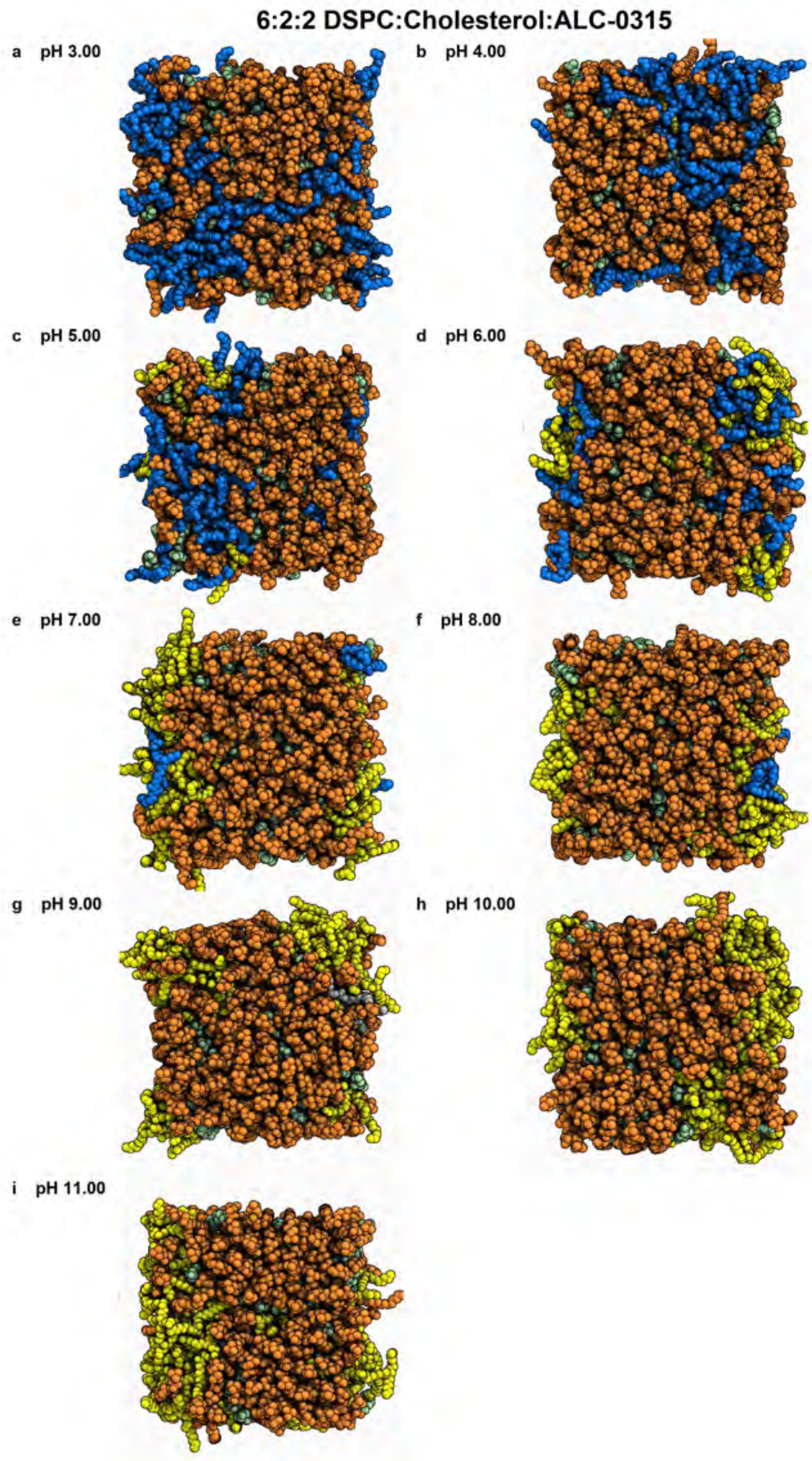

Figure S42: Top-view snapshots of the membrane in the final frame of the simulation at the corresponding pH values. The helper lipid is shown in orange, cholesterol in green, the protonated aminolipid (net charge +1) in blue, the deprotonated aminolipid (net charge 0) in yellow, and the aminolipids without a specific state ($0.2 \leq \lambda \leq 0.8$) in grey.

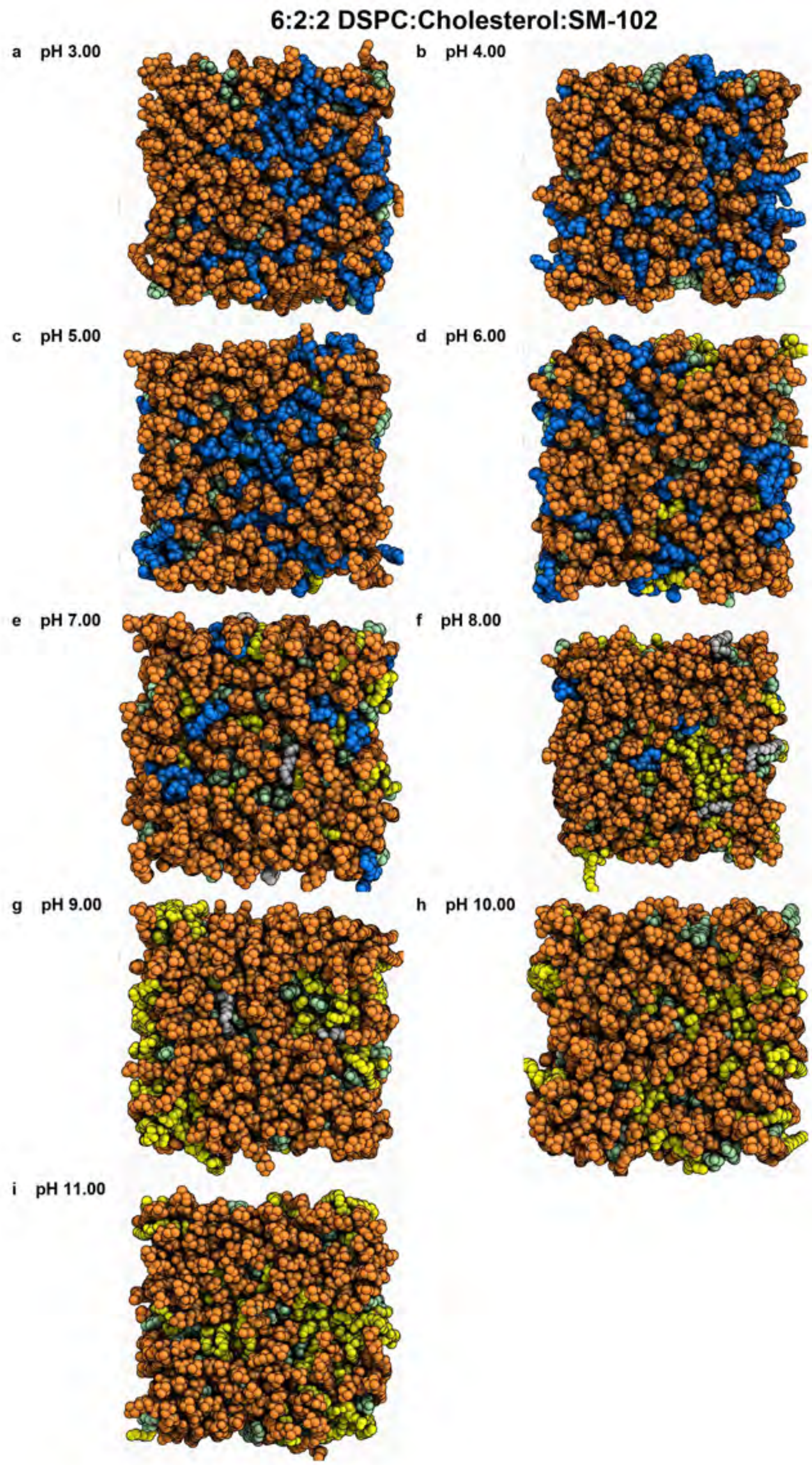

Figure S43: Top-view snapshots of the membrane in the final frame of the simulation at the corresponding pH values. The helper lipid is shown in orange, cholesterol in green, the protonated aminolipid (net charge +1) in blue, the deprotonated aminolipid (net charge 0) in yellow, and the aminolipids without a specific state ($0.2 \leq \lambda \leq 0.8$) in grey.

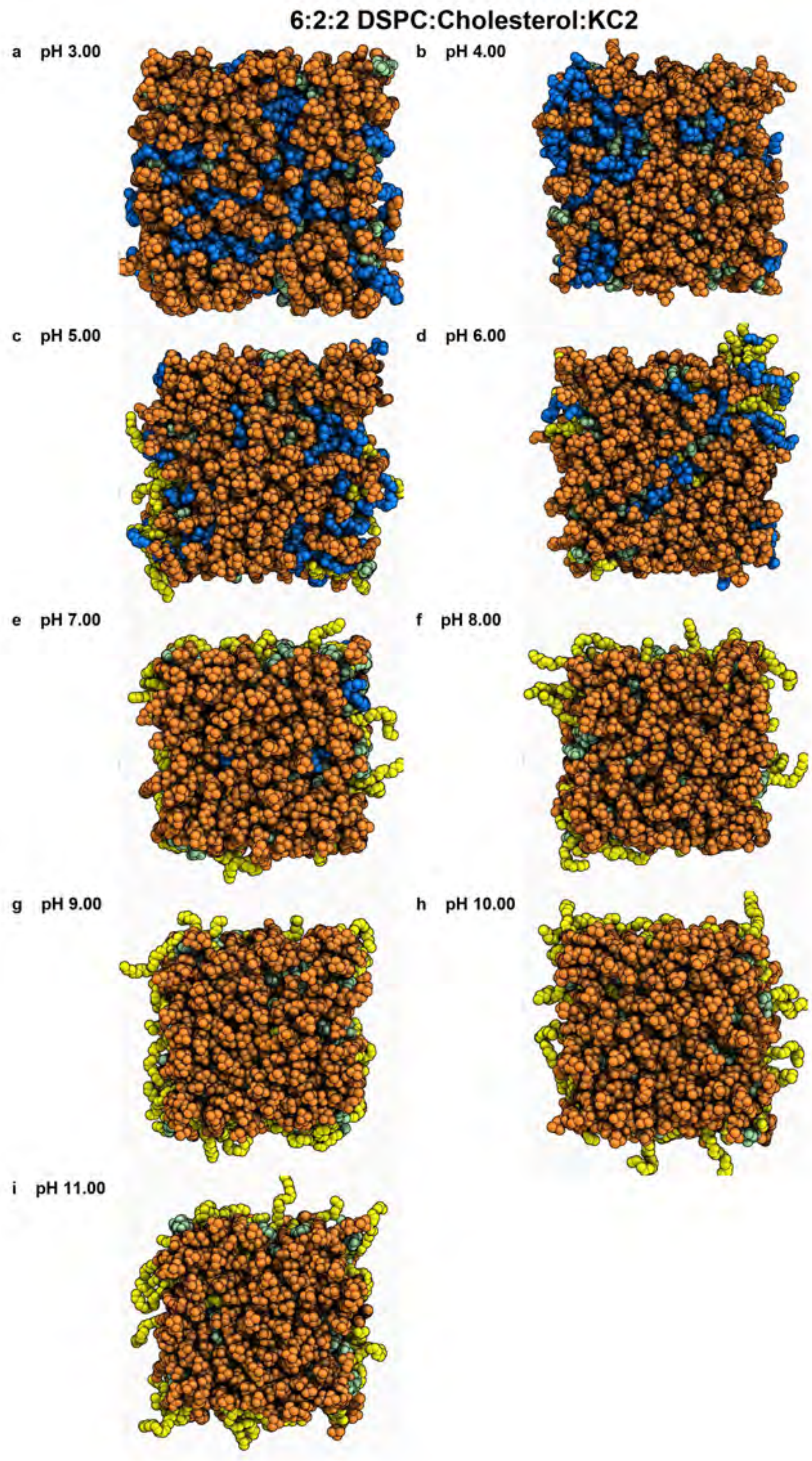

Figure S44: Top-view snapshots of the membrane in the final frame of the simulation at the corresponding pH values. The helper lipid is shown in orange, cholesterol in green, the protonated aminolipid (net charge +1) in blue, the deprotonated aminolipid (net charge 0) in yellow, and the aminolipids without a specific state ($0.2 \leq \lambda \leq 0.8$) in grey.

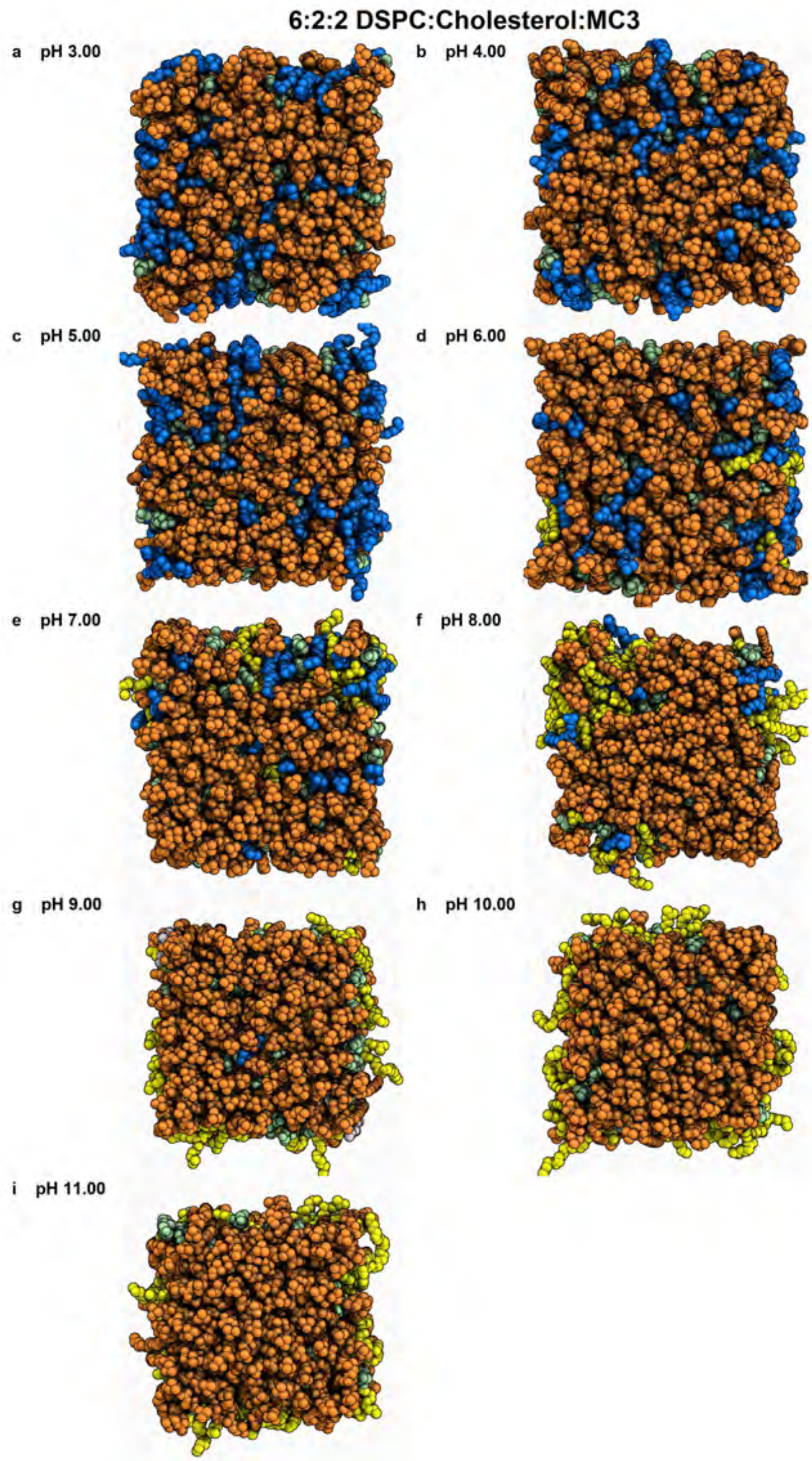

Figure S45: Top-view snapshots of the membrane in the final frame of the simulation at the corresponding pH values. The helper lipid is shown in orange, cholesterol in green, the protonated aminolipid (net charge +1) in blue, the deprotonated aminolipid (net charge 0) in yellow, and the aminolipids without a specific state ($0.2 \leq \lambda \leq 0.8$) in grey.

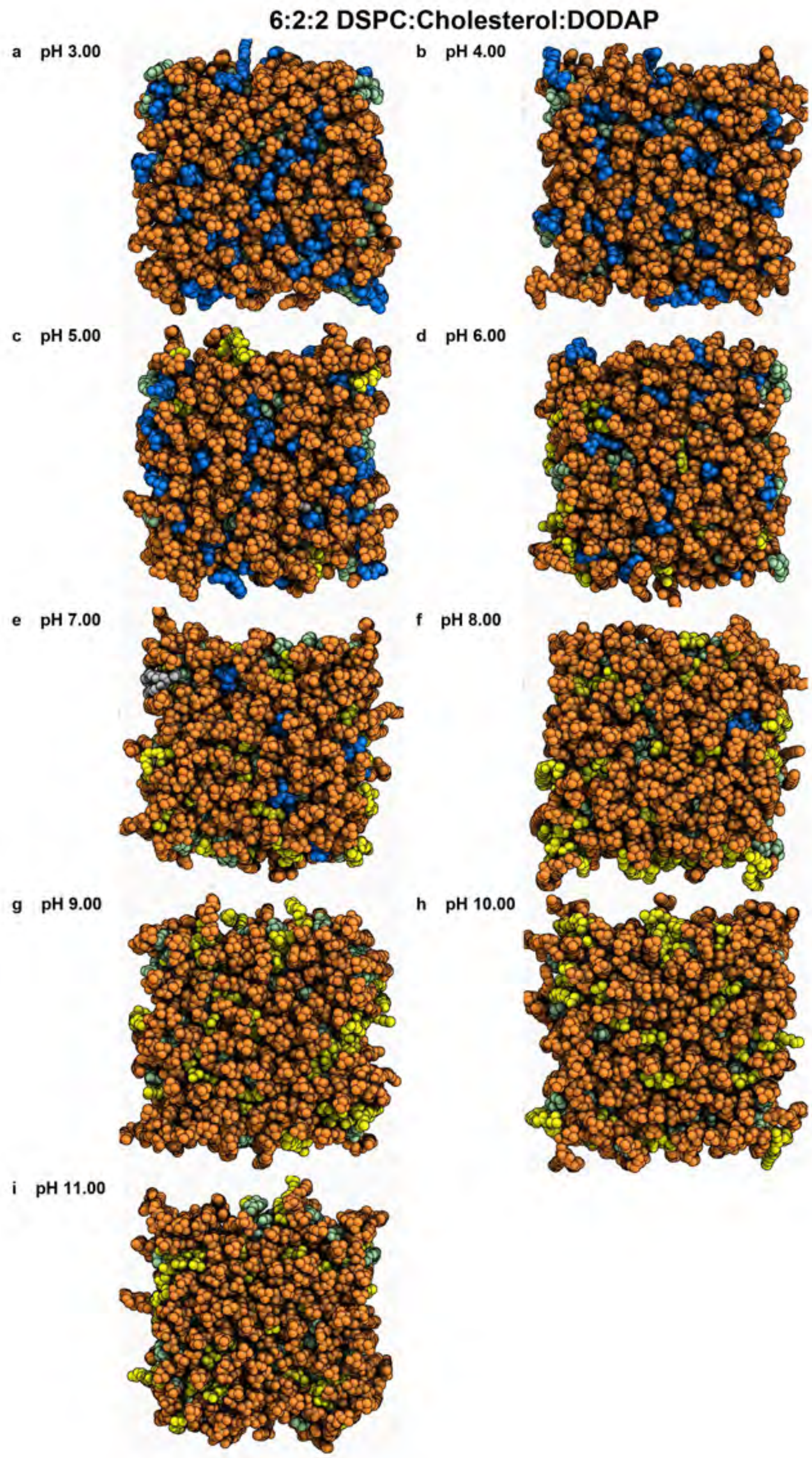

Figure S46: Top-view snapshots of the membrane in the final frame of the simulation at the corresponding pH values. The helper lipid is shown in orange, cholesterol in green, the protonated aminolipid (net charge +1) in blue, the deprotonated aminolipid (net charge 0) in yellow, and the aminolipids without a specific state ($0.2 \leq \lambda \leq 0.8$) in grey.

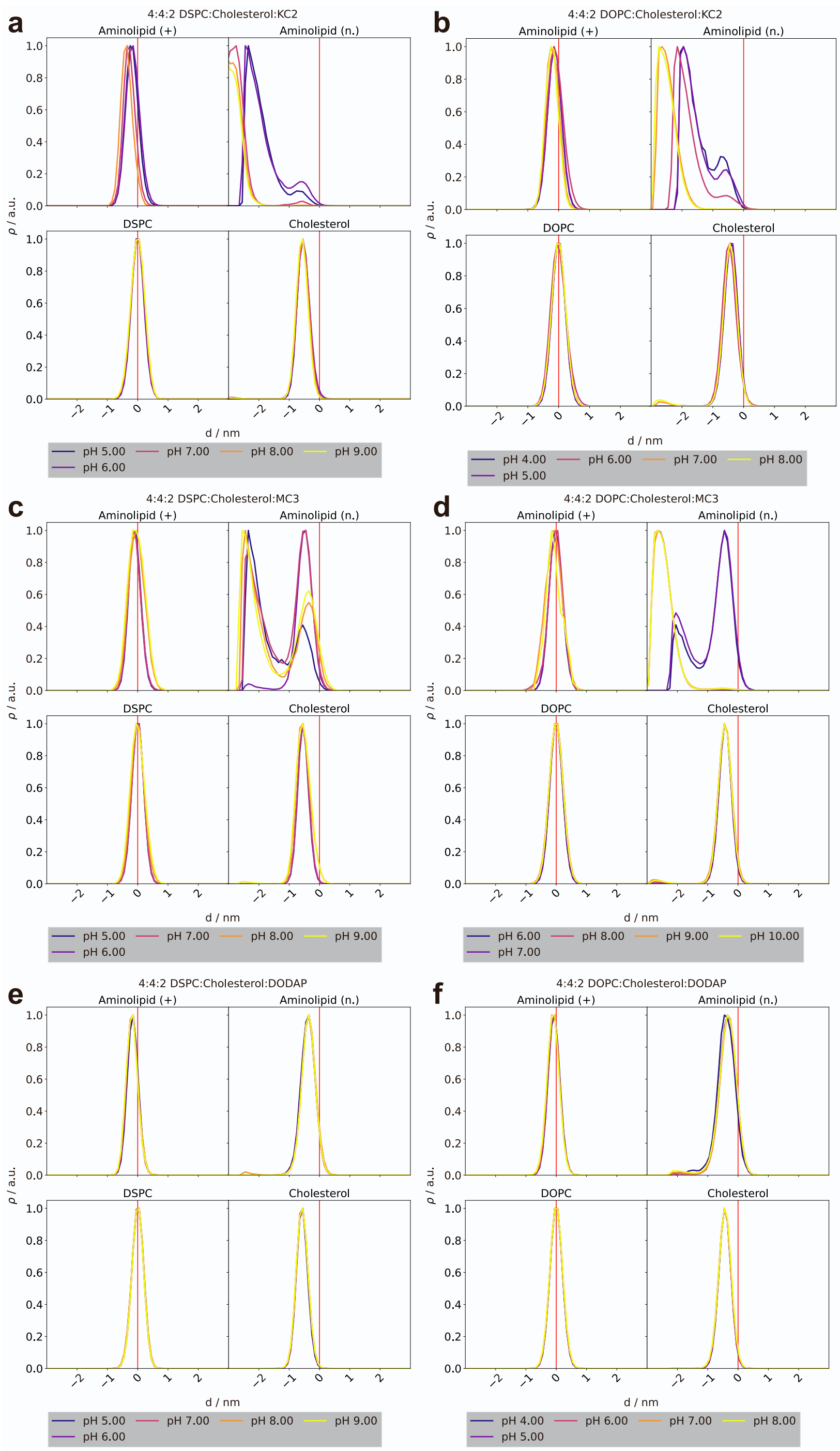

Figure S47: Lipid density profiles along the z-axis for membranes with a 4:4:2 helper lipid:cholesterol:aminolipid composition containing either **a**,**c**,**e** DSPC or **b**,**d**,**f** DOPC as the helper lipid, and **a**,**b** DLin-KC2-DMA, **c**,**d** DLin-MC3-DMA, or **e**,**f** DODAP as the aminolipid. Densities were calculated using one representative atom per lipid (oxygen for cholesterol, nitrogen for the aminolipid, and phosphorus for the helper lipid). Positions are reported relative to the membrane surface, defined as the average z-position of the phosphorus atoms in the helper lipid headgroups of each leaflet. Negative distances (d) correspond to the membrane core, whereas positive distances correspond to the solvent phase. Results were averaged over both leaflets.

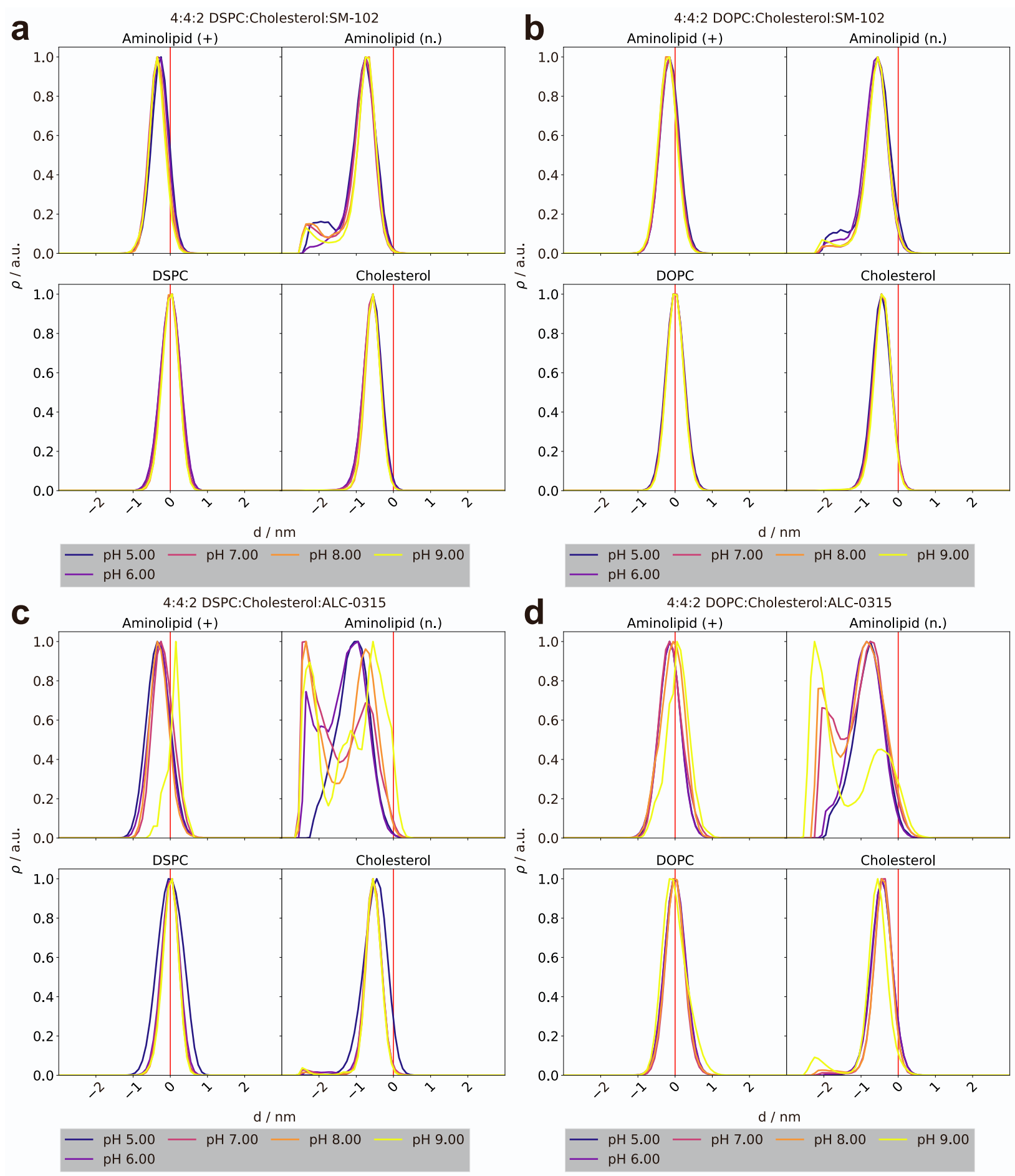

Figure S48: Lipid density profiles along the z-axis for membranes with a 4:4:2 helper lipid:cholesterol:aminolipid composition containing either **a**,**c** DSPC or **b**,**d** DOPC as the helper lipid, and **a**,**b** SM-102, or **c**,**d** ALC-0315 as the aminolipid. Densities were calculated using one representative atom per lipid (oxygen for cholesterol, nitrogen for the aminolipid, and phosphorus for the helper lipid). Positions are reported relative to the membrane surface, defined as the average z-position of the phosphorus atoms in the helper lipid headgroups of each leaflet. Negative distances (d) correspond to the membrane core, whereas positive distances correspond to the solvent phase. Results were averaged over both leaflets.

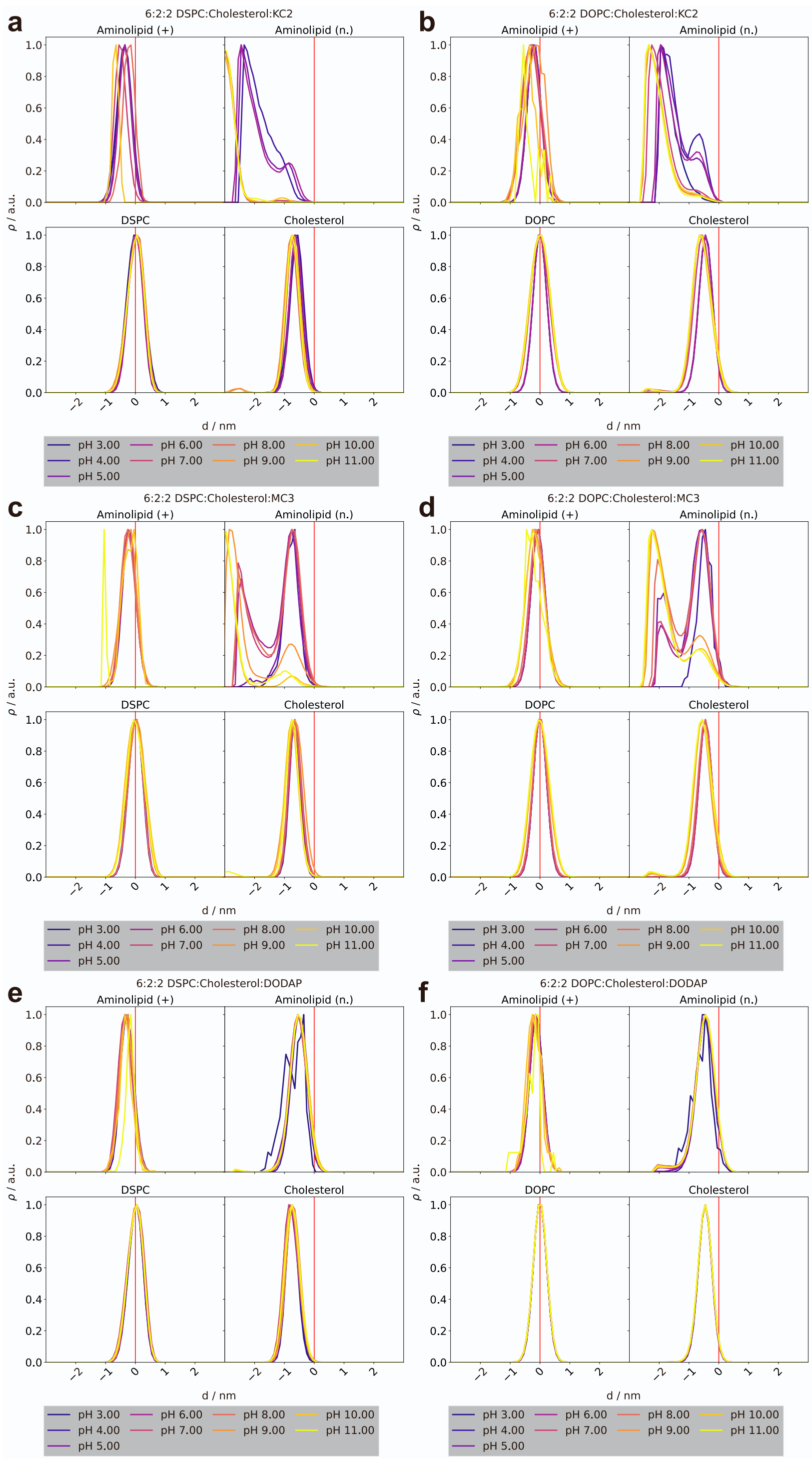

Figure S49: Lipid density profiles along the z-axis for membranes with a 6:2:2 helper lipid:cholesterol:aminolipid composition containing either **a**,**c**,**e** DSPC or **b**,**d**,**f** DOPC as the helper lipid, and **a**,**b** DLin-KC2-DMA, **c**,**d** DLin-MC3-DMA, or **e**,**f** DODAP as the aminolipid. Densities were calculated using one representative atom per lipid (oxygen for cholesterol, nitrogen for the aminolipid, and phosphorus for the helper lipid). Positions are reported relative to the membrane surface, defined as the average z-position of the phosphorus atoms in the helper lipid headgroups of each leaflet. Negative distances (d) correspond to the membrane core, whereas positive distances correspond to the solvent phase. Results were averaged over both leaflets.

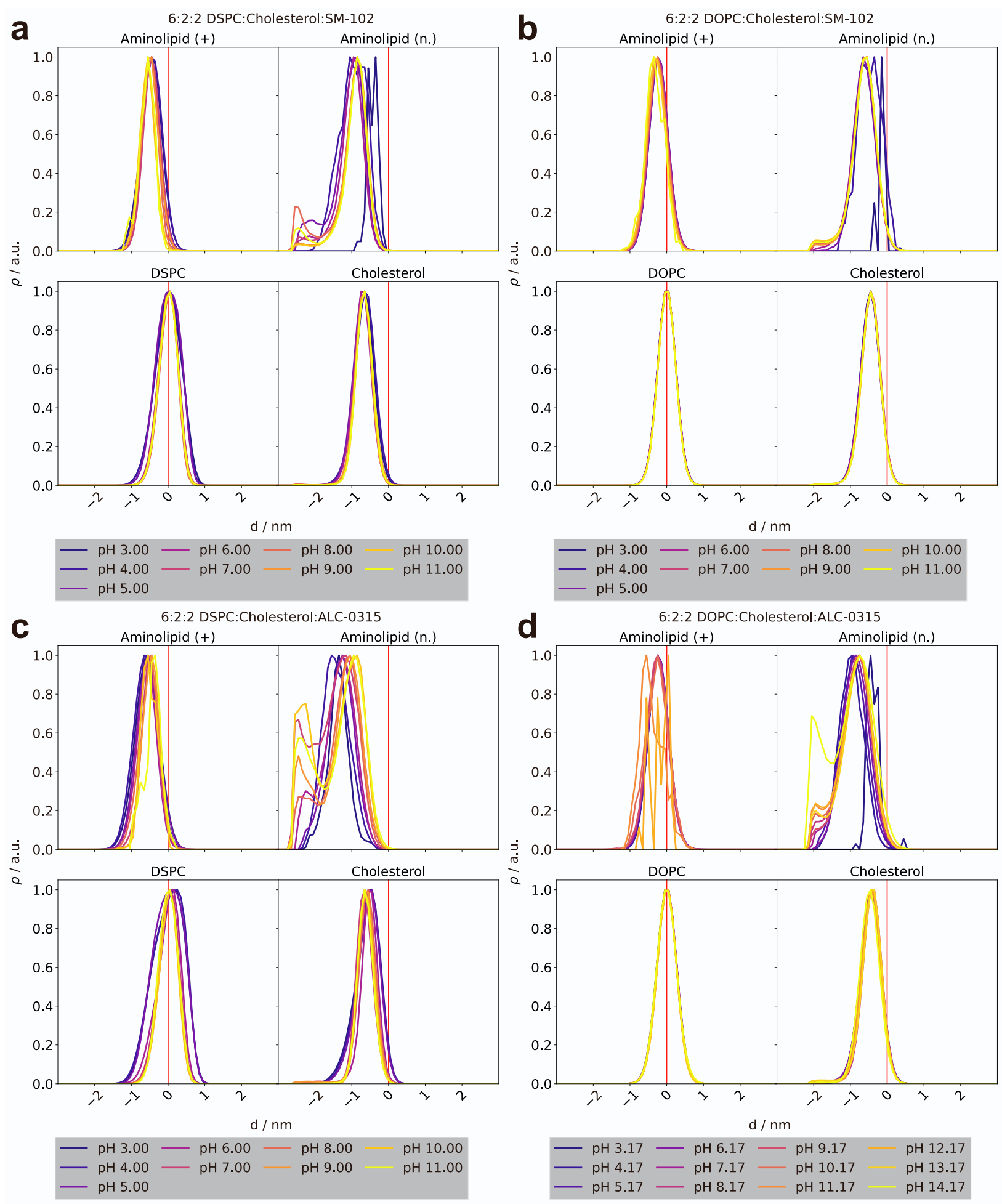

Figure S50: Lipid density profiles along the z-axis for membranes with a 6:2:2 helper lipid:cholesterol:aminolipid composition containing either **a**,**c** DSPC or **b**,**d** DOPC as the helper lipid, and **a**,**b** SM-102, or **c**,**d** ALC-0315 as the aminolipid. Densities were calculated using one representative atom per lipid (oxygen for cholesterol, nitrogen for the aminolipid, and phosphorus for the helper lipid). Positions are reported relative to the membrane surface, defined as the average z-position of the phosphorus atoms in the helper lipid headgroups of each leaflet. Negative distances (d) correspond to the membrane core, whereas positive distances correspond to the solvent phase. Results were averaged over both leaflets.

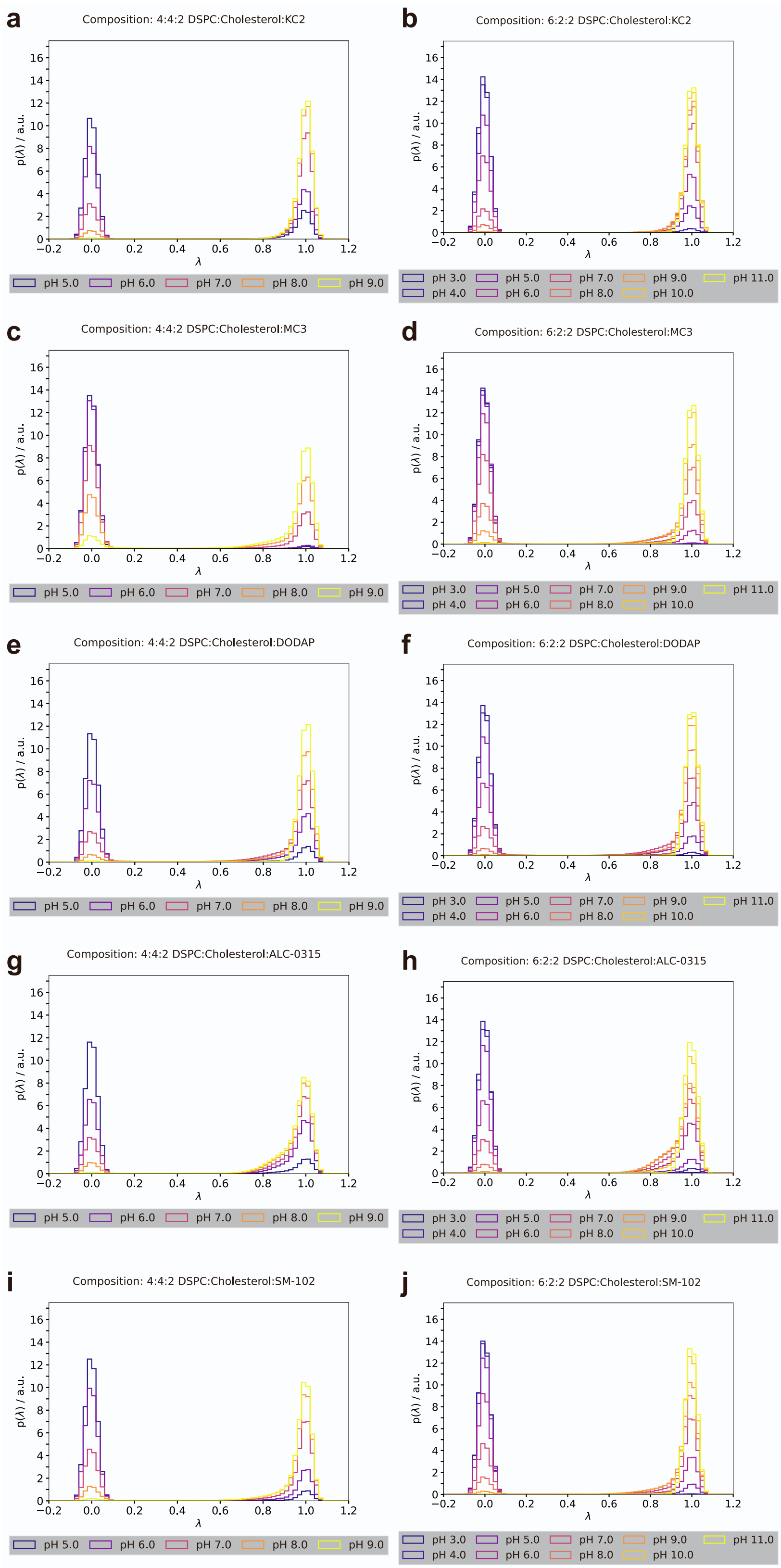

Figure S51: Distribution of the λ-coordinate at each pH, aggregated over all aminolipids in a membrane with **a**,**c**,**e**,**g**,**i** a 4:4:2 or **b**,**d**,**f**,**h**,**j** a 6:2:2 DSPC:cholesterol:aminolipid composition containing either **a**,**b** DLin-KC2-DMA, **c**,**d** DLin-MC3-DMA, **e**,**f** DODAP, **g**,**h** ALC-0315 or **i**,**j** SM-102 as the aminolipid.

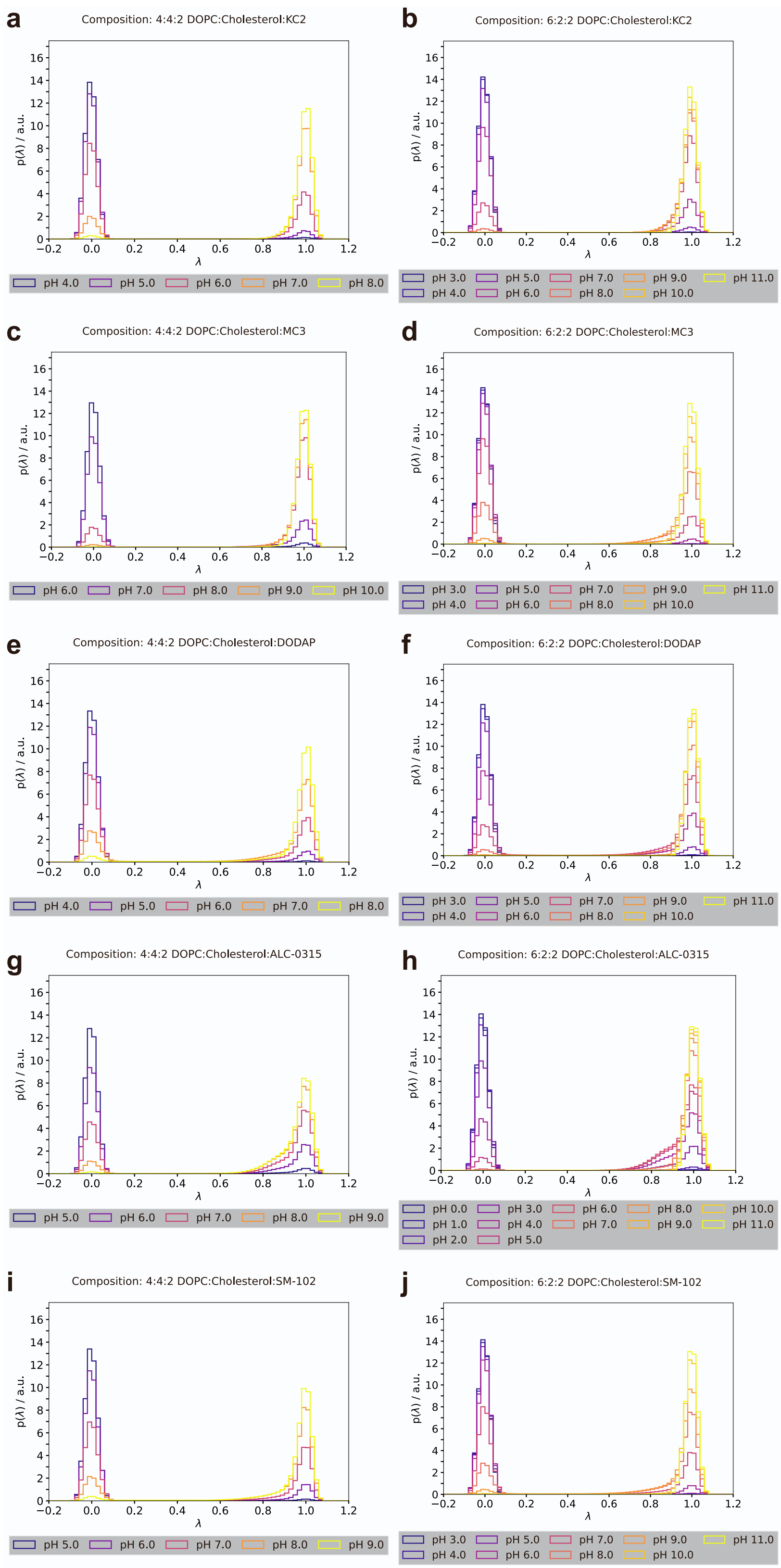

Figure S52: Distribution of the λ-coordinate at each pH, aggregated over all aminolipids in a membrane with **a**,**c**,**e**,**g**,**i** a 4:4:2 or **b**,**d**,**f**,**h**,**j** a 6:2:2 DOPC:cholesterol:aminolipid composition containing either **a**,**b** DLin-KC2-DMA, **c**,**d** DLin-MC3-DMA, **e**,**f** DODAP, **g**,**h** ALC-0315 or **i**,**j** SM-102 as the aminolipid.

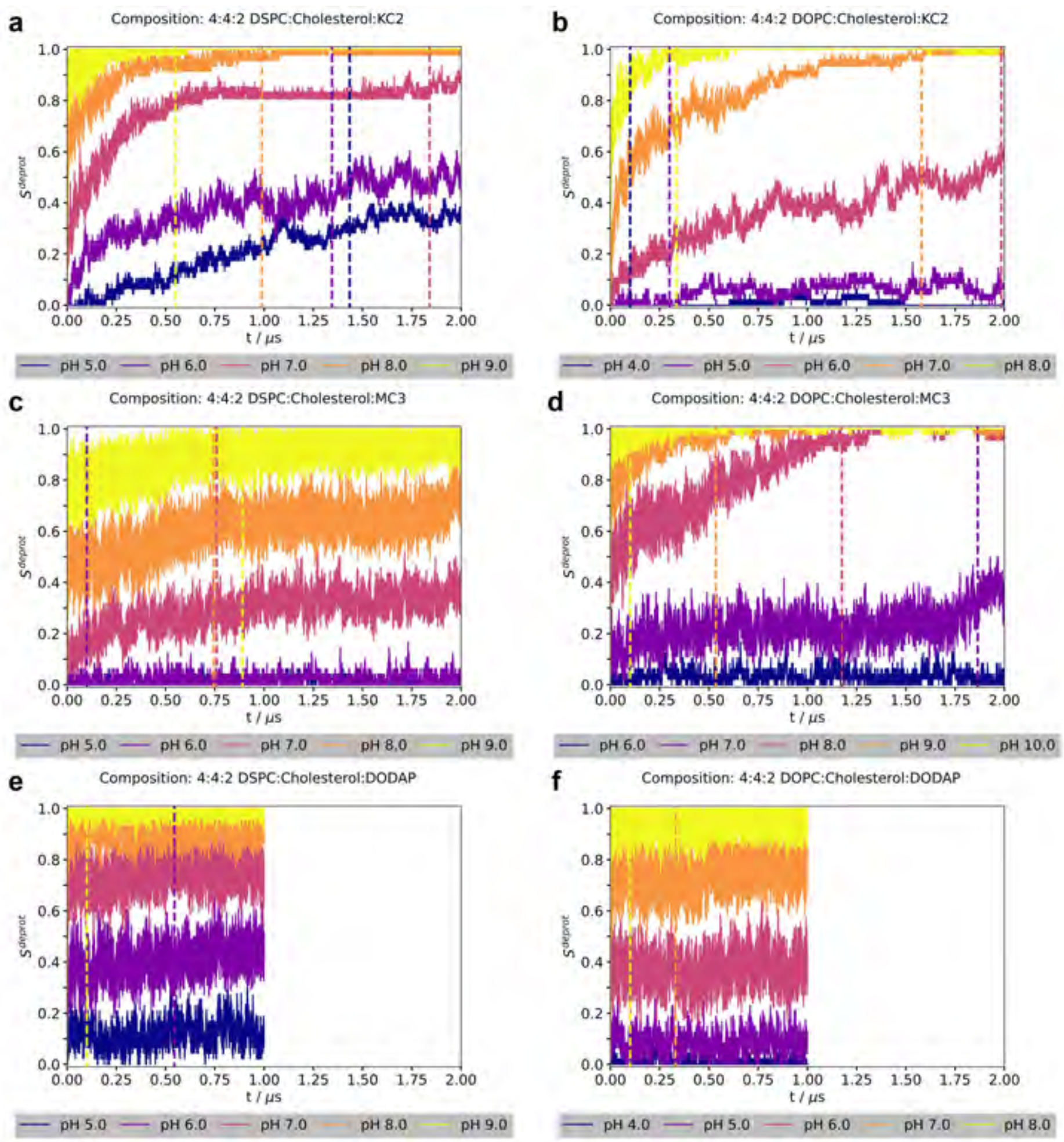

Figure S53: Fraction of deprotonated aminolipids (S^{deprot}) per frame for membranes with a 4:4:2 helper lipid:cholesterol:aminolipid composition containing either **a,c,e** DSPC or **b,d,f** DOPC as the helper lipid, and **a,b** DLin-KC2-DMA, **c,d** DLin-MC3-DMA, or **e,f** DODAP as the aminolipid. Vertical dashed lines indicate the equilibration time determined for each pH value.

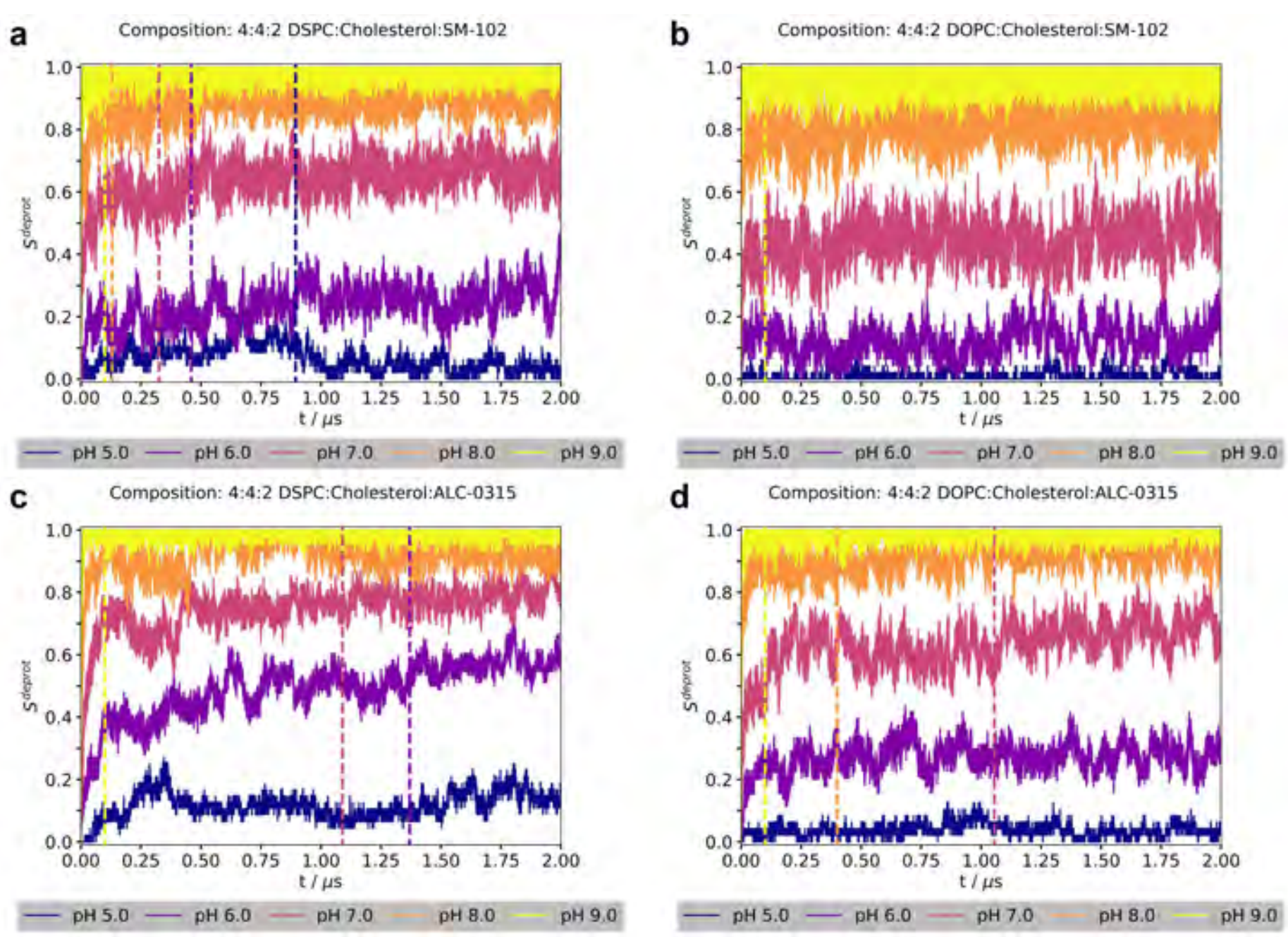

Figure S54: Fraction of deprotonated aminolipids (S^{deprot}) per frame for membranes with a 4:4:2 helper lipid:cholesterol:aminolipid composition containing either **a,c** DSPC or **b,d** DOPC as the helper lipid, and **a,b** SM-102, or **c,d** ALC-0315 as the aminolipid. Vertical dashed lines indicate the equilibration time determined for each pH value.

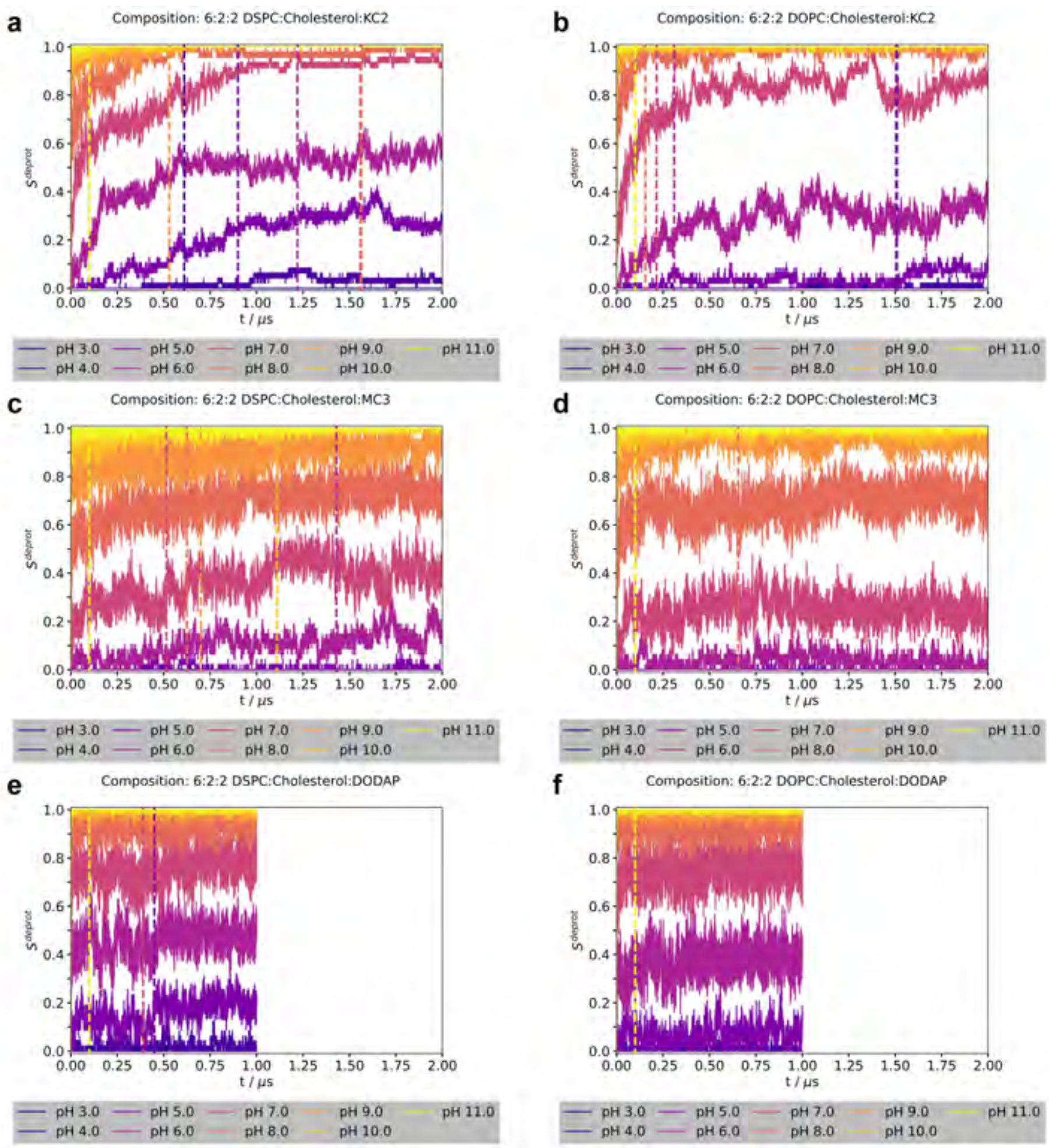

Figure S55: Fraction of deprotonated aminolipids (S^{deprot}) per frame for membranes with a 6:2:2 helper lipid:cholesterol:aminolipid composition containing either **a,c,e** DSPC or **b,d,f** DOPC as the helper lipid, and **a,b** DLin-KC2-DMA, **c,d** DLin-MC3-DMA, or **e,f** DODAP as the aminolipid. Vertical dashed lines indicate the equilibration time determined for each pH value.

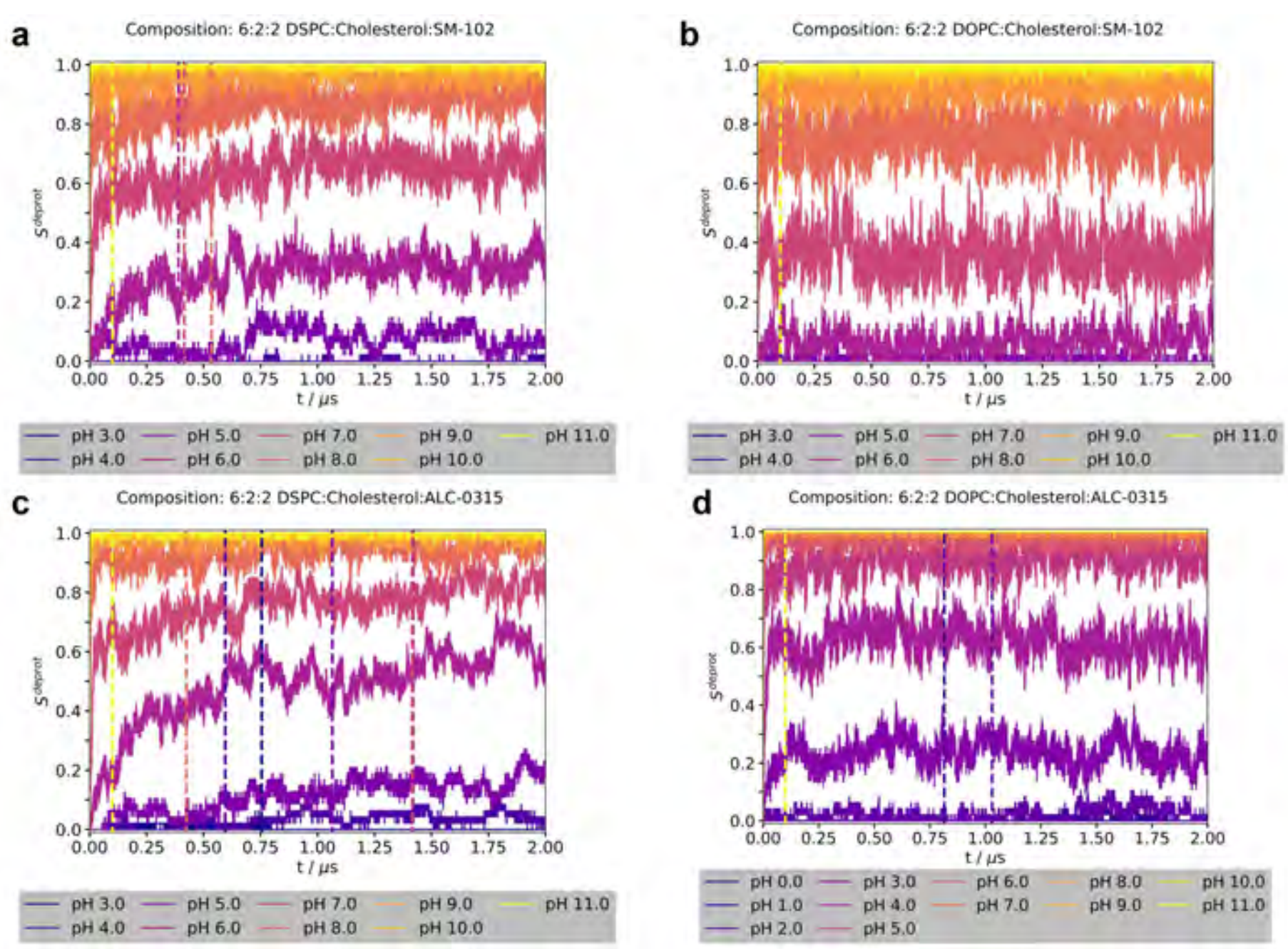

Figure S56: Fraction of deprotonated aminolipids (S^{deprot}) per frame for membranes with a 6:2:2 helper lipid:cholesterol:aminolipid composition containing either **a,c** DSPC or **b,d** DOPC as the helper lipid, and **a,b** SM-102, or **c,d** ALC-0315 as the aminolipid. Vertical dashed lines indicate the equilibration time determined for each pH value.

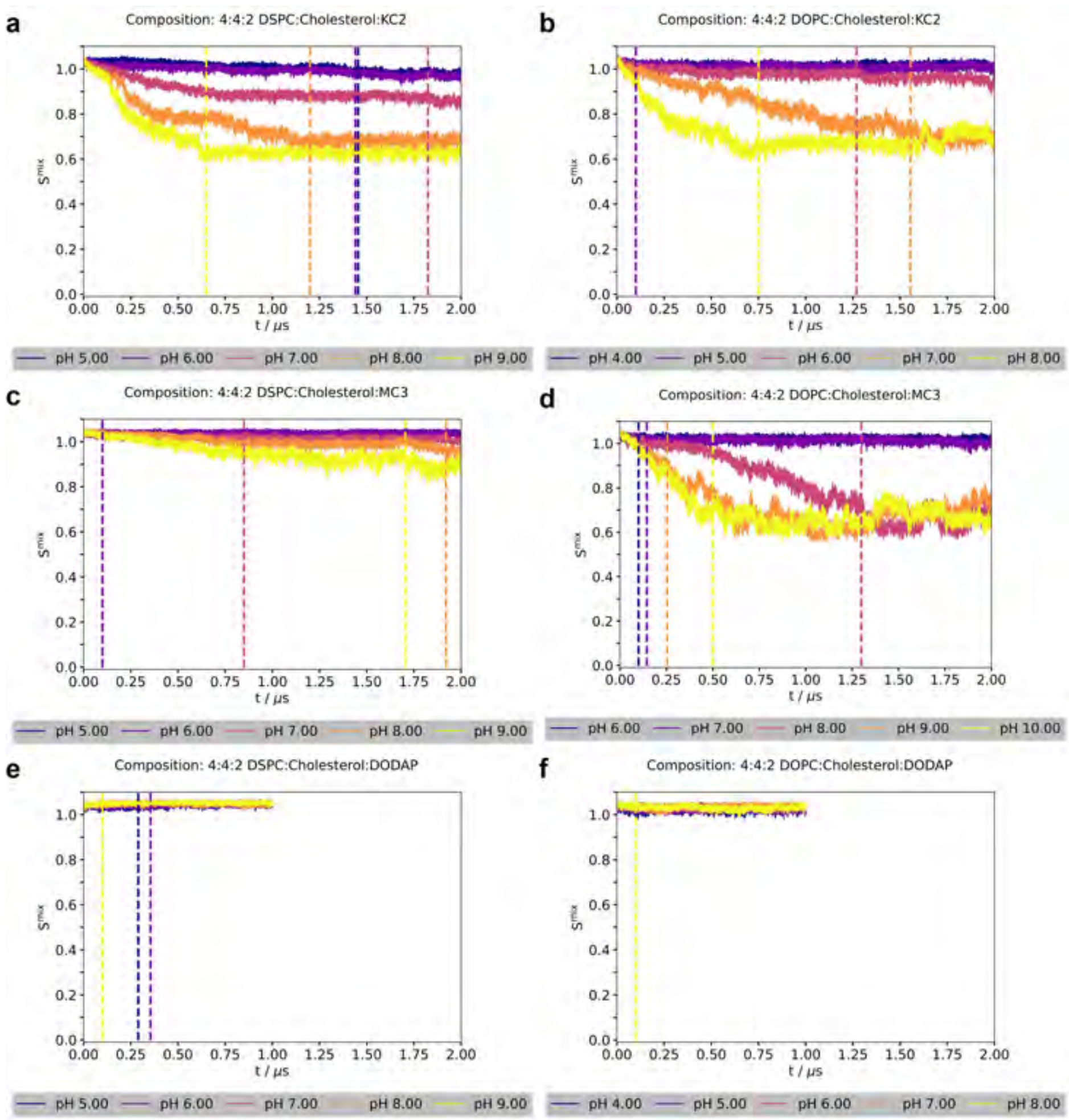

Figure S57: Conditional mixing entropy (S^{mix}) per frame for membranes with a 4:4:2 helper lipid:cholesterol:aminolipid composition containing either **a,c,e** DSPC or **b,d,f** DOPC as the helper lipid, and **a,b** DLin-KC2-DMA, **c,d** DLin-MC3-DMA, or **e,f** DODAP as the aminolipid. Vertical dashed lines indicate the equilibration time determined for each pH value. Conditional mixing entropies were calculated following Brandani *et al.*,[3] using a 1 nm cutoff to define neighboring lipids. Contacts were defined based on the distance between reference atoms in the headgroups of the target lipids: the titratable nitrogen for aminolipids, the oxygen atom for cholesterol, and the sn-2 carbon atom for phospholipids.

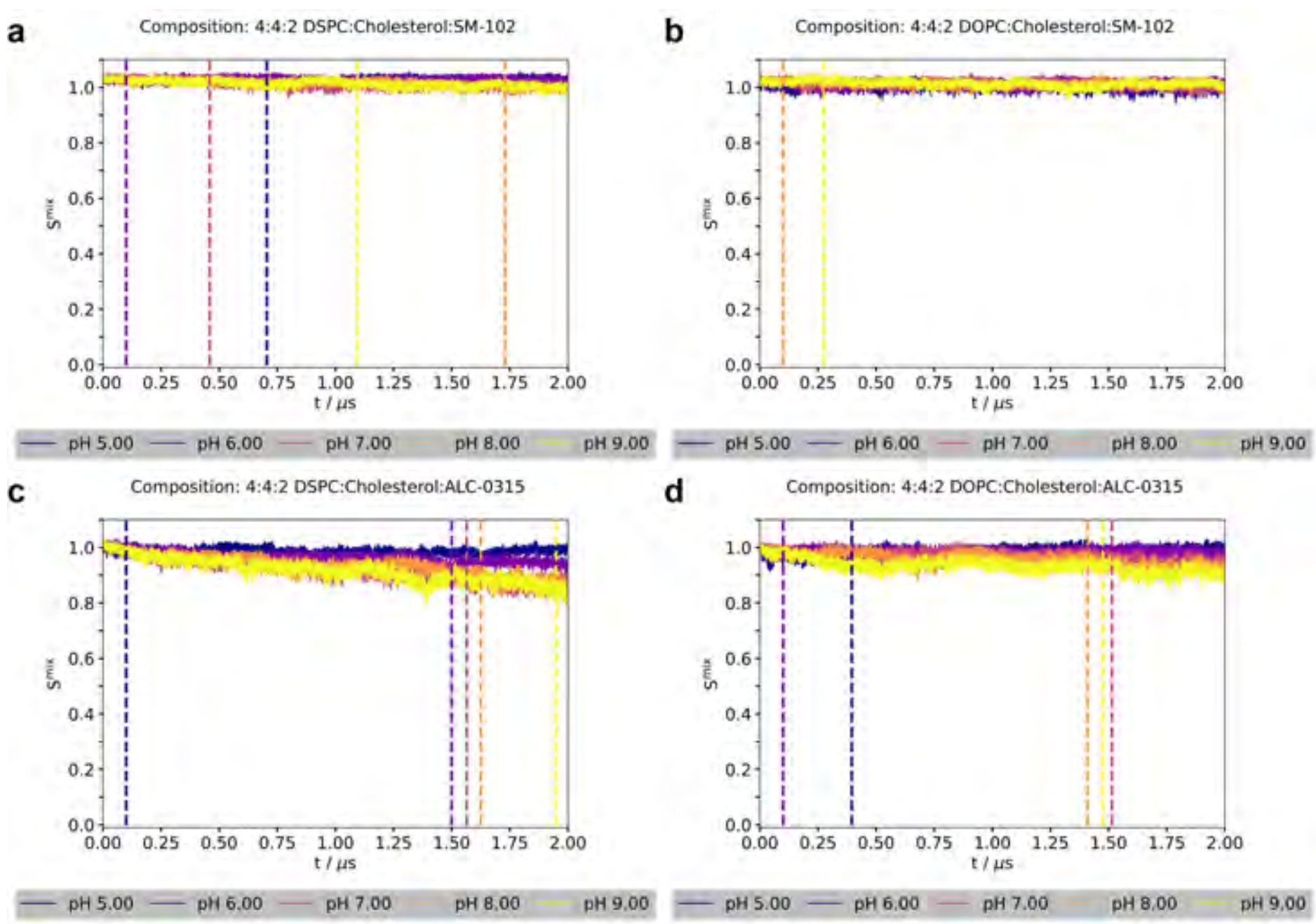

Figure S58: Conditional mixing entropy (S^{mix}) per frame for membranes with a 4:4:2 helper lipid:cholesterol:aminolipid composition containing either **a,c** DSPC or **b,d** DOPC as the helper lipid, and **a,b** SM-102, or **c,d** ALC-0315 as the aminolipid. Vertical dashed lines indicate the equilibration time determined for each pH value. Conditional mixing entropies were calculated following Brandani *et al.*,[3] using a 1 nm cutoff to define neighboring lipids. Contacts were defined based on the distance between reference atoms in the headgroups of the target lipids: the titratable nitrogen for aminolipids, the oxygen atom for cholesterol, and the sn-2 carbon atom for phospholipids.

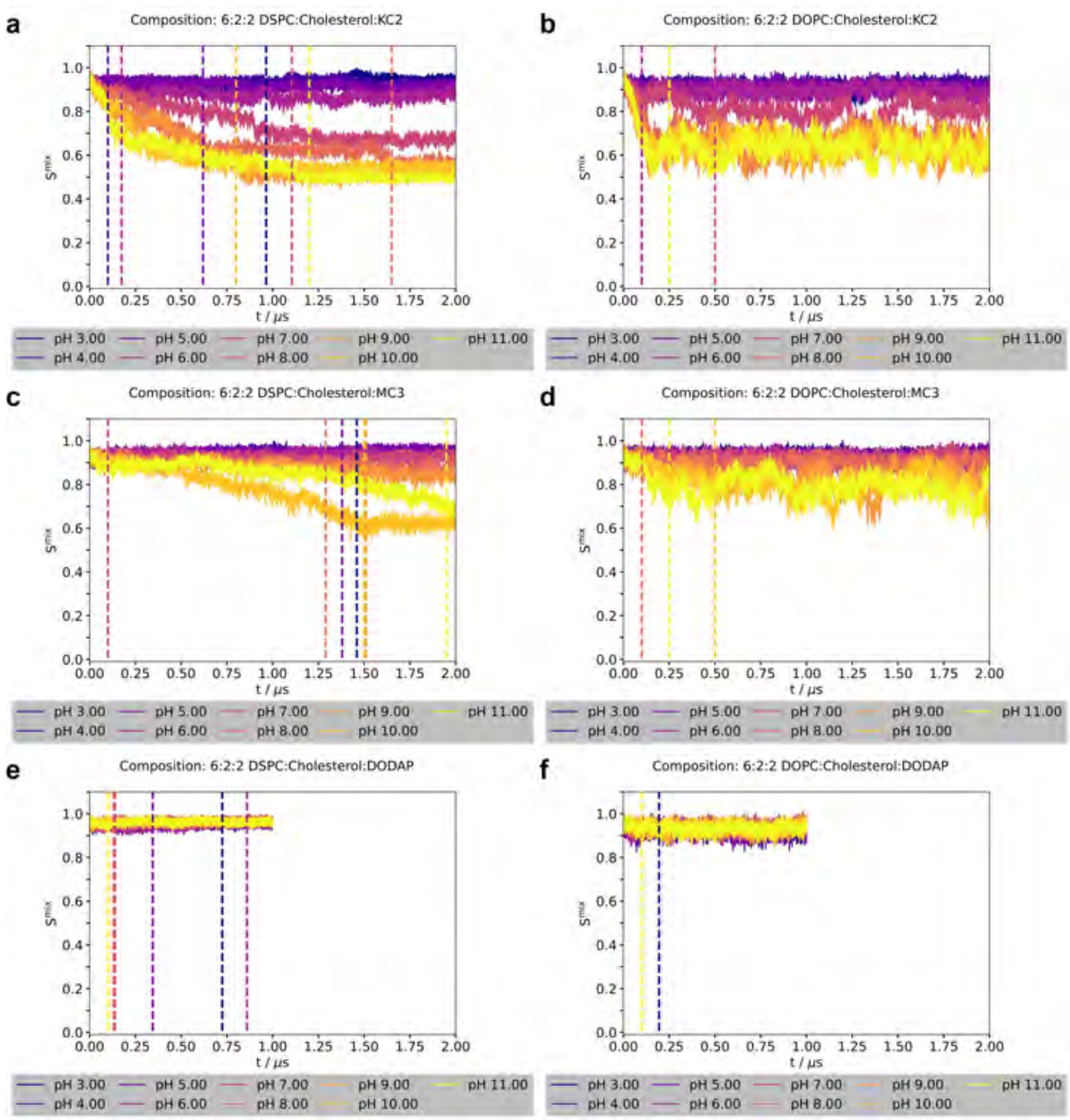

Figure S59: Conditional mixing entropy (S^{mix}) per frame for membranes with a 6:2:2 helper lipid:cholesterol:aminolipid composition containing either **a,c,e** DSPC or **b,d,f** DOPC as the helper lipid, and **a,b** DLin-KC2-DMA, **c,d** DLin-MC3-DMA, or **e,f** DODAP as the aminolipid. Vertical dashed lines indicate the equilibration time determined for each pH value. Conditional mixing entropies were calculated following Brandani *et al.*,[3] using a 1 nm cutoff to define neighboring lipids. Contacts were defined based on the distance between reference atoms in the headgroups of the target lipids: the titratable nitrogen for aminolipids, the oxygen atom for cholesterol, and the sn-2 carbon atom for phospholipids.

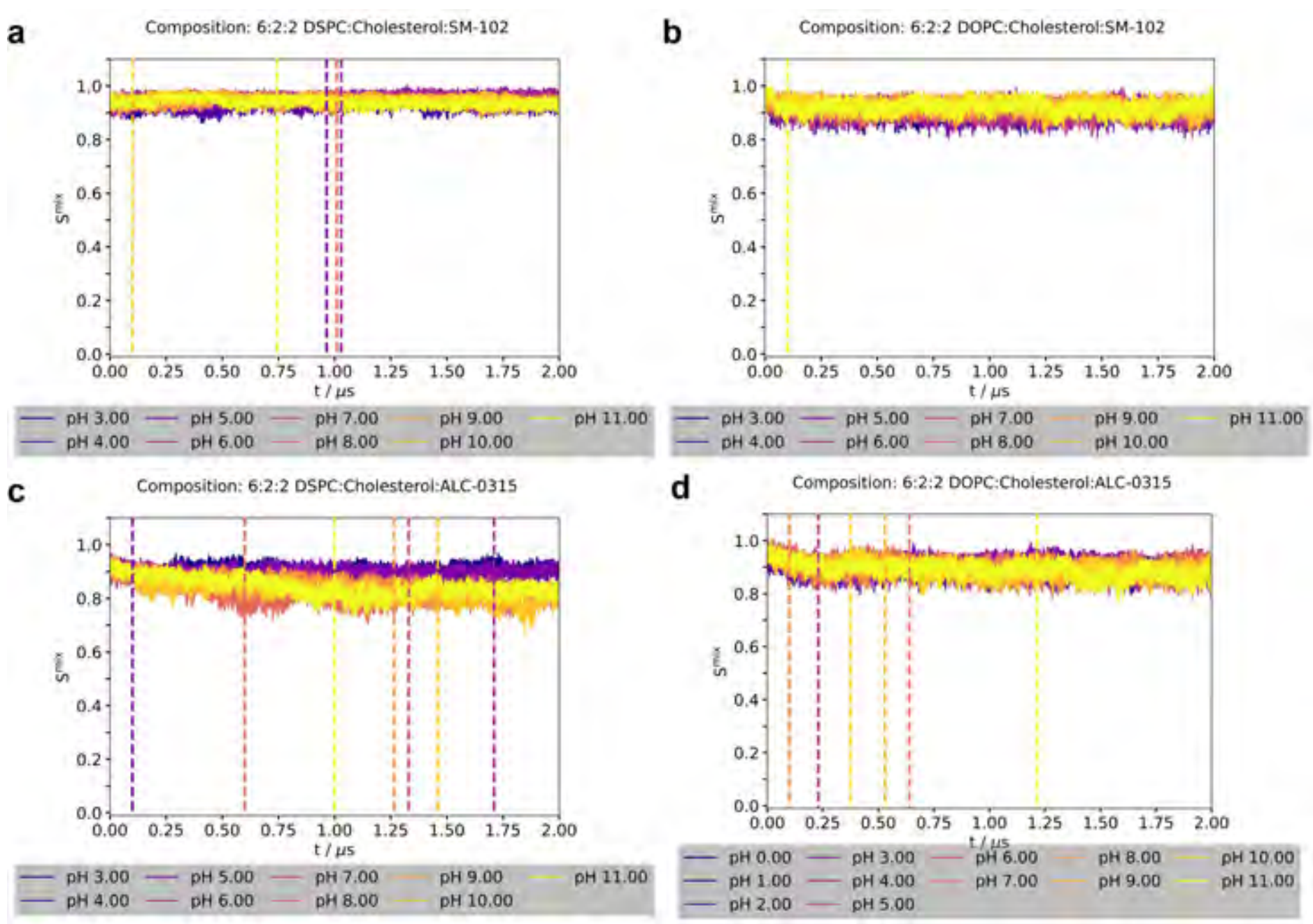

Figure S60: Conditional mixing entropy (S^{mix}) per frame for membranes with a 6:2:2 helper lipid:cholesterol:aminolipid composition containing either **a,c** DSPC or **b,d** DOPC as the helper lipid, and **a,b** SM-102, or **c,d** ALC-0315 as the aminolipid. Vertical dashed lines indicate the equilibration time determined for each pH value. Conditional mixing entropies were calculated following Brandani *et al.*,[3] using a 1 nm cutoff to define neighboring lipids. Contacts were defined based on the distance between reference atoms in the headgroups of the target lipids: the titratable nitrogen for aminolipids, the oxygen atom for cholesterol, and the sn-2 carbon atom for phospholipids.

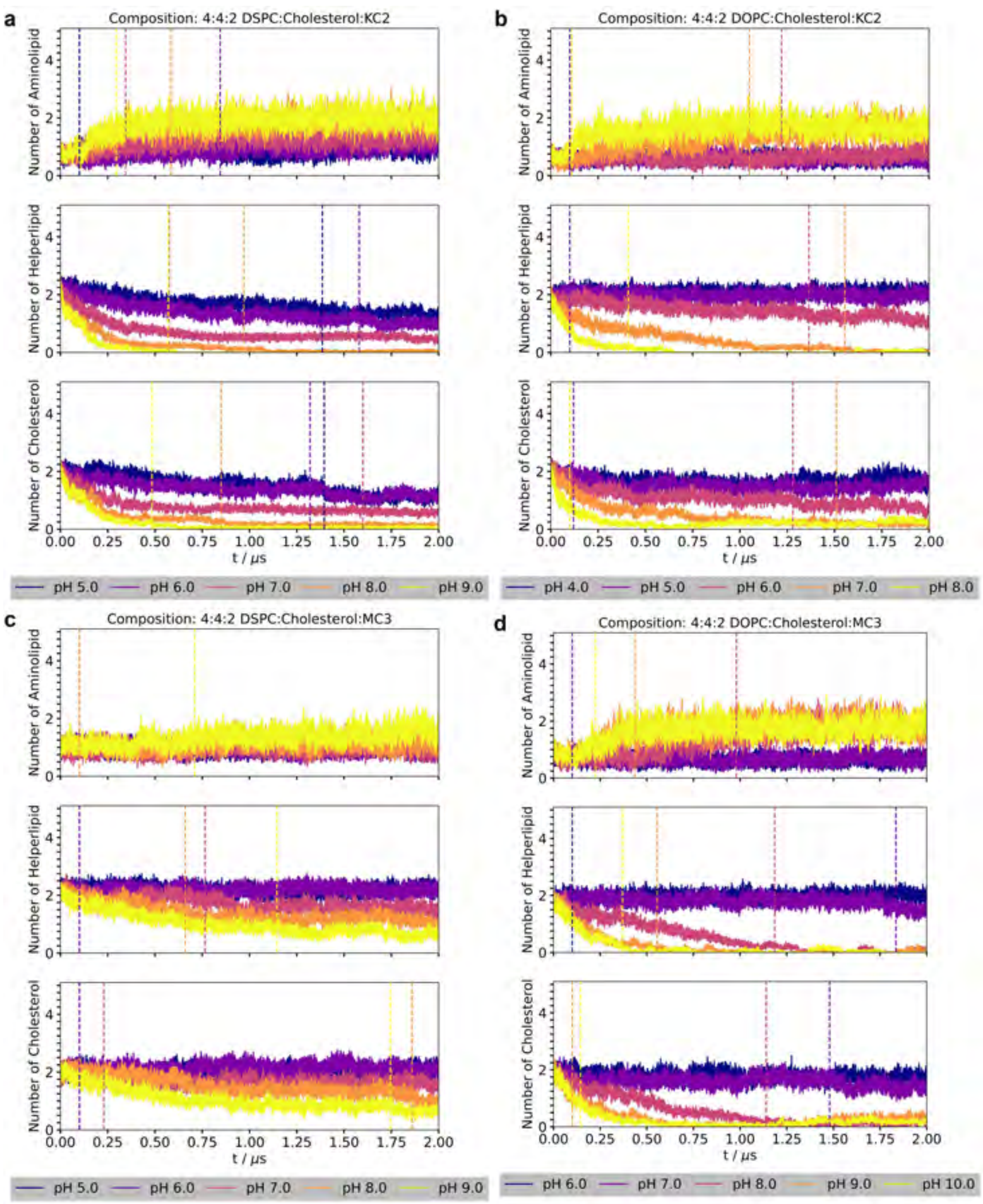

Figure S61: Number of neighboring lipids per aminolipid per frame for membranes with a 4:4:2 helper lipid:cholesterol:aminolipid composition containing either **a,c** DSPC or **b,d** DOPC as the helper lipid, and **a,b** DLin-KC2-DMA, or **c,d** DLin-MC3-DMA as the aminolipid. Vertical dashed lines indicate the equilibration time determined for each pH value. A cutoff distance of 1 nm was used to define neighboring lipids. Contacts were defined based on the distance between reference atoms in the lipid headgroups: the titratable nitrogen atom for aminolipids, the oxygen atom for cholesterol, and the sn-2 carbon atom for phospholipids.

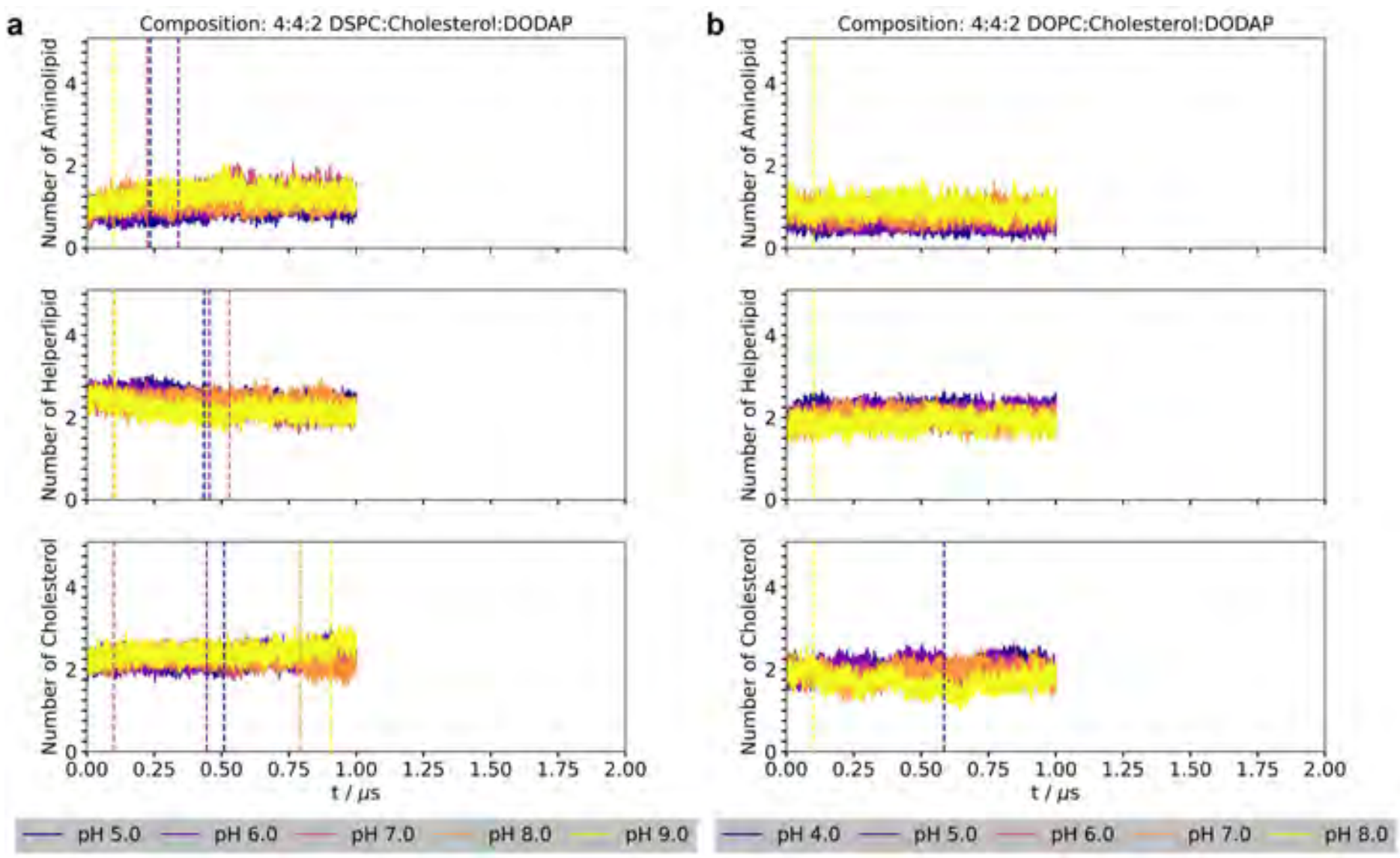

Figure S62: Number of neighboring lipids per aminolipid per frame for membranes with a 4:4:2 helper lipid:cholesterol:aminolipid composition containing either **a** DSPC or **b** DOPC as the helper lipid, and DODAP as the aminolipid. Vertical dashed lines indicate the equilibration time determined for each pH value. A cutoff distance of 1 nm was used to define neighboring lipids. Contacts were defined based on the distance between reference atoms in the lipid headgroups: the titratable nitrogen atom for aminolipids, the oxygen atom for cholesterol, and the sn-2 carbon atom for phospholipids.

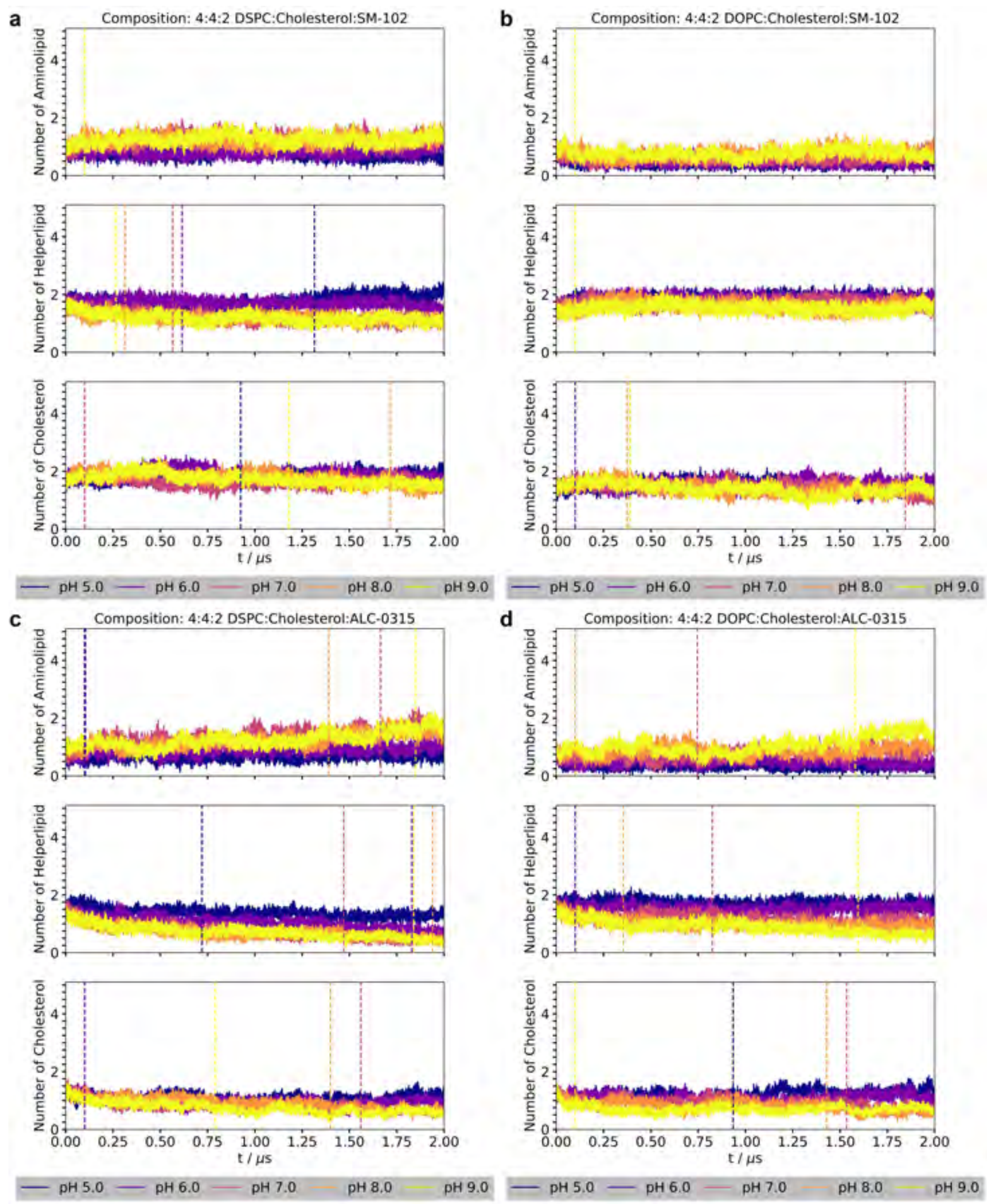

Figure S63: Number of neighboring lipids per aminolipid per frame for membranes with a 4:4:2 helper lipid:cholesterol:aminolipid composition containing either **a,c** DSPC or **b,d** DOPC as the helper lipid, and **a,b** SM-102, or **c,d** ALC-0315 as the aminolipid. Vertical dashed lines indicate the equilibration time determined for each pH value. A cutoff distance of 1 nm was used to define neighboring lipids. Contacts were defined based on the distance between reference atoms in the lipid headgroups: the titratable nitrogen atom for aminolipids, the oxygen atom for cholesterol, and the sn-2 carbon atom for phospholipids.

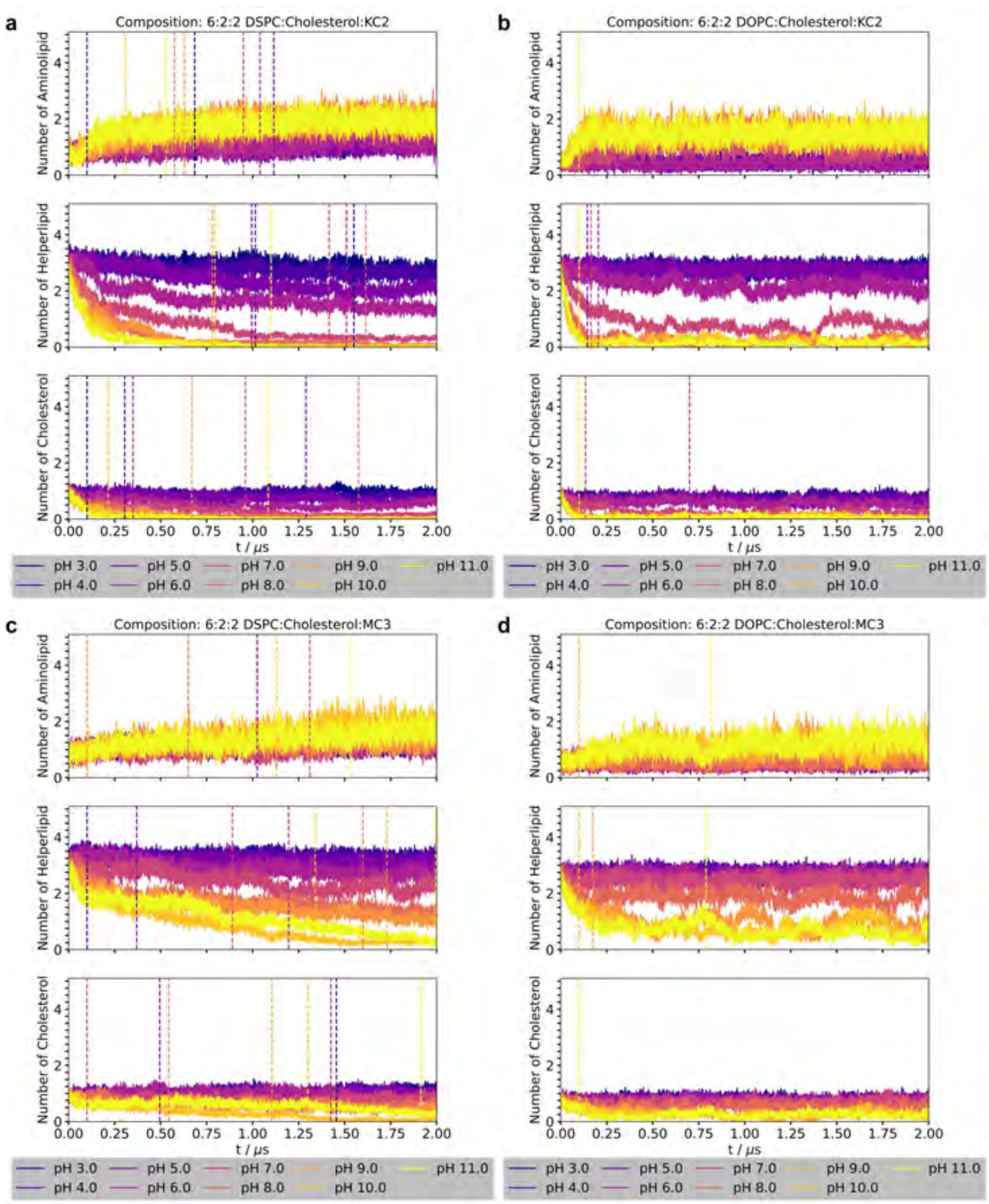

Figure S64: Number of neighboring lipids per aminolipid per frame for membranes with a 6:2:2 helper lipid:cholesterol:aminolipid composition containing either **a,c** DSPC or **b,d** DOPC as the helper lipid, and **a,b** DLin-KC2-DMA, or **c,d** DLin-MC3-DMA as the aminolipid. Vertical dashed lines indicate the equilibration time determined for each pH value. A cutoff distance of 1 nm was used to define neighboring lipids. Contacts were defined based on the distance between reference atoms in the lipid headgroups: the titratable nitrogen atom for aminolipids, the oxygen atom for cholesterol, and the sn-2 carbon atom for phospholipids.

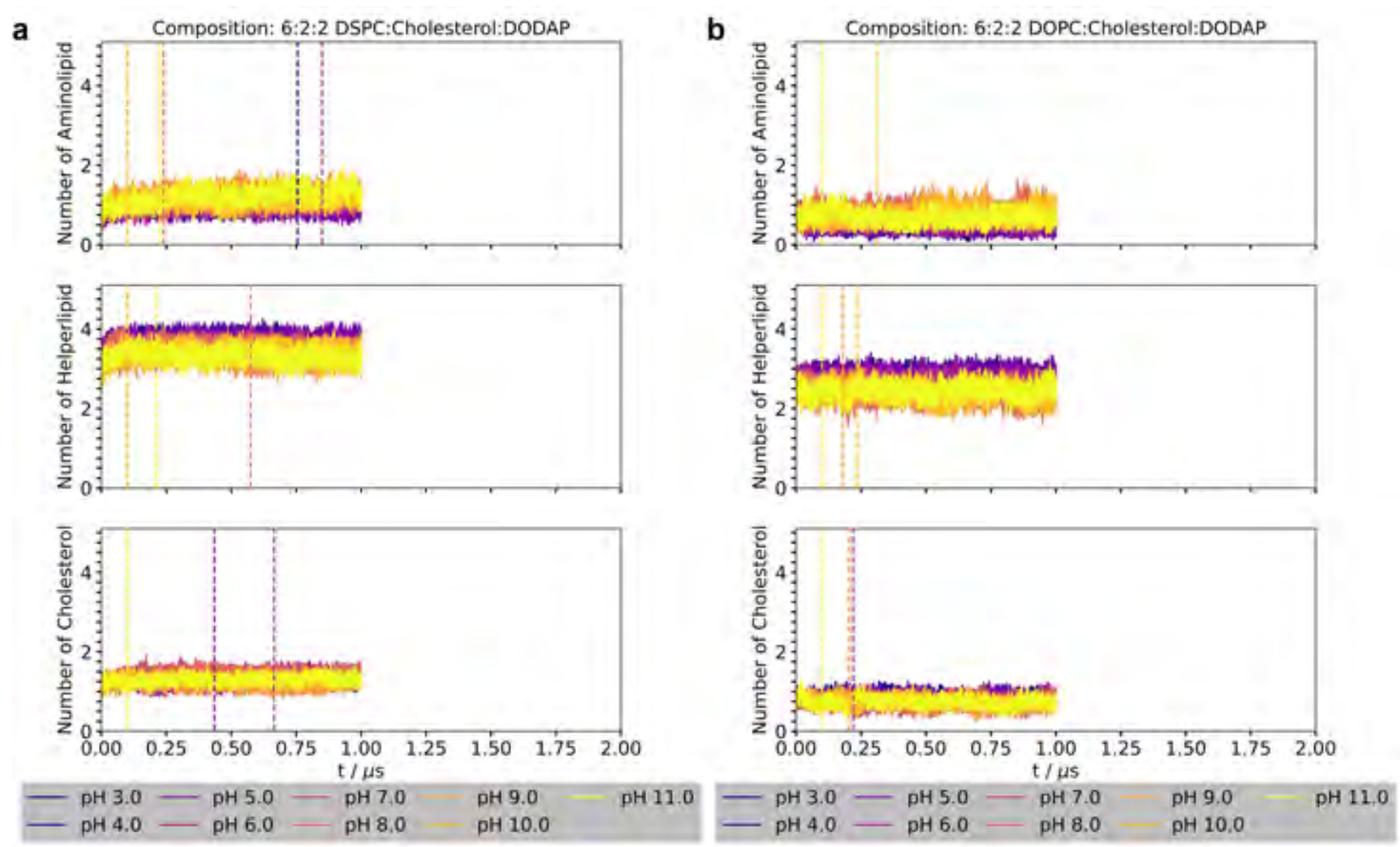

Figure S65: Number of neighboring lipids per aminolipid per frame for membranes with a 6:2:2 helper lipid:cholesterol:aminolipid composition containing either **a** DSPC or **b** DOPC as the helper lipid, and DODAP as the aminolipid. Vertical dashed lines indicate the equilibration time determined for each pH value. A cutoff distance of 1 nm was used to define neighboring lipids. Contacts were defined based on the distance between reference atoms in the lipid headgroups: the titratable nitrogen atom for aminolipids, the oxygen atom for cholesterol, and the sn-2 carbon atom for phospholipids.

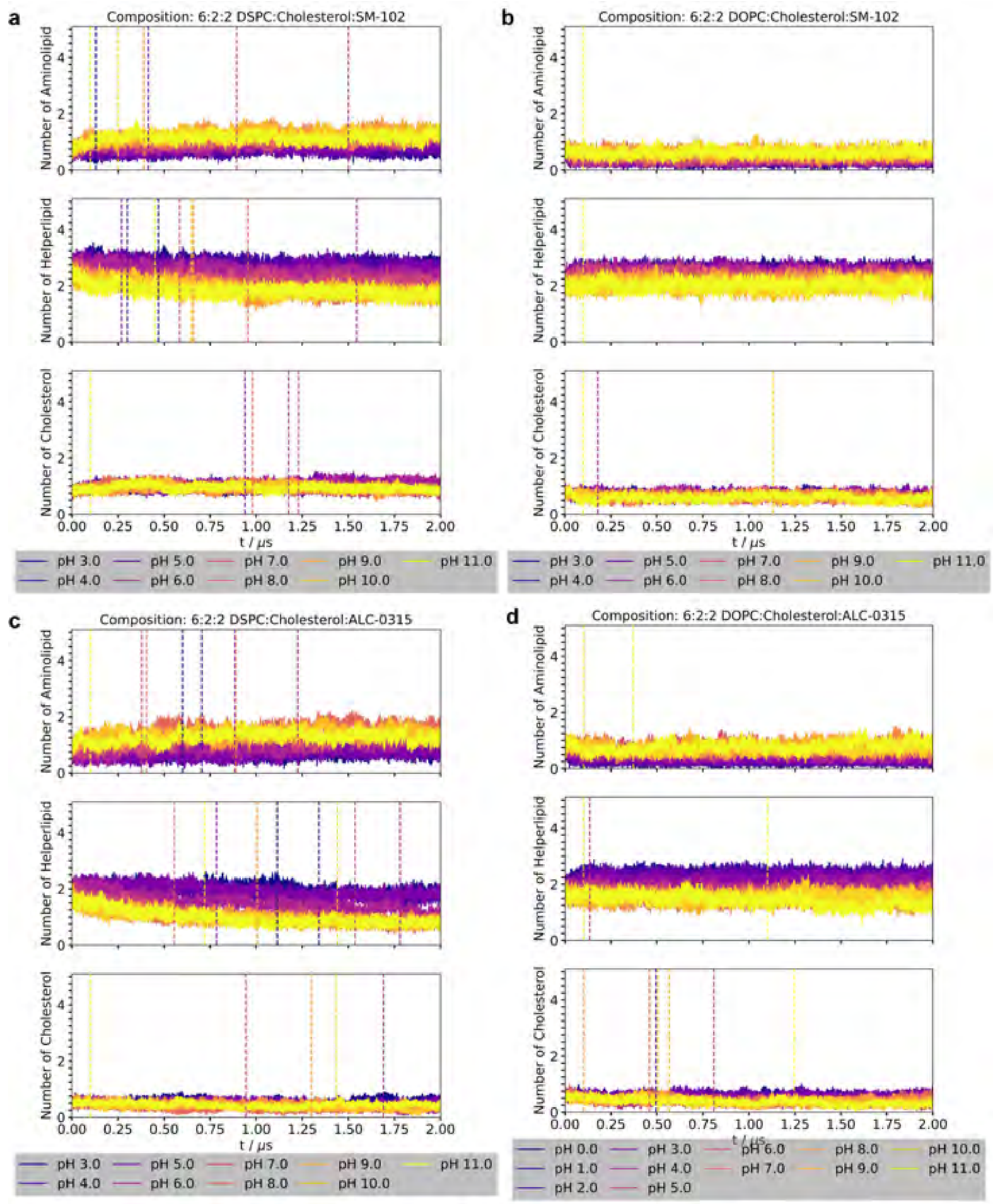

Figure S66: Number of neighboring lipids per aminolipid per frame for membranes with a 6:2:2 helper lipid:cholesterol:aminolipid composition containing either **a,c** DSPC or **b,d** DOPC as the helper lipid, and **a,b** SM-102, or **c,d** ALC-0315 as the aminolipid. Vertical dashed lines indicate the equilibration time determined for each pH value. A cutoff distance of 1 nm was used to define neighboring lipids. Contacts were defined based on the distance between reference atoms in the lipid headgroups: the titratable nitrogen atom for aminolipids, the oxygen atom for cholesterol, and the sn-2 carbon atom for phospholipids.

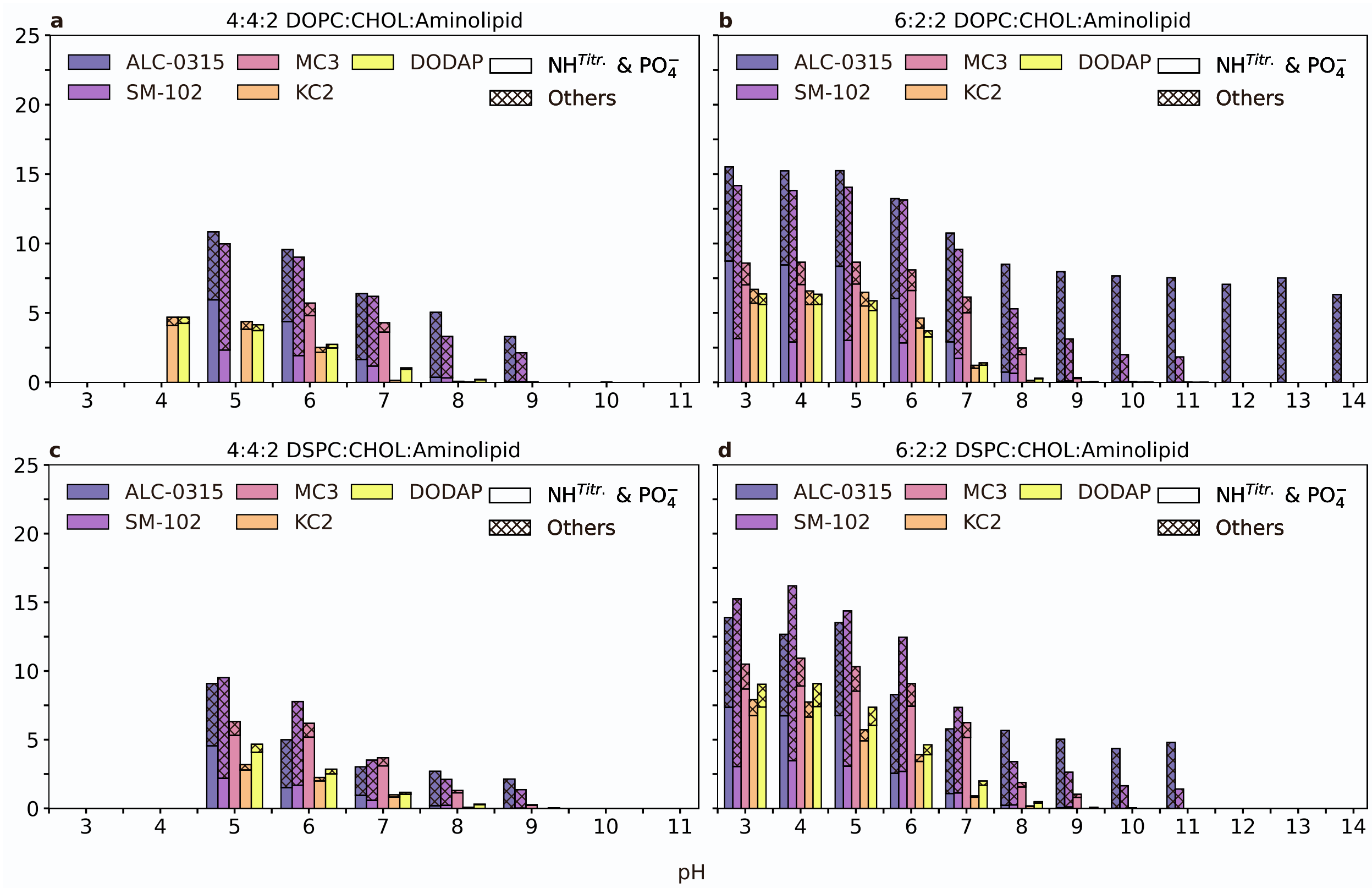

Figure S67: Average number of hydrogen bonds between aminolipids and helper lipids as a function of pH for DOPC-based (top) or DSPC-based (bottom) membranes containing **a**,**c** 40 mol% cholesterol and **b**,**d** 20 mol% cholesterol. Hydrogen bonds were separated into interactions between the titratable site of the aminolipids and phospholipid phosphate groups ("NH$^{Titr.}$ & PO_4^-"), and all remaining hydrogen bonds **not** involving this specific donor-acceptor pair ("Others"). Hydrogen bonds were identified using the MDAnalysis library (v2.7.0) with a distance cutoff of 3.0 Å and an angle cutoff of 150°.

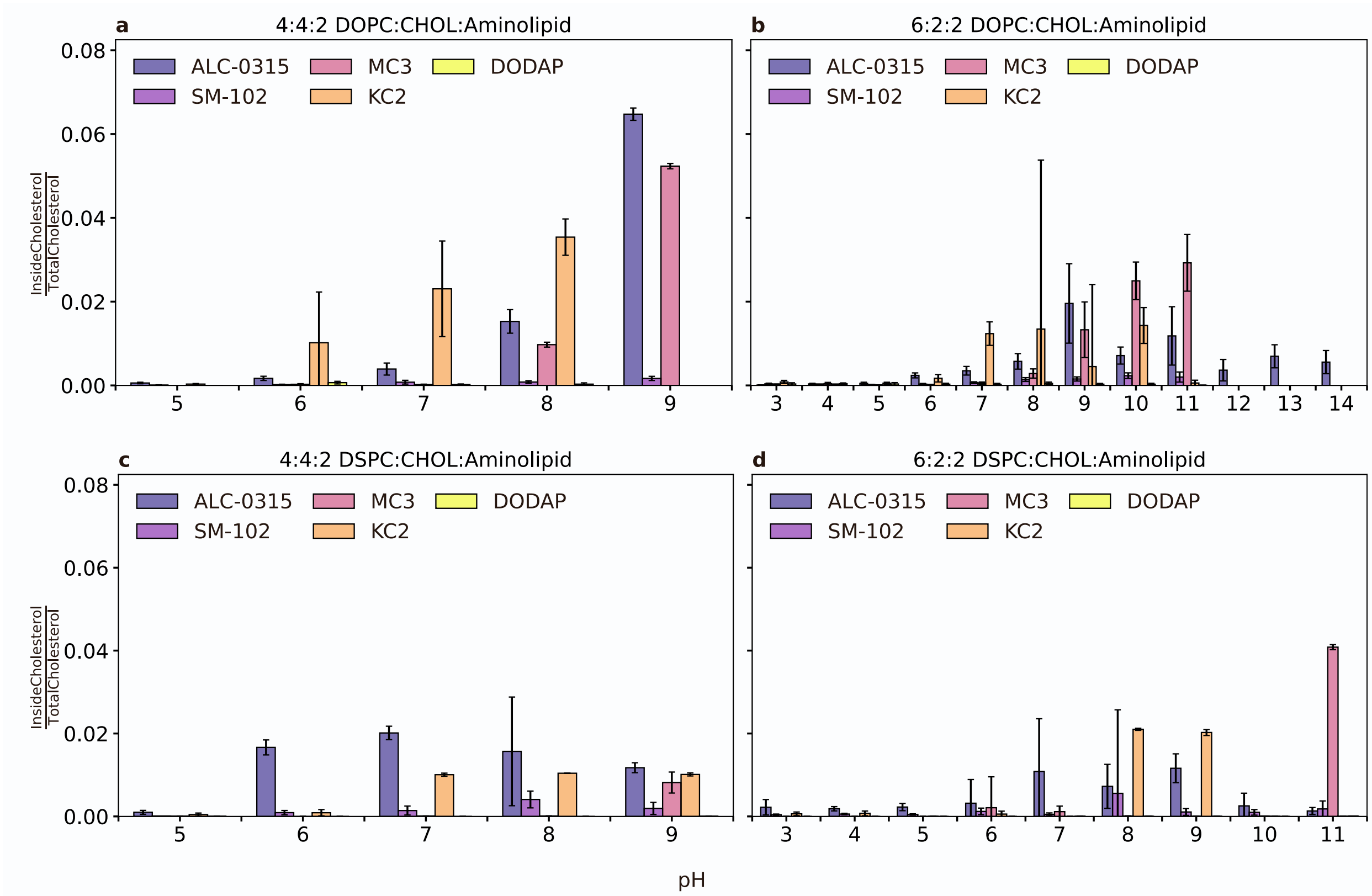

Figure S68: Fraction of cholesterol located within the membrane core as a function of pH for DOPC-based (top) or DSPC-based (bottom) membranes containing **a**,**c** 40 mol% cholesterol and **b**,**d** 20 mol% cholesterol. Cholesterol positions were determined from the z-coordinates of the cholesterol hydroxyl oxygen. Cholesterol molecules were assigned to the membrane core if the reference atom was located more than 1.8 nm from the median phosphorus position of the respective leaflet. Error bars represent the standard error of the mean obtained by block averaging. Abbreviations used: CHOL = cholesterol.

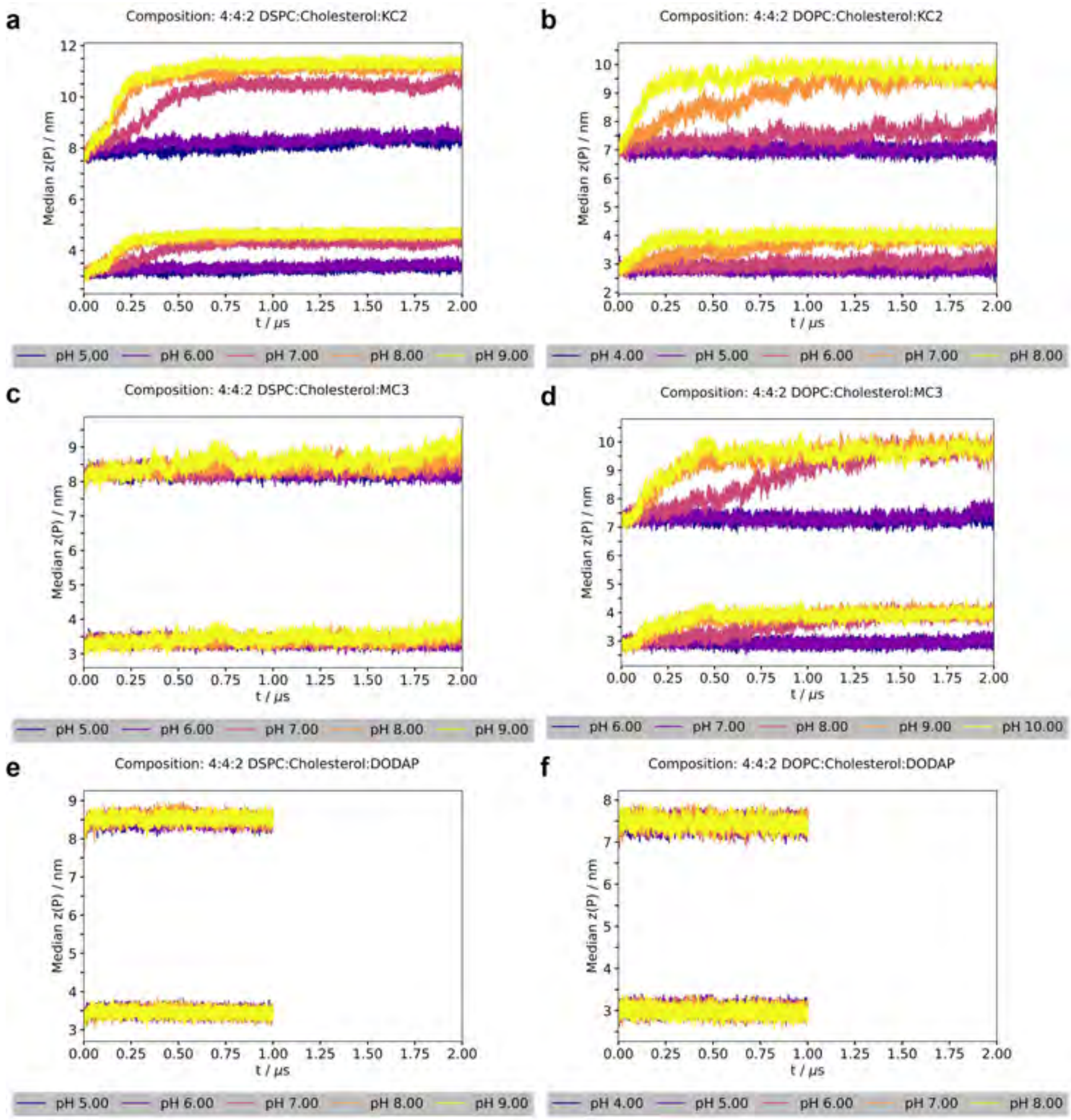

Figure S69: Average z-position of the phosphorus atoms in the upper and lower leaflets per frame for systems with a 4:4:2 helper lipid:cholesterol:aminolipid composition containing either **a,c,e** DSPC or **b,d,f** DOPC as the helper lipid, and **a,b** DLin-KC2-DMA, **c,d** DLin-MC3-DMA or **e,f** DODAP as the aminolipid. Average z-positions were computed using a periodic trigonometric approach (see Methods) to mitigate artifacts arising when the membrane is split across periodic boundary conditions.

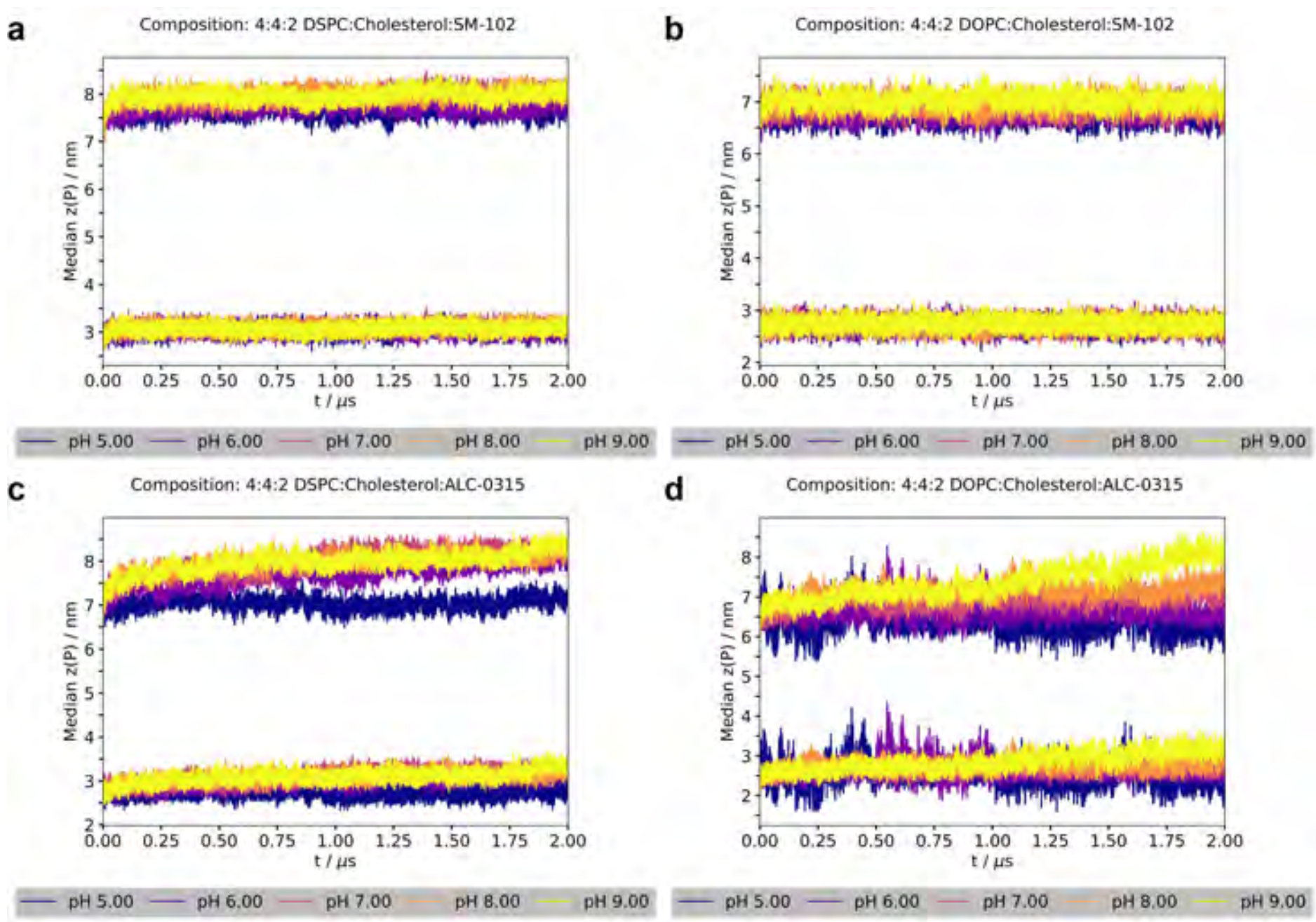

Figure S70: Average z-position of the phosphorus atoms in the upper and lower leaflets per frame for systems with a 4:4:2 helper lipid:cholesterol:aminolipid composition containing either **a,c** DSPC or **b,d** DOPC as the helper lipid, and **a,b** SM-102, or **c,d** ALC-0315 as the aminolipid. Average z-positions were computed using a periodic trigonometric approach (see Methods) to mitigate artifacts arising when the membrane is split across periodic boundary conditions.

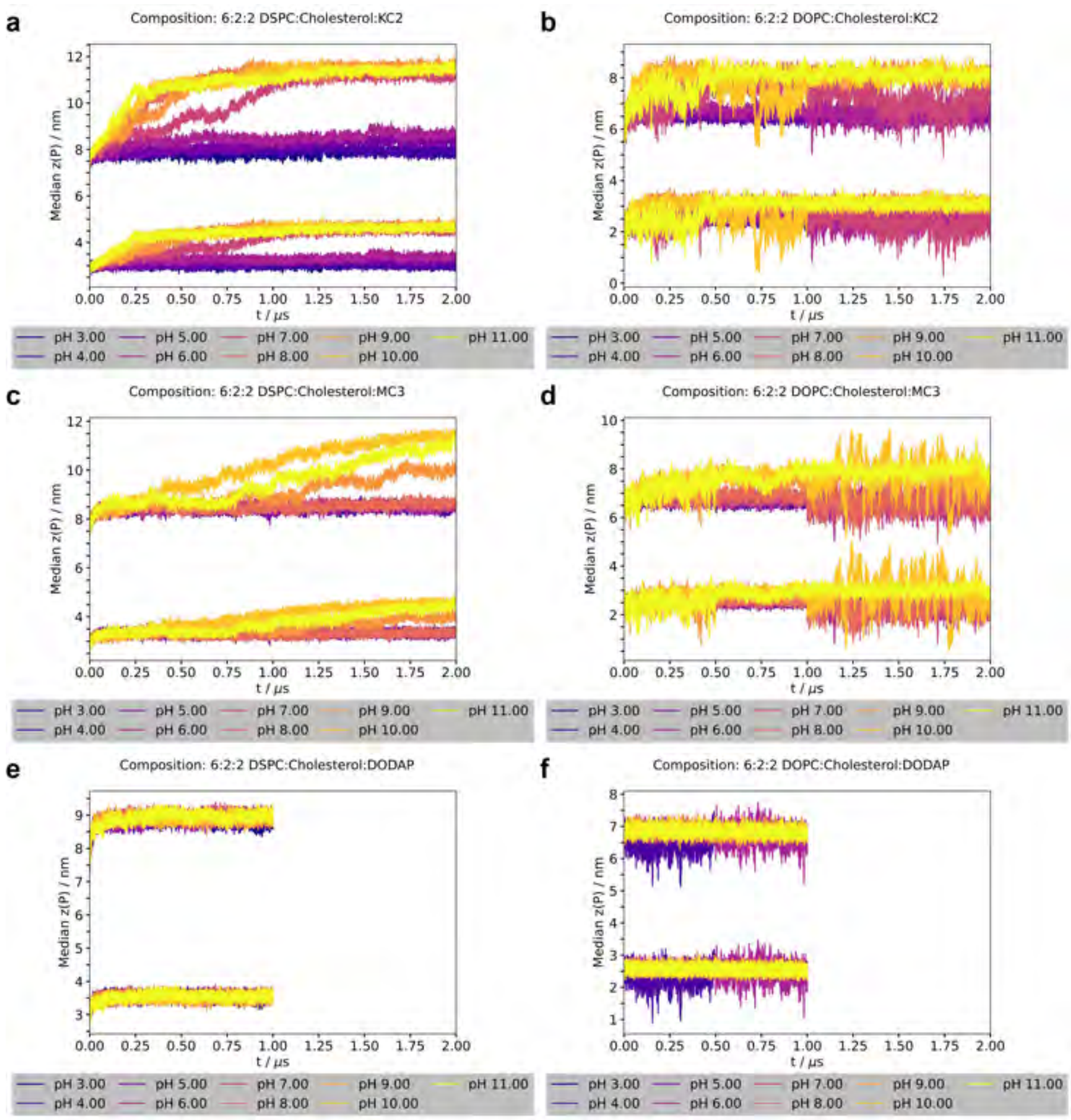

Figure S71: Average z-position of the phosphorus atoms in the upper and lower leaflets per frame for systems with a 6:2:2 helper lipid:cholesterol:aminolipid composition containing either **a,c,e** DSPC or **b,d,f** DOPC as the helper lipid, and **a,b** DLin-KC2-DMA, **c,d** DLin-MC3-DMA or **e,f** DODAP as the aminolipid. Average z-positions were computed using a periodic trigonometric approach (see Methods) to mitigate artifacts arising when the membrane is split across periodic boundary conditions.

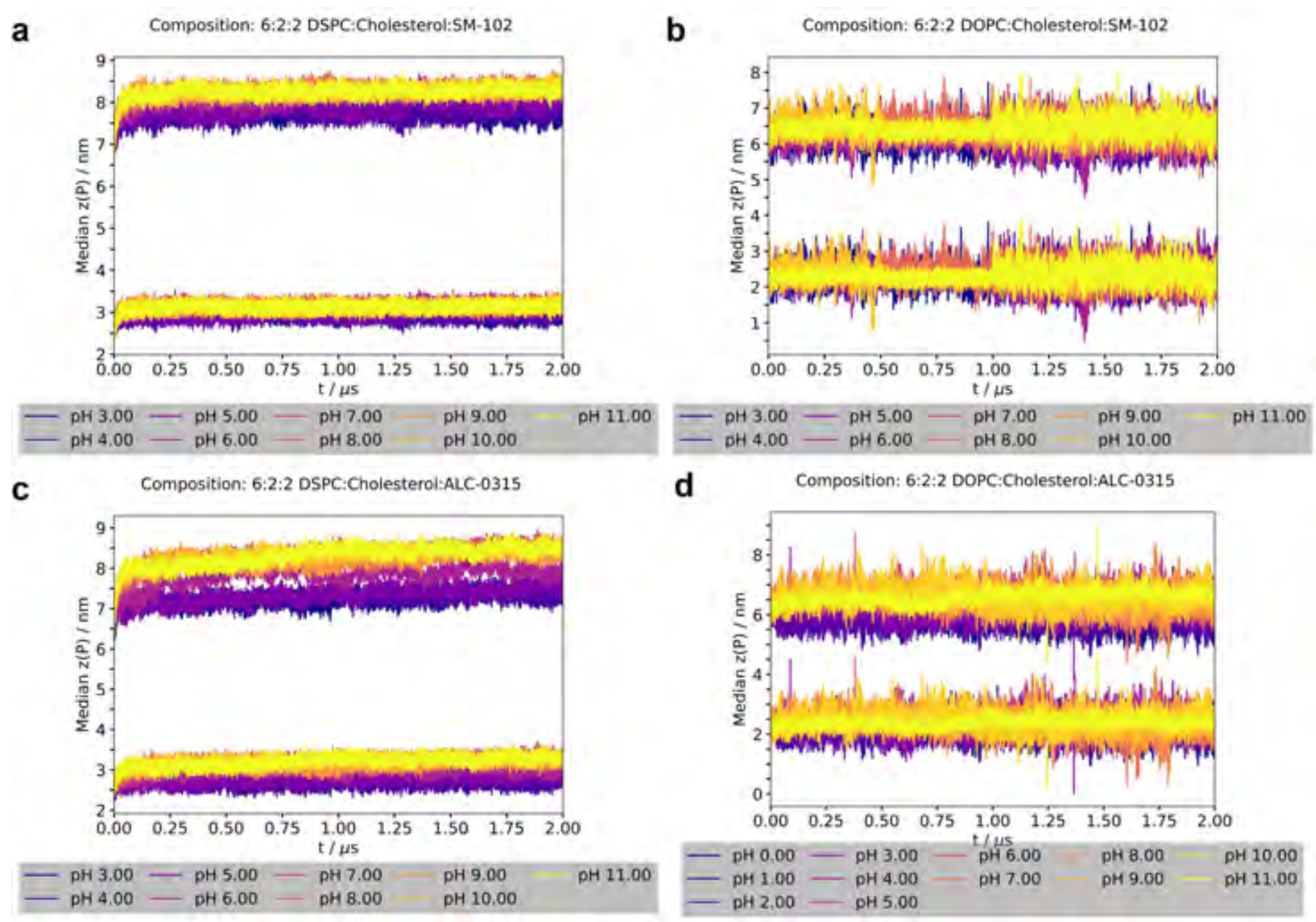

Figure S72: Average z-position of the phosphorus atoms in the upper and lower leaflets per frame for systems with a 6:2:2 helper lipid:cholesterol:aminolipid composition containing either **a,c** DSPC or **b,d** DOPC as the helper lipid, and **a,b** SM-102, or **c,d** ALC-0315 as the aminolipid. Average z-positions were computed using a periodic trigonometric approach (see Methods) to mitigate artifacts arising when the membrane is split across periodic boundary conditions.

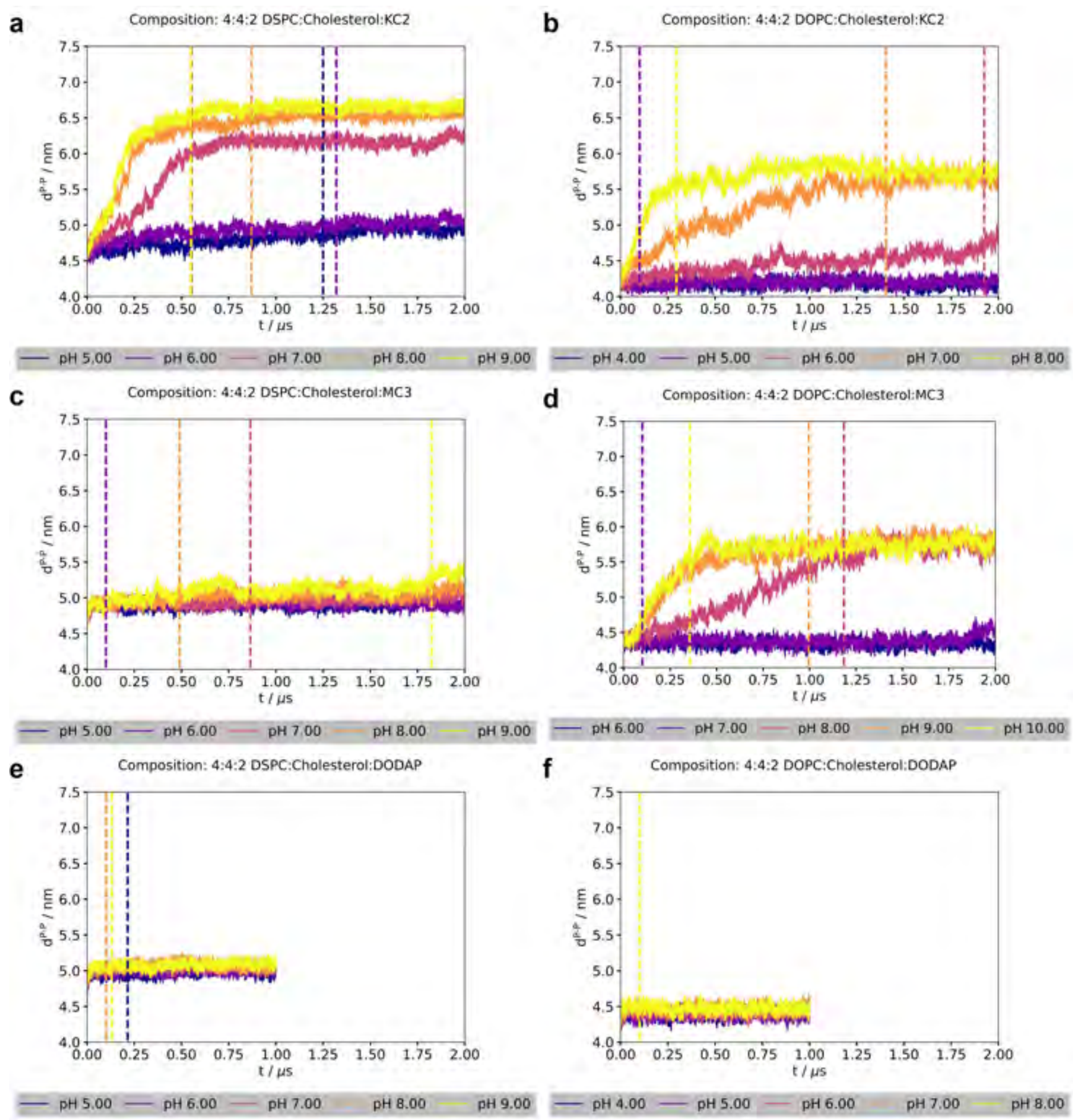

Figure S73: Membrane thickness per frame for systems with a 4:4:2 helper lipid:cholesterol:aminolipid composition containing either **a,c,e** DSPC or **b,d,f** DOPC as the helper lipid, and **a,b** DLin-KC2-DMA, **c,d** DLin-MC3-DMA or **e,f** DODAP as the aminolipid. Thickness is defined as the distance between the average z-positions of the phosphorus atoms in the upper and lower leaflets. Vertical dashed lines indicate the equilibration time determined for each pH value. Average z-positions were computed using a periodic trigonometric approach (see Methods) to mitigate artifacts arising when the membrane is split across periodic boundary conditions.

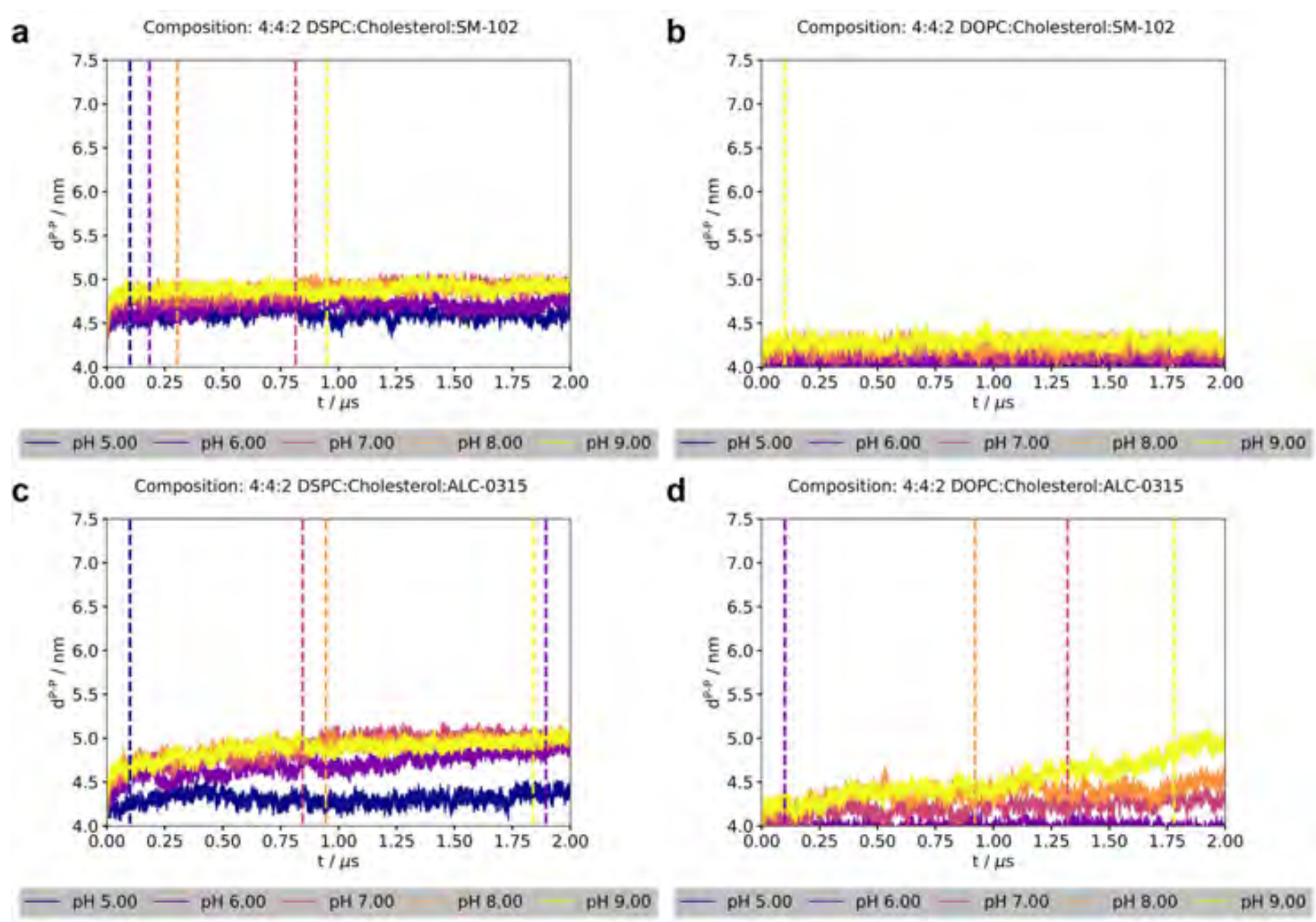

Figure S74: Membrane thickness per frame for systems with a 4:4:2 helper lipid:cholesterol:aminolipid composition containing either **a,c** DSPC or **b,d** DOPC as the helper lipid, and **a,b** SM-102, or **c,d** ALC-0315 as the aminolipid. Thickness is defined as the distance between the average z-positions of the phosphorus atoms in the upper and lower leaflets. Vertical dashed lines indicate the equilibration time determined for each pH value. Average z-positions were computed using a periodic trigonometric approach (see Methods) to mitigate artifacts arising when the membrane is split across periodic boundary conditions.

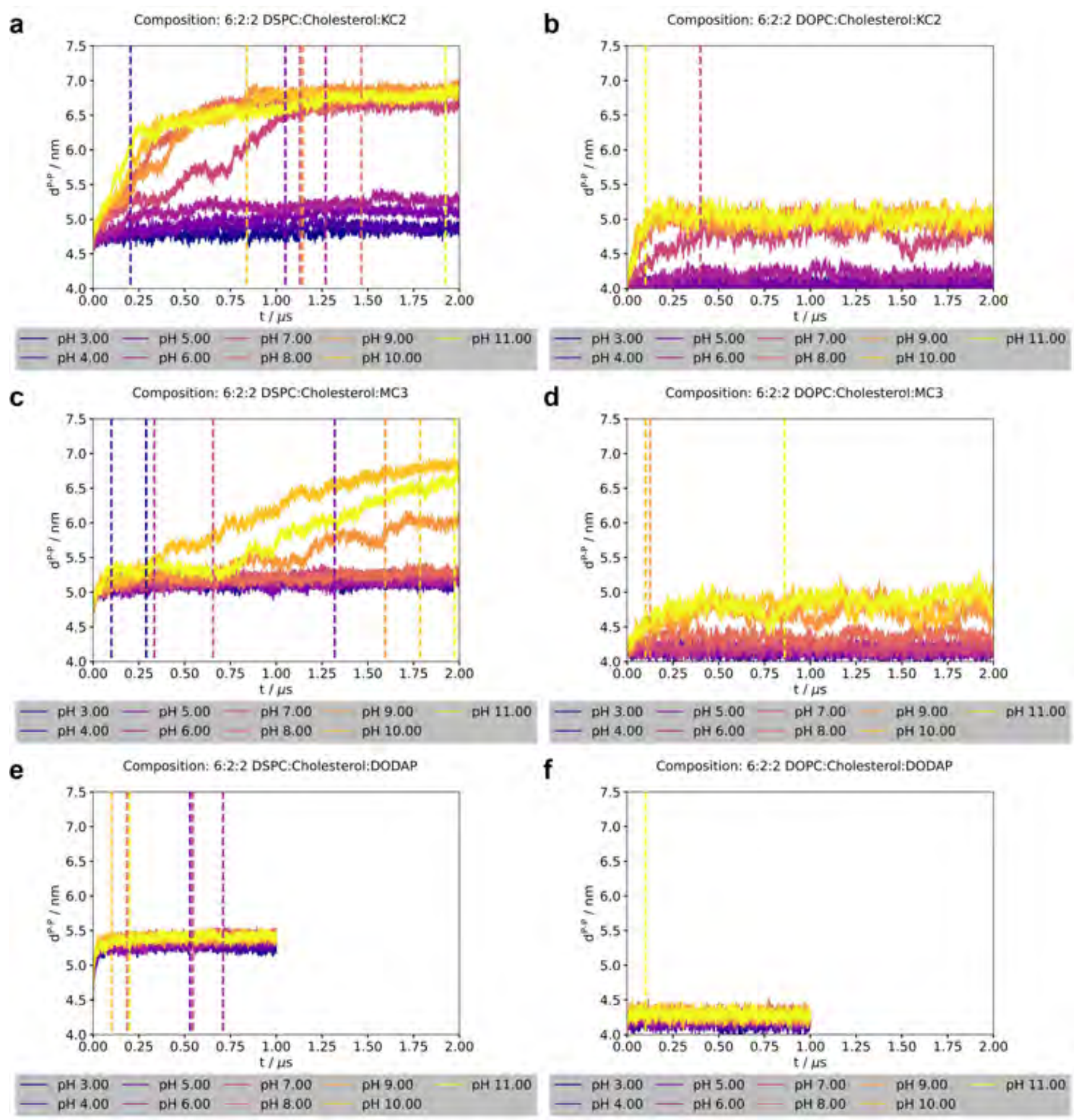

Figure S75: Membrane thickness per frame for systems with a 6:2:2 helper lipid:cholesterol:aminolipid composition containing either **a,c,e** DSPC or **b,d,f** DOPC as the helper lipid, and **a,b** DLin-KC2-DMA, **c,d** DLin-MC3-DMA or **e,f** DODAP as the aminolipid. Thickness is defined as the distance between the average z-positions of the phosphorus atoms in the upper and lower leaflets. Vertical dashed lines indicate the equilibration time determined for each pH value. Average z-positions were computed using a periodic trigonometric approach (see Methods) to mitigate artifacts arising when the membrane is split across periodic boundary conditions.

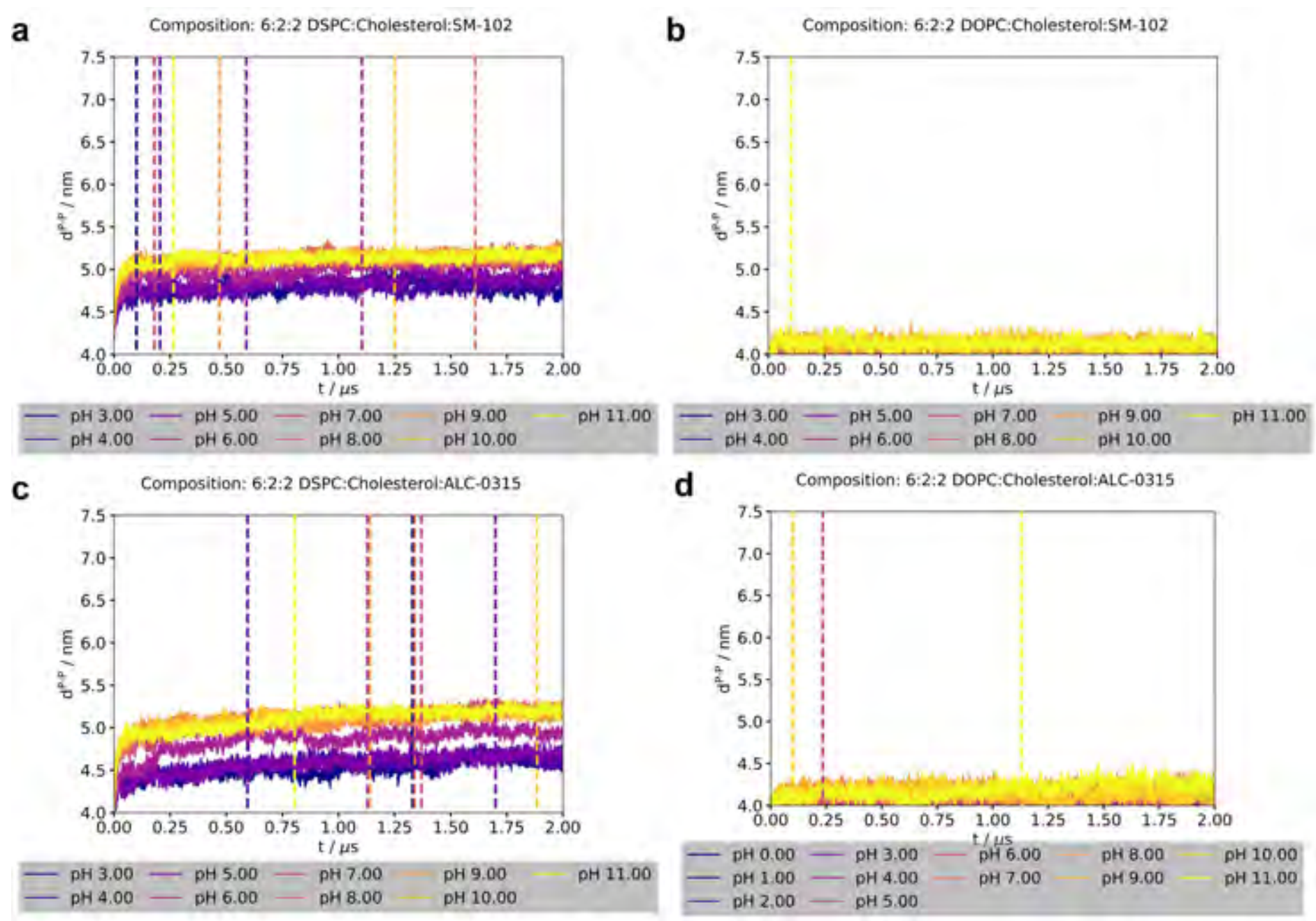

Figure S76: Membrane thickness per frame for systems with a 6:2:2 helper lipid:cholesterol:aminolipid composition containing either **a,c** DSPC or **b,d** DOPC as the helper lipid, and **a,b** SM-102, or **c,d** ALC-0315 as the aminolipid. Thickness is defined as the distance between the average z-positions of the phosphorus atoms in the upper and lower leaflets. Vertical dashed lines indicate the equilibration time determined for each pH value. Average z-positions were computed using a periodic trigonometric approach (see Methods) to mitigate artifacts arising when the membrane is split across periodic boundary conditions.

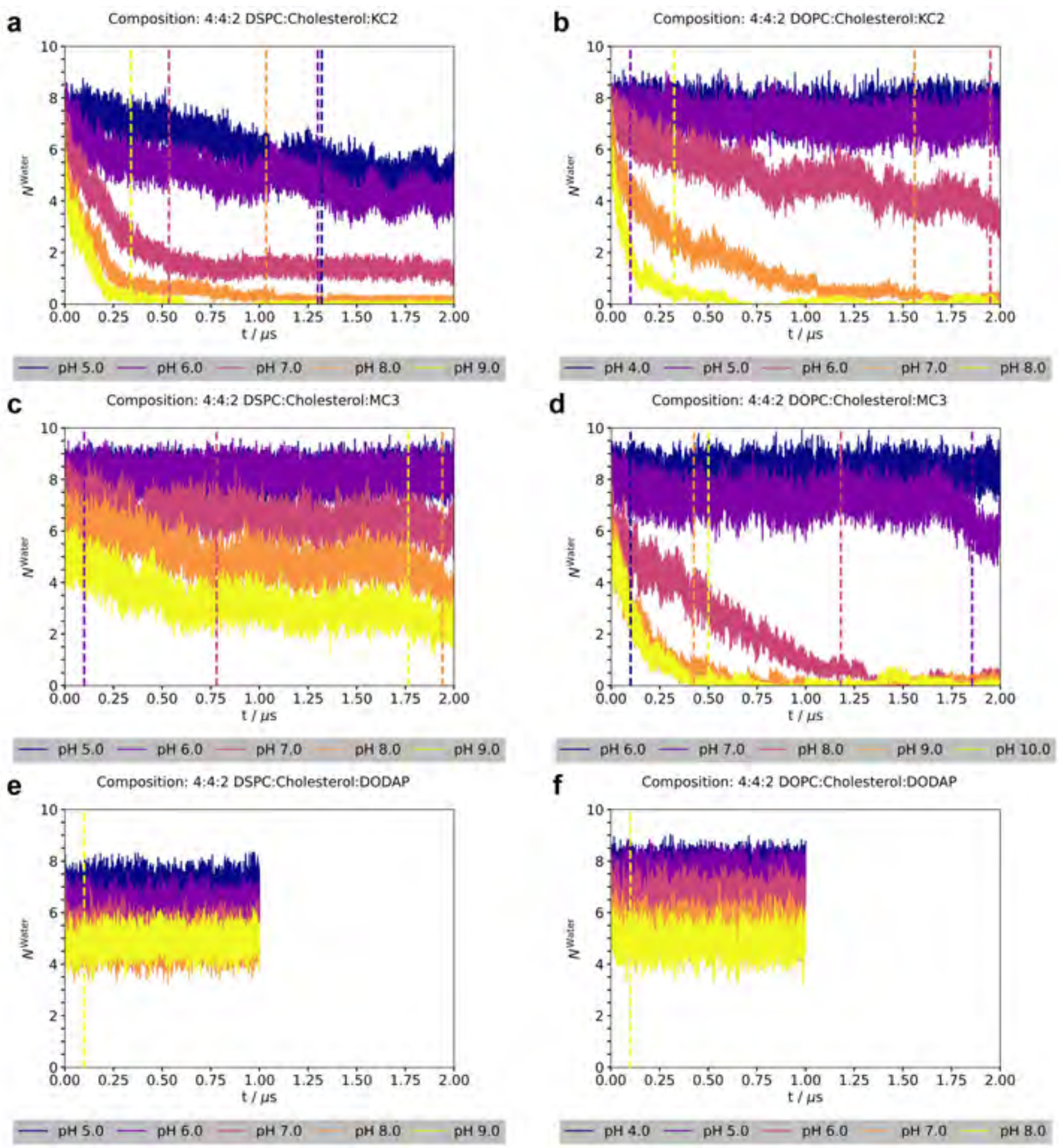

Figure S77: Average number of water molecules within 0.5 nm of the titratable headgroups (ρ_n^{Water}) per frame for membranes with a 4:4:2 helper lipid:cholesterol:aminolipid composition containing either **a,c,e** DSPC or **b,d,f** DOPC as the helper lipid, and **a,b** DLin-KC2-DMA, **c,d** DLin-MC3-DMA or **e,f** DODAP as the aminolipid. Vertical dashed lines indicate the equilibration time determined for each pH value. The reference atom of the aminolipid was the hydrogen atom of the titratable headgroup, whereas the oxygen atom was used as the reference for water.

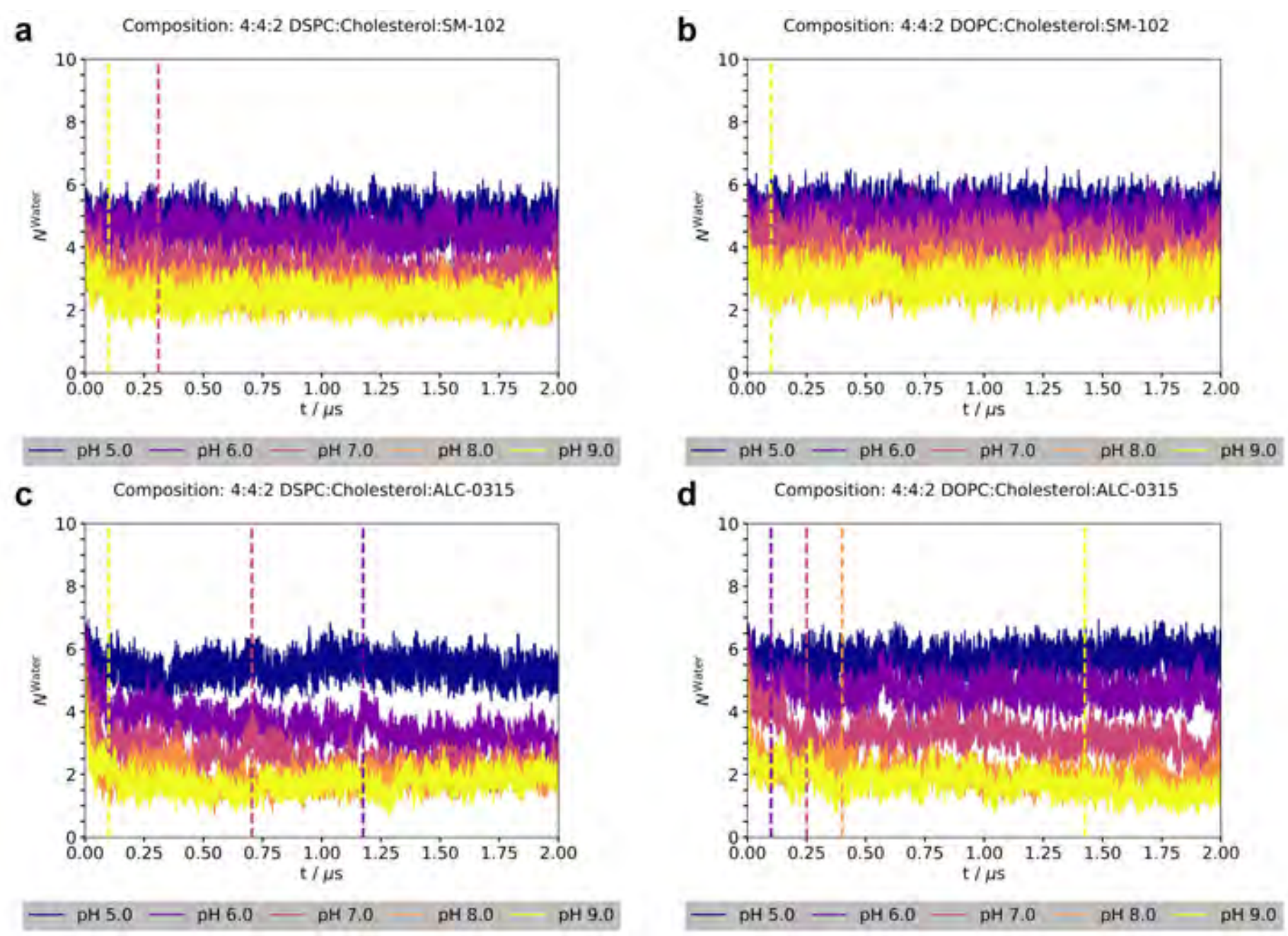

Figure S78: Average number of water molecules within 0.5 nm of the titratable headgroups (ρ_n^{Water}) per frame for membranes with a 4:4:2 helper lipid:cholesterol:aminolipid composition containing either **a,c** DSPC or **b,d** DOPC as the helper lipid, and **a,b** SM-102, or **c,d** ALC-0315 as the aminolipid. Vertical dashed lines indicate the equilibration time determined for each pH value. The reference atom of the aminolipid was the hydrogen atom of the titratable headgroup, whereas the oxygen atom was used as the reference for water.

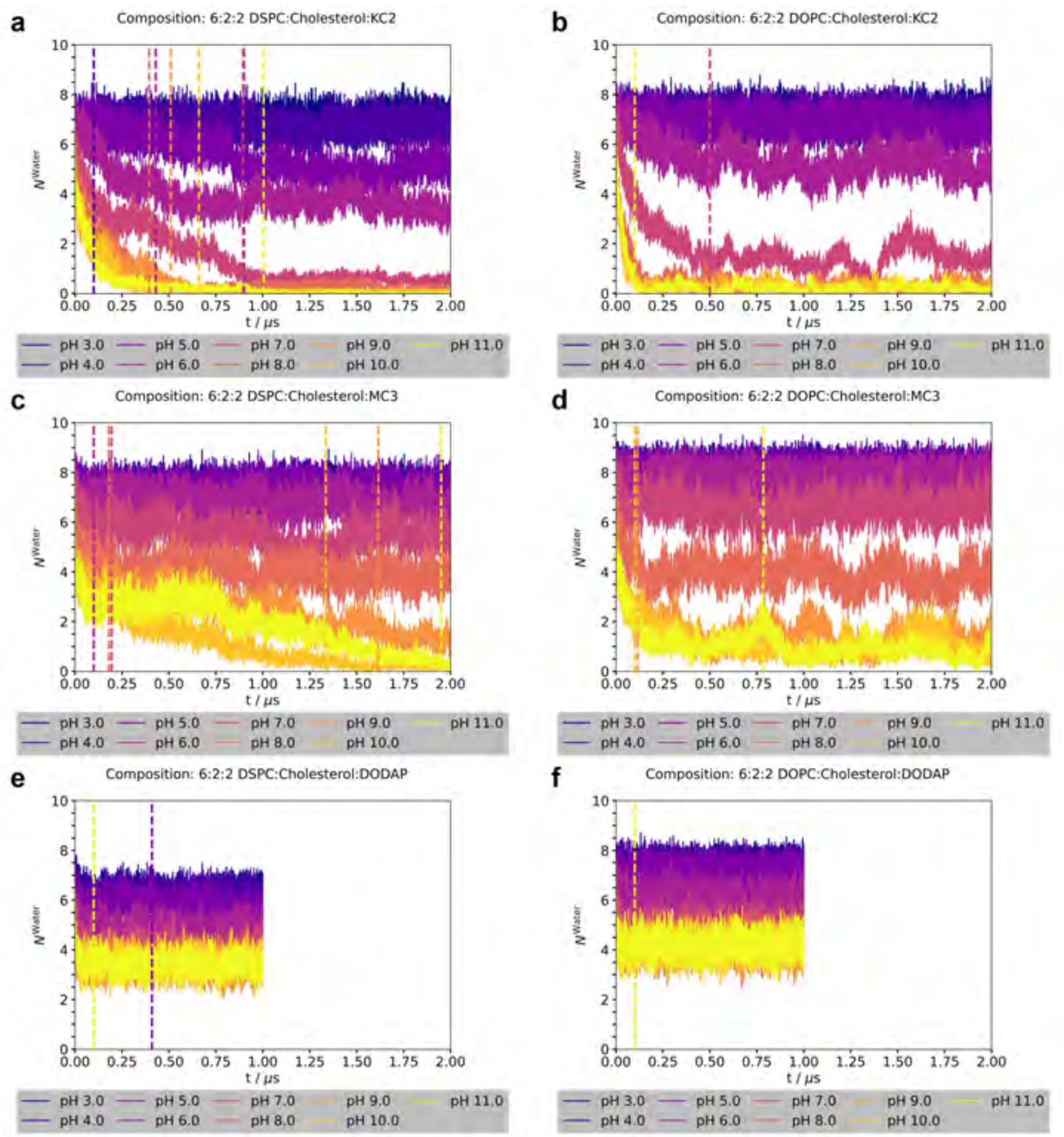

Figure S79: Average number of water molecules within 0.5 nm of the titratable headgroups (ρ_n^{Water}) per frame for membranes with a 6:2:2 helper lipid:cholesterol:aminolipid composition containing either **a,c,e** DSPC or **b,d,f** DOPC as the helper lipid, and **a,b** DLin-KC2-DMA, **c,d** DLin-MC3-DMA or **e,f** DODAP as the aminolipid. Vertical dashed lines indicate the equilibration time determined for each pH value. The reference atom of the aminolipid was the hydrogen atom of the titratable headgroup, whereas the oxygen atom was used as the reference for water.

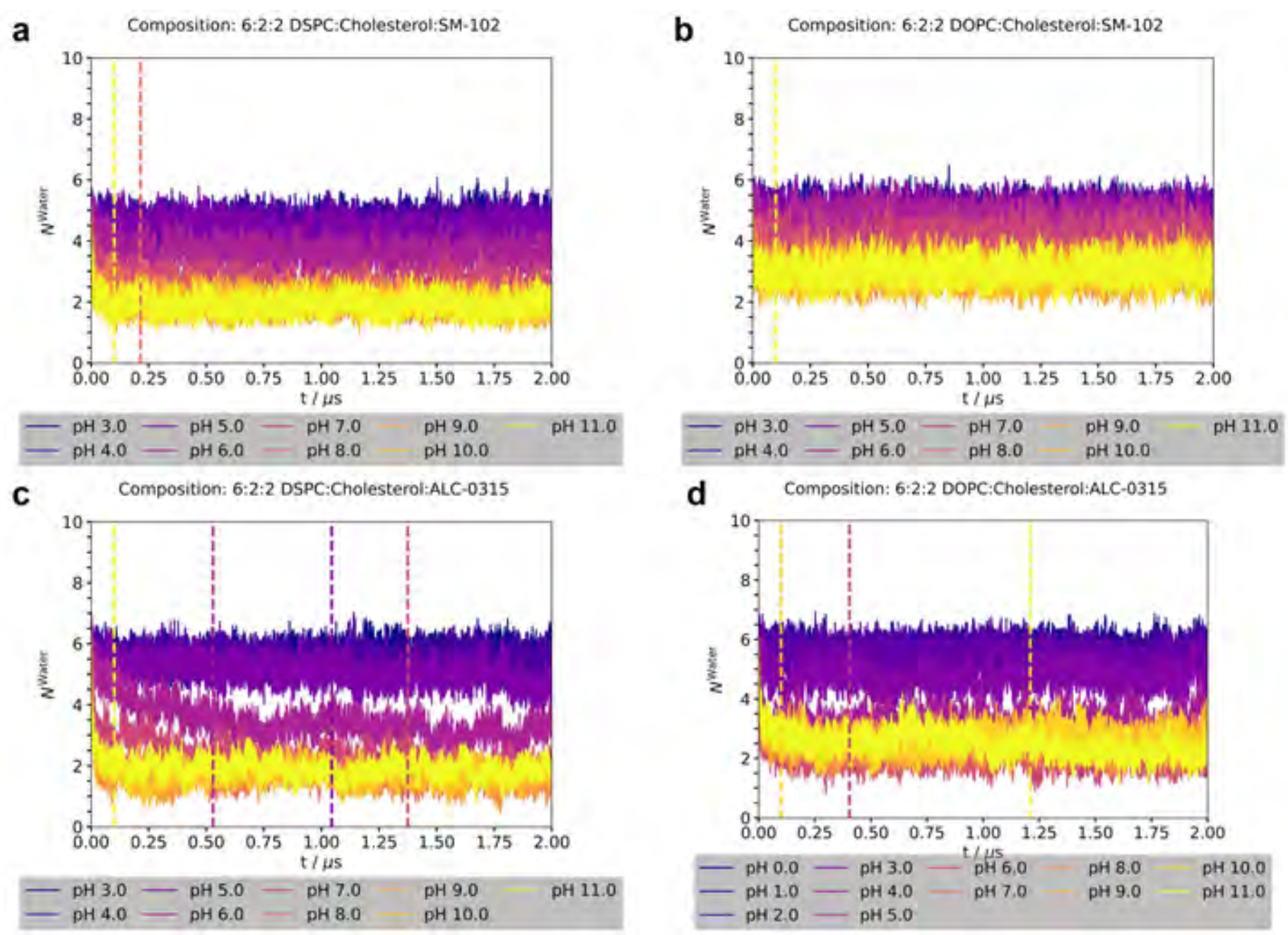

Figure S80: Average number of water molecules within 0.5 nm of the titratable headgroups (ρ_n^{Water}) per frame for membranes with a 6:2:2 helper lipid:cholesterol:aminolipid composition containing either **a,c** DSPC or **b,d** DOPC as the helper lipid, and **a,b** SM-102, or **c,d** ALC-0315 as the aminolipid. Vertical dashed lines indicate the equilibration time determined for each pH value. The reference atom of the aminolipid was the hydrogen atom of the titratable headgroup, whereas the oxygen atom was used as the reference for water.

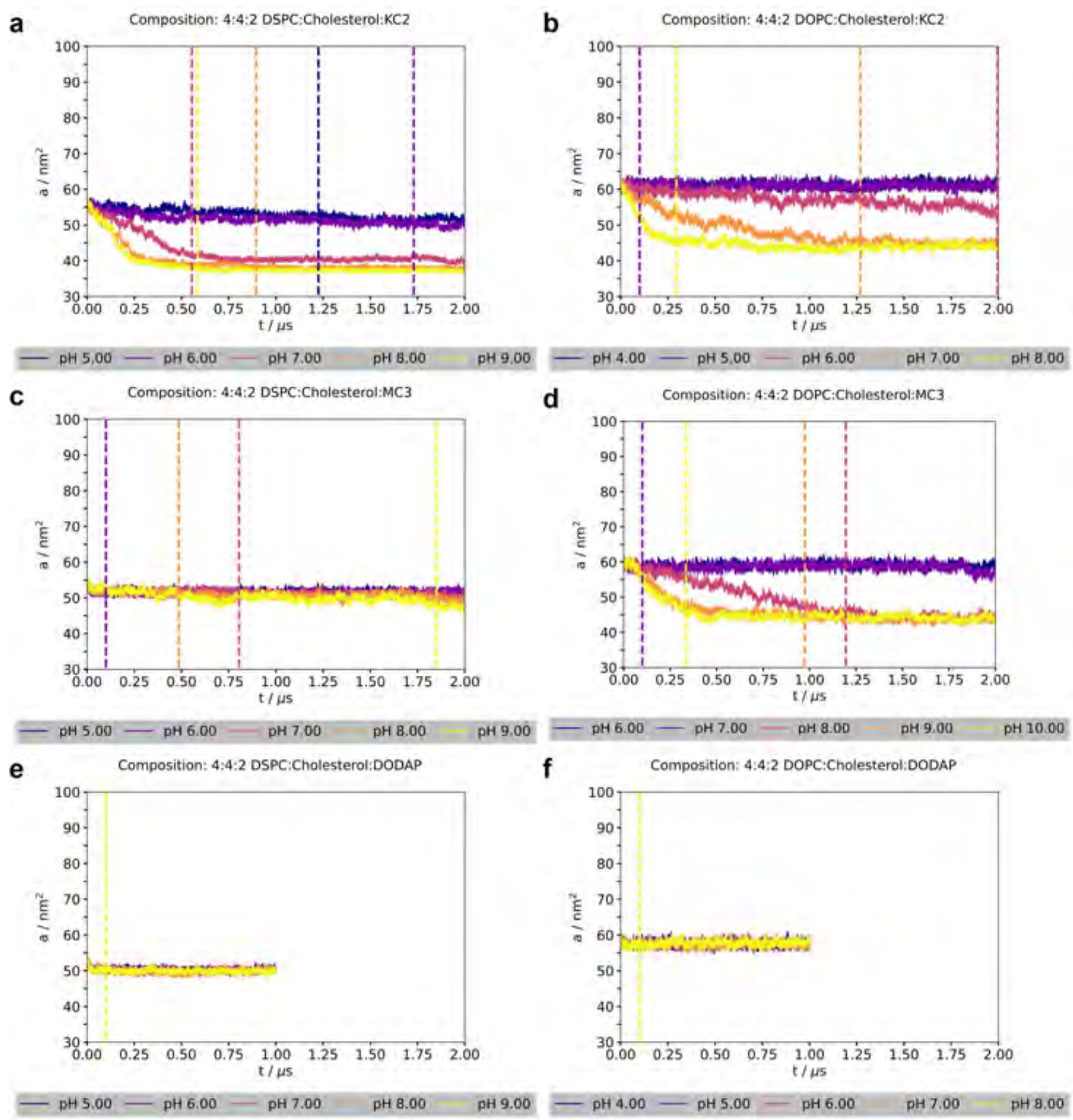

Figure S81: Leaflet surface area per frame for membranes with a 4:4:2 helper lipid:cholesterol:aminolipid composition containing either **a,c,e** DSPC or **b,d,f** DOPC as the helper lipid, and **a,b** DLin-KC2-DMA, **c,d** DLin-MC3-DMA or **e,f** DODAP as the aminolipid. Vertical dashed lines indicate the equilibration time determined for each pH value.

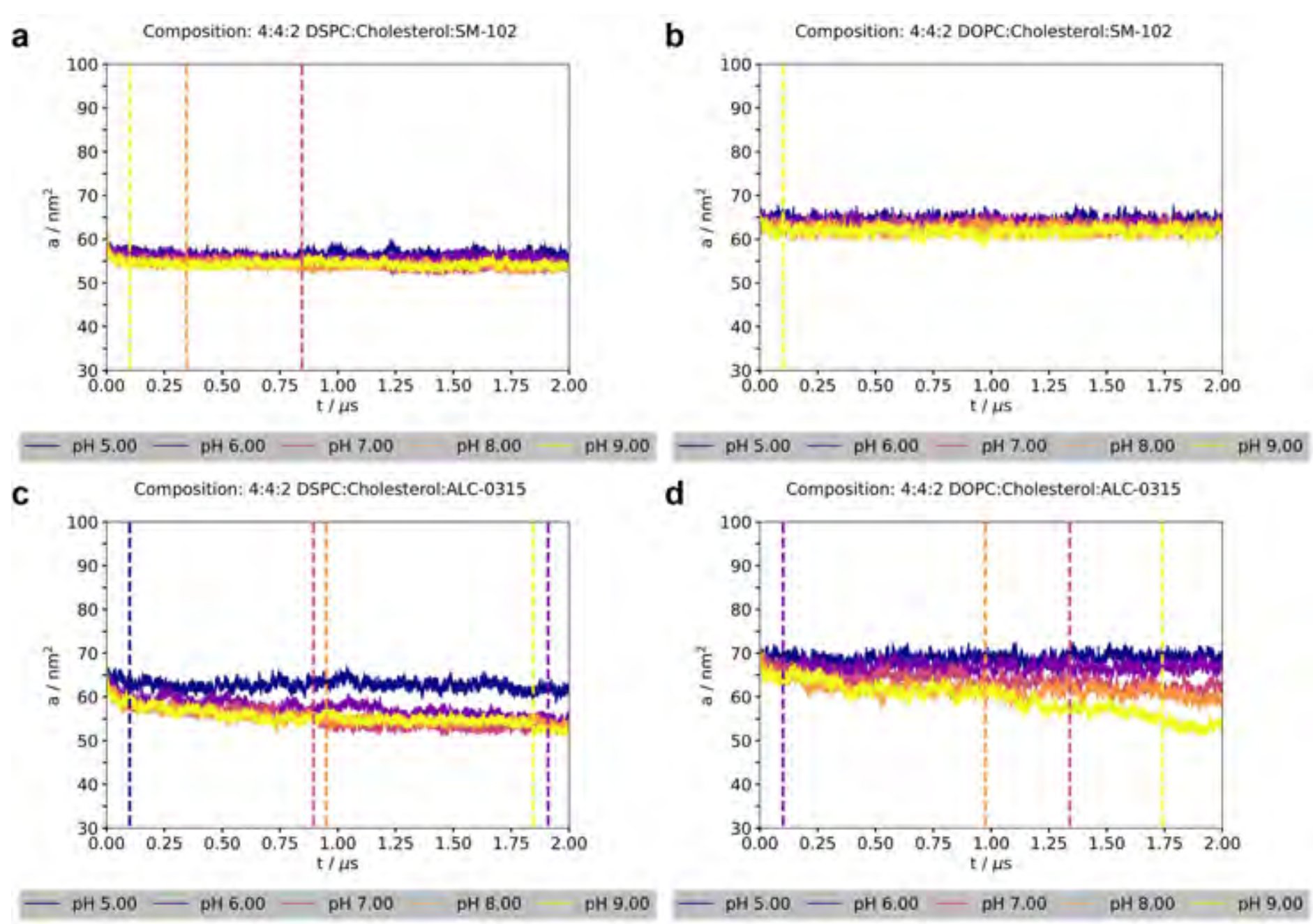

Figure S82: Leaflet surface area per frame for membranes with a 4:4:2 helper lipid:cholesterol:aminolipid composition containing either **a,c** DSPC or **b,d** DOPC as the helper lipid, and **a,b** SM-102, or **c,d** ALC-0315 as the aminolipid. Vertical dashed lines indicate the equilibration time determined for each pH value.

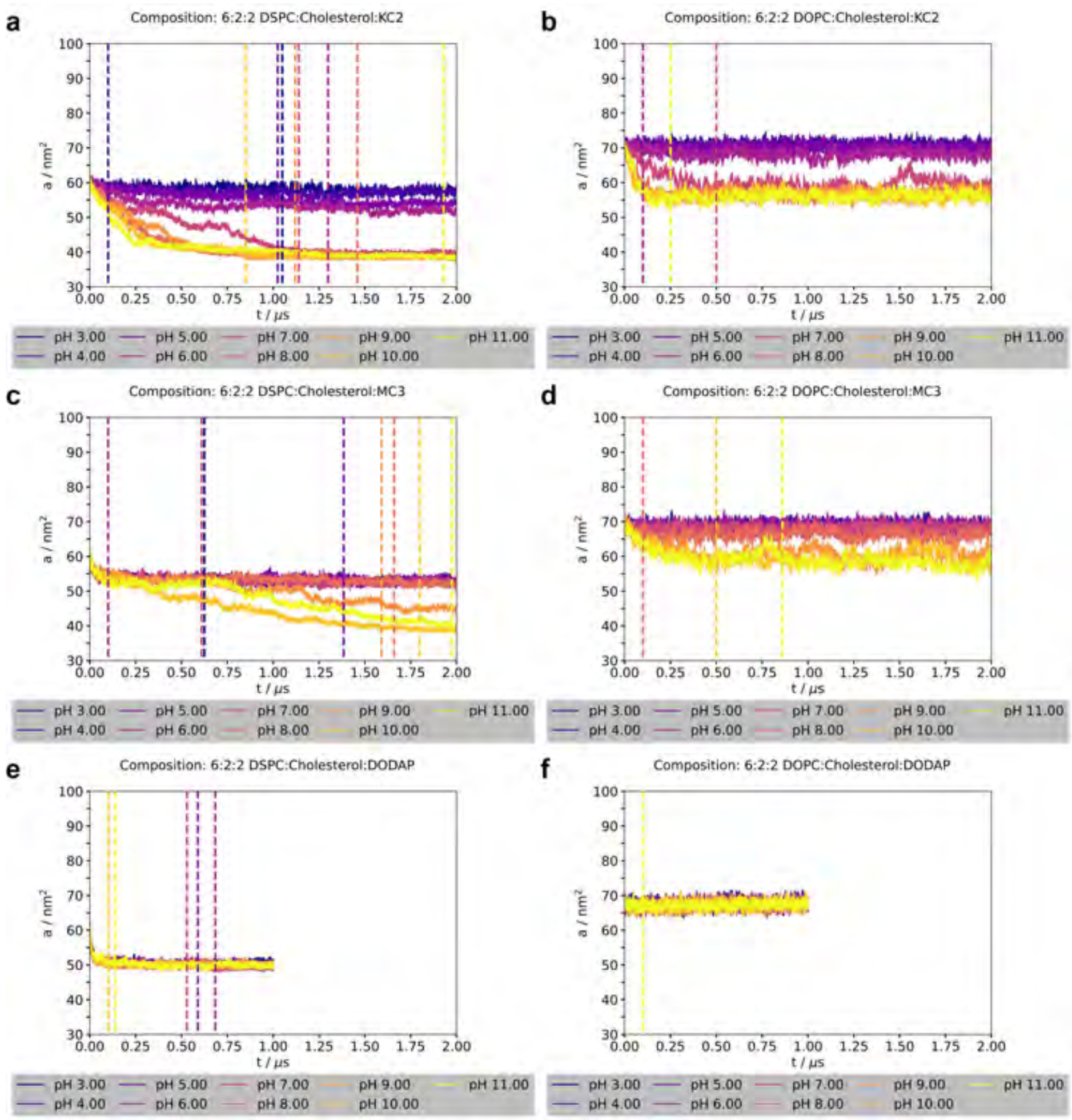

Figure S83: Leaflet surface area per frame for membranes with a 6:2:2 helper lipid:cholesterol:aminolipid composition containing either **a,c,e** DSPC or **b,d,f** DOPC as the helper lipid, and **a,b** DLin-KC2-DMA, **c,d** DLin-MC3-DMA or **e,f** DODAP as the aminolipid. Vertical dashed lines indicate the equilibration time determined for each pH value.

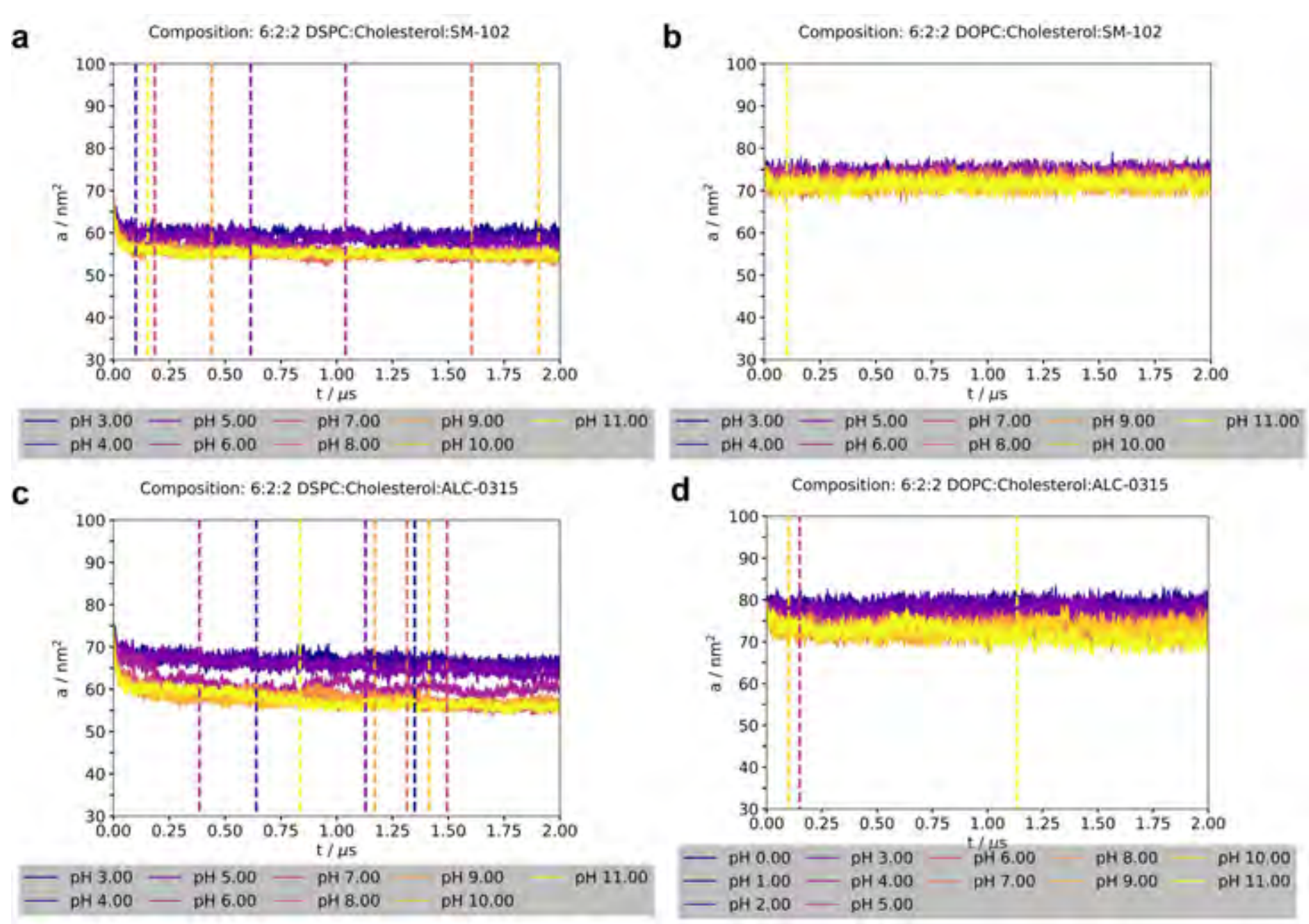

Figure S84: Leaflet surface area per frame for membranes with a 6:2:2 helper lipid:cholesterol:aminolipid composition containing either **a,c** DSPC or **b,d** DOPC as the helper lipid, and **a,b** SM-102, or **c,d** ALC-0315 as the aminolipid. Vertical dashed lines indicate the equilibration time determined for each pH value.

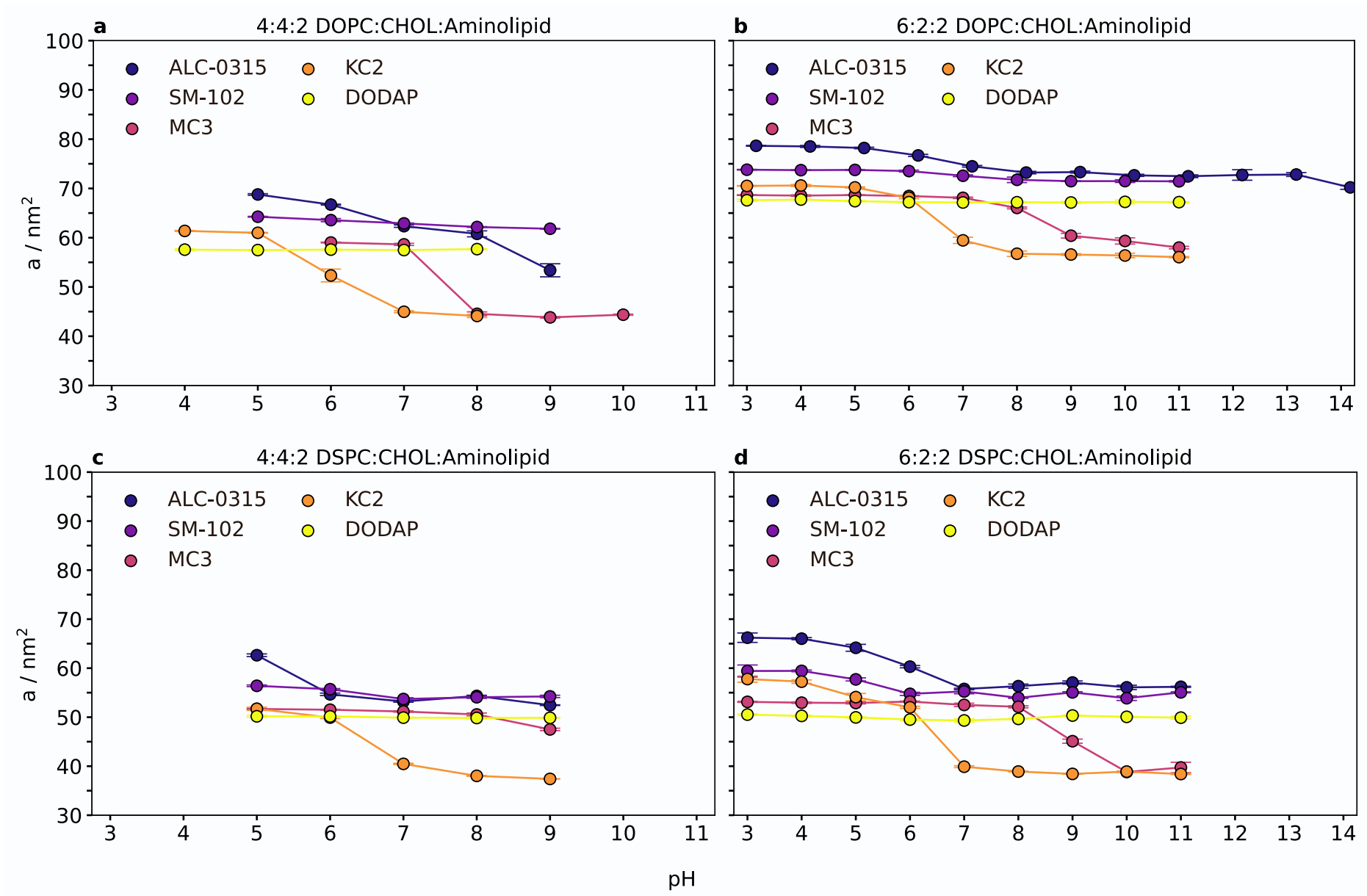

Figure S85: Average leaflet surface area as a function of pH. Mean values of the surface area are shown for DOPC-based (top) and DSPC-based (bottom) membranes containing **a,c** 40 mol% cholesterol and **b,d** 20 mol% cholesterol. Error bars represent the standard error of the mean obtained by block averaging. Abbreviations used: CHOL = cholesterol.

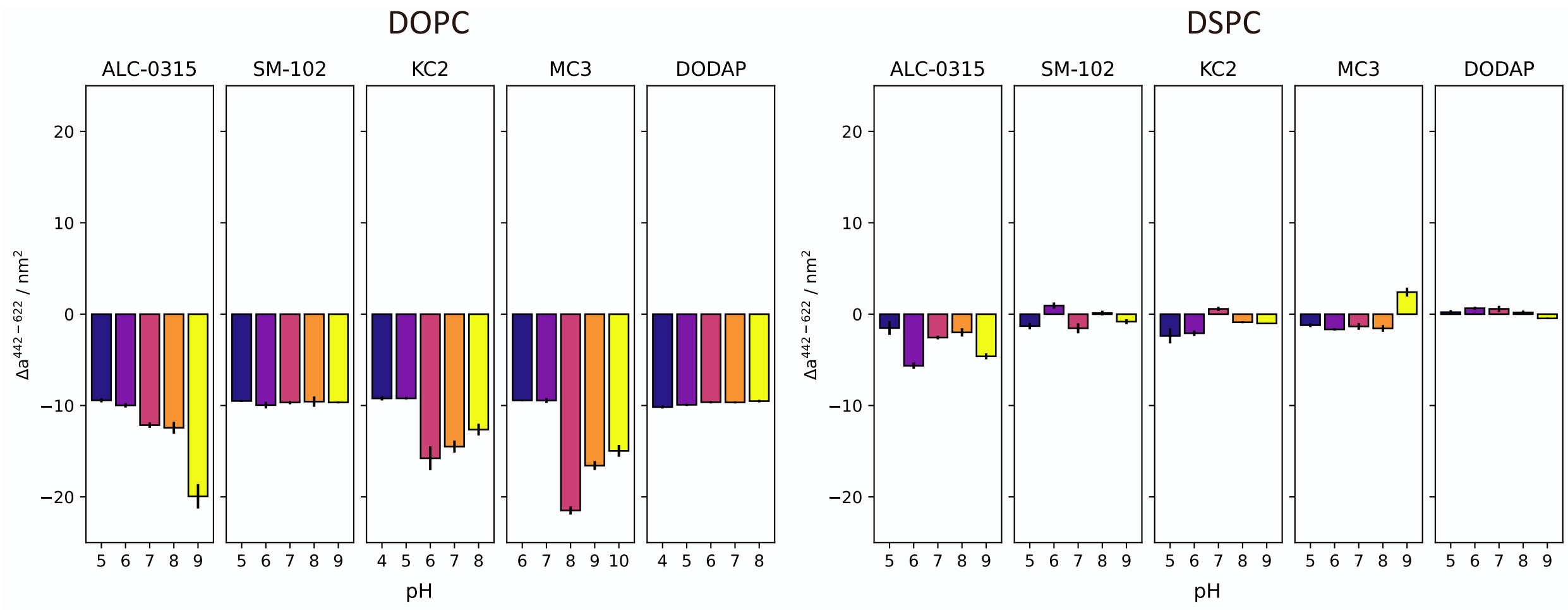

Figure S86: Difference in average leaflet surface area by increasing the cholesterol concentration from 20 mol% to 40 mol% ($\Delta a = a^{442} - a^{622}$) as a function of pH. Negative values refer to a smaller average membrane surface area per leaflet for 40 mol% cholesterol. Left and right panels correspond to DOPC and DSPC membranes, respectively. Error bars represent the standard error of the mean obtained by error propagation, assuming independent estimates.

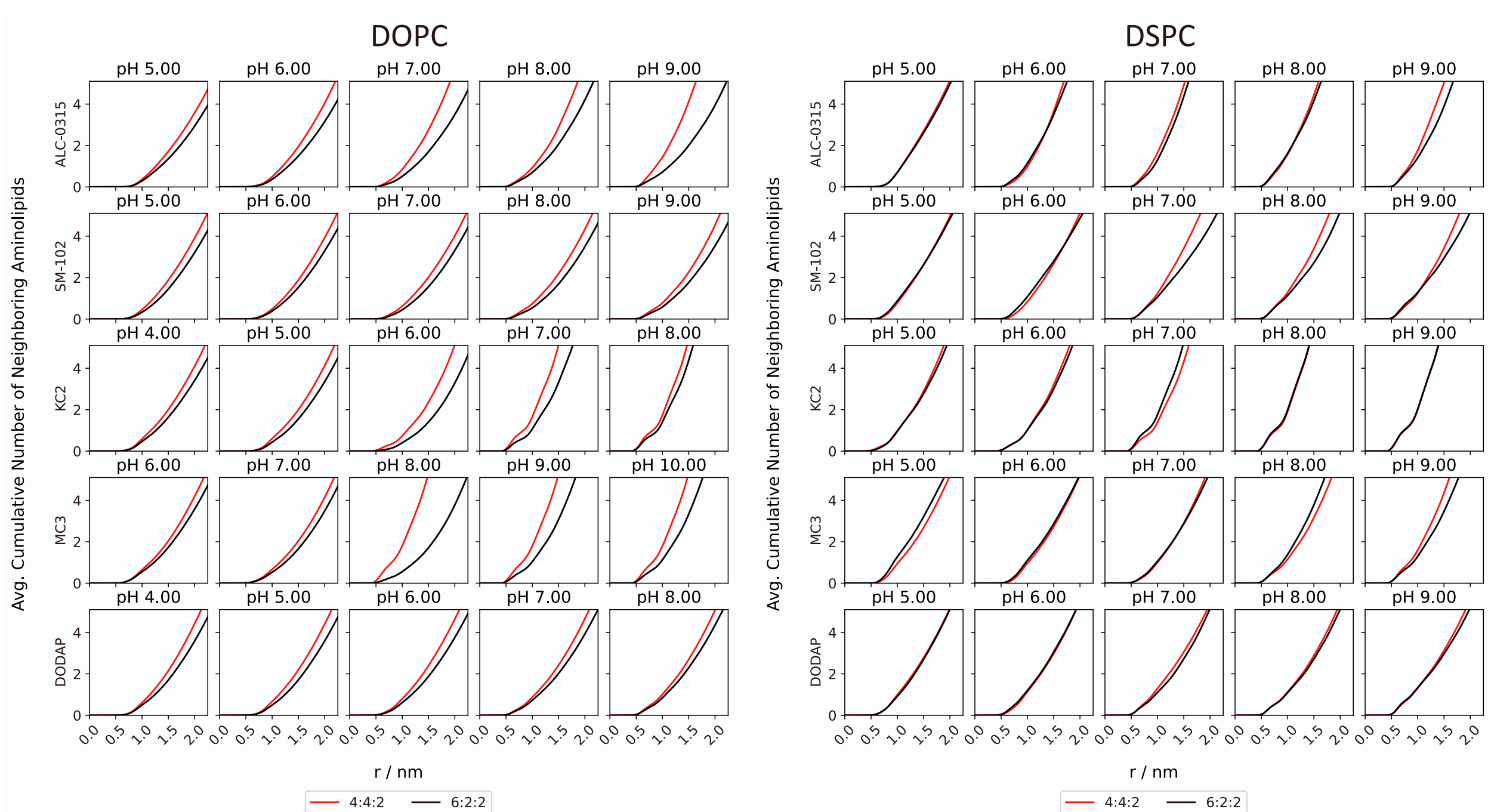

Figure S87: Average cumulative number of aminolipids surrounding other aminolipids as a function of radial distance. The titratable nitrogen atom of each aminolipid was used as the reference point for the distance calculation. Left and right panels correspond to DOPC and DSPC membranes, respectively. Lines correspond either to 20 mol% (black) or 40 mol% (red) cholesterol.

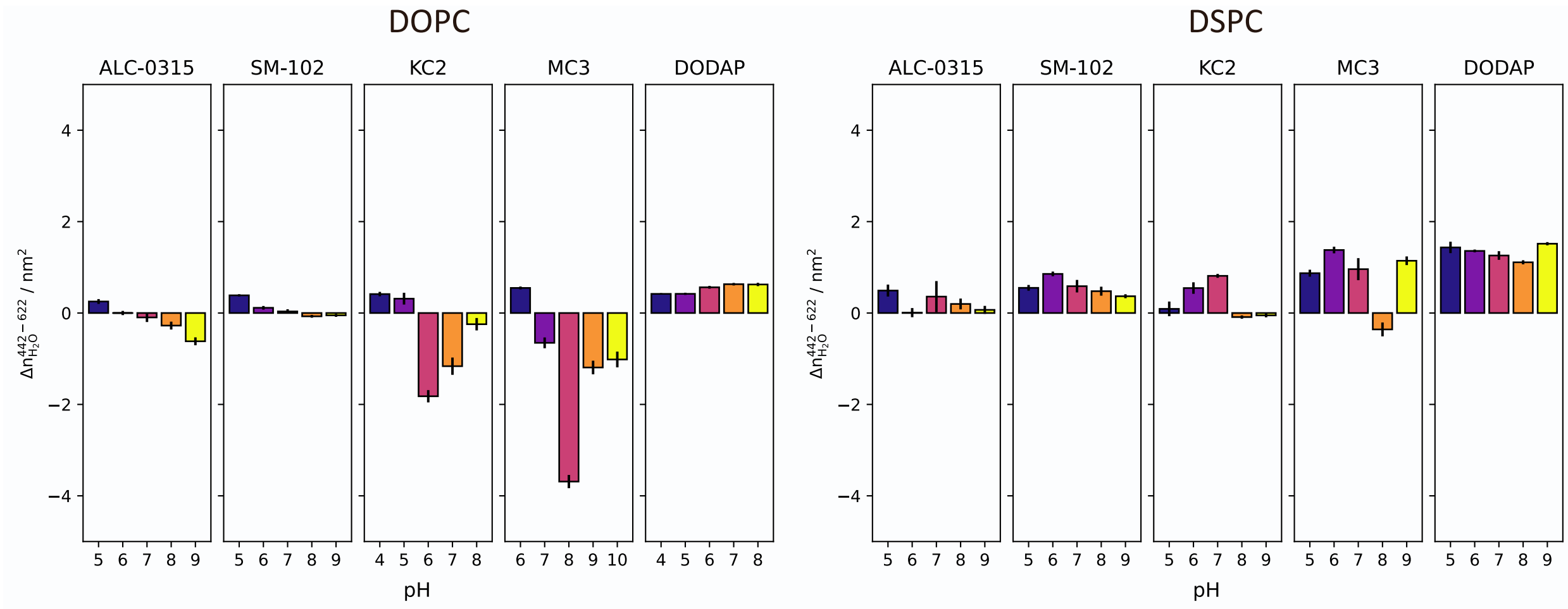

Figure S88: Difference in the average number of water molecules within 0.5 nm of the aminolipid titratable site by increasing the cholesterol concentration from 20 mol% to 40 mol% ($\Delta n_{\mathrm{H_2O}} = a^{442}_{\mathrm{H_2O}} - a^{622}_{\mathrm{H_2O}}$) as a function of pH. Negative values refer to a smaller average number of water molecules for 40 mol% cholesterol. Left and right panels correspond to DOPC and DSPC membranes, respectively. Error bars represent the standard error of the mean obtained by error propagation, assuming independent estimates.

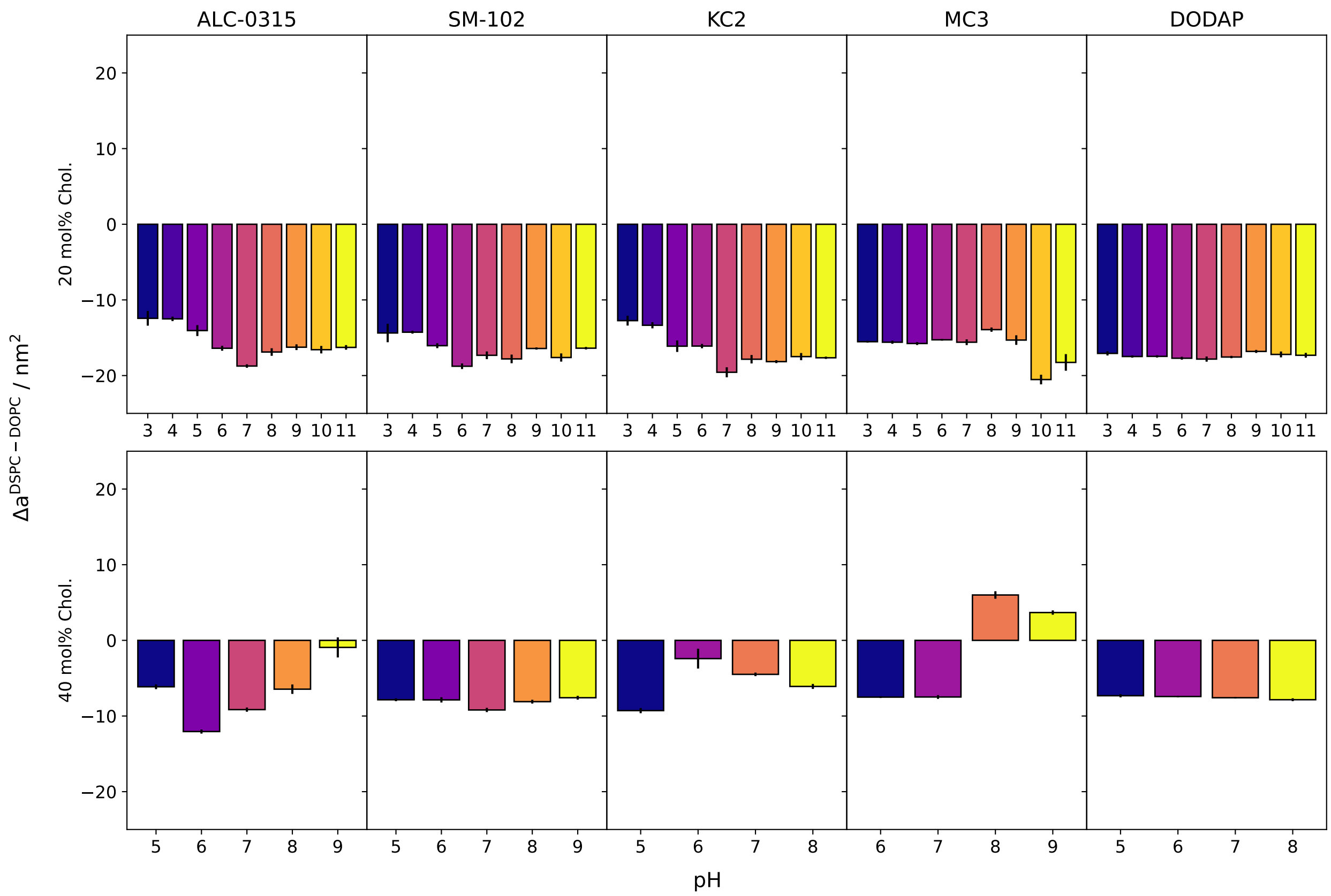

Figure S89: Difference in average leaflet surface area by exchanging DOPC with DSPC as phospholipid in the investigated membranes ($\Delta a = a^{DSPC} - a^{DOPC}$) as function of pH. Negative values refer to a smaller average membrane surface area per leaflet for compositions with DSPC. Upper and lower panel correspond to 20 mol% and 40 mol% cholesterol, respectively. Error bars represent the standard error of the mean obtained by error propagation, assuming independent estimates.

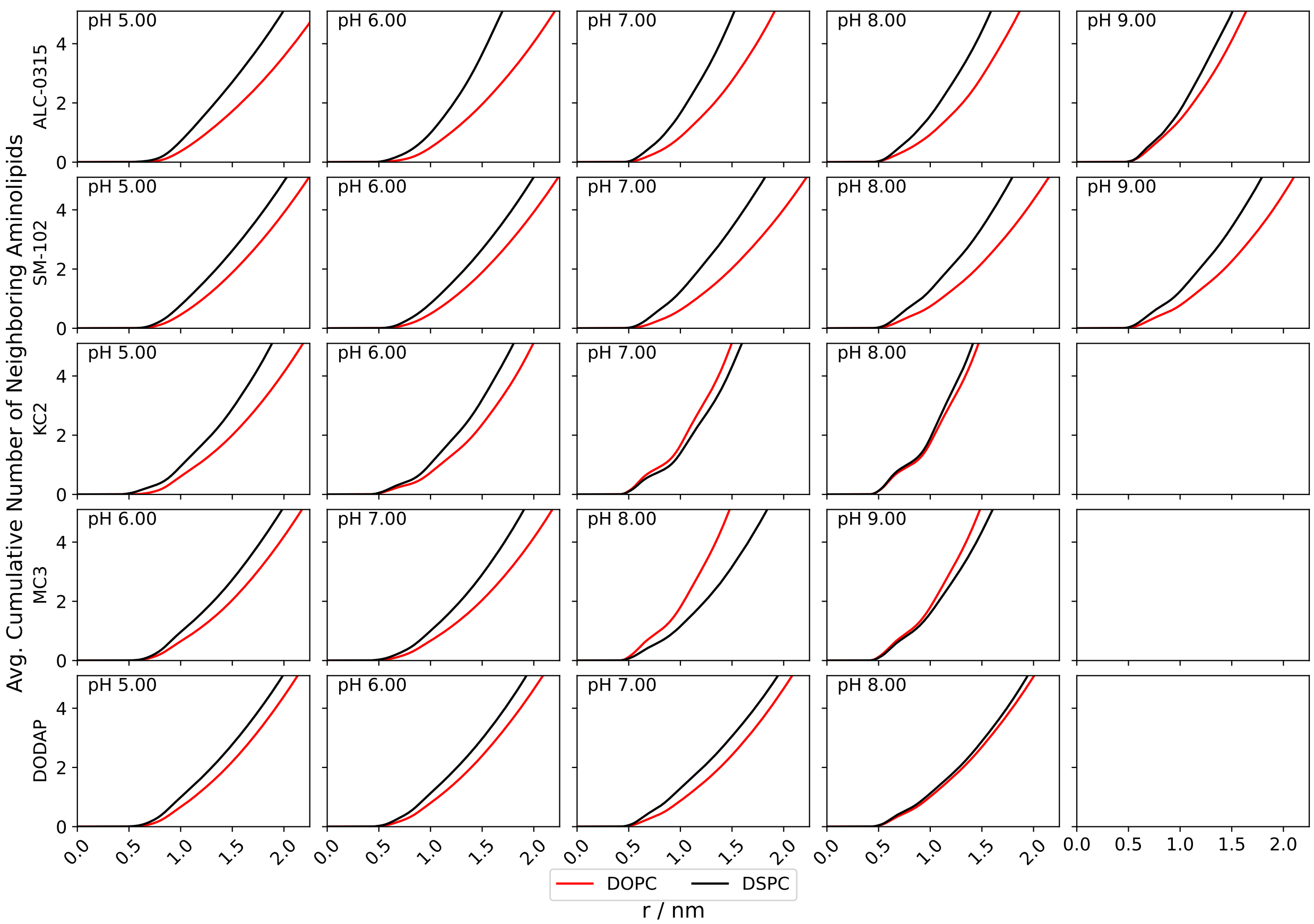

Figure S90: Average cumulative number of neighboring aminolipids as a function of radial distance at a cholesterol concentration of 40 mol%. The titratable nitrogen atom of each aminolipid was used as the reference point for the distance calculation.

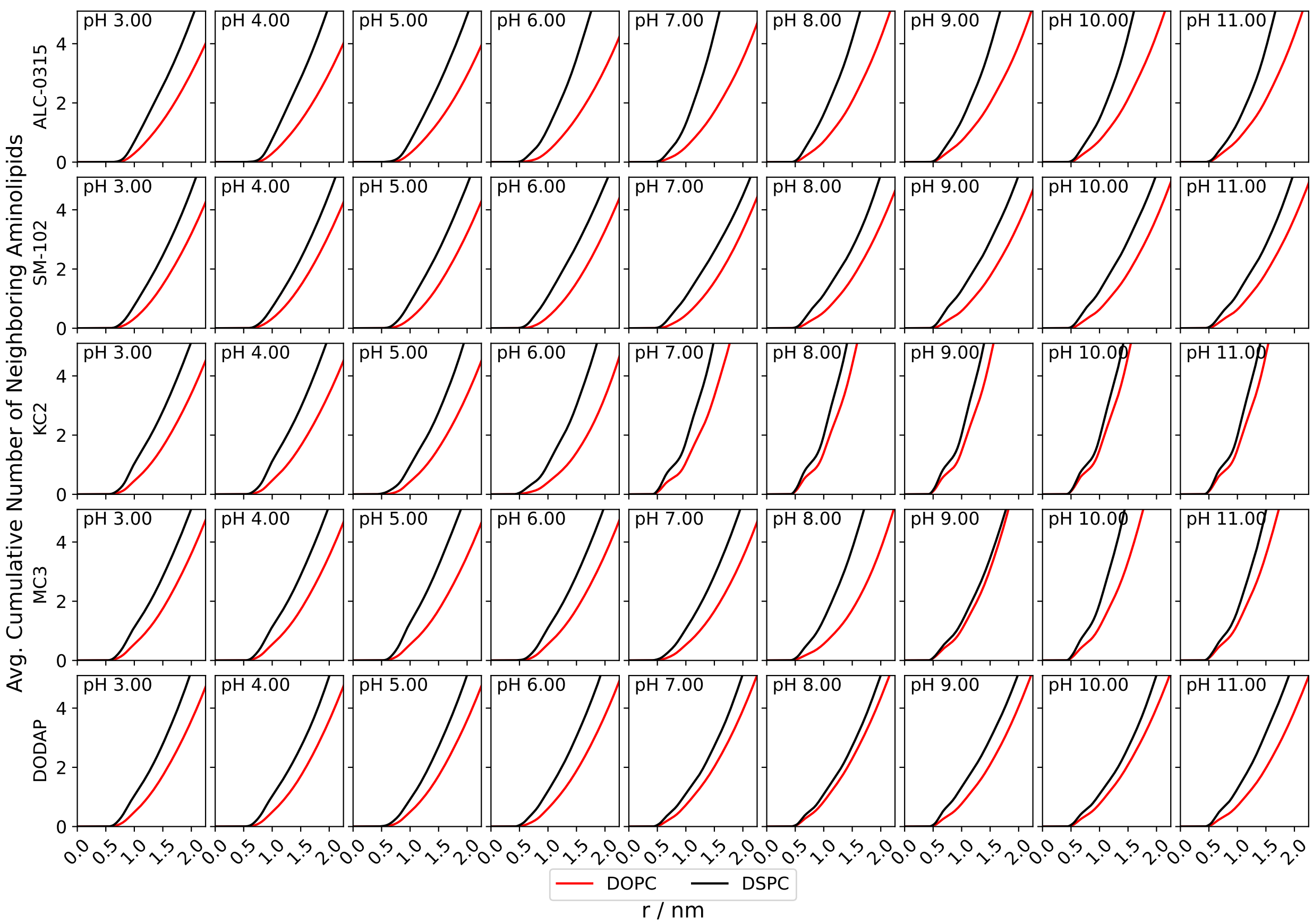

Figure S91: Average cumulative number of aminolipids surrounding other aminolipids as a function of radial distance at a cholesterol concentration of 20 mol%. The titratable nitrogen atom of each aminolipid was used as the reference point for the distance calculation.

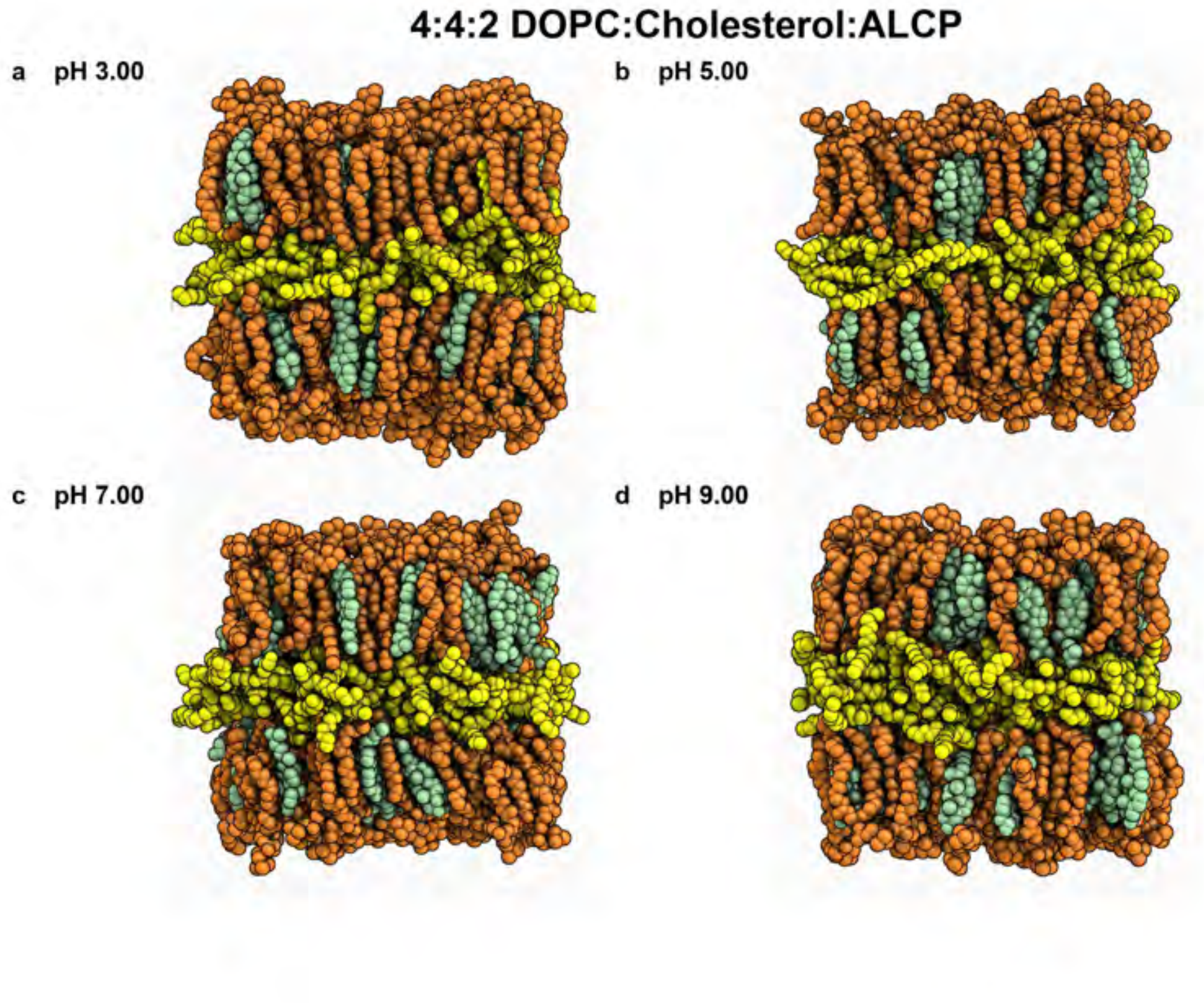

Figure S92: Side-view snapshots of the membrane in the final frame of the simulation at the corresponding pH values. Simulations were setup by placing deprotonated aminolipids in the membrane core. The helper lipid is shown in orange, cholesterol in green, the protonated amnolipid (net charge + 1) in blue, the deprotonated aminolipid (net charge 0) in yellow, and the aminolipids without a specific state ($0.2 \leq \lambda \leq 0.8$) in grey.

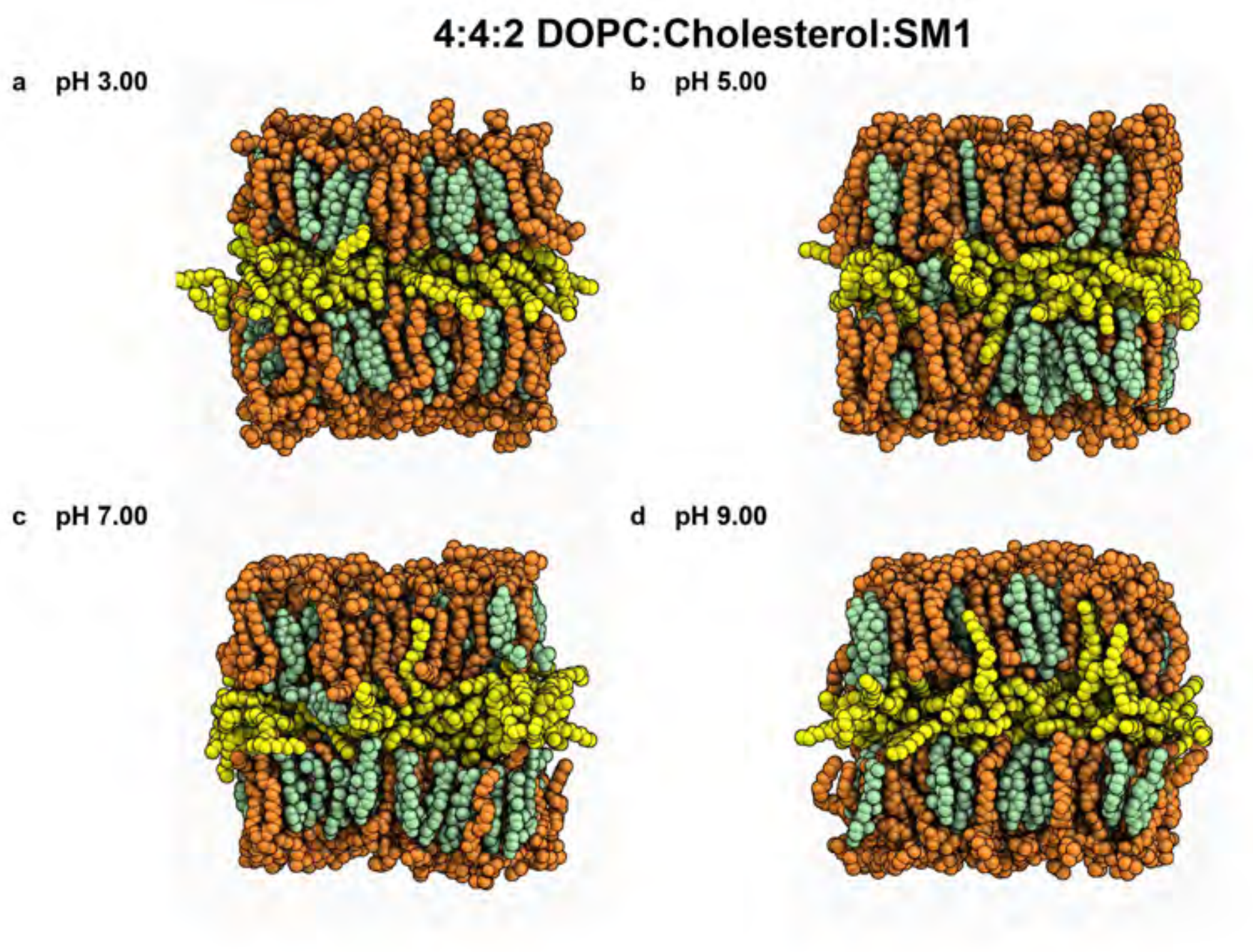

Figure S93: Side-view snapshots of the membrane in the final frame of the simulation at the corresponding pH values. Simulations were setup by placing deprotonated aminolipids in the membrane core. The helper lipid is shown in orange, cholesterol in green, the protonated amnolipid (net charge + 1) in blue, the deprotonated aminolipid (net charge 0) in yellow, and the aminolipids without a specific state ($0.2 \leq \lambda \leq 0.8$) in grey.

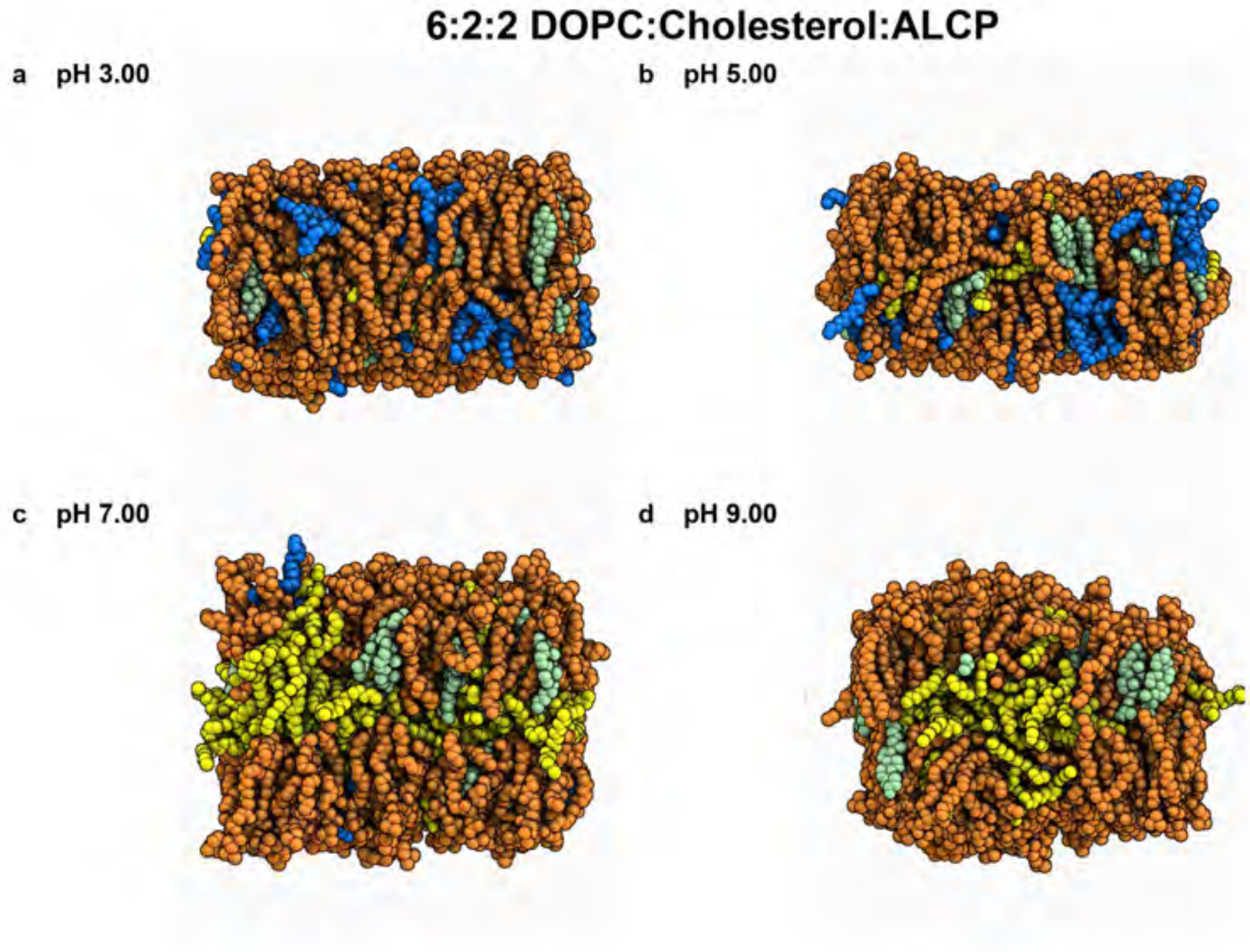

Figure S94: Side-view snapshots of the membrane in the final frame of the simulation at the corresponding pH values. Simulations were setup by placing deprotonated aminolipids in the membrane core. The helper lipid is shown in orange, cholesterol in green, the protonated amnolipid (net charge + 1) in blue, the deprotonated aminolipid (net charge 0) in yellow, and the aminolipids without a specific state ($0.2 \leq \lambda \leq 0.8$) in grey.

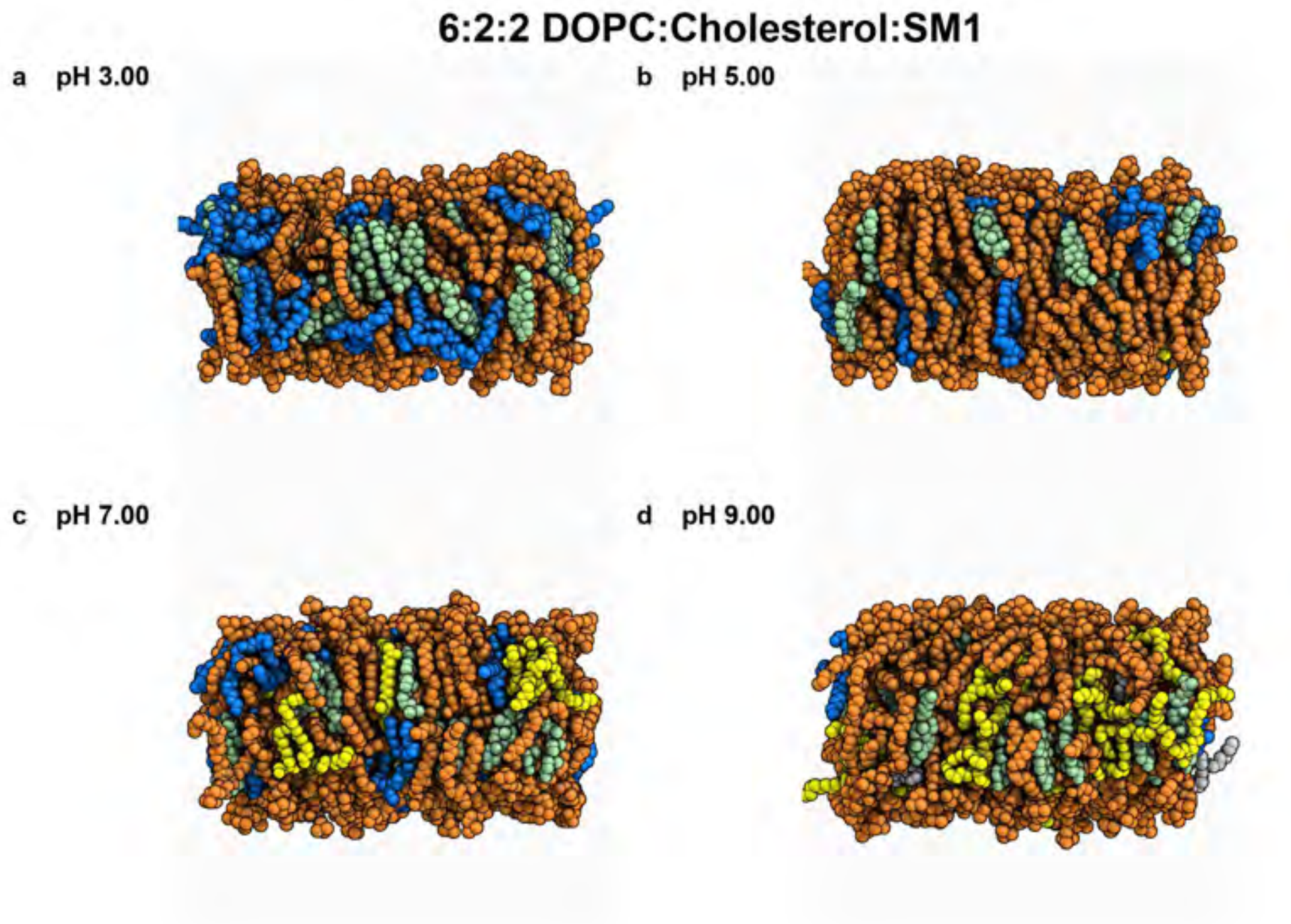

Figure S95: Side-view snapshots of the membrane in the final frame of the simulation at the corresponding pH values. Simulations were setup by placing deprotonated aminolipids in the membrane core. The helper lipid is shown in orange, cholesterol in green, the protonated amnolipid (net charge + 1) in blue, the deprotonated aminolipid (net charge 0) in yellow, and the aminolipids without a specific state ($0.2 \leq \lambda \leq 0.8$) in grey.

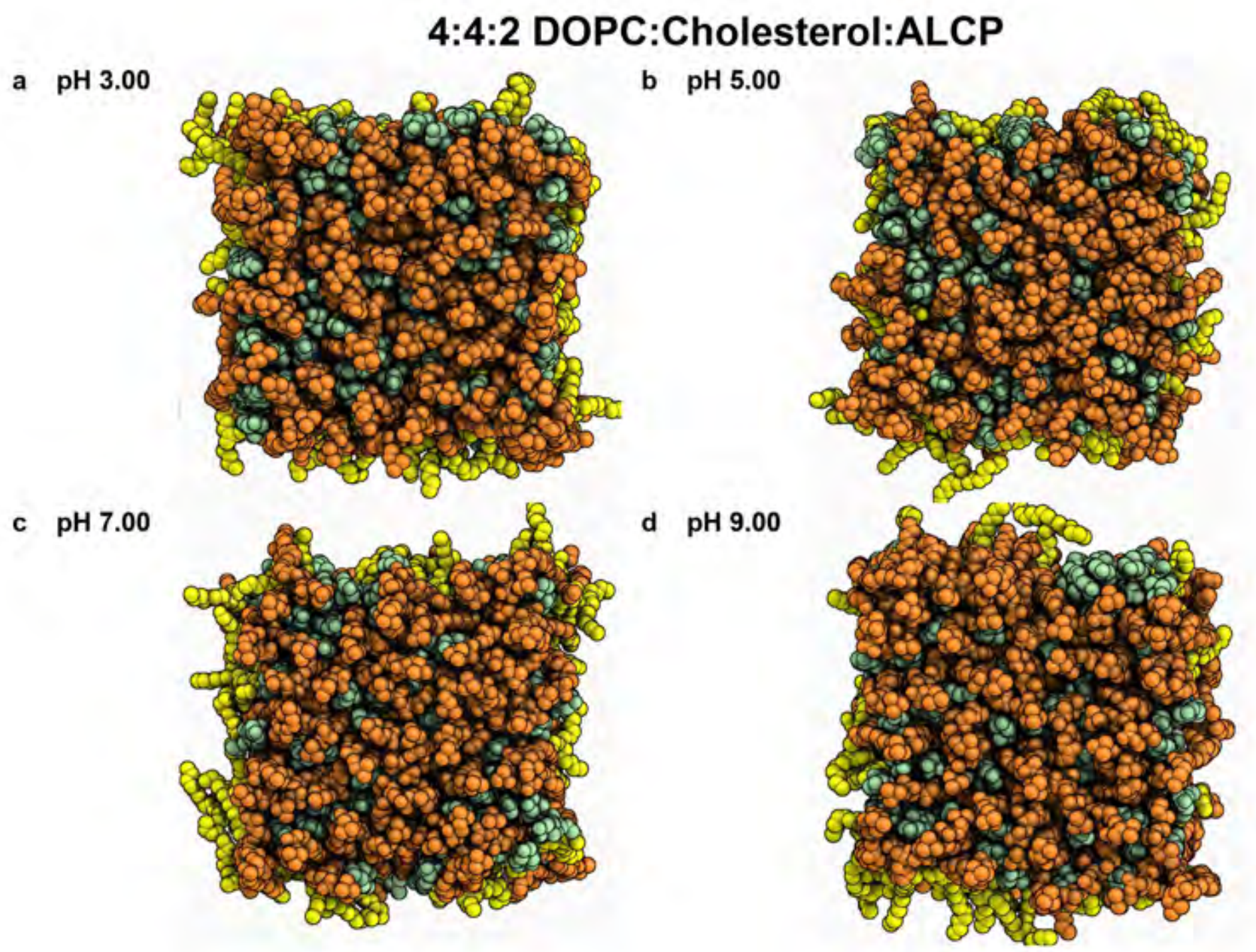

Figure S96: Top-view snapshots of the membrane in the final frame of the simulation at the corresponding pH values. Simulations were setup by placing deprotonated aminolipids in the membrane core. The helper lipid is shown in orange, cholesterol in green, the protonated amnolipid (net charge + 1) in blue, the deprotonated aminolipid (net charge 0) in yellow, and the aminolipids without a specific state ($0.2 \leq \lambda \leq 0.8$) in grey.

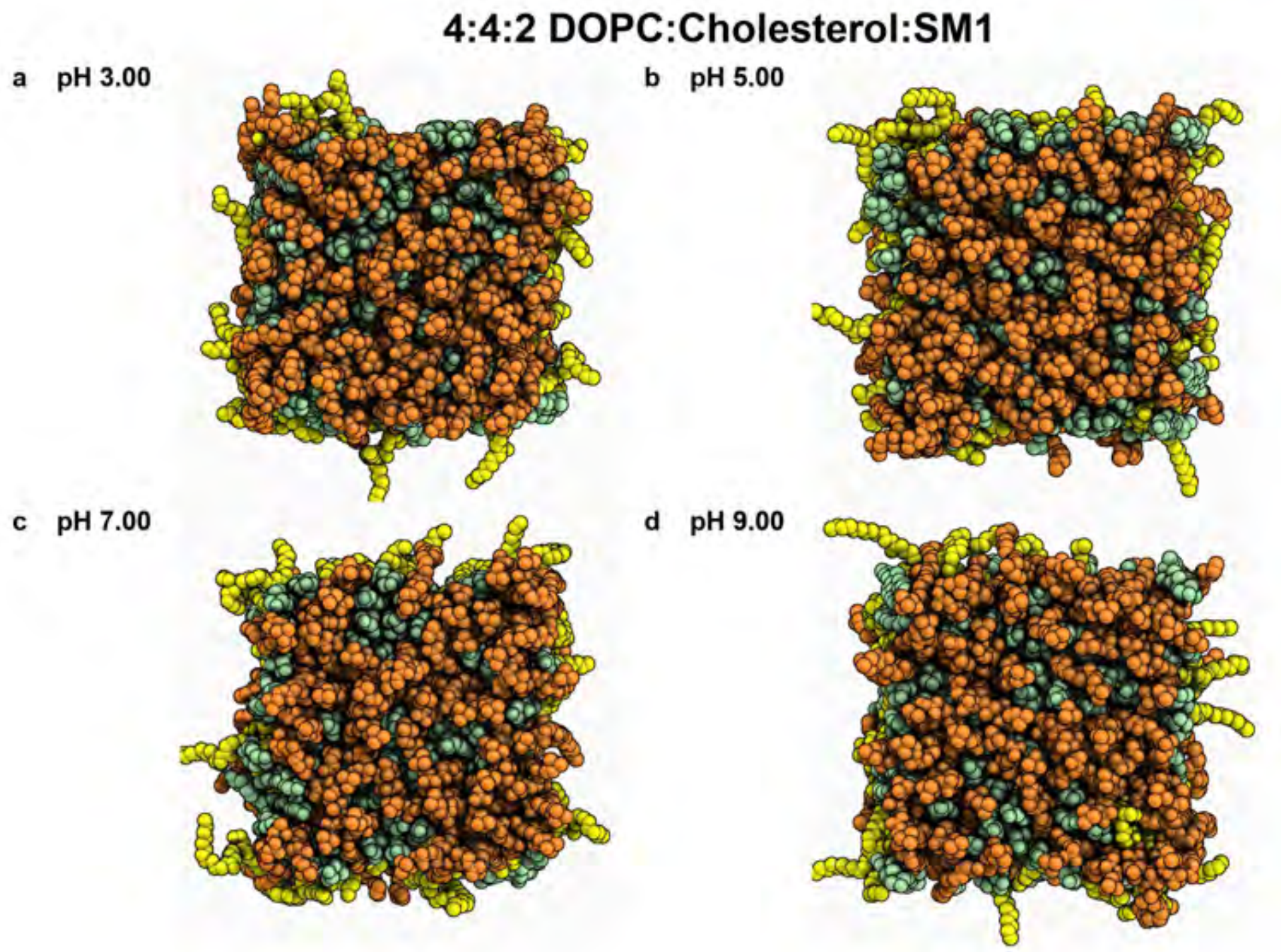

Figure S97: Top-view snapshots of the membrane in the final frame of the simulation at the corresponding pH values. Simulations were setup by placing deprotonated aminolipids in the membrane core. The helper lipid is shown in orange, cholesterol in green, the protonated amnolipid (net charge + 1) in blue, the deprotonated aminolipid (net charge 0) in yellow, and the aminolipids without a specific state ($0.2 \leq \lambda \leq 0.8$) in grey.

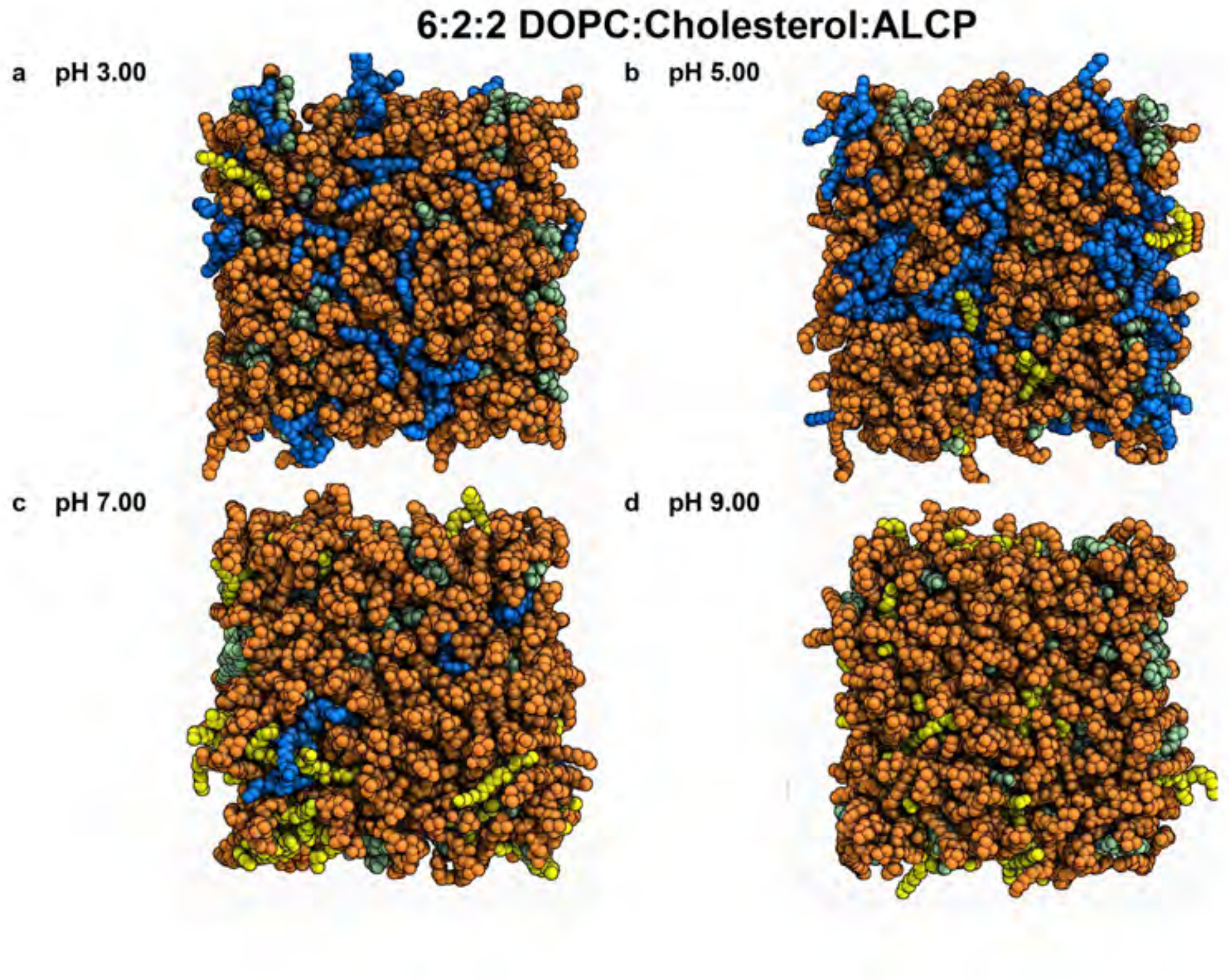

Figure S98: Top-view snapshots of the membrane in the final frame of the simulation at the corresponding pH values. Simulations were setup by placing deprotonated aminolipids in the membrane core. The helper lipid is shown in orange, cholesterol in green, the protonated amnolipid (net charge + 1) in blue, the deprotonated aminolipid (net charge 0) in yellow, and the aminolipids without a specific state ($0.2 \leq \lambda \leq 0.8$) in grey.

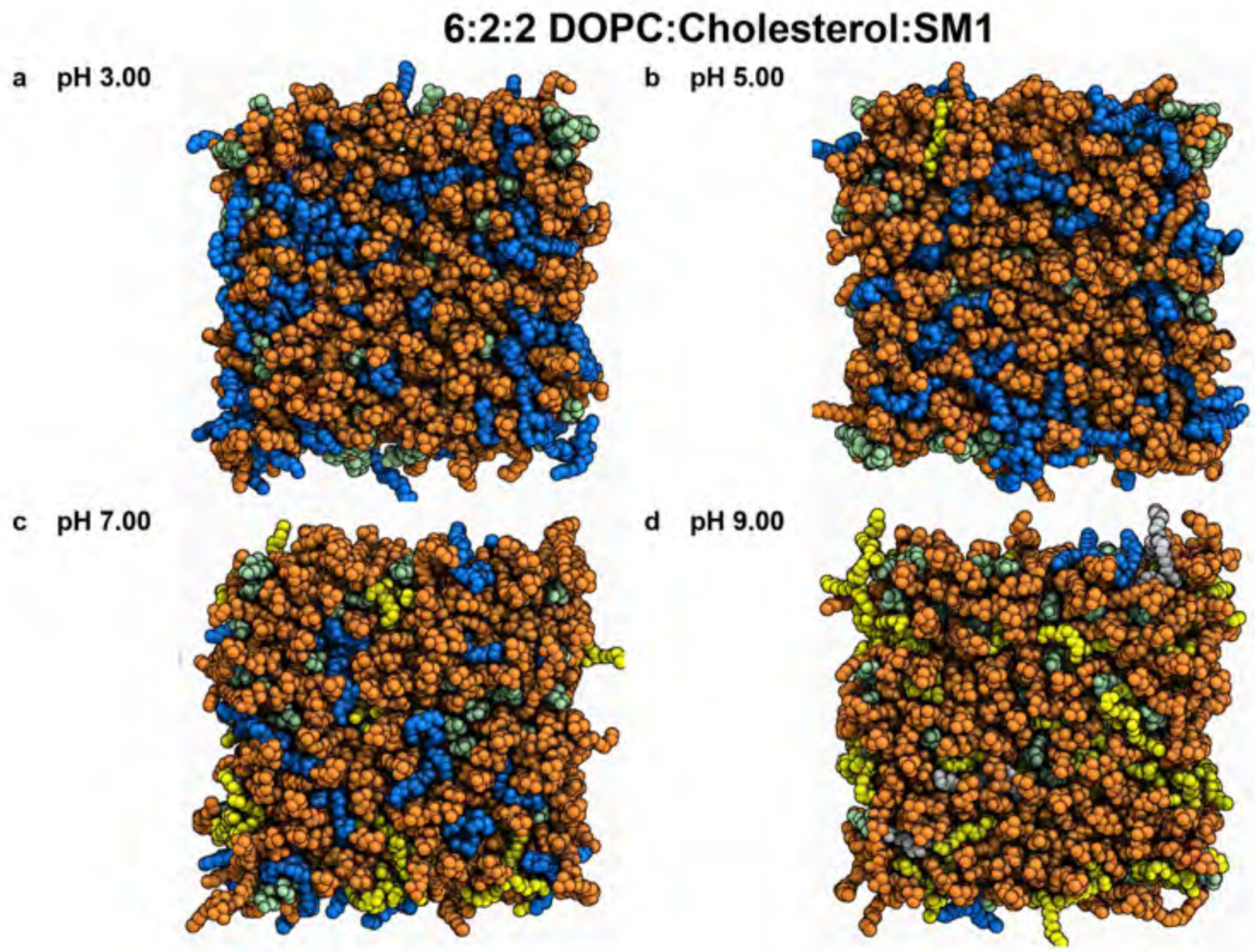

Figure S99: Top-view snapshots of the membrane in the final frame of the simulation at the corresponding pH values. Simulations were setup by placing deprotonated aminolipids in the membrane core. The helper lipid is shown in orange, cholesterol in green, the protonated amnolipid (net charge + 1) in blue, the deprotonated aminolipid (net charge 0) in yellow, and the aminolipids without a specific state ($0.2 \leq \lambda \leq 0.8$) in grey.

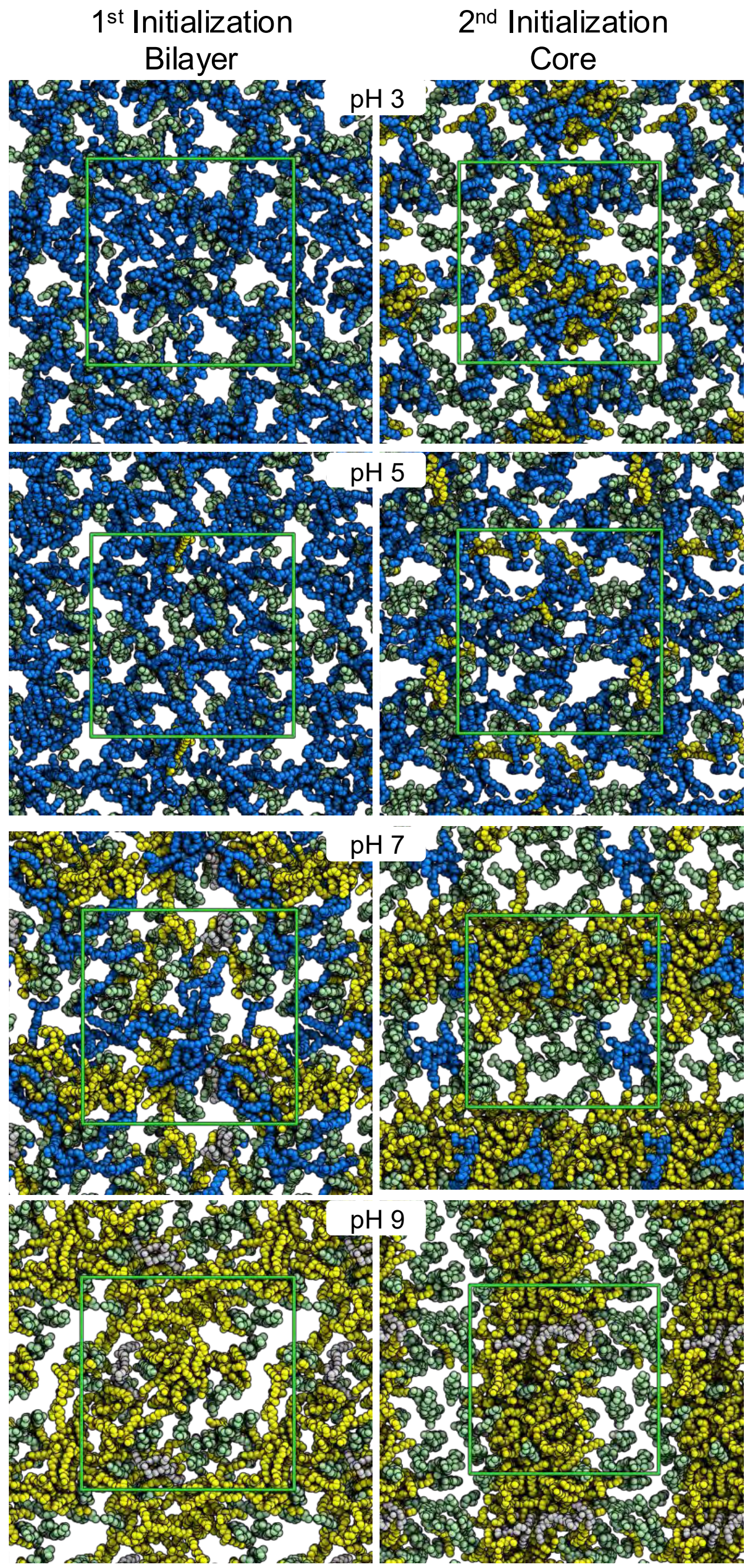

Figure S100: Top-view snapshots of the final simulation frame obtained from the two initialization strategies for ALC-0315 in the 6:2:2 DOPC:cholesterol:aminolipid composition: a bilayer with all aminolipids initially protonated (left) and a surface–core structure with initially deprotonated aminolipids (right), shown at the corresponding pH values in the absence of helper lipids. Cholesterol is shown in green, the protonated amnolipid (net charge + 1) in blue, the deprotonated aminolipid (net charge 0) in yellow, and the aminolipids without a specific state ($0.2 \leq \lambda \leq 0.8$) in grey.

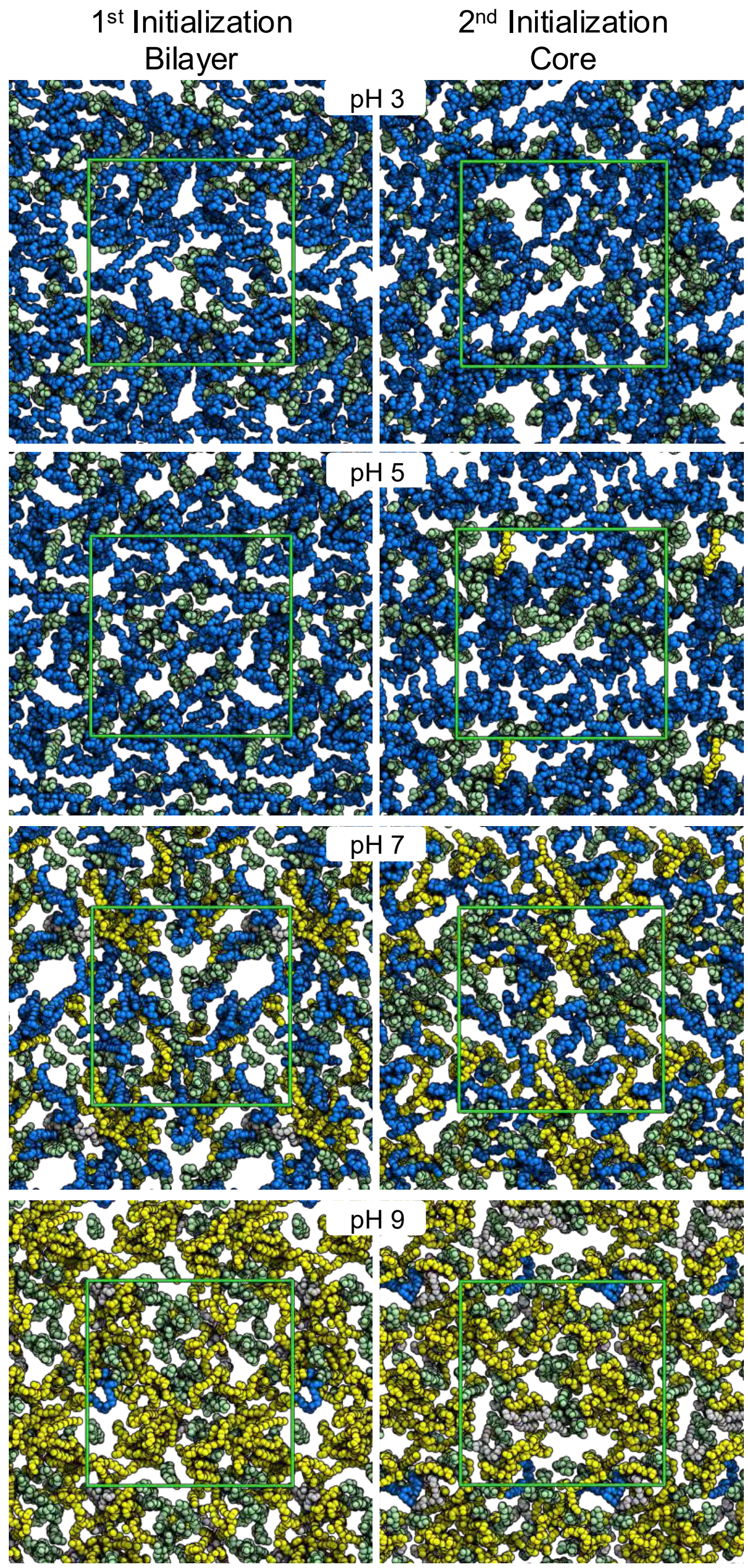

Figure S101: Top-view snapshots of the final simulation frame obtained from the two initialization strategies for SM-102 in the 6:2:2 DOPC:cholesterol:aminolipid composition: a bilayer with all aminolipids initially protonated (left) and a surface–core structure with initially deprotonated aminolipids (right), shown at the corresponding pH values in the absence of helper lipids. Cholesterol is shown in green, the protonated amnolipid (net charge + 1) in blue, the deprotonated aminolipid (net charge 0) in yellow, and the aminolipids without a specific state ($0.2 \leq \lambda \leq 0.8$) in grey.

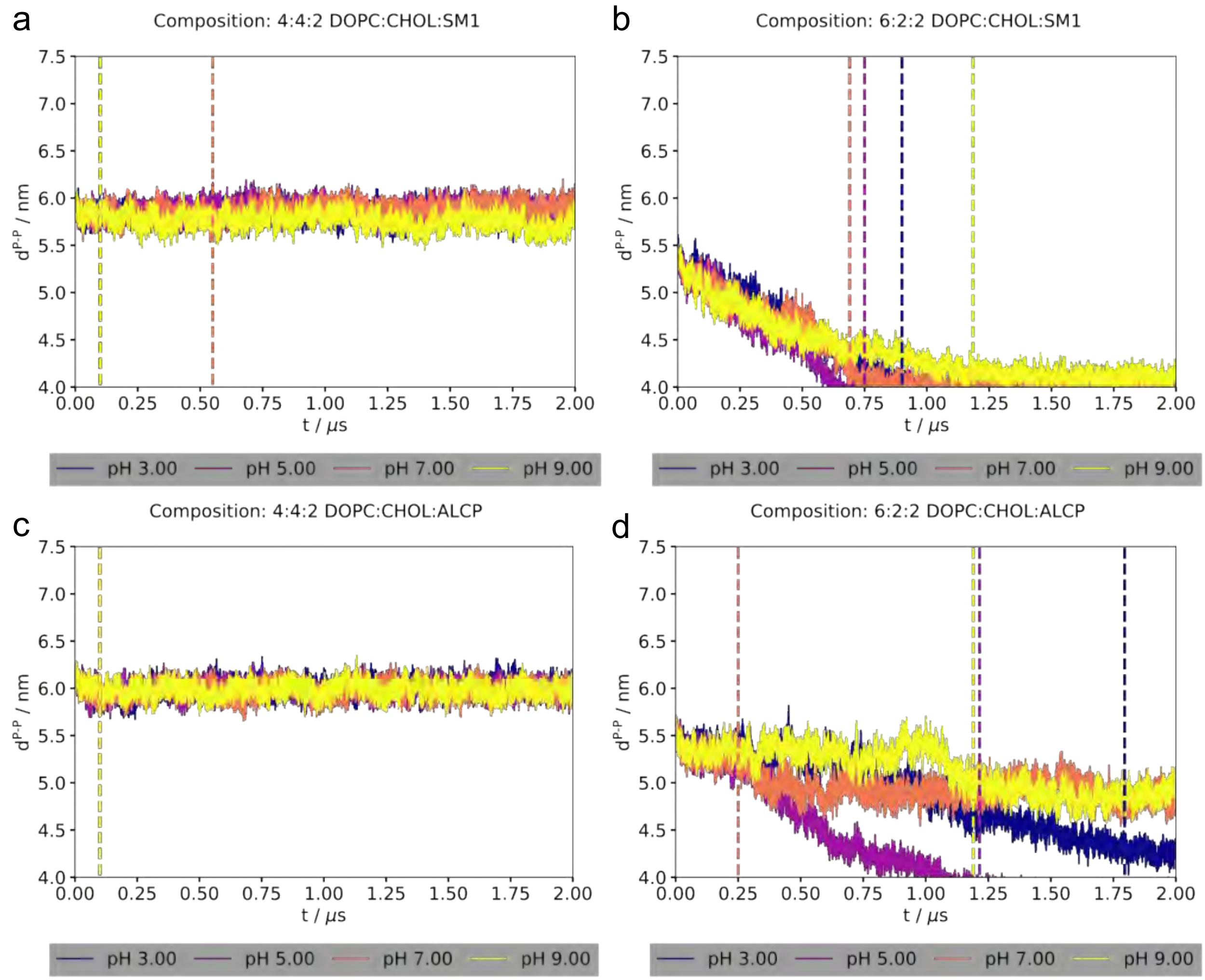

Figure S102: Membrane thickness per frame for systems with **a**,**c** a 4:4:2 or **b**,**d** a 6:2:2 helper lipid:cholesterol:aminolipid composition containing either **a**,**b** SM-102, or **c**,**d** ALC-0315 as the aminolipid. Simulations were setup by placing deprotonated aminolipids in the membrane core. Thickness is defined as the distance between the median z-positions (no trigonometric approach) of the phosphorus atoms in the upper and lower leaflets. Vertical dashed lines indicate the equilibration time determined for each pH value. Abbreviations used: ALCP = ALC-0315, SM1 = SM-102, CHOL = cholesterol.

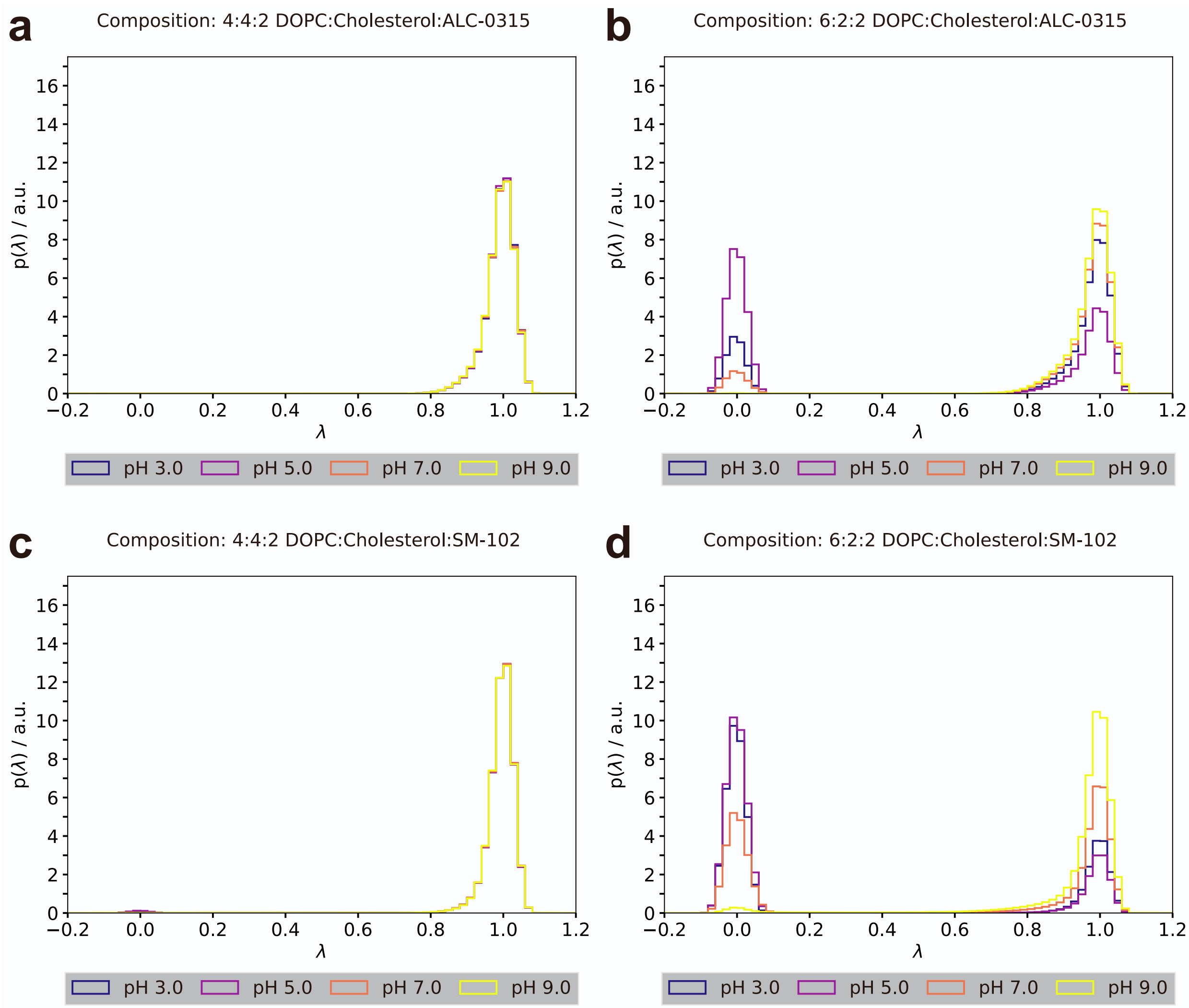

Figure S103: Distribution of the λ-coordinate at each pH, aggregated over all aminolipids in a membrane with **a**,**c** a 4:4:2 or **b**,**d** a 6:2:2 DOPC:cholesterol:aminolipid composition containing either **a**,**b** ALC-0315 or **c**,**d** SM-102 as the aminolipid. Simulations were setup by placing deprotonated aminolipids in the membrane core.

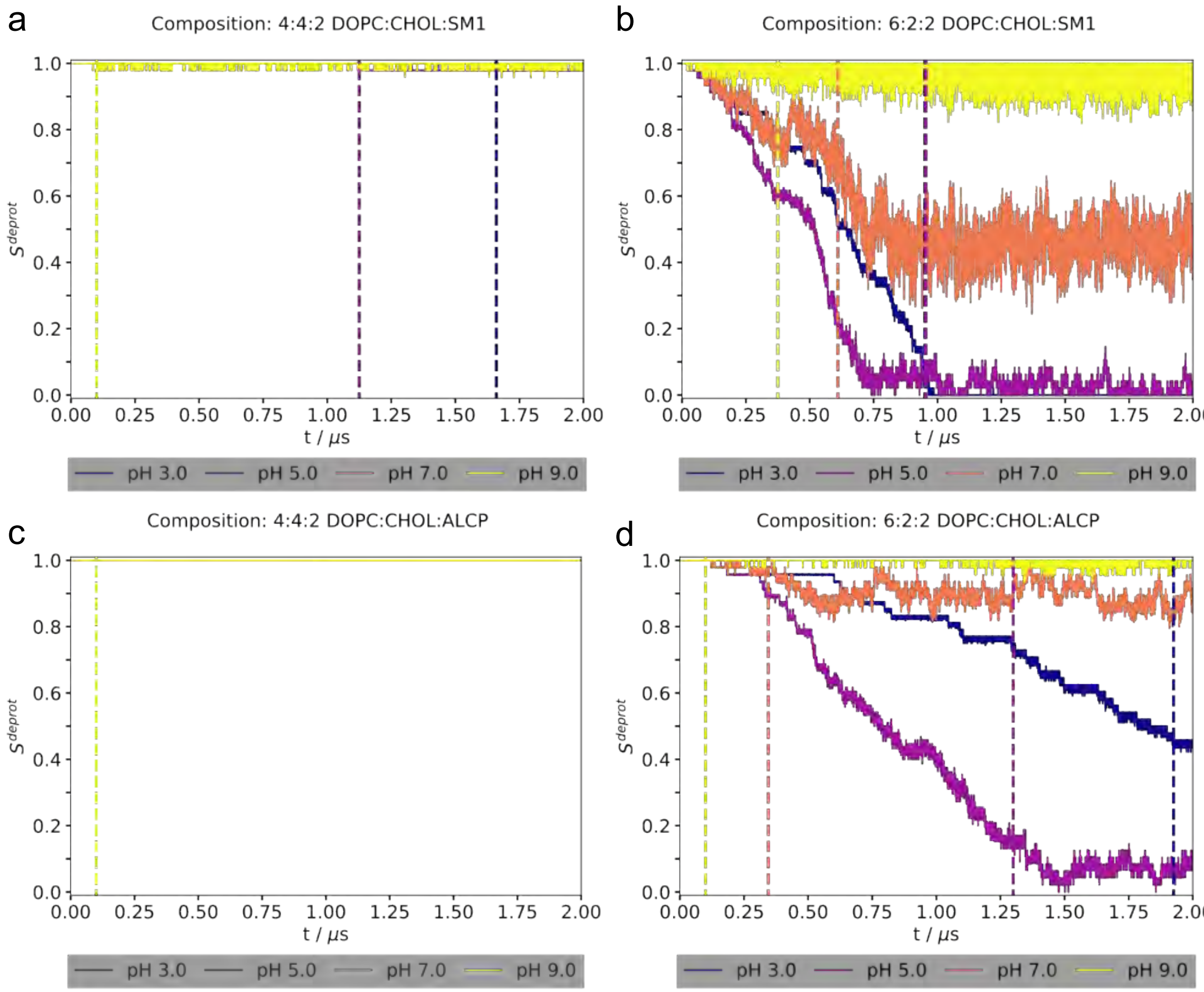

Figure S104: Fraction of deprotonated aminolipids (S^{deprot}) per frame for membranes with **a**,**c** a 4:4:2 or **b**,**d** a 6:2:2 DOPC:cholesterol:aminolipid composition containing either **a**,**b** ALC-0315 or **c**,**d** SM-102 as the aminolipid. Simulations were setup by placing deprotonated aminolipids in the membrane core. Vertical dashed lines indicate the equilibration time determined for each pH value. Abbreviations used: ALCP = ALC-0315, SM1 = SM-102, CHOL = cholesterol.

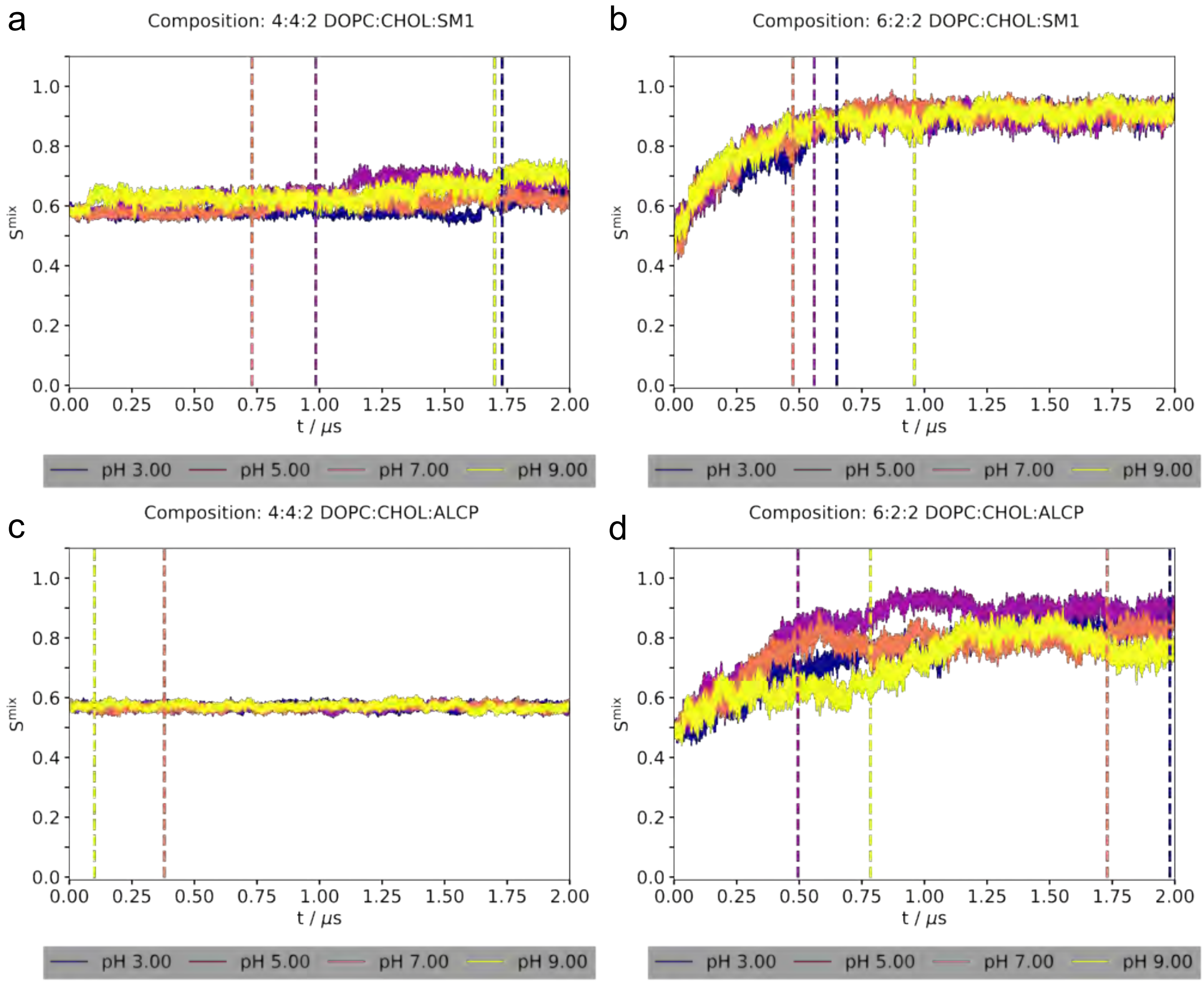

Figure S105: Conditional mixing entropy (S^{mix}) per frame for membranes with **a**,**c** a 4:4:2 or **b**,**d** a 6:2:2 DOPC:cholesterol:aminolipid composition containing either **a**,**b** SM-102 or **c**,**d** ALC-0315 as the aminolipid. Simulations were setup by placing deprotonated aminolipids in the membrane core. Vertical dashed lines indicate the equilibration time determined for each pH value. Conditional mixing entropies were calculated following Brandani *et al.*,[3] using a 1 nm cutoff to define neighboring lipids. Contacts were defined based on the distance between reference atoms in the headgroups of the target lipids: the titratable nitrogen for aminolipids, the oxygen atom fo cholesterol, and the sn-2 carbon atom for phospholipids. Abbreviations used: ALCP = ALC-0315, SM1 = SM-102, CHOL = cholesterol.

References

(1) Trollmann, M. F.; Rossetti, P.; Böckmann, R. A. Constant-pH MD Simulations of Lipids. *bioRxiv* **2024**, 2024–12.

(2) Trollmann, M. F. W.; Böckmann, R. A. Decoding pH-Driven Phase Transition of Lipid Nanoparticles. *Small* **2026**, e11381.

(3) Brandani, G. B.; Schor, M.; Macphee, C. E.; Grubmüller, H.; Zachariae, U.; Marenduzzo, D. Quantifying disorder through conditional entropy: an application to fluid mixing. *PLoS One* **2013**, *8*, e65617.
