# Supplementary material for: Membrane environment sets the functional pKa of ionizable lipids
Source: Biophys J. 2026 Jun 26;125(15):3918–37. doi: 10.1016/j.bpj.2026.06.030 (PMC13507446; doi:10.1016/j.bpj.2026.06.030)
Supplement: Supplementary file 2 [file mmc2.pdf]

Biophysical Journal
Article

Membrane environment sets the functional pK_a of ionizable lipids

Marius F. W. Trollmann,[1,2,*] Paolo Rossetti,[1] and Rainer A. Böckmann[1,2,3,4,*]
[1]Computational Biology, Department of Biology, Friedrich-Alexander-Universität Erlangen-Nürnberg, Erlangen, Germany; [2]Erlangen National High-Performance Computing Center (NHR@FAU), Erlangen, Germany; [3]FAU Profile Center Immunomedicine (FAU I-MED), Erlangen, Germany; and [4]FAU Research Center New Bioactive Compounds – FAU NeW, Erlangen, Germany

ABSTRACT Ionizable aminolipids enable lipid nanoparticles (LNPs) to encapsulate nucleic acids at neutral pH and to release their cargo upon endosomal acidification. The discrepancy between this effective, acidic LNP pK_a and the basic intrinsic pK_a of aminolipids, however, remains poorly understood. Here, we performed microsecond constant-pH molecular dynamics simulations of five widely used aminolipids (DODAP, DLin-MC3-DMA, DLin-KC2-DMA, ALC-0315, and SM-102) embedded in different LNP-relevant ternary DOPC/DSPC-cholesterol membranes to quantify how aminolipid structure and membrane composition jointly govern aminolipid protonation and the associated pH-dependent membrane remodeling. Across all systems, membrane embedding lowers the apparent aminolipid pK_a, yielding physiologically relevant values of 6–7.5 corresponding to shifts by up to 3.5 pK_a units or approx. 20 kJ mol^{-1} with respect to the intrinsic pK_a. Strikingly, pH-driven membrane remodeling upon deprotonation differs between aminolipids: polyunsaturated aminolipids undergo surface-to-core translocation, branched aminolipids preferentially form laterally segregated surface domains, and DODAP remains interfacially anchored through sustained hydration and hydrogen bonding. Saturated helper lipids (DSPC) systematically enhance segregation and amplify pK_a shifts relative to DOPC. Together, these results identify membrane phase behavior as a primary regulator of aminolipid protonation equilibria and provide insights into how formulation may influence LNP pK_a and thereby could control membrane remodeling and delivery performance.

SIGNIFICANCE The efficiency of lipid nanoparticle (LNP) formulations for delivering RNA therapeutics depends on the protonation behavior of their aminolipids. While protonated aminolipids drive nucleic acid encapsulation and endosomal membrane fusion, charge-neutral LNPs are required during systemic circulation to reduce toxicity. Designing improved LNPs therefore requires understanding how membrane environment and aminolipid structure determine protonation. Here, large-scale all-atom constant-pH molecular dynamics simulations at microsecond timescales were used to characterize environment-dependent pK_a values of aminolipids in LNP-relevant membranes. Our simulations reveal how membrane composition and aminolipid structure influence dynamic protonation patterns and pH-dependent membrane reorganization. These findings provide a molecular basis for understanding aminolipid behavior in model membranes resembling LNP surfaces and illustrate how constant-pH simulations can support the rational design of next-generation RNA delivery systems.

INTRODUCTION

The delivery of nucleic acids using lipid nanoparticle (LNP) formulations has opened new avenues for the development of innovative therapies and vaccines targeting a wide spectrum of diseases.[1] Structurally, LNPs consist of a hydrophobic core surrounded by a lipid monolayer.[1,2] The negatively charged nucleic acids can be efficiently encapsulated and stabilized within LNPs through the incorporation of cationic lipids.[3]

LNPs typically comprise four different lipid components: aminolipids, helper lipids, cholesterol, and lipids conjugated to polyethylene glycol (PEG-lipids), each fulfilling a distinct functional role. These components are subject to diverse optimization strategies, resulting in a broad and highly tunable LNP compositional space. Aminolipids are the predominant component (usually 40%–50%) of LNPs[4] and their pH responsiveness is central to loading or encapsulation efficiency, their bio-distribution, safety, and intracellular payload delivery.[5]

Submitted March 22, 2026, and accepted for publication June 23, 2026.
*Correspondence: marius.trollmann@fau.de or rainer.boeckmann@fau.de
Editor: Heiko Heerklotz.
https://doi.org/10.1016/j.bpj.2026.06.030
© 2026 The Author(s). Published by Elsevier Inc. on behalf of Biophysical Society.
This is an open access article under the CC BY license (http://creativecommons.org/licenses/by/4.0/).

Cholesterol is the second most abundant component ($\approx 40\%$ in currently reported LNP formulations) and provides support to surface-core organization of LNPs, conferring fluidity while keeping a tight lipid packing preventing payload escape. The substitution of cholesterol with alternative sterols may affect delivery efficiency and favor different tissue tropism, although the precise mechanism is not clear.[6] Notably, chimeric molecules joining the two major LNP components (aminolipid and sterol) achieved satisfactory safety and efficacy profiles both *in vitro* and *in vivo*,[7] highlighting the untapped potential for exploring novel LNP components.

Helper lipids are usually zwitterionic and bilayer-forming phospholipids. While DSPC is included in all three US Food and Drug Administration (FDA)-approved LNP compositions, sphingomyelin (SM) and ceramides have been proposed as alternative helper lipids in several patents.[8,9] Importantly, SM lipids have been shown to confer peculiar bio-distribution properties,[10] highlighting helper-lipid type and molar ratio as critical parameters for further LNP optimization.

PEG-lipids are typically included at low molar fractions (1%–2%) to prevent LNP aggregation and prolong their half-life *in vivo*.[11] The lengths of the PEG chain and of the hydrophobic anchor govern PEG desorption kinetics, thereby influencing LNP biodistribution[12]: Shorter hydrophobic anchors favor faster desorption and early formation of the protein corona promoting liver tropism, while bulkier anchors prolong LNP circulation time and tumor accumulation[13] but may cause anti-PEG immunogenicity.[14] Recent developments in the design of novel LNP formulations have removed PEG-lipids entirely from the composition.[15]

Early aminolipids were based on quaternary amine headgroups, rendering them permanently cationic. This inherent cationicity facilitates strong electrostatic interactions with nucleic acids, yielding overall positively charged LNP formulations. While such formulations are generally considered beneficial for cellular uptake by promoting interactions with the negatively charged cell surface and glycocalyx,[3,16] permanently cationic lipids have been shown to induce hepatotoxicity, lung toxicity, and result in rapid clearance of LNPs from the bloodstream due to pronounced first-pass effects.[17]

These limitations were largely overcome by the development of cationic ionizable aminolipids (CILs), which contain secondary or tertiary amine groups capable of undergoing dynamic (de)protonation.[3] CILs are characterized by the following pH-dependent behavior:

- At acidic pH (≈ 4), CILs are protonated, enabling efficient nucleic acid encapsulation.[3]
- At physiological pH (7.4), CILs adopt a deprotonated, neutral state, resulting in the formation of LNPs with a lipidic core composed of deprotonated aminolipids and cholesterol, surrounded by a lipid monolayer. In mRNA-loaded LNPs, the negative charge of the nucleic acids is shielded by locally protonated aminolipids.[2,18]
- Upon endosomal acidification (≈ 6), CILs re-protonate at the LNP surface,[18] enhancing electrostatic interactions with anionic endosomal lipids.[19] In addition, the bilayer-destabilizing properties of CILs likely promote membrane fusion and thereby nucleic acid release.[1]

Accordingly, the protonation behavior of aminolipids is central to LNP function. It is determined by the intrinsic pK_a of the aminolipid defined as the pK_a of the functional ionizable group in infinite dilution conditions (pK_a^{AL}). Experimentally, pK_a^{AL} values are typically determined by potentiometric titration of water-soluble aminolipid analogs and can be tuned by modifying the distance between the ionizable nitrogen and the electron-withdrawing groups (Figure 1).[5]

In contrast, the apparent pK_a of full LNPs (pK_a^{LNP}) depends not only on the intrinsic pK_a^{AL} but additionally on the local LNP composition, structure, and molecular organization and on the particular method employed to measure the pK_a^{LNP}. While reported intrinsic pK_a^{AL} values range from 7 to 10 (Figure 1c), effective LNP formulations consistently exhibit pK_a^{LNP} values between 6 and 7, i.e., consistently slightly below physiological pH.

A widely used approach to determine pK_a^{LNP} is the TNS-binding assay (TNS, 6-(p-Toluidino)-2-naphthalenesulfonic acid),[22] which monitors fluorescence changes upon TNS partitioning into LNP membranes. Because TNS binding increases with membrane positive charge—and thus aminolipid protonation—this method provides an indirect measure of membrane charge. We previously demonstrated that TNS localizes predominantly within the LNP monolayer shell; consequently, pK_a^{LNP} values obtained from TNS assays primarily reflect aminolipid protonation in the LNP shell rather than in the core.[18] Accordingly, TNS assays do not allow us to sense the protonation states of aminolipids residing in the LNP core.[5,20] This limitation has recently been addressed using DNA-based fluorescent probes, which revealed that the LNP core is highly permeable to protons.[23]

Beyond intrinsic and LNP-level pK_a values, one may further define the apparent aminolipid pK_a^{app} as the pH at which the probability of aminolipid protonation equals that of deprotonation within a given environment. This apparent pK_a^{AL} depends on the local lipid environment, including surrounding lipid species and aminolipid density. Classical molecular dynamics (MD) simulations have been successfully employed to estimate pK_a^{app} values in defined environments[4]; however, such approaches neglect cooperative protonation effects and typically rely on predefined protonation states. In contrast, constant-pH molecular dynamics (CpHMD) simulations[24] enable unbiased sampling of dynamic protonation equilibria of aminolipids as a function of the local environment.[18,25]

The function of aminolipids is strongly influenced by both their intrinsic pK_a^{AL} and their molecular shape.[26] While the

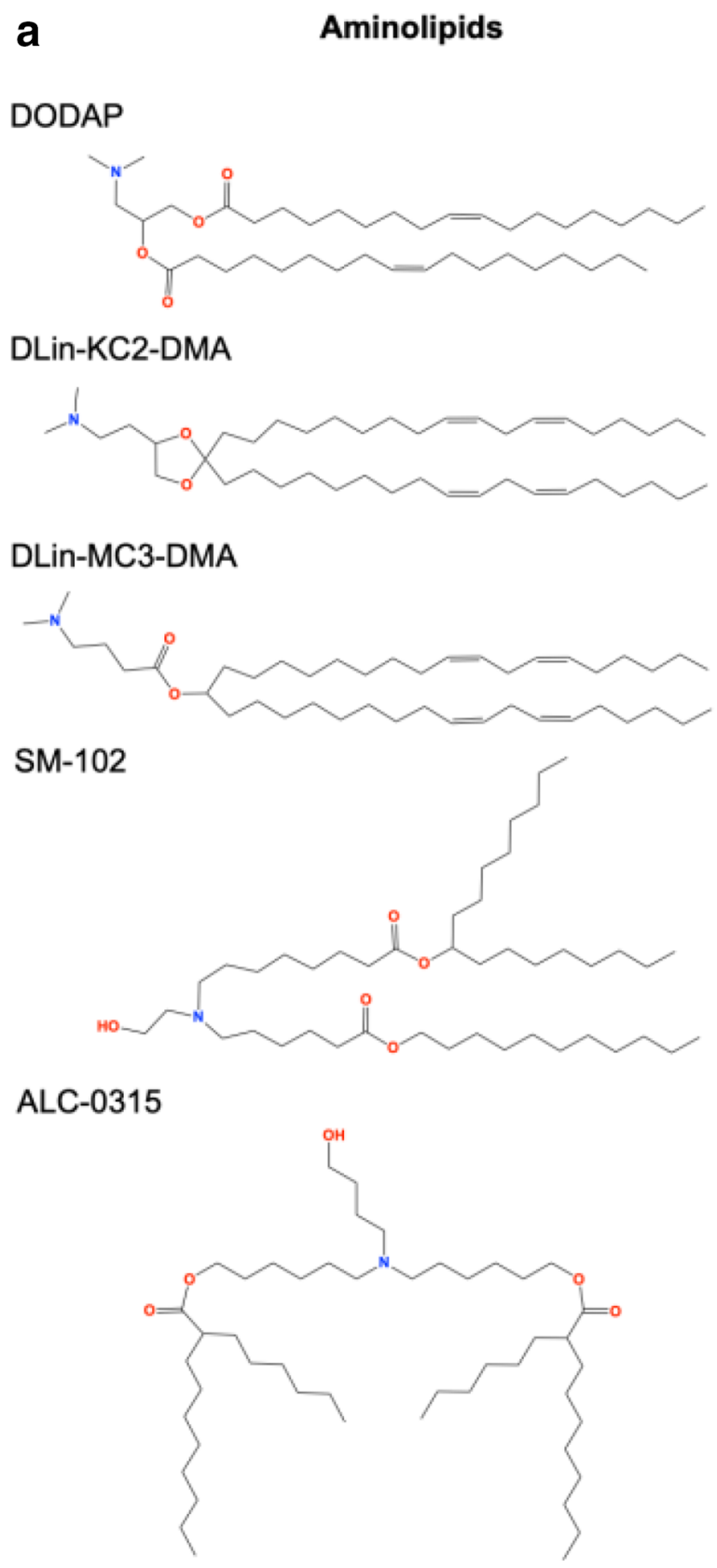

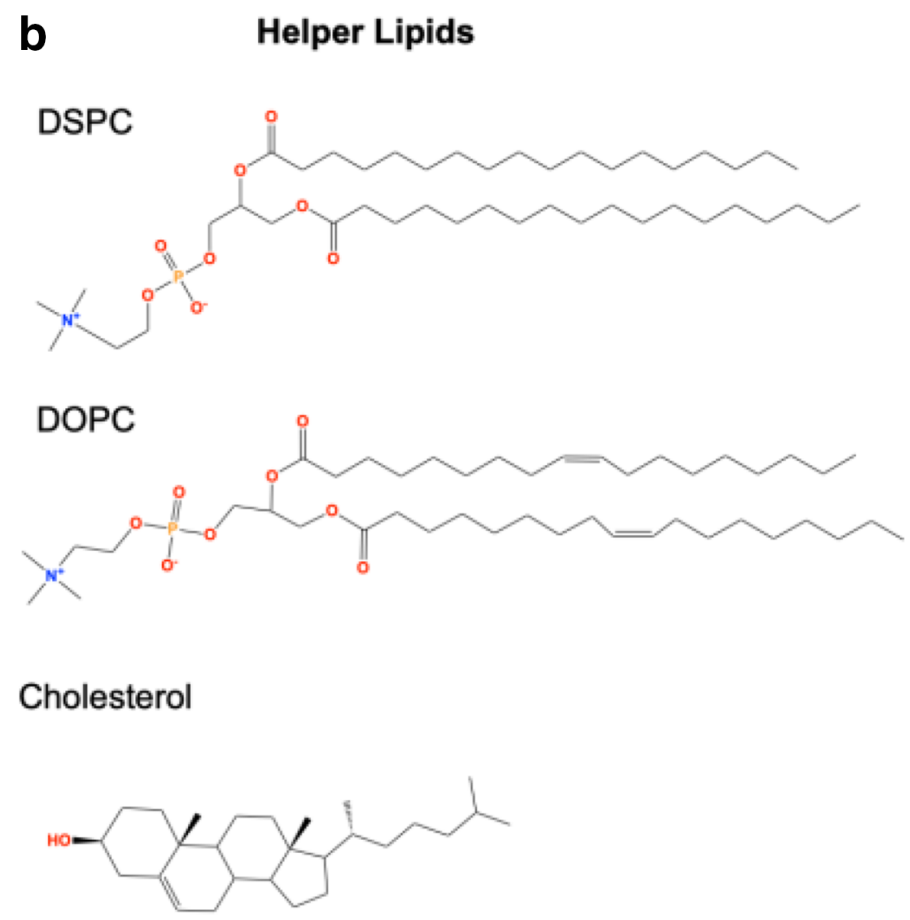

c

Aminolipid	Intrinsic pK_a
DODAP	7.65
DLin-KC2-DMA	9.34
DLin-MC3-DMA	9.4
SM-102	8.9
ALC-0315	9.26

Figure 1. (a) Chemical structures of the aminolipids investigated in this study. (b) Helper phospholipids used in model membranes. (c) Intrinsic pK_a values of DODAP (3-(Dimethylamino)-1,2-propanediyl (9Z,9'Z)bis(-9-ocatdecenoate), DLin-KC2-DMA (2-{2,2-Di[(9Z,12Z)-9,12-octadecadien-1-yl]-1-yl]-1,3-dioxolan-4-yl}-N,N-dimethylethanamine), DLin-MC3-DMA ((6Z,9Z,28Z,31Z)-6,9,28,31-Heptatriacontatetraen-19-yl 4-(dimetyhlamino) butanoat),[20] ALC-0315 ([(4-Hydroxybutyl) imino]di-6,1-hexanediyl bis(2-hexyldecanoate)),[4,21] and SM-102 (9-Heptadecanyl 8-{(2-hydroxyethyl)[6-oxo-6-(undecyloxy)hexyl]amino}octanoate).[4,21]

headgroup chemistry is decisive for the intrinsic pK_a^{AL}, the number and saturation of fatty acyl chains govern membrane embedding and aminolipid shape, which in turn modulate the pK_a shift, fusogenicity, and membrane remodeling.

The aminolipids most frequently employed in LNP formulations include the following (Figure 1):

- DODAP, which features two oleyl chains linked by a 1,3-propanediol backbone and a tertiary amine headgroup.
- DLin-MC3-DMA (MC3) possesses two linoleyl tails and just one ester group, conferring enhanced hydrophobicity and bilayer-destabilizing properties. MC3 belongs to the Patisiran (Onpattro) LNP composition, the first FDA-approved small interfering RNA (siRNA) and LNP-based therapeutic, used to treat hereditary transthyretin-mediated amyloidosis.[27]
- DLin-KC2-DMA (KC2) shares the acyl chains of MC3 but has a different headgroup domain, characterized by a dioxolane ring.
- ALC-0315, a next-generation, four-tailed aminolipid introduced following the pioneering work of Akinc et al.[28] ALC-0315 is a component of the Pfizer SARS-CoV-2 mRNA vaccine and has demonstrated improved siRNA delivery relative to MC3 in preclinical studies.[29]
- SM-102, the aminolipid used in the Moderna SARS-CoV-2 vaccine, is characterized by a branched three-tail architecture and a slightly lower intrinsic pK_a due to the shorter distance between the hydroxyl group and the tertiary amine.

Contemporary aminolipid design increasingly combines multi-tail architectures with unsaturations, cyclohexyl moieties, and anteiso-branched chains,[8,9,30,31] reflecting continued efforts to expand the aminolipid chemical space.

In this work, we investigate how aminolipid structure and lipid environment jointly influence protonation equilibria and pH-dependent membrane remodeling. Specifically, five well-characterized aminolipids (see above) spanning different stages of structural evolution are studied in membranes with varying helper phospholipid composition and cholesterol content using microsecond-scale all-atom CpHMD simulations. Our analysis focuses on aminolipid pK_a shifts ($\Delta pK_a = pK_a^{app} - pK_a^{AL}$) within ternary membranes of differing acyl-chain saturation and cholesterol concentration, as well as on the pH-dependent membrane structure and organization.

MATERIALS AND METHODS

System preparation

Systems containing ALC-0315 and SM-102 were assembled in a bilayer configuration together with the corresponding helper lipid and cholesterol using the MemPackGen tool[32] from the AmberTools suite.[33] Systems containing MC3,

KC2, and DODAP were prepared using CHARMM-GUI,[34] employing the protonated form of the respective aminolipid in combination with the helper lipid and cholesterol.

In all cases, the membrane systems were solvated with approximately 12,000 TIPS3P[35] water molecules, corresponding to a hydration level of roughly 50 water molecules per lipid, and neutralized by the addition of chloride counterions.

Core initialization

Initial structures with all 48 aminolipids fully deprotonated ($\lambda_i^{\text{init}} = 1\ \forall\, i \in [1\ldots48]$) and embedded in the membrane core were generated for the branched aminolipids ALC-0315 and SM-102 using only DOPC as phospholipid. Two membranes with DOPC:cholesterol ratios of 3:1 and 1:1 were built with CHARMM-GUI's Membrane Builder[34] (no added ions), equilibrated following the recommended CHARMM-GUI workflow, and subsequently simulated for 100 ns to relax the area per lipid. The leaflets of the resulting structure were then translated along the *z* axis to accommodate a pre-equilibrated patch of 48 neutral aminolipids.

The aminolipid patch was prepared by compressing a cube of 48 neutral aminolipids—at their equilibrium densities ($\rho^{\text{ALC}-0315} = 887.1 \pm 1.94$ kg m^{-3}; $\rho^{\text{SM}-102} = 893.46 \pm 1.71$ kg m^{-3})—using the c-rescale barostat under semi-isotropic pressure coupling ($P^z = 150$ bar, $P^{xy} = 0.05$ bar, $\tau_P = 5.0$ ps, compressibility $= 4.5 \times 10^{-6}$ bar^{-1}). A snapshot was extracted once the *x*/*y* dimensions matched the lateral area of the target membrane.

The combined structure contained severe atomic clashes and was therefore energy minimized in two stages. In the first stage, Lennard-Jones and Coulombic interactions of the aminolipids were scaled by a factor of 0.1, with a soft-core scheme[36] applied to the Lennard-Jones potential (`sc-alpha` = 0.5, `sc-power` = 1, `sc-sigma` = 0.3); steepest-descent minimization was run for up to 100 steps with an initial step size of 0.01 nm. In the second stage, unscaled non-bonded interactions were used with steepest descent (step size 0.001 nm, maximum 75 steps). Throughout both stages, dihedral restraints ($k^{\text{dihres}} = 100$ kJ mol^{-1}) were applied to the chiral carbon atoms of DOPC and ALC-0315. The minimized structure was then equilibrated in a 1 ns NPT simulation (time step 1 fs) using the MD parameters described above, and visually inspected for residual clashes or ring penetrations before serving as input for the CpHMD simulations. The number of buffer particles was estimated from the charge fluctuations and expected protonation fractions observed in the 2-μs bilayer simulations. All preparatory simulations used GROMACS 2025.3.[37]

Force-field parameters

All molecular mechanics parameters for the aminolipids were derived using the CHARMM General Force Field (CGenFF)[38] in combination with the CHARMM36 lipid force field.[39] Parameters for the protonated and deprotonated states of MC3, KC2, and DODAP were adopted from the work of Park et al.[40] Force-field parameters for ALC-0315 were taken from Trollmann and Böckmann.[18] SM-102 was parameterized following the same workflow previously established for ALC-0315.[18]

An initial topology for SM-102 was generated using the CGenFF program (v4.0),[41–43] compatible with CGenFF v4.6. Partial atomic charges were assigned based on standard CHARMM chemical moieties.[38,39] All bonded parameters for the protonated form of SM-102 exhibited penalty scores below 1.5 and were therefore accepted without further refinement.

Parameters for cholesterol, DSPC (1,2-distearoyl-*sn*-glycero-3-phosphocholine), and DOPC (1,2-Dioleoyl-*sn*-glycero-3-phosphocholine) were taken from the CHARMM36 lipid force field.[39,44,45] All simulations employed the July 2022 release of the CHARMM36 force-field port for GROMACS.

General MD parameters

All MD simulations were performed using the classical leap-frog integrator with a time step of 2 fs. The minimum update frequency of the neighbor list was set to 20 steps using the Verlet cutoff scheme, with the `Verlet-buffer-tolerance` set to the default value of 0.005 kJ mol^{-1} ps^{-1}. Consequently, the short-range neighbor-list cutoff (`rlist`) was automatically adjusted during the simulation.

Electrostatic interactions were computed using the smooth particle-mesh Ewald (SPME) method,[46] with a real-space coulomb cutoff of 1.2 nm and a Fourier grid spacing of 0.14 nm. van der Waals interactions were evaluated with a cutoff of 1.2 nm and smoothly switched off between 1.0 and 1.2 nm using the force-switch modifier.

All simulations were conducted at a reference temperature of 310 K, maintained using the velocity-rescaling (v-rescale) thermostat with a coupling time constant of 1 ps. Two independent temperature-coupling groups were employed: one for all membrane components (lipids) and one for the solvent components (water, counterions, and buffer particles). Pressure was maintained at 1 bar using the c-rescale barostat with semi-isotropic pressure coupling, a coupling time constant of 5 ps, and the standard isothermal compressibility of water (4.5×10^{-5} bar^{-1}).

All bonds involving hydrogen atoms were constrained using the LINCS algorithm, employing default settings (fourth-order expansion and one iteration).

Constant-pH parameters

All CpHMD simulations were performed using the scalable λ-dynamics implementation by Aho et al.[24] within the GROMACS MD engine.[47,48] For each aminolipid, a single titratable site was defined, comprising a subset of atoms

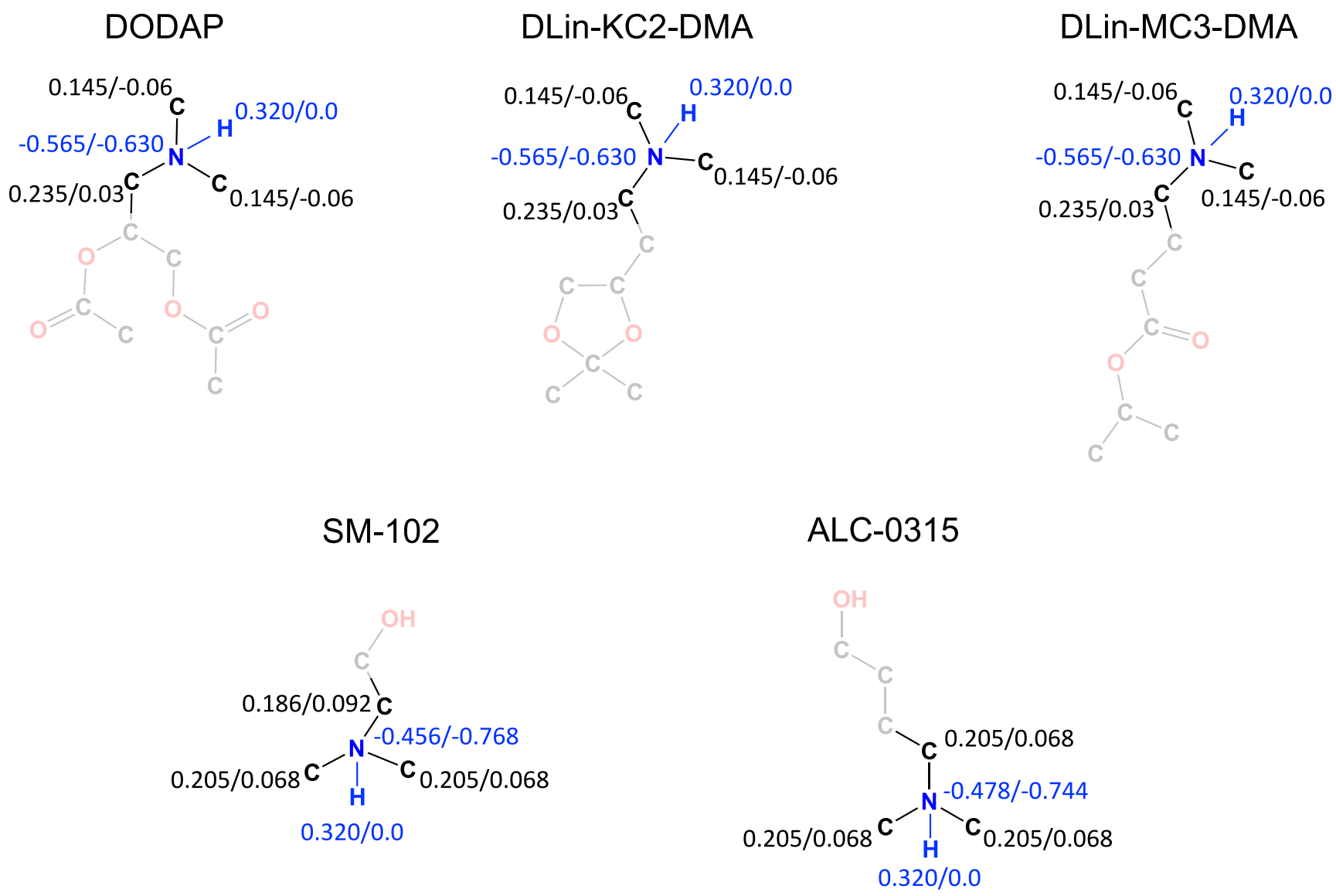

Figure 2. Titratable sites of the studied aminolipids. Headgroup structures of each aminolipid with the corresponding partial charges in the protonated and deprotonated states. Carbons are shown in black, oxygens in red, nitrogens in blue, and polar hydrogens in blue/red. Aliphatic hydrogens are omitted for clarity. Transparent atoms indicate non-titratable groups whose partial charges remain fixed.

whose partial charges were scaled according to the instantaneous protonation state. An overview of the selected titratable atoms and their partial charges is provided in Figure 2.

In the current CpHMD implementation, λ-dynamics is used to interpolate between the protonated ($\lambda_i = 0$) and deprotonated ($\lambda_i = 1$) states of titratable site i by scaling the atomic partial charges. The resulting λ-dependent electrostatic potential $\Phi(\mathbf{R}_i, \lambda)$ acting on atom i is given by

$$\Phi(\mathbf{R}_i, \lambda) \;=\; \sum_{j=1}^{n_{\text{rest}}} \frac{q_j}{4\pi\varepsilon_0 r_{ij}} + \sum_{m=1}^{N_{\text{sites}}} \sum_{j=1}^{n_m} \frac{(1-\lambda_m)q_j^A + \lambda_m q_j^B}{4\pi\varepsilon_0 r_{ij}}, \tag{1}$$

where n_{rest} denotes the number of atoms not belonging to any titratable site, N_{sites} is the total number of titratable sites, n_m is the number of atoms in titratable site m, and q_j^A and q_j^B are the partial charges of atom j in the protonated and deprotonated states, respectively. Through this coupling, the protonation state of each aminolipid becomes dependent on its local environment, with the λ coordinate evolving dynamically according to Newton's equations of motion.

Accurate CpHMD simulations require estimation of the protonation free-energy term $V^{\text{MM}}(\lambda)$ for each titratable site.[24,49,50] Parameterization followed the workflow established in our previous work.[18,25] Briefly, a single aminolipid and one buffer particle were placed in a $6 \times 6 \times 6$ nm^3 simulation box containing water and 150 mM Na^+Cl^-. Thermodynamic integration (TI) was then performed to estimate $\partial V^{\text{MM}}/\partial\lambda$ at fixed values of the λ coordinate. For each aminolipid, 25 independent TI simulations were carried out with λ values spanning the interval $[-0.10, 1.10]$ in increments of 0.05. Simulation lengths depended on aminolipid size, with larger aminolipids (ALC-0315 and SM-102) requiring longer trajectories to achieve convergence. A detailed overview of TI window lengths is provided in Table S1.

Following the recommendations of Buslaev et al.,[49] the resulting $\partial V^{\text{MM}}/\partial\lambda$ profiles were fitted using high-order polynomials. A ninth-order polynomial was employed for ALC-0315 and SM-102, while an eighth-order polynomial was sufficient for the remaining aminolipids. In addition to V^{MM}, two auxiliary potentials act on the λ coordinates: V^{pH}, which accounts for the effect of the environmental pH, and V^{bias}, which prevents divergence of λ toward unphysical values and stabilizes sampling near the physical end states ($\lambda \approx 0$ and $\lambda \approx 1$). For all simulations, the barrier height of V^{bias} was set to 7.5 kJ mol^{-1}.

Adjustment of buffer particles

The initial number of buffer particles was set to 97, corresponding to the maximum number of buffer particles necessary in the simulations ($2N_{\text{sites}} + 1$, where N_{sites} is the number of titratable sites). This choice ensures charge compensation for the largest possible fluctuation in membrane charge, corresponding to complete deprotonation of all aminolipids in the system. Although buffer particles are designed to minimize interference with the system,[49] their number should be reduced to the required minimum.

After 500 ns of simulation, the dominant changes in membrane protonation had occurred, resulting in a substantial redistribution of charge to the buffer particles. At this stage, the simulations were restarted to reduce the number of buffer particles and to reset their partial charges to zero (i.e., $\lambda^{\text{Buffer}} = 0.5$). The required number of buffer particles was determined based on the charge fluctuations and drift observed during the preceding trajectory segment. The

number of counterions was adjusted accordingly to maintain overall charge neutrality. Buffer particles located closest to the membrane and counterions farthest from the membrane were preferentially removed. Protonation states of all titratable sites in the final frame of the previous run were used to initialize the restarted simulations.

To ensure numerical stability after particle removal, a short energy minimization of up to 30 iterations was performed prior to resuming the simulations. This procedure was repeated once more after an additional 500 ns of simulation time.

Validation

For each aminolipid, the fitted polynomial coefficients were validated using two complementary approaches. (1) A CpHMD simulation of a single aminolipid in solution was performed without the V^{pH} contribution and with the barrier height set to 0.0 kJ mol^{-1}. Under these conditions, the λ coordinate is expected to sample the interval [0, 1] approximately uniformly. (2) Titration simulations at infinite dilution were carried out, in which a single aminolipid was simulated over a series of pH values controlled by V^{pH}, using a barrier height of 7.5 kJ mol^{-1}. From the fraction of simulation frames in which the aminolipid was deprotonated at each pH value, the intrinsic $\mathrm{pK_a}$ was determined. Agreement between the numerically obtained intrinsic $\mathrm{pK_a}$ and the value specified in the simulation input (.mdp file) confirmed that the fitted polynomial coefficients accurately reproduce the protonation free-energy profile.

Equilibration and error estimation

Estimating observables from MD trajectories as time averages without significant drift is a common challenge. Given the large number of simulations performed in this study, an automated and robust procedure was implemented to assess equilibration and convergence for any averaged observable of interest (e.g., S^{deprot}, S^{mix}, and thickness).

To quantify potential drift, a linear model was first fitted to the observable over the full trajectory interval [0, T]. Subsequently, progressively larger initial portions of the trajectory were discarded, and the linear fit was recomputed for each truncated interval [τ_i, T]:

$$y(t) \;=\; \beta_i\, t + \epsilon_i, \qquad t \in [\tau_i, T]. \tag{2}$$

where β_i and ϵ_i denote the slope and intercept of the linear regression, respectively. For each truncated interval, the drift was defined as the difference between the fitted model values at the endpoints:

$$\mathrm{Drift}_i \;=\; y(T) \,-\, y(\tau_i). \tag{3}$$

To obtain a dimensionless and objective measure of drift, this quantity was normalized by the empirical standard deviation σ_i of the observable within the same interval:

$$O_i \;=\; \left|\frac{\mathrm{Drift}_i}{\sigma_i}\right|. \tag{4}$$

An observable was considered equilibrated once $O_i < 1$ for the first time, indicating that the residual drift was smaller than the intrinsic fluctuations of the signal. Standard errors of the mean were then estimated from the equilibrated portion of the trajectory using block averaging. The resulting error estimates were fitted to either a single- or double-exponential model following the approach of Hess.[51] In cases where the automated workflow yielded insufficient equilibration times, the starting points were adjusted manually. The final assigned equilibration times are shown as vertical lines in Figures S53 – S66, S73 – S84, S102, S104, and S105.

Protonation-state analysis

Protonation states of individual aminolipids were assigned following the recommendations of Aho et al.[24] For lipid i in simulation frame j, the lipid was classified as protonated if $\lambda_j^i < 0.2$ and as deprotonated if $\lambda_j^i > 0.8$. Frames in which λ_j^i fell within the intermediate range were excluded from protonation-state analysis for that lipid. For each simulation frame, the fraction of deprotonated aminolipids was computed as

$$\mathrm{S}_j^{\mathrm{deprot}} \;=\; \frac{\mathrm{N}_j^{\mathrm{deprot}}}{\mathrm{N}_j^{\mathrm{deprot}} + \mathrm{N}_j^{\mathrm{prot}}}, \tag{5}$$

where $\mathrm{N}_j^{\mathrm{deprot}}$ and $\mathrm{N}_j^{\mathrm{prot}}$ denote the numbers of deprotonated and protonated aminolipids, respectively. For simulations at infinite dilution containing a single aminolipid, N^{deprot} and N^{prot} correspond to the number of frames in which the aminolipid was classified as deprotonated or protonated, respectively. The λ-dynamics approach used in CpHMD simulations requires sampling of predominantly physical states ($\lambda_i \approx 0.0$ or $\lambda_i \approx 1.0$), while minimizing occupancy of the transition region ($0.2 \le \lambda \le 0.8$) through the application of an additional biasing potential, $V^{\mathrm{bias}}(\lambda)$. Figures S51, S52, and S103 demonstrate that this criterion was satisfied in all simulations performed in this study.

Apparent $\mathrm{pK_a}$ values were obtained by fitting the pH-dependent mean fraction of deprotonated aminolipids to the generalized Henderson-Hasselbalch equation,

$$h(\mathrm{pH}; n, pK_a) \;=\; \langle S^{\mathrm{deprot}} \rangle^{\mathrm{pH}} \;=\; \frac{1}{1 + 10^{n(pKa\,-\,\mathrm{pH})}}, \tag{6}$$

where $\langle S^{\mathrm{deprot}} \rangle^{\mathrm{pH}}$ denotes the time-averaged fraction of deprotonated aminolipids at a given pH. The parameter n describes the cooperativity of the transition, and $\mathrm{pK_a}$ corresponds to the pH at which $\langle S_j^{\mathrm{deprot}} \rangle^{\mathrm{pH}} \;=\; 0.5$.

For intrinsic $\mathrm{pK_a}$ estimation, n was fixed to 1.0 during least-squares fitting. For apparent $\mathrm{pK_a}$ values, an iterative

reweighting least-squares scheme was employed to improve fit robustness. Initial weights were set to the inverse variance of the mean, $w^{\mathrm{pH}} = 1/(\sigma^{\mathrm{pH}})^2$, and were iteratively updated by multiplying them with the derivative of Equation 6,

$$\frac{\mathrm{d}h(\mathrm{pH}; n, \mathrm{pK_a})}{\mathrm{d\,pH}} = \ln(10) \cdot n \cdot h(\mathrm{pH}; n, \mathrm{pK_a})$$

$$[1 - h(\mathrm{pH}; n, \mathrm{pK_a})]. \tag{7}$$

This procedure increases the relative weight of data points in the transition region, where statistical uncertainty is highest, while retaining contributions from well-sampled low- and high-pH regimes. Standard errors of the mean σ^{pH} were clipped to a minimum value of 10^{-4}. The iterative scheme was terminated when the Euclidean norm of the difference between successive parameter vectors fell below 10^{-8}.

Confidence intervals for fitted parameters were estimated using parametric bootstrapping.[52,53] At each pH value, S^{deprot} was assumed to follow a normal distribution,

$$S^{\mathrm{deprot}} \sim \mathcal{N}\left(\langle S^{\mathrm{deprot}} \rangle^{\mathrm{pH}}, (\sigma^{\mathrm{pH}})^2\right), \tag{8}$$

where $\langle S^{\mathrm{deprot}} \rangle^{\mathrm{pH}}$ denotes the mean over the equilibrated trajectory segment and σ^{pH} is the corresponding standard error of the mean obtained from block averaging. For each condition, 50,000 bootstrap samples were generated. Reported $\mathrm{pK_a}$ values correspond to the mean of the bootstrap distribution. The 95% confidence intervals were defined by the 2.5th and 97.5th percentiles of the bootstrap distribution.

Periodic center-of-mass definition

Each trajectory was post-processed by centering the membrane in the middle of the simulation box and reconstructing molecules fragmented across periodic boundary conditions (PBCs). However, this workflow may occasionally result in the membrane being split across the periodic boundary in the z direction. To avoid potential bias in the membrane-thickness calculation and in density profiles along the z axis, a periodic trigonometric approach similar to Bai et al.[54] was applied to determine the average position of the phosphorus atoms in the helper lipids. First, the z coordinates z_i were wrapped back into the primary unit cell. The coordinates were then mapped onto the unit circle according to

$$\theta_i = \frac{2\pi z_i}{L_z}, \tag{9}$$

where L_z denotes the box length in the z direction. The averages of the sine and cosine components over all N phosphorus atoms were computed as

$$\langle \sin\theta \rangle = \frac{1}{N}\sum_{i=1}^{N} \sin(\theta_i), \tag{10}$$

$$\langle \cos\theta \rangle = \frac{1}{N}\sum_{i=1}^{N} \cos(\theta_i). \tag{11}$$

The mean z position was then reconstructed using the two-argument arctangent function:

$$\langle z^{\mathrm{COM}} \rangle = \frac{L_z}{2\pi}\,\mathrm{atan2}(\langle \sin\theta \rangle, \langle \cos\theta \rangle). \tag{12}$$

Finally, $\langle z^{\mathrm{COM}} \rangle$ was mapped back into the simulation box. At all stages, it was ensured that the average position of the upper leaflet remained greater than that of the lower leaflet. The average z positions per frame for each leaflet are shown in Figures S69 – S72.

RESULTS

This study examines how membrane composition modulates the protonation equilibria of embedded aminolipids and, conversely, how pH-dependent aminolipid protonation reshapes membrane organization. Symmetric lipid bilayers were composed of 20% aminolipids, 60% or 40% of either fully saturated DSPC or diunsaturated DOPC as helper lipid, and 20% or 40% cholesterol. These compositions were selected to capture key physicochemical features of LNP-solvent interfaces, including the high cholesterol and phospholipid content of the LNP shell.[18] At the same time, they form stable bilayers across the investigated pH range (pH 3–11), allowing us to dissect how helper-lipid saturation and cholesterol concentration affect aminolipid protonation and interfacial membrane organization.

Each system contained 240 lipid and cholesterol molecules at a hydration level of approximately 50 water molecules per lipid (or cholesterol molecule, detailed compositions are provided in Tables S2 – S4). System neutrality was maintained using a pH-dependent number of chloride ions and buffer particles (see Tables S1 – S4). The total aggregate simulation time amounted to approximately 0.29 ms. Details of aminolipid parameterization, system setup, and analysis are provided in the materials and methods section.

$\mathrm{pK_a}$ shifts of aminolipids in ternary membranes

The pH dependence of aminolipid protonation was quantified by computing the mean fraction of deprotonated molecules, S^{deprot} (Figure 3), over a pH range from 3 to 11. For all aminolipids and membrane compositions, the resulting titration curves were well described by a generalized Henderson-Hasselbalch equation. Fitting the averaged S^{deprot} values yielded the $\mathrm{pK}_\mathrm{a}^{app}$ values of the aminolipids, as well as the associated cooperativity parameters n (Table 1).

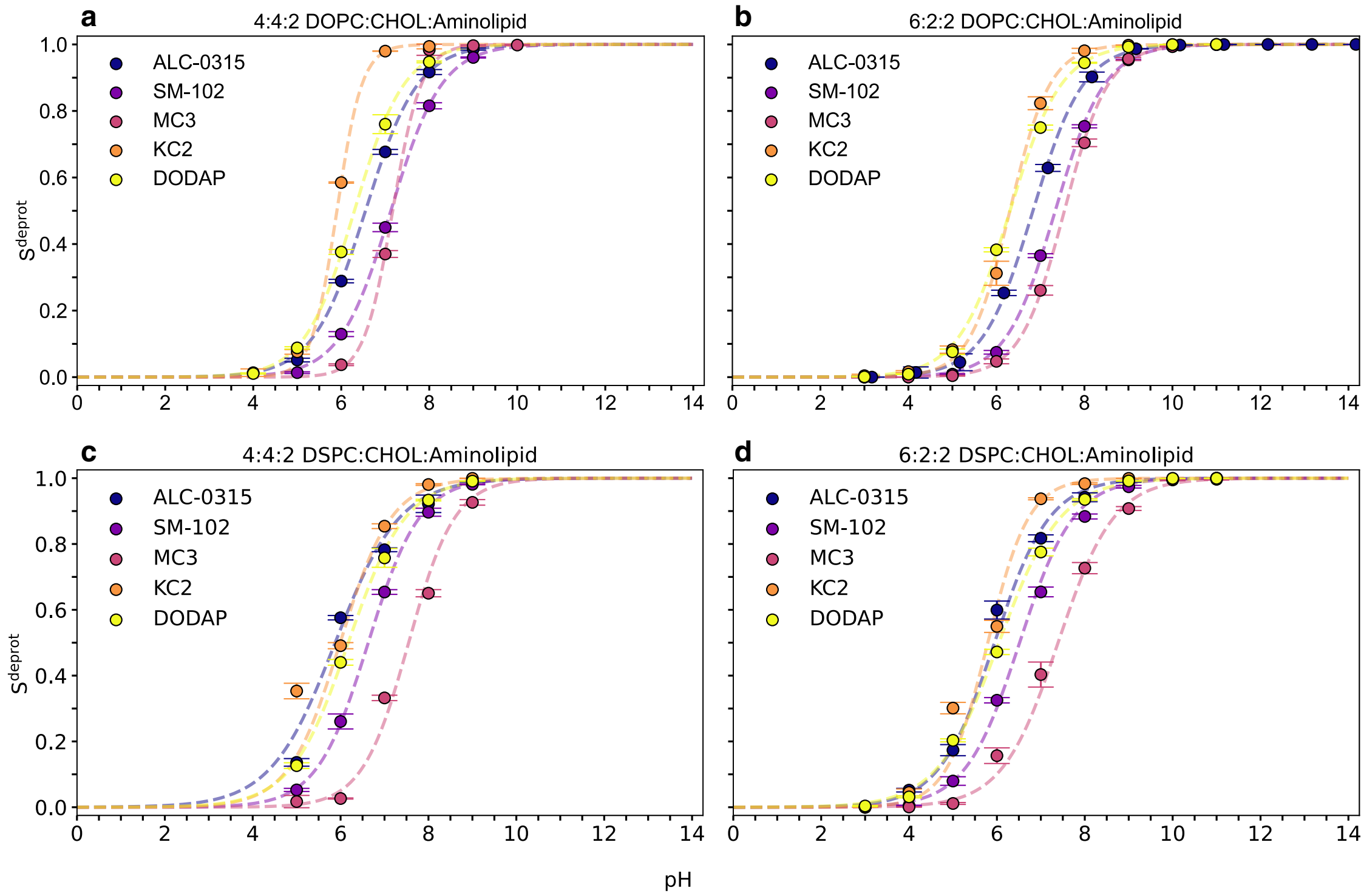

Figure 3. Titration curves of aminolipids in different membrane compositions. Titration curves are shown for DOPC-based (*top*) and DSPC-based (*bottom*) membranes containing (a and c) 40 mol % cholesterol and (b and d) 20 mol % cholesterol. The aminolipid content was fixed at 20 mol % in all systems. Error bars denote the standard error of the mean, obtained by block averaging (see materials and methods). The data were fitted using the generalized Henderson-Hasselbalch equation (see materials and methods), and the resulting fit parameters are summarized in Table 1. The values of S^{deprot} as a function of time are shown in Figures S53 – S56. CHOL, cholesterol.

Across all systems, the pK_a^{app} values were consistently lower than the intrinsic pK_a^{AL} values of the corresponding aminolipids at infinite dilution (Figures 4a and 4b), indicating a destabilization of the protonated state within membrane environments. The magnitude of the pK_a shift depended on both membrane composition and aminolipid structure. The largest shift was observed for KC2 in DSPC-based membranes at a 60:20:20 DSPC:cholesterol:KC2 composition, with $\Delta pK_a = 3.55$, corresponding to a protonation free-energy change of $\Delta G_a = \ln 10 \cdot \Delta pK_a \cdot RT \approx 21.1$ kJ mol^{-1}. In contrast, the smallest shift was found for DODAP ($\Delta pK_a = 1.35$) in a 40:40:20 DOPC:cholesterol:DODAP membrane (Figures 4a and 3b). The magnitude of the observed ΔpK_a values followed the trend KC2 > ALC-0315 > MC3 > SM-102 > DODAP in DOPC membranes, whereas, for DSPC membranes, the trend was KC2 > ALC-0315 > SM-102 > MC3 > DODAP.

Table 1. Apparent pK_a and cooperativity (*n*) values estimated from fits to the averaged degree of aminolipid deprotonation as a function of pH

Composition HL:CHL:AL	AL	DOPC pK_a	DOPC *n*	DSPC pK_a	DSPC *n*
4:4:2	ALC-0315	6.55 ± 0.03	0.73 ± 0.03	5.88 ± 0.03	0.59 ± 0.03
4:4:2	SM-102	7.13 ± 0.04	0.75 ± 0.02	6.63 ± 0.03	0.74 ± 0.03
4:4:2	MC3	7.17 ± 0.03	1.33 ± 0.11	7.55 ± 0.05	0.82 ± 0.03
4:4:2	KC2	5.90 ± 0.01	1.52 ± 0.03	5.99 ± 0.05	0.74 ± 0.04
4:4:2	DODAP	6.30 ± 0.03	0.77 ± 0.02	6.18 ± 0.04	0.67 ± 0.01
6:2:2	ALC-0315	6.83 ± 0.04	0.75 ± 0.02	5.94 ± 0.07	0.69 ± 0.03
6:2:2	SM-102	7.34 ± 0.02	0.77 ± 0.01	6.52 ± 0.05	0.70 ± 0.02
6:2:2	MC3	7.54 ± 0.04	0.87 ± 0.05	7.39 ± 0.08	0.68 ± 0.02
6:2:2	KC2	6.31 ± 0.10	0.95 ± 0.17	5.79 ± 0.06	0.93 ± 0.03
6:2:2	DODAP	6.31 ± 0.02	0.74 ± 0.01	6.05 ± 0.03	0.62 ± 0.01

Errors denote the 95% confidence interval calculated via parametric bootstrapping (see materials and methods). HL, helper lipid; CHL, cholesterol; AL, aminolipid.

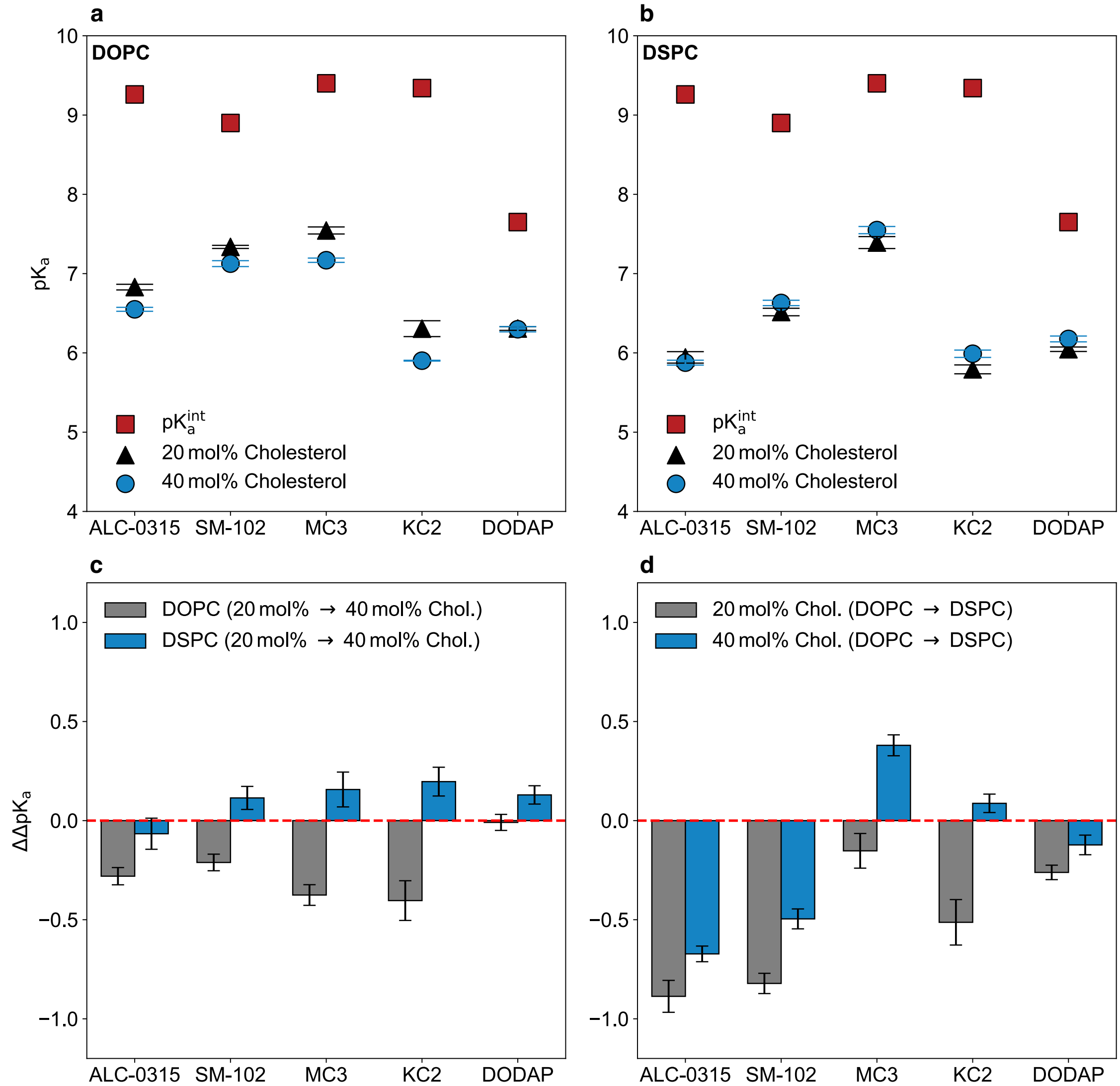

Figure 4. Influence of membrane composition on aminolipid pK_a. Comparison of pK_a^{app} values for (a) DOPC- and (b) DSPC-based membranes containing 20 mol % or 40 mol % cholesterol with the intrinsic pK_a^{AL} values at infinite dilution. (c and d) Differential pK_a shifts ($\Delta\Delta pK_a$), where $\Delta pK_a = pK_a{}^{app} - pK_a{}^{AL}$ and $\Delta\Delta pK_a$ denotes the difference between two ΔpK_a values. Specifically, (c) quantifies the effect of cholesterol content (20 mol % → 40 mol %) for the different aminolipids, shown separately for DOPC- and DSPC-based membranes, whereas (d) reports the effect of helper-lipid identity (DOPC → DSPC) at fixed cholesterol fractions (20mol % and 40 mol %). Error bars indicate 95% confidence intervals: for (a) and (b), intervals were obtained by bootstrap resampling of the titration fits (see materials and methods); for (c) and (d), confidence intervals were calculated by propagation of the bootstrap-derived standard errors, assuming independent estimates.

The influence of cholesterol on the pK_a shift differed between unsaturated (DOPC) and saturated (DSPC) membranes. In DOPC systems, increasing cholesterol content led to larger pK_a shifts, whereas, in DSPC systems, the effect was weaker and, for all aminolipids except ALC-0315, reversed (Figure 4c). For most compositions, replacing DOPC with DSPC resulted in an increased pK_a shift (more negative, Figure 4d), an effect that was particularly pronounced for branched aminolipids, with increases of approximately 0.5–1 pK_a unit. Only for the polyunsaturated aminolipids KC2 and MC3 at high cholesterol content was a slight increase in the apparent pK_a observed upon DOPC → DSPC substitution. Notably, these aminolipids also exhibited the strongest sensitivity to cholesterol concentration (Figure 4c).

pH-driven (de-)mixing of ternary membranes containing aminolipids

The response of the ternary membranes to changes in environmental pH depends strongly on both membrane composition and aminolipid structure. pH induced changes in membrane organization were quantified using the normalized average conditional mixing entropy, S^{mix} (Figure 5).[55]

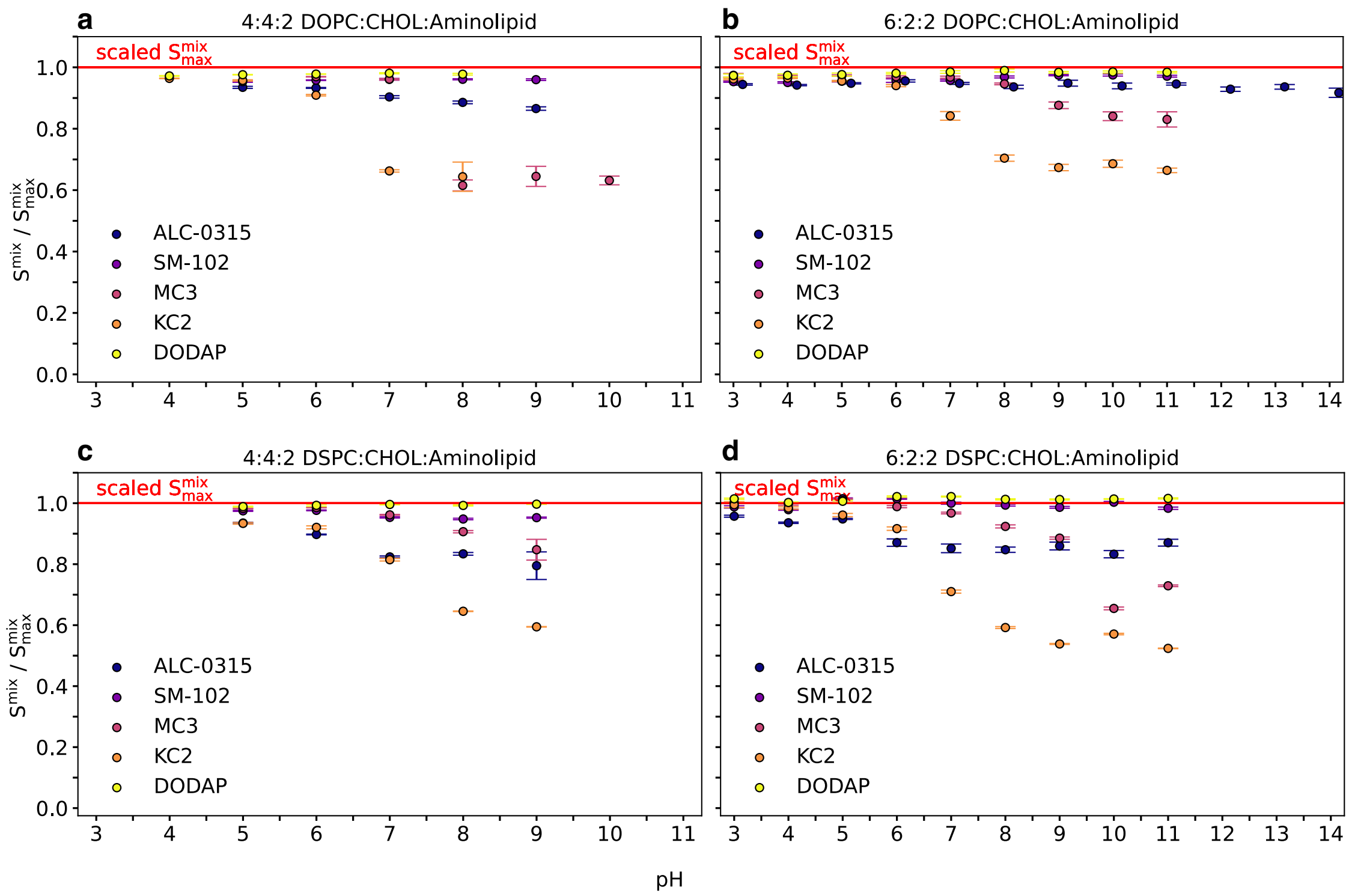

Figure 5. Standardized mixing entropy (S^{mix}) as a function of pH. Mean values of S^{mix} are shown for DOPC-based (*top*) and DSPC-based (*bottom*) membranes containing (a and c) 40 mol % cholesterol and (b and d) 20 mol % cholesterol. Conditional mixing entropies were calculated following Brandani et al.,[55] using a 1-nm cutoff to define neighboring lipids. Contacts were defined based on the distance between reference atoms in the headgroups of the target lipids: the titratable nitrogen for aminolipids, the oxygen atom for cholesterol, and the sn-2 carbon atom for phospholipids. Error bars indicate the standard error of the mean, obtained by block averaging (see materials and methods). The values of S^{mix} as a function of time are shown in Figures S57 – S60. CHOL, cholesterol.

For all compositions, S^{mix} reached a maximum at acidic pH (pH 3), corresponding to homogeneously mixed bilayers containing phospholipids, aminolipids, and cholesterol. With increasing pH, several membrane compositions exhibited pronounced de-mixing ($S^{mix} < 1$), resulting in the formation of compositionally distinct phases.

Neighbor analysis using a 1 nm cutoff (Figure 6) showed that aminolipid deprotonation at higher pH increases aminolipid-aminolipid contacts, while contacts with phospholipids are markedly reduced. The aminolipid-phospholipid interactions are primarily mediated by hydrogen bonds between the aminolipid titratable sites and phospholipid phosphate groups (Figures 7 and S67). Upon increasing pH, these interactions are either redistributed toward cholesterol and neighboring aminolipids, as observed for ALC-0315, SM-102, and DODAP, or are largely lost, as observed for DLin-KC2-DMA and DLin-MC3-DMA.

The branched aminolipids ALC-0315 and SM-102 showed pronounced hydrogen-bonding capacity (Figure 7), consistent with their two ester linkages and hydroxyl-containing headgroups. For ALC-0315, the total number of intermolecular hydrogen bonds remained nearly constant across the investigated pH range, whereas the fraction of ALC-0315–ALC-0315 hydrogen bonds increased with pH. DODAP retained hydrogen bonding with cholesterol through its ester moieties even at elevated pH. In contrast, DLin-KC2-DMA showed the weakest hydrogen-bonding propensity, in line with the low polarity of its 1,3-dioxolane headgroup (Figure 1).

The overall tendency toward segregation followed the order KC2 (highest segregation) > MC3 > ALC-0315 > SM-102 > DODAP (lowest segregation) and was most pronounced in membranes containing higher fractions of DSPC. For the polyunsaturated aminolipids KC2 and MC3, increasing pH-induced aminolipid migration into the hydrophobic membrane core, where they localized between the phospholipid leaflets (Figure 8), consistent with previous LNP-mimetic studies.[2,57,58] In contrast, branched aminolipids (ALC-0315 and SM-102) predominantly underwent *lateral* segregation from surrounding phospholipids, an effect that was strongest for the four-tailed ALC-0315 in DSPC-containing membranes (Figures 9a and 9b). Migration of aminolipids toward the membrane interior has also been reported for ALC-0315 and SM-102 in standard LNP formulations.[2,18,59] Over the investigated timescale of 1 μs, DODAP showed little to no segregation under any conditions, consistent with its high mixing entropy (Figure 4). Notably, across all

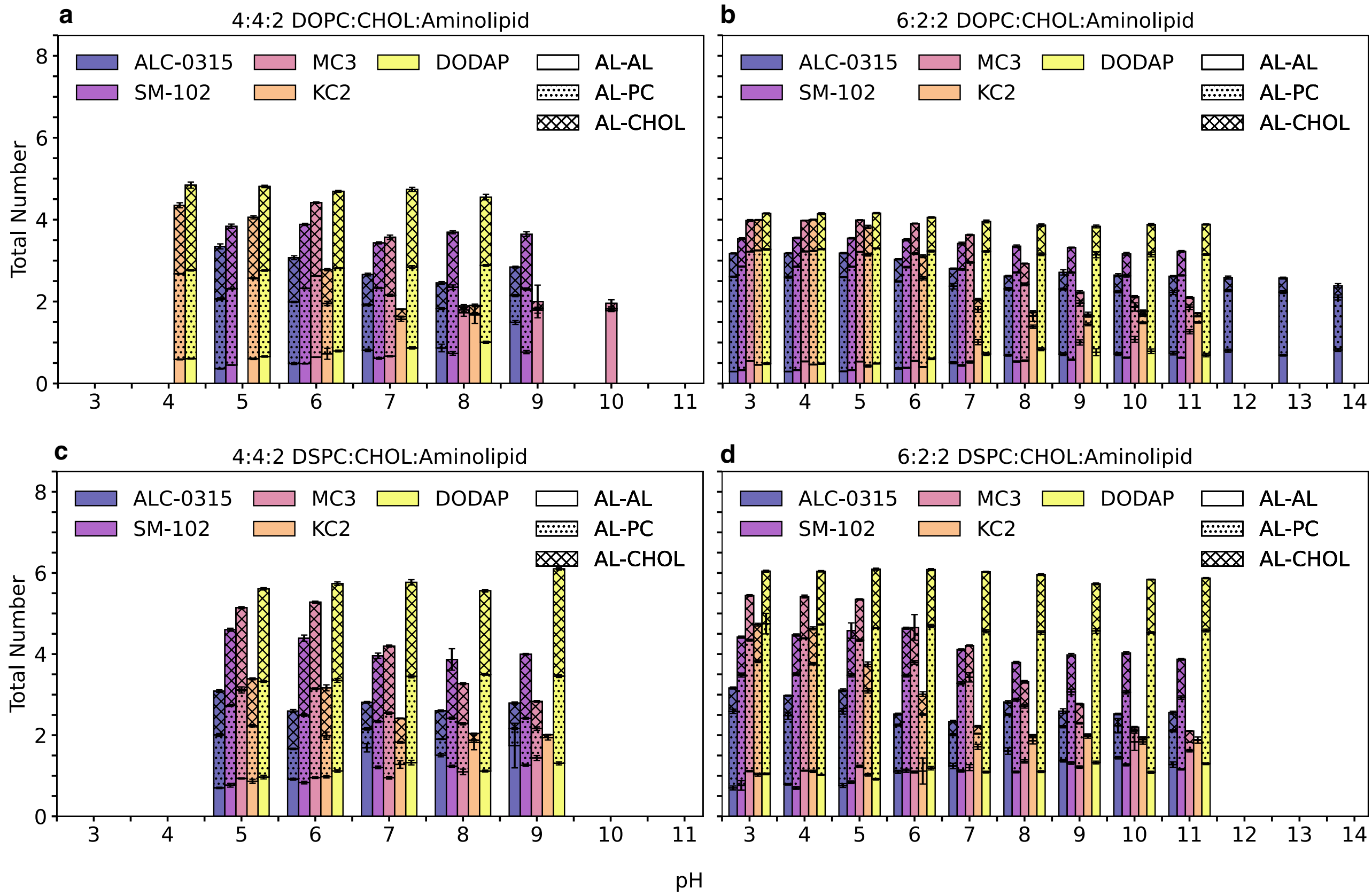

Figure 6. Local pH-dependent lipid environment of aminolipids. Average number of neighboring lipids within 1 nm of aminolipids as a function of pH for DOPC-based (*top*) and DSPC-based (*bottom*) membranes containing (a and c) 40 mol % cholesterol and (b and d) 20 mol % cholesterol. Contacts were defined based on the distance between reference atoms in the headgroups of the target lipids: the titratable nitrogen for aminolipids, the oxygen atom for cholesterol, and the sn-2 carbon atom for phospholipids. Error bars indicate the standard error of the mean, obtained by block averaging (see materials and methods). The average number of neighboring lipids per aminolipid as a function of time are shown in Figures S61 – S66. AL, aminolipid; PC, phosphatidylcholine; CHOL, cholesterol.

investigated membrane compositions, only a minor fraction of cholesterol (∼1%–6%) migrated into the membrane core with increasing environmental pH, as shown in Figure S68. Representative snapshots of all systems after 2 μs (or 1 μs) are shown in Figures S7 – S46.

Phase separation was accompanied by pronounced changes in membrane structure. Average bilayer thickness profiles (Figure 10) first confirm that DOPC-containing membranes (Figures 10a and 10b) are generally thinner than their DSPC counterparts (Figures 10c and 9D), reflecting the presence of di-saturated acyl chains. More importantly, distinct trends emerged between acidic and basic pH regimes. At low pH (<6), bilayer thickness followed the order DODAP (thickest) > KC2/MC3 > SM-102 > ALC-0315 (thinnest), whereas, at high pH, the ranking shifted to KC2 (thickest) > MC3 > DODAP > ALC-0315/SM-102 (thinnest). These changes reflect pH-dependent aminolipid relocation within the membrane, which directly modulates bilayer thickness.

Notably, KC2- and MC3-containing membranes thickened by approximately 1.5 nm upon aminolipid migration toward the membrane core (Figure 10). This transition occurred at lower pH for KC2 than for MC3 and was observed across all tested compositions, except for the 4:4:2 DSPC:cholesterol:MC3 system, where membrane thickening was minimal. In contrast, systems containing ALC-0315 or SM-102 exhibited only minor thickness changes, while DODAP-containing membranes remained essentially unaffected across the entire pH range.

Increasing pH further induced dehydration of the aminolipid titratable sites, quantified as the average number of water molecules within 0.5 nm of the N–H proton (Figure 11). MC3, KC2, and DODAP displayed high hydration at low pH, consistent with solvent-exposed titratable sites. Upon inward migration of KC2 and MC3, their hydration shells were almost completely lost, whereas DODAP remained at the membrane surface and exhibited only a modest reduction in solvation. In contrast, the titratable sites of branched aminolipids (ALC-0315 and SM-102) were less solvent exposed even at low pH, resulting in less pronounced dehydration at higher pH. Nonetheless, a clear dehydration

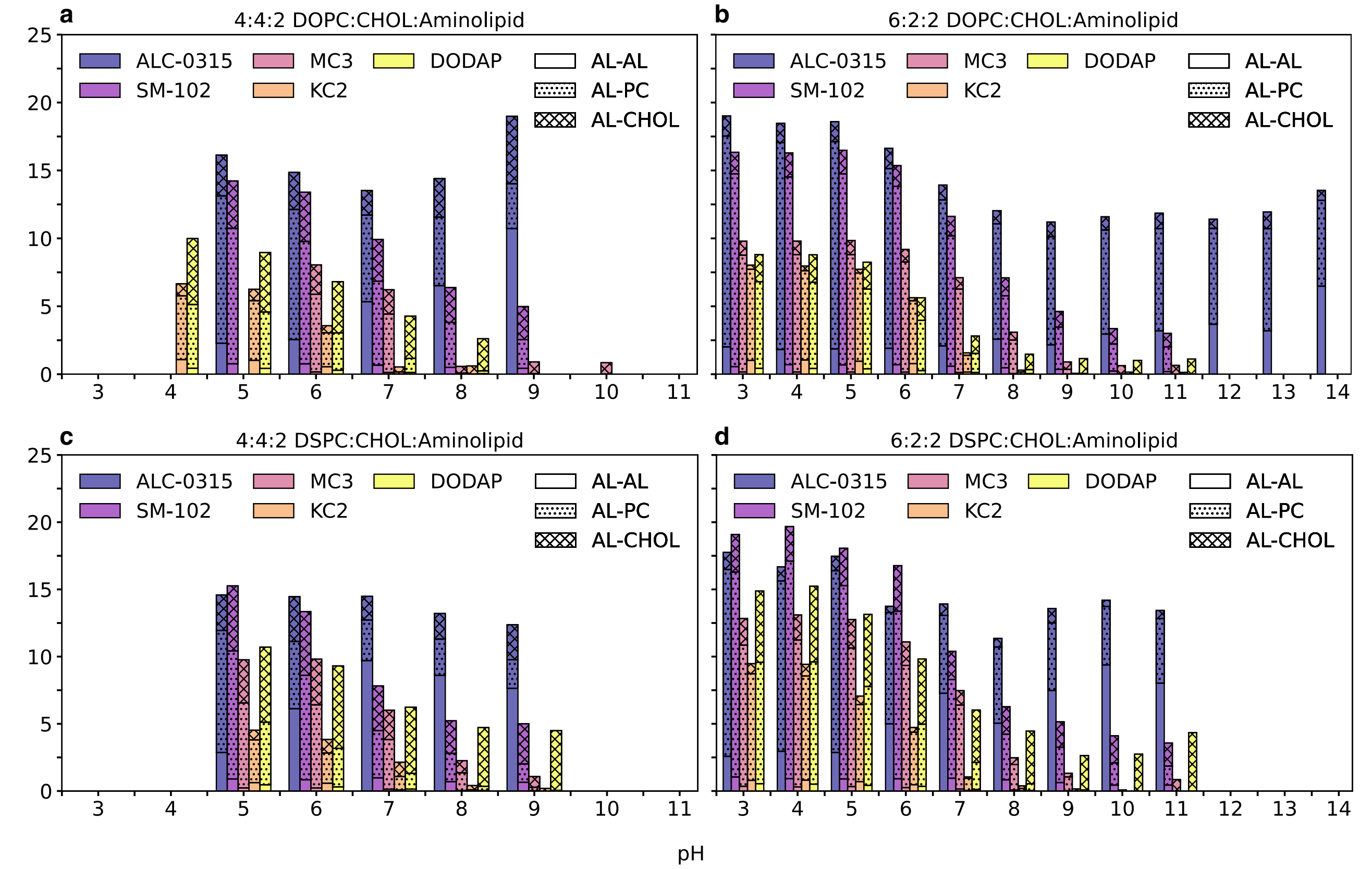

Figure 7. pH-dependent hydrogen bonding of aminolipids. Average number of hydrogen bonds between aminolipids and the surrounding membrane components as a function of pH for DOPC-based (*top*) and DSPC-based (*bottom*) membranes containing (a and c) 40 mol % cholesterol and (b and d) 20 mol % cholesterol. Hydrogen bonds were identified using the MDAnalysis library (v2.7.0) with a distance cutoff of 3.0 Å and an angle cutoff of 150°. AL, aminolipid; PC, phosphatidylcholine; CHOL, cholesterol.

trend was observed at neutral and basic pH, indicating a condensing effect associated with lateral segregation of these aminolipids.

DISCUSSION

CpHMD simulations of aminolipid-containing membranes enabled an unbiased analysis of pH-driven membrane

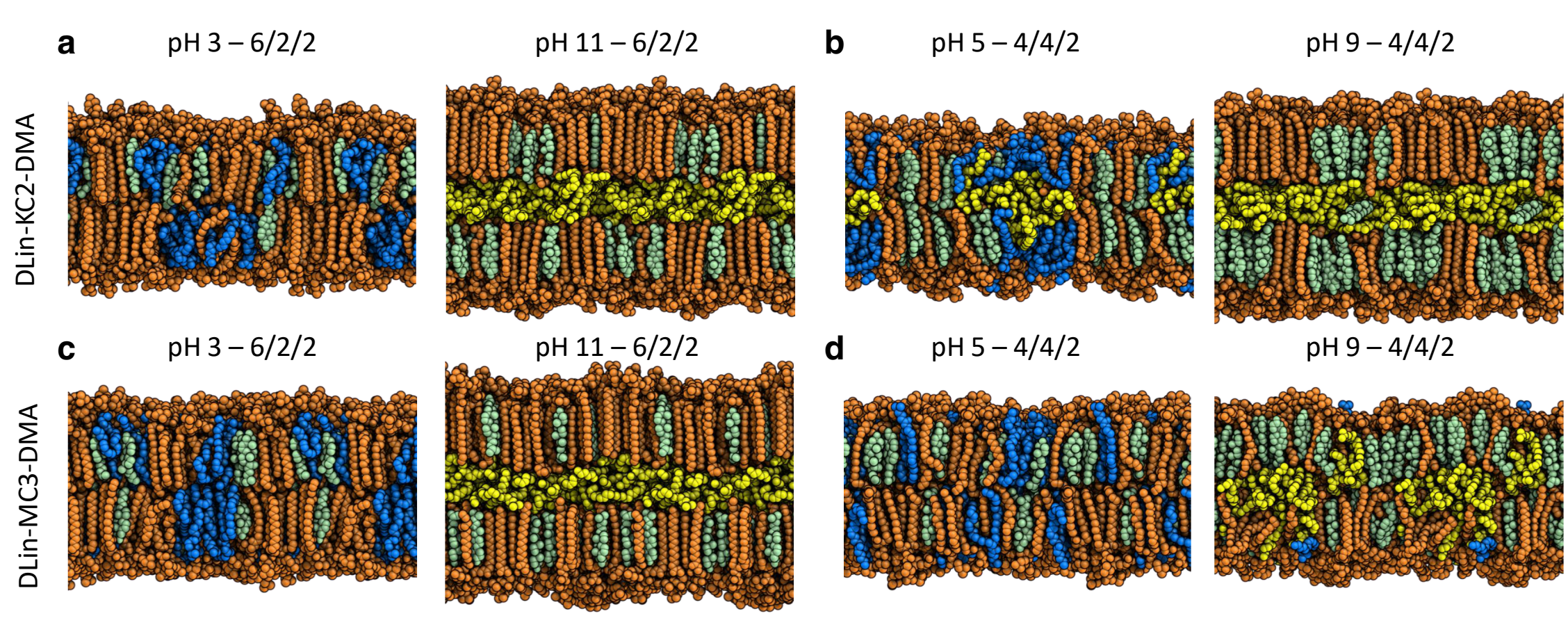

Figure 8. Side views of DSPC:cholesterol:MC3/KC2 membranes. Representative snapshots of DSPC-based membranes after 2 μs containing DLin-KC2-DMA (*top row*) or DLin-MC3-DMA (*bottom row*) at cholesterol fractions of 20 mol % (a and c) and 40 mol % (b and d). DSPC lipids are shown in orange and cholesterol in green. At low pH, protonated aminolipids (*blue*) reside at the membrane surface, whereas, at higher pH, the deprotonated aminolipids (*yellow*) migrate into the membrane core. Aminolipids without a specific protonation state ($0.2 \leq \lambda \leq 0.8$) are colored in gray. Figures were rendered with PyMOL.[56]

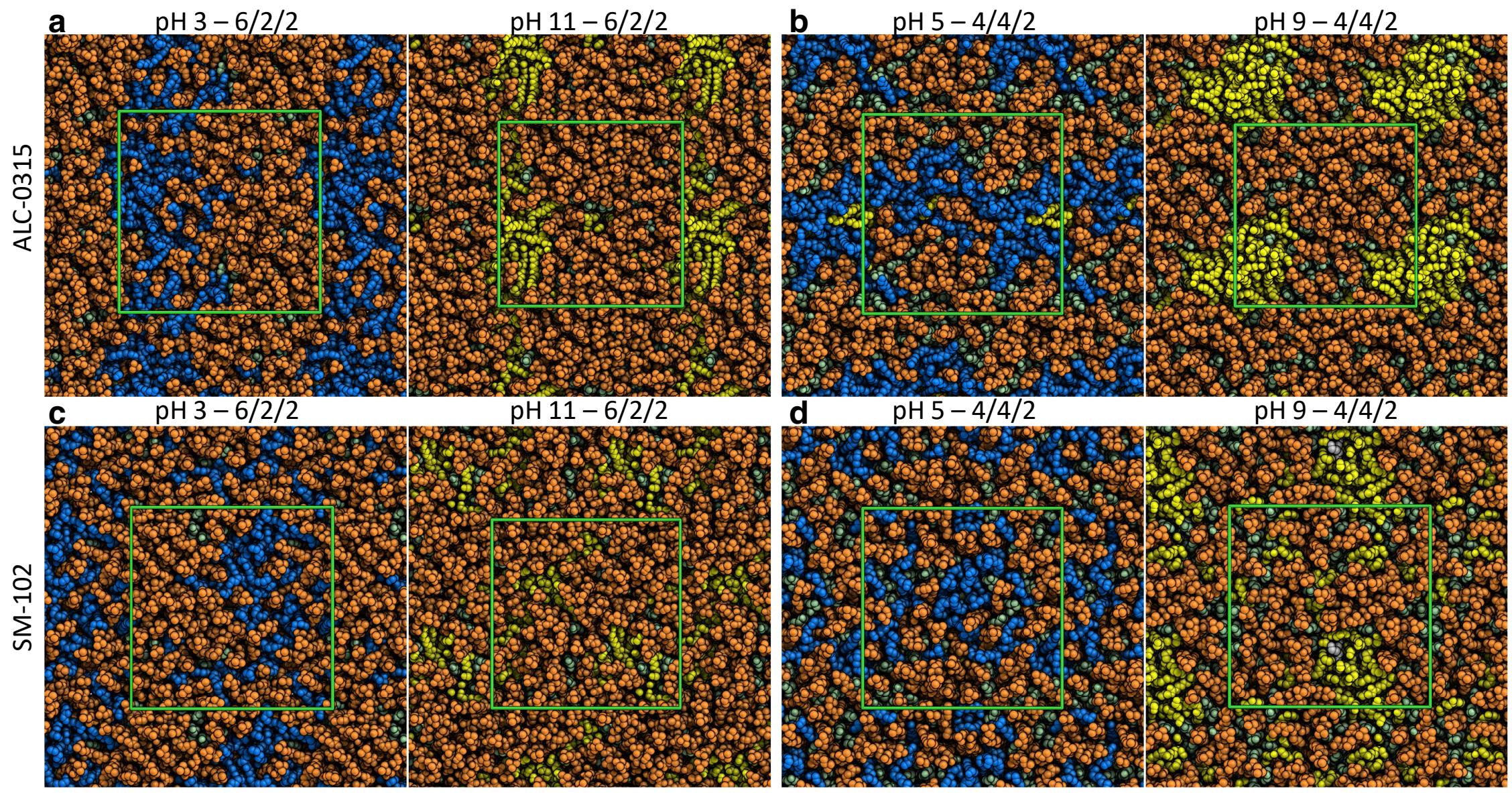

Figure 9. Top views of DSPC:cholesterol:ALC-0315/SM-102 membranes. Representative snapshots of DSPC-based membranes after 2 μs containing ALC-0315 (*top row*) or SM-102 (*bottom row*) at cholesterol fractions of 20 mol % (a and c) and 40 mol % (b and d). DSPC lipids are shown in orange and cholesterol in green. At low pH, protonated aminolipids (*blue*) are homogeneously mixed with other membrane components, whereas, at higher pH, deprotonated aminolipids (*yellow*) undergo pronounced lateral segregation. Aminolipids without a specific protonation state ($0.2 \leq \lambda \leq 0.8$) are colored in gray. Figures were rendered with PyMOL.[56]

remodeling associated with aminolipid (de)protonation. The simulations reveal a nonlinear coupling between membrane composition, membrane organization, and aminolipid protonation equilibria. A central advantage of CpHMD is that the protonation-state distribution emerges self-consistently from the coupling between local membrane environment and dynamic aminolipid protonation. In contrast to constant-protonation simulations, CpHMD does not require externally imposed protonation states or additional assumptions about the equilibrium degree of protonation.[57] This makes CpHMD well suited for analyzing apparent, environment-dependent pK_a^{app} values. Here, we show that CpHMD simulations of aminolipid-containing membranes yield well-defined titration curves, in contrast to previous work.[60]

Despite pronounced differences in headgroup chemistry, acyl-chain number, saturation, and intrinsic pK_a values ranging from 7.6 to 9.4, the apparent aminolipid pK_a values converged to a relatively narrow, physiologically relevant range of 6–7.5. These values are only slightly higher than pK_a^{LNP} estimates reported for full LNPs using TNS-binding assays or ζ-potential measurements.[20,61–63] This agreement is notable because the ternary membranes studied here differ substantially from typical LNP formulations, which often contain approximately 50:38.5:10:1.5 mol % aminolipid:cholesterol:helper-lipid:PEG-lipid.[20,64] Comparison with previous *in silico* studies[2,18] and related experimental work[65,66] suggests that the present model membranes more closely reproduce the surface-exposed lipid environment of LNPs than their bulk composition. This likely explains the close correspondence between the apparent aminolipid pK_a values obtained here and experimentally determined LNP pK_a values. This interpretation is further supported by previous MD studies,[18,67] which showed that RNA in the LNP core affects aminolipid protonation only locally, within approximately 1 nm. It is also consistent with experimental data showing that the apparent pK_a, in contrast to the isoelectric point, is relatively insensitive to RNA content when measured by TNS assays or ζ-potential measurements.[20] Together, these findings suggest that cargo has only a limited influence on aminolipid protonation equilibria at the LNP surface. This is particularly relevant because the lipid-solvent interface governs key *in vivo* processes, including protein-corona formation and endosomal membrane fusion. Modeling the pH responsiveness of this interface therefore provides mechanistic insight into LNP activity. At the same time, atomistic MD simulations cannot fully capture the complexity of biological environments. Processes such as PEG shedding,[68] anti-PEG immune responses,[69] aminolipid metabolism,[70,71] endosomal maturation,[72] protein binding,[66] inflammation exacerbation,[73] and organ tropism[74] remain beyond the scope of current atomistic simulations. Direct quantitative relationships between the simplified LNP model systems studied here and endosomal escape efficiency should therefore be interpreted with appropriate caution.

Aminolipid structure as determinant of pK_a shifts

The distinct pK_a shifts observed for the five aminolipids can be rationalized by differences in headgroup polarity,

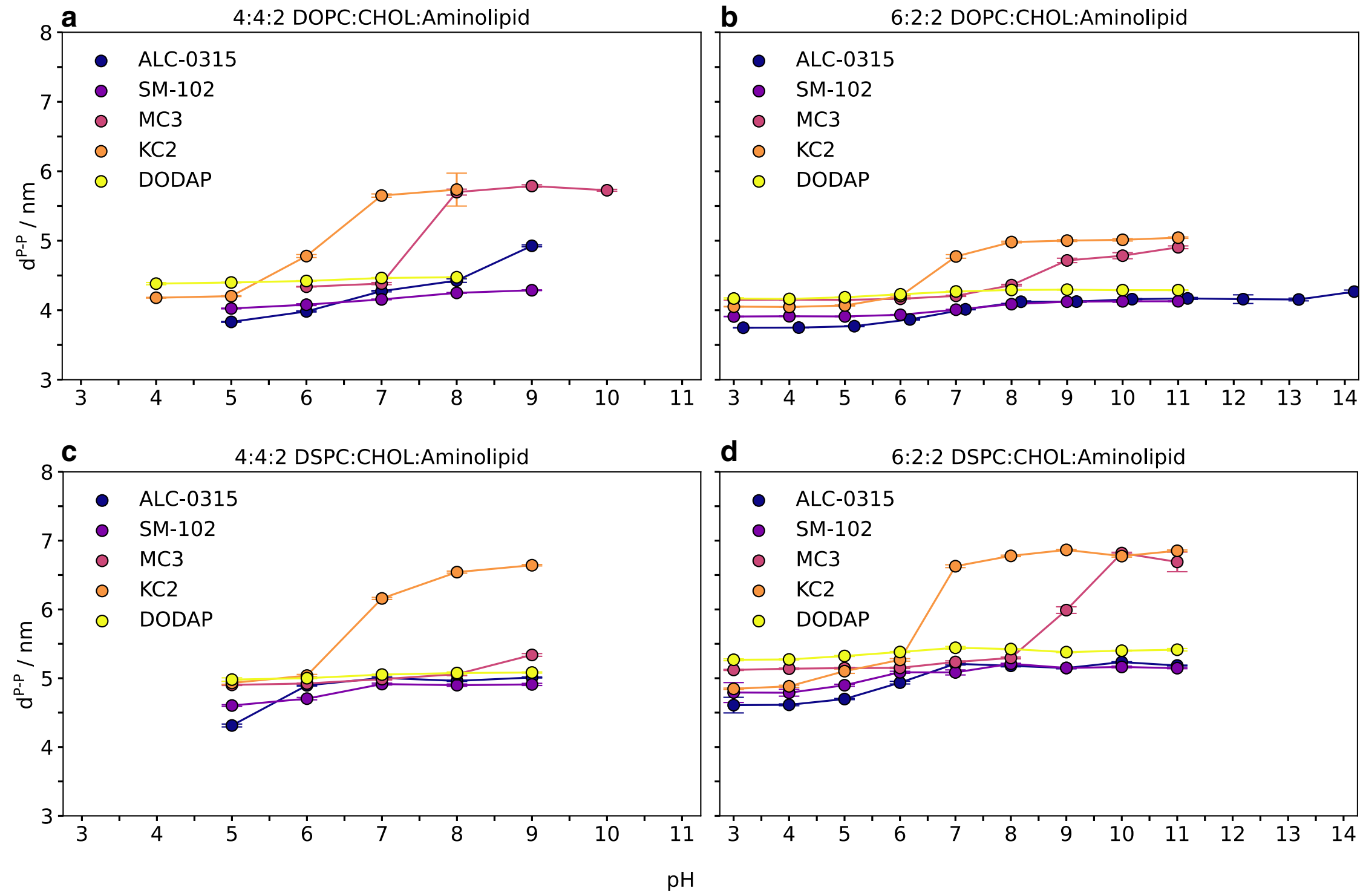

Figure 10. pH-dependent membrane thickness of aminolipid-containing membranes. Membrane thickness as a function of pH for DOPC-based (*top*) and DSPC-based (*bottom*) membranes containing (a and c) 40 mol % cholesterol and (b and d) 20 mol % cholesterol. Thickness was defined as the distance between the average z positions of the phosphorus atoms in the upper and lower membrane leaflets. Average z positions were computed using a periodic trigonometric approach (see materials and methods). Error bars indicate the standard error of the mean, obtained by block averaging (see materials and methods). The values for the thickness as a function of time are shown in Figures S73 – S76. CHOL, cholesterol.

hydration, hydrogen-bonding capacity, and acyl-chain mismatch with the surrounding membrane. DLin-KC2-DMA and ALC-0315 exhibited the largest pK_a shifts across the investigated compositions, followed by DLin-MC3-DMA and SM-102. In DSPC-containing membranes, SM-102 showed larger shifts than DLin-MC3-DMA, whereas the opposite trend was observed in DOPC-containing membranes. DODAP consistently displayed the smallest pK_a shifts (Figure 4).

DLin-KC2-DMA has the most apolar headgroup among the aminolipids studied here because of its 1,3-dioxolane moiety, which limits tight hydrogen-bond formation (Figure 7). In addition, its shorter headgroup, containing two acyl carbons rather than three as in DLin-MC3-DMA, reduces hydration around the titratable site at low pH (Figure 11). This weakly hydrated environment promotes deprotonation with increasing pH and facilitates subsequent migration into the membrane core (Figures 10, S47a, S47b, S49a, and S49b). ALC-0315, by contrast, has the most pronounced conical molecular shape among the investigated aminolipids, resulting in the largest membrane surface area at low pH (Figure S85). Despite this reduced lipid packing, its titratable site remains less solvent accessible than those of DLin-KC2-DMA, DLin-MC3-DMA, or DODAP at acidic pH. At the same time, the hydroxyl group and two ester moieties of ALC-0315 form an extended hydrogen-bonding network with neighboring aminolipids and PC lipids that persists even at elevated pH (Figure 6). Together with its pronounced acyl-chain mismatch with the surrounding phospholipids, this promotes lateral segregation, as illustrated for DSPC-containing membranes in Figure 9. This demixing is slightly stronger for ALC-0315 than for SM-102 (Figure 5) and likely facilitates deprotonation through charge-charge repulsion within aminolipid-rich domains.

Although DLin-MC3-DMA and SM-102 are structurally related to DLin-KC2-DMA and ALC-0315, respectively, both exhibited smaller pK_a shifts than their corresponding counterparts. Compared with DLin-KC2-DMA, DLin-MC3-DMA contains a longer headgroup with a polar ester linker, resulting in stronger hydrogen bonding and higher hydration around the titratable site at low pH (Figures 7 and 11). Consequently, DLin-MC3-DMA showed a weaker tendency to migrate into the membrane core (Figures 5, S47c, S47d, S49c, and S49d), stabilizing the protonated state by approximately 7.5–9.2 kJ mol^{-1} relative to DLin-KC2-DMA. SM-102 combines relatively low hydration with enhanced hydrogen-bonding capacity, similar to ALC-0315. However, its reduced chain mismatch lowers the tendency to form aminolipid-rich clusters (Figures 6 and 10), likely because its non-branched, fully saturated

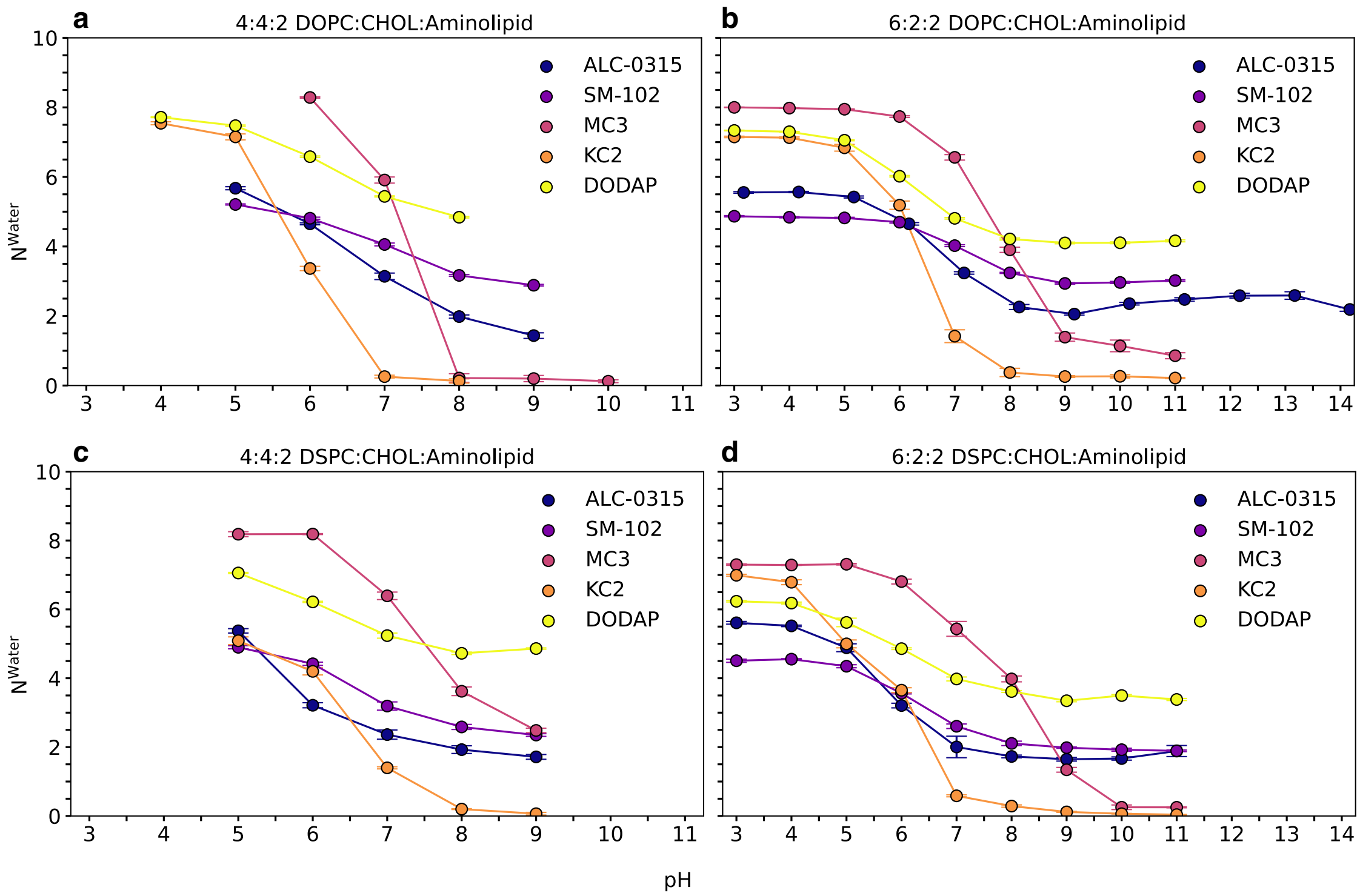

Figure 11. pH-dependent hydration of aminolipid titratable sites. Average number of water molecules within 0.5 nm of the aminolipid titratable site as a function of pH for systems containing (a and b) DOPC or (c and d) DSPC phospholipids. Error bars indicate the standard error of the mean, obtained by block averaging (see materials and methods). The values for the number of water molecules as a function of time are shown in Figures S71 – S74. ror bars indic CHOL, cholesterol.

acyl chain enables more favorable packing with phospholipids and cholesterol. In DSPC-containing membranes, this results in larger pK_a shifts for SM-102 than for DLin-MC3-DMA (Figure 4).

The smallest pK_a shift was observed for DODAP. This aminolipid remains anchored at the membrane interface by its highly polar, strongly hydrated headgroup (Figure 11) and by two ester linkages connecting the headgroup to the oleyl tails. DODAP preferentially forms hydrogen bonds with both cholesterol and phospholipids (Figure 7), which stabilizes its interfacial localization (Figures S47e, S47f, S49e and S4f) and thereby limits the magnitude of the apparent pK_a shift.

Taken together, the pK_a shifts of the investigated aminolipids are governed primarily by three molecular determinants: (1) solvent accessibility of the titratable site, (2) polarity and hydrogen-bonding capacity of the headgroup and linker region, and (3) acyl-chain mismatch with the surrounding phospholipids. Thus, aminolipid structure is a key determinant of pK_a shifts, but the magnitude of these shifts can still be significantly influenced by the membrane environment (Figures 4c and 4d).

Lipid environment as determinant of pK_a shifts

Increasing the cholesterol concentration from 20 to 40 mol % slightly enhanced the pK_a shifts (i.e., lowered the apparent pK_a, in DOPC-containing membranes), whereas in DSPC-containing membranes it caused only a minor reduction of the shifts, corresponding to slightly higher apparent pK_a values (Figure 4c). Cholesterol enrichment is known to condense lipid bilayers,[52,75] an effect that is also reflected in the reduced average membrane surface area of our systems (Figure S85). This condensation is more pronounced in membranes containing unsaturated DOPC than in membranes composed of saturated DSPC (Figure S86). Consequently, aminolipids in DOPC-containing membranes are brought into closer proximity, as reflected by the increased cumulative number of neighboring aminolipids in the 4:4:2 compositions (Figure S87). The resulting charge-charge repulsion destabilizes the protonated state and promotes larger pK_a shifts at higher cholesterol content. This effect is not observed for DODAP, where increased solvent accessibility of the titratable site (Figure S88) compensates the repulsion-driven shift. In DSPC-containing membranes, higher solvent accessibility at 40 mol % cholesterol (Figure S88) similarly stabilizes the charged state and counteracts condensation-driven pK_a shifts.

Compared with changes in cholesterol concentration, the choice of helper phospholipid had an even stronger effect on the magnitude of the pK_a shifts (Figure 4d). Replacing DOPC with DSPC generally increased the pK_a shift (i.e.,

lowered pK_a^{app}), consistent with reduced membrane surface area (Figure S89) and tighter aminolipid packing (Figures S90 and S91). This effect is further reinforced by enhanced segregation from saturated DSPC lipids due to larger acyl-chain mismatch, particularly for the branched aminolipids ALC-0315 and SM-102. Under 40 mol % cholesterol conditions, DLin-KC2-DMA and DLin-MC3-DMA deviated from this trend. The $\Delta\Delta pK_a$ value for DLin-KC2-DMA was small and only weakly significant, whereas DLin-MC3-DMA showed stabilization of the protonated state. This is consistent with reduced core migration in the 4:4:2 DSPC:cholesterol:MC3 membrane (Figure S49c) and the consequently higher solvent accessibility of the titratable site (Figures 11a–11c).

Although not explicitly investigated here, the mechanisms identified in this study suggest that increasing the aminolipid fraction toward values typical of LNP formulations (i.e., 40%–50 mol %) would likely further increase the magnitude of the apparent pK_a shifts. A higher density of titratable sites increases charge-charge repulsion between protonated aminolipids and simultaneously promotes formation of an aminolipid-rich hydrophobic core, in which a substantial fraction of aminolipids becomes shielded from the aqueous environment. Both effects are expected to favor deprotonation and thus lower the apparent pK_a. Consistent with this interpretation, our previous CpHMD simulations of ALC-0315-containing LNPs revealed two distinct compartments at neutral pH, a solvent-exposed surface layer and an aminolipid-rich core, accompanied by an apparent pK_a shift of 4.33.[18] As discussed above, meaningful comparison with experimentally determined LNP pK_a values is possible only when considering aminolipids located at the LNP-solvent interface, because this is the fraction probed by techniques such as the TNS assay.

Relations between simulation results and experimental observations

To our knowledge, no experimental data are available for planar lipid membranes with the compositions studied here. Nevertheless, qualitative comparisons to related experimental systems can be drawn, while keeping in mind that the simplified membrane compositions employed here do not fully recapitulate realistic LNP formulations. Small-angle X-ray scattering measurements by He et al.[63] on LNPs formulated at 50:10:38.5:1.5 mol % aminolipid:DSPC:cholesterol:DMG-PEG2000 reported headgroup *d*-spacings for DODMA, a DODAP analog, MC3, ALC-0315, and SM-102 that agree well with both the absolute values of 4–6 nm and the relative trend ALC-0315 < SM-102 < MC3 < DODMA observed here for DSPC-containing membranes (Figures 10c and 9d). Lipoplexes containing DOPC and DODAP or MC3 show slightly larger *d*-spacings and increased thickness at higher pH,[76] consistent with our observations.

For DODAP, our simulations do not indicate pronounced segregation. Consistent with this observation, ^{1}H MAS NOESY (Magic Angle Spinning Nuclear Overhauser Effect Spectroscopy) experiments on DMPC:DODAP mixtures reported a homogeneous lipid distribution over a broad composition range of 90:10 to 50:50 mol % DMPC:DODAP at pH 7.4.[77] Similarly, ^{2}H NMR measurements on POPC:DODAP membranes found no evidence for substantial domain formation between 90:10 and 20:80 mol % POPC:DODAP at pH 7.4.[78] In full LNPs, Carrasco et al.[20] reported a more heterogeneous internal morphology for DODAP-containing formulations than for DLin-MC3-DMA- or DLin-KC2-DMA-containing formulations, with bleb-like structures occurring more frequently.

More detailed experimental evidence for pH-dependent segregation and internal reorganization is available for DLin-KC2-DMA and DLin-MC3-DMA in realistic LNP formulations and simpler model membranes. Cryogenic transmission electron microscopy (cryo-TEM) studies consistently report electron-dense, internally amorphous morphologies for LNPs containing DLin-KC2-DMA[20,79–81] or DLin-MC3-DMA,[20,65,82,83] in agreement with the rapid and complete translocation of these aminolipids into the membrane core observed in our simulations. POPC:KC2:cholesterol systems studied experimentally and computationally by Ramezanpour et al.[81] showed that neutral KC2 segregates from POPC in both 70:30 mol % POPC:KC2 and 25:45:30 mol % POPC:cholesterol:KC2 membranes. However, the authors noted that the strong confinement of KC2 within the bilayer interior observed in MD simulations may partly result from the applied periodic boundary conditions. Ibrahim et al.[84] combined experiments and simulations to study MC3 in DOPC membranes at 5, 10, and 15 mol % MC3. They compared AMBER, Slipids, and CHARMM36 lipid models, with the CHARMM36 parameters matching those used here. Their optimized AMBER parameters and CHARMM36 showed broadly similar behavior, with deprotonated MC3 favoring the membrane core, whereas Slipids[85] predicted deprotonated MC3 to remain at the membrane-solvent interface. Although CHARMM36 showed a slightly greater tendency for deprotonated lipids to remain at the membrane surface and for protonated lipids to form more tightly packed membranes, both models reproduced neutron reflectometry data comparably well.

For the branched aminolipids, cryo-TEM and MD studies have consistently reported homogeneous, electron-dense interiors for LNPs containing ALC-0315[2,18,59,61,62] and SM-102.[59,82,83] However, no experimental data are currently available, to our knowledge, for these aminolipids under the relatively dilute conditions investigated here in planar model membranes. To further assess the significance of the lateral segregation observed in our simulations, we performed additional CpHMD simulations of 4:4:2 and 6:2:2 DOPC:cholesterol:ALC-0315/SM-102 membranes in which

all aminolipids were initially placed in a deprotonated state within the membrane core at pH 3, 5, 7, and 9 (Table S4). At 40 mol % cholesterol, corresponding to 50 mol % cholesterol in the DOPC/cholesterol surface, the condensing effect of cholesterol confined the aminolipids within the membrane core and prevented major membrane reorganization on the simulation timescale (Figures S92, S93, S96, S97, S102a, and S102c). Consequently, the aminolipids remained uniformly distributed within the membrane core and deprotonated irrespective of environmental pH (Figures S104a and S104c). These simulations indicate that membrane reorganizations involving aminolipids with lower diffusional mobility, arising from bulkier tails, higher cholesterol concentrations, or more saturated phospholipids, are associated with higher free-energy barriers and can become kinetically trapped on timescales accessible to atomistic CpHMD simulations.

In contrast, at 20 mol % cholesterol, corresponding to 25 mol % cholesterol in the DOPC/cholesterol surface, the aminolipids gradually migrated back toward the membrane surface (Figures S94, S95, S98, and S99), thereby decreasing the overall membrane thickness (Figures S102b and S102d). During this process, they adjusted their protonation state according to the environmental pH (Figures S104b and S104d). SM-102, which contains only one branched acyl chain, underwent faster and more pronounced reorganization than the bulkier ALC-0315, indicating higher diffusional mobility. For the 6:2:2 composition, the final membrane structures obtained from surface- and core-initialized simulations showed notable similarities (Figures S100 and S101). In the core-initialized ALC-0315 simulations, lateral segregation was even more pronounced, although the aminolipid-rich domains remained partially covered by DOPC lipids, as illustrated by the pH 9 snapshot in Figure S100.

Together, simulations starting from different initial structures indicate that branched aminolipids follow a distinct segregation pathway upon deprotonation compared with aminolipids bearing dioleyl or dilinoleyl tails. These results may depend on the specific membrane compositions studied here. In particular, the phospholipid-to-cholesterol ratio and the relatively low concentration of branched aminolipids may favor lateral rather than vertical segregation. Nevertheless, our findings are consistent with previous classical MD studies reporting concentration-dependent membrane reorganization. Dehghani-Ghahnaviyeh et al.,[86] for example, reported pronounced structural rearrangements in DSPC membranes containing 40 mol % cholesterol only when the lipid 5 concentration exceeded 20 mol % using a racemic mixture of protonated and deprotonated species corresponding approximately to pH 7. A similar trend was reported for KC2 by Ramezanpour et al.,[81] who found that a fraction of KC2 remained at the membrane surface in 90:10 mol % POPC:KC2 membranes, whereas KC2 localized entirely within the membrane core in 70:30 mol % POPC:KC2 membranes. These studies support the view that aminolipid segregation and membrane reorganization become increasingly pronounced with increasing aminolipid concentration in planar model membranes.

The overall agreement between simulations and experiments supports the use of the CHARMM36 force field for modeling aminolipid-containing membranes. For conventional membrane lipids, atomistic force fields such as CHARMM36, Lipid14, and Slipids reproduce key bilayer properties, including membrane thickness, area per lipid, and deuterium order parameters, with generally good agreement to experiment.[87,88] Similarly, comparisons of phospholipid-cholesterol systems have shown that CHARMM36, Slipids, Lipid17, OPLS, and related force fields capture similar qualitative trends for cholesterol-induced membrane condensation, although quantitative differences remain; CHARMM36 tends to show a comparatively strong condensation effect.[89,90] Therefore, while force-field-dependent differences in hydration, hydrogen bonding, and local packing are expected, the qualitative conclusions of this work are unlikely to depend strongly on the chosen parameter set. The observed protonation-dependent membrane reorganization is governed by fundamental molecular properties, including lipid shape, hydration, and hydrogen-bonding capacity. Nevertheless, given the highly charged nature of aminolipid-containing membranes, particularly at low pH, future work should assess the influence of charge-scaling approaches such as those employed in the prosECCo75 force field.[91]

CONCLUSION

Using all-atom CpHMD simulations, this study demonstrates that aminolipid protonation equilibria and membrane reorganization are jointly governed by aminolipid molecular structure and lipid membrane composition. CpHMD parameters for each aminolipid were derived using thermodynamic integration[49] and applied to simplified ternary membrane systems, enabling an unbiased description of pH-dependent protonation and membrane remodeling. Although these model membranes do not reproduce the full structural complexity of LNPs, they provide controlled access to the local interfacial lipid environment that governs aminolipid protonation behavior. At the same time, this approach avoids artificial constraints on lateral degrees of freedom, such as fixed monolayer area, which may suppress intrinsic pH-driven reorganization and lead to non-physical charge distributions in LNP-mimetic systems.[92]

Across all investigated compositions, the apparent, or functional, aminolipid pK_a values were lower than the corresponding intrinsic values at infinite dilution, consistent with experimental observations for LNPs. The magnitude of this shift depended on both aminolipid architecture and membrane composition. Distinct reorganization mechanisms emerged for different aminolipid classes. The sketch (Figure 12) summarizes the membrane organizations and associated protonation states of the investigated aminolipids

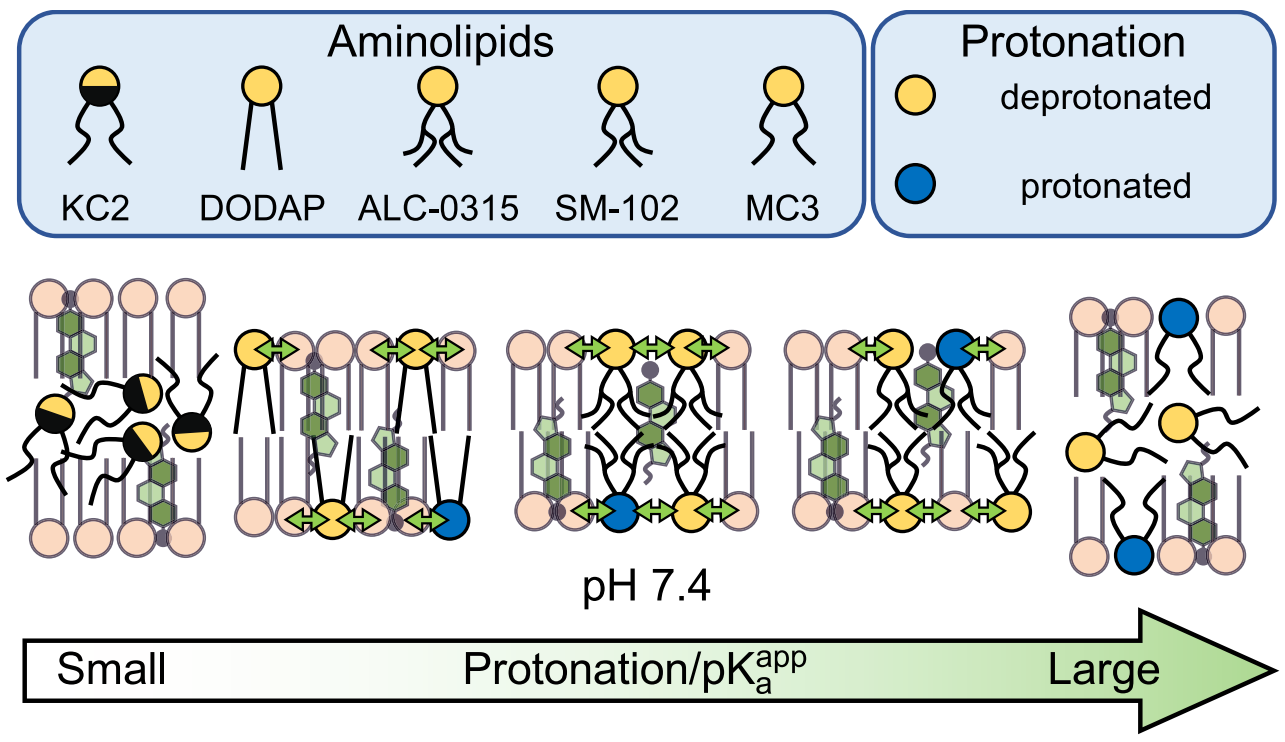

Figure 12. Overview of aminolipid protonation and membrane organization at pH 7.4. The scheme summarizes the general behavior of the aminolipids investigated in this study based on the protonation patterns derived from the titration curves in Figure 2 and the membrane organizations shown in Figures 7, 8, and S7 – S46 at physiological pH (7.4). The aminolipid structures and membrane schematics are arranged according to the average degree of (de-)protonation/pK$_a^{app}$ across all membrane compositions. Green arrows indicate enhanced hydrogen bonding between lipids.

at pH 7.4. The polyunsaturated aminolipids DLin-KC2-DMA and DLin-MC3-DMA rapidly segregated from helper lipids upon deprotonation and migrated toward the hydrophobic membrane core. This behavior reflects the fluidity of their diunsaturated acyl chains and their limited capacity to maintain stabilizing hydrogen bonds at the membrane interface. In contrast, DODAP remained localized at the membrane surface over the full pH range because its polar, hydrated headgroup and ester linkages stabilize interfacial localization, resulting in the smallest observed ΔpK_a.

The branched aminolipids ALC-0315 and SM-102 followed a different reorganization pathway. Upon deprotonation, they underwent pronounced lateral segregation and intermediate headgroup dehydration while remaining largely surface localized. Rather than migrating into the membrane core, as observed for DLin-KC2-DMA and DLin-MC3-DMA, these aminolipids formed in-plane aminolipid-rich domains. Their ΔpK_a values were highly sensitive to helper lipid identity, indicating that acyl-chain mismatch and local packing are major determinants of their protonation equilibria. This distinct reorganization pathway may influence the surface-to-core distribution of branched aminolipids in LNPs. Enrichment of inverted-cone-shaped aminolipids at the particle surface could increase interfacial curvature stress and thereby contribute to larger LNP radii.

Collectively, our results show that aminolipid phase separation, whether vertical through surface-to-core migration or lateral through in-plane clustering, destabilizes the protonated state and increases the shift between intrinsic and apparent pK_a values. The extent and nature of this shift depend critically on aminolipid molecular geometry and membrane composition. Saturated helper lipids such as DSPC generally enhanced segregation relative to unsaturated DOPC and amplified pK_a shifts across aminolipid classes. These findings identify distinct molecular mechanisms by which LNP composition can modulate aminolipid pH response. More broadly, they demonstrate that simplified, compositionally controlled membrane systems can provide mechanistic insight into aminolipid protonation and membrane remodeling, thereby supporting the rational design of next-generation LNP formulations.

DATA AVAILABILITY

All simulation input files, force-field parameters, topology files, and representative starting structures are made availableon Zenodo (https://doi.org/10.5281/zenodo.21240872).

ACKNOWLEDGMENTS

The authors gratefully acknowledge the scientific support and HPC resources provided by the Erlangen National High Performance Computing Center (NHR@FAU) of the Friedrich-Alexander-Universität Erlangen-Nürnberg (FAU). The hardware is funded by the German Research Foundation (DFG).

AUTHOR CONTRIBUTIONS

M.F.W.T and R.A.B. designed the research. M.F.W.T. carried out all simulations. M.F.W.T. and P.R. analyzed the data. All authors wrote the article.

DECLARATION OF INTERESTS

The authors declare no competing interests.

SUPPLEMENTAL INFORMATION

Supplemental Information can be found online at https://doi.org/10.1016/j.bpj.2026.06.030.

REFERENCES

1. Brimacombe, C. A., J. A. Kulkarni, …, P. R. Cullis. 2025. Rational design of lipid nanoparticles for enabling gene therapies. *Mol. Ther. Methods Clin. Dev.* 33:101518.
2. Trollmann, M. F. W., and R. A. Böckmann. 2022. mRNA lipid nanoparticle phase transition. *Biophys. J.* 121:3927–3939.
3. Leung, A. K. K., Y. Y. C. Tam, and P. R. Cullis. 2014. Lipid nanoparticles for short interfering RNA delivery. *Adv. Genet.* 88:71–110.
4. Hamilton, N. B., S. Arns, …, J. C. Shelley. 2025. Calculating apparent pKa values of ionizable lipids in lipid nanoparticles. *Mol. Pharm.* 22:588–593.
5. Simonsen, J. B., and P. Larsson. 2025. A perspective on the apparent pKa of ionizable lipids in mRNA-LNPs. *J. Contr. Release.* 384:113879.
6. McMillan, C., A. Druschitz, …, Y. Perrie. 2025. Influence of ionisable lipid and sterol variations on lipid nanoparticle properties and performance. *J. Contr. Release.* 386:114056.
7. Wang, C., Y. Zhou, …, Y. Zhang. 2025. Lipid nanoparticles consisting of sterol-conjugated ionizable lipids enable prolonged and safe mRNA delivery. *ACS Appl. Mater. Interfaces.* 17:37763–37773.
8. Ansell, S. M., and X. Du. 2015. Novel lipids and lipid nanoparticle formulations for delivery of nucleic acids. *World Patent WO-2017075531-A1*, filed October 28, 2016, and published May 4, 2017.
9. Hope, M. J., B. Mui, …, X. Du. 2018. Lipid nanoparticle formulations. *World patent WO-2018081480-A1*, filed October 26, 2017, and published May 3, 2018.

10. Chander, N., G. Basha, …, P. R. Cullis. 2023. Lipid nanoparticle mRNA systems containing high levels of sphingomyelin engender higher protein expression in hepatic and extra-hepatic tissues. *Mol. Ther. Methods Clin. Dev.* 30:235–245.

11. Ge, X., L. Chen, …, W. Yuan. 2021. Rationale and application of PEGylated lipid-based system for advanced target delivery of siRNA. *Front. Pharmacol.* 11:598175.

12. Mui, B. L., Y. K. Tam, …, M. J. Hope. 2013. Influence of polyethylene glycol lipid desorption rates on pharmacokinetics and pharmacodynamics of siRNA lipid nanoparticles. *Mol. Ther. Nucleic Acids.* 2:e139.

13. Berger, M., M. Degey, …, G. Piel. 2023. Effect of PEG anchor and serum on Lipid Nanoparticles: Development of a nanoparticles tracking method. *Pharmaceutics.* 15:597.

14. Suzuki, T., Y. Suzuki, …, H. Ishihara. 2020. PEG shedding-rate-dependent blood clearance of PEGylated lipid nanoparticles in mice: Faster PEG shedding attenuates anti-PEG IgM production. *Int. J. Pharm.* 588:119792.

15. Kurek, D., D. Witzigmann, and J. Kulkarni. 2025. Method of delivery of DNA or RNA cargo unshielded lipid nanoparticles and compositions thereof. *US Patent US-12343429-B2*, filed October 4, 2024, and published July 1, 2025.

16. Escalona-Rayo, O., Y. Zeng, …, A. Kros. 2023. In vitro and in vivo evaluation of clinically-approved ionizable cationic lipids shows divergent results between mRNA transfection and vaccine efficacy. *Biomed. Pharmacother.* 165:115065.

17. Audouy, S. A. L., L. F. M. H. de Leij, …, G. Molema. 2002. In vivo characteristics of cationic liposomes as delivery vectors for gene therapy. *Pharm. Res.* 19:1599–1605.

18. Trollmann, M. F. W., and R. A. Böckmann. 2026. Decoding pH-Driven Phase Transition of Lipid Nanoparticles. *Small* e11381.

19. Johansson, J. M., H. Du Rietz, …, A. Wittrup. 2025. Cellular and biophysical barriers to lipid nanoparticle mediated delivery of RNA to the cytosol. *Nat. Commun.* 16:5354.

20. Carrasco, M. J., S. Alishetty, …, M. D. Buschmann. 2021. Ionization and structural properties of mRNA lipid nanoparticles influence expression in intramuscular and intravascular administration. *Commun. Biol.* 4:956.

21. Zhang, C., Y. Ma, …, T. Liu. 2022. Modification of Lipid-Based Nanoparticles: An Efficient Delivery System for Nucleic Acid-Based Immunotherapy. *Molecules.* 27:1943.

22. Heyes, J., L. Palmer, …, I. MacLachlan. 2005. Cationic lipid saturation influences intracellular delivery of encapsulated nucleic acids. *J. Contr. Release.* 107:276–287.

23. Zhao, B., A. Kamanzi, …, P. R. Cullis. 2024. Determination of the interior pH of lipid nanoparticles using a pH-sensitive fluorescent dye-based DNA probe. *Biosens. Bioelectron. X.* 251:116065.

24. Aho, N., P. Buslaev, …, B. Hess. 2022. Scalable Constant pH Molecular Dynamics in GROMACS. *J. Chem. Theor. Comput.* 18:6148–6160.

25. Trollmann, M. F., P. Rossetti, and R. A. Böckmann. 2024. Constant-pH MD Simulations of Lipids. Preprint at bioRxiv. https://doi.org/10.1101/2024.12.06.627182.

26. Tesei, G., Y.-W. Hsiao, …, L. Lindfors. 2024. Lipid shape and packing are key for optimal design of pH-sensitive mRNA lipid nanoparticles. *Proc. Natl. Acad. Sci. USA.* 121:e2311700120.

27. Kristen, A. V., S. Ajroud-Driss, …, L. Obici. 2019. Patisiran, an RNAi therapeutic for the treatment of hereditary transthyretin-mediated amyloidosis. *Neurodegener. Dis. Manag.* 9:5–23.

28. Akinc, A., A. Zumbuehl, …, D. G. Anderson. 2008. A combinatorial library of lipid-like materials for delivery of RNAi therapeutics. *Nat. Biotechnol.* 26:561–569.

29. Ferraresso, F., A. W. Strilchuk, …, C. J. Kastrup. 2022. Comparison of DLin-MC3-DMA and ALC-0315 for siRNA delivery to hepatocytes and hepatic stellate cells. *Mol. Pharm.* 19:2175–2182.

30. Buschmann, M. D., M. J. Carrasco, …, D. Weissman. 2021. Nanomaterial delivery systems for mrna vaccines. *Vaccines.* 9:65.

31. Atmuri, N. D. P., F. Saadati, …, M. A. Ciufolini. 2025. Design of cationic ionizable lipids for the delivery of therapeutic nucleic acids. *Mol. Ther. Methods Clin. Dev.* 33:101585.

32. Schott-Verdugo, S., and H. Gohlke. 2019. PACKMOL-Memgen: A simple-to-use, generalized workflow for membrane-protein-lipid-bilayer system building. *J. Chem. Inf. Model.* 59:2522–2528.

33. Case, D. A., H. M. Aktulga, …, K. M. Merz, Jr. 2023. AmberTools. *J. Chem. Inf. Model.* 63:6183–6191.

34. Jo, S., T. Kim, …, W. Im. 2008. CHARMM-GUI: a web-based graphical user interface for CHARMM. *J. Comput. Chem.* 29:1859–1865.

35. MacKerell, A. D., D. Bashford, …, D. Yin. 1998. All-Atom Empirical Potential for Molecular Modeling and Dynamics Studies of Proteins. *J. Phys. Chem. B.* 102:3586–3616.

36. Beutler, T. C., A. E. Mark, …, W. F. van Gunsteren. 1994. Avoiding singularities and numerical instabilities in free energy calculations based on molecular simulations. *Chem. Phys. Lett.* 222:529–539.

37. Abraham, M., A. Alekseenko, …, E. Lindahl. 2025. GROMACS 2025.3 Manual. https://doi.org/10.5281/zenodo.16992569.

38. Vanommeslaeghe, K., E. Hatcher, …, A. D. Mackerell, Jr. 2010. CHARMM general force field: A force field for drug-like molecules compatible with the CHARMM all-atom additive biological force fields. *J. Comput. Chem.* 31:671–690.

39. Klauda, J. B., R. M. Venable, …, R. W. Pastor. 2010. Update of the CHARMM all-atom additive force field for lipids: validation on six lipid types. *J. Phys. Chem. B.* 114:7830–7843.

40. Park, S., Y. K. Choi, …, W. Im. 2021. CHARMM-GUI Membrane Builder for lipid nanoparticles with ionizable cationic lipids and PEGylated lipids. *J. Chem. Inf. Model.* 61:5192–5202.

41. Vanommeslaeghe, K., and A. D. MacKerell, Jr. 2012. Automation of the CHARMM General Force Field (CGenFF) I: bond perception and atom typing. *J. Chem. Inf. Model.* 52:3144–3154.

42. Vanommeslaeghe, K., E. P. Raman, and A. D. MacKerell, Jr. 2012. Automation of the CHARMM General Force Field (CGenFF) II: assignment of bonded parameters and partial atomic charges. *J. Chem. Inf. Model.* 52:3155–3168.

43. SILCSBIO. 2023. CGenFF. https://cgenff.com.

44. Pitman, M. C., F. Suits, …, S. E. Feller. 2004. Molecular-level organization of saturated and polyunsaturated fatty acids in a phosphatidylcholine bilayer containing cholesterol. *Biochemistry.* 43:15318–15328.

45. Lim, J. B., B. Rogaski, and J. B. Klauda. 2012. Update of the cholesterol force field parameters in CHARMM. *J. Phys. Chem. B.* 116:203–210.

46. Darden, T., D. York, and L. Pedersen. 1993. Particle Mesh Ewald: An N log(N) Method for Ewald Sums in Large Systems. *J. Chem. Phys.* 98:10089–10092.

47. Van Der Spoel, D., E. Lindahl, …, H. J. C. Berendsen. 2005. GROMACS: fast, flexible, and free. *J. Comput. Chem.* 26:1701–1718.

48. Abraham, M. J., T. Murtola, …, E. Lindahl. 2015. GROMACS: High performance molecular simulations through multi-level parallelism from laptops to supercomputers. *SoftwareX.* 1–2:19–25.

49. Buslaev, P., N. Aho, …, G. Groenhof. 2022. Best Practices in Constant pH MD Simulations: Accuracy and Sampling. *J. Chem. Theor. Comput.* 18:6134–6147.

50. Jansen, A., N. Aho, …, B. Hess. 2024. phbuilder: A Tool for Efficiently Setting up Constant pH Molecular Dynamics Simulations in GROMACS. *J. Chem. Inf. Model.* 64:567–574.

51. Hess, B. 2002. Determining the shear viscosity of model liquids from molecular dynamics simulations. *J. Chem. Phys.* 116:209–217.

52. Pöhnl, M., M. F. W. Trollmann, and R. A. Böckmann. 2023. Nonuniversal impact of cholesterol on membranes mobility, curvature sensing and elasticity. *Nat. Commun.* 14:8038.

53. Ergüder, M. F., and M. Deserno. 2021. Identifying systematic errors in a power spectral analysis of simulated lipid membranes. *J. Chem. Phys.* 154:214103.

54. Bai, L., and D. Breen. 2008. Calculating center of mass in an unbounded 2D environment. *J. Graph. Tool.* 13:53–60.

55. Brandani, G. B., M. Schor, …, D. Marenduzzo. 2013. Quantifying disorder through conditional entropy: an application to fluid mixing. *PLoS One.* 8:e65617.

56. Schrödinger, LLC. 2024. The PyMOL Molecular Graphics System, Version 3.1. https://github.com/schrodinger/pymol-open-source.

57. Grava, M., M. Ibrahim, …, E. Schneck. 2023. Combining molecular dynamics simulations and x-ray scattering techniques for the accurate treatment of protonation degree and packing of ionizable lipids in monolayers. *J. Chem. Phys.* 159:154706.

58. Leung, A. K. K., I. M. Hafez, …, P. R. Cullis. 2012. Lipid nanoparticles containing siRNA synthesized by microfluidic mixing exhibit an electron-dense nanostructured core. *J. Phys. Chem. C.* 116:18440–18450.

59. Paloncýová, M., P. Čechová, …, M. Otyepka. 2021. Role of ionizable lipids in SARS-CoV-2 vaccines as revealed by molecular dynamics simulations: From membrane structure to interaction with mRNA fragments. *J. Phys. Chem. Lett.* 12:11199–11205.

60. Colston, K. J., S. C. Monsalve, and S. T. Schneebeli. 2025. Structure-based modeling of environment-dependent protonation states across LNP formulations with atomistic CpHMD. *Mol. Pharm.* 22:7347–7358.

61. Binici, B., Z. Rattray, and Y. Perrie. 2025. A comparative study of cationic lipid-enriched LNPs for mRNA vaccine delivery. *Int. J. Pharm.* 682:125941.

62. McMillan, C., A. Druschitz, …, Y. Perrie. 2024. Tailoring lipid nanoparticle dimensions through manufacturing processes. *RSC Pharm.* 1:841–853.

63. He, Y., E. H. Pilkington, …, C. W. Pouton. 2025. Morphological changes induced by buffer exchange during preparation of mRNA-lipid nanoparticles occur in a stepwise manner as pH is elevated. *Mol. Ther. Methods Clin. Dev.* 33:101578.

64. Hald Albertsen, C., J. A. Kulkarni, …, J. B. Simonsen. 2022. The role of lipid components in lipid nanoparticles for vaccines and gene therapy. *Adv. Drug Deliv. Rev.* 188:114416.

65. Yanez Arteta, M., T. Kjellman, …, L. Lindfors. 2018. Successful reprogramming of cellular protein production through mRNA delivered by functionalized lipid nanoparticles. *Proc. Natl. Acad. Sci. USA.* 115:E3351–E3360.

66. Sebastiani, F., M. Yanez Arteta, …, M. Cárdenas. 2021. Apolipoprotein E binding drives structural and compositional rearrangement of mRNA-containing lipid nanoparticles. *ACS Nano.* 15:6709–6722.

67. Hossain, K. A., M. Valerio, …, M. Orozco. 2026. When Lipids Embrace RNA: pH-Driven Dynamics and Mechanisms of LNP-Mediated siRNA Delivery. Preprint at bioRxiv. https://doi.org/10.64898/2026.02.11.705380.

68. Niederkofler, S., P. Parkkila, …, F. Höök. 2025. Effects of serum incubation on lipid nanoparticle PEG shedding, mRNA retention, and membrane interactions. *ACS Appl. Mater. Interfaces.* 17:64219–64231.

69. Abe, S., H. Takata, …, T. Ishida. 2025. Impact of pre-existing anti-polyethylene glycol (PEG) IgM on biodistribution and humoral response of intramuscularly administered PEGylated mRNA loaded lipid nanoparticle. *J. Contr. Release.* 383:113821.

70. Sabnis, S., E. S. Kumarasinghe, …, K. E. Benenato. 2018. A novel amino lipid series for mRNA delivery: Improved endosomal escape and sustained pharmacology and safety in non-human primates. *Mol. Ther.* 26:1509–1519.

71. Burdette, D., L. Ci, …, T. Hendrick. 2023. Systemic exposure, metabolism, and elimination of [14C]-labeled amino lipid, lipid 5, after a single administration of mRNA encapsulating lipid nanoparticles to Sprague-Dawley rats. *Drug Metab. Dispos.* 51:804–812.

72. Chahal, G. S., K. J. Helbig, …, E. A. Monson. 2026. The biology of endosomal escape: Strategies for enhanced delivery of therapeutics. *ACS Nano.* 20:1789–1813.

73. Parhiz, H., J. S. Brenner, …, V. R. Muzykantov. 2022. Added to pre-existing inflammation, mRNA-lipid nanoparticles induce inflammation exacerbation (IE). *J. Contr. Release.* 344:50–61.

74. Cheng, M. H. Y., Y. Zhang, …, P. R. Cullis. 2025. Liposomal lipid nanoparticles for extrahepatic delivery of mRNA. *Nat. Commun.* 16:4135.

75. Hung, W.-C., M.-T. Lee, …, H. W. Huang. 2007. The condensing effect of cholesterol in lipid bilayers. *Biophys. J.* 92:3960–3967.

76. Uebbing, L., A. Ziller, …, P. Langguth. 2020. Investigation of pH-responsiveness inside lipid nanoparticles for parenteral mRNA application using small-angle X-ray scattering. *Langmuir.* 36:13331–13341.

77. Huster, D., U. Dietrich, …, K. Arnold. 2000. Lipid matrix properties in cationic membranes interacting with anionic polyelectrolytes: A solid-state NMR approach. *Langmuir.* 16:9225–9232.

78. Mitrakos, P., and P. M. Macdonald. 1996. DNA-induced lateral segregation of cationic amphiphiles in lipid bilayer membranes as detected via 2H NMR. *Biochemistry.* 35:16714–16722.

79. Kulkarni, J. A., D. Witzigmann, …, P. R. Cullis. 2019. On the role of helper lipids in lipid nanoparticle formulations of siRNA. *Nanoscale.* 11:21733–21739.

80. Kulkarni, J. A., M. M. Darjuan, …, P. R. Cullis. 2018. On the formation and morphology of lipid nanoparticles containing ionizable cationic lipids and siRNA. *ACS Nano.* 12:4787–4795.

81. Ramezanpour, M., M. L. Schmidt, …, D. P. Tieleman. 2019. Ionizable amino lipid interactions with POPC: implications for lipid nanoparticle function. *Nanoscale.* 11:14141–14146.

82. Deng, S., H. Shao, …, Z. Zhao. 2024. Development of a cationic polymeric micellar structure with endosomal escape capability enables enhanced intramuscular transfection of mRNA-LNPs. *Vaccines (Basel).* 13:25.

83. Reinhart, A.-G., A. Osterwald, …, M. Keller. 2023. Investigations into mRNA lipid nanoparticles shelf-life stability under nonfrozen conditions. *Mol. Pharm.* 20:6492–6503.

84. Ibrahim, M., J. Gilbert, …, N. Schwierz. 2023. Structural insights on ionizable Dlin-MC3-DMA lipids in DOPC layers by combining accurate atomistic force fields, molecular dynamics simulations and neutron reflectivity. *Nanoscale.* 15:11647–11656.

85. Ermilova, I., and J. Swenson. 2020. DOPC versus DOPE as a helper lipid for gene-therapies: molecular dynamics simulations with DLin-MC3-DMA. *Phys. Chem. Chem. Phys.* 22:28256–28268.

86. Dehghani-Ghahnaviyeh, S., M. Smith, …, S. Sur. 2023. Ionizable amino lipids distribution and effects on DSPC/cholesterol membranes: Implications for lipid nanoparticle structure. *J. Phys. Chem. B.* 127:6928–6939.

87. Leonard, A. N., E. Wang, …, J. B. Klauda. 2019. Developing and testing of lipid force fields with applications to modeling cellular membranes. *Chem. Rev.* 119:6227–6269.

88. Pluhackova, K., S. A. Kirsch, …, R. A. Böckmann. 2016. A Critical Comparison of Biomembrane Force Fields: Structure and Dynamics of Model DMPC, POPC, and POPE Bilayers. *J. Phys. Chem. B.* 120:3888–3903.

89. Javanainen, M., P. Heftberger, …, O. H. S. Ollila. 2023. Quantitative comparison against experiments reveals imperfections in force fields' descriptions of POPC-cholesterol interactions. *J. Chem. Theor. Comput.* 19:6342–6352.

90. Sawdon, J., T. J. Piggot, and J. W. Essex. 2025. How well do empirical molecular mechanics force fields model the cholesterol condensing effect? *J. Chem. Phys.* 162:044901.

91. Nencini, R., C. Tempra, …, H. Martinez-Seara. 2024. Effective inclusion of electronic polarization improves the description of electrostatic interactions: The prosECCo75 biomolecular force field. *J. Chem. Theor. Comput.* 20:7546–7559.

92. Trollmann, M. F. W., P. Rossetti, and R. A. Böckmann. 2025. Revisiting lipid nanoparticle composition and structure: A critical take on simulation approaches. *Proc. Natl. Acad. Sci. USA.* 122:e2422995122.

Biophysical Journal, Volume *125*

Supplemental information

Membrane environment sets the functional pK_a of ionizable lipids

Marius F.W. Trollmann, Paolo Rossetti, and Rainer A. Böckmann

Supplementary Material: Membrane Environment Sets the Functional pK_a of Ionizable Lipids

Marius F.W. Trollmann,[*,†,§] Paolo Rossetti,[†] and Rainer A. Böckmann[*,†,‡,¶,§]

†*Computational Biology, Department of Biology, Friedrich-Alexander-Universität Erlangen-Nürnberg, Erlangen, Germany*

‡*FAU Profile Center Immunomedicine (FAU I-MED), Erlangen, Germany*

¶*FAU Research Center New Bioactive Compounds – FAU NeW, Erlangen, Germany*

§*Erlangen National High-Performance Computing Center (NHR@FAU), Erlangen, Germany*

E-mail: marius.trollmann@fau.de; rainer.boeckmann@fau.de

Phone: +49 (0)9131 8525409

The PDF file includes

- Tables S1-S4
- Figures S1 – S105,
- References

Supplementary Tables

Table S1: All-atom molecular dynamics simulations using the constant-pH method to parametrize and validate $V^{MM}(\lambda)$.

System	Aminolipid:H_2O:Na^+:Cl^-:Buf	Sim. Time (μs)	pH	Objective
$DODAP_A$	1:6945:19:20:1	0.6	-	Thermodynamic Integration
$DLin\text{-}MC3\text{-}DMA_A$[a]	1:6944:19:20:1	0.6	-	Thermodynamic Integration
$DLin\text{-}KC2\text{-}DMA_A$	1:6948:19:20:1	0.6	-	Thermodynamic Integration
$SM\text{-}102_A$	1:6941:19:20:1	1.7	-	Thermodynamic Integration
$ALC\text{-}0315_A$[b]	1:6938:19:20:1	1.7	-	Thermodynamic Integration
$DODAP_B$	1:6944:19:20:3	0.6	-	Goodness of polynomial coefficients
$DLin\text{-}MC3\text{-}DMA_B$[a]	1:6943:19:20:3	0.3	-	Goodness of polynomial coefficients
$DLin\text{-}KC2\text{-}DMA_B$	1:6947:19:20:3	0.6	-	Goodness of polynomial coefficients
$SM\text{-}102_B$	1:6940:19:20:3	1.0	-	Goodness of polynomial coefficients
$ALC\text{-}0315_B$[b]	1:6937:19:20:3	1.7	-	Goodness of polynomial coefficients
$DODAP_C$	1:6944:19:20:3	10×0.6	5–14	Intrinsic pK_a
$DLin\text{-}MC3\text{-}DMA_C$[a]	1:6943:19:20:3	10×0.3	5–14	Intrinsic pK_a
$DLin\text{-}KC2\text{-}DMA_C$	1:6947:19:20:3	10×0.6	5–14	Intrinsic pK_a
$SM\text{-}102_C$	1:6940:19:20:3	10×1.0	5–14	Intrinsic pK_a
$ALC\text{-}0315_C$[b]	1:6937:19:20:3	10×1.0	4.17–14.17	Intrinsic pK_a

[a] Equivalent to.[1]
[b] Equivalent to[2]

Table S2: All-atom molecular dynamics simulations of ternary membranes including the helperlipid DOPC using the constant-pH method.

System	Aminolipid:DOPC:Chol	$H_2O:Na^+:Cl^-:Buf$	Sim. Time (μs)	pH	Objective
$DODAP_A$	48:144:48	11903:0:48:97	0.5	3–11	Apparent pK_a
$DLin\text{-}MC3\text{-}DMA_A$[a]	48:144:48	11903:0:48:97	0.5	3–11	Apparent pK_a
$DLin\text{-}KC2\text{-}DMA_A$	48:144:48	11903:0:48:97	0.5	3–11	Apparent pK_a
$SM\text{-}102_A$	48:144:48	11317:0:48:97	0.5	3–11	Apparent pK_a
$ALC\text{-}0315_A$	48:144:48	11833:0:48:97	0.5	3.17–14.17	Apparent pK_a
$DODAP_A$	48:96:96	11903:0:48:97	0.5	4–8	Apparent pK_a
$DLin\text{-}MC3\text{-}DMA_A$	48:96:96	11903:0:48:97	0.5	6–10	Apparent pK_a
$DLin\text{-}KC2\text{-}DMA_A$	48:96:96	11903:0:48:97	0.5	4–8	Apparent pK_a
$SM\text{-}102_A$	48:96:96	11855:0:48:97	0.5	5–9	Apparent pK_a
$ALC\text{-}0315_A$	48:96:96	11855:0:48:97	0.5	5–9	Apparent pK_a
$DODAP_B$	48:144:48	11903:0:48/48/44/27/11/3/0/0/0:24	0.5	3–11	Apparent pK_a
$DLin\text{-}MC3\text{-}DMA_B$	48:144:48	11903:0:48/48/48/45/31/15/2/0/0:22	0.5	3–11	Apparent pK_a
$DLin\text{-}KC2\text{-}DMA_B$	48:144:48	11903:0:48/48/46/34/9/3/0/0/0:12	0.5	3–11	Apparent pK_a
$SM\text{-}102_B$	48:144:48	11317:0:48/48/48/45/30/9/4/1/0:26	0.5	3–11	Apparent pK_a
$ALC\text{-}0315_B$	48:144:48	11833:0:34/13/4/2/0/0/0/0/0:18	0.5	3.17–14.17	Apparent pK_a
$DODAP_B$	48:96:96	11903:0:48/46/29/9/0:26	0.5	4–8	Apparent pK_a
$DLin\text{-}MC3\text{-}DMA_B$	48:96:96	11903:0:47/36/11/1/0:20	0.5	6–10	Apparent pK_a
$DLin\text{-}KC2\text{-}DMA_B$	48:96:96	11903:0:48/43/33/12/1:14	0.5	4–8	Apparent pK_a
$SM\text{-}102_B$	48:96:96	11855:0:47/42/24/8/4:22	0.5	5–9	Apparent pK_a
$ALC\text{-}0315_B$	48:96:96	11855:0:46/32/17/2/0:22	0.5	5–9	Apparent pK_a
$DLin\text{-}MC3\text{-}DMA_C$	48:144:48	11903:0:48/48/48/45/31/15/2/0/0:22	1.0	3–11	Apparent pK_a
$DLin\text{-}KC2\text{-}DMA_C$	48:144:48	11903:0:48/48/46/34/9/3/0/0/0:12	1.0	3–11	Apparent pK_a
$SM\text{-}102_C$	48:144:48	11317:0:48/48/48/45/30/9/4/1/0:26	1.0	3–11	Apparent pK_a
$ALC\text{-}0315_C$	48:144:48	11833:0:34/13/4/2/0/0/0/0/0:18	1.0	3.17–14.17	Apparent pK_a
$DLin\text{-}MC3\text{-}DMA_C$	48:96:96	11903:0:47/36/11/1/0:20	1.0	6–10	Apparent pK_a
$DLin\text{-}KC2\text{-}DMA_C$	48:96:96	11903:0:48/43/33/12/1:14	1.0	4–8	Apparent pK_a
$SM\text{-}102_C$	48:96:96	11855:0:47/42/24/8/4:22	1.0	5–9	Apparent pK_a
$ALC\text{-}0315_C$	48:96:96	11855:0:46/32/17/2/0:22	1.0	5–9	Apparent pK_a

[a]Equivalent to[2]

Table S3: All-atom molecular dynamics simulations of ternary membranes including the helperlipid DSPC using the constant-pH method.

System	Aminolipid:DSPC:Chol	$H_2O:Na^+:Cl^-:Buf$	Sim. Time (μs)	pH	Objective
$DODAP_A$	48:144:48	11903:0:48:97	0.5	3–11	Apparent pK_a
DLin-MC3-DMA_A	48:144:48	11903:0:48:97	0.5	3–11	Apparent pK_a
DLin-KC2-DMA_A	48:144:48	11903:0:48:97	0.5	3–11	Apparent pK_a
SM-102_A	48:144:48	11317:0:48:97	0.5	3–11	Apparent pK_a
ALC-0315_A	48:144:48	11833:0:48:97	0.5	3–11	Apparent pK_a
$DODAP_A$	48:96:96	11903:0:48:97	0.5	4–8	Apparent pK_a
DLin-MC3-DMA_A	48:96:96	11903:0:48:97	0.5	6–10	Apparent pK_a
DLin-KC2-DMA_A	48:96:96	11903:0:48:97	0.5	4–8	Apparent pK_a
SM-102_A	48:96:96	11855:0:48:97	0.5	5–9	Apparent pK_a
ALC-0315_A	48:96:96	11855:0:48:97	0.5	5–9	Apparent pK_a
$DODAP_B$	48:144:48	11903:0:48/48/44/27/11/3/0/0/0:24	0.5	3–11	Apparent pK_a
DLin-MC3-DMA_B	48:144:48	11903:0:48/48/48/45/31/15/2/0/0:22	0.5	3–11	Apparent pK_a
DLin-KC2-DMA_B	48:144:48	11903:0:48/48/46/34/9/3/0/0/0:12	0.5	3–11	Apparent pK_a
SM-102_B	48:144:48	11317:0:48/48/48/45/30/9/4/1/0:26	0.5	3–11	Apparent pK_a
ALC-0315_B	48:144:48	11833:0:34/13/4/2/0/0/0/0/0:18	0.5	3–11	Apparent pK_a
$DODAP_B$	48:96:96	11903:0:48/46/29/9/0:26	0.5	4–8	Apparent pK_a
DLin-MC3-DMA_B	48:96:96	11903:0:47/36/11/1/0:20	0.5	6–10	Apparent pK_a
DLin-KC2-DMA_B	48:96:96	11903:0:48/43/33/12/1:14	0.5	4–8	Apparent pK_a
SM-102_B	48:96:96	11855:0:47/42/24/8/4:22	0.5	5–9	Apparent pK_a
ALC-0315_B	48:96:96	11855:0:46/32/17/2/0:22	0.5	5–9	Apparent pK_a
DLin-MC3-DMA_C	48:144:48	11903:0:48/48/48/45/31/15/2/0/0:22	1.0	3–11	Apparent pK_a
DLin-KC2-DMA_C	48:144:48	11903:0:48/48/46/34/9/3/0/0/0:12	1.0	3–11	Apparent pK_a
SM-102_C	48:144:48	11317:0:48/48/48/45/30/9/4/1/0:26	1.0	3–11	Apparent pK_a
ALC-0315_C	48:144:48	11833:0:34/13/4/2/0/0/0/0/0:18	1.0	3–11	Apparent pK_a
DLin-MC3-DMA_C	48:96:96	11903:0:47/36/11/1/0:20	1.0	6–10	Apparent pK_a
DLin-KC2-DMA_C	48:96:96	11903:0:48/43/33/12/1:14	1.0	4–8	Apparent pK_a
SM-102_C	48:96:96	11855:0:47/42/24/8/4:22	1.0	5–9	Apparent pK_a
ALC-0315_C	48:96:96	11855:0:46/32/17/2/0:22	1.0	5–9	Apparent pK_a

Table S4: All-atom molecular dynamics simulations of ternary membranes including the helperlipid DOPC using the constant-pH method. The simulations were started with a different initialization by inserting all aminolipids in a deprotonated state into the membrane core.

System	Aminolipid:DOPC:Chol	H_2O:Na^+:Cl^-:Buf	Sim. Time (μs)	pH	Objective
SM-102	48:144:48	11903:0:0:97	2.0	3	Core initialization
SM-102	48:144:48	11903:0:0:97	2.0	5	Core initialization
SM-102	48:144:48	11910:0:0:90	2.0	7	Core initialization
SM-102	48:144:48	11972:0:0:28	2.0	9	Core initialization
ALC-0315	48:144:48	11903:0:0:97	2.0	3	Core initialization
ALC-0315	48:144:48	11903:0:0:97	2.0	5	Core initialization
ALC-0315	48:144:48	11943:0:0:57	2.0	7	Core initialization
ALC-0315	48:144:48	11980:0:0:20	2.0	9	Core initialization
SM-102	48:96:96	11903:0:0:97	2.0	3	Core initialization
SM-102	48:96:96	11903:0:0:97	2.0	5	Core initialization
SM-102	48:96:96	11920:0:0:80	2.0	7	Core initialization
SM-102	48:96:96	11974:0:0:26	2.0	9	Core initialization
ALC-0315	48:96:96	11903:0:0:97	2.0	3	Core initialization
ALC-0315	48:96:96	11903:0:0:97	2.0	5	Core initialization
ALC-0315	48:96:96	11949:0:0:51	2.0	7	Core initialization
ALC-0315	48:96:96	11979:0:0:21	2.0	9	Core initialization

Supplementary Figures

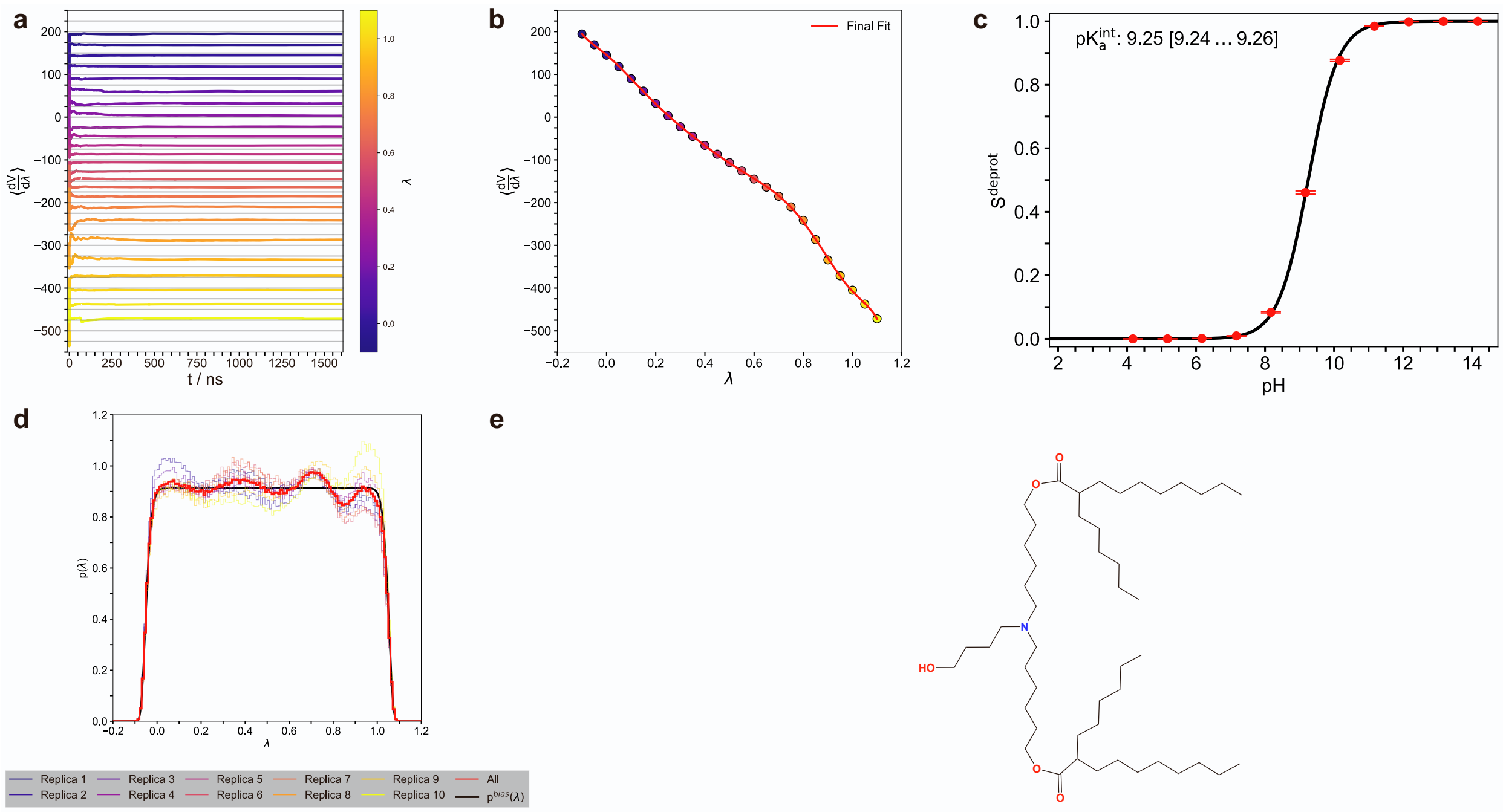

Figure S1: **a, b** Parameterization and **c, d** validation of polynomial coefficients used to model the correction potential for the protonation free energy of the **e** aminolipid ALC-0315. **a** Convergence of the estimated averages for $\frac{\partial V^{MM}}{\partial \lambda}$ obtained from thermodynamic integration simulations. **b** Fit of a ninth-order polynomial to the estimates of $\langle \frac{\partial V^{MM}}{\partial \lambda} \rangle$. **c** Titration curve of the aminolipid at infinite dilution, fitted with the Henderson-Hasselbalch equation. Error bars represent the standard error of the mean over ten replicas per pH level. The λ-dependent potentials $V^{bias}(\lambda)$ (barrier height 7.5 kJ·mol^{-1}), $V^{pH}(\lambda)$ (corresponding to the respective pH value), and the parameterized $V^{MM}(\lambda)$ were applied. **d** Distributions of the λ-coordinate obtained from ten replica simulations of the aminolipid at infinite dilution. In this case, only $V^{bias}(\lambda)$ (barrier height 0.0 kJ·mol^{-1}) and the parameterized $V^{MM}(\lambda)$ were applied. **e** Molecular structure of the aminolipid.

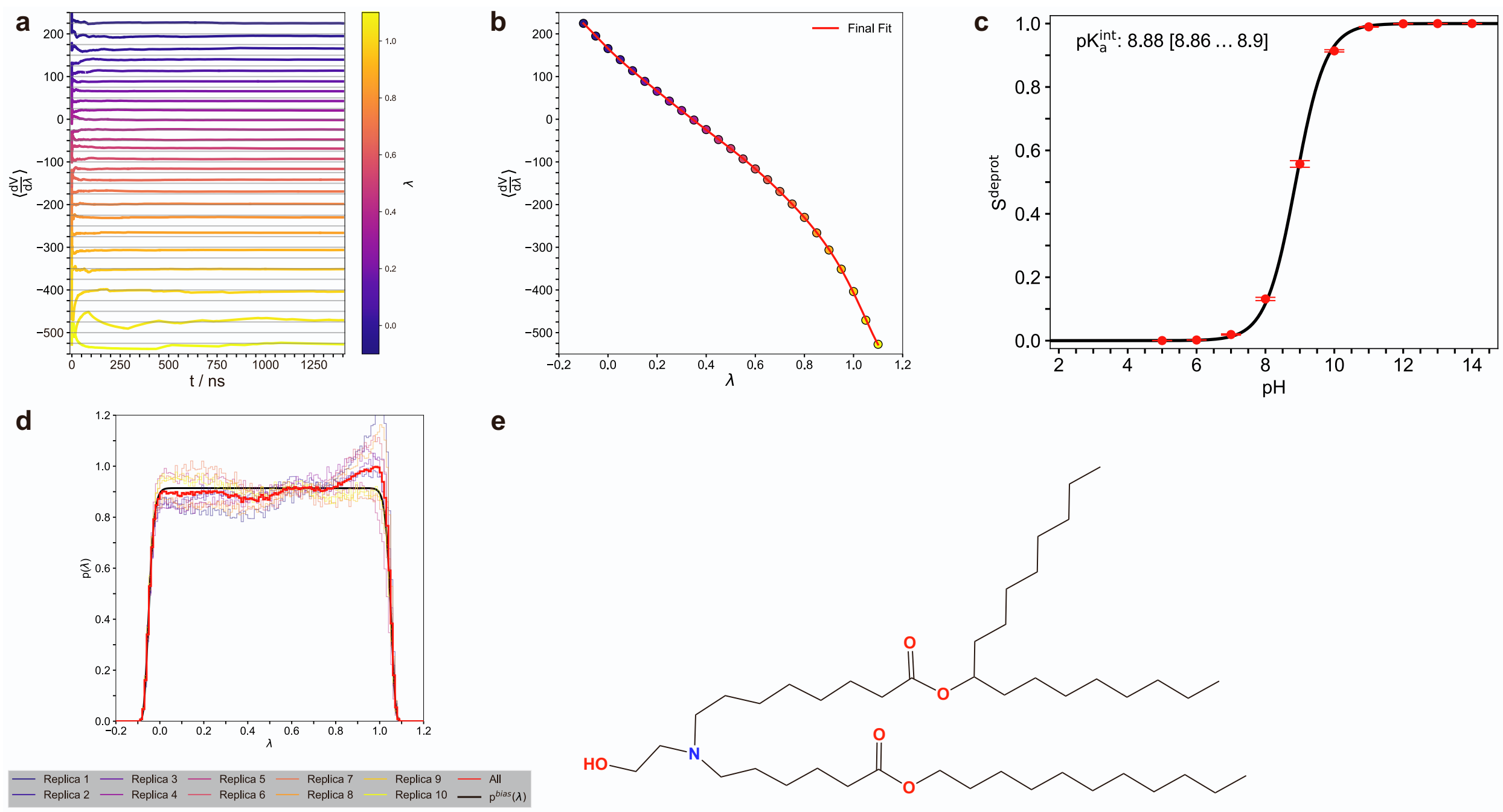

Figure S2: **a, b** Parameterization and **c, d** validation of polynomial coefficients used to model the correction potential for the protonation free energy of the **e** aminolipid SM-102. **a** Convergence of the estimated averages for $\frac{\partial V^{MM}}{\partial \lambda}$ obtained from thermodynamic integration simulations. **b** Fit of a ninth-order polynomial to the estimates of $\langle \frac{\partial V^{MM}}{\partial \lambda} \rangle$. **c** Titration curve of the aminolipid at infinite dilution, fitted with the Henderson-Hasselbalch equation. Error bars represent the standard error of the mean over ten replicas per pH level. The λ-dependent potentials $V^{bias}(\lambda)$ (barrier height 7.5 kJ·mol^{-1}), $V^{pH}(\lambda)$ (corresponding to the respective pH value), and the parameterized $V^{MM}(\lambda)$ were applied. **d** Distributions of the λ-coordinate obtained from ten replica simulations of the aminolipid at infinite dilution. In this case, only $V^{bias}(\lambda)$ (barrier height 0.0 kJ·mol^{-1}) and the parameterized $V^{MM}(\lambda)$ were applied. **e** Molecular structure of the aminolipid.

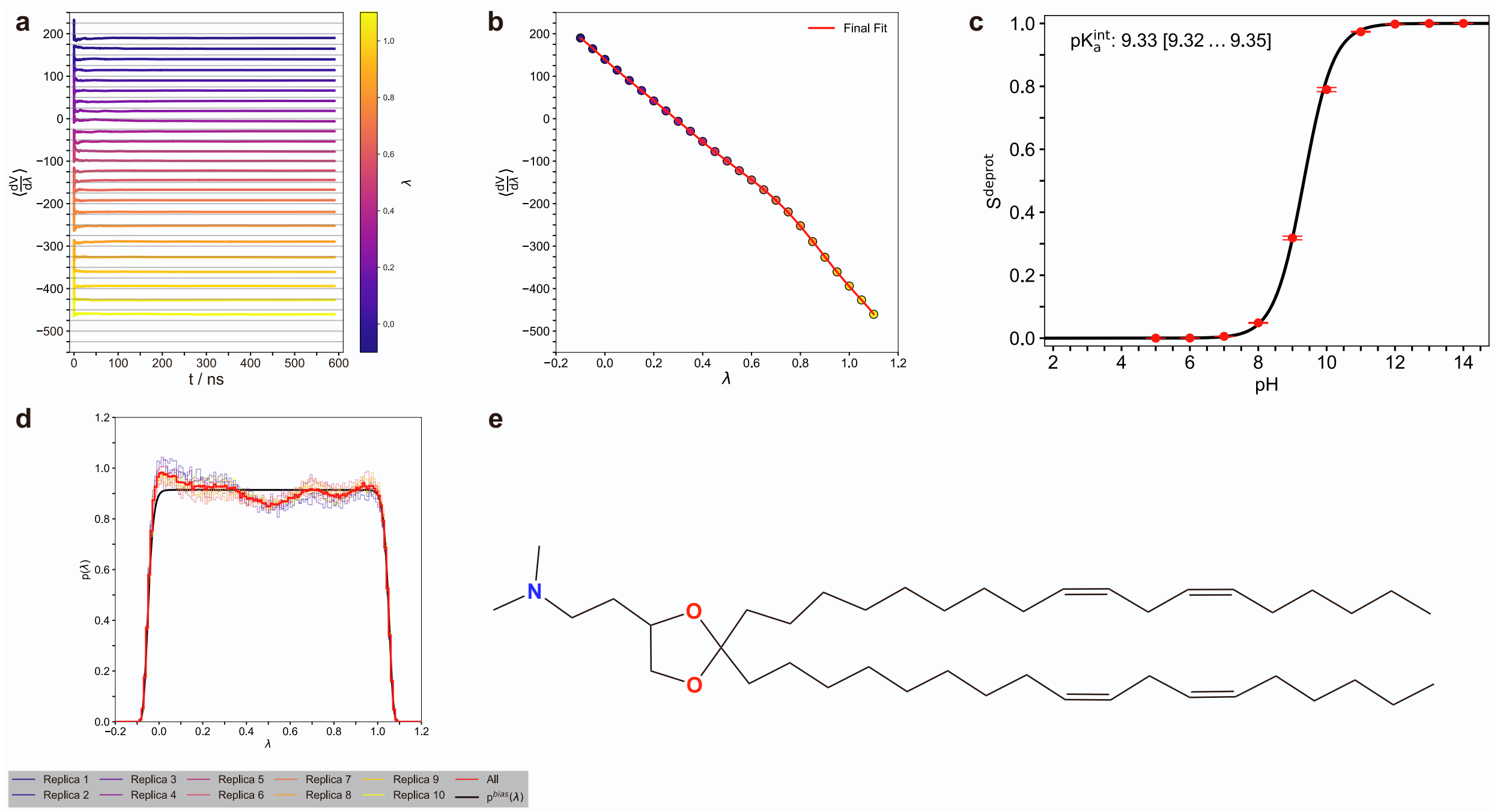

Figure S3: **a, b** Parameterization and **c, d** validation of polynomial coefficients used to model the correction potential for the protonation free energy of the **e** aminolipid DLin-KC2-DMA. **a** Convergence of the estimated averages for $\frac{\partial V^{MM}}{\partial \lambda}$ obtained from thermodynamic integration simulations. **b** Fit of an eighth-order polynomial to the estimates of $\langle \frac{\partial V^{MM}}{\partial \lambda} \rangle$. **c** Titration curve of the aminolipid at infinite dilution, fitted with the Henderson-Hasselbalch equation. Error bars represent the standard error of the mean over ten replicas per pH level. The λ-dependent potentials $V^{bias}(\lambda)$ (barrier height 7.5 kJ·mol^{-1}), $V^{pH}(\lambda)$ (corresponding to the respective pH value), and the parameterized $V^{MM}(\lambda)$ were applied. **d** Distributions of the λ-coordinate obtained from ten replica simulations of the aminolipid at infinite dilution. In this case, only $V^{bias}(\lambda)$ (barrier height 0.0 kJ·mol^{-1}) and the parameterized $V^{MM}(\lambda)$ were applied. **e** Molecular structure of the aminolipid.

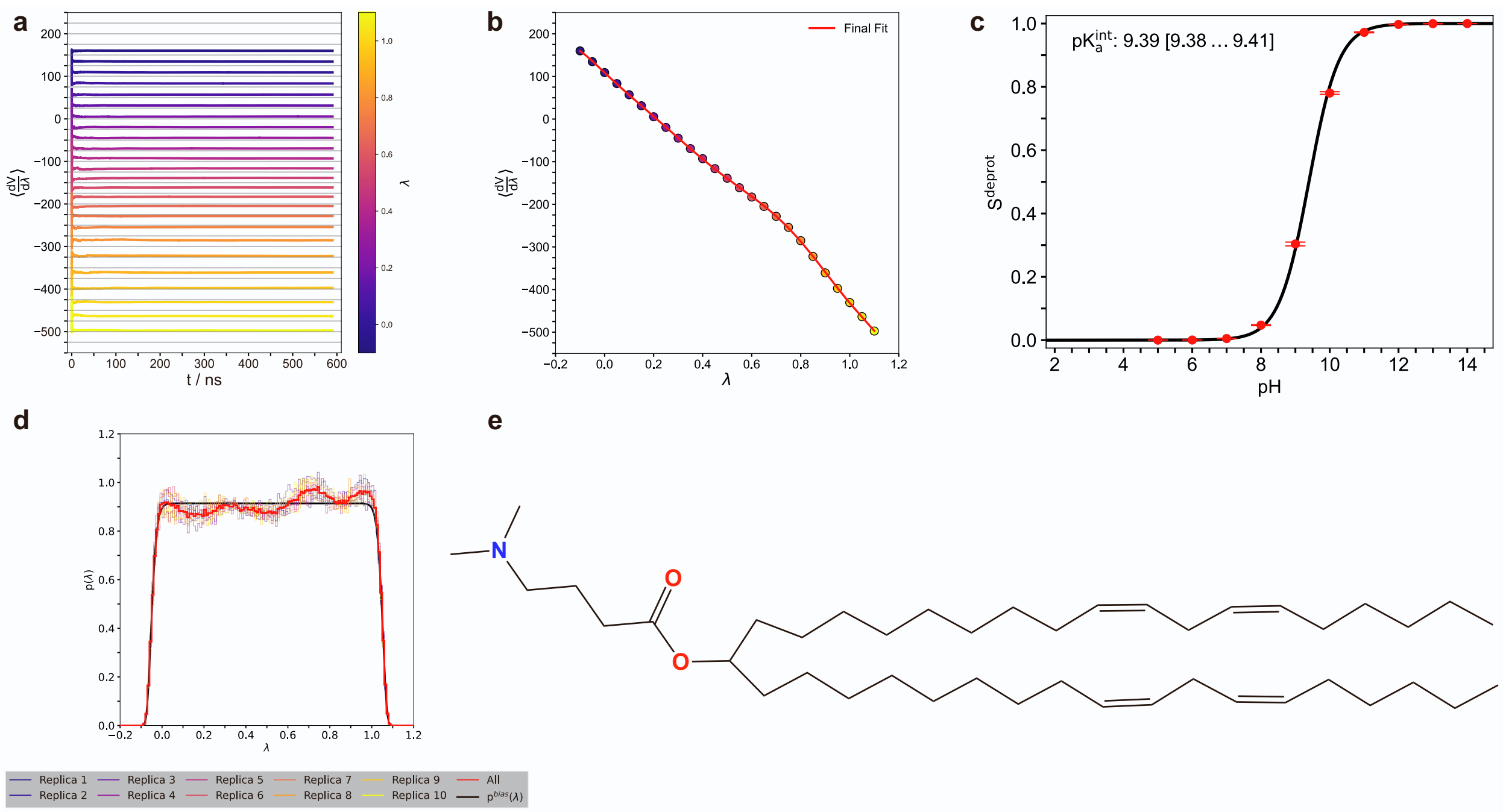

Figure S4: **a, b** Parameterization and **c, d** validation of polynomial coefficients used to model the correction potential for the protonation free energy of the **e** aminolipid DLin-MC3-DMA. **a** Convergence of the estimated averages for $\frac{\partial V^{MM}}{\partial \lambda}$ obtained from thermodynamic integration simulations. **b** Fit of an eighth-order polynomial to the estimates of $\langle \frac{\partial V^{MM}}{\partial \lambda} \rangle$. **c** Titration curve of the aminolipid at infinite dilution, fitted with the Henderson-Hasselbalch equation. Error bars represent the standard error of the mean over ten replicas per pH level. The λ-dependent potentials $V^{bias}(\lambda)$ (barrier height 7.5 kJ·mol^{-1}), $V^{pH}(\lambda)$ (corresponding to the respective pH value), and the parameterized $V^{MM}(\lambda)$ were applied. **d** Distributions of the λ-coordinate obtained from ten replica simulations of the aminolipid at infinite dilution. In this case, only $V^{bias}(\lambda)$ (barrier height 0.0 kJ·mol^{-1}) and the parameterized $V^{MM}(\lambda)$ were applied. **e** Molecular structure of the aminolipid.

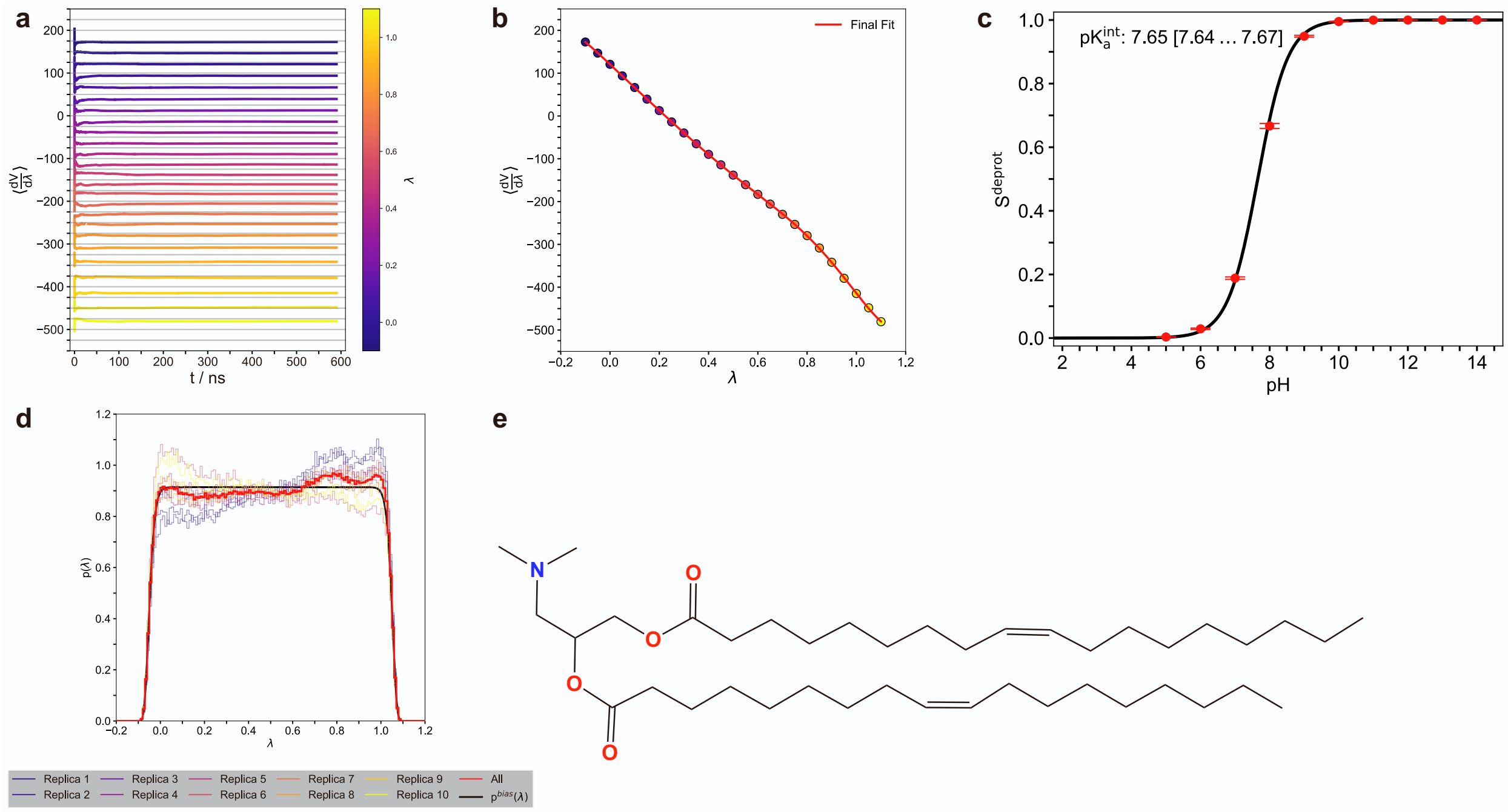

Figure S5: **a, b** Parameterization and **c, d** validation of polynomial coefficients used to model the correction potential for the protonation free energy of the **e** aminolipid DODAP. **a** Convergence of the estimated averages for $\frac{\partial V^{MM}}{\partial \lambda}$ obtained from thermodynamic integration simulations. **b** Fit of an eighth-order polynomial to the estimates of $\langle \frac{\partial V^{MM}}{\partial \lambda} \rangle$. **c** Titration curve of the aminolipid at infinite dilution, fitted with the Henderson-Hasselbalch equation. Error bars represent the standard error of the mean over ten replicas per pH level. The λ-dependent potentials $V^{bias}(\lambda)$ (barrier height 7.5 kJ·mol^{-1}), $V^{pH}(\lambda)$ (corresponding to the respective pH value), and the parameterized $V^{MM}(\lambda)$ were applied. **d** Distributions of the λ-coordinate obtained from ten replica simulations of the aminolipid at infinite dilution. In this case, only $V^{bias}(\lambda)$ (barrier height 0.0 kJ·mol^{-1}) and the parameterized $V^{MM}(\lambda)$ were applied. **e** Molecular structure of the aminolipid.

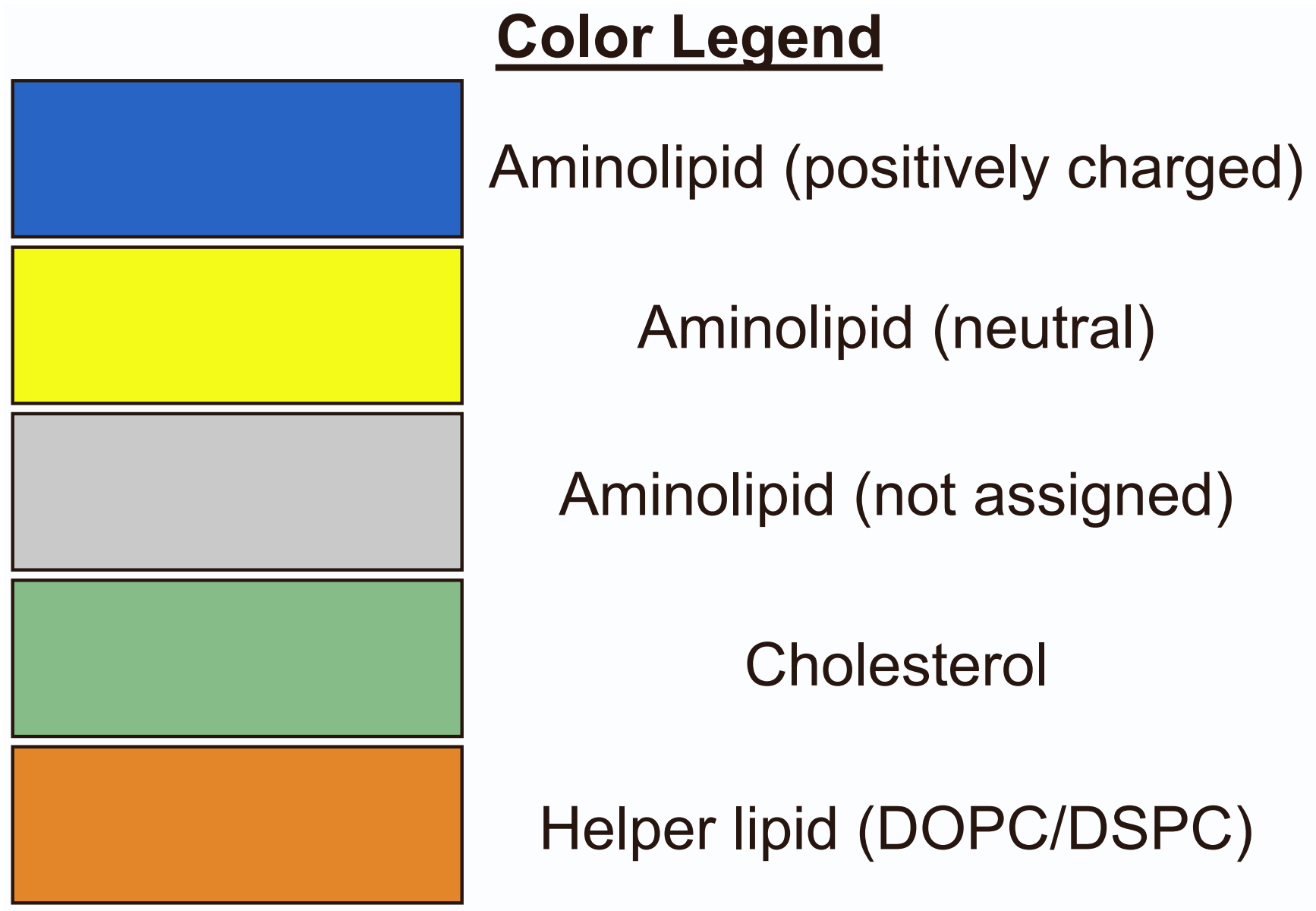

Figure S6: Color legend for all structural snapshots in the main text and Supporting Information. An aminolipid is classified as positively charged (blue) when its λ coordinate is below 0.2 (protonated) and as neutral (yellow) when λ exceeds 0.8 (deprotonated). For $0.2 \leq \lambda \leq 0.8$, the aminolipid is considered to be in a transition state (grey). Cholesterol is shown in green, and the helper lipids (DOPC/DSPC) in orange.

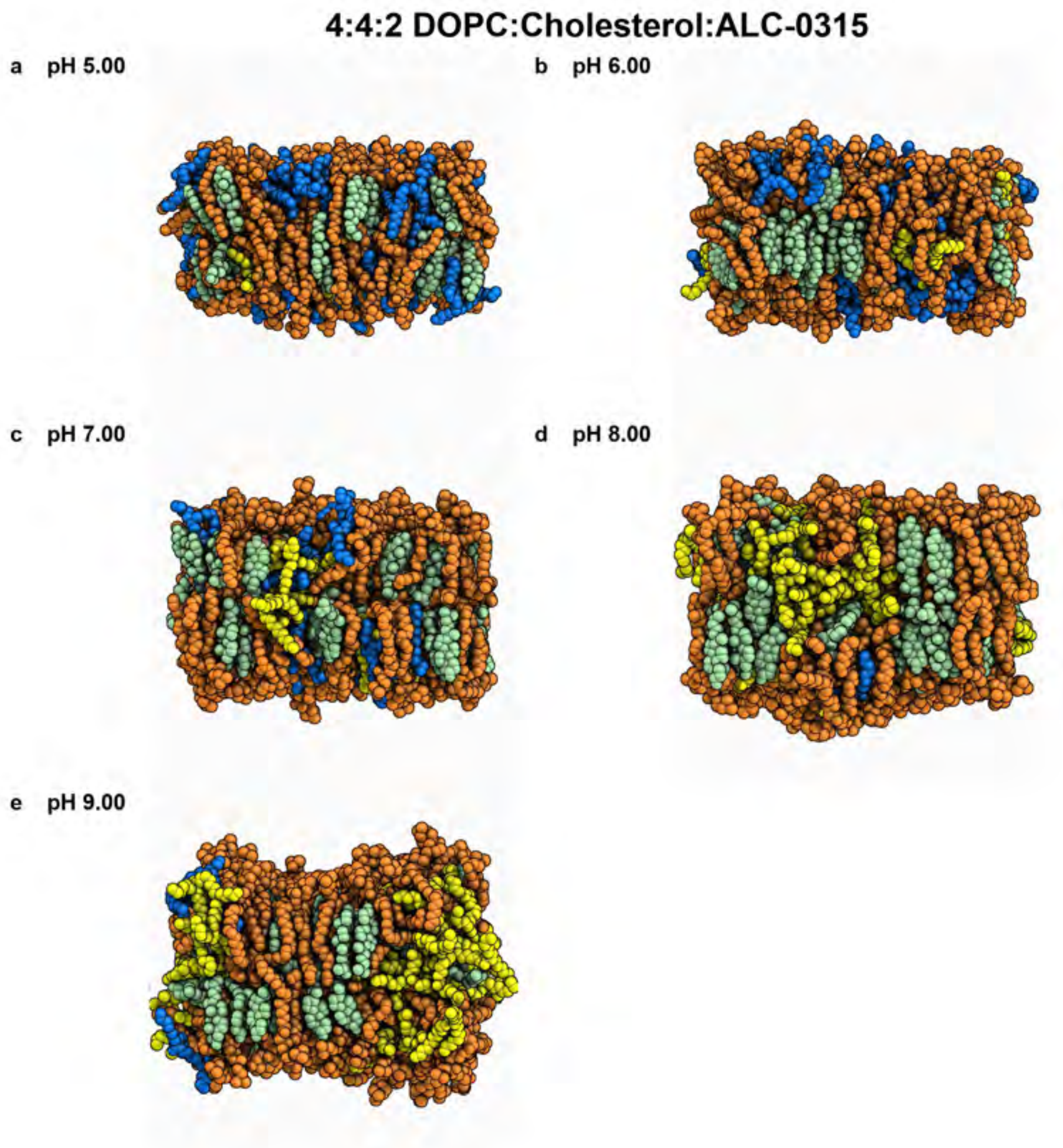

Figure S7: Side-view snapshots of the membrane in the final frame of the simulation at the corresponding pH values. The helper lipid is shown in orange, cholesterol in green, the protonated aminolipid (net charge +1) in blue, the deprotonated aminolipid (net charge 0) in yellow, and the aminolipids without a specific state ($0.2 \leq \lambda \leq 0.8$) in grey.

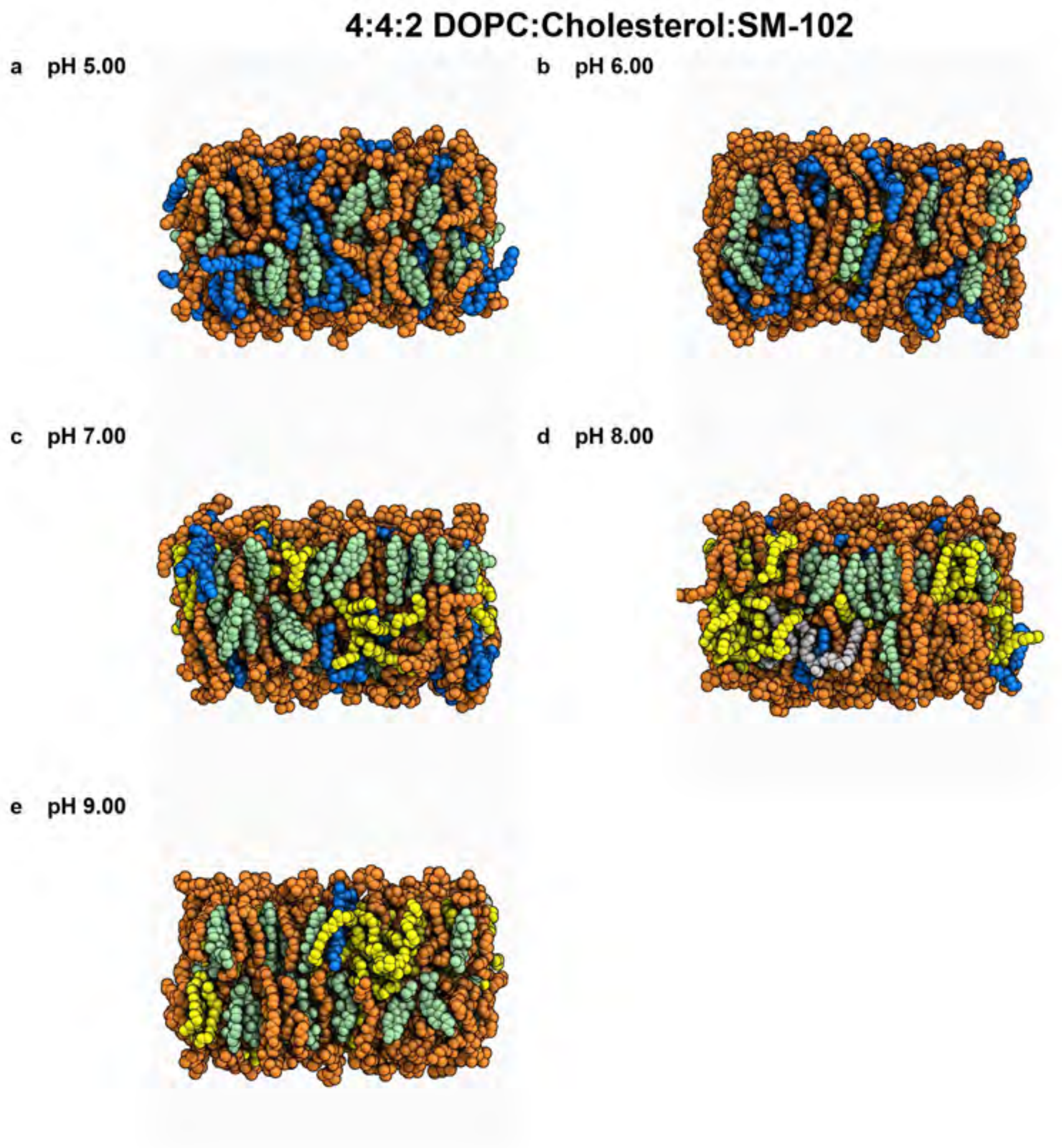

Figure S8: Side-view snapshots of the membrane in the final frame of the simulation at the corresponding pH values. The helper lipid is shown in orange, cholesterol in green, the protonated aminolipid (net charge +1) in blue, the deprotonated aminolipid (net charge 0) in yellow, and the aminolipids without a specific state ($0.2 \leq \lambda \leq 0.8$) in grey.

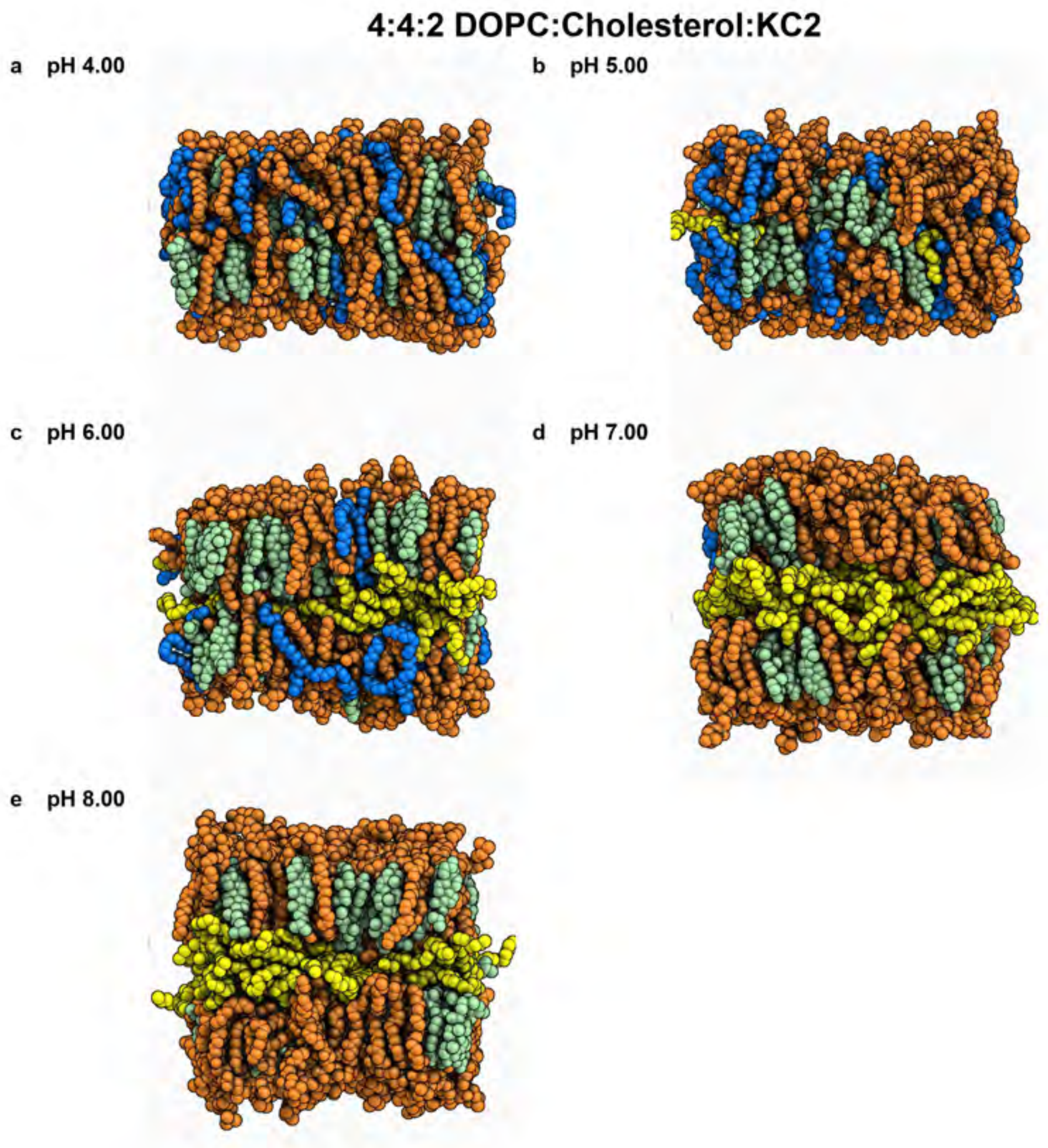

Figure S9: Side-view snapshots of the membrane in the final frame of the simulation at the corresponding pH values. The helper lipid is shown in orange, cholesterol in green, the protonated aminolipid (net charge +1) in blue, the deprotonated aminolipid (net charge 0) in yellow, and the aminolipids without a specific state ($0.2 \leq \lambda \leq 0.8$) in grey.

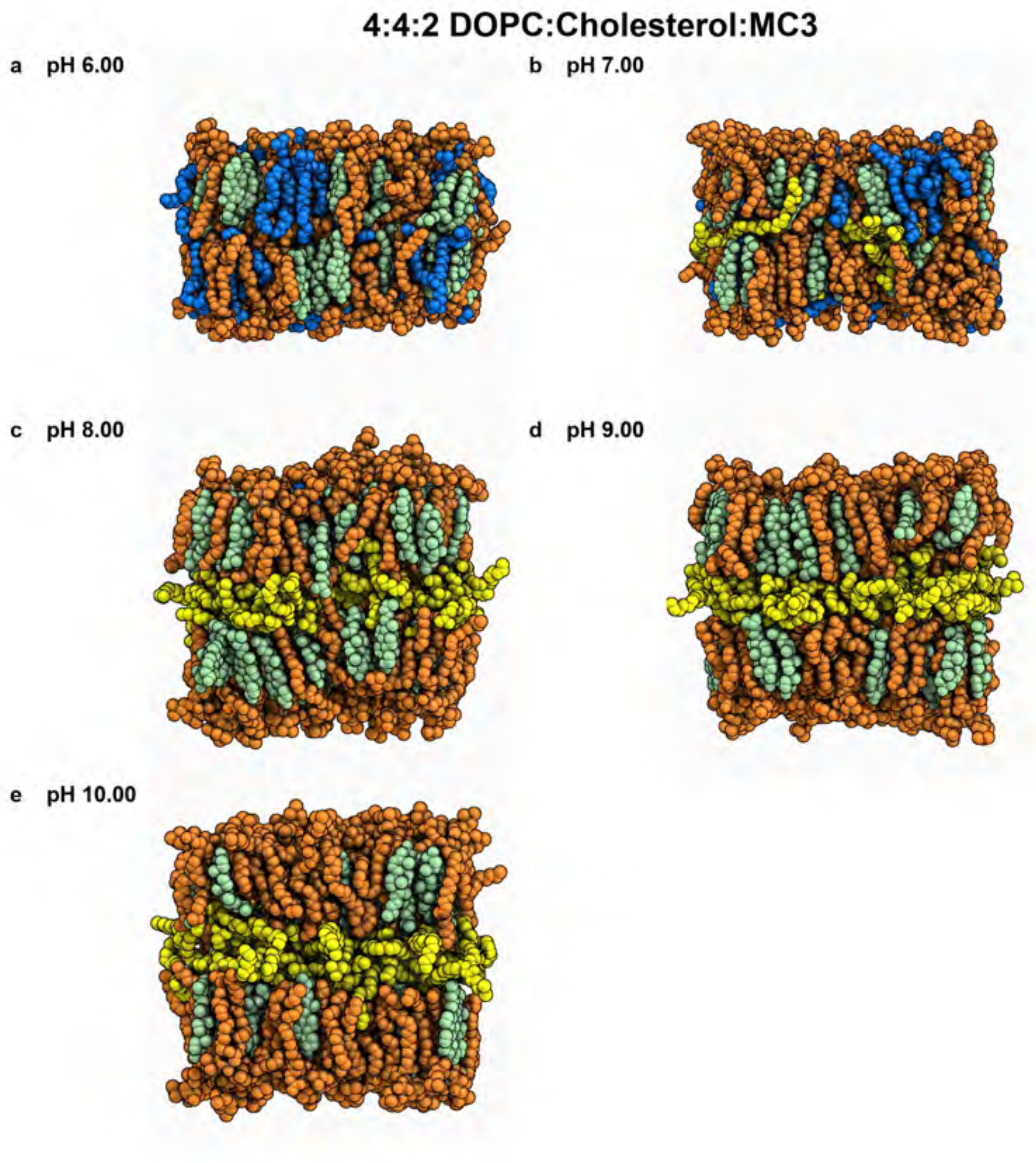

Figure S10: Side-view snapshots of the membrane in the final frame of the simulation at the corresponding pH values. The helper lipid is shown in orange, cholesterol in green, the protonated aminolipid (net charge +1) in blue, the deprotonated aminolipid (net charge 0) in yellow, and the aminolipids without a specific state ($0.2 \leq \lambda \leq 0.8$) in grey.

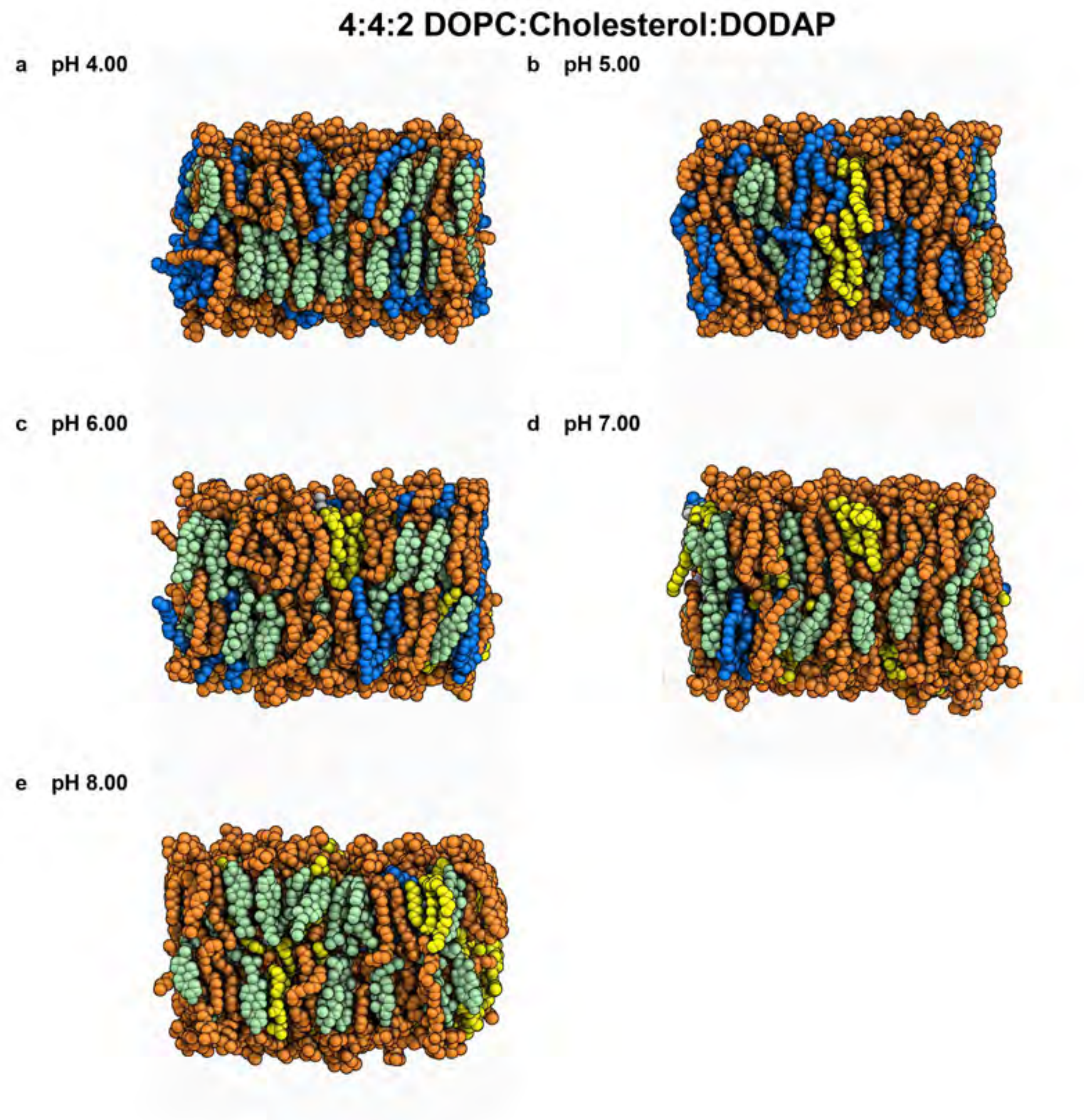

Figure S11: Side-view snapshots of the membrane in the final frame of the simulation at the corresponding pH values. The helper lipid is shown in orange, cholesterol in green, the protonated aminolipid (net charge +1) in blue, the deprotonated aminolipid (net charge 0) in yellow, and the aminolipids without a specific state ($0.2 \leq \lambda \leq 0.8$) in grey.

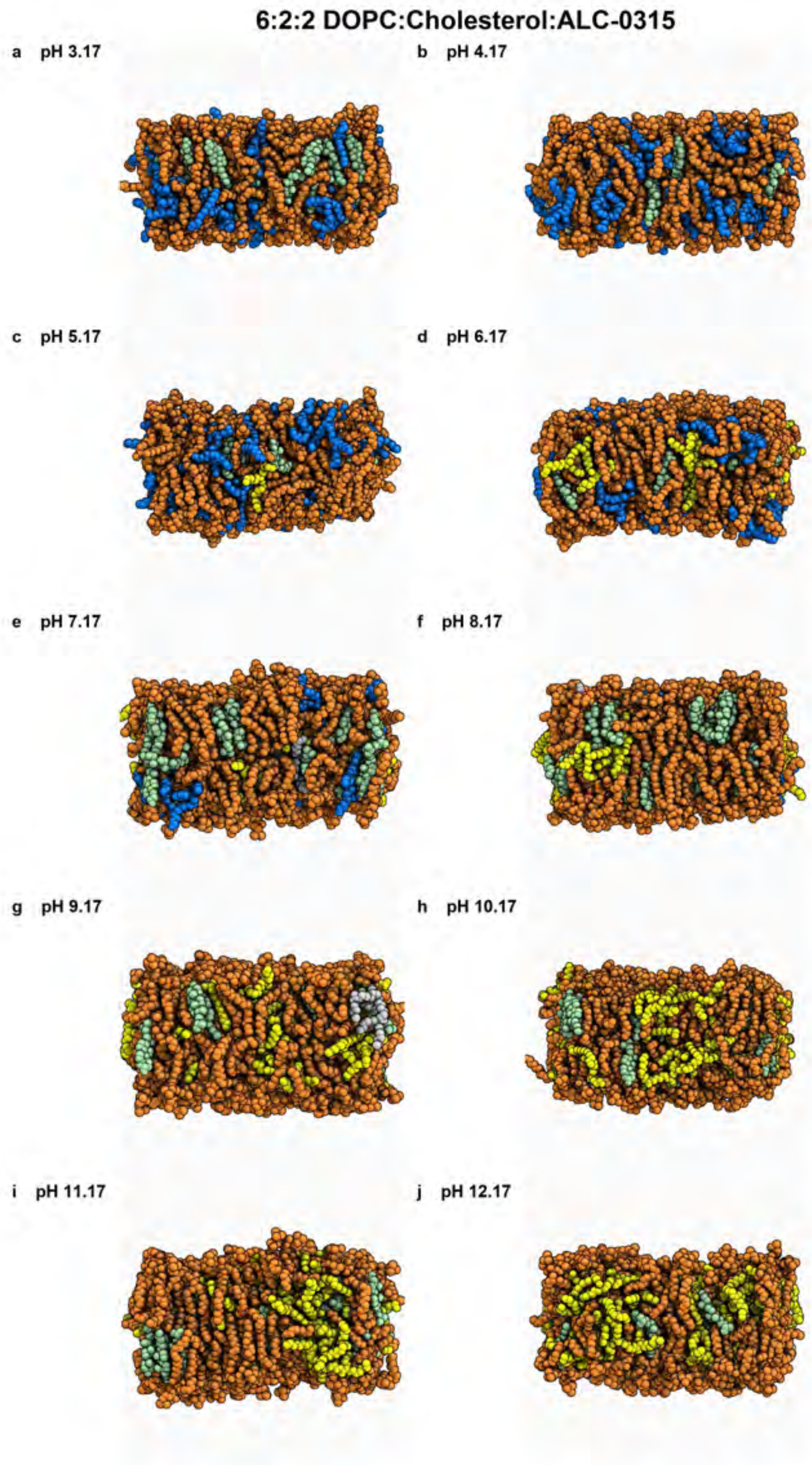

Figure S12: Side-view snapshots of the membrane in the final frame of the simulation at the corresponding pH values. The helper lipid is shown in orange, cholesterol in green, the protonated aminolipid (net charge +1) in blue, the deprotonated aminolipid (net charge 0) in yellow, and the aminolipids without a specific state ($0.2 \leq \lambda \leq 0.8$) in grey.

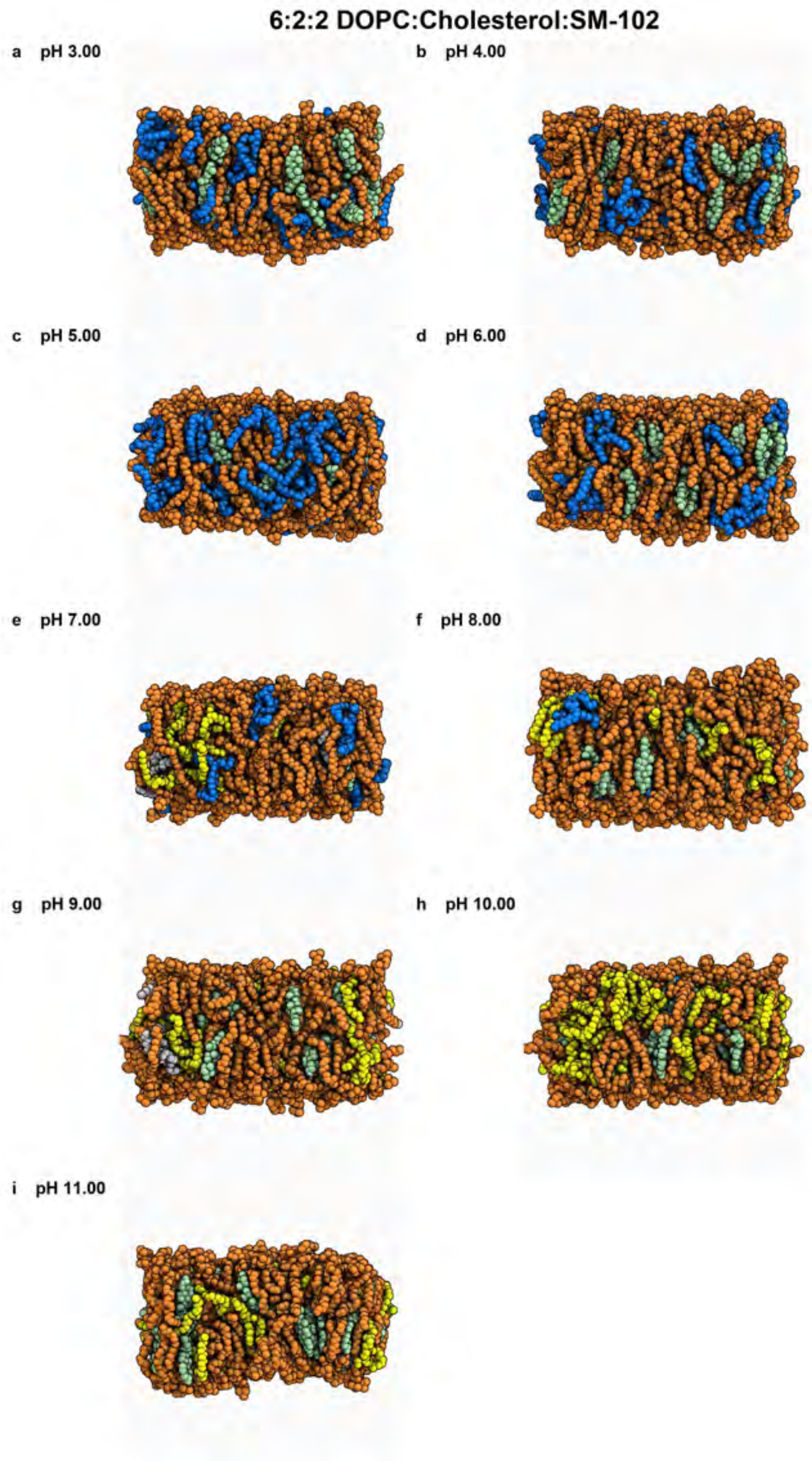

Figure S13: Side-view snapshots of the membrane in the final frame of the simulation at the corresponding pH values. The helper lipid is shown in orange, cholesterol in green, the protonated aminolipid (net charge +1) in blue, the deprotonated aminolipid (net charge 0) in yellow, and the aminolipids without a specific state ($0.2 \leq \lambda \leq 0.8$) in grey.

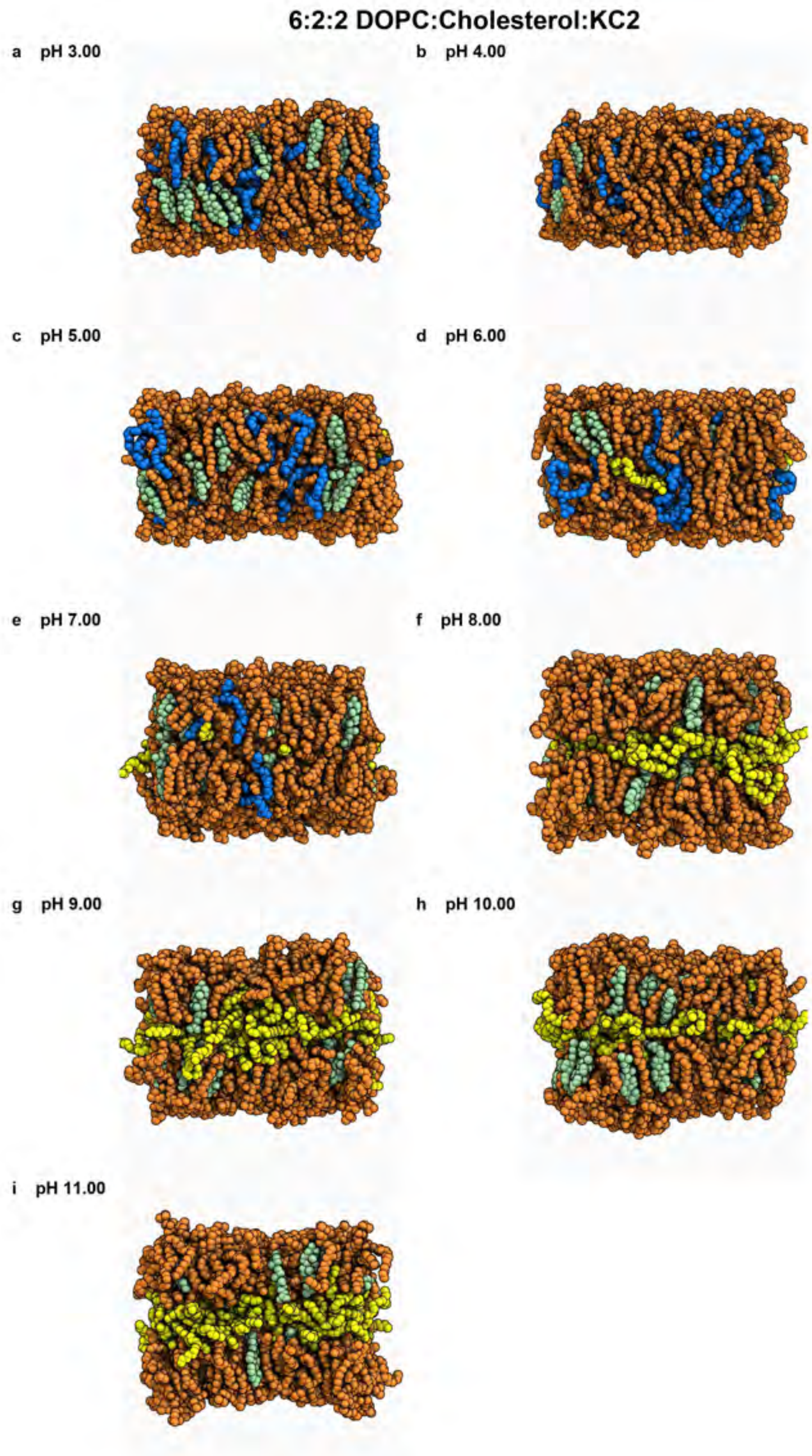

Figure S14: Side-view snapshots of the membrane in the final frame of the simulation at the corresponding pH values. The helper lipid is shown in orange, cholesterol in green, the protonated aminolipid (net charge +1) in blue, the deprotonated aminolipid (net charge 0) in yellow, and the aminolipids without a specific state ($0.2 \leq \lambda \leq 0.8$) in grey.

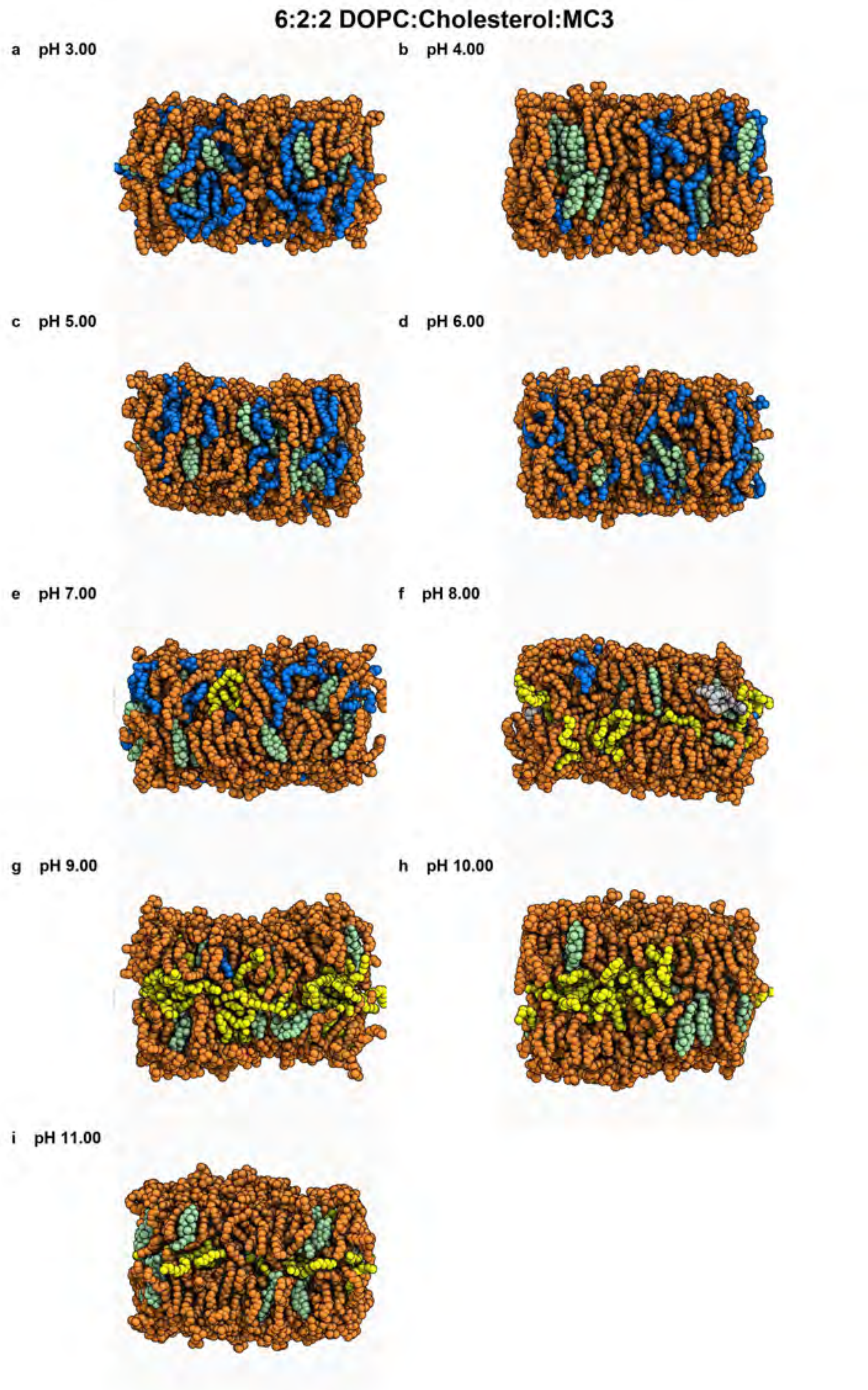

Figure S15: Side-view snapshots of the membrane in the final frame of the simulation at the corresponding pH values. The helper lipid is shown in orange, cholesterol in green, the protonated aminolipid (net charge +1) in blue, the deprotonated aminolipid (net charge 0) in yellow, and the aminolipids without a specific state ($0.2 \leq \lambda \leq 0.8$) in grey.

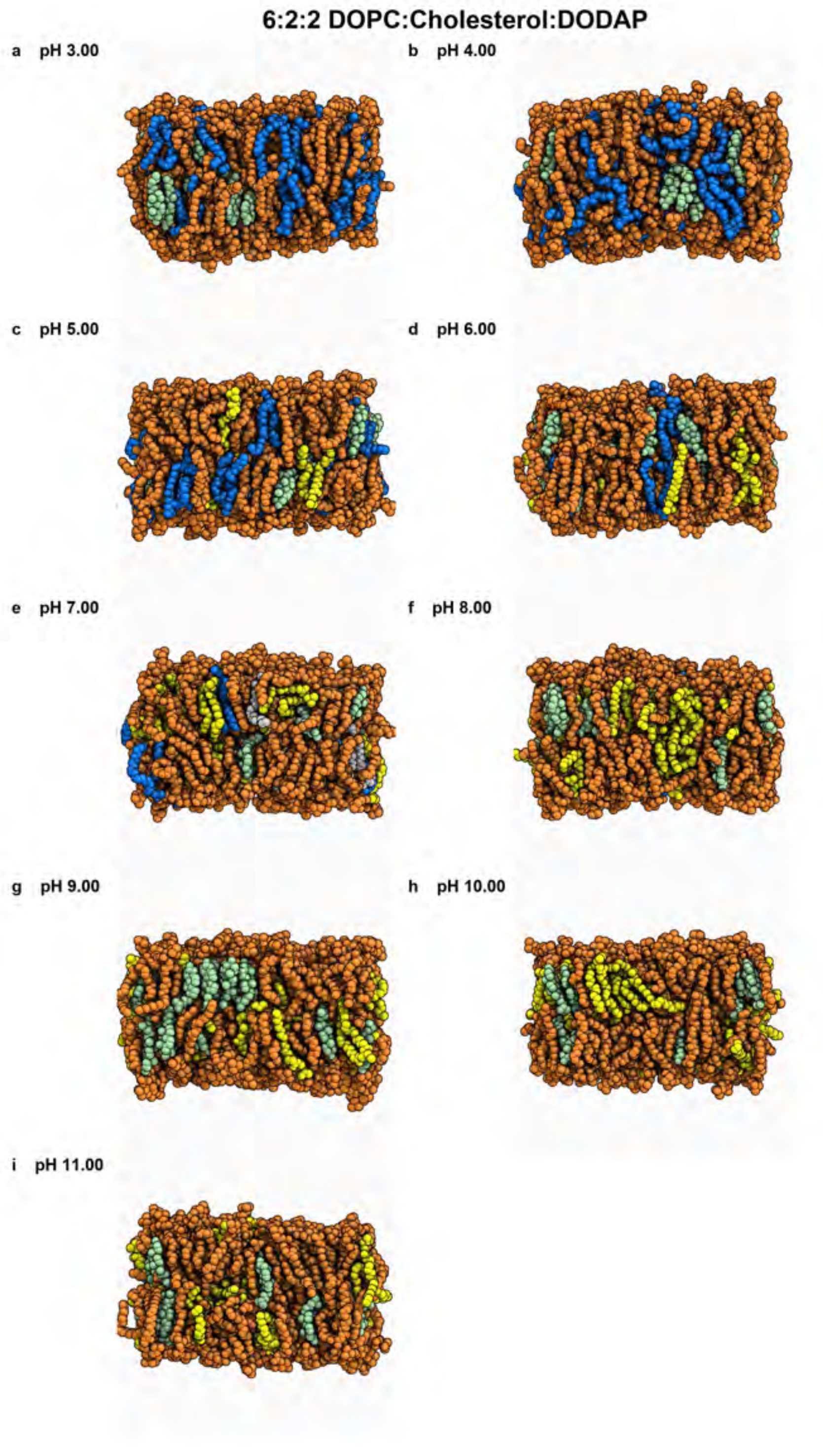

Figure S16: Side-view snapshots of the membrane in the final frame of the simulation at the corresponding pH values. The helper lipid is shown in orange, cholesterol in green, the protonated aminolipid (net charge +1) in blue, the deprotonated aminolipid (net charge 0) in yellow, and the aminolipids without a specific state ($0.2 \leq \lambda \leq 0.8$) in grey.

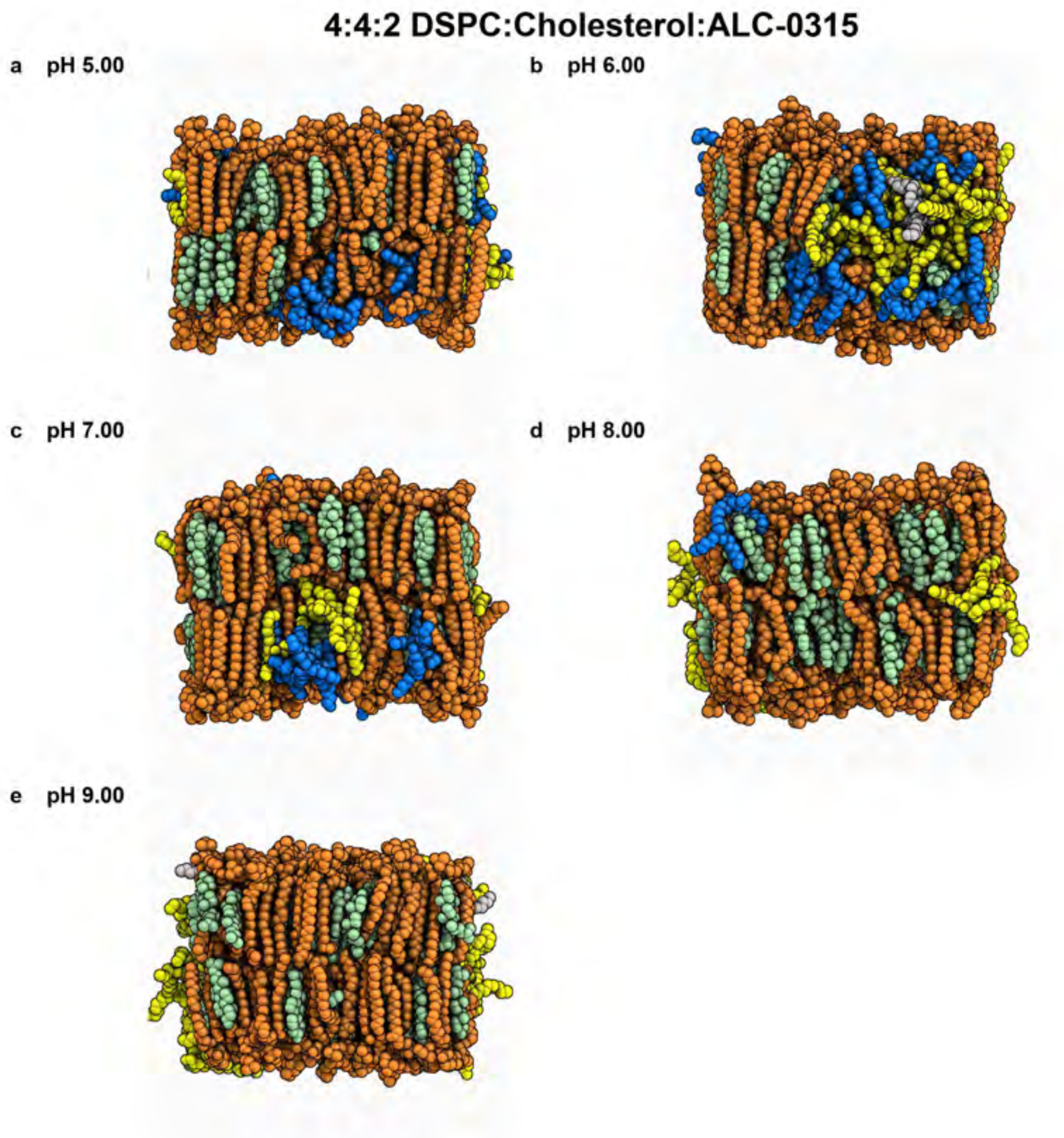

Figure S17: Side-view snapshots of the membrane in the final frame of the simulation at the corresponding pH values. The helper lipid is shown in orange, cholesterol in green, the protonated aminolipid (net charge +1) in blue, the deprotonated aminolipid (net charge 0) in yellow, and the aminolipids without a specific state ($0.2 \leq \lambda \leq 0.8$) in grey.

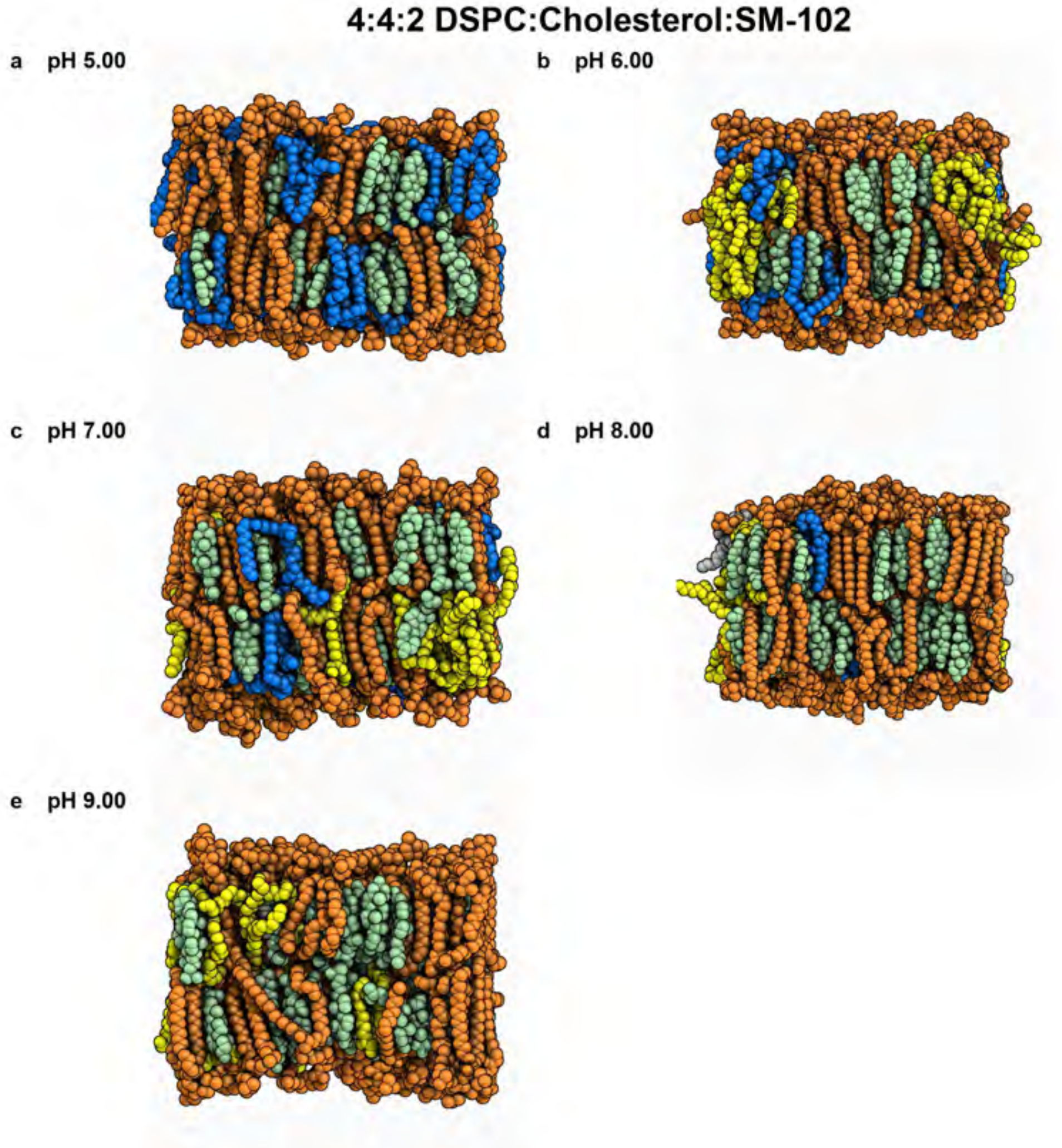

Figure S18: Side-view snapshots of the membrane in the final frame of the simulation at the corresponding pH values. The helper lipid is shown in orange, cholesterol in green, the protonated aminolipid (net charge +1) in blue, the deprotonated aminolipid (net charge 0) in yellow, and the aminolipids without a specific state ($0.2 \leq \lambda \leq 0.8$) in grey.

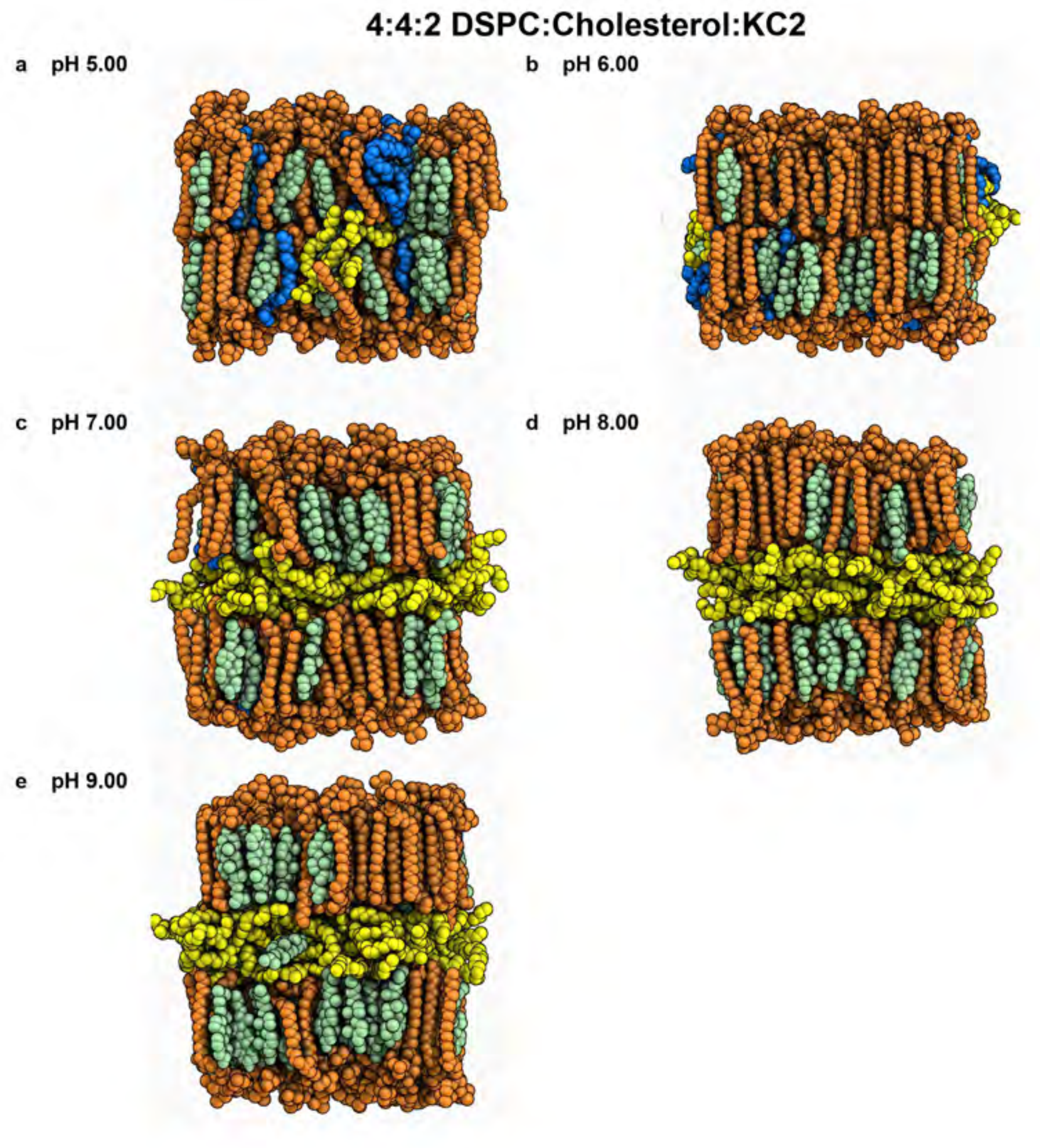

Figure S19: Side-view snapshots of the membrane in the final frame of the simulation at the corresponding pH values. The helper lipid is shown in orange, cholesterol in green, the protonated aminolipid (net charge +1) in blue, the deprotonated aminolipid (net charge 0) in yellow, and the aminolipids without a specific state ($0.2 \leq \lambda \leq 0.8$) in grey.

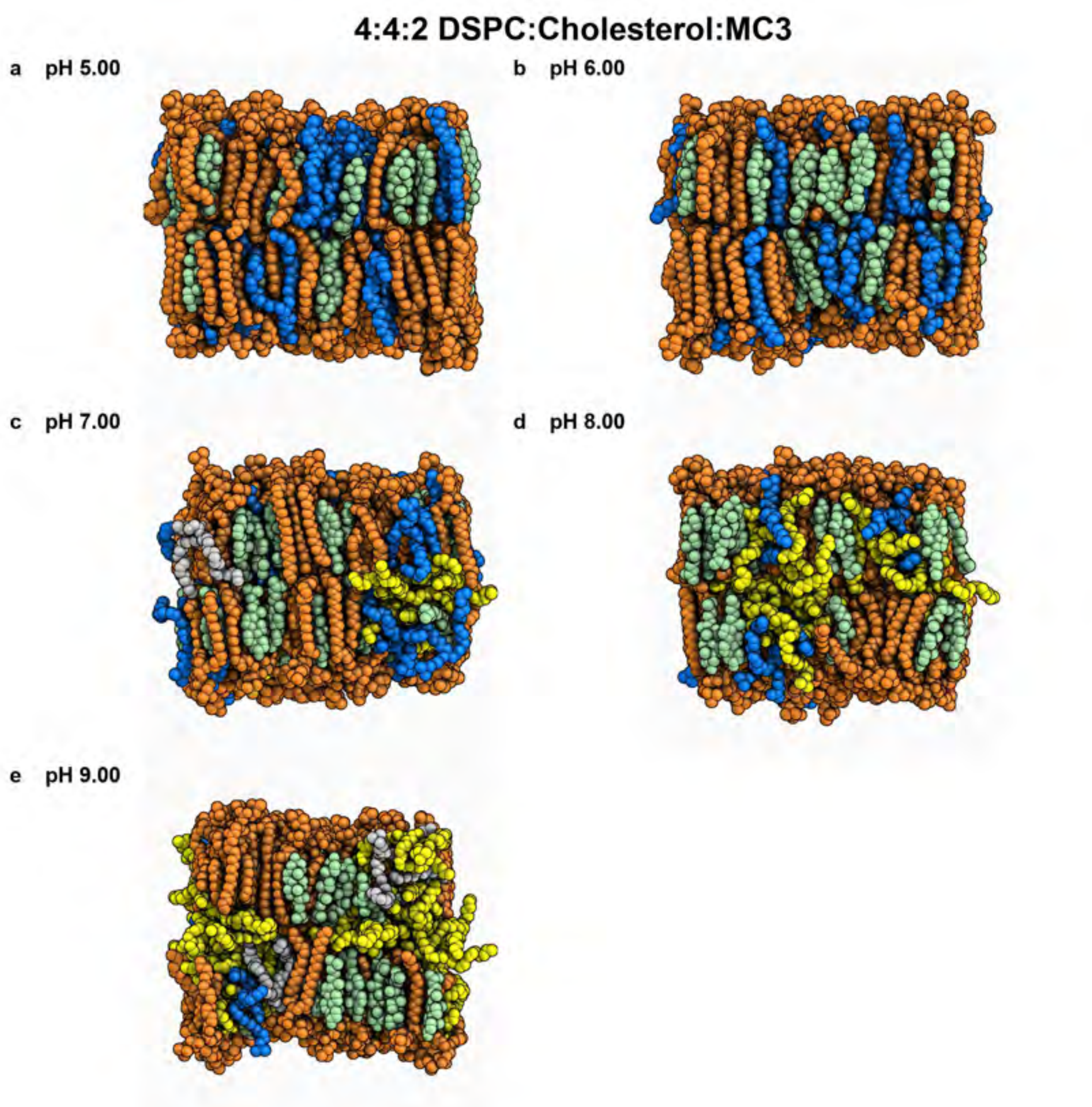

Figure S20: Side-view snapshots of the membrane in the final frame of the simulation at the corresponding pH values. The helper lipid is shown in orange, cholesterol in green, the protonated aminolipid (net charge +1) in blue, the deprotonated aminolipid (net charge 0) in yellow, and the aminolipids without a specific state ($0.2 \leq \lambda \leq 0.8$) in grey.

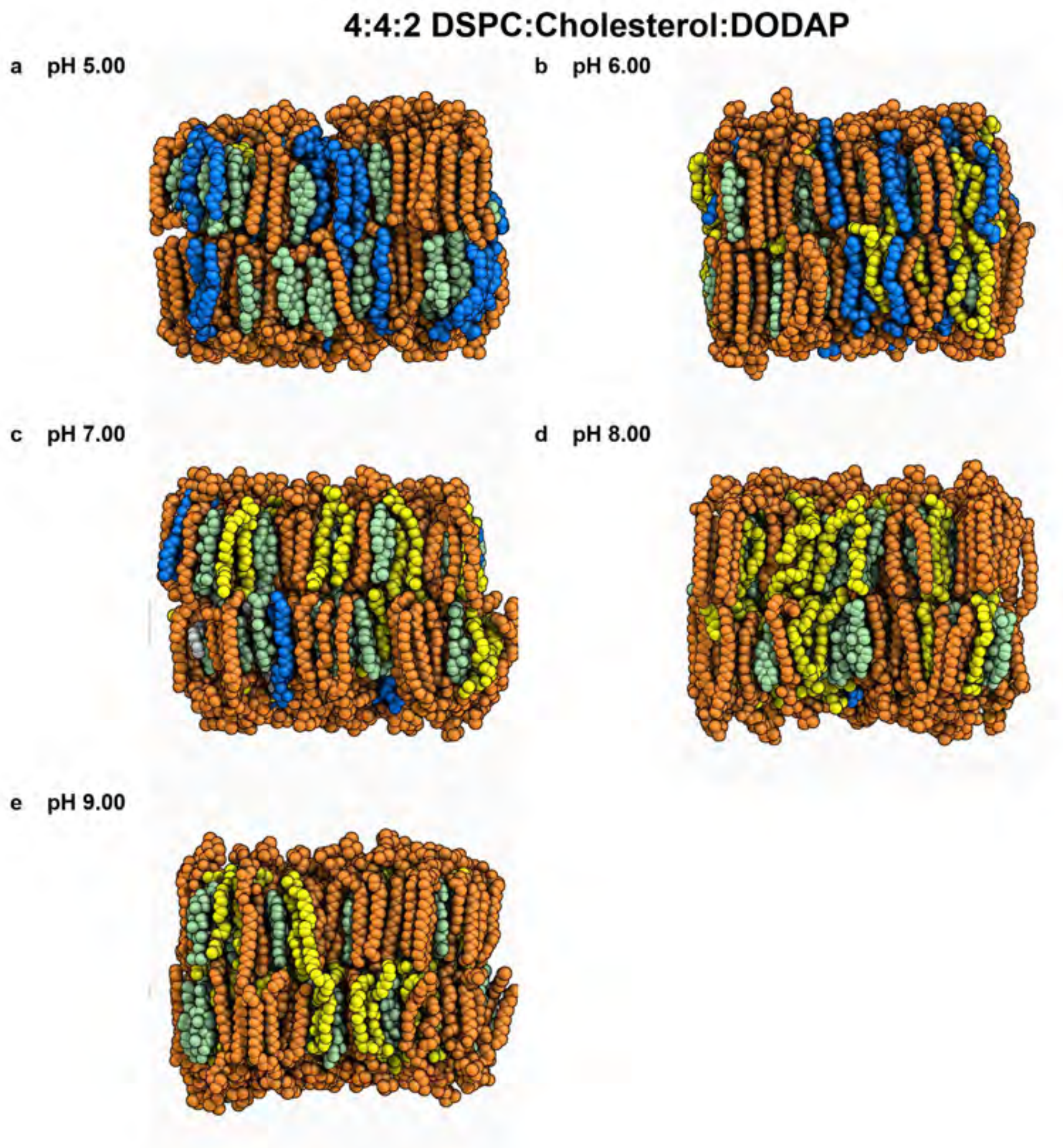

Figure S21: Side-view snapshots of the membrane in the final frame of the simulation at the corresponding pH values. The helper lipid is shown in orange, cholesterol in green, the protonated aminolipid (net charge +1) in blue, the deprotonated aminolipid (net charge 0) in yellow, and the aminolipids without a specific state ($0.2 \leq \lambda \leq 0.8$) in grey.

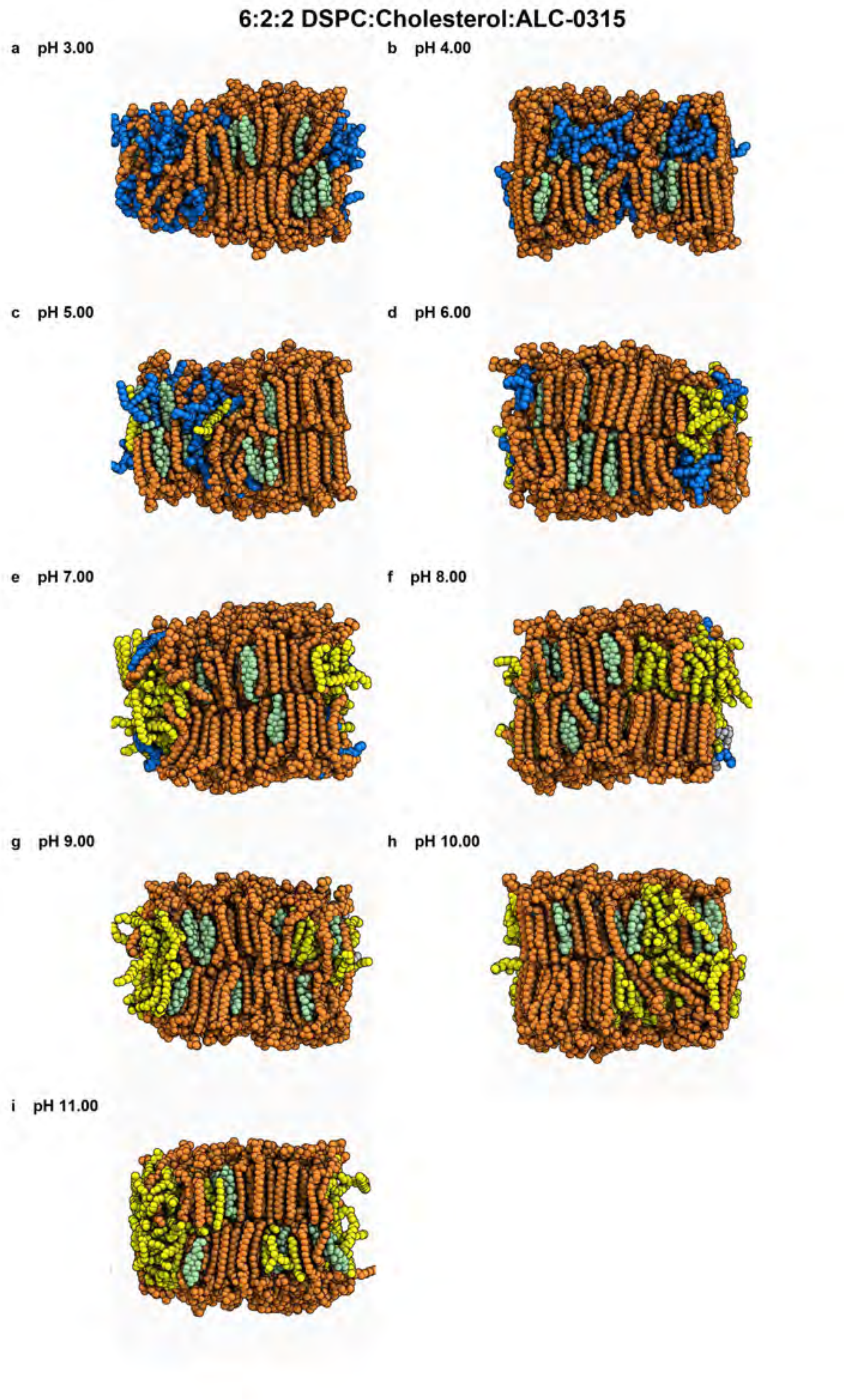

Figure S22: Side-view snapshots of the membrane in the final frame of the simulation at the corresponding pH values. The helper lipid is shown in orange, cholesterol in green, the protonated aminolipid (net charge +1) in blue, the deprotonated aminolipid (net charge 0) in yellow, and the aminolipids without a specific state ($0.2 \leq \lambda \leq 0.8$) in grey.

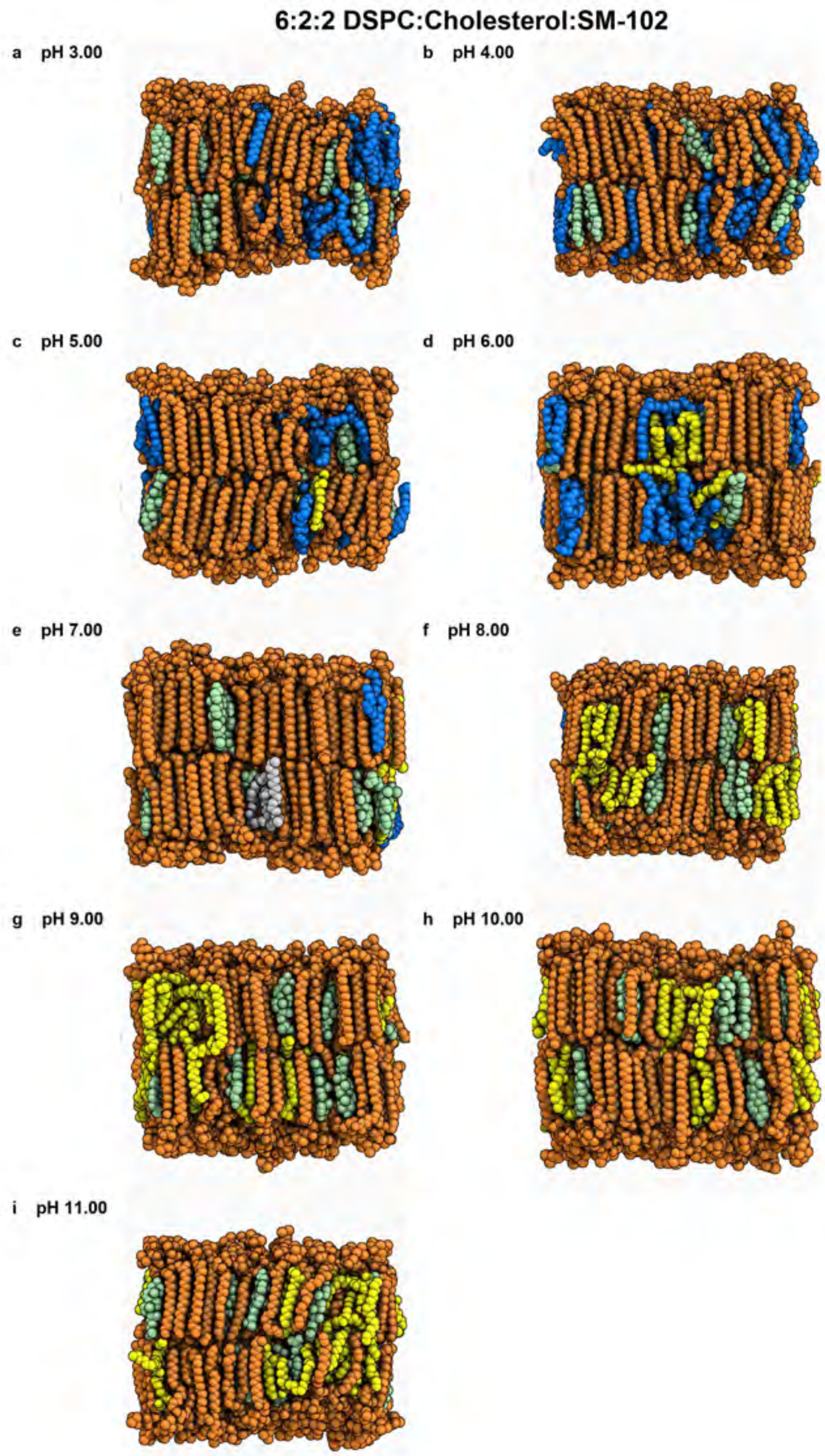

Figure S23: Side-view snapshots of the membrane in the final frame of the simulation at the corresponding pH values. The helper lipid is shown in orange, cholesterol in green, the protonated aminolipid (net charge +1) in blue, the deprotonated aminolipid (net charge 0) in yellow, and the aminolipids without a specific state ($0.2 \leq \lambda \leq 0.8$) in grey.

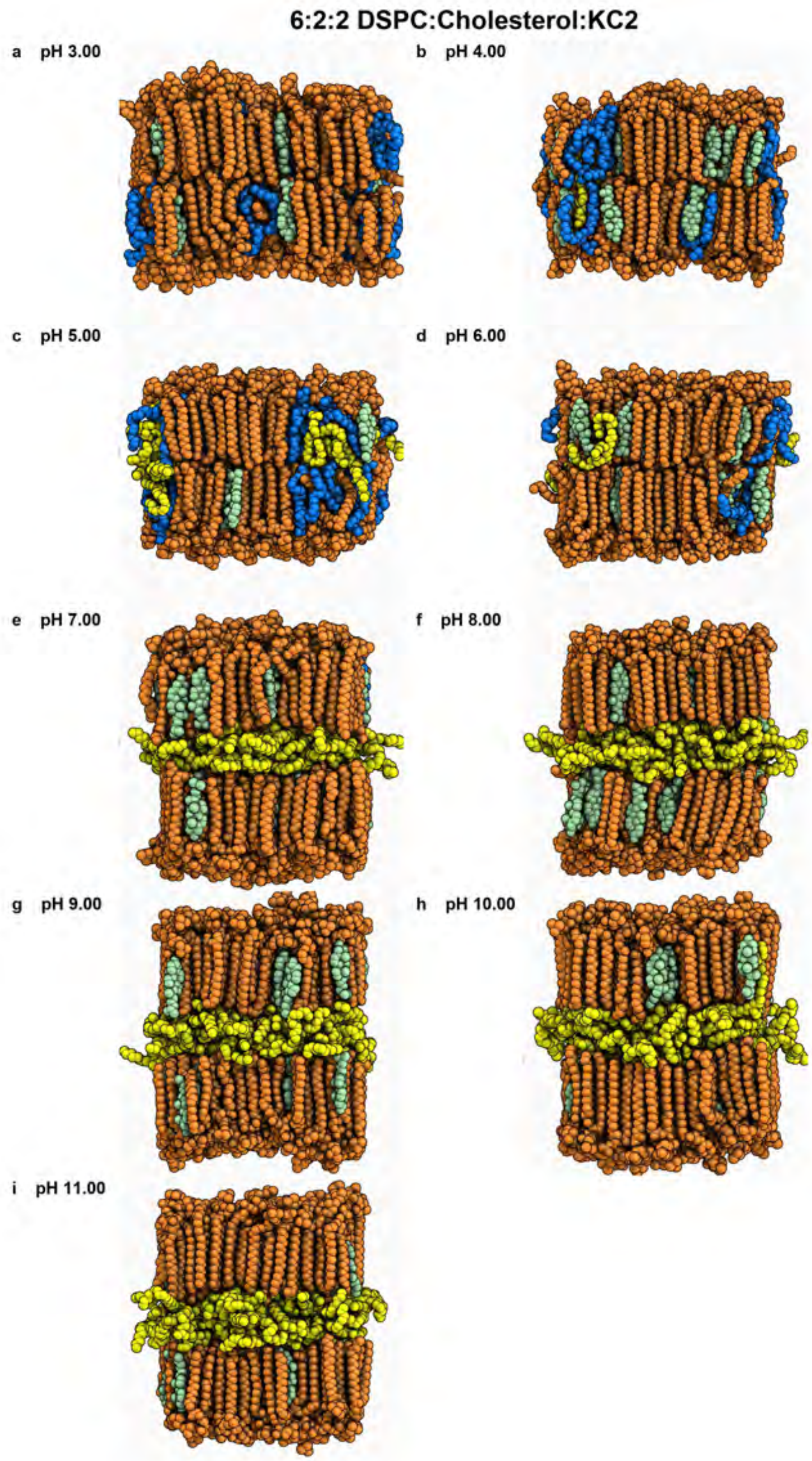

Figure S24: Side-view snapshots of the membrane in the final frame of the simulation at the corresponding pH values. The helper lipid is shown in orange, cholesterol in green, the protonated aminolipid (net charge +1) in blue, the deprotonated aminolipid (net charge 0) in yellow, and the aminolipids without a specific state ($0.2 \leq \lambda \leq 0.8$) in grey.

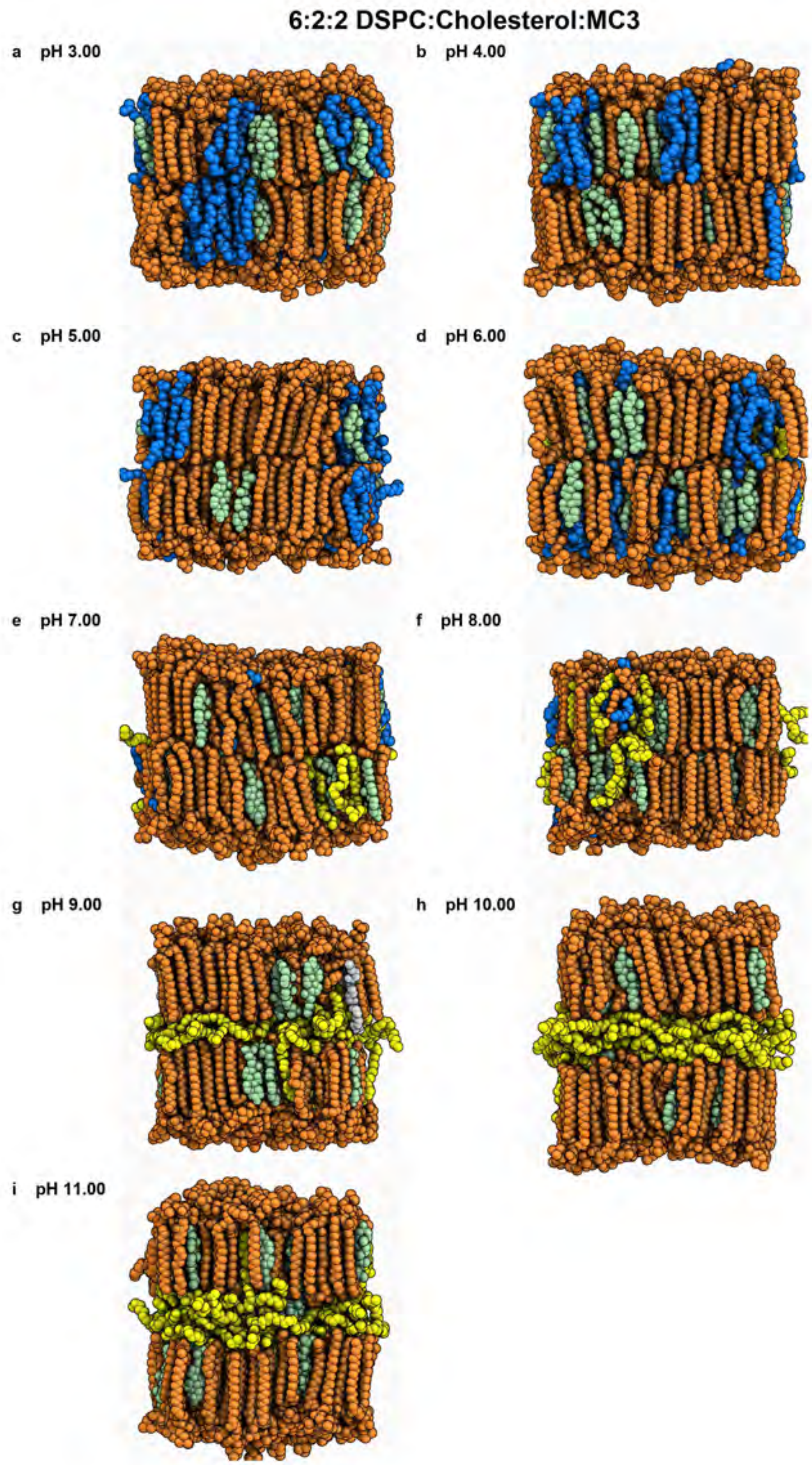

Figure S25: Side-view snapshots of the membrane in the final frame of the simulation at the corresponding pH values. The helper lipid is shown in orange, cholesterol in green, the protonated aminolipid (net charge +1) in blue, the deprotonated aminolipid (net charge 0) in yellow, and the aminolipids without a specific state ($0.2 \leq \lambda \leq 0.8$) in grey.

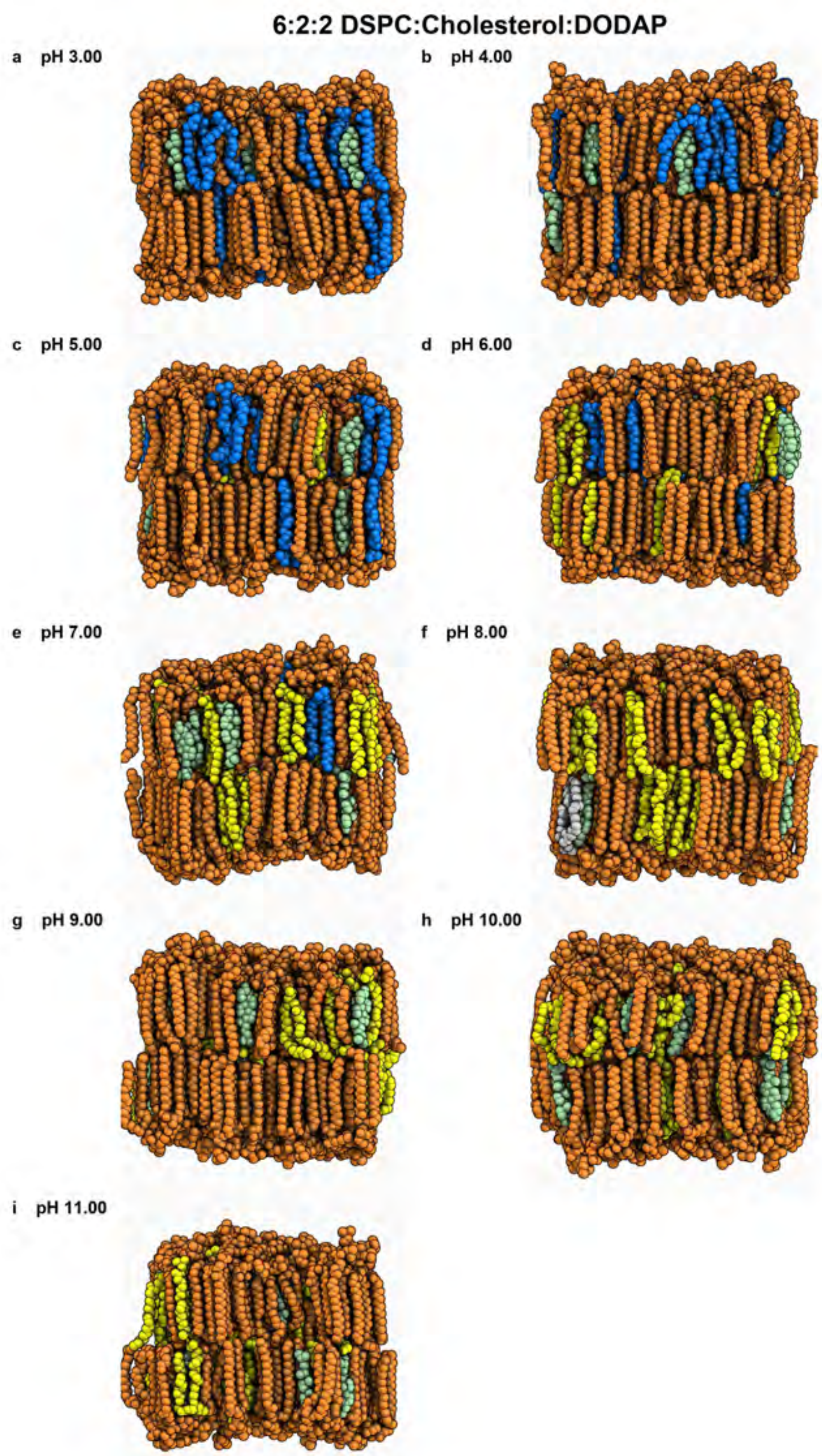

Figure S26: Side-view snapshots of the membrane in the final frame of the simulation at the corresponding pH values. The helper lipid is shown in orange, cholesterol in green, the protonated aminolipid (net charge +1) in blue, the deprotonated aminolipid (net charge 0) in yellow, and the aminolipids without a specific state ($0.2 \leq \lambda \leq 0.8$) in grey.

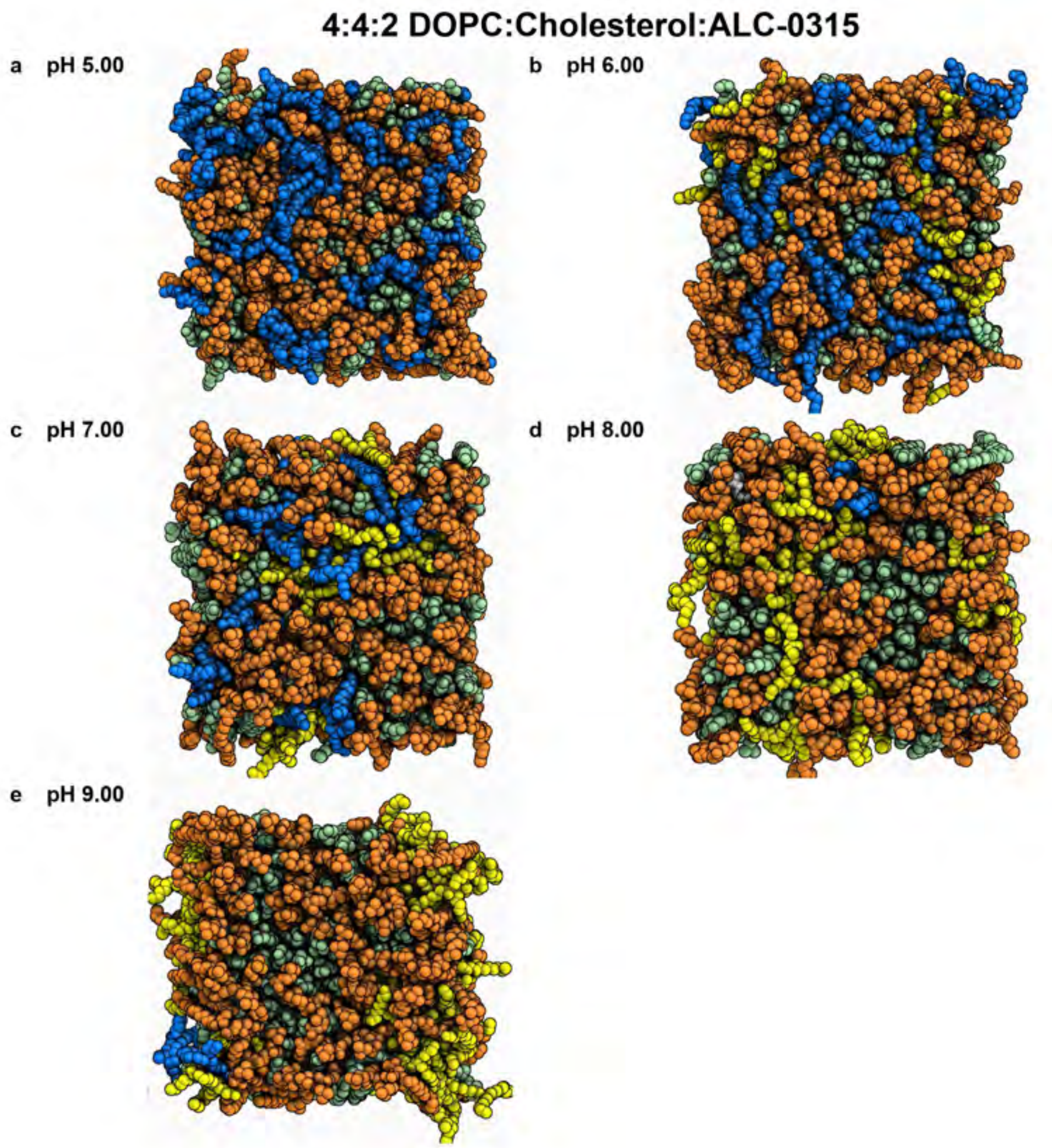

Figure S27: Top-view snapshots of the membrane in the final frame of the simulation at the corresponding pH values. The helper lipid is shown in orange, cholesterol in green, the protonated aminolipid (net charge +1) in blue, the deprotonated aminolipid (net charge 0) in yellow, and the aminolipids without a specific state ($0.2 \leq \lambda \leq 0.8$) in grey.

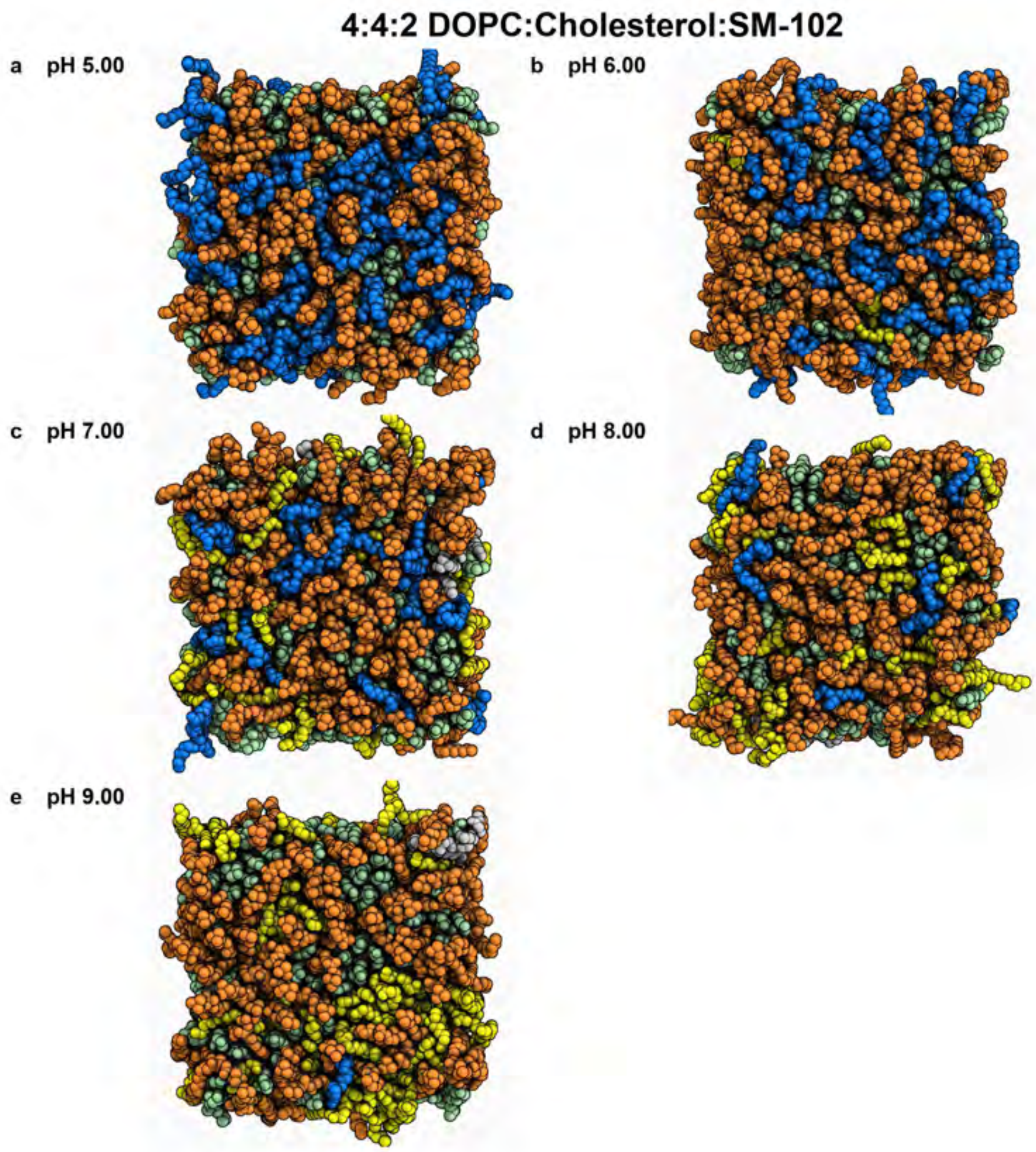

Figure S28: Top-view snapshots of the membrane in the final frame of the simulation at the corresponding pH values. The helper lipid is shown in orange, cholesterol in green, the protonated aminolipid (net charge +1) in blue, the deprotonated aminolipid (net charge 0) in yellow, and the aminolipids without a specific state ($0.2 \leq \lambda \leq 0.8$) in grey.

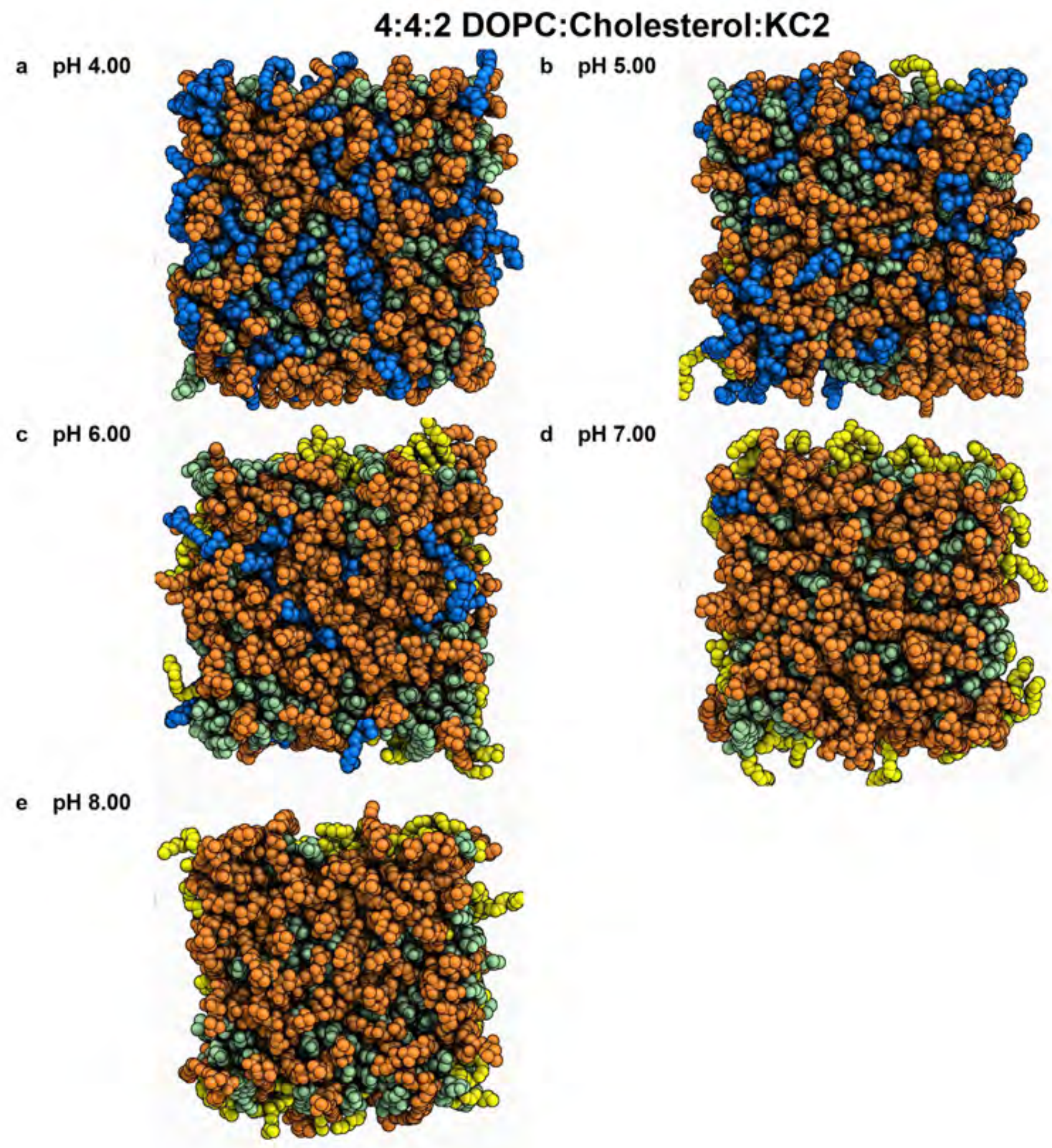

Figure S29: Top-view snapshots of the membrane in the final frame of the simulation at the corresponding pH values. The helper lipid is shown in orange, cholesterol in green, the protonated aminolipid (net charge +1) in blue, the deprotonated aminolipid (net charge 0) in yellow, and the aminolipids without a specific state ($0.2 \leq \lambda \leq 0.8$) in grey.

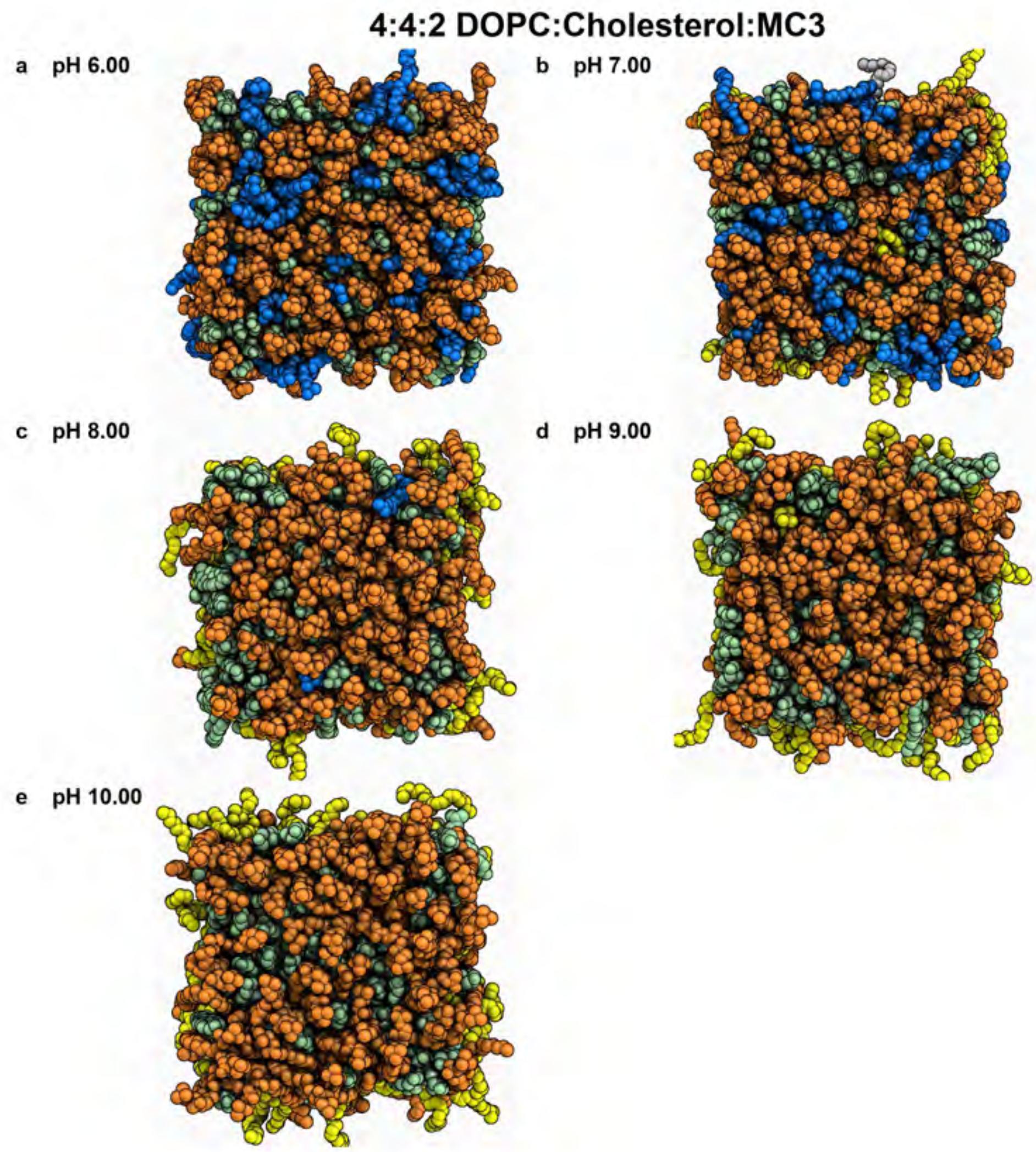

Figure S30: Top-view snapshots of the membrane in the final frame of the simulation at the corresponding pH values. The helper lipid is shown in orange, cholesterol in green, the protonated aminolipid (net charge +1) in blue, the deprotonated aminolipid (net charge 0) in yellow, and the aminolipids without a specific state ($0.2 \leq \lambda \leq 0.8$) in grey.

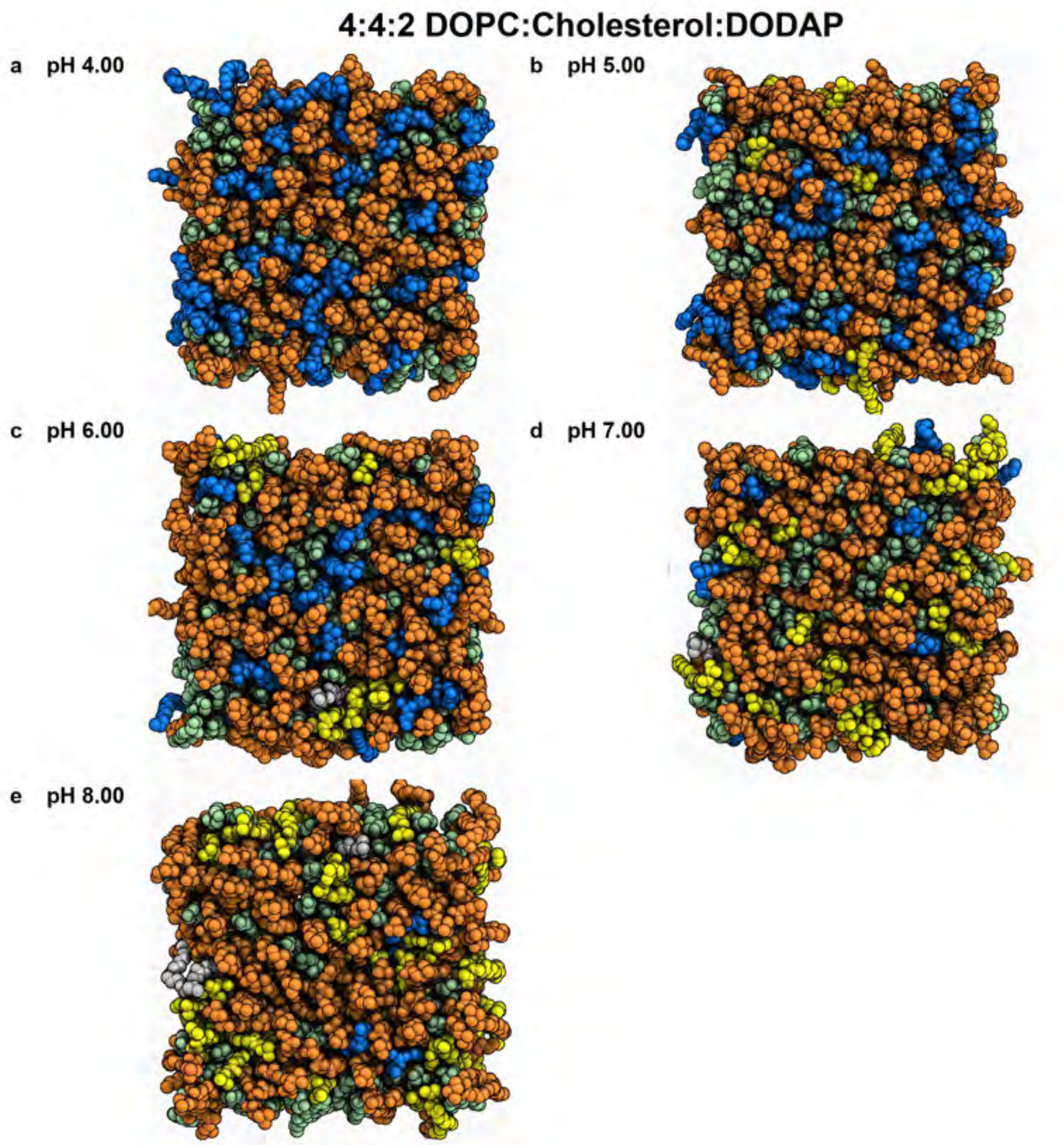

Figure S31: Top-view snapshots of the membrane in the final frame of the simulation at the corresponding pH values. The helper lipid is shown in orange, cholesterol in green, the protonated aminolipid (net charge +1) in blue, the deprotonated aminolipid (net charge 0) in yellow, and the aminolipids without a specific state ($0.2 \leq \lambda \leq 0.8$) in grey.

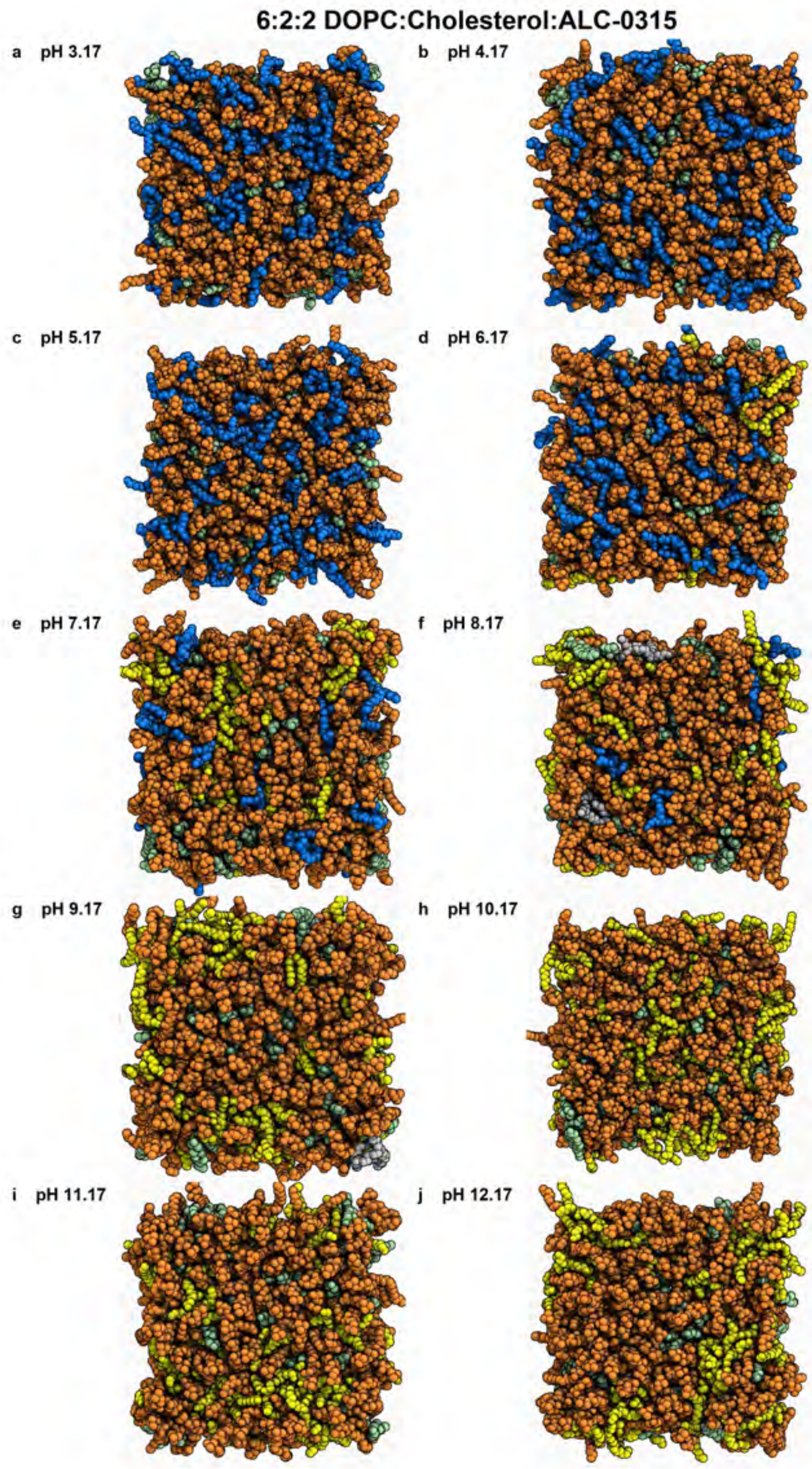

Figure S32: Top-view snapshots of the membrane in the final frame of the simulation at the corresponding pH values. The helper lipid is shown in orange, cholesterol in green, the protonated aminolipid (net charge +1) in blue, the deprotonated aminolipid (net charge 0) in yellow, and the aminolipids without a specific state ($0.2 \leq \lambda \leq 0.8$) in grey.

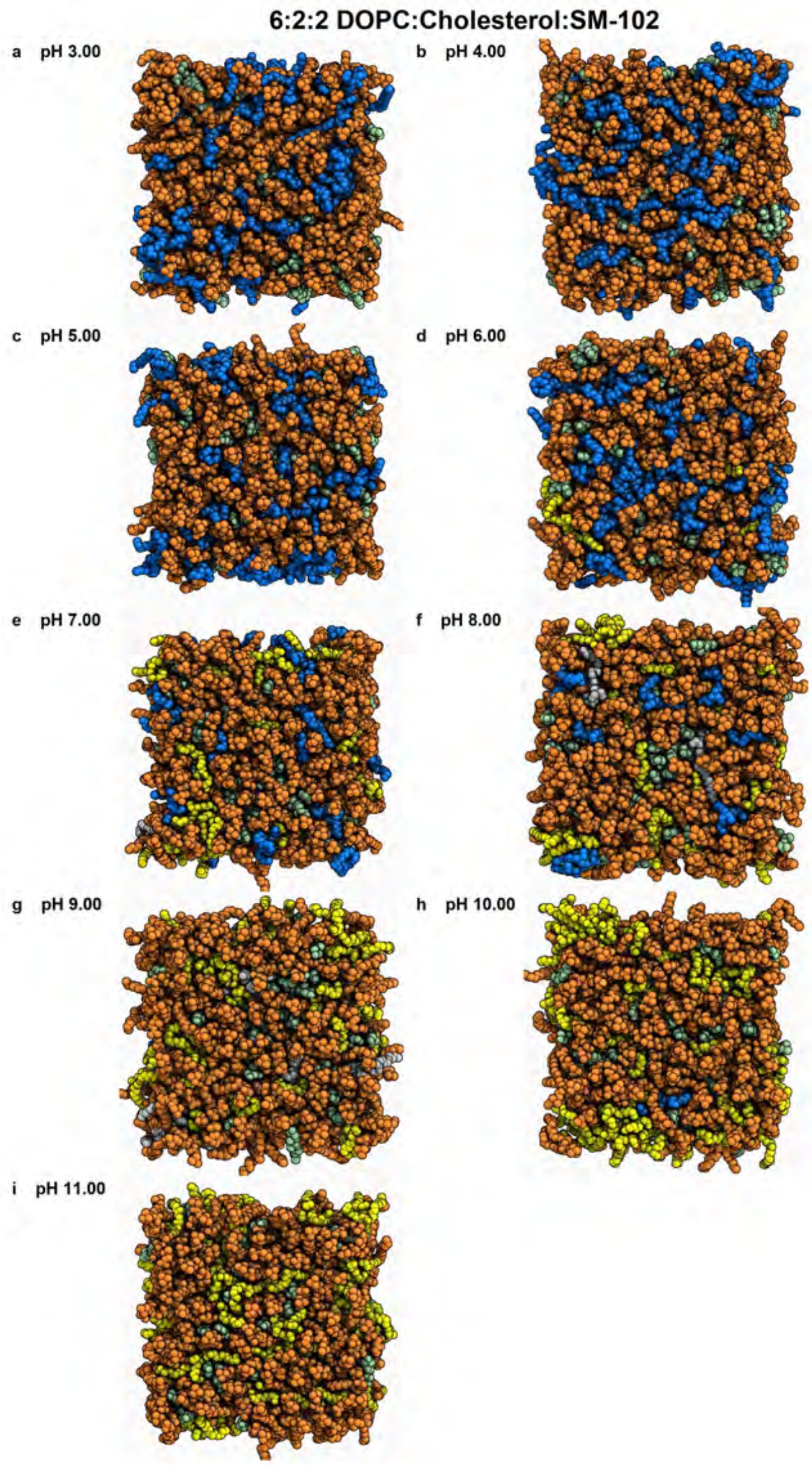

Figure S33: Top-view snapshots of the membrane in the final frame of the simulation at the corresponding pH values. The helper lipid is shown in orange, cholesterol in green, the protonated aminolipid (net charge +1) in blue, the deprotonated aminolipid (net charge 0) in yellow, and the aminolipids without a specific state ($0.2 \leq \lambda \leq 0.8$) in grey.

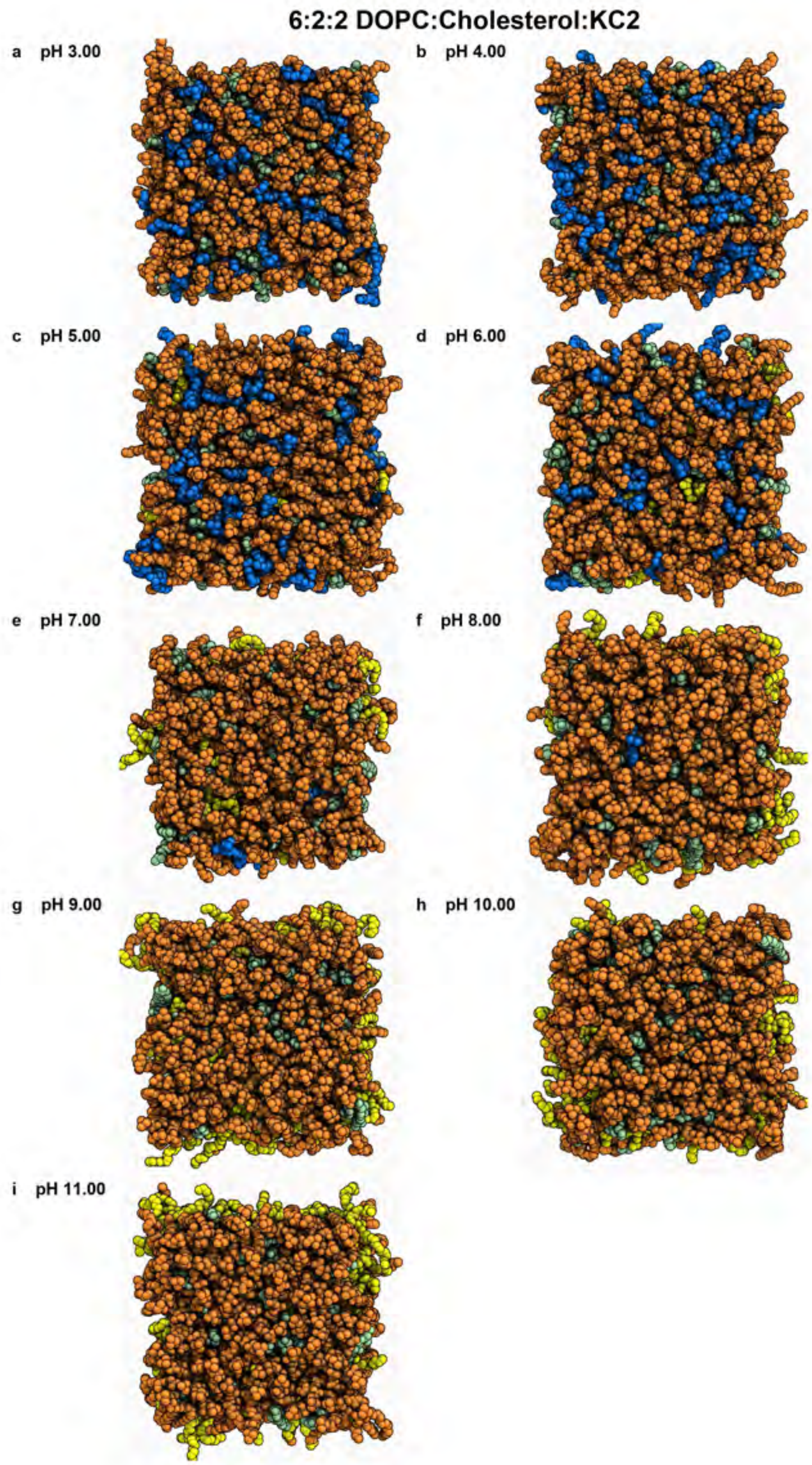

Figure S34: Top-view snapshots of the membrane in the final frame of the simulation at the corresponding pH values. The helper lipid is shown in orange, cholesterol in green, the protonated aminolipid (net charge +1) in blue, the deprotonated aminolipid (net charge 0) in yellow, and the aminolipids without a specific state ($0.2 \leq \lambda \leq 0.8$) in grey.

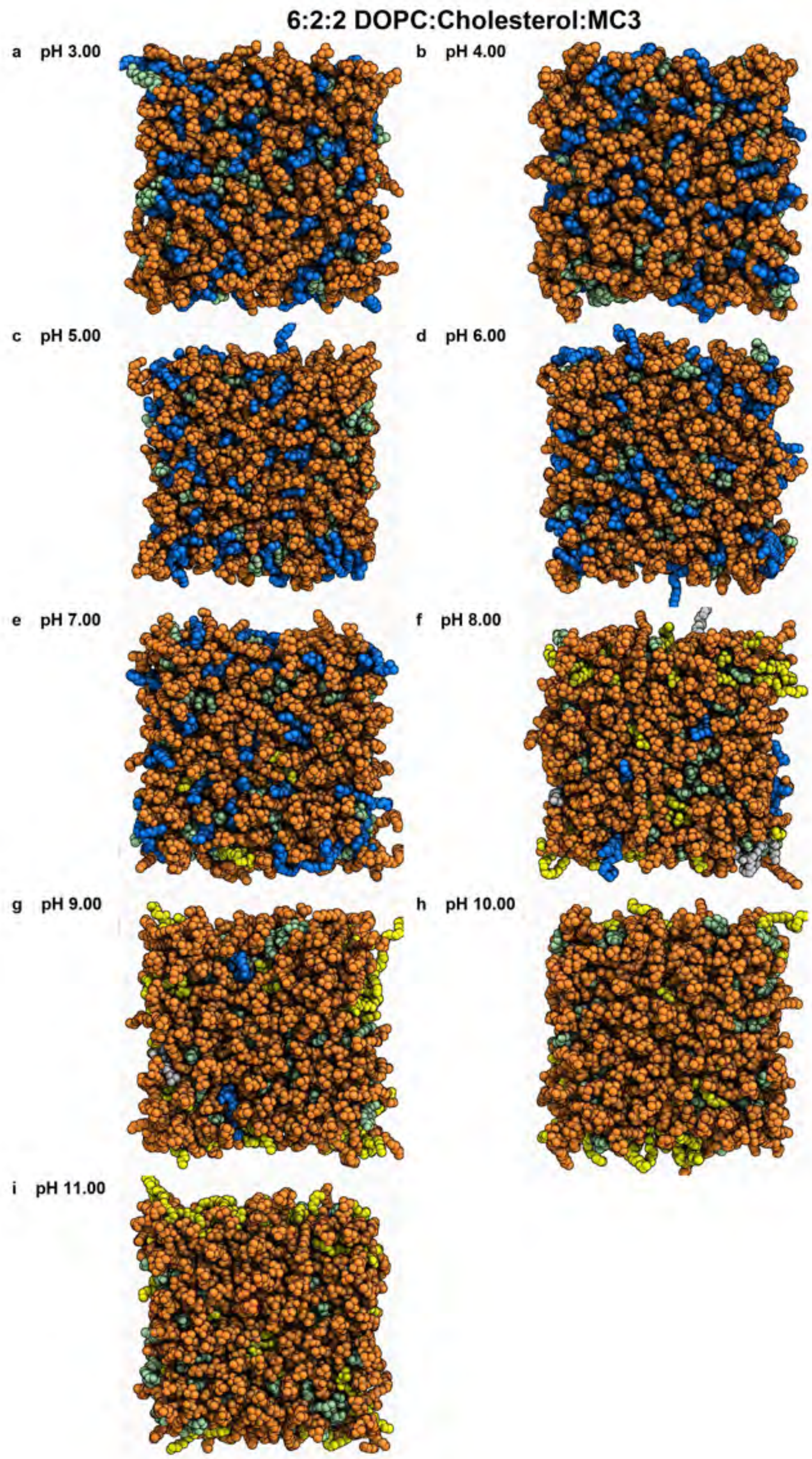

Figure S35: Top-view snapshots of the membrane in the final frame of the simulation at the corresponding pH values. The helper lipid is shown in orange, cholesterol in green, the protonated aminolipid (net charge +1) in blue, the deprotonated aminolipid (net charge 0) in yellow, and the aminolipids without a specific state ($0.2 \leq \lambda \leq 0.8$) in grey.

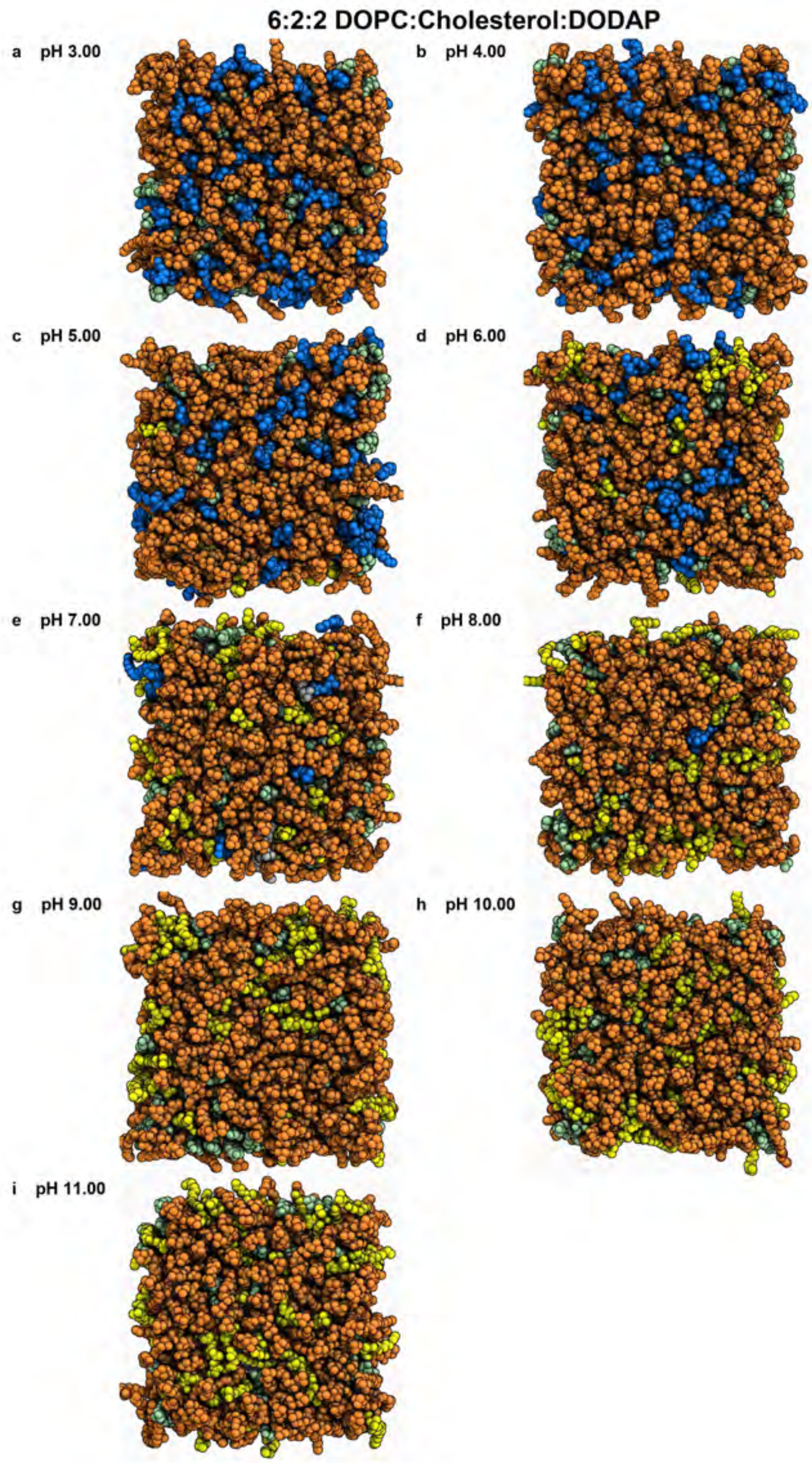

Figure S36: Top-view snapshots of the membrane in the final frame of the simulation at the corresponding pH values. The helper lipid is shown in orange, cholesterol in green, the protonated aminolipid (net charge +1) in blue, the deprotonated aminolipid (net charge 0) in yellow, and the aminolipids without a specific state ($0.2 \leq \lambda \leq 0.8$) in grey.

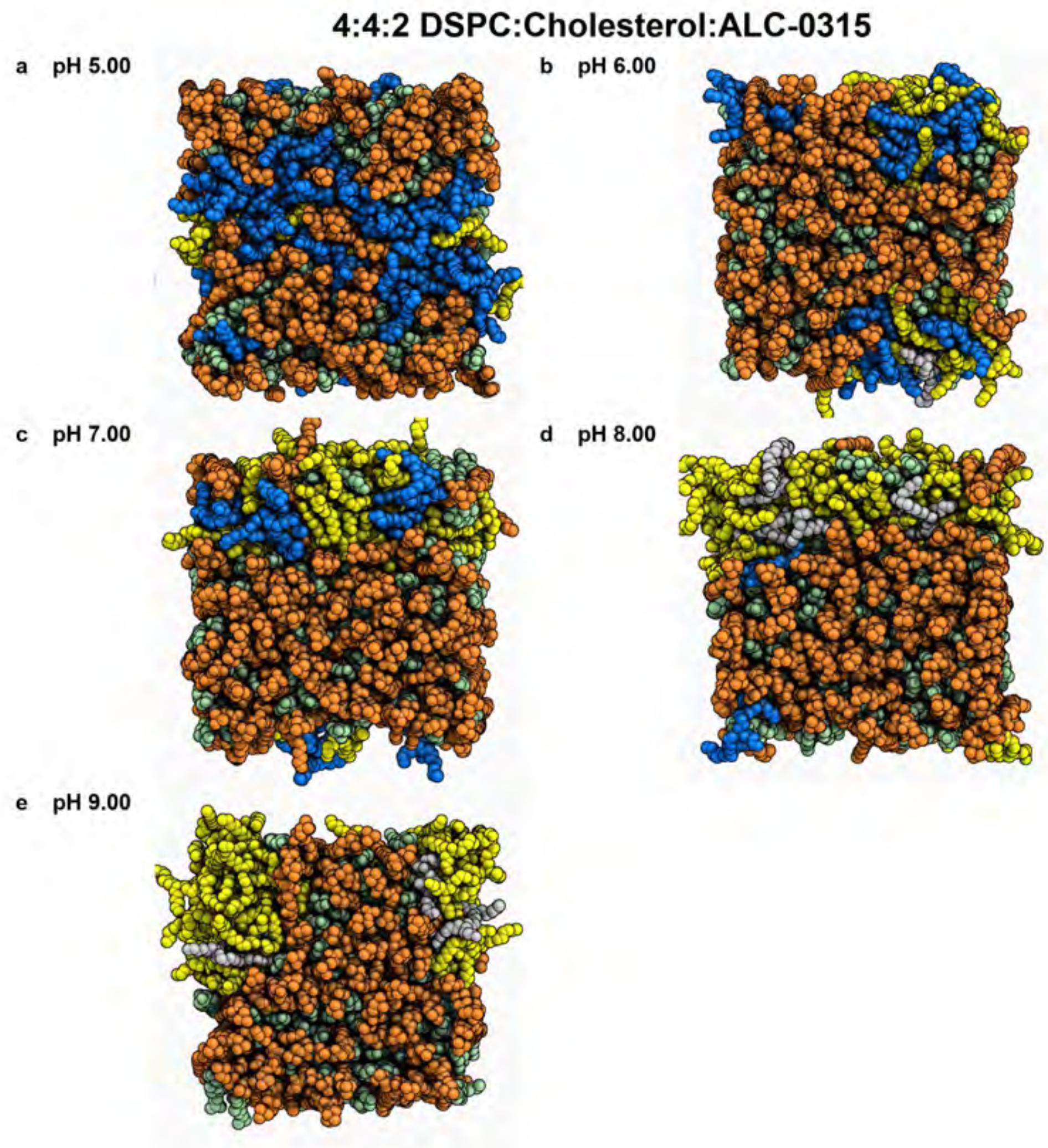

Figure S37: Top-view snapshots of the membrane in the final frame of the simulation at the corresponding pH values. The helper lipid is shown in orange, cholesterol in green, the protonated aminolipid (net charge +1) in blue, the deprotonated aminolipid (net charge 0) in yellow, and the aminolipids without a specific state ($0.2 \leq \lambda \leq 0.8$) in grey.

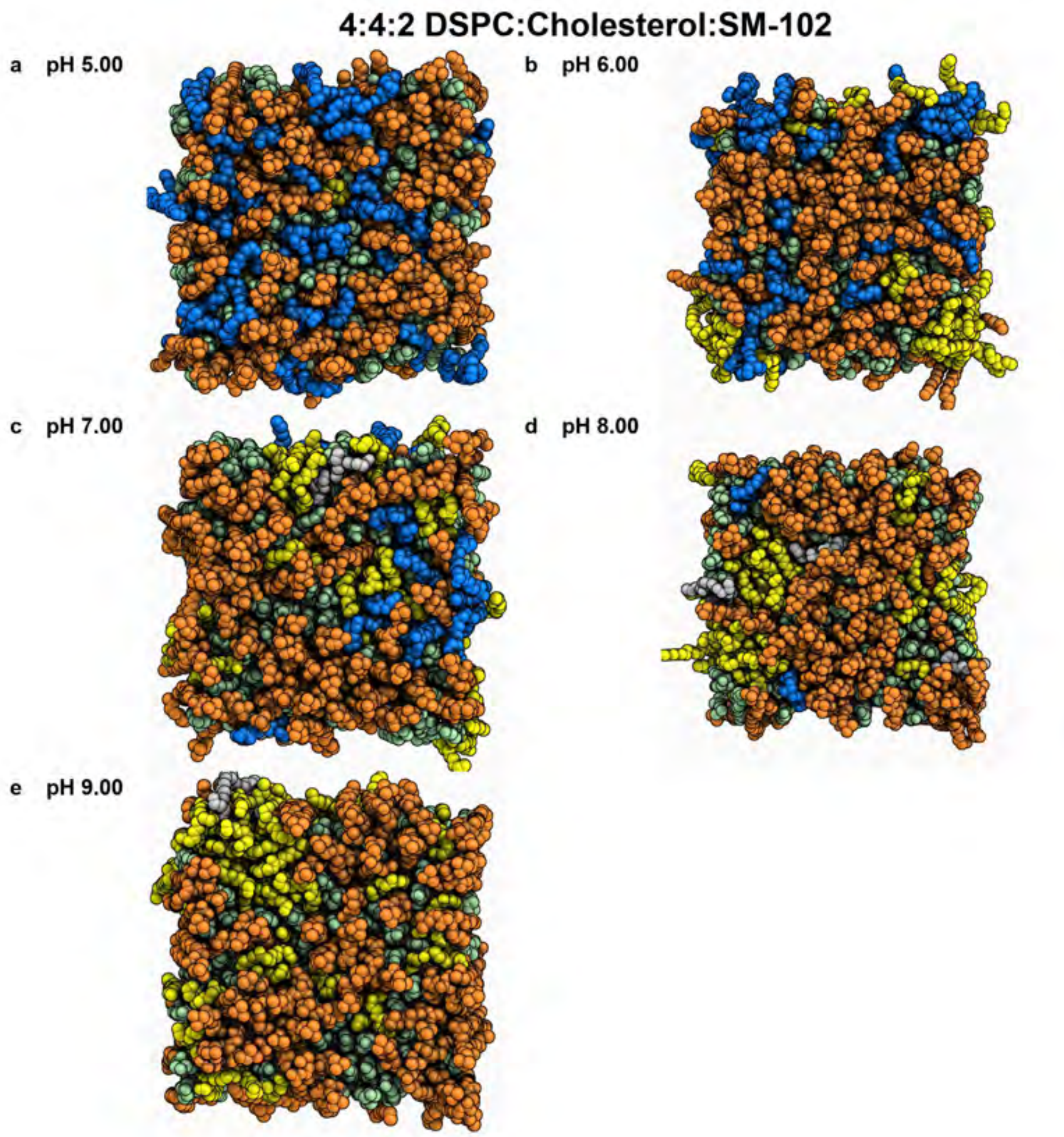

Figure S38: Top-view snapshots of the membrane in the final frame of the simulation at the corresponding pH values. The helper lipid is shown in orange, cholesterol in green, the protonated aminolipid (net charge +1) in blue, the deprotonated aminolipid (net charge 0) in yellow, and the aminolipids without a specific state ($0.2 \leq \lambda \leq 0.8$) in grey.

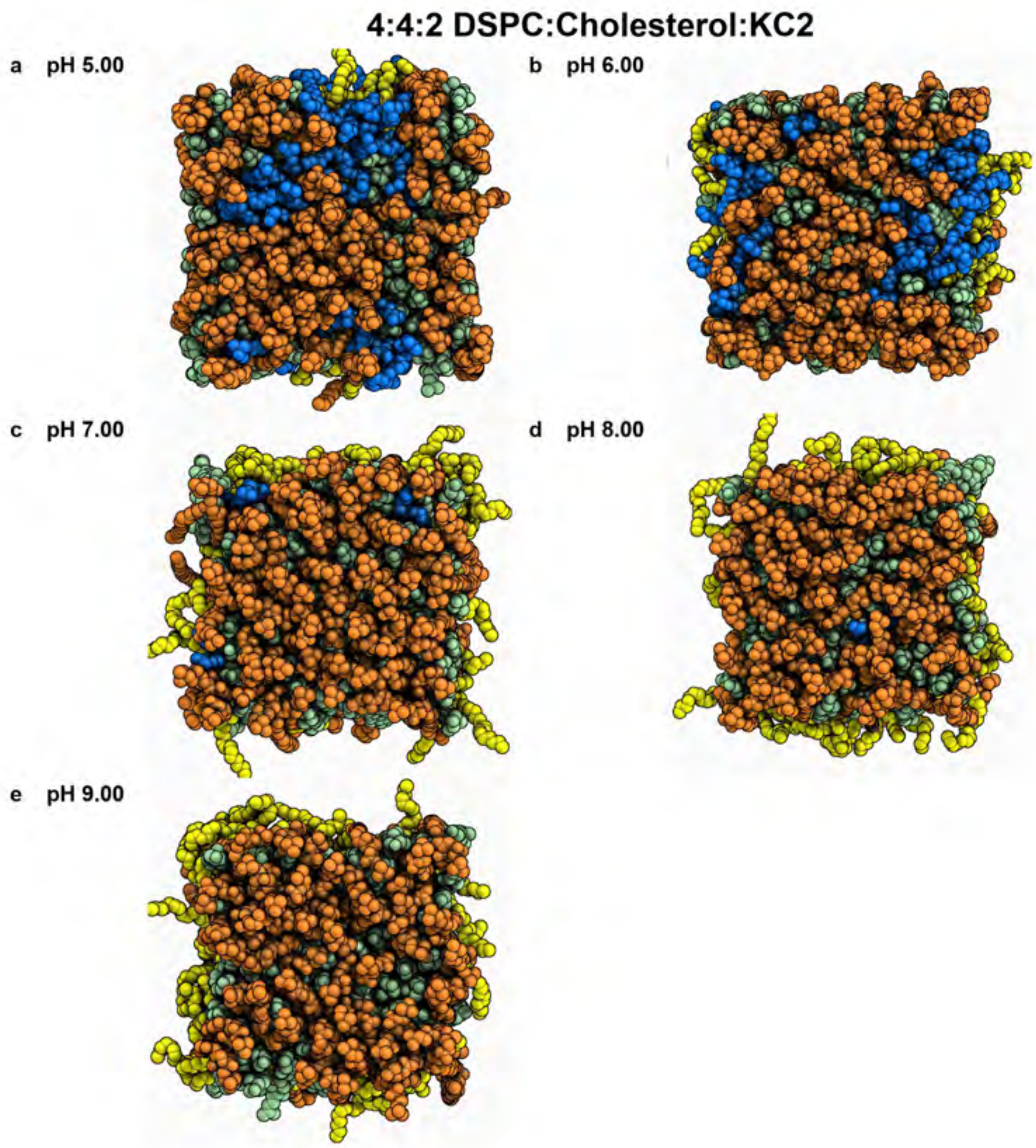

Figure S39: Top-view snapshots of the membrane in the final frame of the simulation at the corresponding pH values. The helper lipid is shown in orange, cholesterol in green, the protonated aminolipid (net charge +1) in blue, the deprotonated aminolipid (net charge 0) in yellow, and the aminolipids without a specific state ($0.2 \leq \lambda \leq 0.8$) in grey.

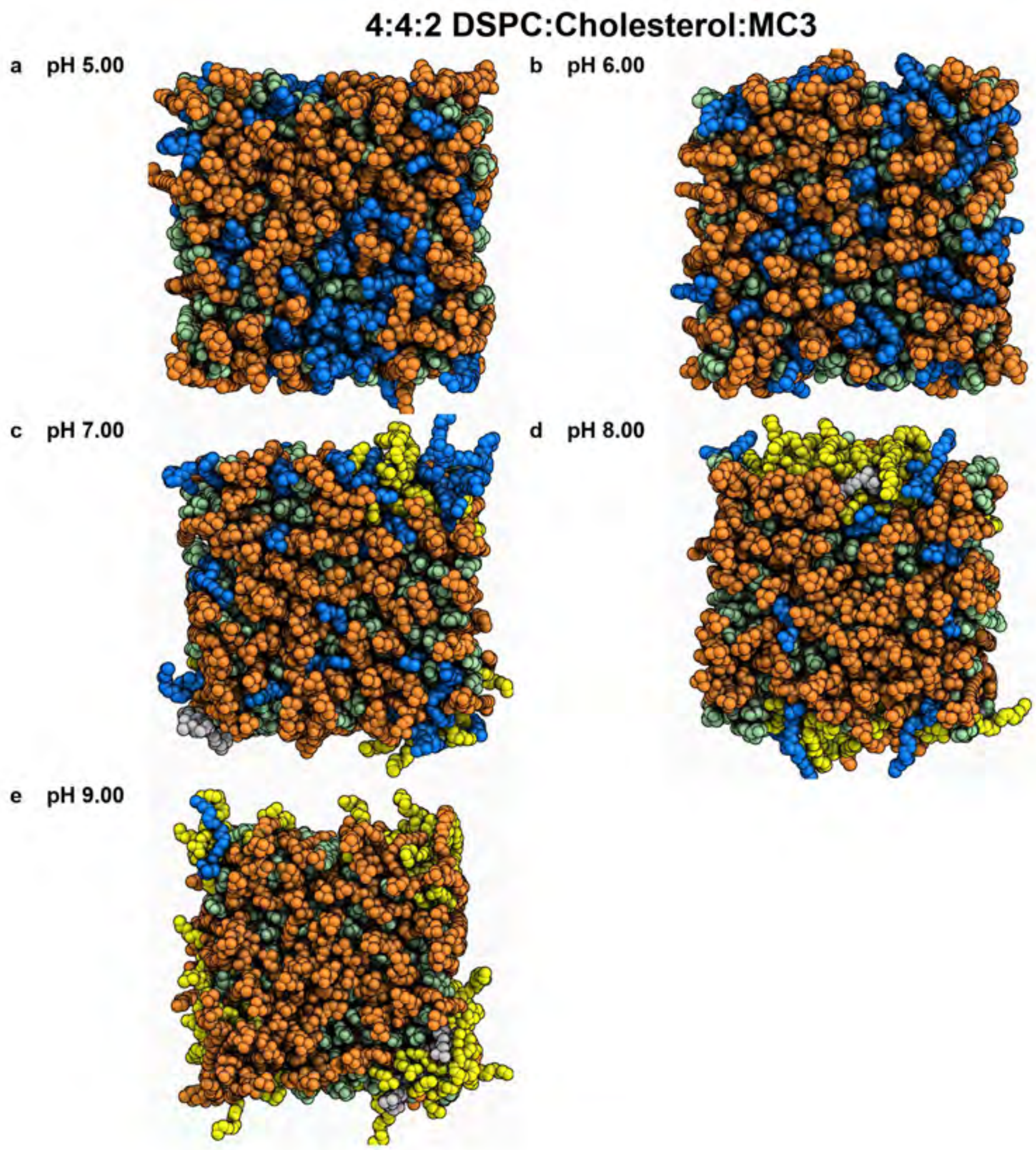

Figure S40: Top-view snapshots of the membrane in the final frame of the simulation at the corresponding pH values. The helper lipid is shown in orange, cholesterol in green, the protonated aminolipid (net charge +1) in blue, the deprotonated aminolipid (net charge 0) in yellow, and the aminolipids without a specific state ($0.2 \leq \lambda \leq 0.8$) in grey.

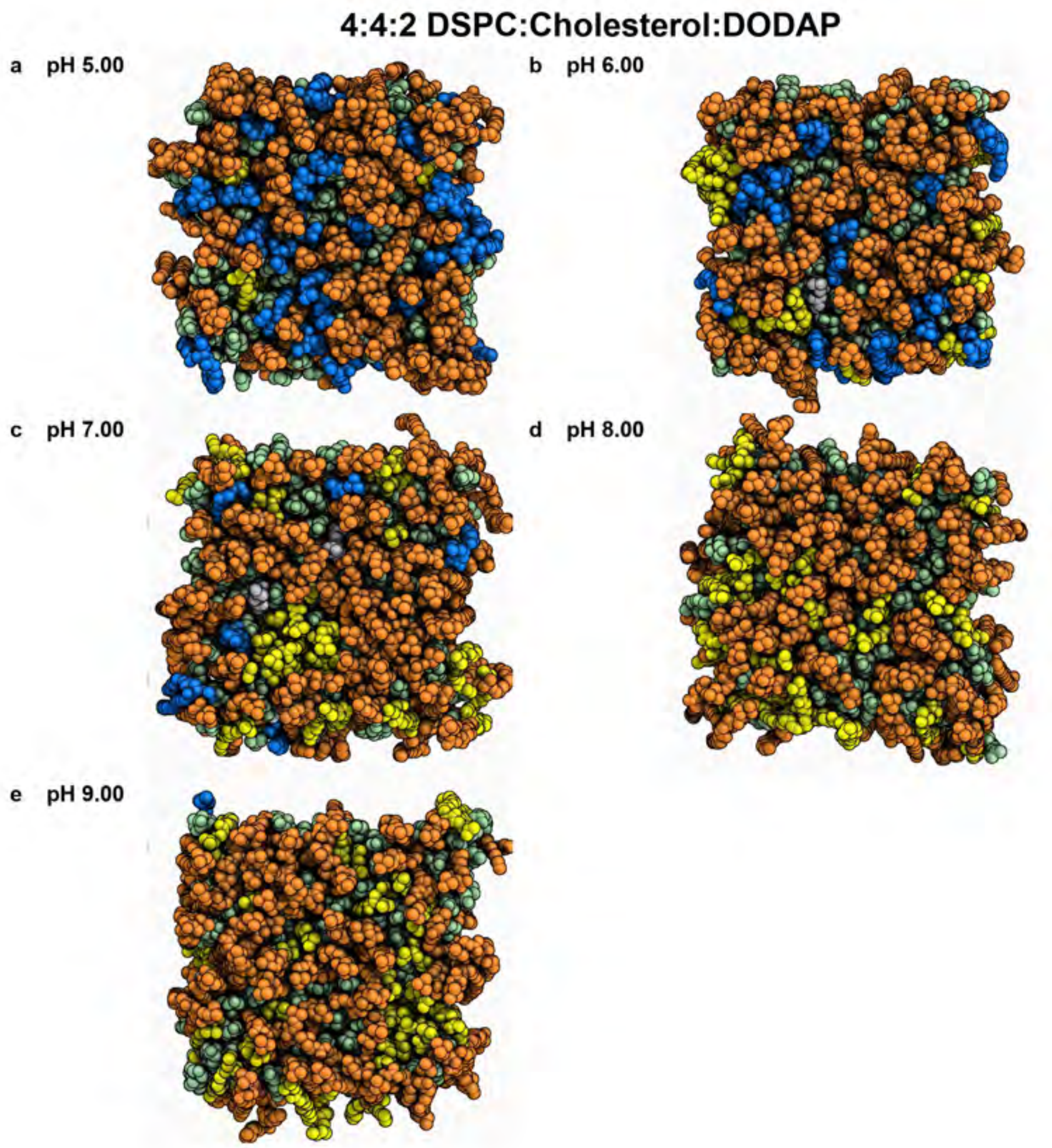

Figure S41: Top-view snapshots of the membrane in the final frame of the simulation at the corresponding pH values. The helper lipid is shown in orange, cholesterol in green, the protonated aminolipid (net charge +1) in blue, the deprotonated aminolipid (net charge 0) in yellow, and the aminolipids without a specific state ($0.2 \leq \lambda \leq 0.8$) in grey.

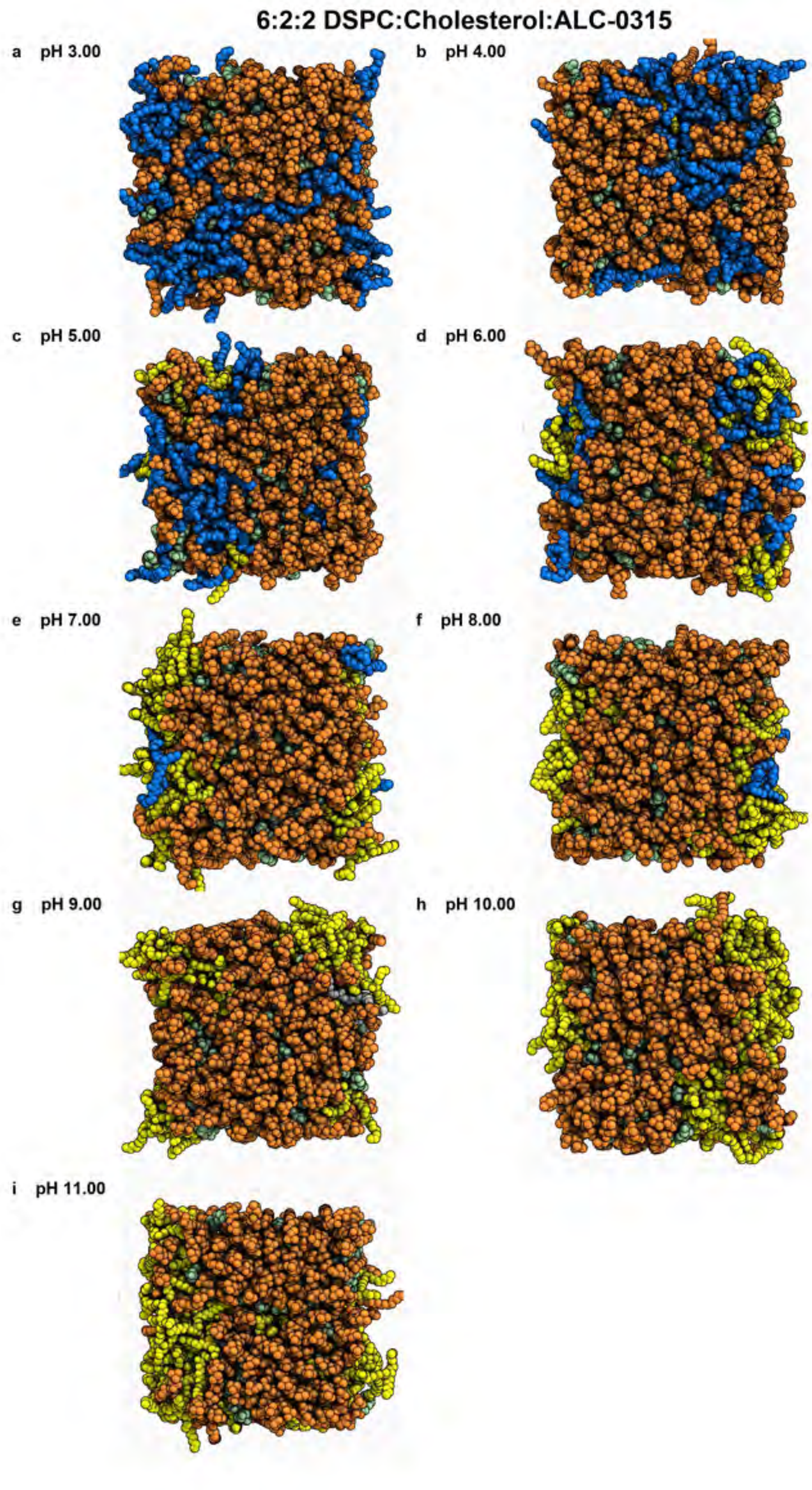

Figure S42: Top-view snapshots of the membrane in the final frame of the simulation at the corresponding pH values. The helper lipid is shown in orange, cholesterol in green, the protonated aminolipid (net charge +1) in blue, the deprotonated aminolipid (net charge 0) in yellow, and the aminolipids without a specific state ($0.2 \leq \lambda \leq 0.8$) in grey.

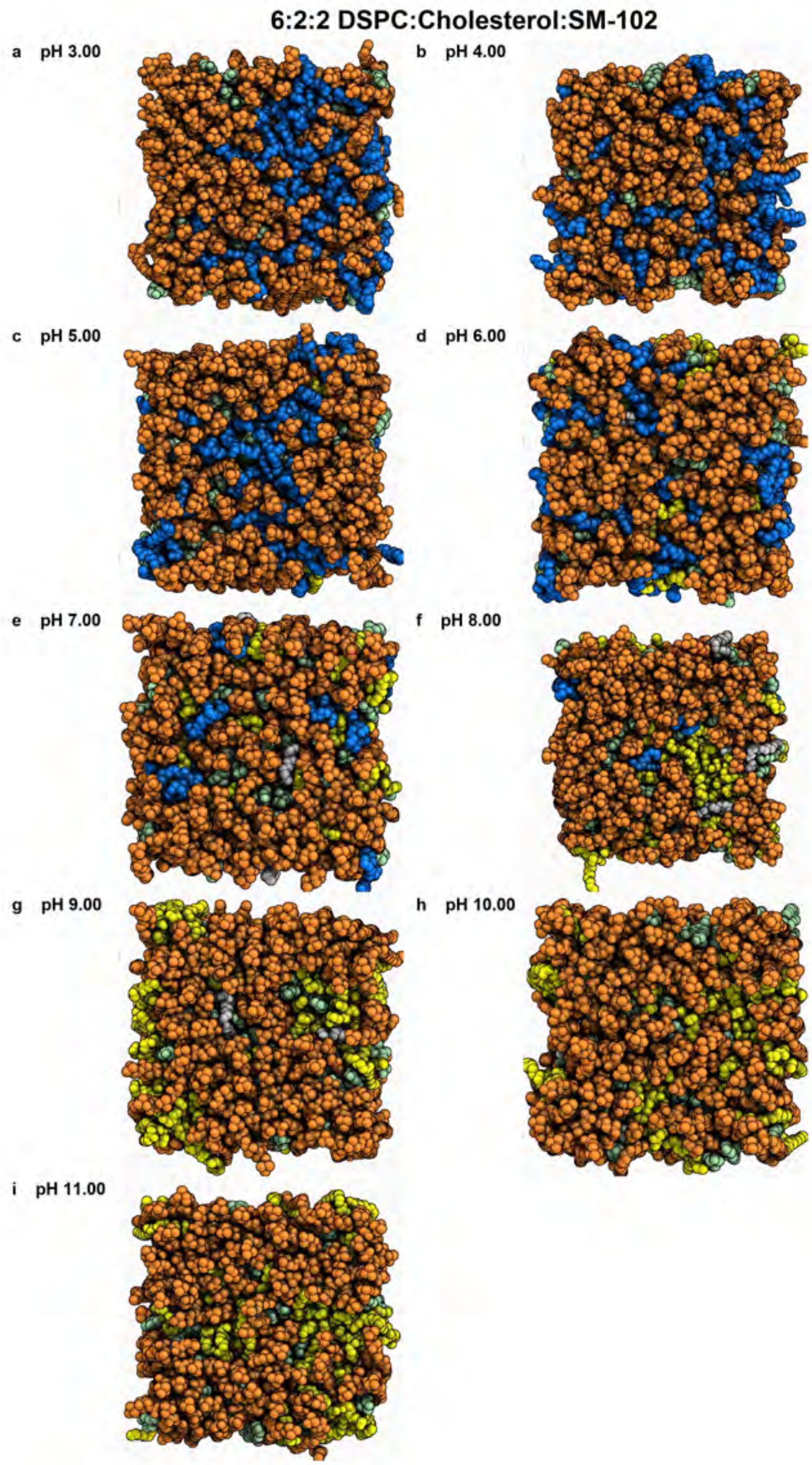

Figure S43: Top-view snapshots of the membrane in the final frame of the simulation at the corresponding pH values. The helper lipid is shown in orange, cholesterol in green, the protonated aminolipid (net charge +1) in blue, the deprotonated aminolipid (net charge 0) in yellow, and the aminolipids without a specific state ($0.2 \leq \lambda \leq 0.8$) in grey.

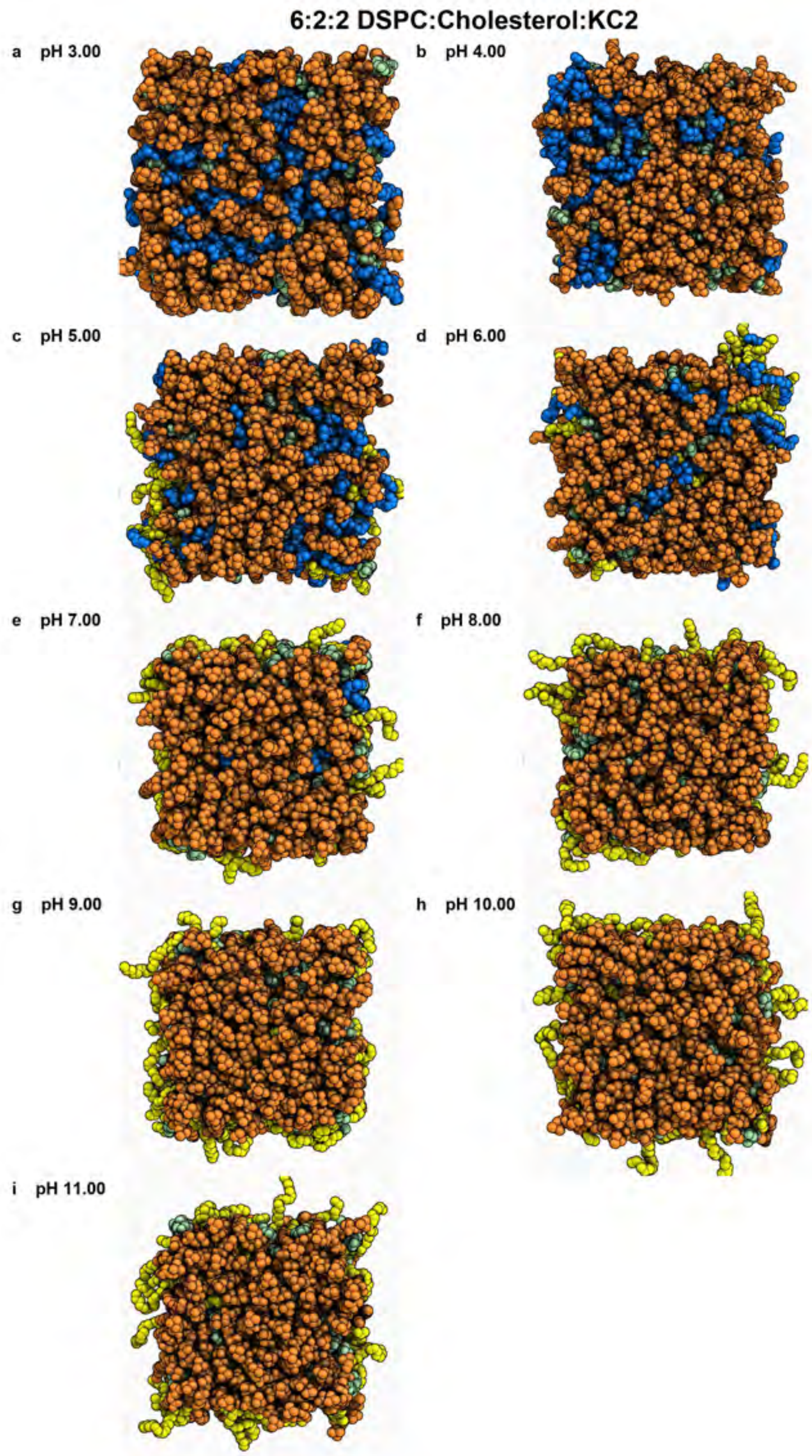

Figure S44: Top-view snapshots of the membrane in the final frame of the simulation at the corresponding pH values. The helper lipid is shown in orange, cholesterol in green, the protonated aminolipid (net charge +1) in blue, the deprotonated aminolipid (net charge 0) in yellow, and the aminolipids without a specific state ($0.2 \leq \lambda \leq 0.8$) in grey.

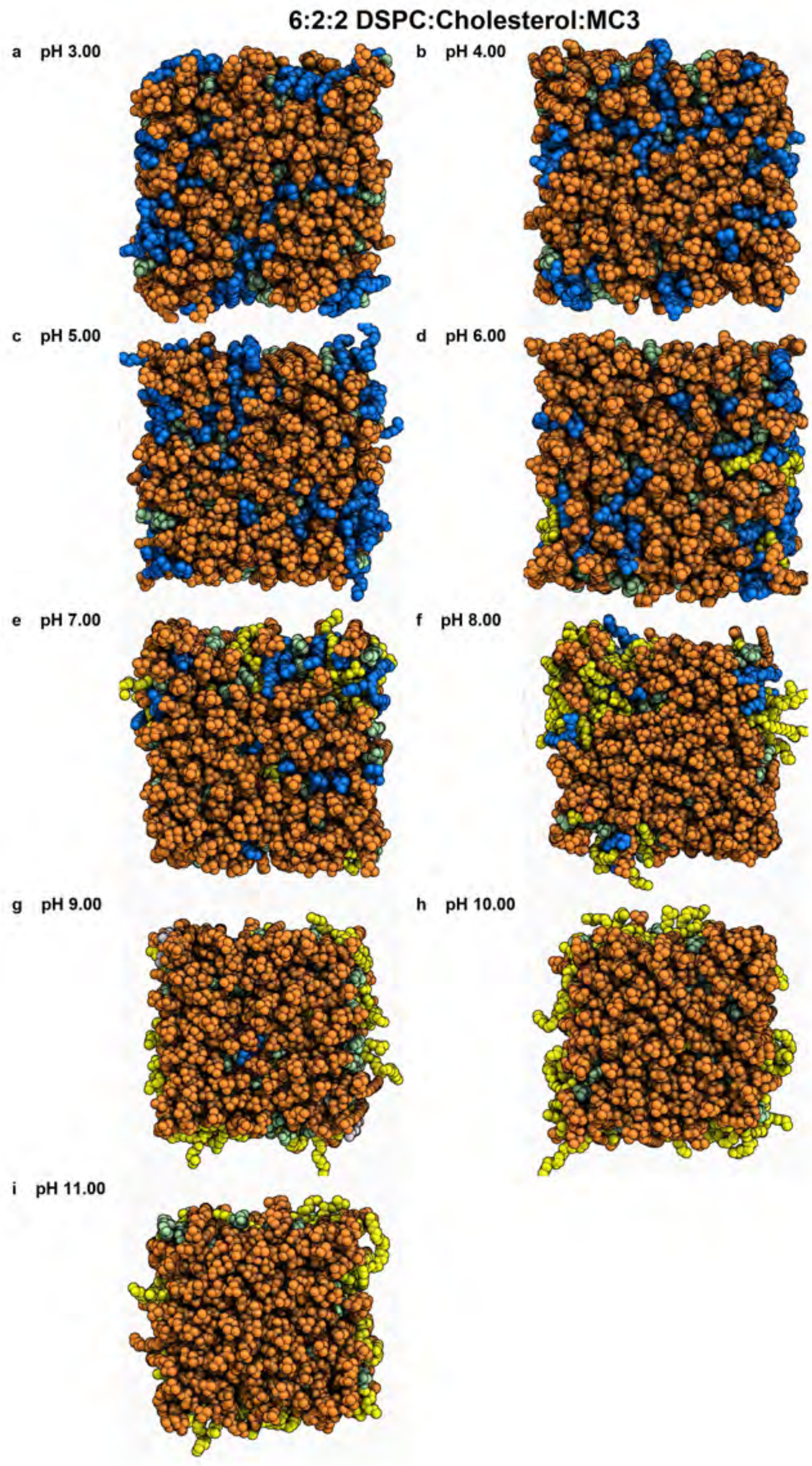

Figure S45: Top-view snapshots of the membrane in the final frame of the simulation at the corresponding pH values. The helper lipid is shown in orange, cholesterol in green, the protonated aminolipid (net charge +1) in blue, the deprotonated aminolipid (net charge 0) in yellow, and the aminolipids without a specific state ($0.2 \leq \lambda \leq 0.8$) in grey.

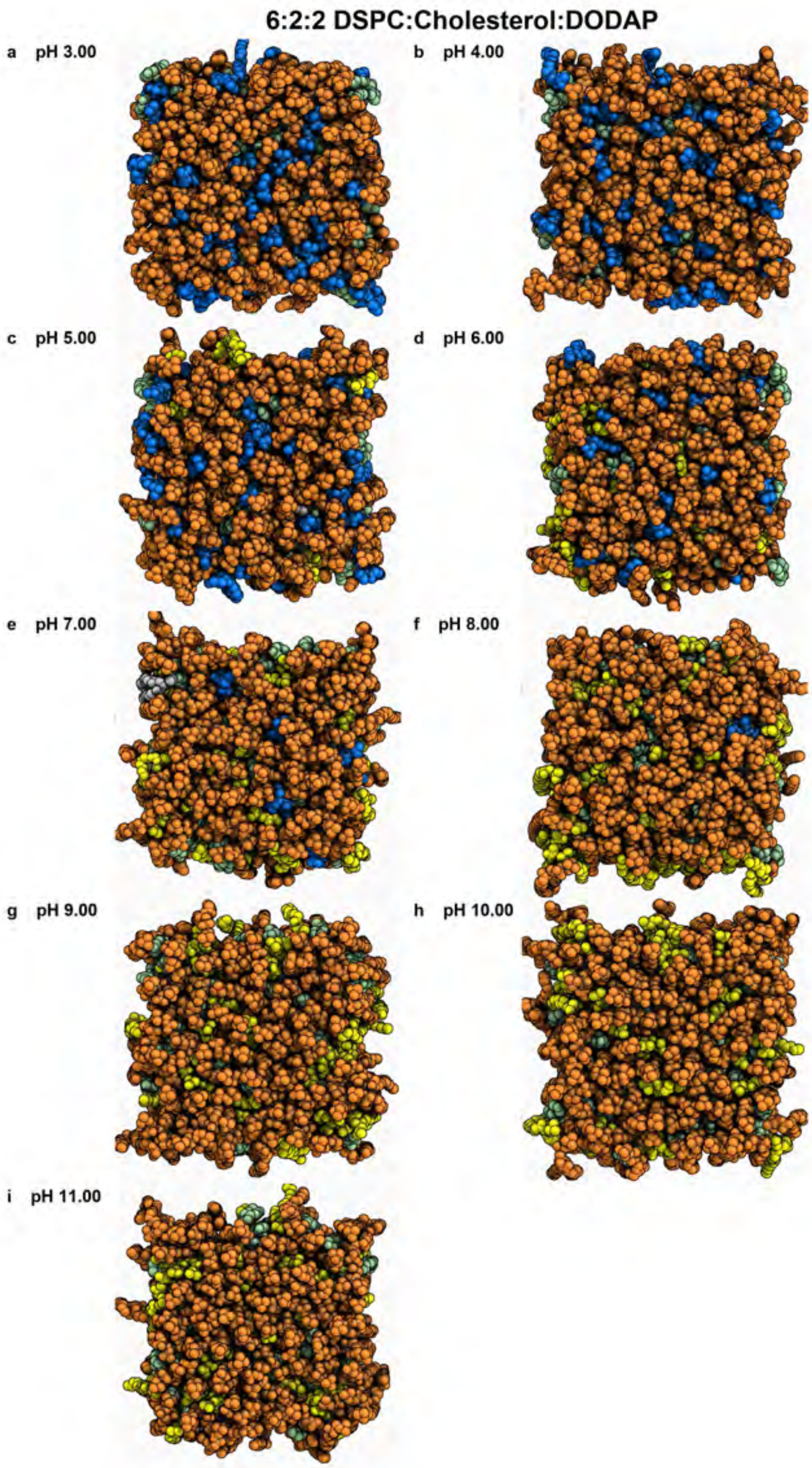

Figure S46: Top-view snapshots of the membrane in the final frame of the simulation at the corresponding pH values. The helper lipid is shown in orange, cholesterol in green, the protonated aminolipid (net charge +1) in blue, the deprotonated aminolipid (net charge 0) in yellow, and the aminolipids without a specific state ($0.2 \leq \lambda \leq 0.8$) in grey.

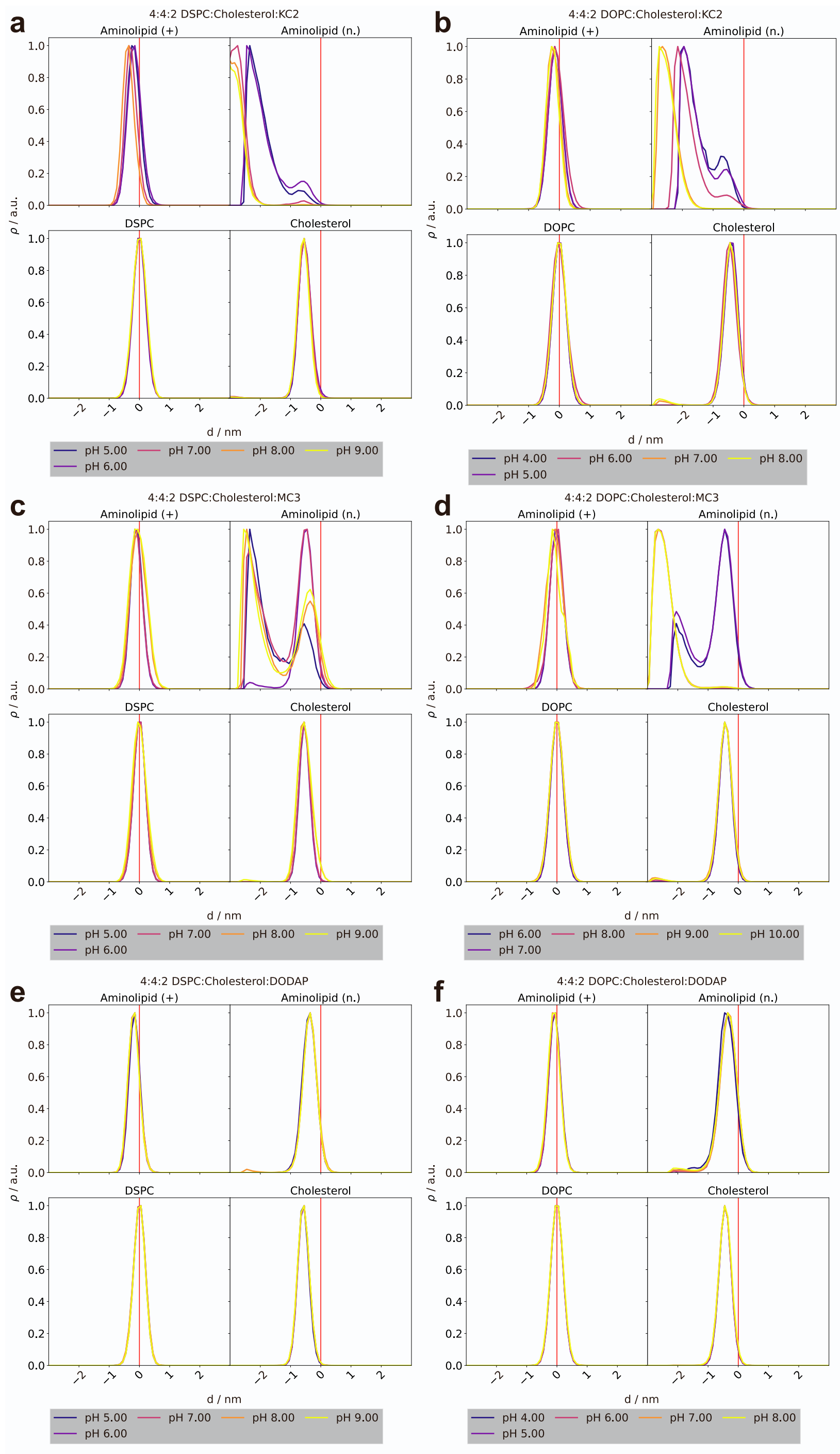

Figure S47: Lipid density profiles along the z-axis for membranes with a 4:4:2 helper lipid:cholesterol:aminolipid composition containing either **a**,**c**,**e** DSPC or **b**,**d**,**f** DOPC as the helper lipid, and **a**,**b** DLin-KC2-DMA, **c**,**d** DLin-MC3-DMA, or **e**,**f** DODAP as the aminolipid. Densities were calculated using one representative atom per lipid (oxygen for cholesterol, nitrogen for the aminolipid, and phosphorus for the helper lipid). Positions are reported relative to the membrane surface, defined as the average z-position of the phosphorus atoms in the helper lipid headgroups of each leaflet. Negative distances (d) correspond to the membrane core, whereas positive distances correspond to the solvent phase. Results were averaged over both leaflets.

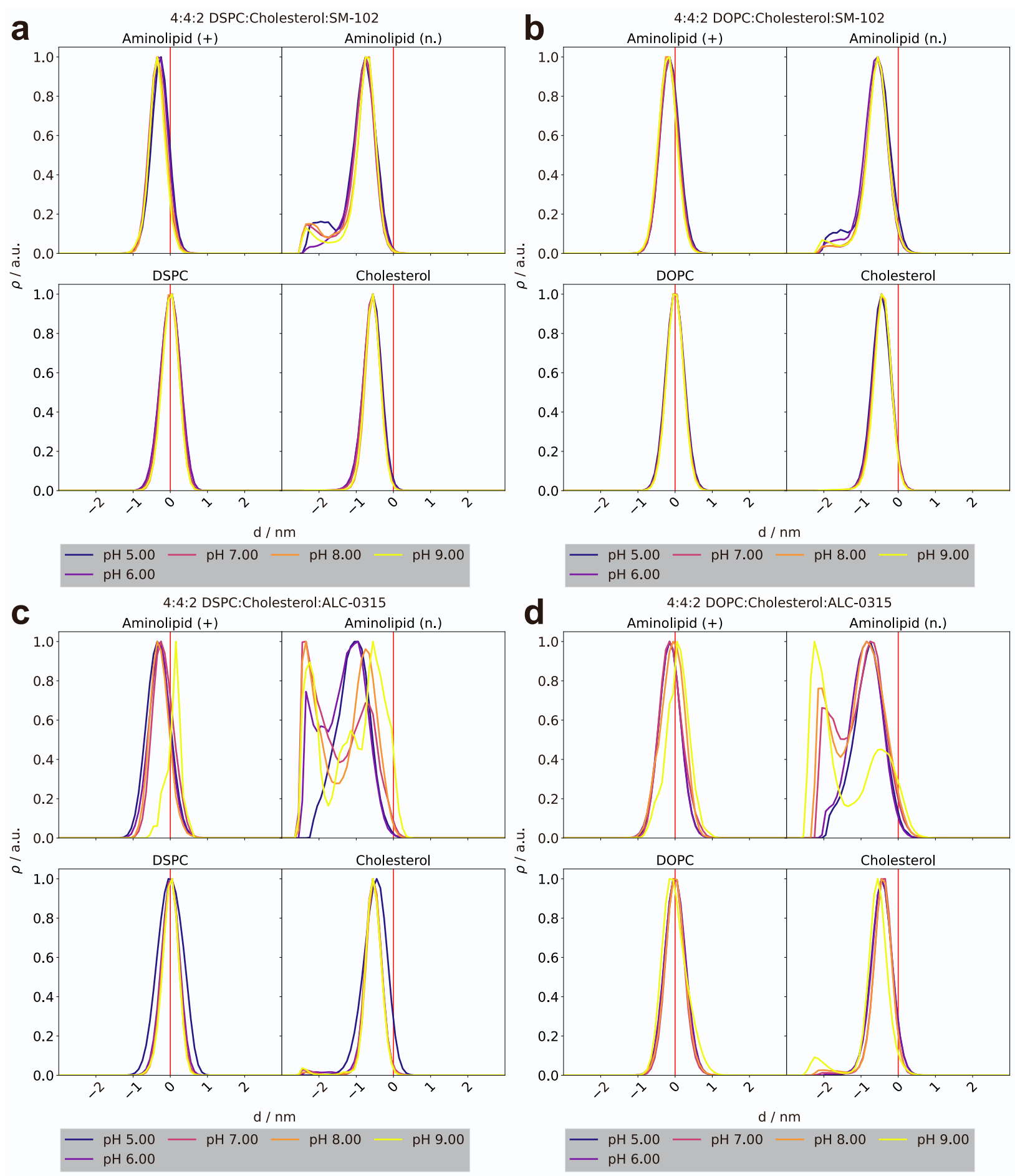

Figure S48: Lipid density profiles along the z-axis for membranes with a 4:4:2 helper lipid:cholesterol:aminolipid composition containing either **a**,**c** DSPC or **b**,**d** DOPC as the helper lipid, and **a**,**b** SM-102, or **c**,**d** ALC-0315 as the aminolipid. Densities were calculated using one representative atom per lipid (oxygen for cholesterol, nitrogen for the aminolipid, and phosphorus for the helper lipid). Positions are reported relative to the membrane surface, defined as the average z-position of the phosphorus atoms in the helper lipid headgroups of each leaflet. Negative distances (d) correspond to the membrane core, whereas positive distances correspond to the solvent phase. Results were averaged over both leaflets.

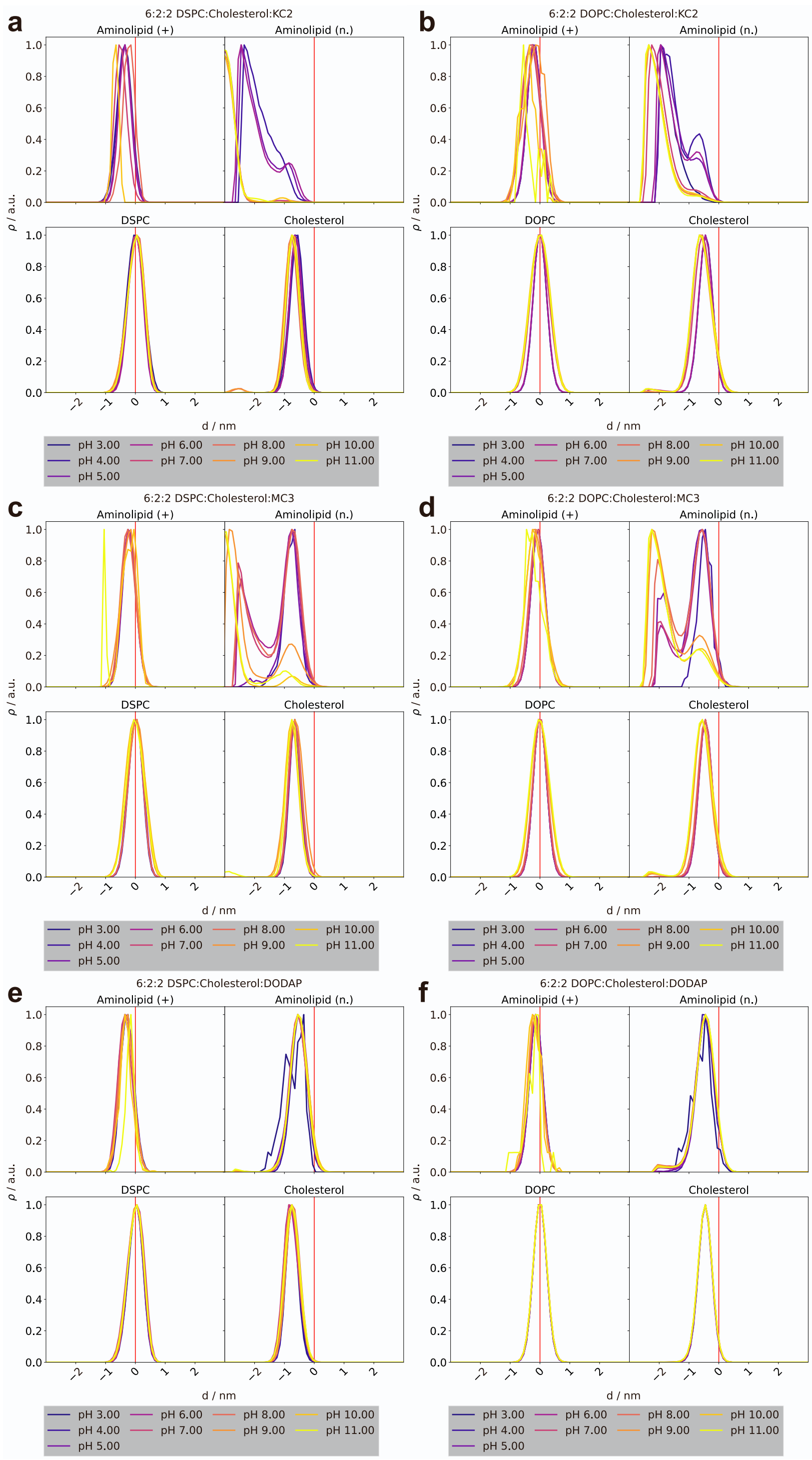

Figure S49: Lipid density profiles along the z-axis for membranes with a 6:2:2 helper lipid:cholesterol:aminolipid composition containing either **a**,**c**,**e** DSPC or **b**,**d**,**f** DOPC as the helper lipid, and **a**,**b** DLin-KC2-DMA, **c**,**d** DLin-MC3-DMA, or **e**,**f** DODAP as the aminolipid. Densities were calculated using one representative atom per lipid (oxygen for cholesterol, nitrogen for the aminolipid, and phosphorus for the helper lipid). Positions are reported relative to the membrane surface, defined as the average z-position of the phosphorus atoms in the helper lipid headgroups of each leaflet. Negative distances (d) correspond to the membrane core, whereas positive distances correspond to the solvent phase. Results were averaged over both leaflets.

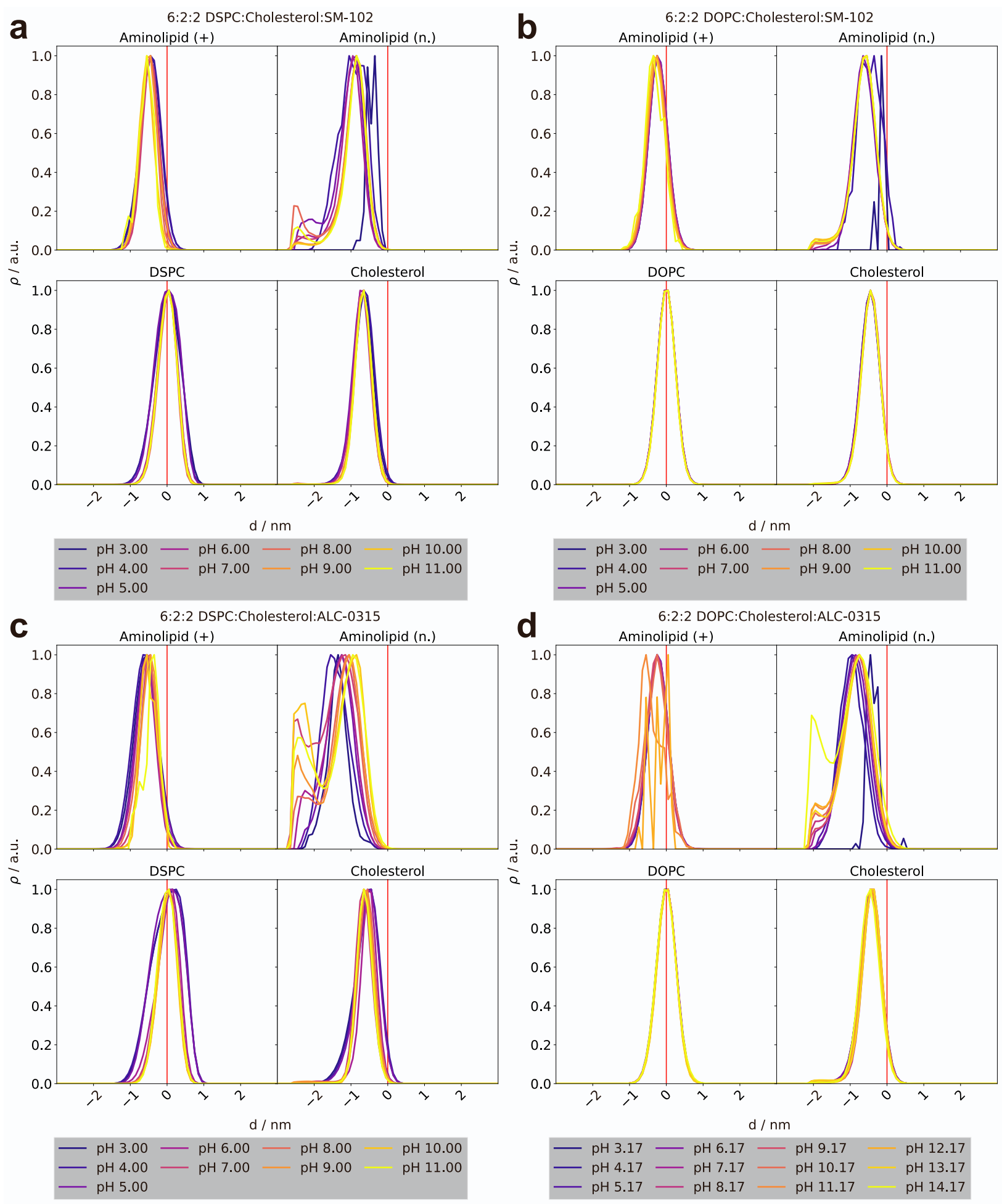

Figure S50: Lipid density profiles along the z-axis for membranes with a 6:2:2 helper lipid:cholesterol:aminolipid composition containing either **a**,**c** DSPC or **b**,**d** DOPC as the helper lipid, and **a**,**b** SM-102, or **c**,**d** ALC-0315 as the aminolipid. Densities were calculated using one representative atom per lipid (oxygen for cholesterol, nitrogen for the aminolipid, and phosphorus for the helper lipid). Positions are reported relative to the membrane surface, defined as the average z-position of the phosphorus atoms in the helper lipid headgroups of each leaflet. Negative distances (d) correspond to the membrane core, whereas positive distances correspond to the solvent phase. Results were averaged over both leaflets.

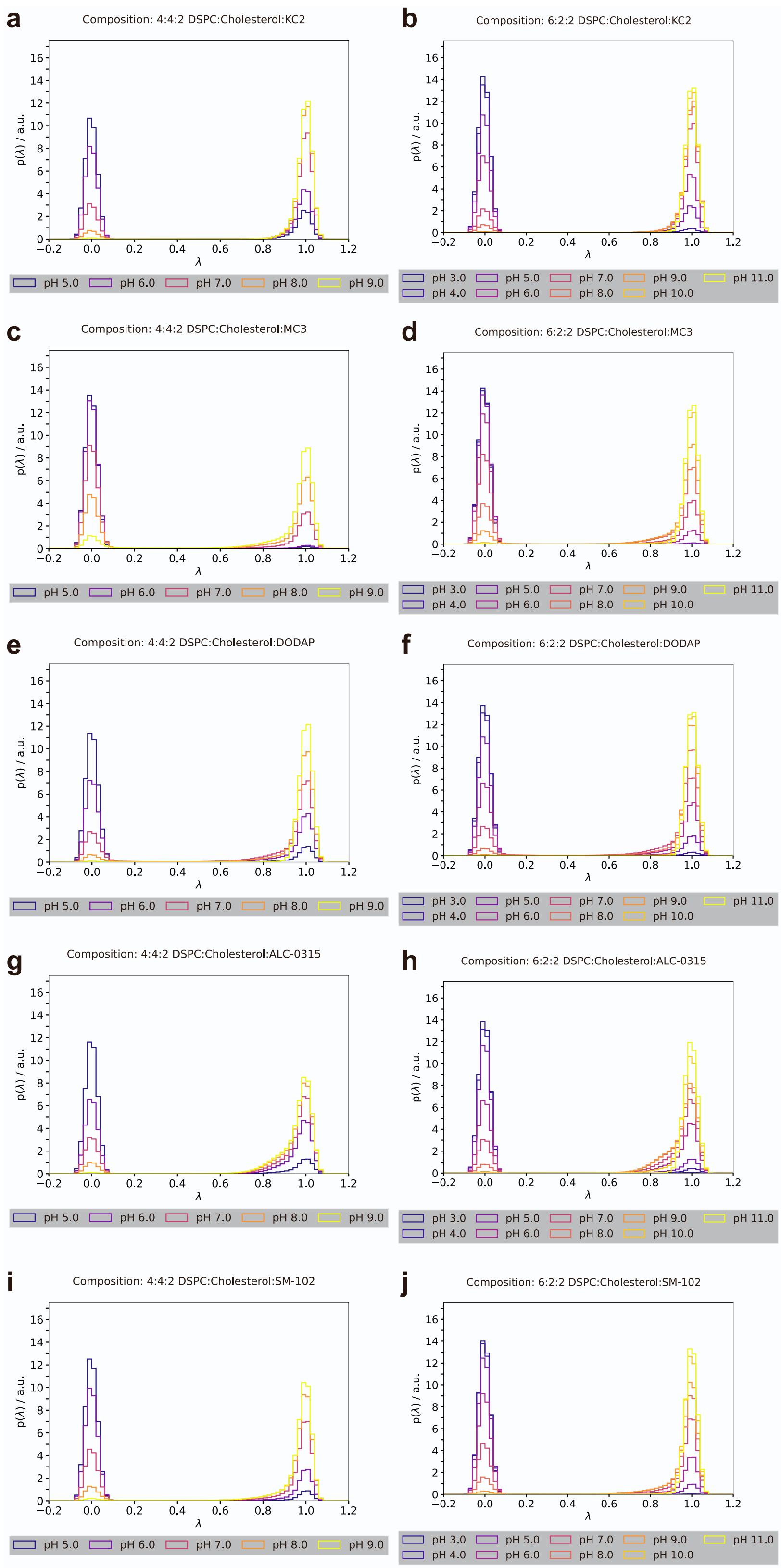

Figure S51: Distribution of the λ-coordinate at each pH, aggregated over all aminolipids in a membrane with **a**,**c**,**e**,**g**,**i** a 4:4:2 or **b**,**d**,**f**,**h**,**j** a 6:2:2 DSPC:cholesterol:aminolipid composition containing either **a**,**b** DLin-KC2-DMA, **c**,**d** DLin-MC3-DMA, **e**,**f** DODAP, **g**,**h** ALC-0315 or **i**,**j** SM-102 as the aminolipid.

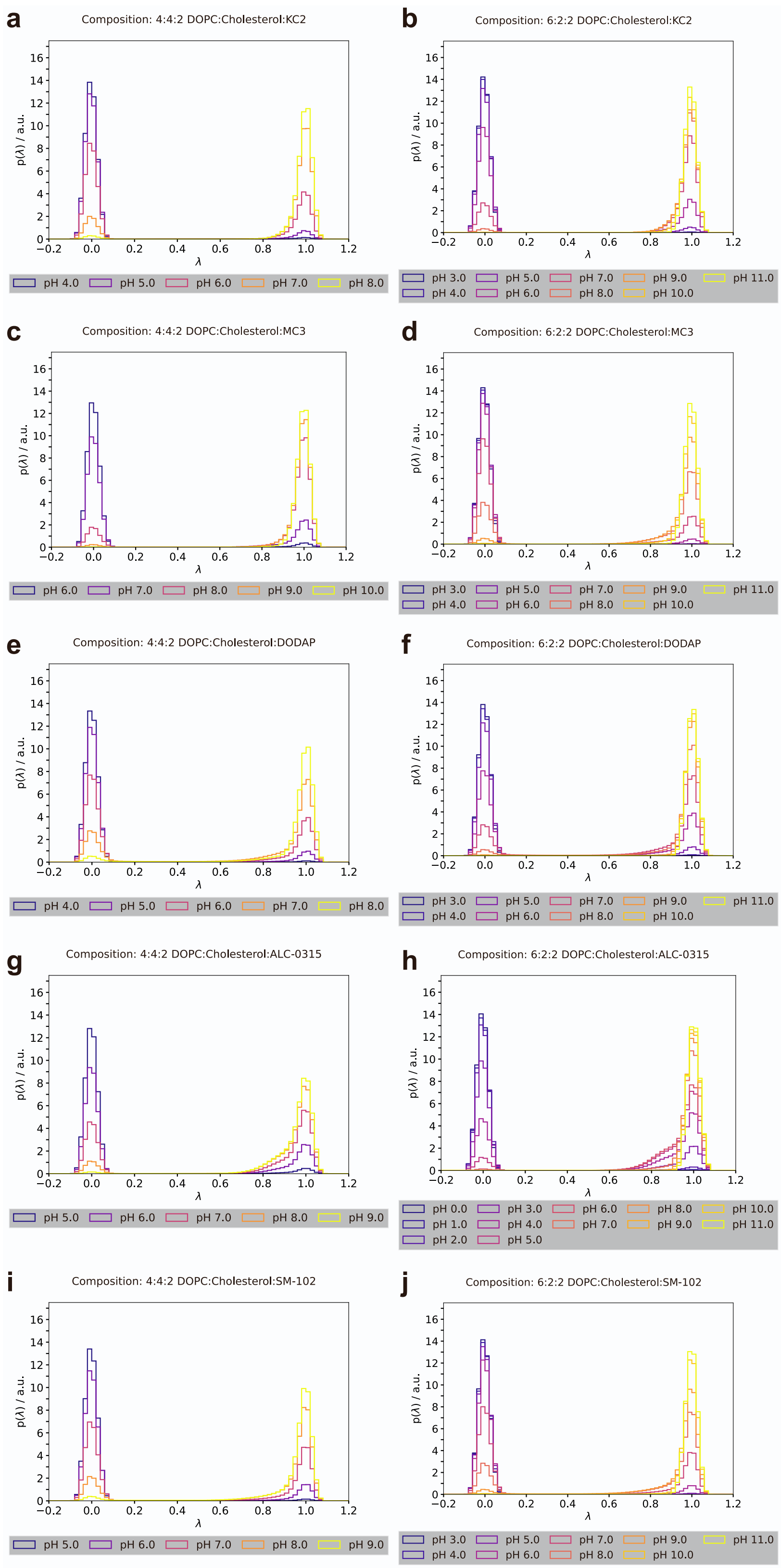

Figure S52: Distribution of the λ-coordinate at each pH, aggregated over all aminolipids in a membrane with **a**,**c**,**e**,**g**,**i** a 4:4:2 or **b**,**d**,**f**,**h**,**j** a 6:2:2 DOPC:cholesterol:aminolipid composition containing either **a**,**b** DLin-KC2-DMA, **c**,**d** DLin-MC3-DMA, **e**,**f** DODAP, **g**,**h** ALC-0315 or **i**,**j** SM-102 as the aminolipid.

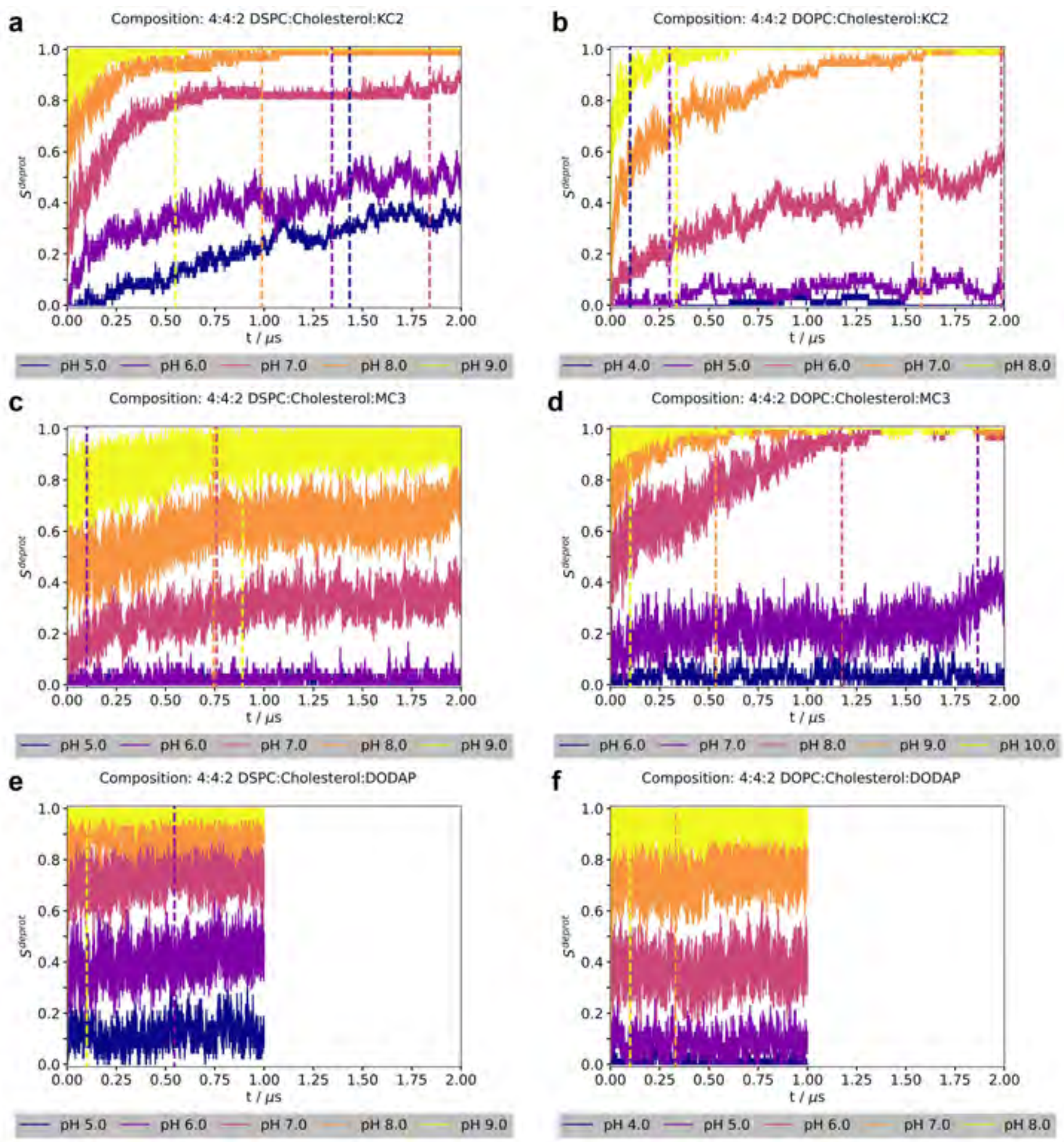

Figure S53: Fraction of deprotonated aminolipids (S^{deprot}) per frame for membranes with a 4:4:2 helper lipid:cholesterol:aminolipid composition containing either **a,c,e** DSPC or **b,d,f** DOPC as the helper lipid, and **a,b** DLin-KC2-DMA, **c,d** DLin-MC3-DMA, or **e,f** DODAP as the aminolipid. Vertical dashed lines indicate the equilibration time determined for each pH value.

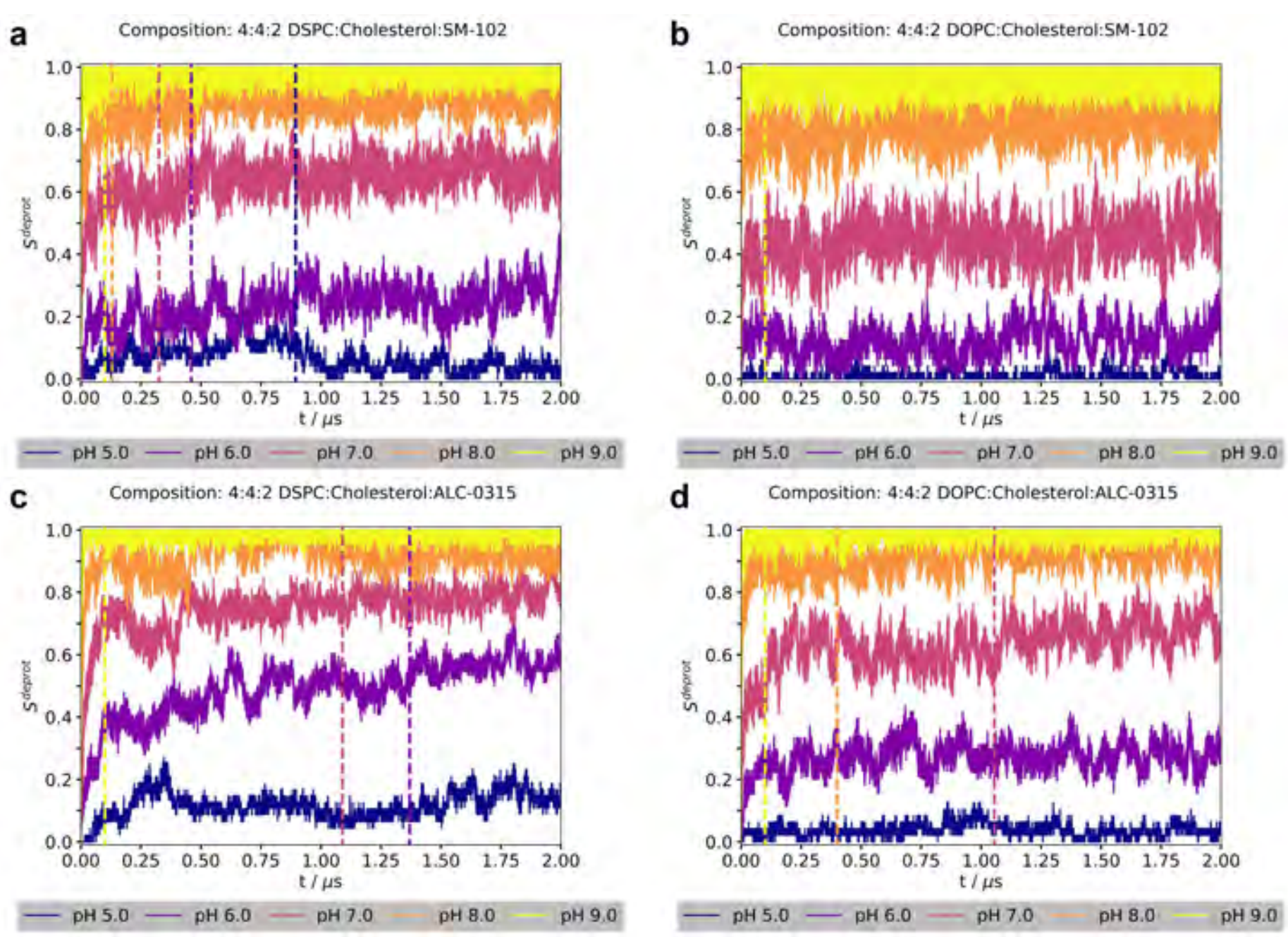

Figure S54: Fraction of deprotonated aminolipids (S^{deprot}) per frame for membranes with a 4:4:2 helper lipid:cholesterol:aminolipid composition containing either **a,c** DSPC or **b,d** DOPC as the helper lipid, and **a,b** SM-102, or **c,d** ALC-0315 as the aminolipid. Vertical dashed lines indicate the equilibration time determined for each pH value.

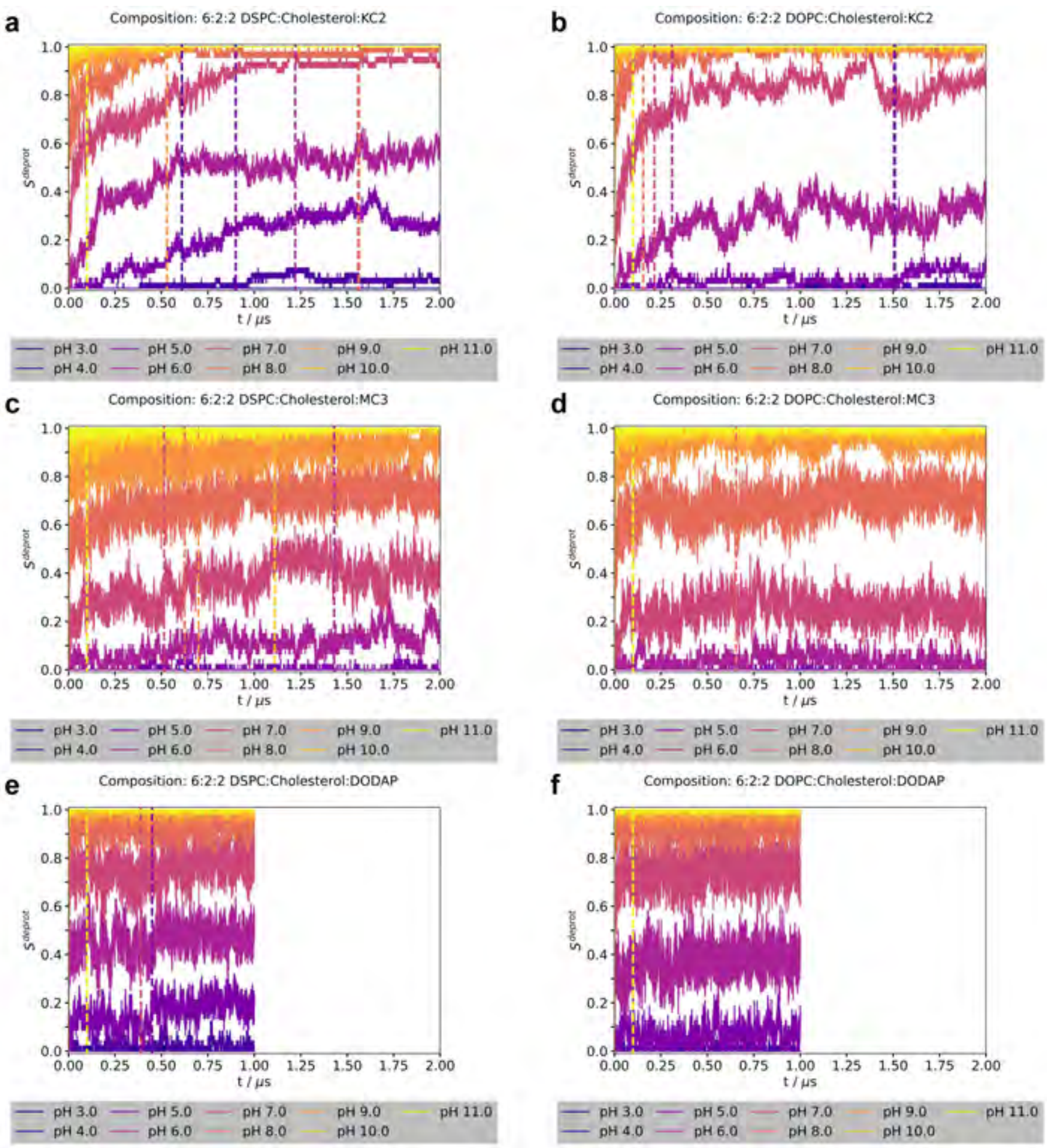

Figure S55: Fraction of deprotonated aminolipids (S^{deprot}) per frame for membranes with a 6:2:2 helper lipid:cholesterol:aminolipid composition containing either **a,c,e** DSPC or **b,d,f** DOPC as the helper lipid, and **a,b** DLin-KC2-DMA, **c,d** DLin-MC3-DMA, or **e,f** DODAP as the aminolipid. Vertical dashed lines indicate the equilibration time determined for each pH value.

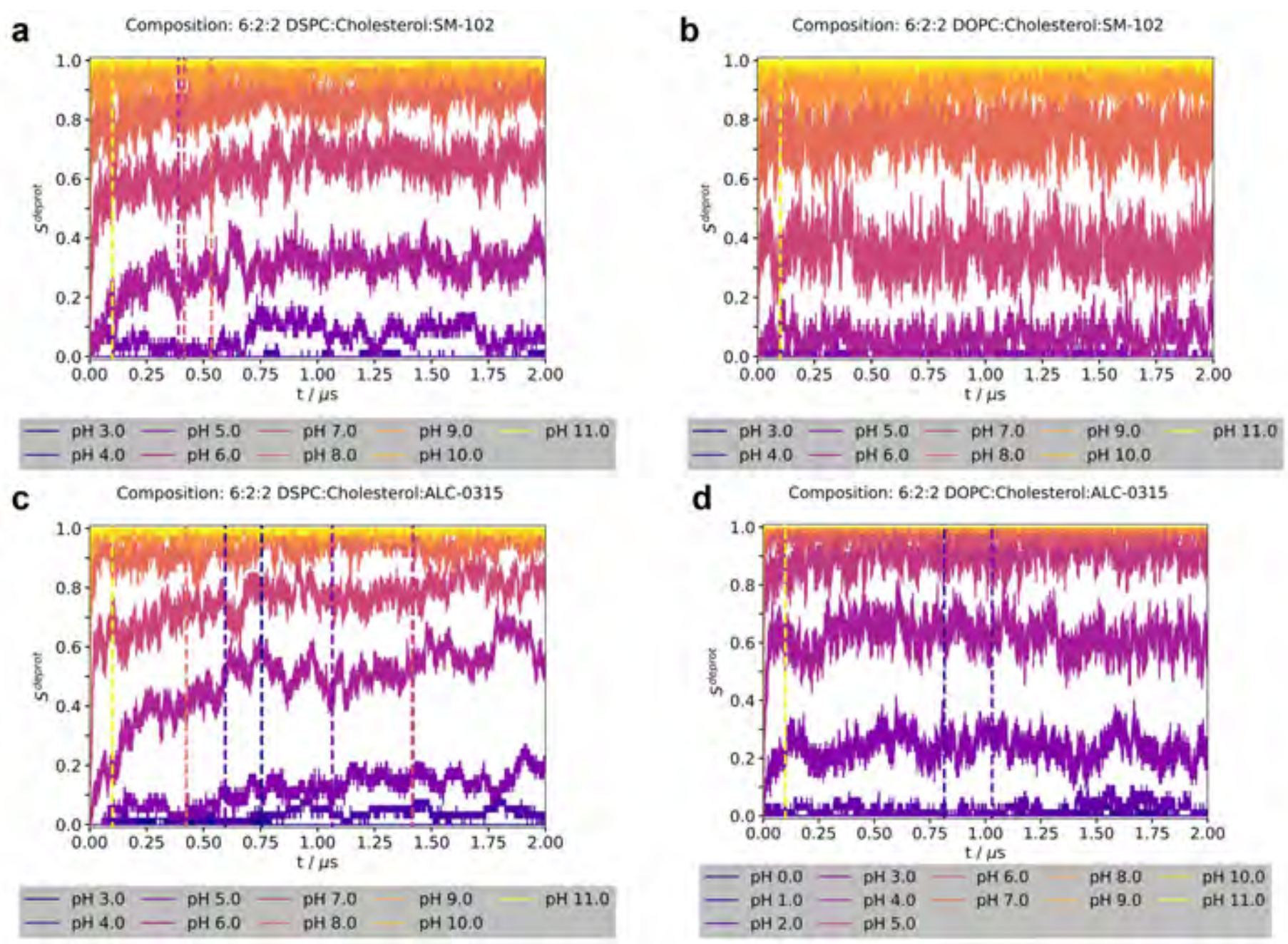

Figure S56: Fraction of deprotonated aminolipids (S^{deprot}) per frame for membranes with a 6:2:2 helper lipid:cholesterol:aminolipid composition containing either **a,c** DSPC or **b,d** DOPC as the helper lipid, and **a,b** SM-102, or **c,d** ALC-0315 as the aminolipid. Vertical dashed lines indicate the equilibration time determined for each pH value.

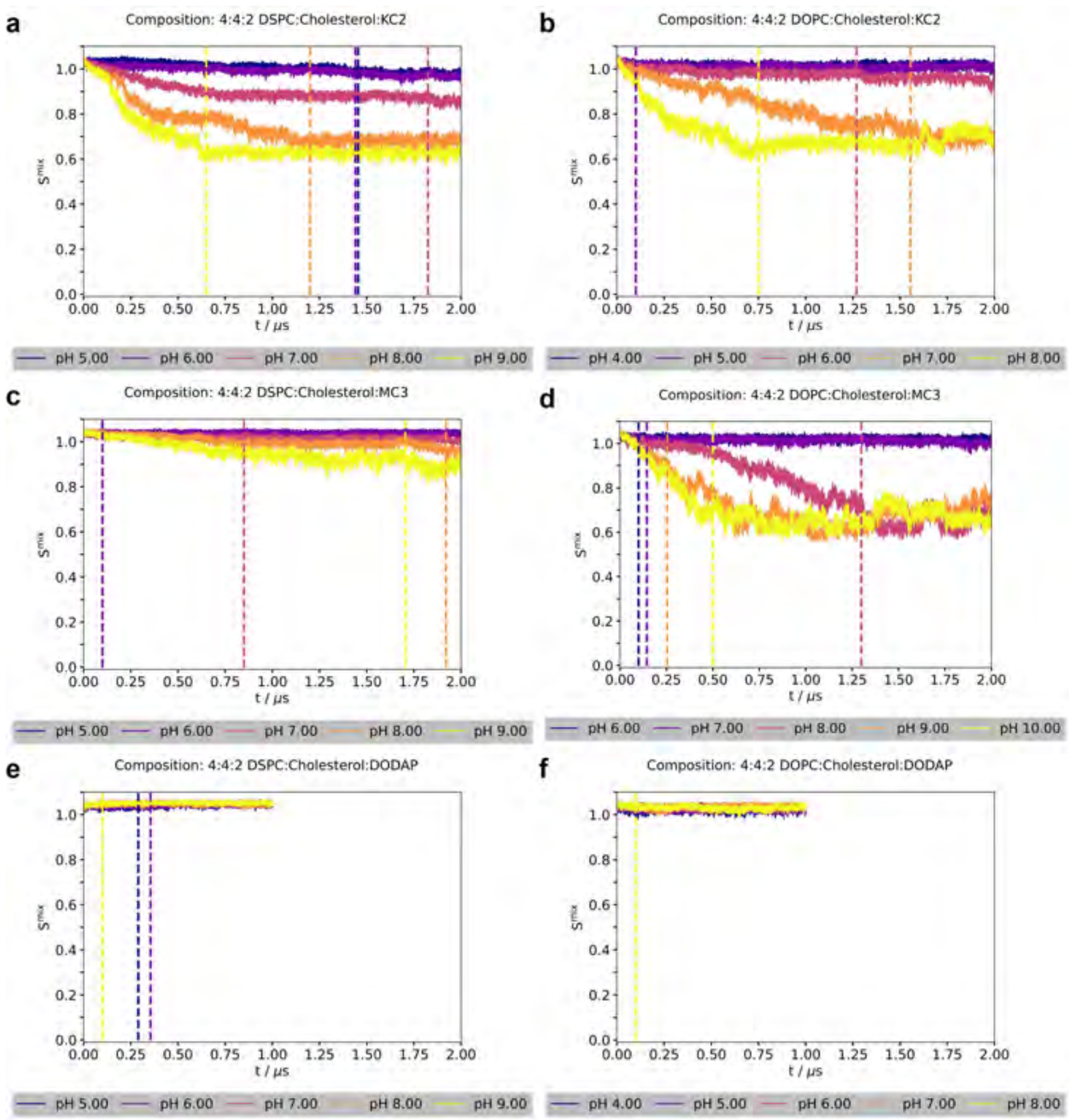

Figure S57: Conditional mixing entropy (S^{mix}) per frame for membranes with a 4:4:2 helper lipid:cholesterol:aminolipid composition containing either **a,c,e** DSPC or **b,d,f** DOPC as the helper lipid, and **a,b** DLin-KC2-DMA, **c,d** DLin-MC3-DMA, or **e,f** DODAP as the aminolipid. Vertical dashed lines indicate the equilibration time determined for each pH value. Conditional mixing entropies were calculated following Brandani *et al.*,[3] using a 1 nm cutoff to define neighboring lipids. Contacts were defined based on the distance between reference atoms in the headgroups of the target lipids: the titratable nitrogen for aminolipids, the oxygen atom for cholesterol, and the sn-2 carbon atom for phospholipids.

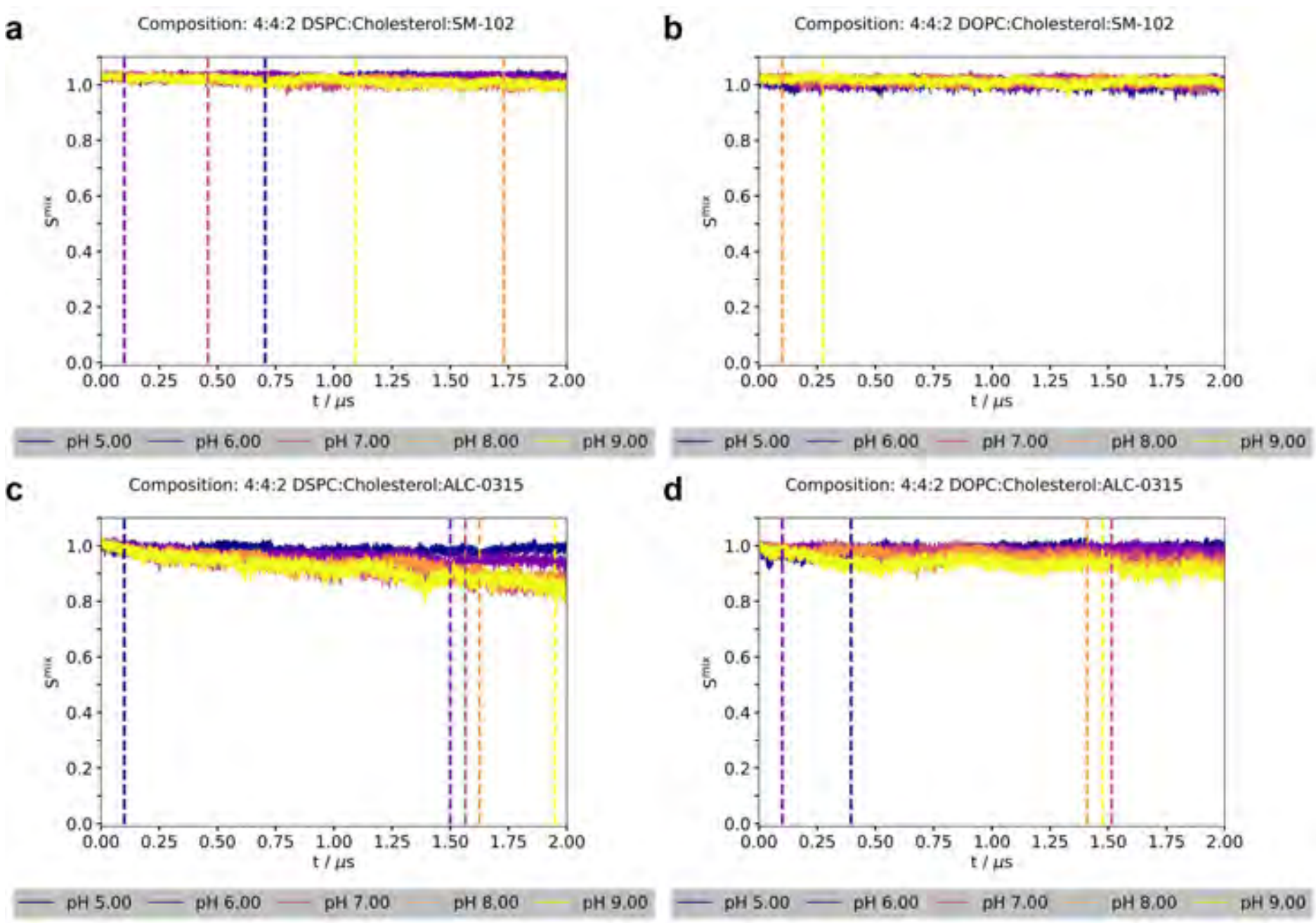

Figure S58: Conditional mixing entropy (S^{mix}) per frame for membranes with a 4:4:2 helper lipid:cholesterol:aminolipid composition containing either **a,c** DSPC or **b,d** DOPC as the helper lipid, and **a,b** SM-102, or **c,d** ALC-0315 as the aminolipid. Vertical dashed lines indicate the equilibration time determined for each pH value. Conditional mixing entropies were calculated following Brandani *et al.*,[3] using a 1 nm cutoff to define neighboring lipids. Contacts were defined based on the distance between reference atoms in the headgroups of the target lipids: the titratable nitrogen for aminolipids, the oxygen atom for cholesterol, and the sn-2 carbon atom for phospholipids.

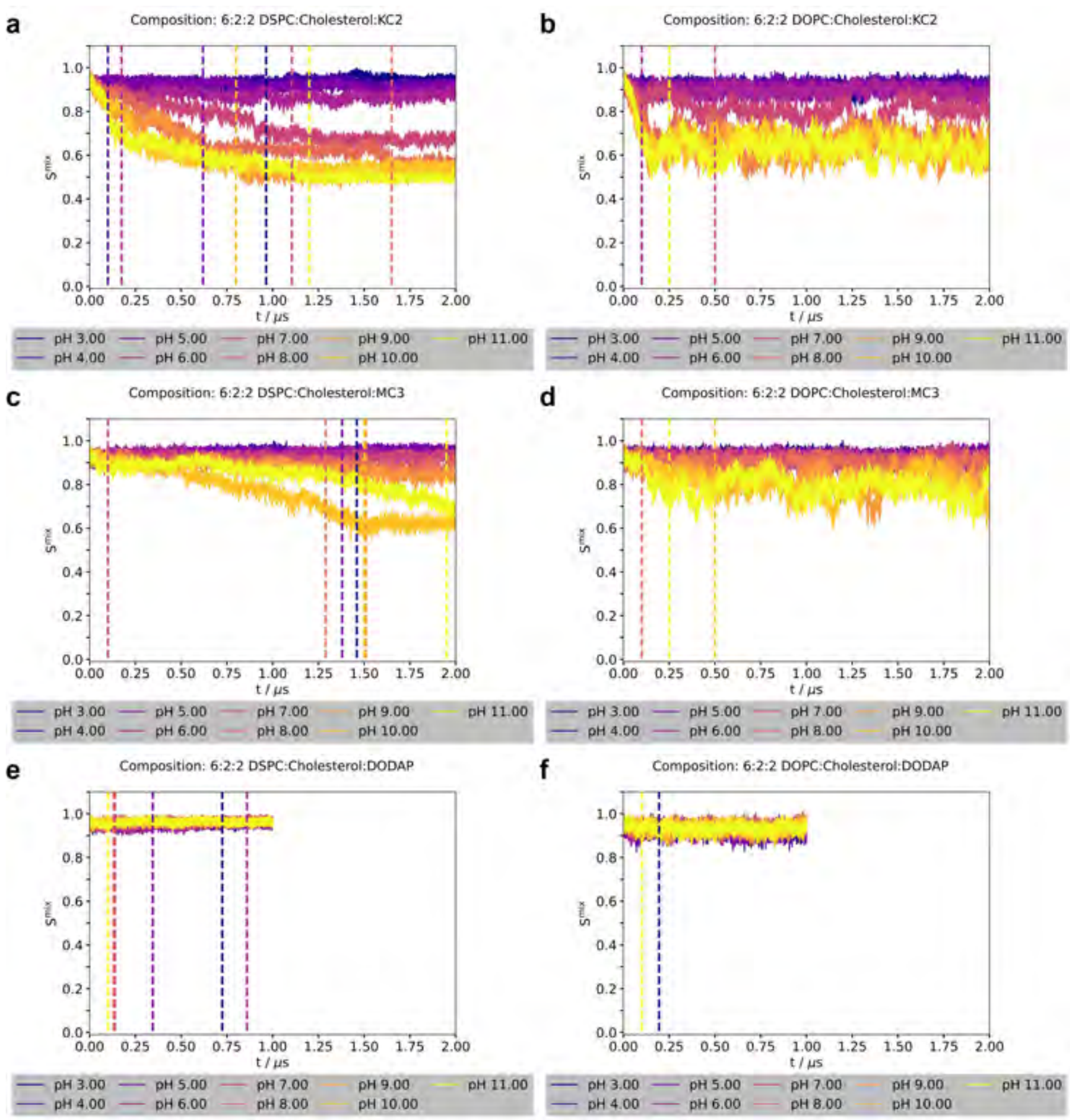

Figure S59: Conditional mixing entropy (S^{mix}) per frame for membranes with a 6:2:2 helper lipid:cholesterol:aminolipid composition containing either **a,c,e** DSPC or **b,d,f** DOPC as the helper lipid, and **a,b** DLin-KC2-DMA, **c,d** DLin-MC3-DMA, or **e,f** DODAP as the aminolipid. Vertical dashed lines indicate the equilibration time determined for each pH value. Conditional mixing entropies were calculated following Brandani *et al.*,[3] using a 1 nm cutoff to define neighboring lipids. Contacts were defined based on the distance between reference atoms in the headgroups of the target lipids: the titratable nitrogen for aminolipids, the oxygen atom for cholesterol, and the sn-2 carbon atom for phospholipids.

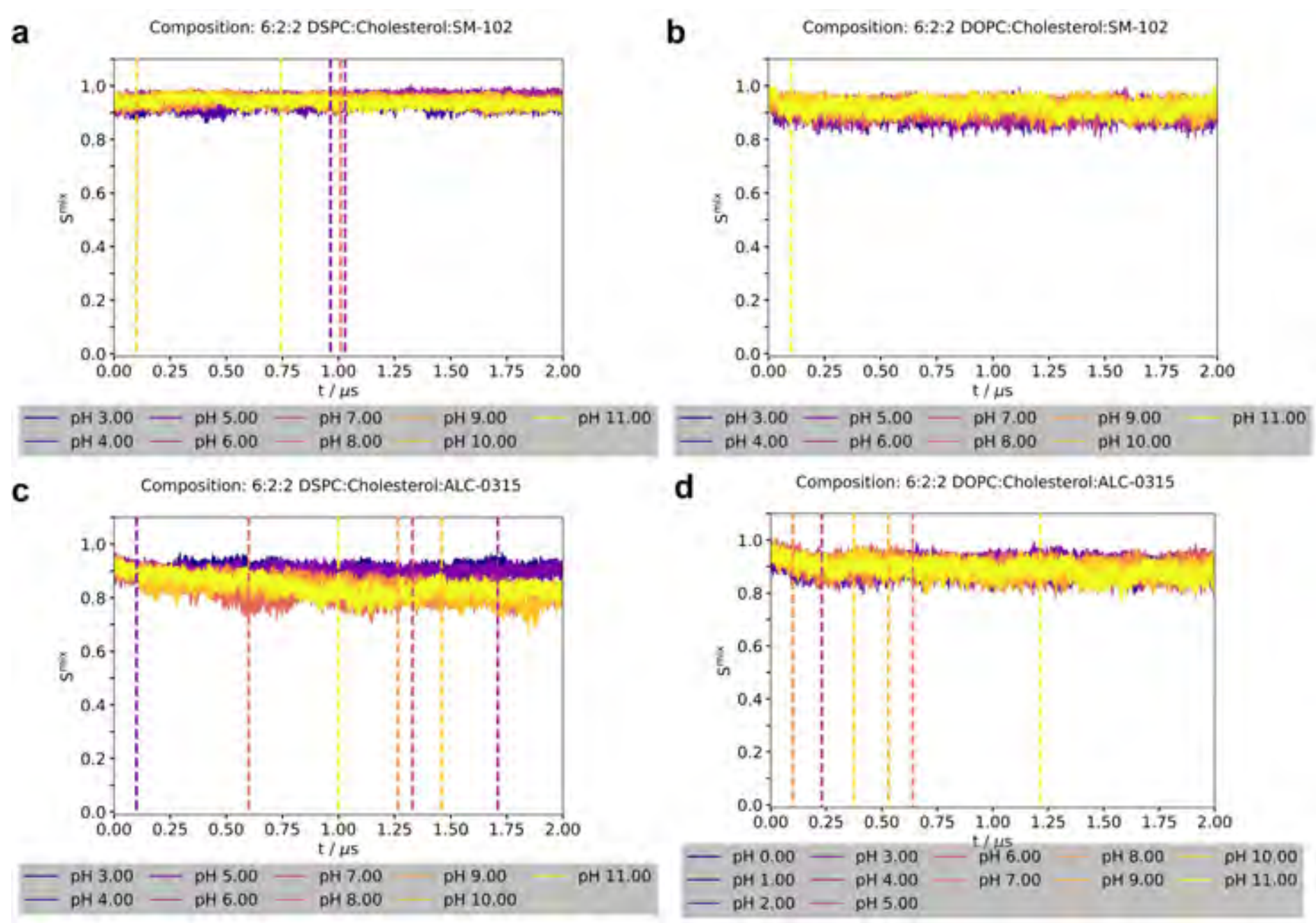

Figure S60: Conditional mixing entropy (S^{mix}) per frame for membranes with a 6:2:2 helper lipid:cholesterol:aminolipid composition containing either **a,c** DSPC or **b,d** DOPC as the helper lipid, and **a,b** SM-102, or **c,d** ALC-0315 as the aminolipid. Vertical dashed lines indicate the equilibration time determined for each pH value. Conditional mixing entropies were calculated following Brandani *et al.*,[3] using a 1 nm cutoff to define neighboring lipids. Contacts were defined based on the distance between reference atoms in the headgroups of the target lipids: the titratable nitrogen for aminolipids, the oxygen atom for cholesterol, and the sn-2 carbon atom for phospholipids.

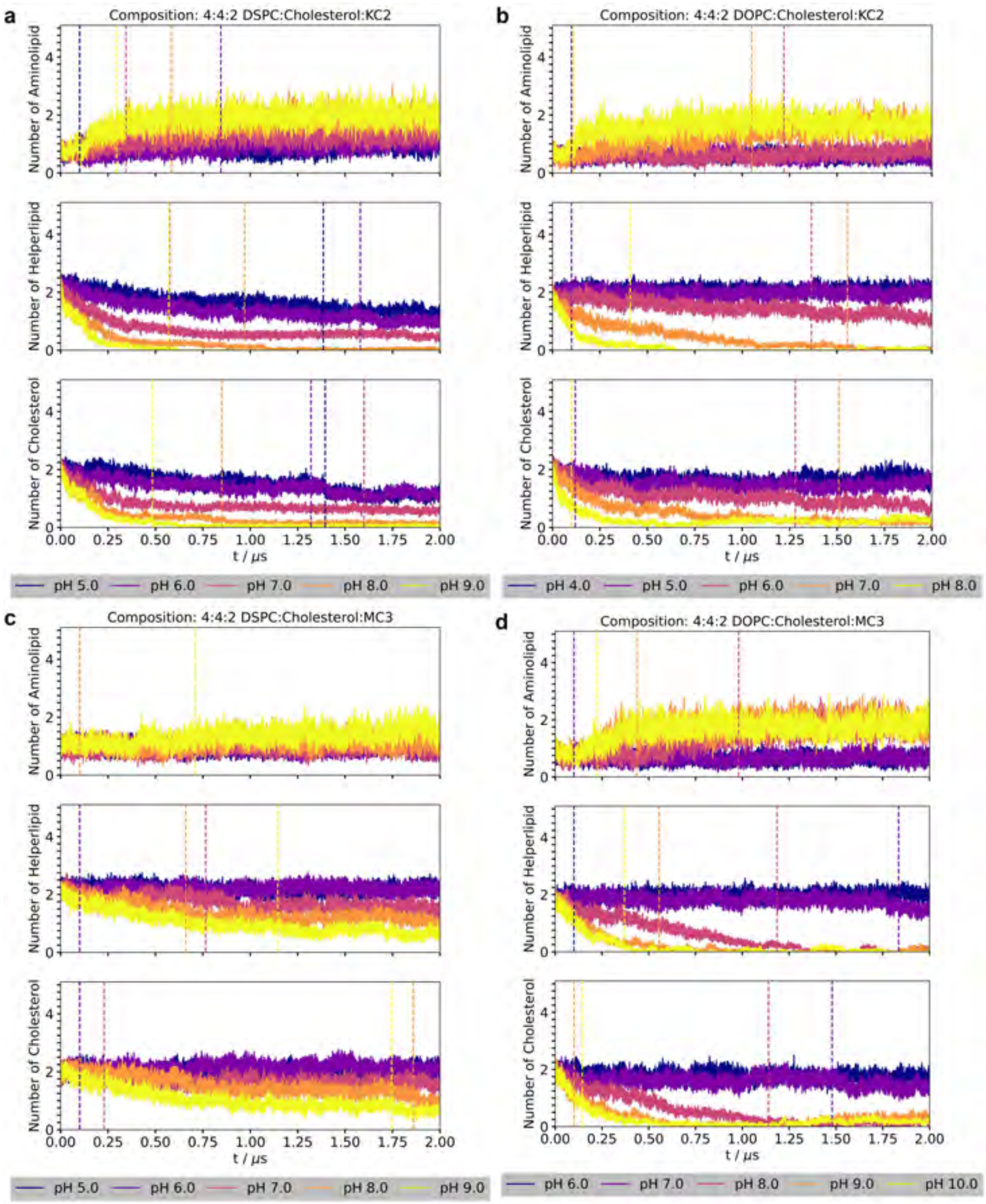

Figure S61: Number of neighboring lipids per aminolipid per frame for membranes with a 4:4:2 helper lipid:cholesterol:aminolipid composition containing either **a,c** DSPC or **b,d** DOPC as the helper lipid, and **a,b** DLin-KC2-DMA, or **c,d** DLin-MC3-DMA as the aminolipid. Vertical dashed lines indicate the equilibration time determined for each pH value. A cutoff distance of 1 nm was used to define neighboring lipids. Contacts were defined based on the distance between reference atoms in the lipid headgroups: the titratable nitrogen atom for aminolipids, the oxygen atom for cholesterol, and the sn-2 carbon atom for phospholipids.

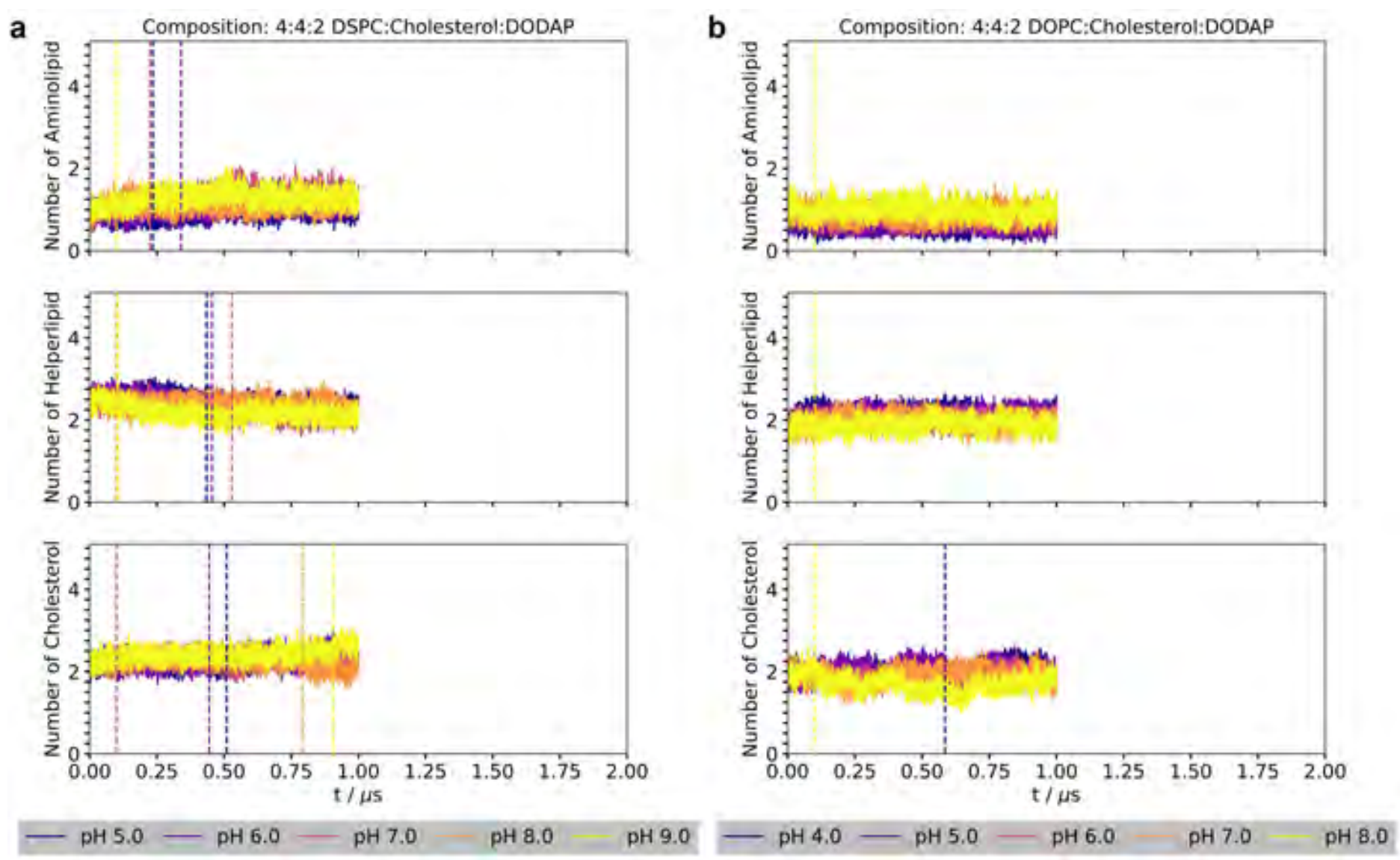

Figure S62: Number of neighboring lipids per aminolipid per frame for membranes with a 4:4:2 helper lipid:cholesterol:aminolipid composition containing either **a** DSPC or **b** DOPC as the helper lipid, and DODAP as the aminolipid. Vertical dashed lines indicate the equilibration time determined for each pH value. A cutoff distance of 1 nm was used to define neighboring lipids. Contacts were defined based on the distance between reference atoms in the lipid headgroups: the titratable nitrogen atom for aminolipids, the oxygen atom for cholesterol, and the sn-2 carbon atom for phospholipids.

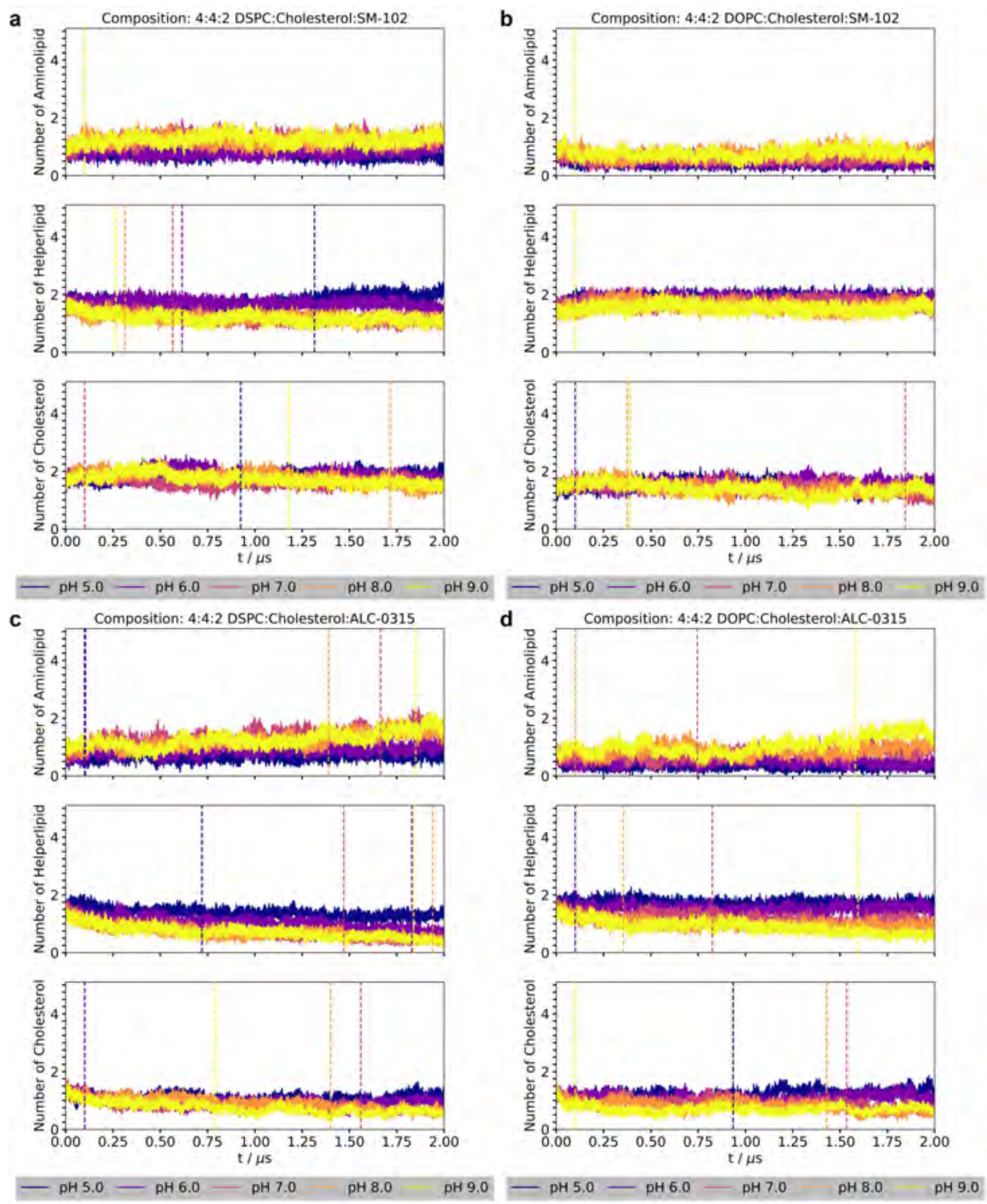

Figure S63: Number of neighboring lipids per aminolipid per frame for membranes with a 4:4:2 helper lipid:cholesterol:aminolipid composition containing either **a,c** DSPC or **b,d** DOPC as the helper lipid, and **a,b** SM-102, or **c,d** ALC-0315 as the aminolipid. Vertical dashed lines indicate the equilibration time determined for each pH value. A cutoff distance of 1 nm was used to define neighboring lipids. Contacts were defined based on the distance between reference atoms in the lipid headgroups: the titratable nitrogen atom for aminolipids, the oxygen atom for cholesterol, and the sn-2 carbon atom for phospholipids.

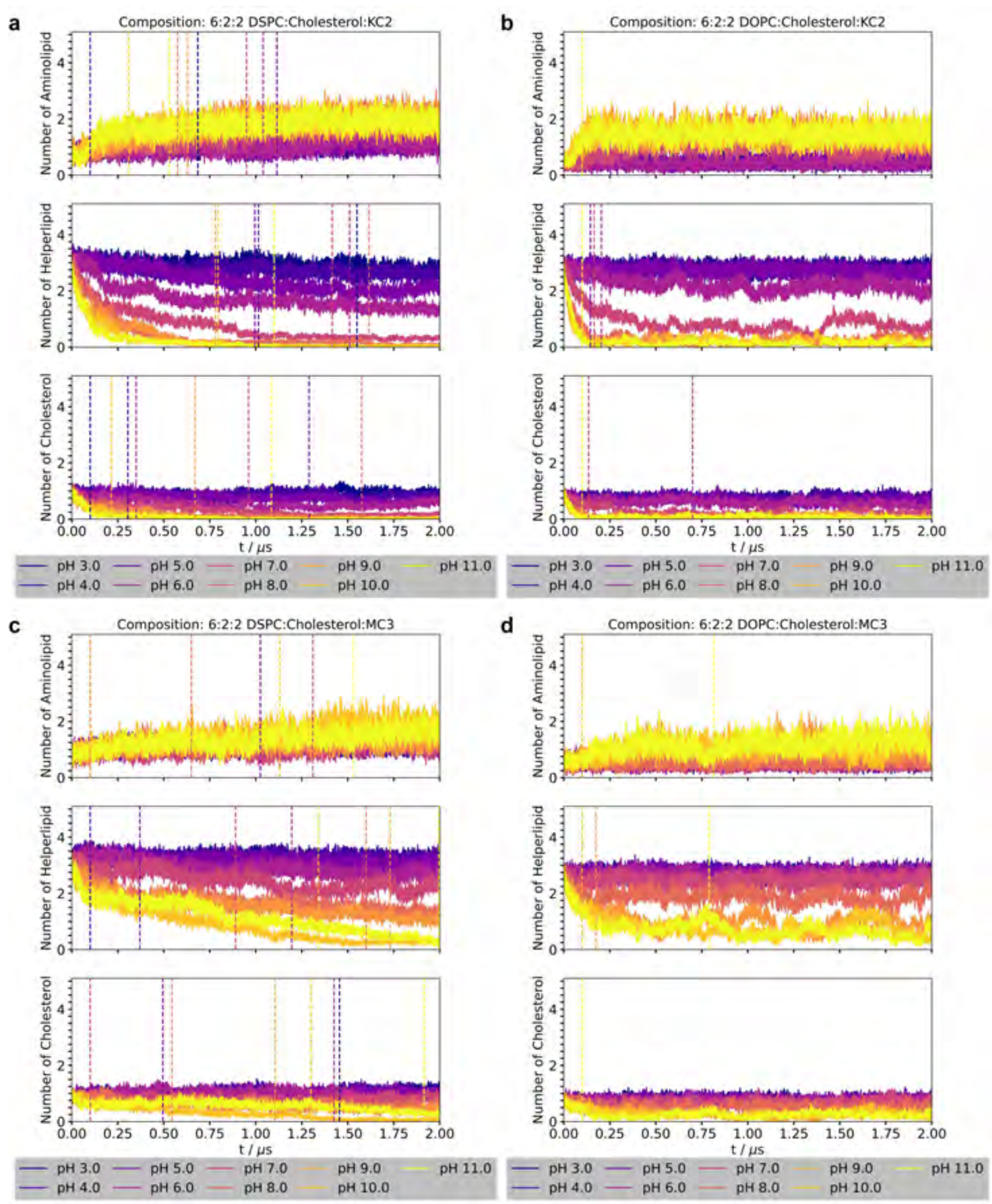

Figure S64: Number of neighboring lipids per aminolipid per frame for membranes with a 6:2:2 helper lipid:cholesterol:aminolipid composition containing either **a,c** DSPC or **b,d** DOPC as the helper lipid, and **a,b** DLin-KC2-DMA, or **c,d** DLin-MC3-DMA as the aminolipid. Vertical dashed lines indicate the equilibration time determined for each pH value. A cutoff distance of 1 nm was used to define neighboring lipids. Contacts were defined based on the distance between reference atoms in the lipid headgroups: the titratable nitrogen atom for aminolipids, the oxygen atom for cholesterol, and the sn-2 carbon atom for phospholipids.

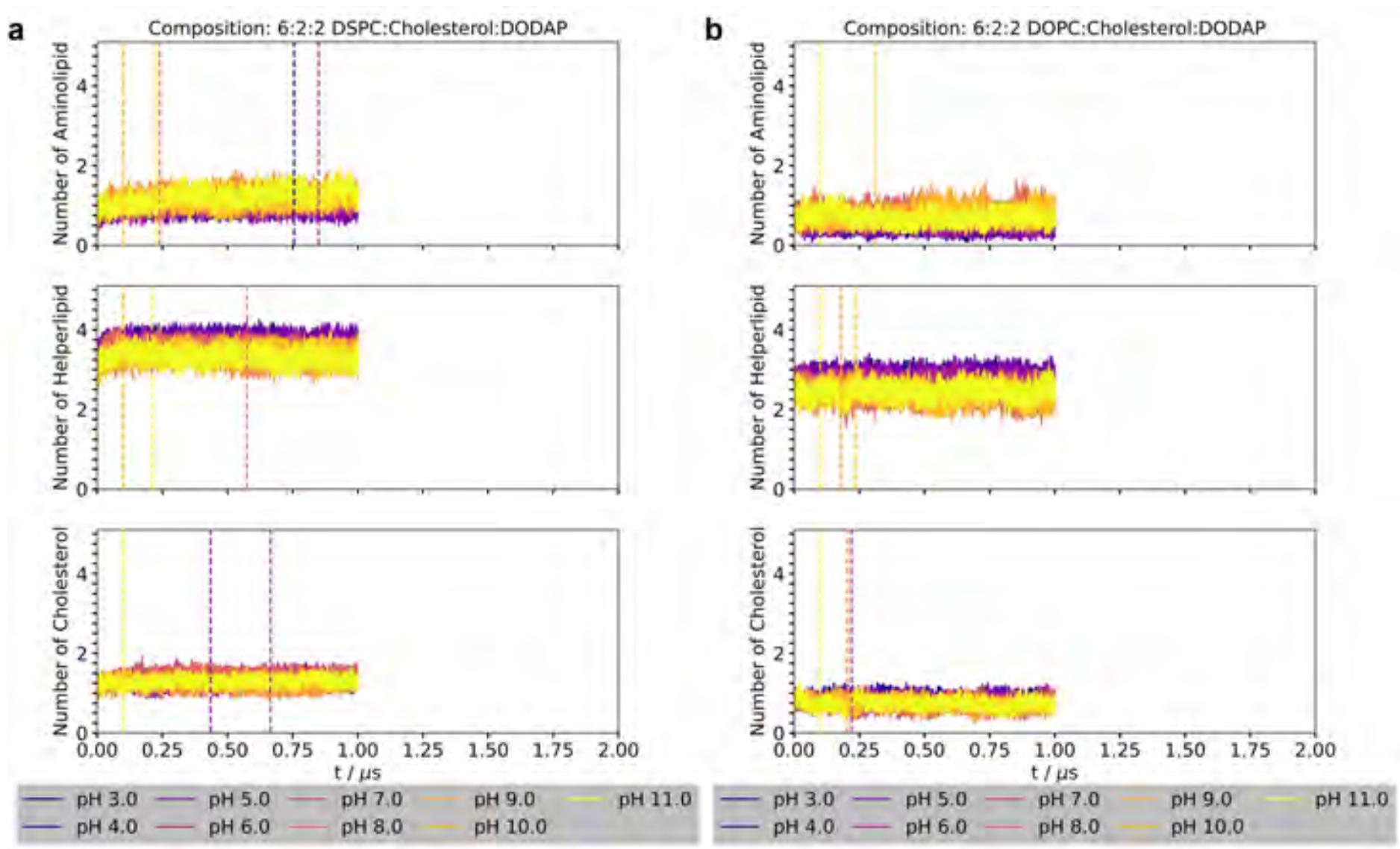

Figure S65: Number of neighboring lipids per aminolipid per frame for membranes with a 6:2:2 helper lipid:cholesterol:aminolipid composition containing either **a** DSPC or **b** DOPC as the helper lipid, and DODAP as the aminolipid. Vertical dashed lines indicate the equilibration time determined for each pH value. A cutoff distance of 1 nm was used to define neighboring lipids. Contacts were defined based on the distance between reference atoms in the lipid headgroups: the titratable nitrogen atom for aminolipids, the oxygen atom for cholesterol, and the sn-2 carbon atom for phospholipids.

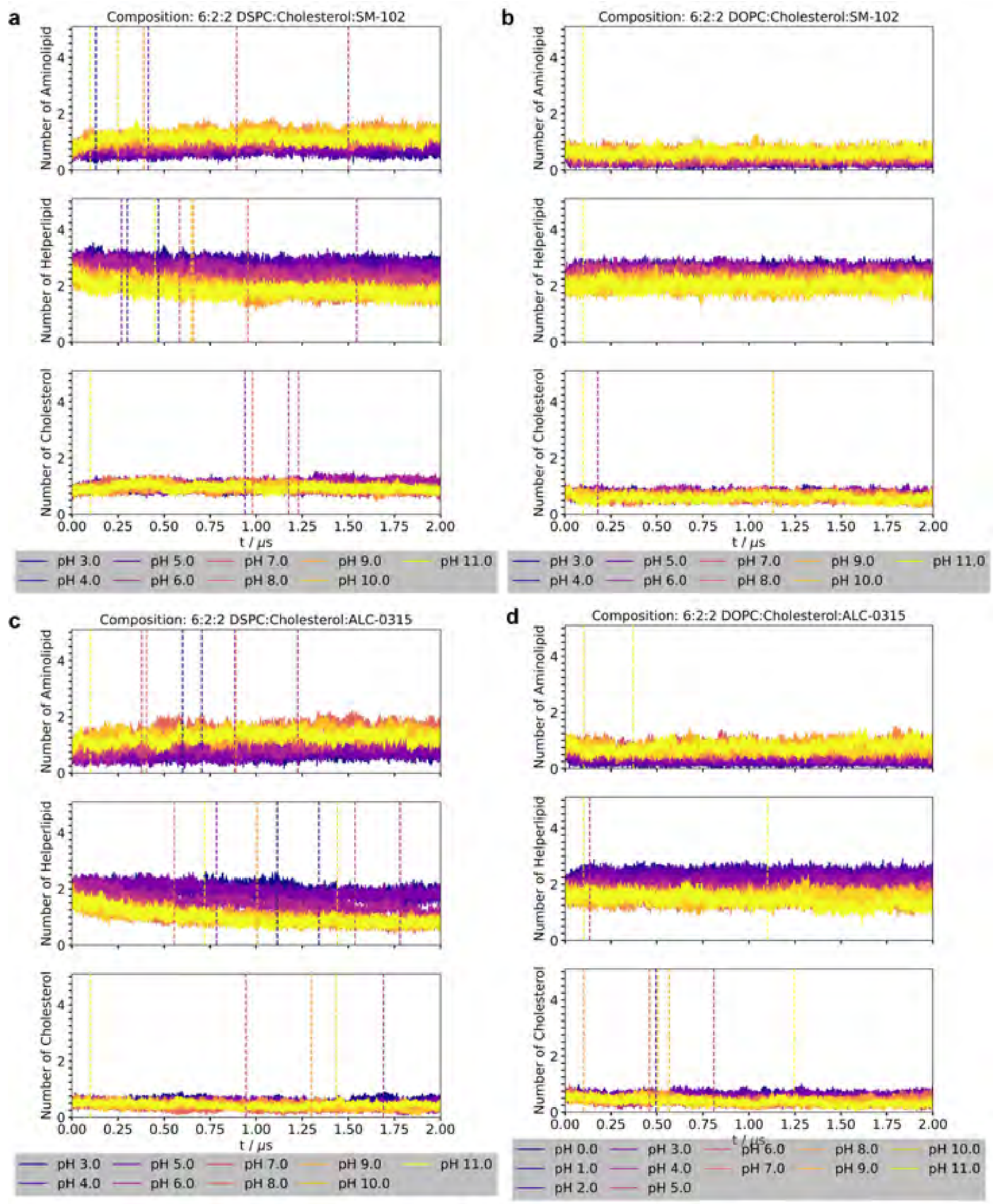

Figure S66: Number of neighboring lipids per aminolipid per frame for membranes with a 6:2:2 helper lipid:cholesterol:aminolipid composition containing either **a,c** DSPC or **b,d** DOPC as the helper lipid, and **a,b** SM-102, or **c,d** ALC-0315 as the aminolipid. Vertical dashed lines indicate the equilibration time determined for each pH value. A cutoff distance of 1 nm was used to define neighboring lipids. Contacts were defined based on the distance between reference atoms in the lipid headgroups: the titratable nitrogen atom for aminolipids, the oxygen atom for cholesterol, and the sn-2 carbon atom for phospholipids.

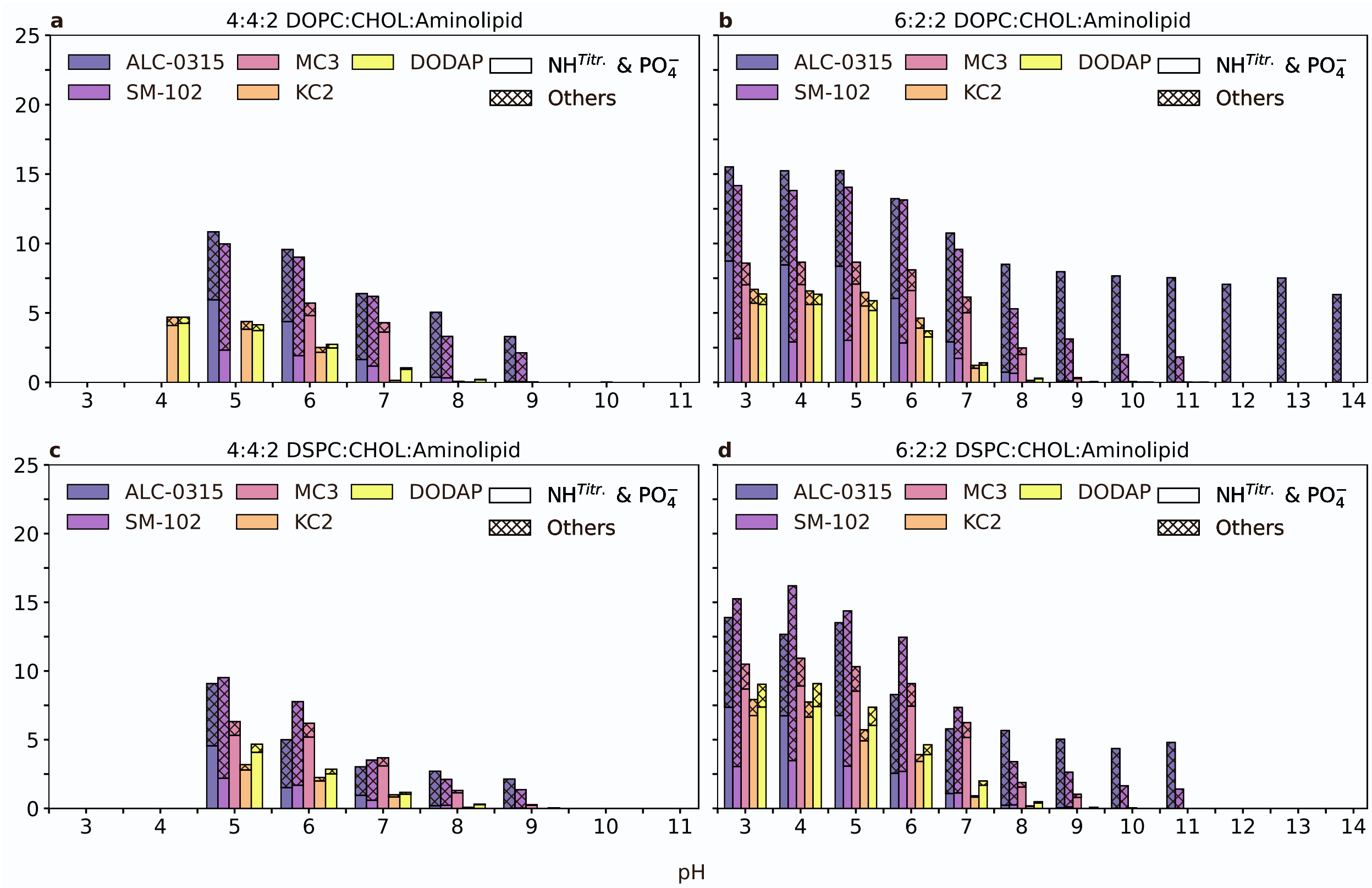

Figure S67: Average number of hydrogen bonds between aminolipids and helper lipids as a function of pH for DOPC-based (top) or DSPC-based (bottom) membranes containing **a**,**c** 40 mol% cholesterol and **b**,**d** 20 mol% cholesterol. Hydrogen bonds were separated into interactions between the titratable site of the aminolipids and phospholipid phosphate groups ("NH$^{Titr.}$ & PO_4^-"), and all remaining hydrogen bonds **not** involving this specific donor-acceptor pair ("Others"). Hydrogen bonds were identified using the MDAnalysis library (v2.7.0) with a distance cutoff of 3.0 Å and an angle cutoff of 150°.

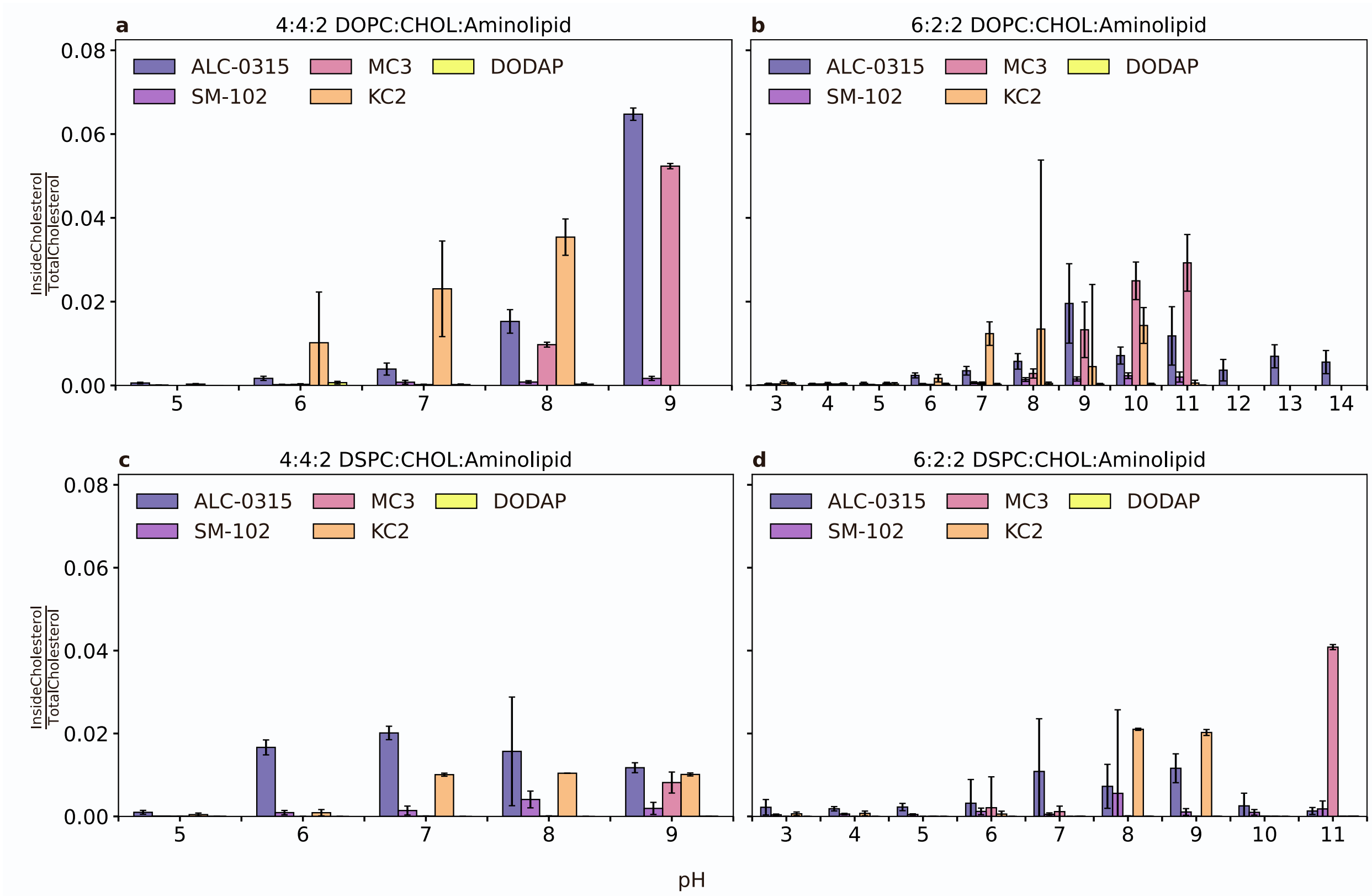

Figure S68: Fraction of cholesterol located within the membrane core as a function of pH for DOPC-based (top) or DSPC-based (bottom) membranes containing **a**,**c** 40 mol% cholesterol and **b**,**d** 20 mol% cholesterol. Cholesterol positions were determined from the z-coordinates of the cholesterol hydroxyl oxygen. Cholesterol molecules were assigned to the membrane core if the reference atom was located more than 1.8 nm from the median phosphorus position of the respective leaflet. Error bars represent the standard error of the mean obtained by block averaging. Abbreviations used: CHOL = cholesterol.

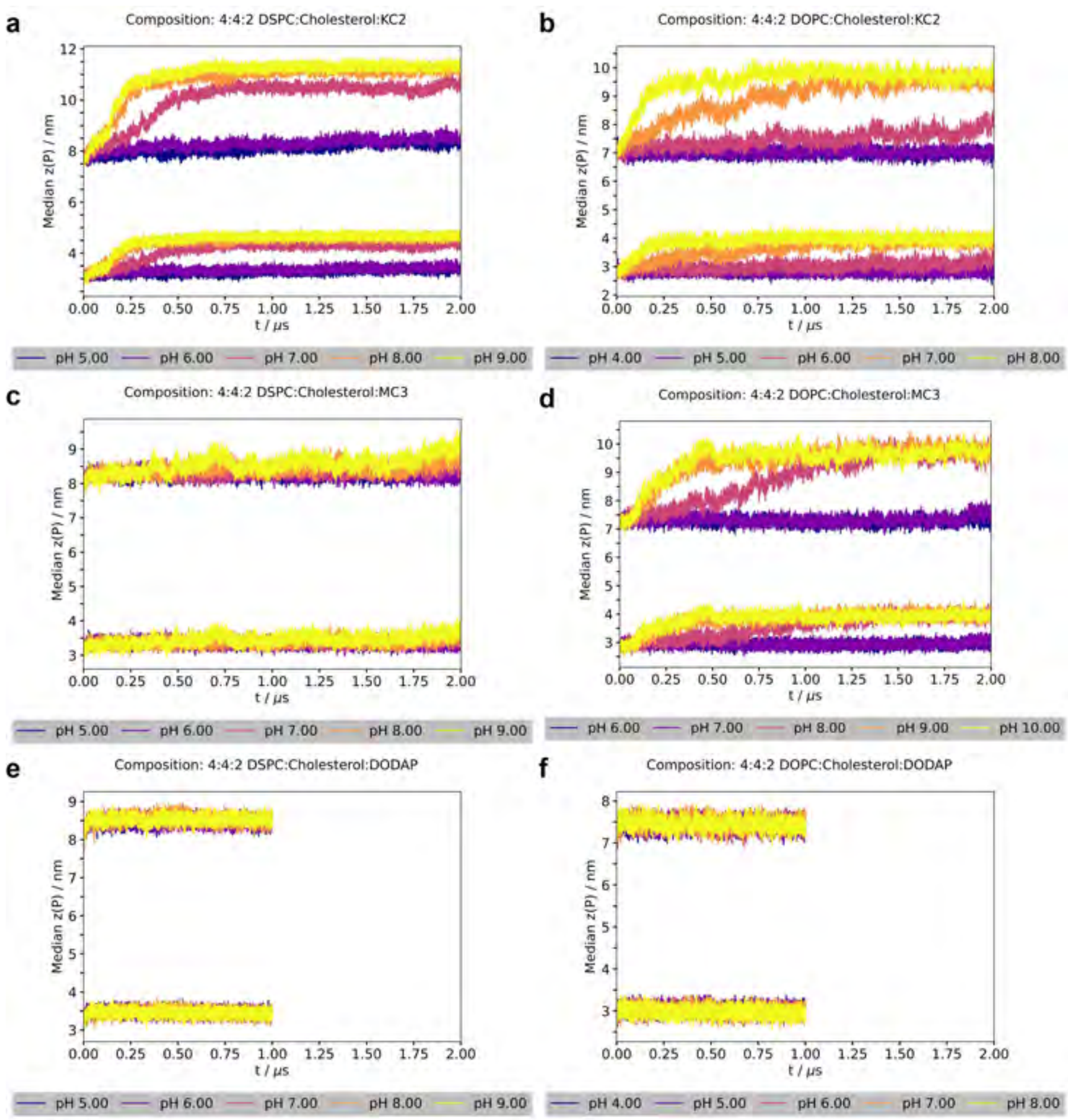

Figure S69: Average z-position of the phosphorus atoms in the upper and lower leaflets per frame for systems with a 4:4:2 helper lipid:cholesterol:aminolipid composition containing either **a,c,e** DSPC or **b,d,f** DOPC as the helper lipid, and **a,b** DLin-KC2-DMA, **c,d** DLin-MC3-DMA or **e,f** DODAP as the aminolipid. Average z-positions were computed using a periodic trigonometric approach (see Methods) to mitigate artifacts arising when the membrane is split across periodic boundary conditions.

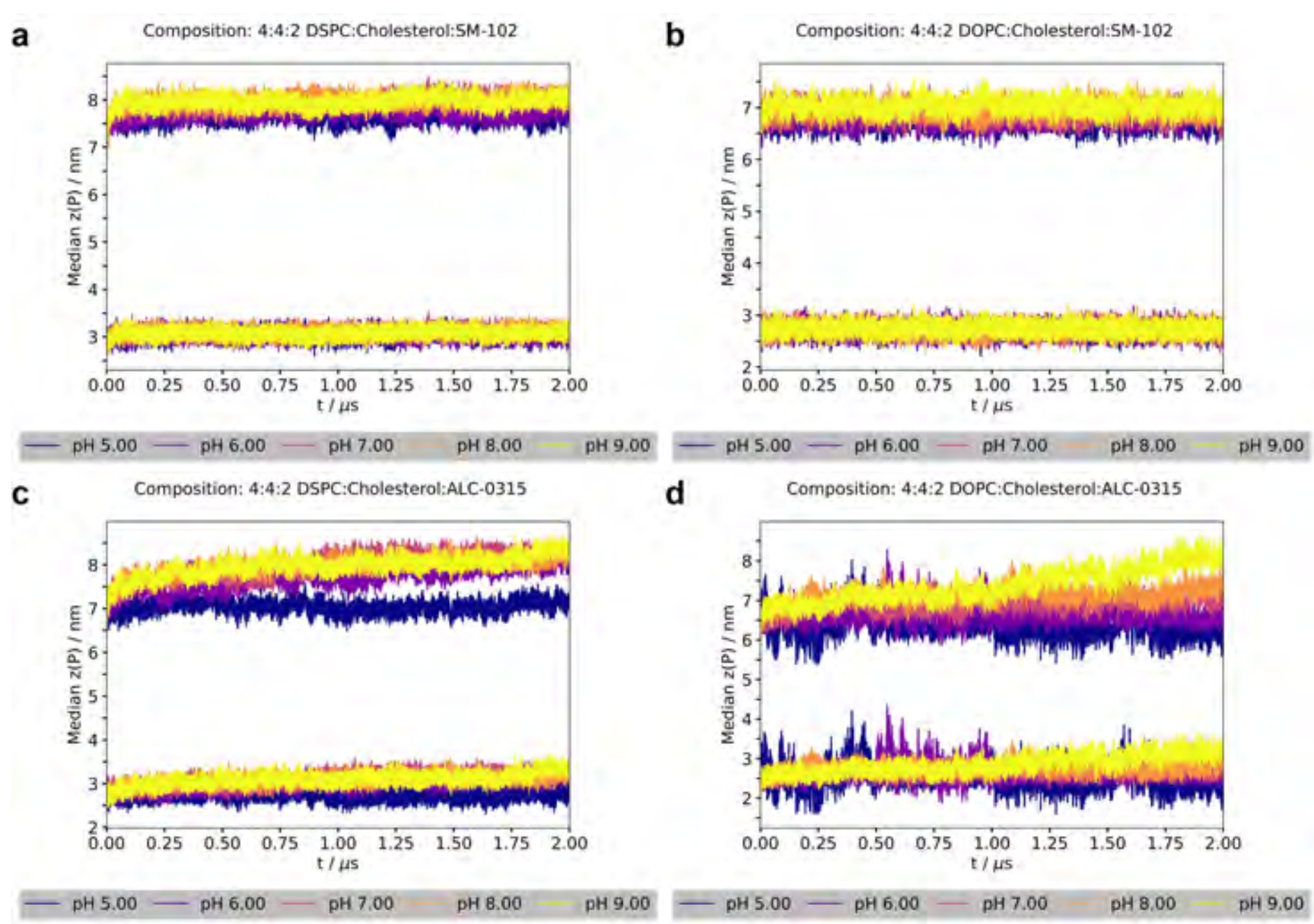

Figure S70: Average z-position of the phosphorus atoms in the upper and lower leaflets per frame for systems with a 4:4:2 helper lipid:cholesterol:aminolipid composition containing either **a,c** DSPC or **b,d** DOPC as the helper lipid, and **a,b** SM-102, or **c,d** ALC-0315 as the aminolipid. Average z-positions were computed using a periodic trigonometric approach (see Methods) to mitigate artifacts arising when the membrane is split across periodic boundary conditions.

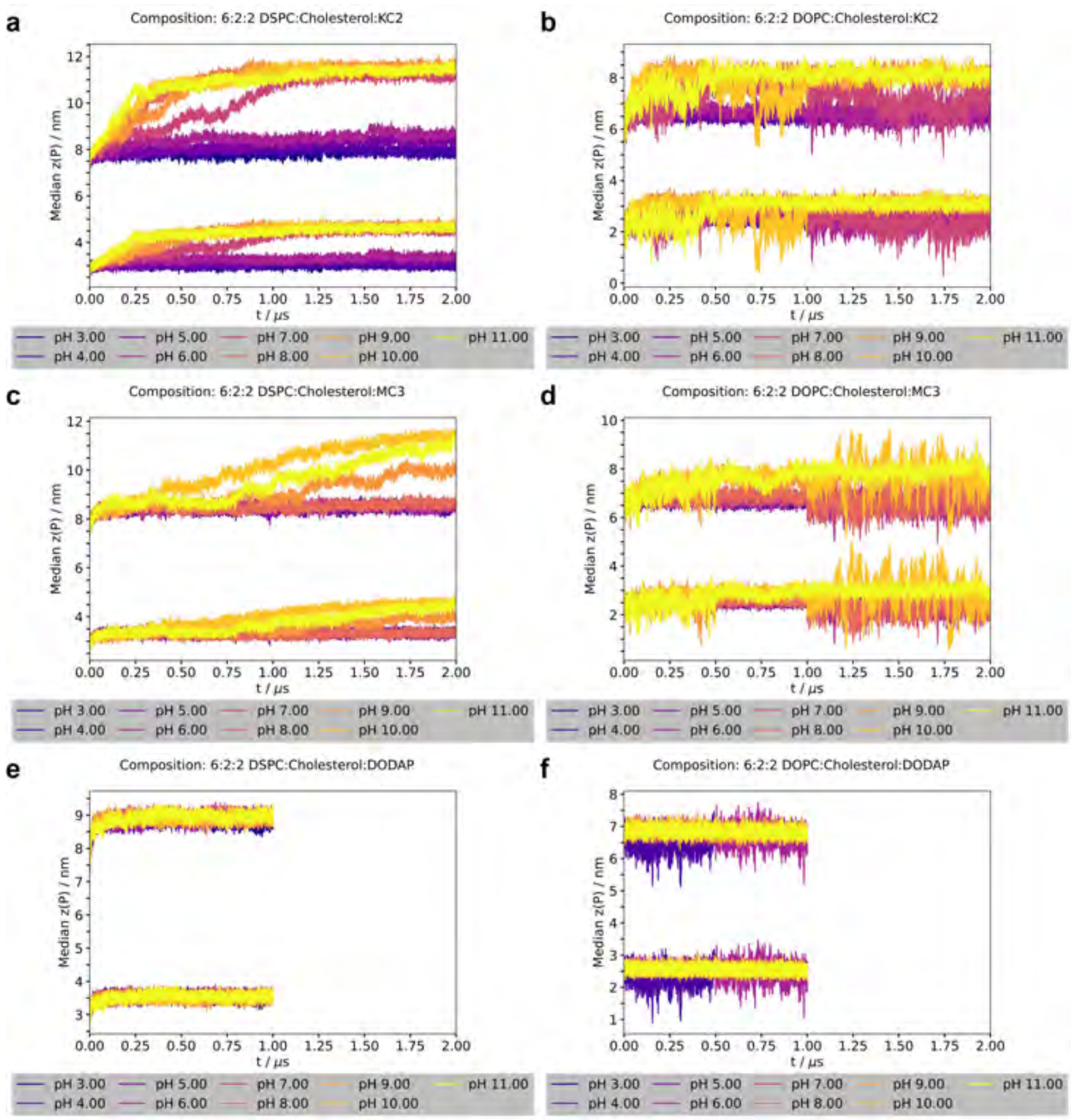

Figure S71: Average z-position of the phosphorus atoms in the upper and lower leaflets per frame for systems with a 6:2:2 helper lipid:cholesterol:aminolipid composition containing either **a,c,e** DSPC or **b,d,f** DOPC as the helper lipid, and **a,b** DLin-KC2-DMA, **c,d** DLin-MC3-DMA or **e,f** DODAP as the aminolipid. Average z-positions were computed using a periodic trigonometric approach (see Methods) to mitigate artifacts arising when the membrane is split across periodic boundary conditions.

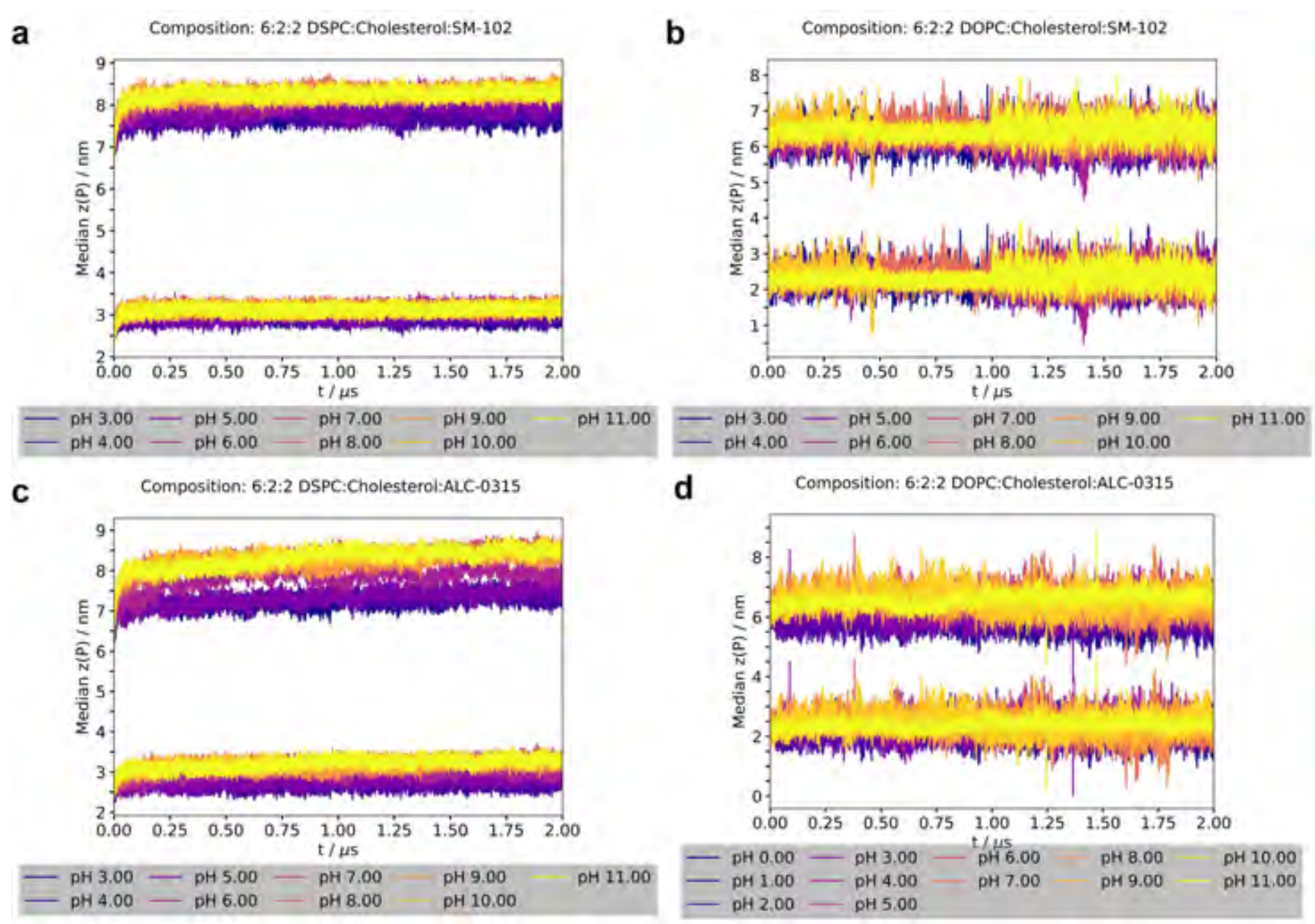

Figure S72: Average z-position of the phosphorus atoms in the upper and lower leaflets per frame for systems with a 6:2:2 helper lipid:cholesterol:aminolipid composition containing either **a,c** DSPC or **b,d** DOPC as the helper lipid, and **a,b** SM-102, or **c,d** ALC-0315 as the aminolipid. Average z-positions were computed using a periodic trigonometric approach (see Methods) to mitigate artifacts arising when the membrane is split across periodic boundary conditions.

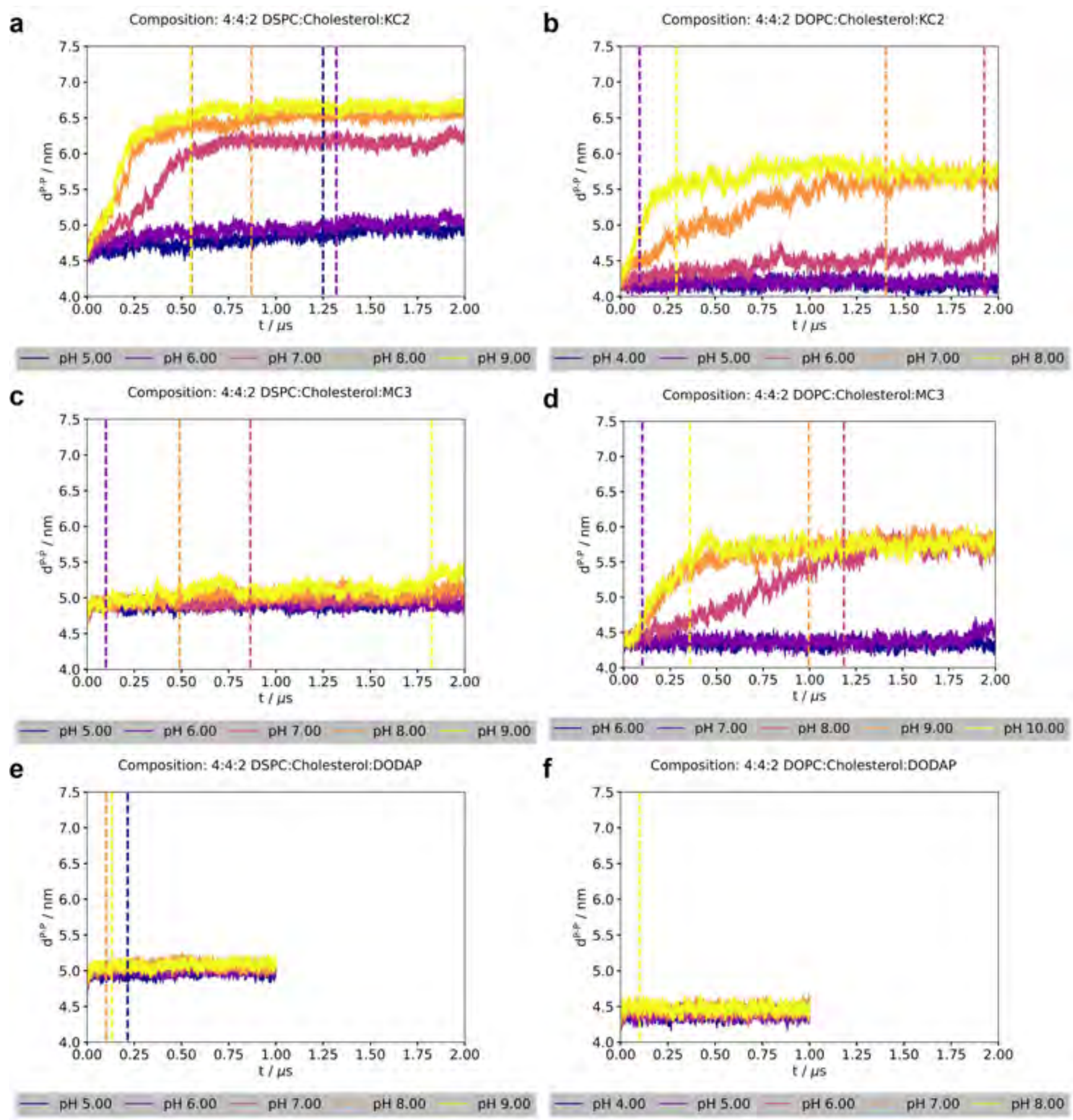

Figure S73: Membrane thickness per frame for systems with a 4:4:2 helper lipid:cholesterol:aminolipid composition containing either **a,c,e** DSPC or **b,d,f** DOPC as the helper lipid, and **a,b** DLin-KC2-DMA, **c,d** DLin-MC3-DMA or **e,f** DODAP as the aminolipid. Thickness is defined as the distance between the average z-positions of the phosphorus atoms in the upper and lower leaflets. Vertical dashed lines indicate the equilibration time determined for each pH value. Average z-positions were computed using a periodic trigonometric approach (see Methods) to mitigate artifacts arising when the membrane is split across periodic boundary conditions.

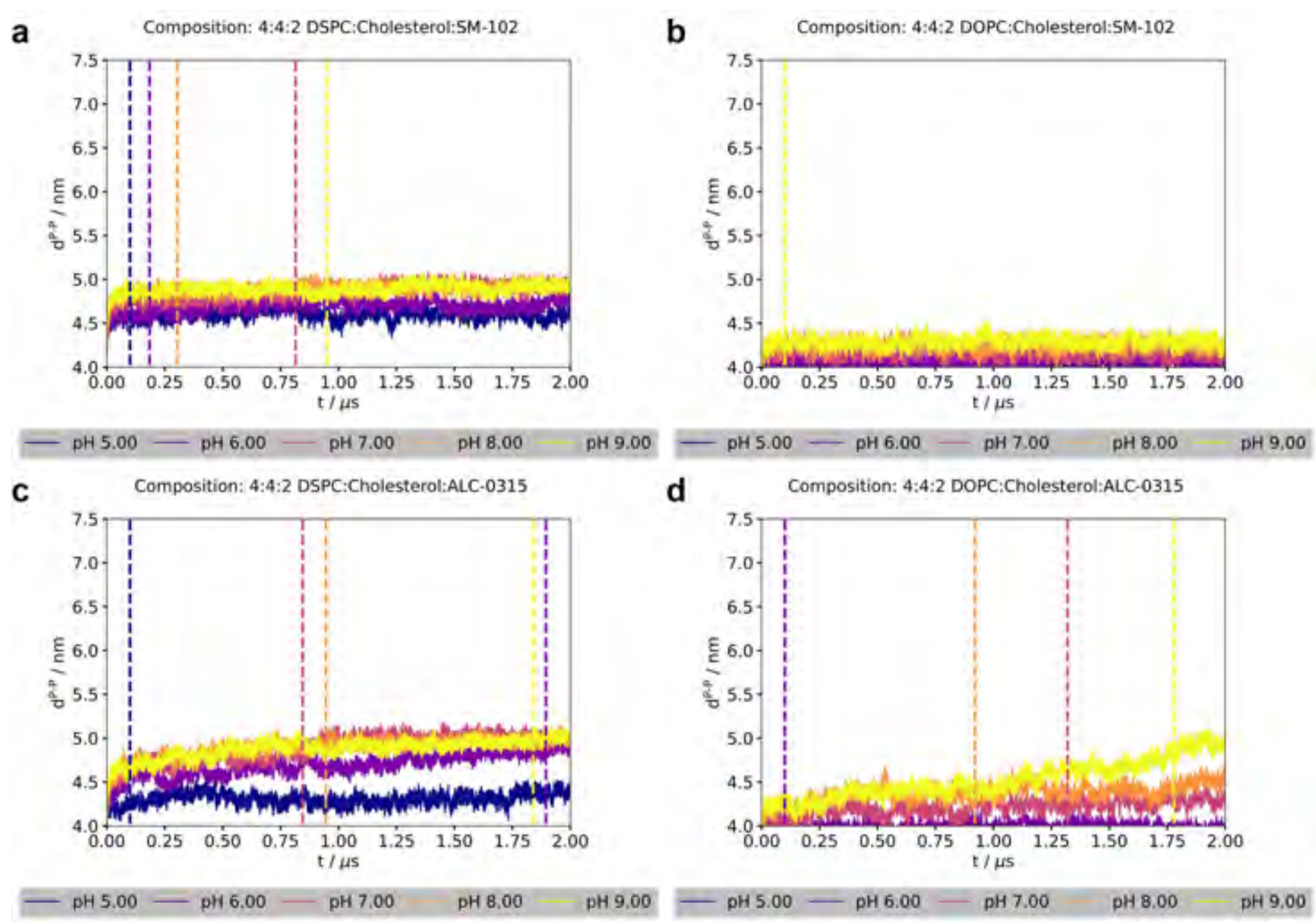

Figure S74: Membrane thickness per frame for systems with a 4:4:2 helper lipid:cholesterol:aminolipid composition containing either **a,c** DSPC or **b,d** DOPC as the helper lipid, and **a,b** SM-102, or **c,d** ALC-0315 as the aminolipid. Thickness is defined as the distance between the average z-positions of the phosphorus atoms in the upper and lower leaflets. Vertical dashed lines indicate the equilibration time determined for each pH value. Average z-positions were computed using a periodic trigonometric approach (see Methods) to mitigate artifacts arising when the membrane is split across periodic boundary conditions.

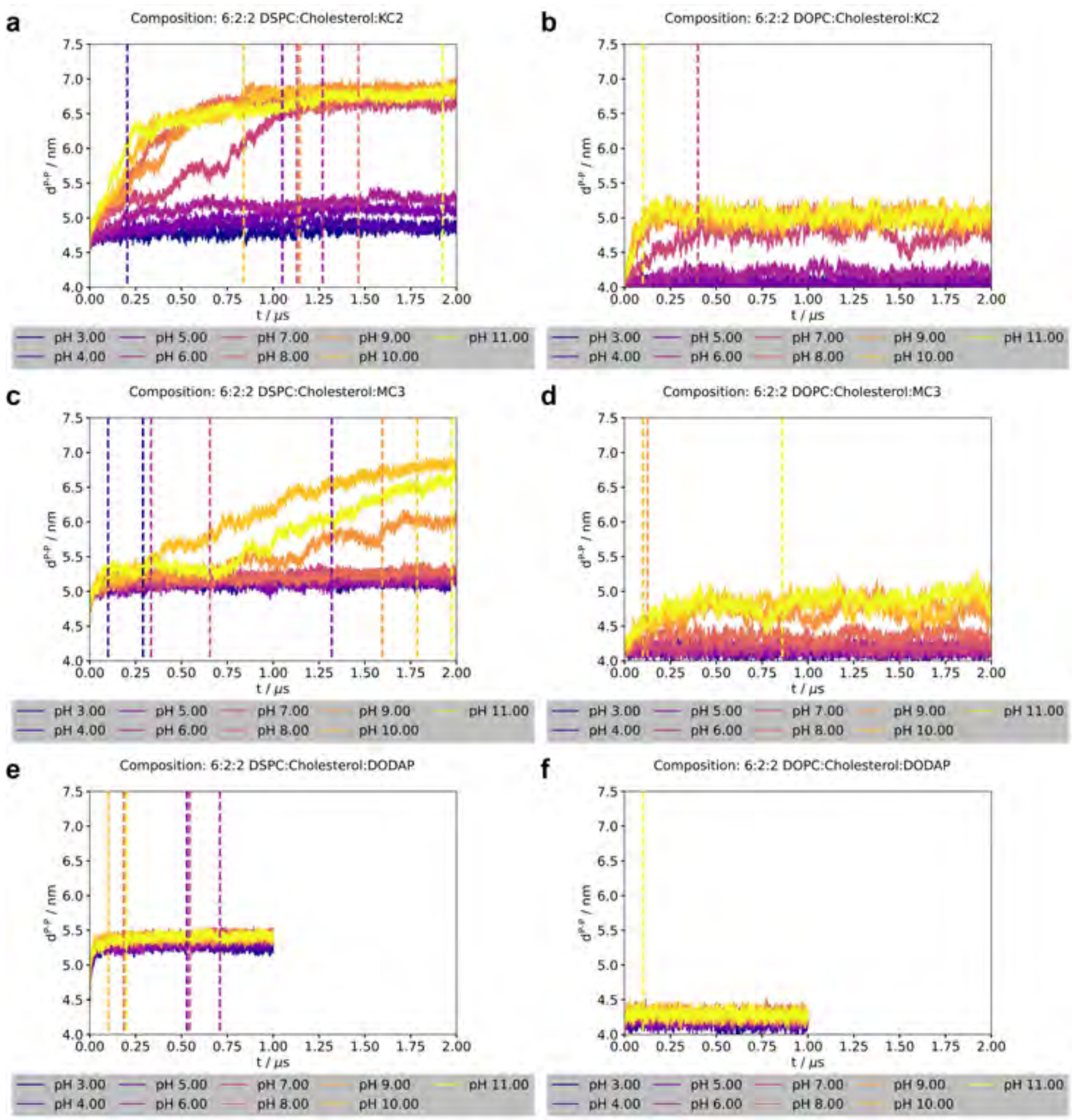

Figure S75: Membrane thickness per frame for systems with a 6:2:2 helper lipid:cholesterol:aminolipid composition containing either **a,c,e** DSPC or **b,d,f** DOPC as the helper lipid, and **a,b** DLin-KC2-DMA, **c,d** DLin-MC3-DMA or **e,f** DODAP as the aminolipid. Thickness is defined as the distance between the average z-positions of the phosphorus atoms in the upper and lower leaflets. Vertical dashed lines indicate the equilibration time determined for each pH value. Average z-positions were computed using a periodic trigonometric approach (see Methods) to mitigate artifacts arising when the membrane is split across periodic boundary conditions.

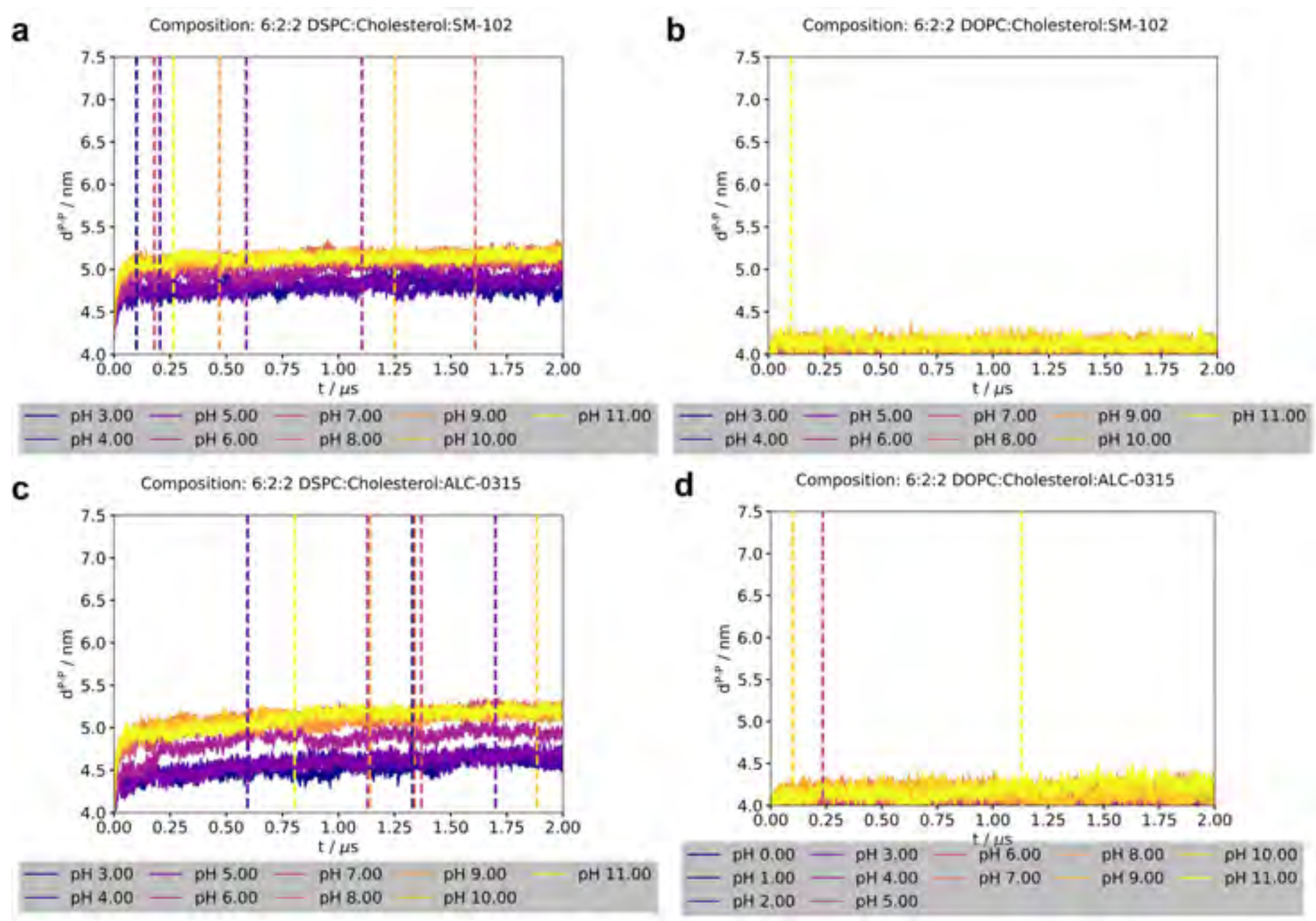

Figure S76: Membrane thickness per frame for systems with a 6:2:2 helper lipid:cholesterol:aminolipid composition containing either **a,c** DSPC or **b,d** DOPC as the helper lipid, and **a,b** SM-102, or **c,d** ALC-0315 as the aminolipid. Thickness is defined as the distance between the average z-positions of the phosphorus atoms in the upper and lower leaflets. Vertical dashed lines indicate the equilibration time determined for each pH value. Average z-positions were computed using a periodic trigonometric approach (see Methods) to mitigate artifacts arising when the membrane is split across periodic boundary conditions.

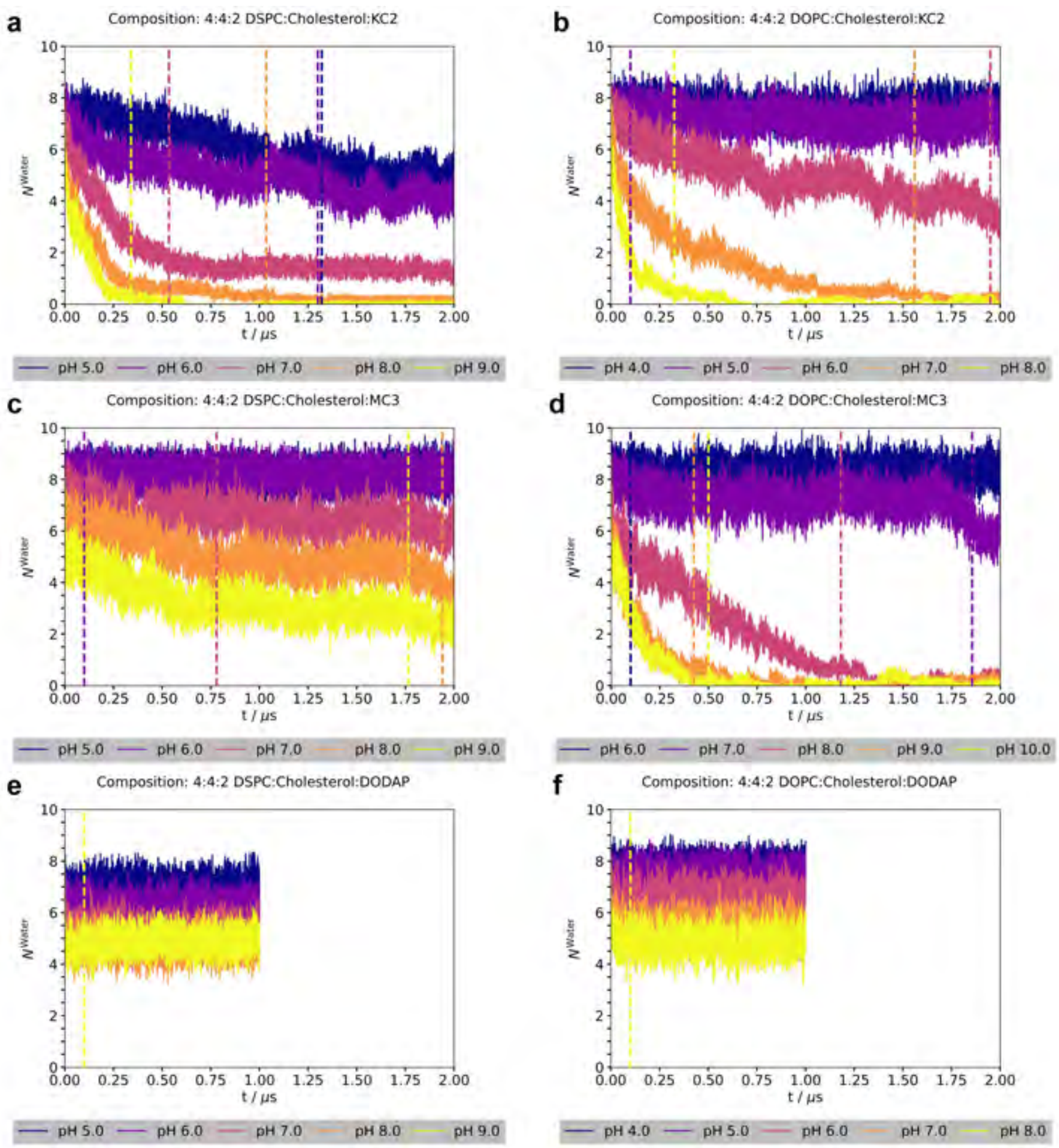

Figure S77: Average number of water molecules within 0.5 nm of the titratable headgroups (ρ_n^{Water}) per frame for membranes with a 4:4:2 helper lipid:cholesterol:aminolipid composition containing either **a,c,e** DSPC or **b,d,f** DOPC as the helper lipid, and **a,b** DLin-KC2-DMA, **c,d** DLin-MC3-DMA or **e,f** DODAP as the aminolipid. Vertical dashed lines indicate the equilibration time determined for each pH value. The reference atom of the aminolipid was the hydrogen atom of the titratable headgroup, whereas the oxygen atom was used as the reference for water.

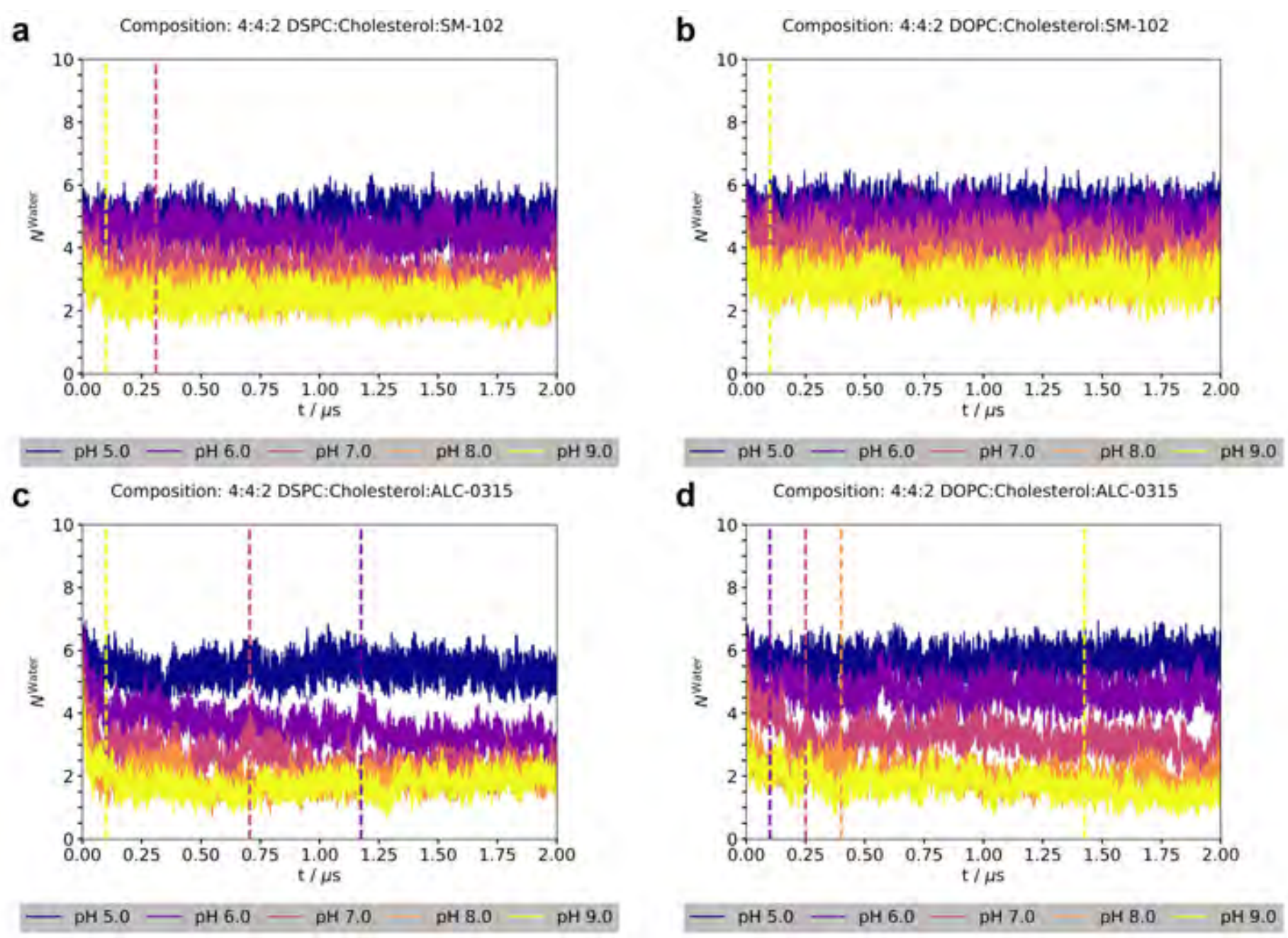

Figure S78: Average number of water molecules within 0.5 nm of the titratable headgroups (ρ_n^{Water}) per frame for membranes with a 4:4:2 helper lipid:cholesterol:aminolipid composition containing either **a,c** DSPC or **b,d** DOPC as the helper lipid, and **a,b** SM-102, or **c,d** ALC-0315 as the aminolipid. Vertical dashed lines indicate the equilibration time determined for each pH value. The reference atom of the aminolipid was the hydrogen atom of the titratable headgroup, whereas the oxygen atom was used as the reference for water.

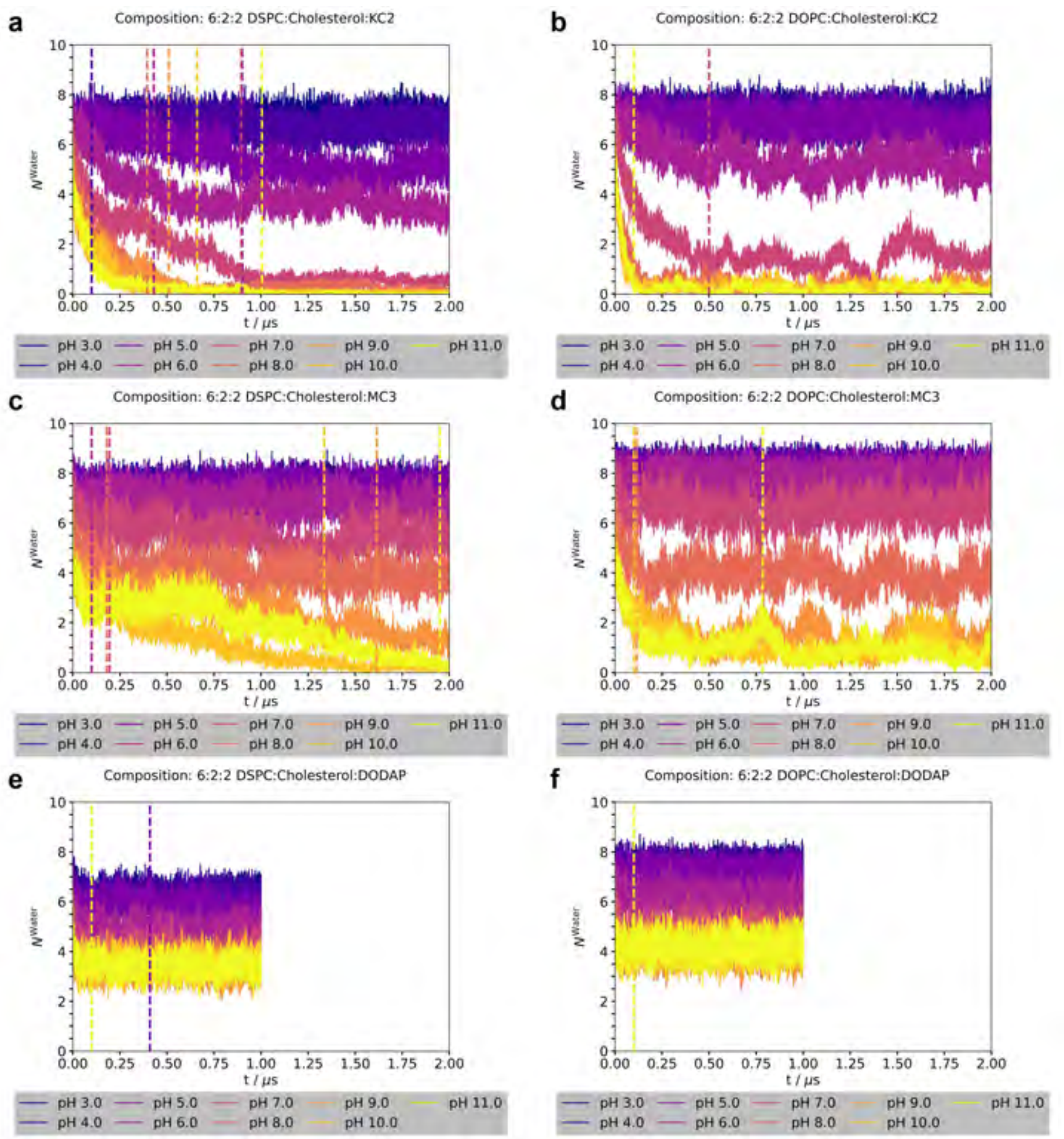

Figure S79: Average number of water molecules within 0.5 nm of the titratable headgroups (ρ_n^{Water}) per frame for membranes with a 6:2:2 helper lipid:cholesterol:aminolipid composition containing either **a,c,e** DSPC or **b,d,f** DOPC as the helper lipid, and **a,b** DLin-KC2-DMA, **c,d** DLin-MC3-DMA or **e,f** DODAP as the aminolipid. Vertical dashed lines indicate the equilibration time determined for each pH value. The reference atom of the aminolipid was the hydrogen atom of the titratable headgroup, whereas the oxygen atom was used as the reference for water.

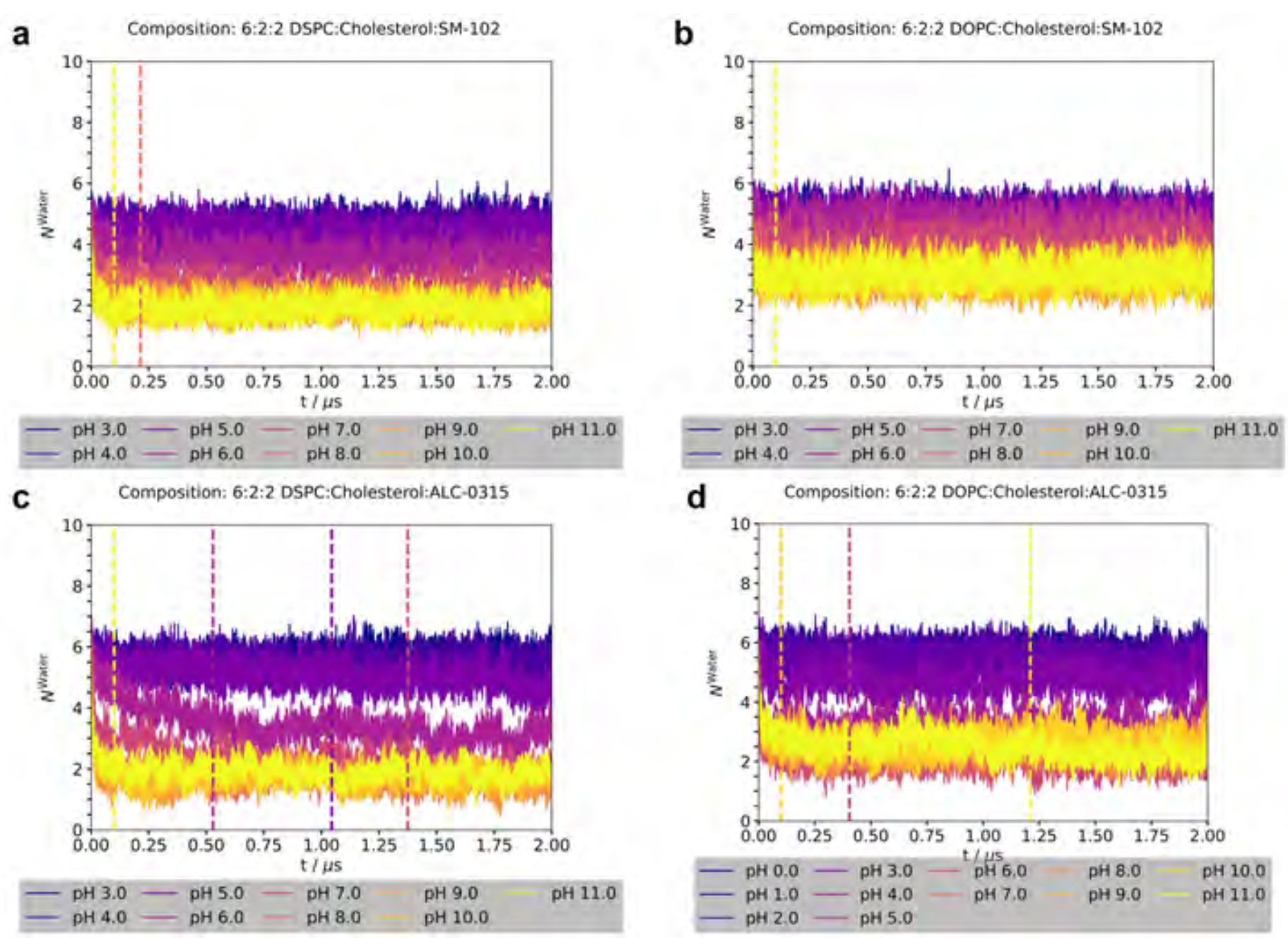

Figure S80: Average number of water molecules within 0.5 nm of the titratable headgroups (ρ_n^{Water}) per frame for membranes with a 6:2:2 helper lipid:cholesterol:aminolipid composition containing either **a,c** DSPC or **b,d** DOPC as the helper lipid, and **a,b** SM-102, or **c,d** ALC-0315 as the aminolipid. Vertical dashed lines indicate the equilibration time determined for each pH value. The reference atom of the aminolipid was the hydrogen atom of the titratable headgroup, whereas the oxygen atom was used as the reference for water.

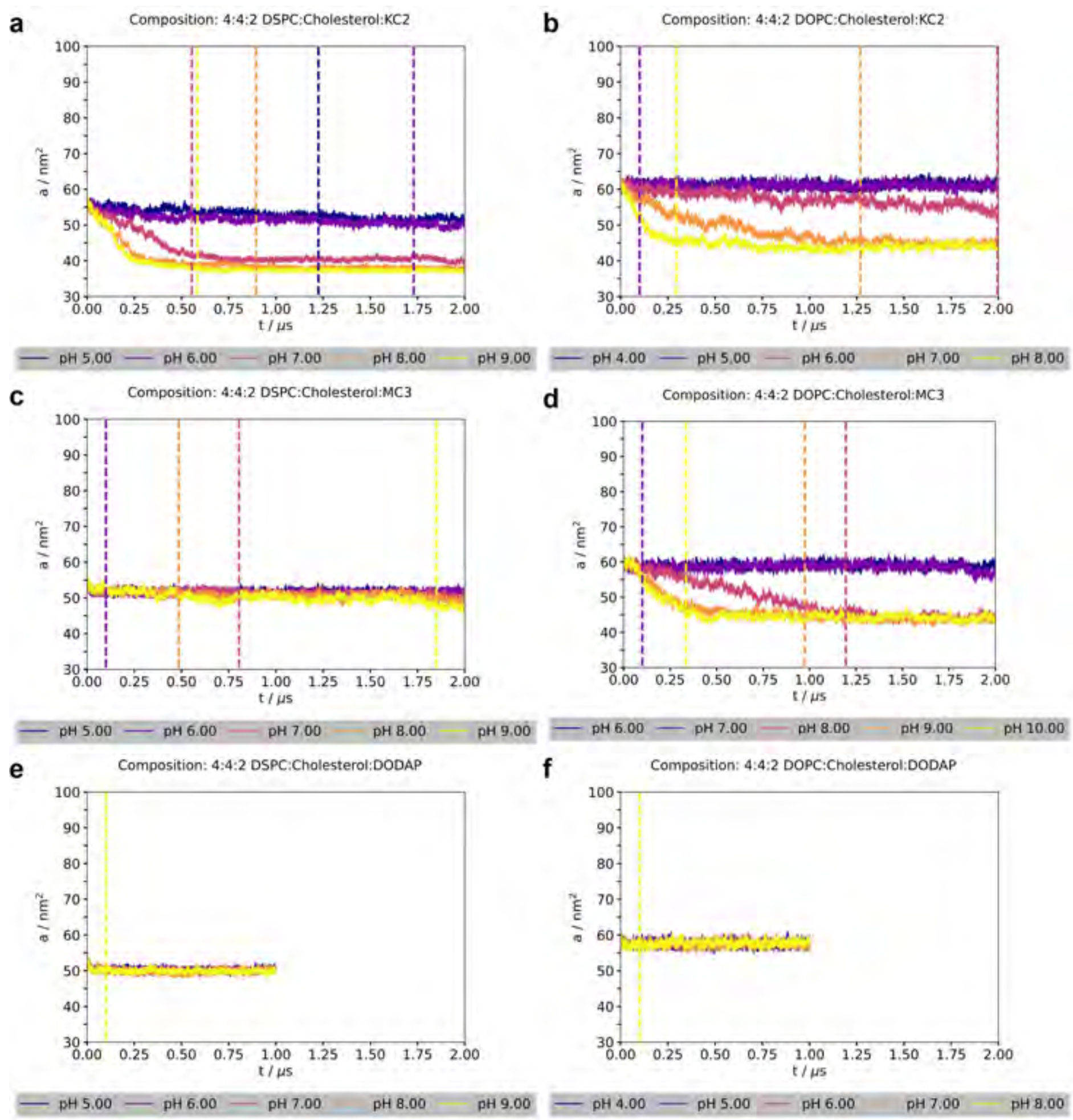

Figure S81: Leaflet surface area per frame for membranes with a 4:4:2 helper lipid:cholesterol:aminolipid composition containing either **a,c,e** DSPC or **b,d,f** DOPC as the helper lipid, and **a,b** DLin-KC2-DMA, **c,d** DLin-MC3-DMA or **e,f** DODAP as the aminolipid. Vertical dashed lines indicate the equilibration time determined for each pH value.

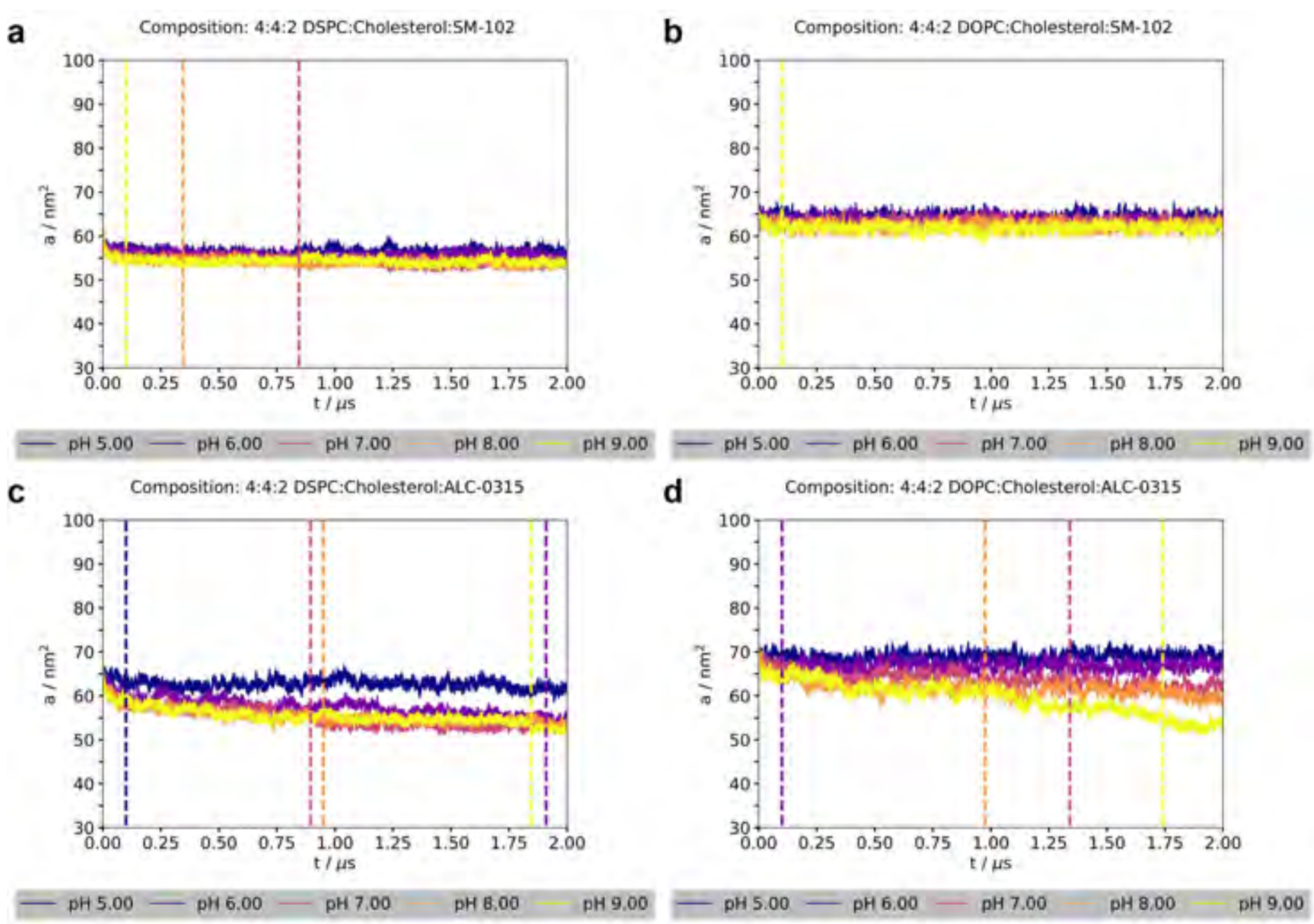

Figure S82: Leaflet surface area per frame for membranes with a 4:4:2 helper lipid:cholesterol:aminolipid composition containing either **a,c** DSPC or **b,d** DOPC as the helper lipid, and **a,b** SM-102, or **c,d** ALC-0315 as the aminolipid. Vertical dashed lines indicate the equilibration time determined for each pH value.

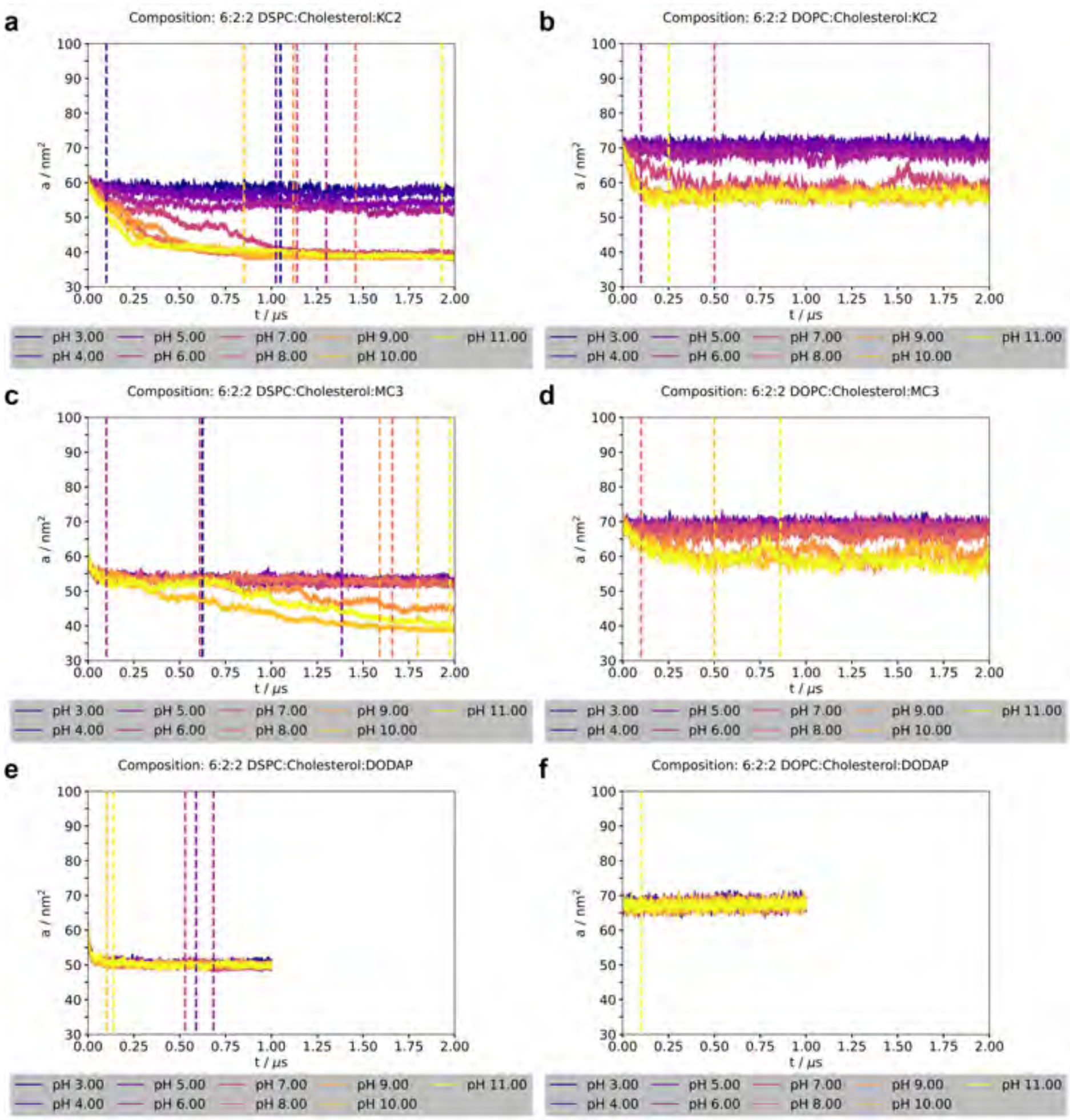

Figure S83: Leaflet surface area per frame for membranes with a 6:2:2 helper lipid:cholesterol:aminolipid composition containing either **a,c,e** DSPC or **b,d,f** DOPC as the helper lipid, and **a,b** DLin-KC2-DMA, **c,d** DLin-MC3-DMA or **e,f** DODAP as the aminolipid. Vertical dashed lines indicate the equilibration time determined for each pH value.

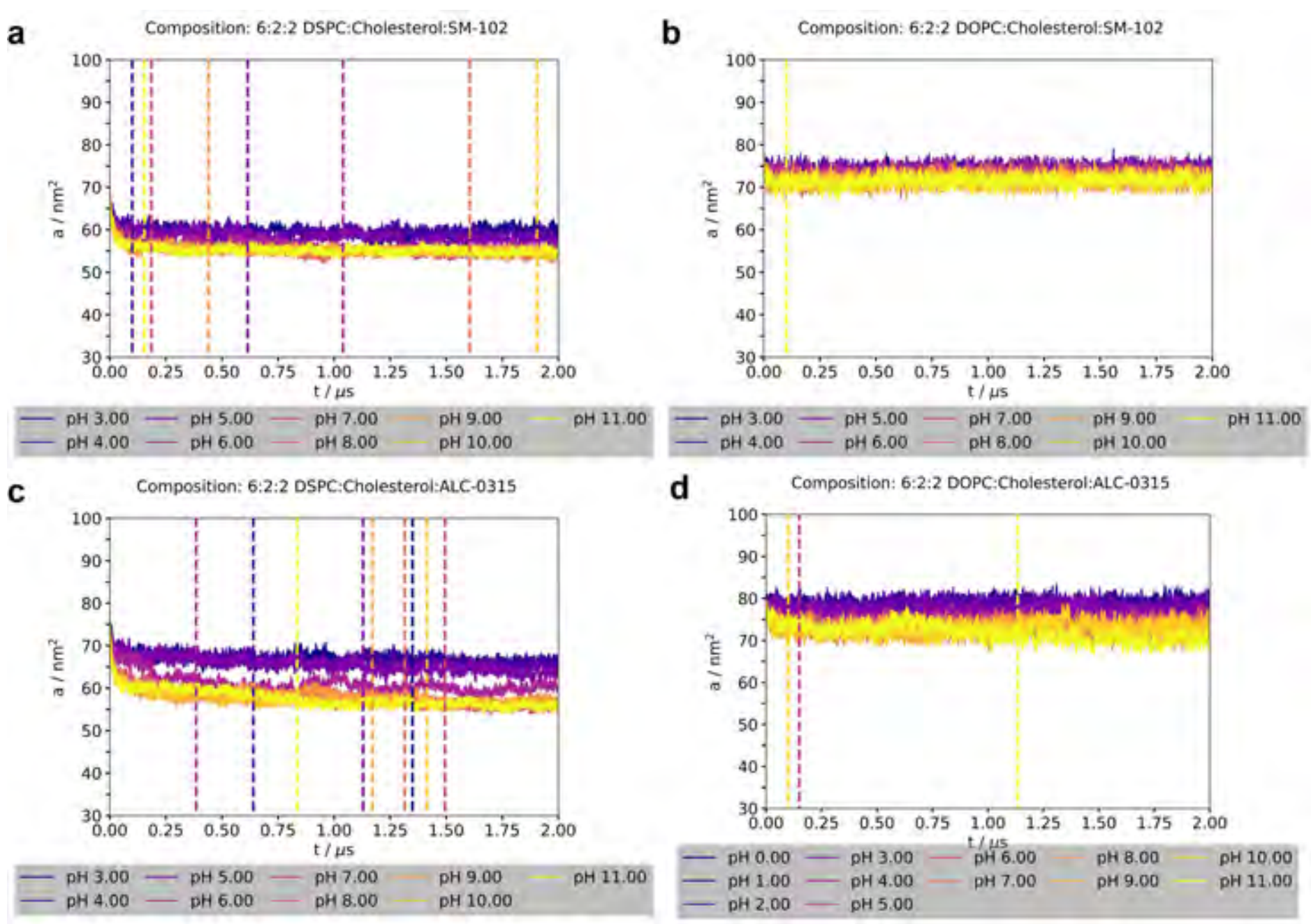

Figure S84: Leaflet surface area per frame for membranes with a 6:2:2 helper lipid:cholesterol:aminolipid composition containing either **a,c** DSPC or **b,d** DOPC as the helper lipid, and **a,b** SM-102, or **c,d** ALC-0315 as the aminolipid. Vertical dashed lines indicate the equilibration time determined for each pH value.

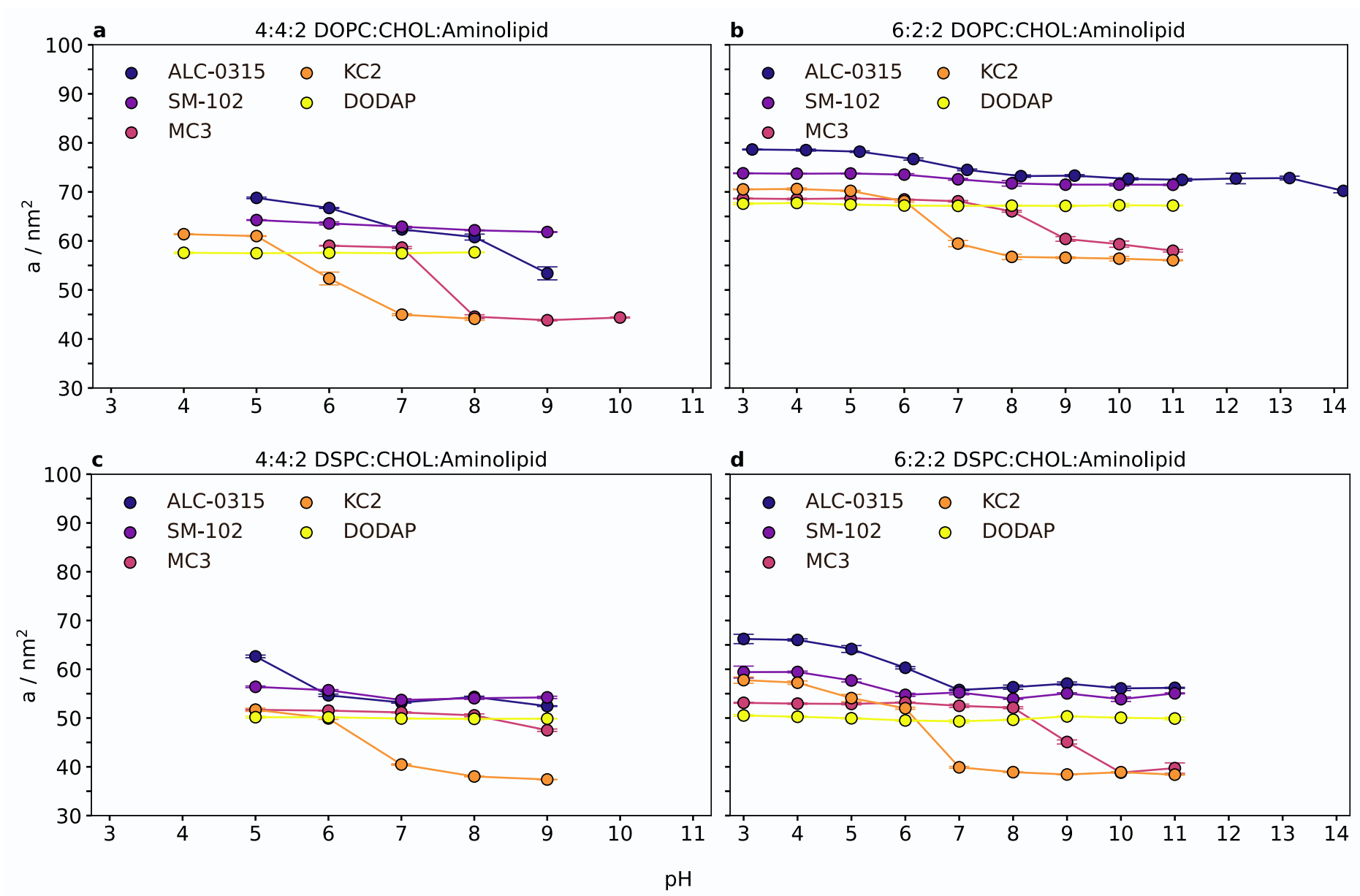

Figure S85: Average leaflet surface area as a function of pH. Mean values of the surface area are shown for DOPC-based (top) and DSPC-based (bottom) membranes containing **a,c** 40 mol% cholesterol and **b,d** 20 mol% cholesterol. Error bars represent the standard error of the mean obtained by block averaging. Abbreviations used: CHOL = cholesterol.

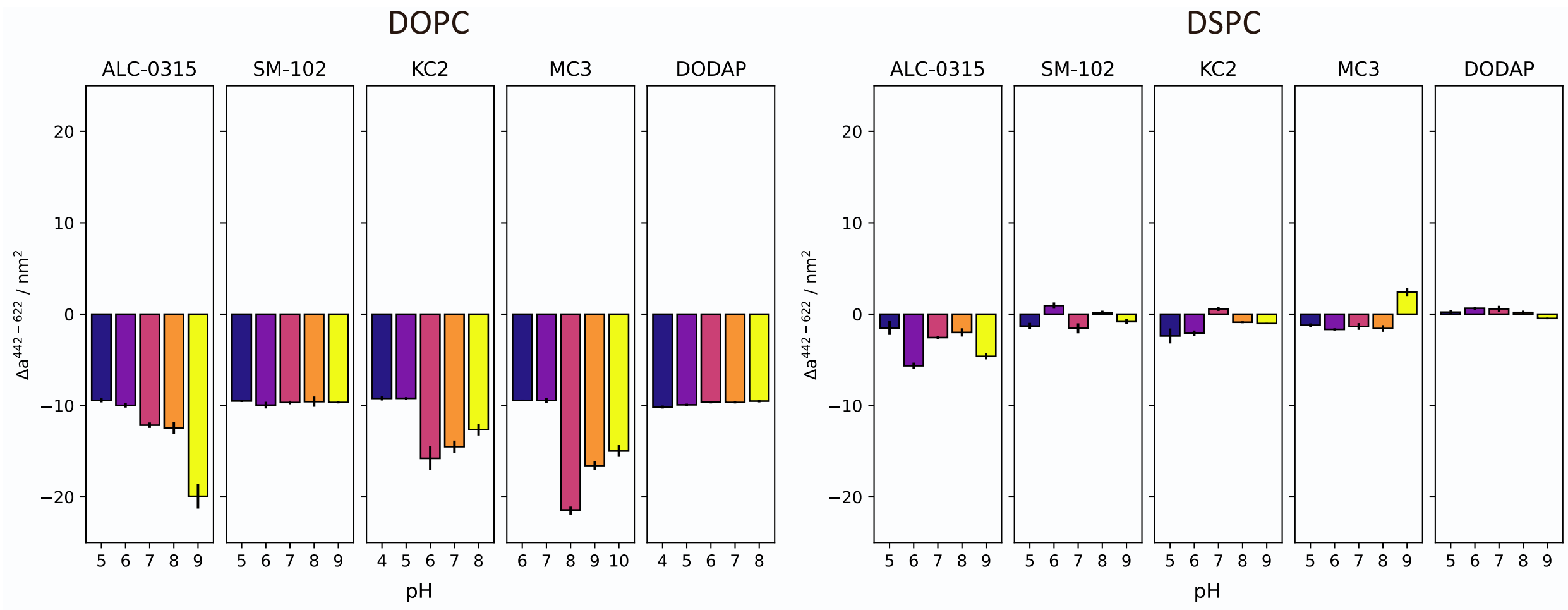

Figure S86: Difference in average leaflet surface area by increasing the cholesterol concentration from 20 mol% to 40 mol% ($\Delta a = a^{442} - a^{622}$) as a function of pH. Negative values refer to a smaller average membrane surface area per leaflet for 40 mol% cholesterol. Left and right panels correspond to DOPC and DSPC membranes, respectively. Error bars represent the standard error of the mean obtained by error propagation, assuming independent estimates.

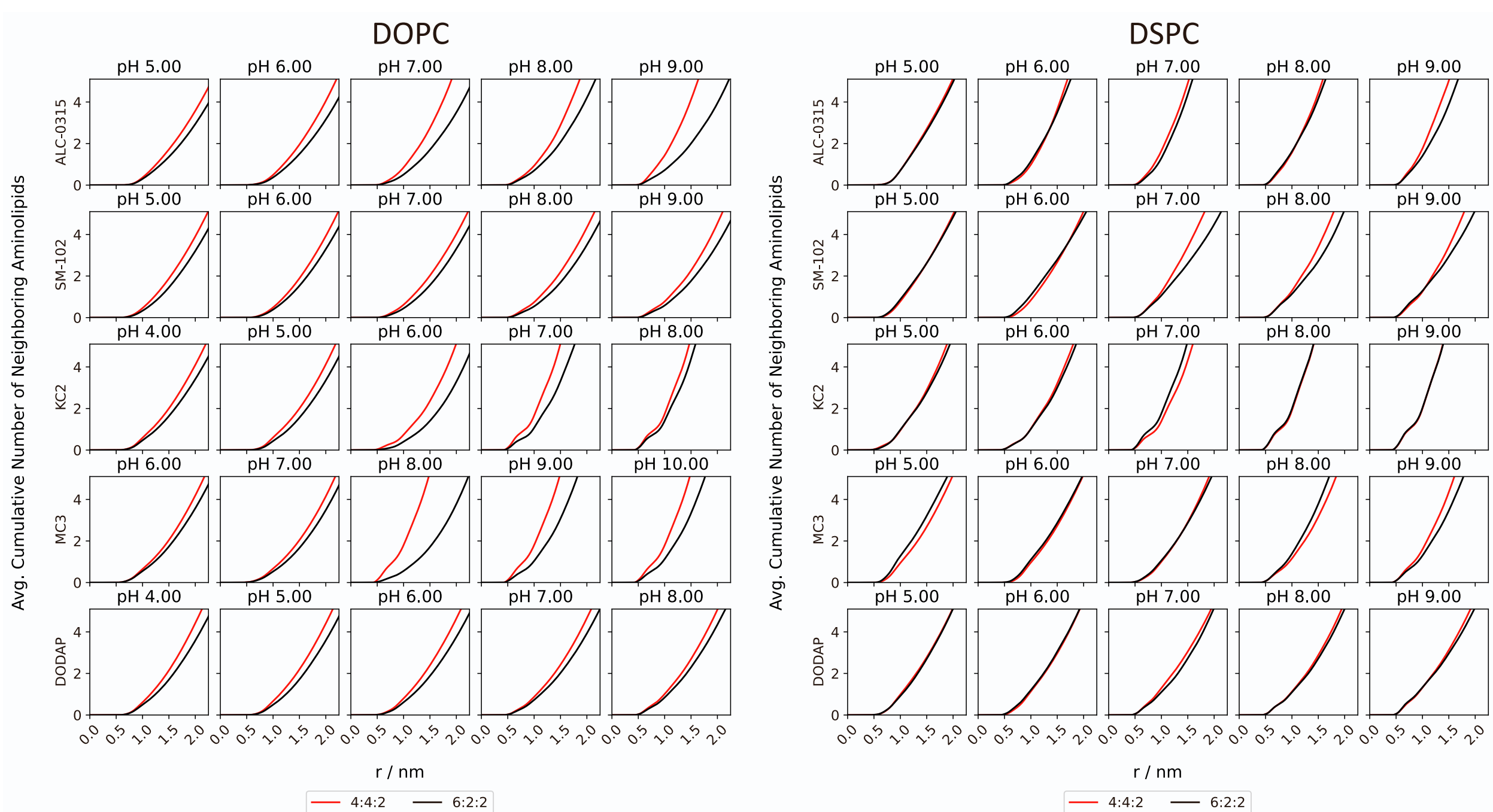

Figure S87: Average cumulative number of aminolipids surrounding other aminolipids as a function of radial distance. The titratable nitrogen atom of each aminolipid was used as the reference point for the distance calculation. Left and right panels correspond to DOPC and DSPC membranes, respectively. Lines correspond either to 20 mol% (black) or 40 mol% (red) cholesterol.

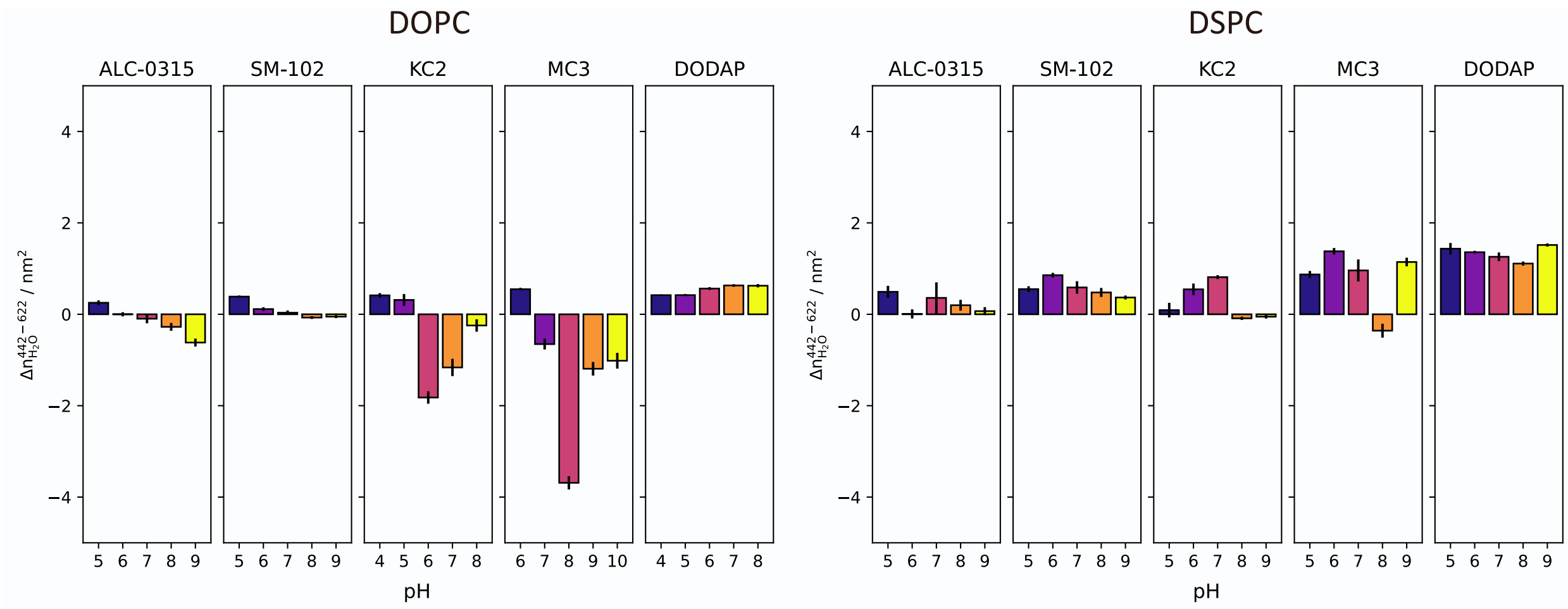

Figure S88: Difference in the average number of water molecules within 0.5 nm of the aminolipid titratable site by increasing the cholesterol concentration from 20 mol% to 40 mol% ($\Delta n_{\mathrm{H_2O}} = a^{442}_{\mathrm{H_2O}} - a^{622}_{\mathrm{H_2O}}$) as a function of pH. Negative values refer to a smaller average number of water molecules for 40 mol% cholesterol. Left and right panels correspond to DOPC and DSPC membranes, respectively. Error bars represent the standard error of the mean obtained by error propagation, assuming independent estimates.

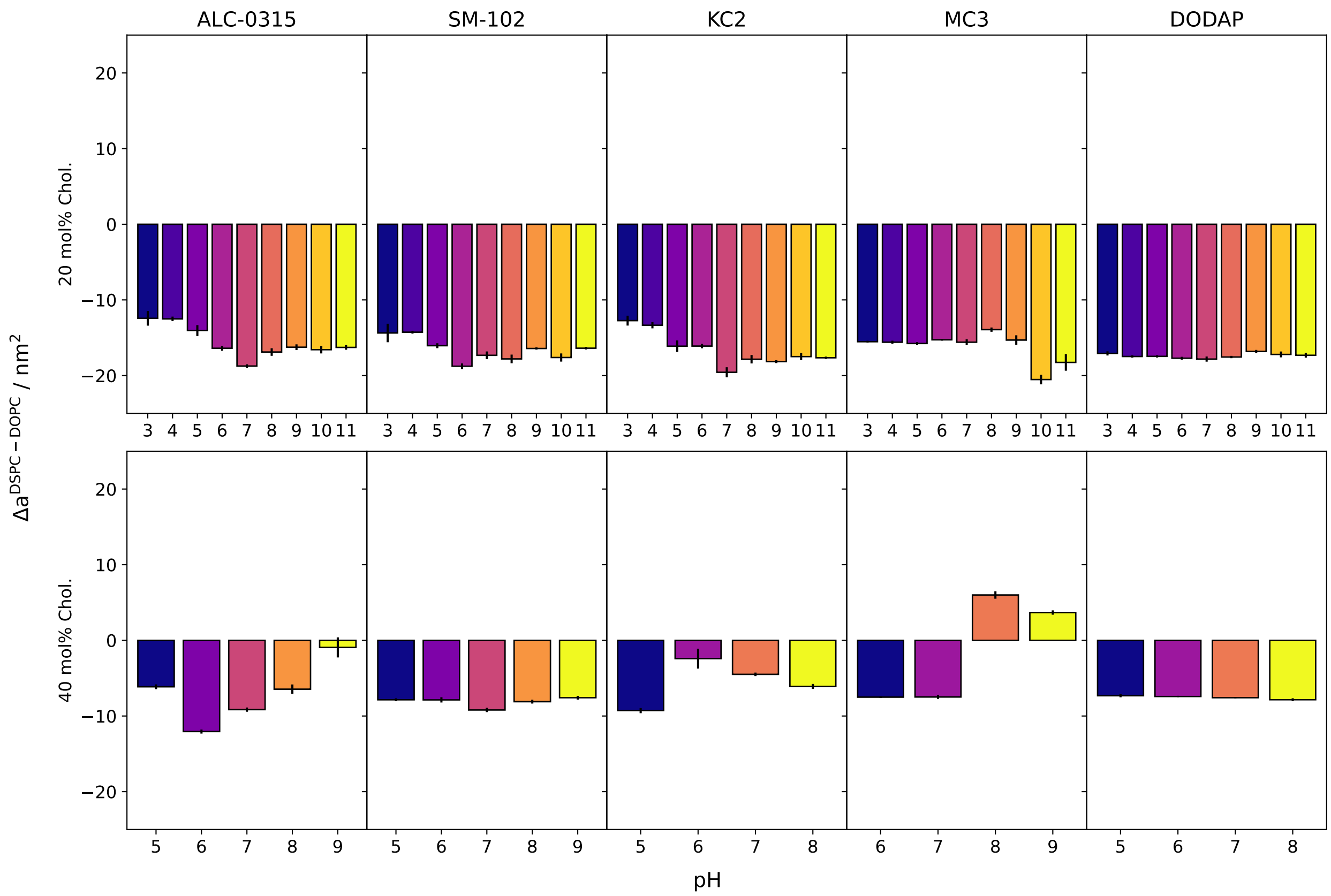

Figure S89: Difference in average leaflet surface area by exchanging DOPC with DSPC as phospholipid in the investigated membranes ($\Delta a = a^{DSPC} - a^{DOPC}$) as function of pH. Negative values refer to a smaller average membrane surface area per leaflet for compositions with DSPC. Upper and lower panel correspond to 20 mol% and 40 mol% cholesterol, respectively. Error bars represent the standard error of the mean obtained by error propagation, assuming independent estimates.

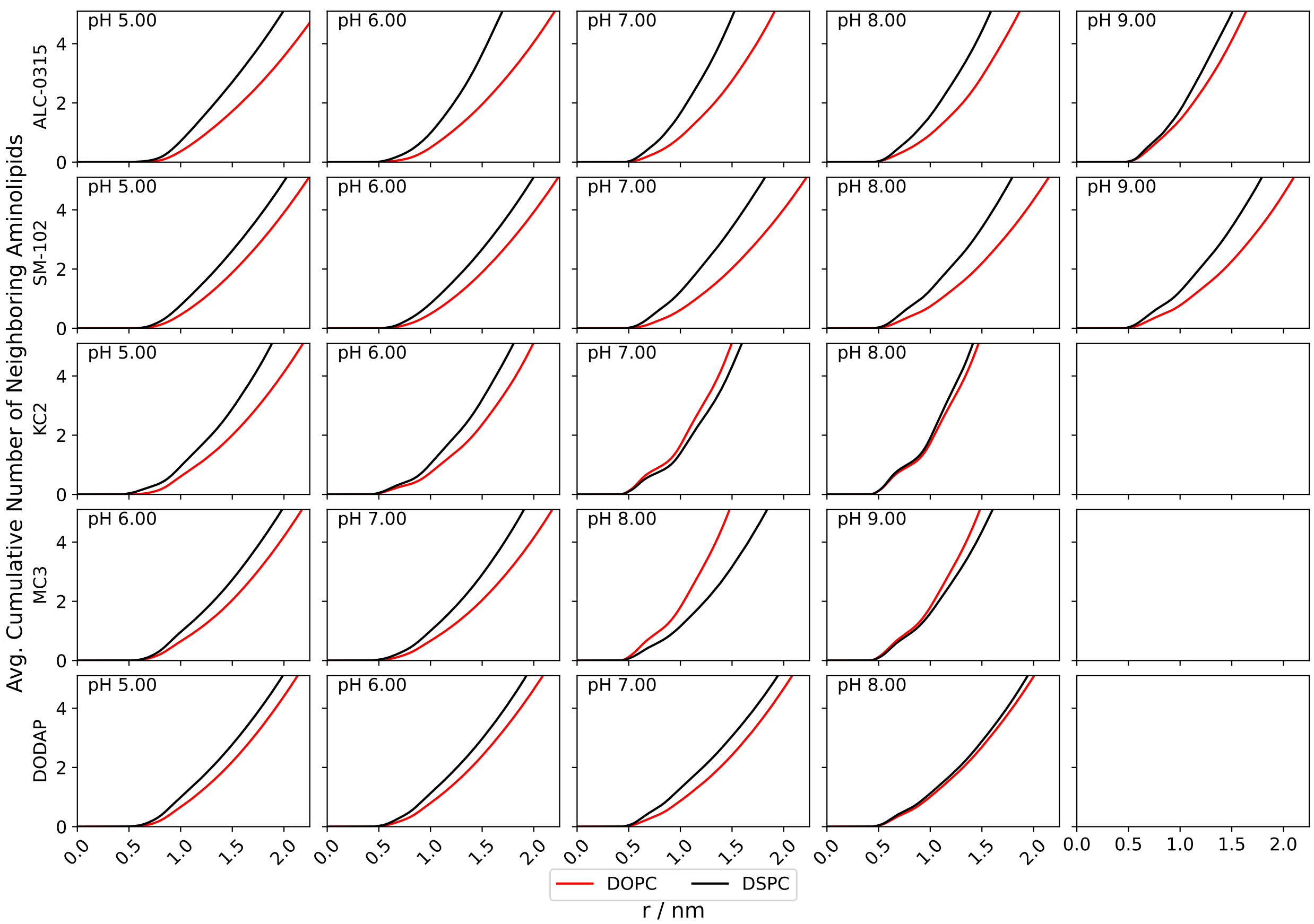

Figure S90: Average cumulative number of neighboring aminolipids as a function of radial distance at a cholesterol concentration of 40 mol%. The titratable nitrogen atom of each aminolipid was used as the reference point for the distance calculation.

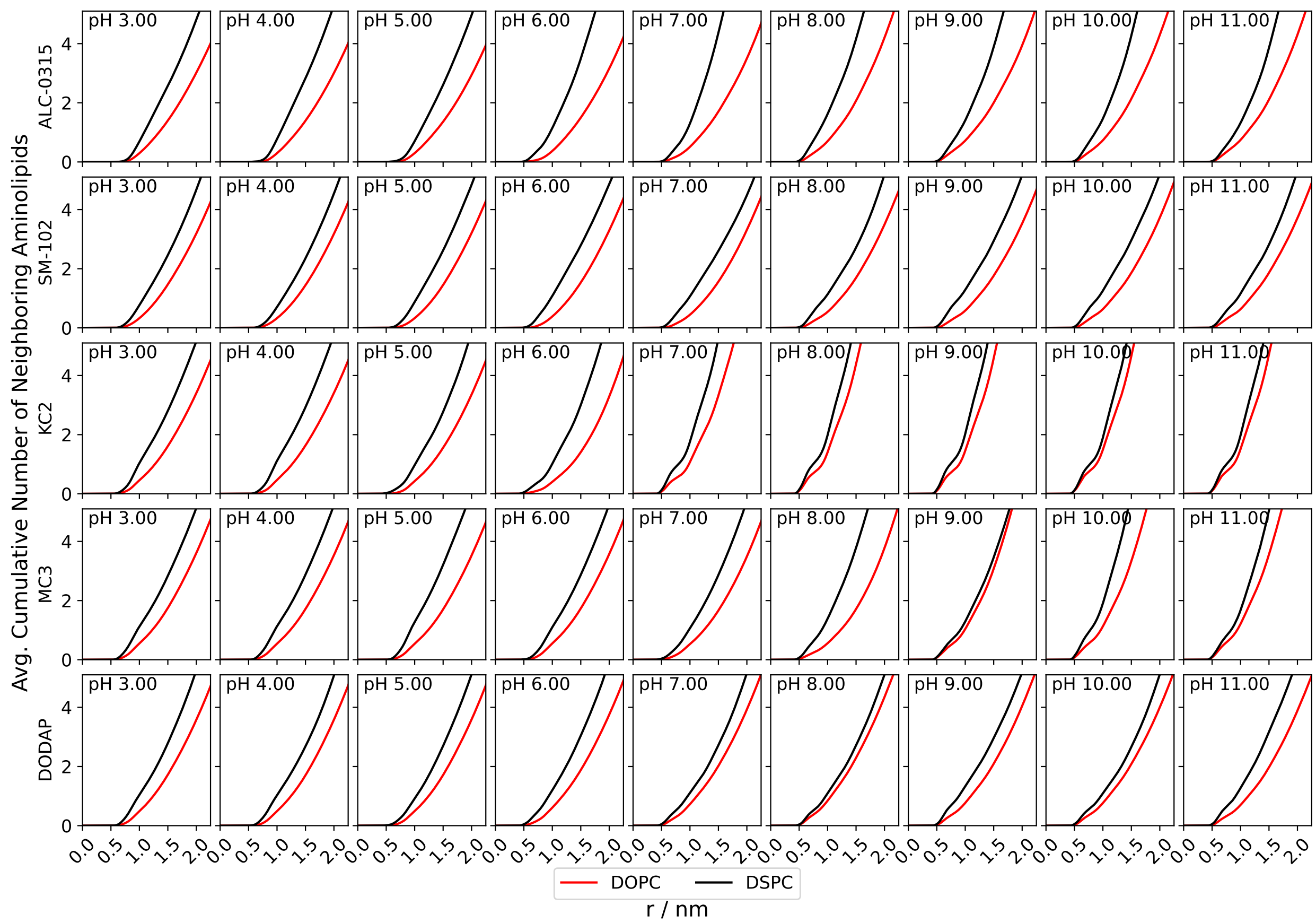

Figure S91: Average cumulative number of aminolipids surrounding other aminolipids as a function of radial distance at a cholesterol concentration of 20 mol%. The titratable nitrogen atom of each aminolipid was used as the reference point for the distance calculation.

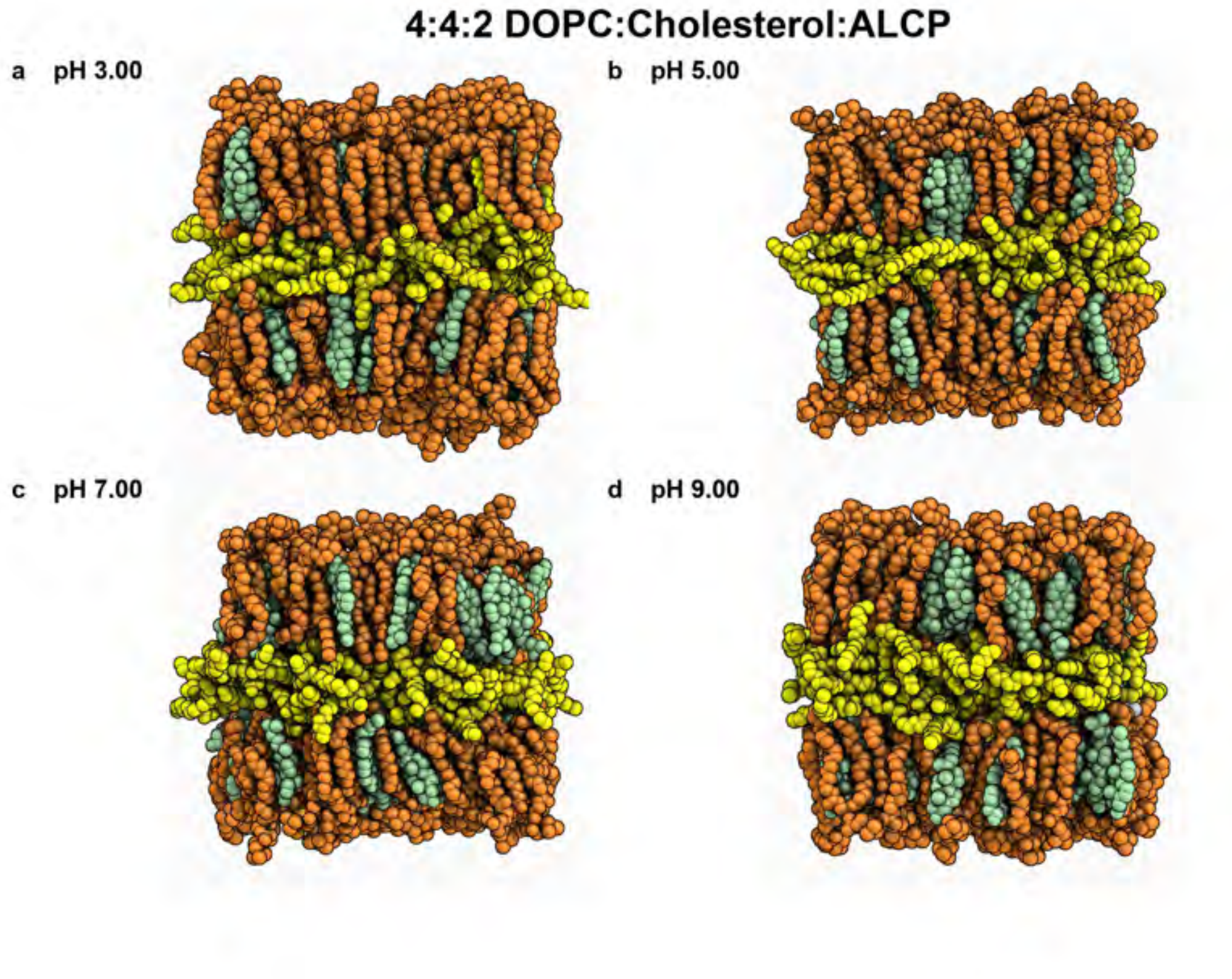

Figure S92: Side-view snapshots of the membrane in the final frame of the simulation at the corresponding pH values. Simulations were setup by placing deprotonated aminolipids in the membrane core. The helper lipid is shown in orange, cholesterol in green, the protonated amnolipid (net charge + 1) in blue, the deprotonated aminolipid (net charge 0) in yellow, and the aminolipids without a specific state ($0.2 \leq \lambda \leq 0.8$) in grey.

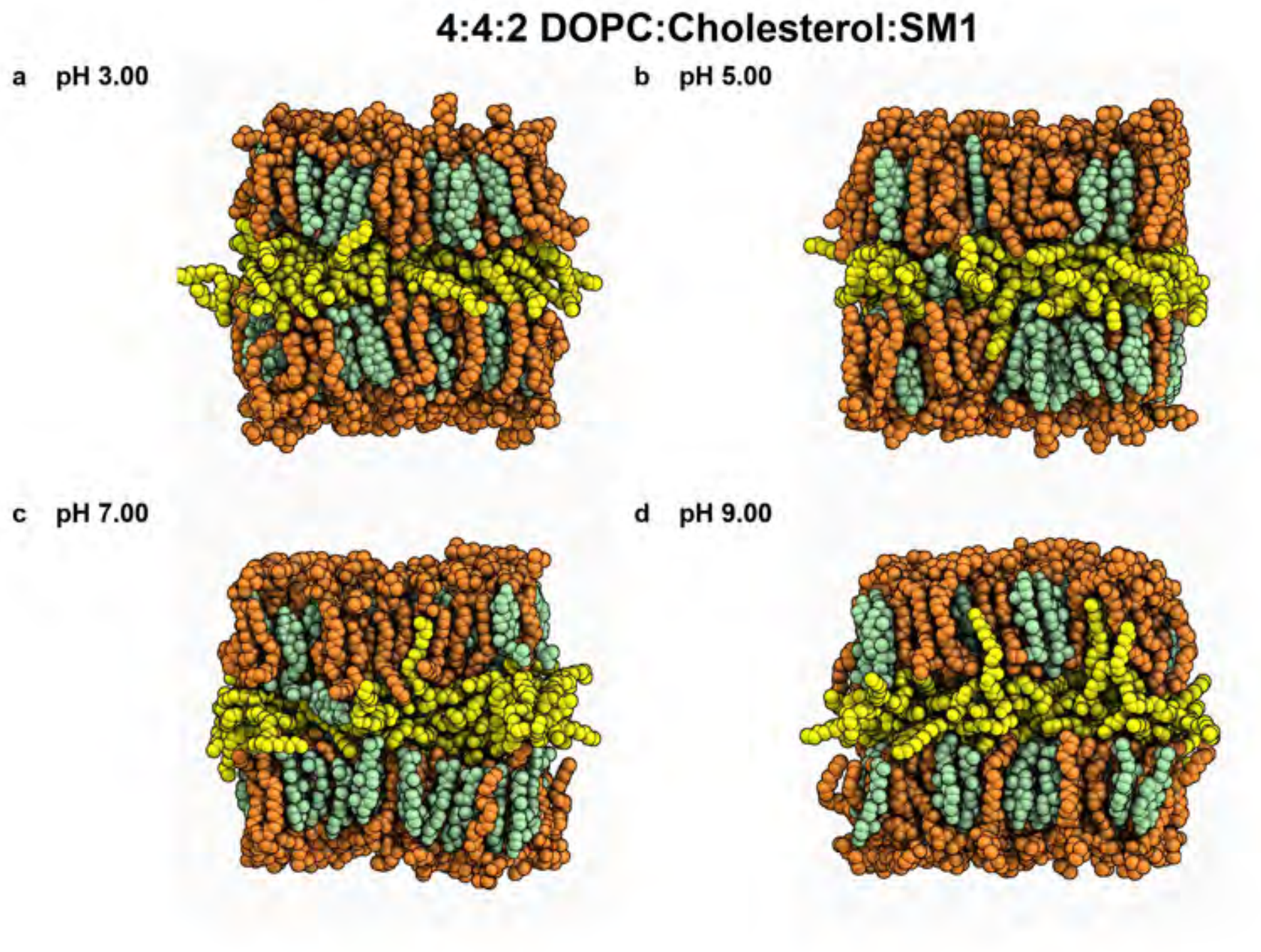

Figure S93: Side-view snapshots of the membrane in the final frame of the simulation at the corresponding pH values. Simulations were setup by placing deprotonated aminolipids in the membrane core. The helper lipid is shown in orange, cholesterol in green, the protonated amnolipid (net charge + 1) in blue, the deprotonated aminolipid (net charge 0) in yellow, and the aminolipids without a specific state ($0.2 \leq \lambda \leq 0.8$) in grey.

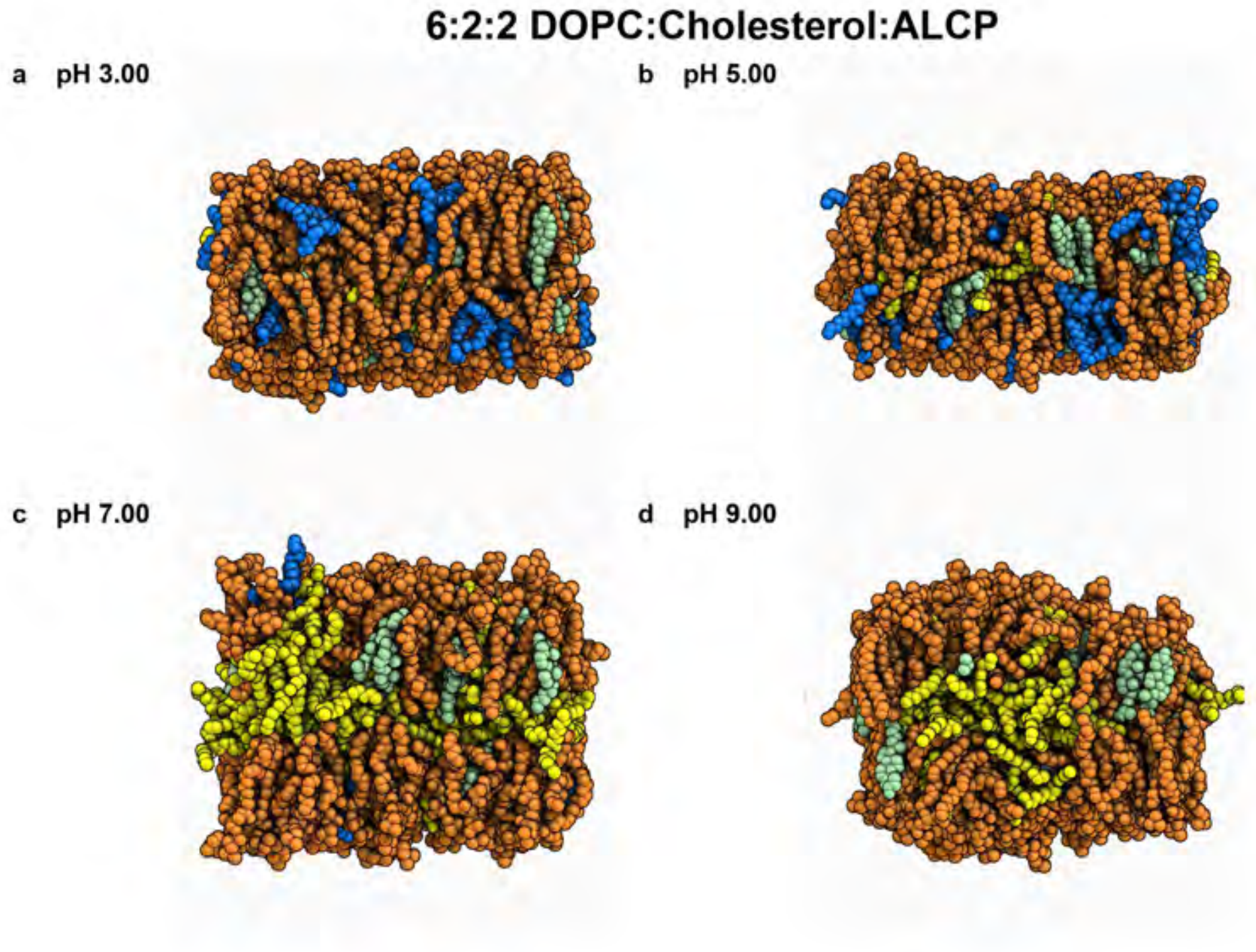

Figure S94: Side-view snapshots of the membrane in the final frame of the simulation at the corresponding pH values. Simulations were setup by placing deprotonated aminolipids in the membrane core. The helper lipid is shown in orange, cholesterol in green, the protonated amnolipid (net charge + 1) in blue, the deprotonated aminolipid (net charge 0) in yellow, and the aminolipids without a specific state ($0.2 \leq \lambda \leq 0.8$) in grey.

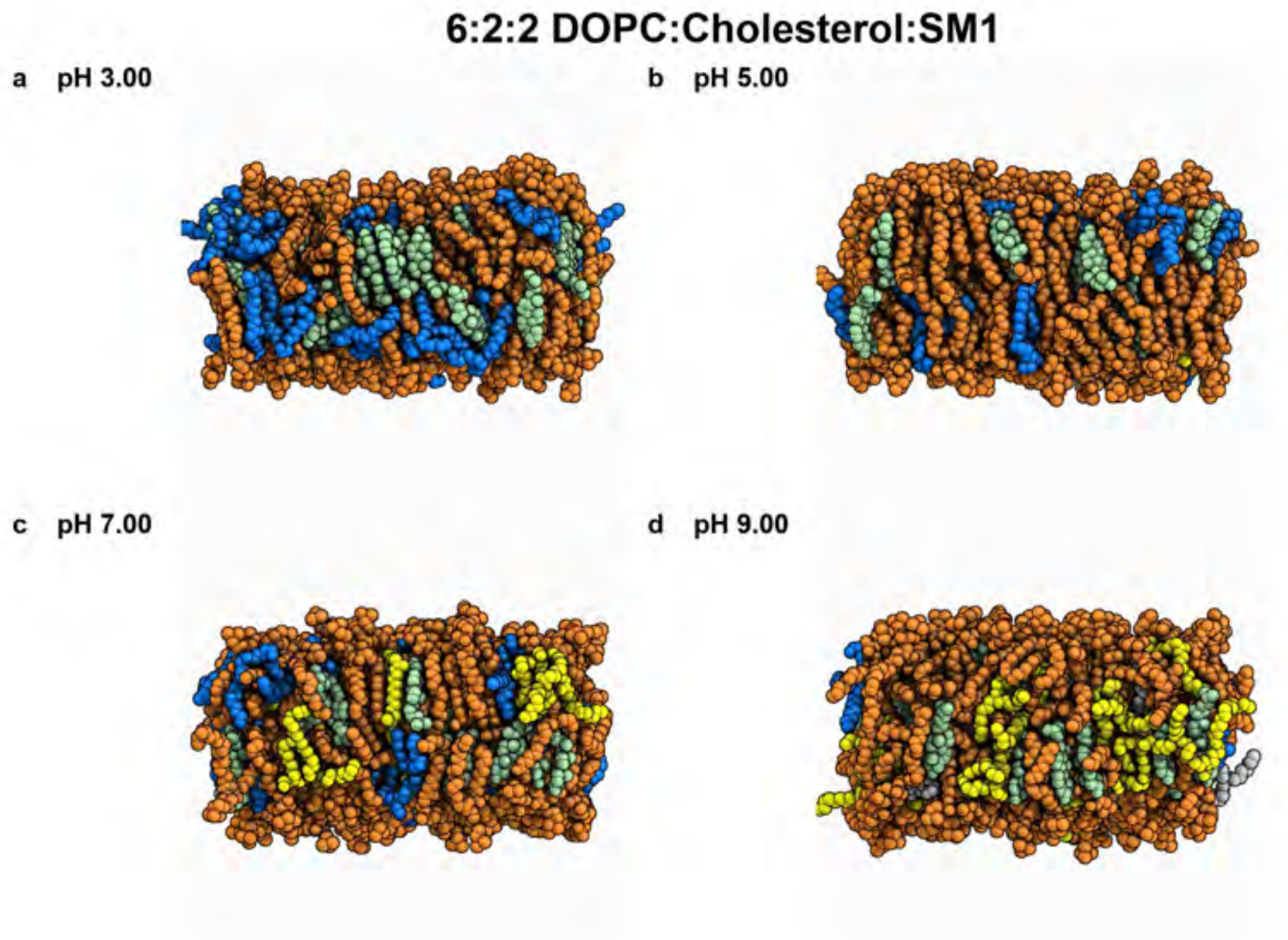

Figure S95: Side-view snapshots of the membrane in the final frame of the simulation at the corresponding pH values. Simulations were setup by placing deprotonated aminolipids in the membrane core. The helper lipid is shown in orange, cholesterol in green, the protonated amnolipid (net charge + 1) in blue, the deprotonated aminolipid (net charge 0) in yellow, and the aminolipids without a specific state ($0.2 \leq \lambda \leq 0.8$) in grey.

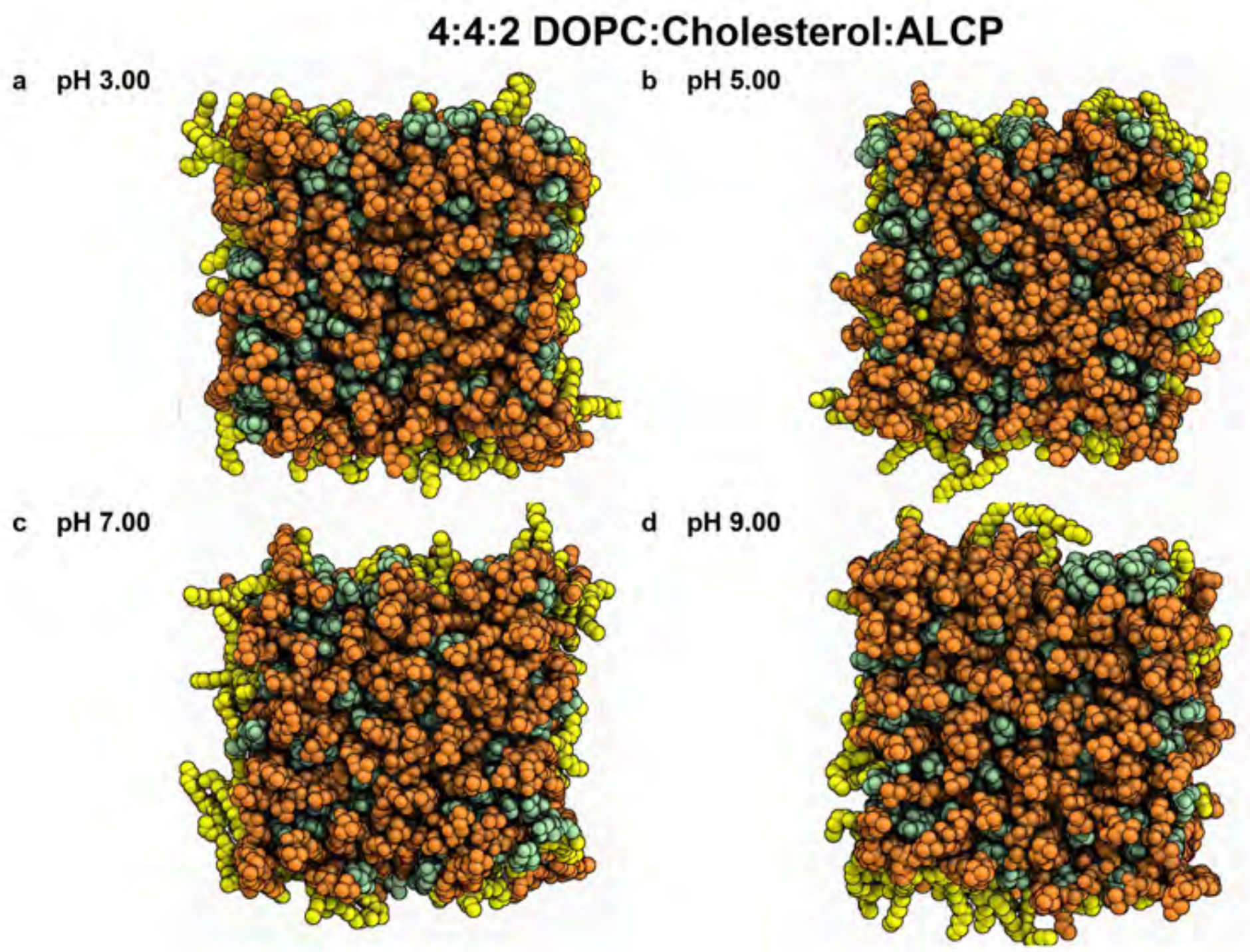

Figure S96: Top-view snapshots of the membrane in the final frame of the simulation at the corresponding pH values. Simulations were setup by placing deprotonated aminolipids in the membrane core. The helper lipid is shown in orange, cholesterol in green, the protonated amnolipid (net charge + 1) in blue, the deprotonated aminolipid (net charge 0) in yellow, and the aminolipids without a specific state ($0.2 \leq \lambda \leq 0.8$) in grey.

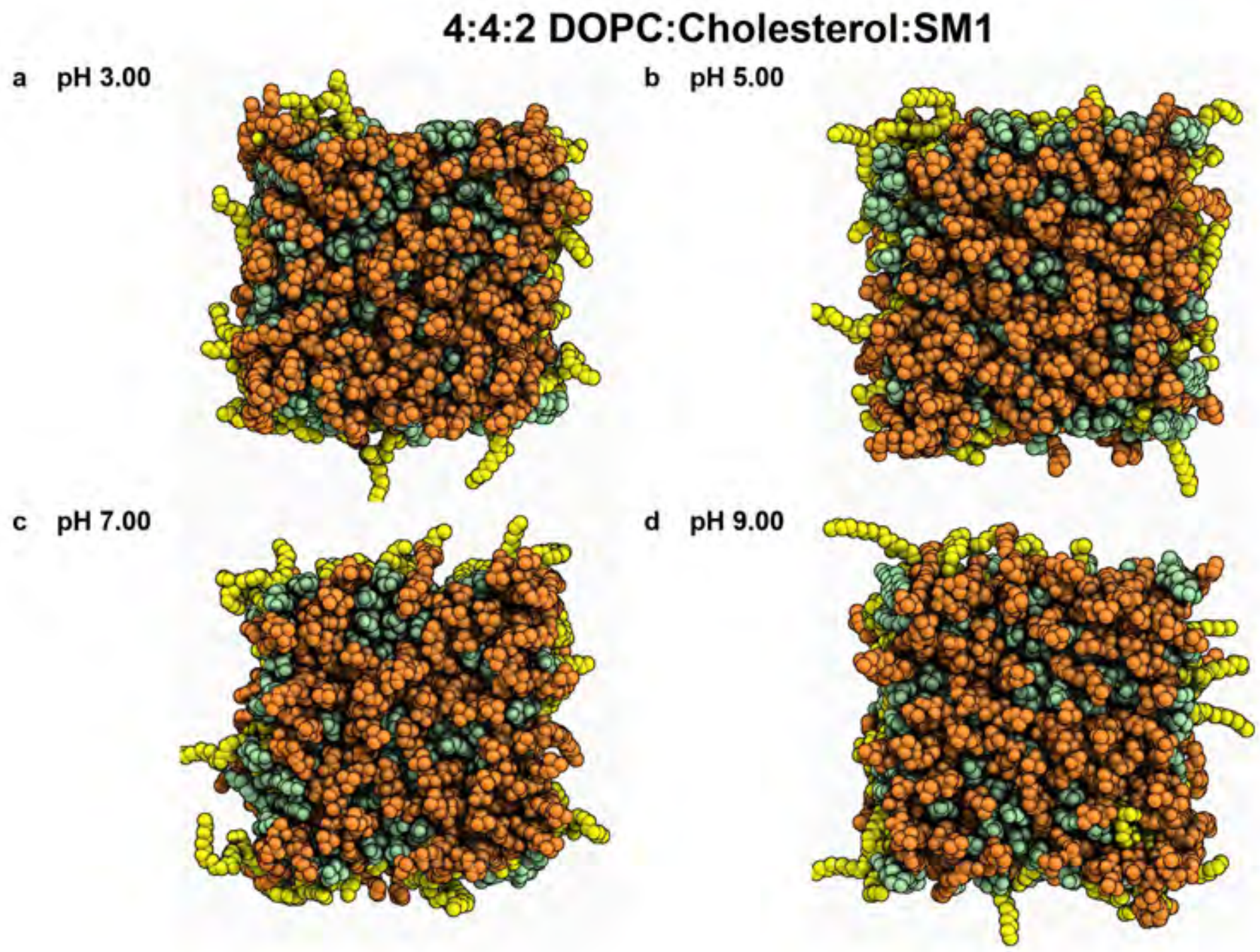

Figure S97: Top-view snapshots of the membrane in the final frame of the simulation at the corresponding pH values. Simulations were setup by placing deprotonated aminolipids in the membrane core. The helper lipid is shown in orange, cholesterol in green, the protonated amnolipid (net charge + 1) in blue, the deprotonated aminolipid (net charge 0) in yellow, and the aminolipids without a specific state ($0.2 \leq \lambda \leq 0.8$) in grey.

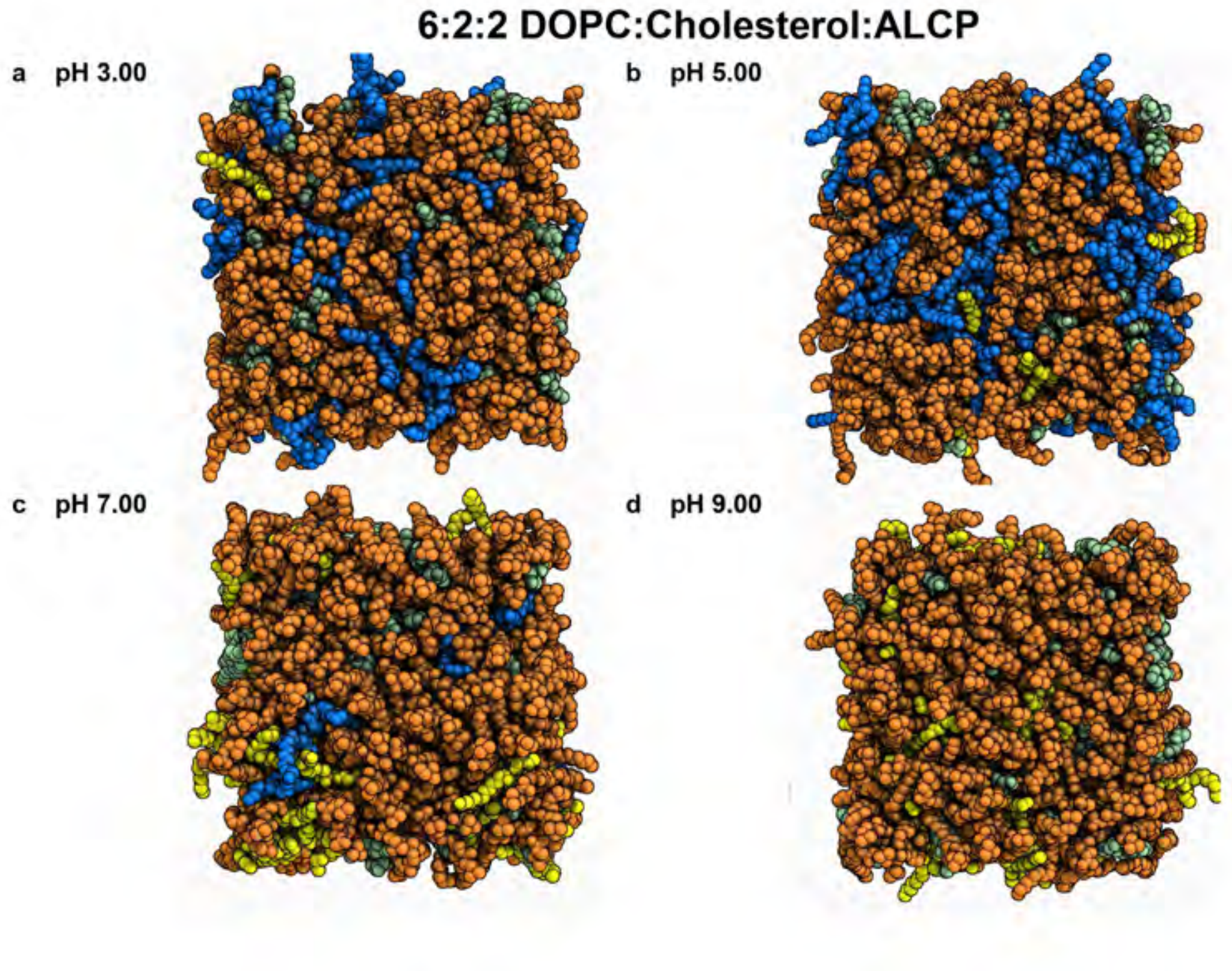

Figure S98: Top-view snapshots of the membrane in the final frame of the simulation at the corresponding pH values. Simulations were setup by placing deprotonated aminolipids in the membrane core. The helper lipid is shown in orange, cholesterol in green, the protonated amnolipid (net charge + 1) in blue, the deprotonated aminolipid (net charge 0) in yellow, and the aminolipids without a specific state ($0.2 \leq \lambda \leq 0.8$) in grey.

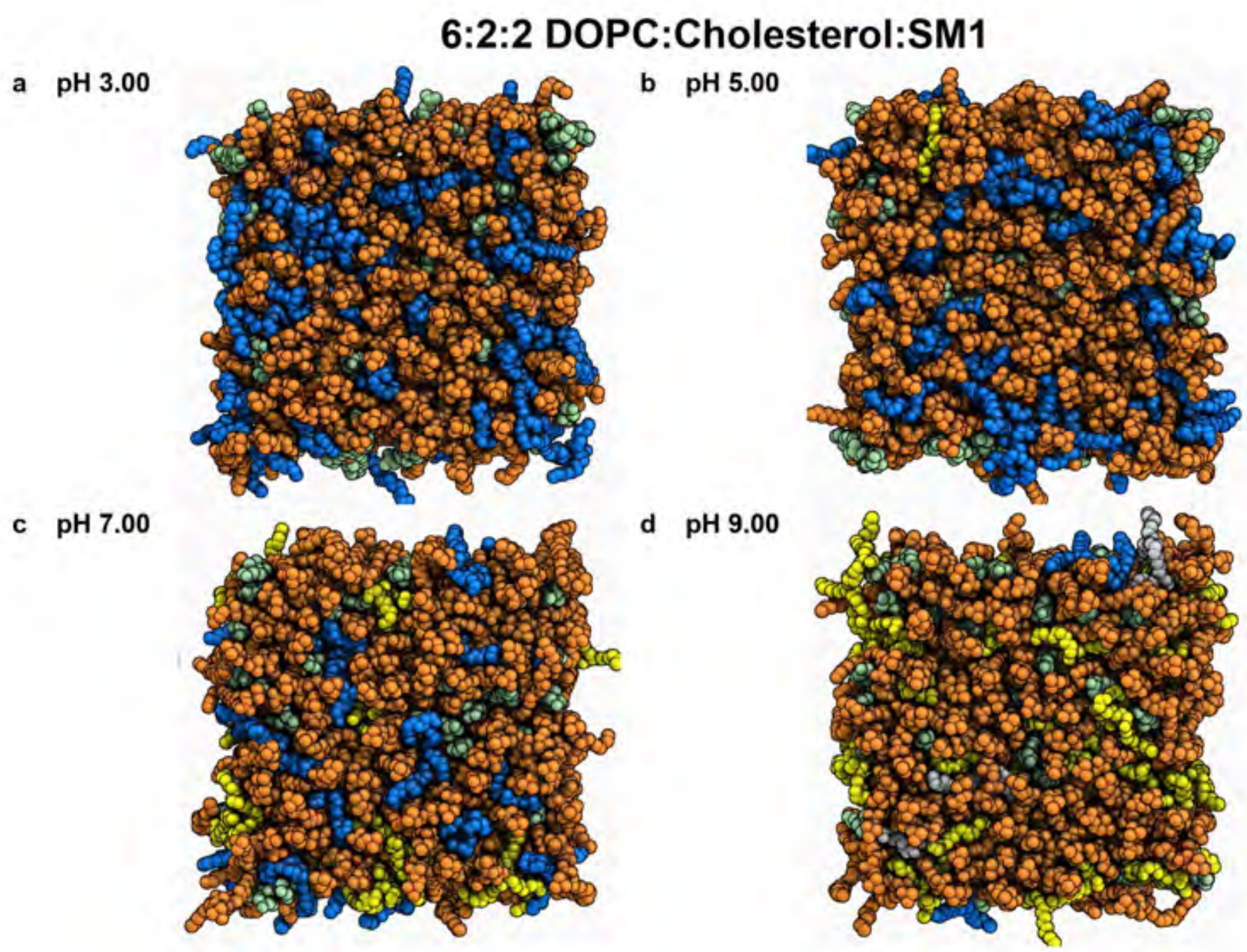

Figure S99: Top-view snapshots of the membrane in the final frame of the simulation at the corresponding pH values. Simulations were setup by placing deprotonated aminolipids in the membrane core. The helper lipid is shown in orange, cholesterol in green, the protonated amnolipid (net charge + 1) in blue, the deprotonated aminolipid (net charge 0) in yellow, and the aminolipids without a specific state ($0.2 \leq \lambda \leq 0.8$) in grey.

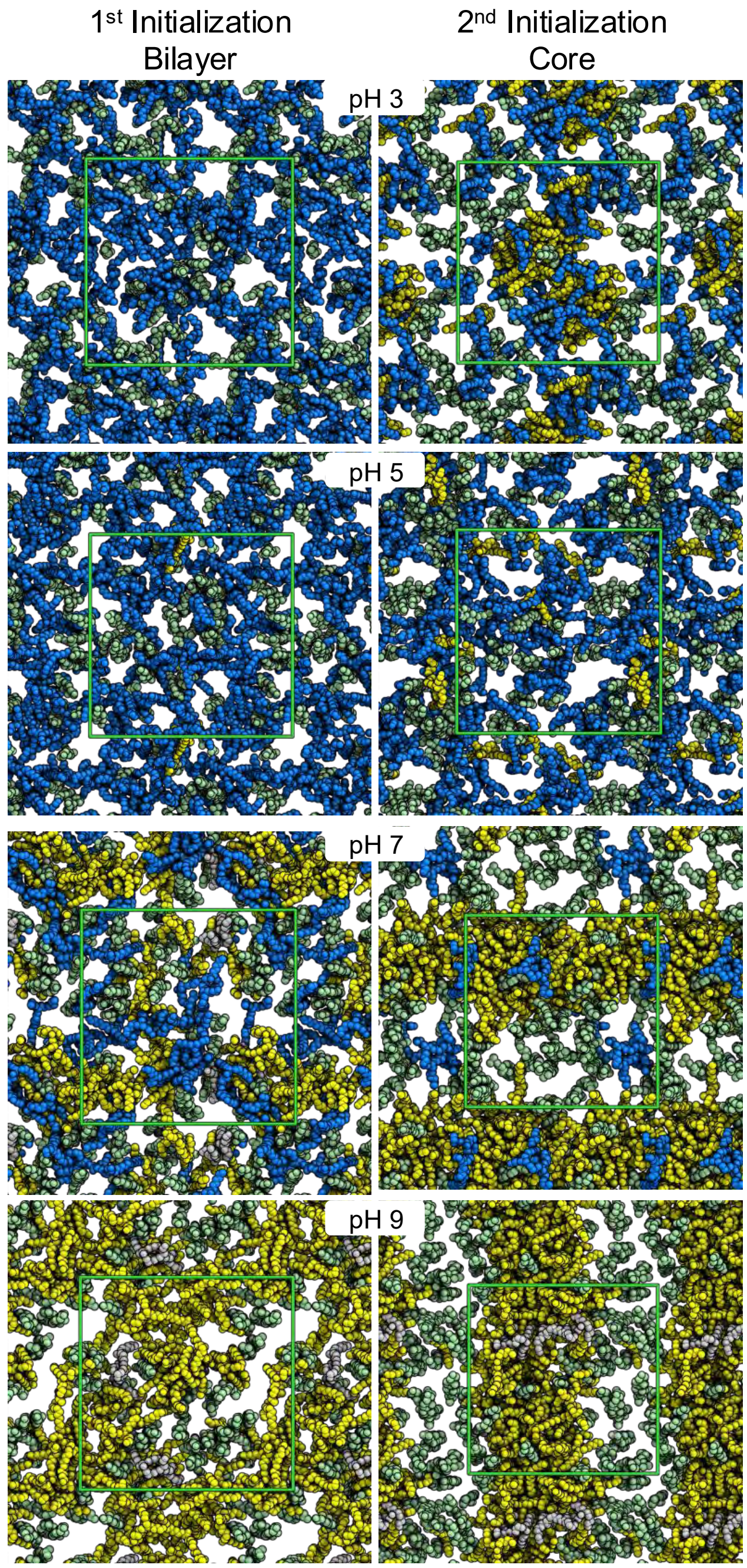

Figure S100: Top-view snapshots of the final simulation frame obtained from the two initialization strategies for ALC-0315 in the 6:2:2 DOPC:cholesterol:aminolipid composition: a bilayer with all aminolipids initially protonated (left) and a surface–core structure with initially deprotonated aminolipids (right), shown at the corresponding pH values in the absence of helper lipids. Cholesterol is shown in green, the protonated amnolipid (net charge + 1) in blue, the deprotonated aminolipid (net charge 0) in yellow, and the aminolipids without a specific state ($0.2 \leq \lambda \leq 0.8$) in grey.

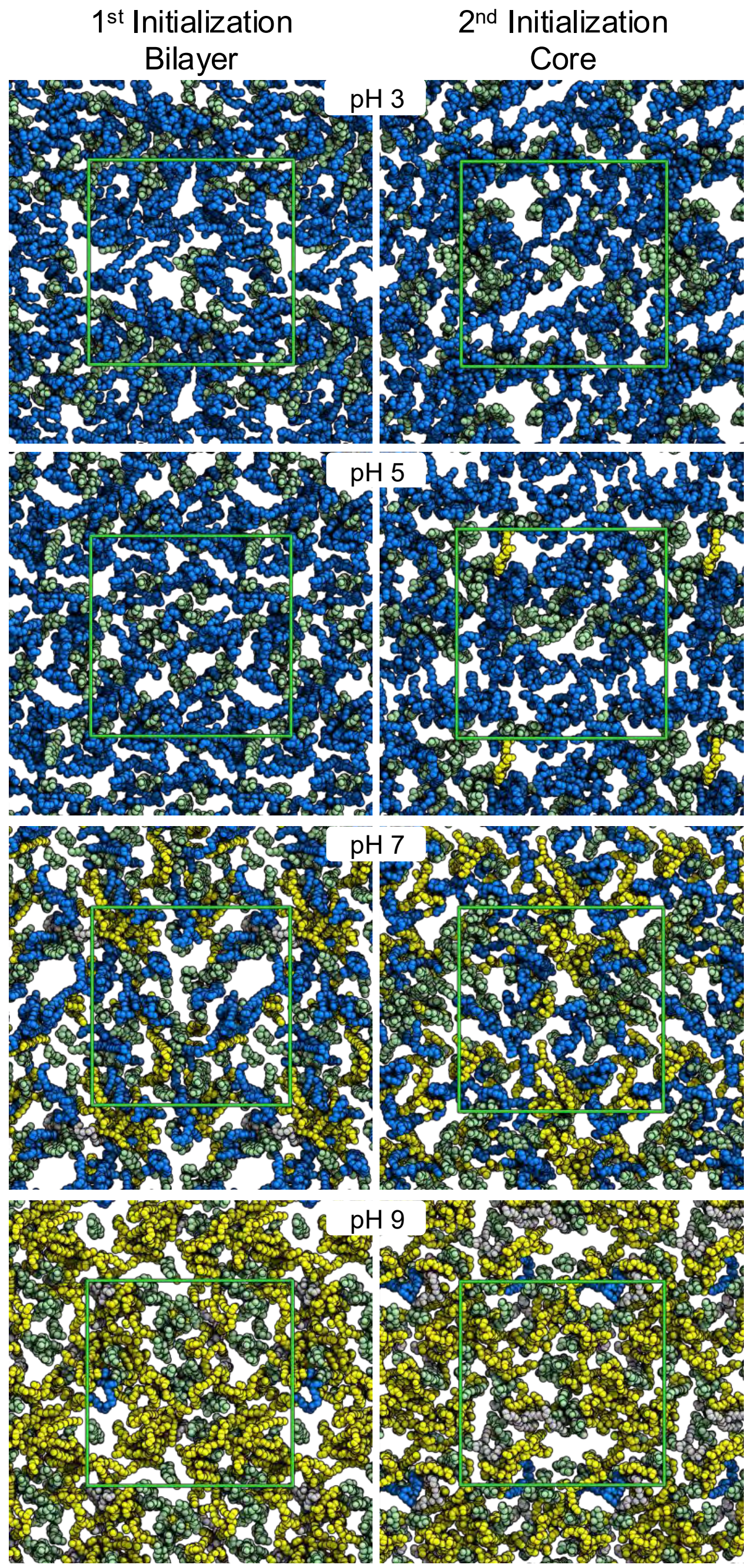

Figure S101: Top-view snapshots of the final simulation frame obtained from the two initialization strategies for SM-102 in the 6:2:2 DOPC:cholesterol:aminolipid composition: a bilayer with all aminolipids initially protonated (left) and a surface–core structure with initially deprotonated aminolipids (right), shown at the corresponding pH values in the absence of helper lipids. Cholesterol is shown in green, the protonated amnolipid (net charge + 1) in blue, the deprotonated aminolipid (net charge 0) in yellow, and the aminolipids without a specific state ($0.2 \leq \lambda \leq 0.8$) in grey.

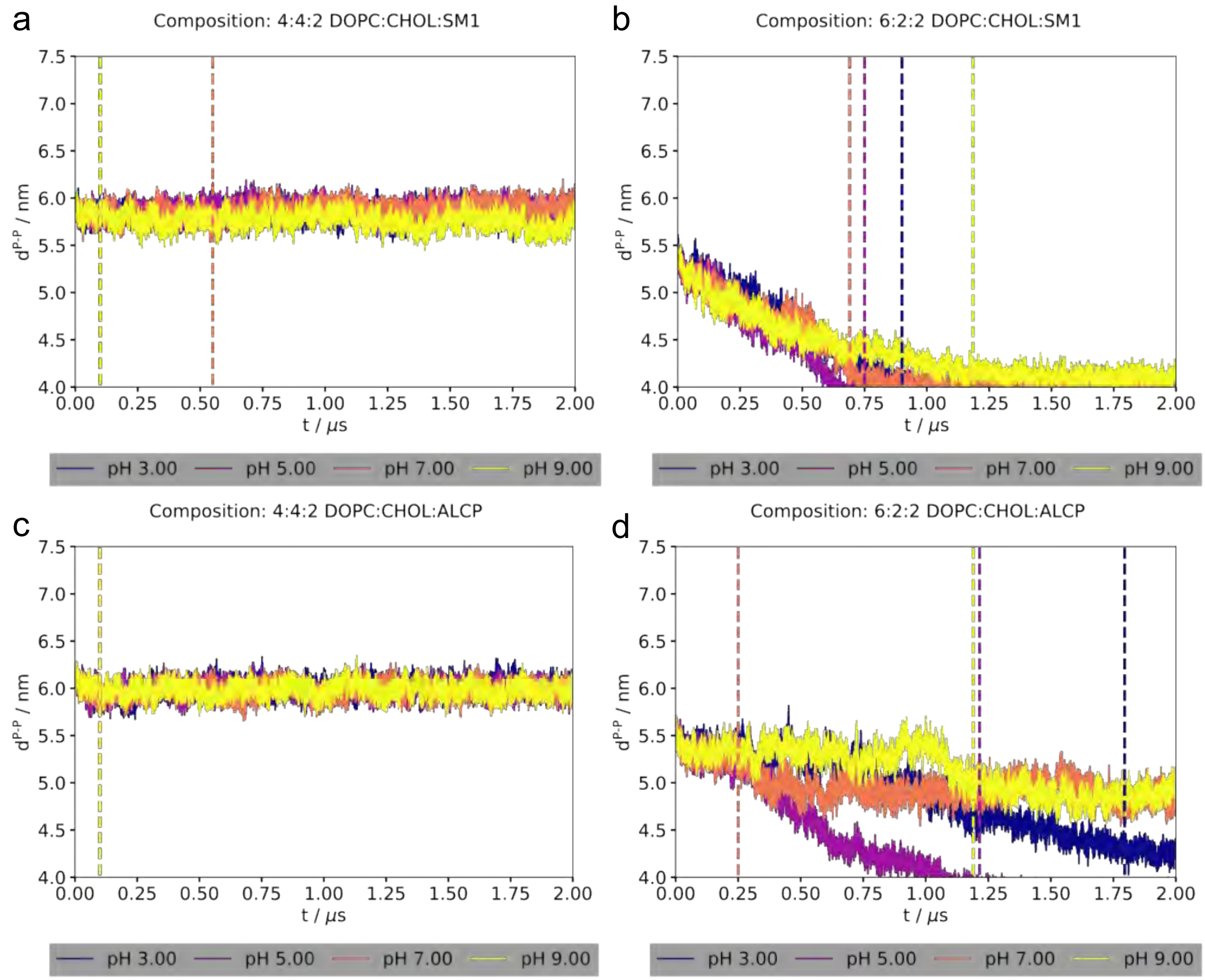

Figure S102: Membrane thickness per frame for systems with **a**,**c** a 4:4:2 or **b**,**d** a 6:2:2 helper lipid:cholesterol:aminolipid composition containing either **a**,**b** SM-102, or **c**,**d** ALC-0315 as the aminolipid. Simulations were setup by placing deprotonated aminolipids in the membrane core. Thickness is defined as the distance between the median z-positions (no trigonometric approach) of the phosphorus atoms in the upper and lower leaflets. Vertical dashed lines indicate the equilibration time determined for each pH value. Abbreviations used: ALCP = ALC-0315, SM1 = SM-102, CHOL = cholesterol.

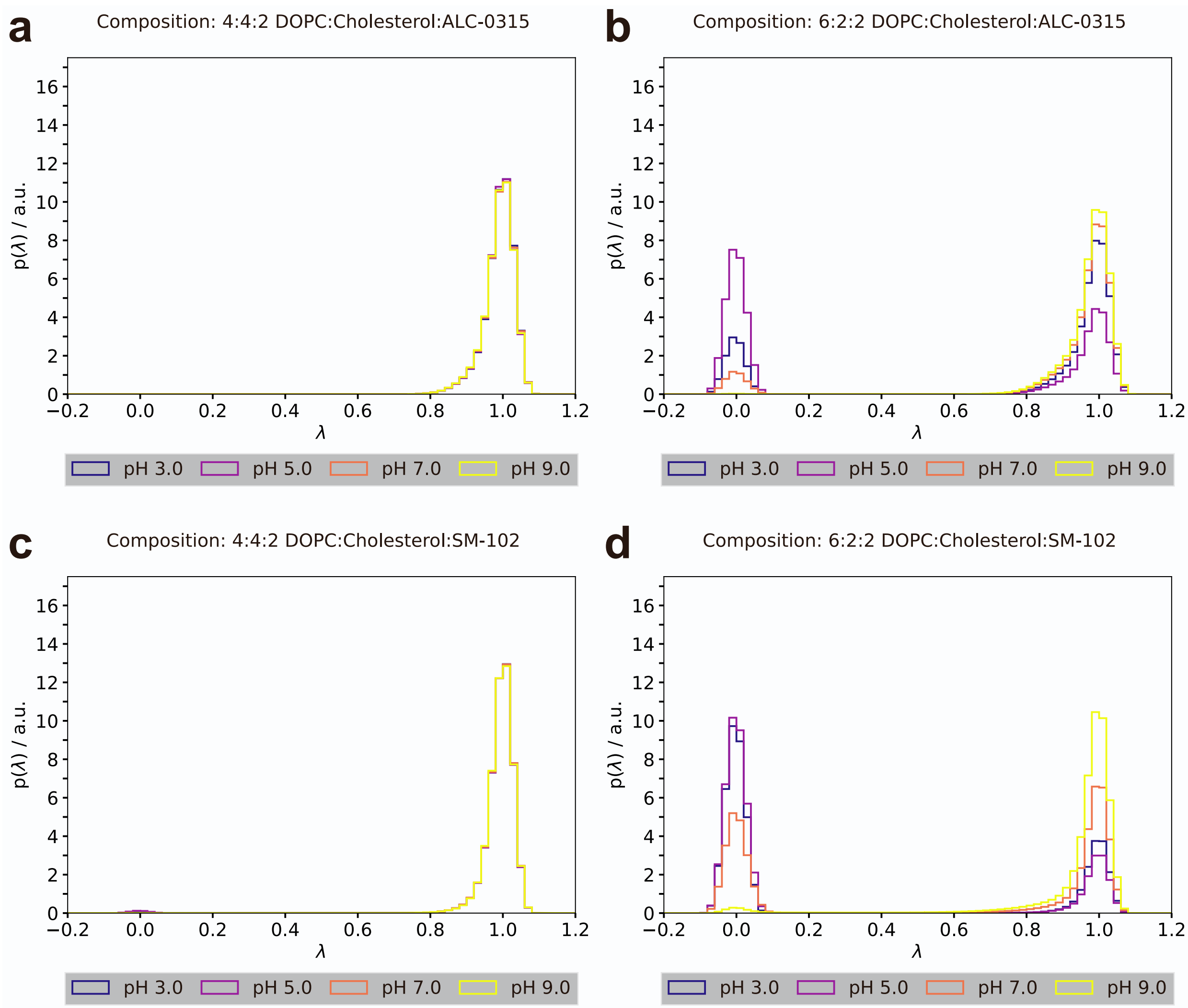

Figure S103: Distribution of the λ-coordinate at each pH, aggregated over all aminolipids in a membrane with **a**,**c** a 4:4:2 or **b**,**d** a 6:2:2 DOPC:cholesterol:aminolipid composition containing either **a**,**b** ALC-0315 or **c**,**d** SM-102 as the aminolipid. Simulations were setup by placing deprotonated aminolipids in the membrane core.

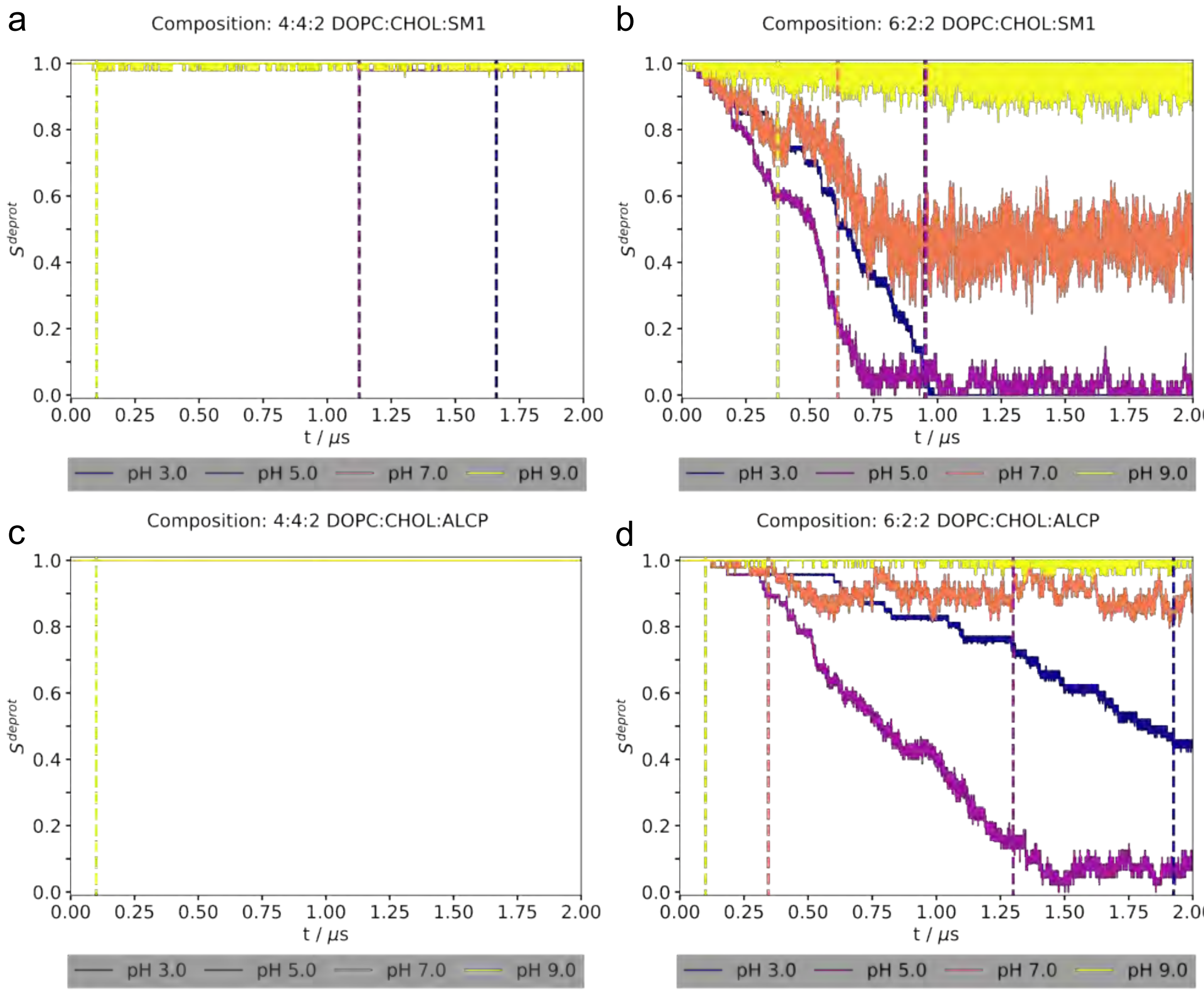

Figure S104: Fraction of deprotonated aminolipids (S^{deprot}) per frame for membranes with **a**,**c** a 4:4:2 or **b**,**d** a 6:2:2 DOPC:cholesterol:aminolipid composition containing either **a**,**b** ALC-0315 or **c**,**d** SM-102 as the aminolipid. Simulations were setup by placing deprotonated aminolipids in the membrane core. Vertical dashed lines indicate the equilibration time determined for each pH value. Abbreviations used: ALCP = ALC-0315, SM1 = SM-102, CHOL = cholesterol.

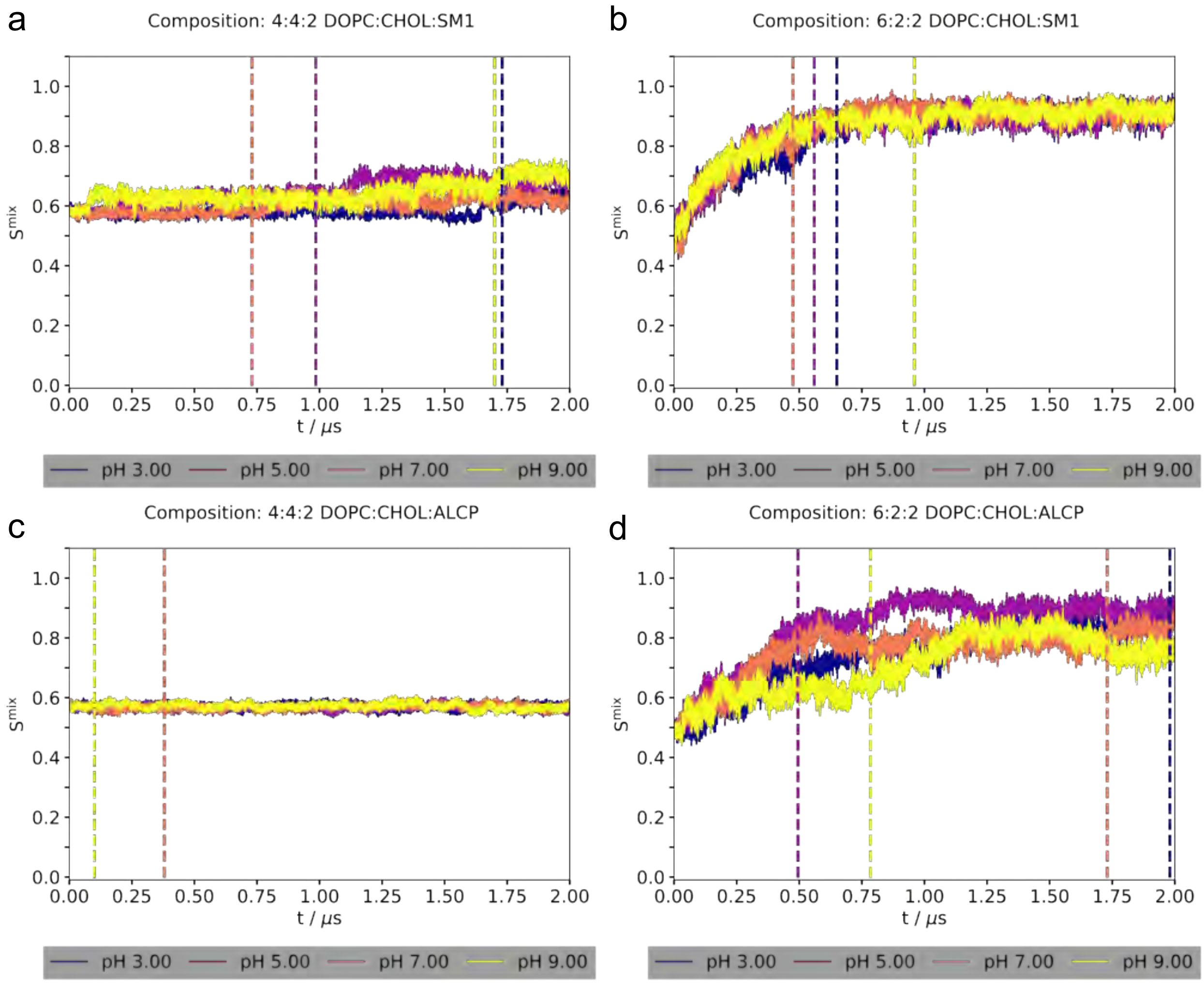

Figure S105: Conditional mixing entropy (S^{mix}) per frame for membranes with **a**,**c** a 4:4:2 or **b**,**d** a 6:2:2 DOPC:cholesterol:aminolipid composition containing either **a**,**b** SM-102 or **c**,**d** ALC-0315 as the aminolipid. Simulations were setup by placing deprotonated aminolipids in the membrane core. Vertical dashed lines indicate the equilibration time determined for each pH value. Conditional mixing entropies were calculated following Brandani *et al.*,[3] using a 1 nm cutoff to define neighboring lipids. Contacts were defined based on the distance between reference atoms in the headgroups of the target lipids: the titratable nitrogen for aminolipids, the oxygen atom fo cholesterol, and the sn-2 carbon atom for phospholipids. Abbreviations used: ALCP = ALC-0315, SM1 = SM-102, CHOL = cholesterol.

References

(1) Trollmann, M. F.; Rossetti, P.; Böckmann, R. A. Constant-pH MD Simulations of Lipids. *bioRxiv* **2024**, 2024–12.

(2) Trollmann, M. F. W.; Böckmann, R. A. Decoding pH-Driven Phase Transition of Lipid Nanoparticles. *Small* **2026**, e11381.

(3) Brandani, G. B.; Schor, M.; Macphee, C. E.; Grubmüller, H.; Zachariae, U.; Marenduzzo, D. Quantifying disorder through conditional entropy: an application to fluid mixing. *PLoS One* **2013**, *8*, e65617.
